# Community based complex interventions to sustain independence in older people: systematic review and network meta-analysis

**DOI:** 10.1136/bmj-2023-077764

**Published:** 2024-03-21

**Authors:** Thomas F Crocker, Joie Ensor, Natalie Lam, Magda Jordão, Ram Bajpai, Matthew Bond, Anne Forster, Richard D Riley, Deirdre Andre, Caroline Brundle, Alison Ellwood, John Green, Matthew Hale, Lubena Mirza, Jessica Morgan, Ismail Patel, Eleftheria Patetsini, Matthew Prescott, Ridha Ramiz, Oliver Todd, Rebecca Walford, John Gladman, Andrew Clegg

**Affiliations:** 1 Academic Unit for Ageing and Stroke Research (University of Leeds), Bradford Institute for Health Research, Bradford Teaching Hospitals NHS Foundation Trust, Bradford, UK; 2 Institute of Applied Health Research, College of Medical and Dental Sciences, University of Birmingham, Birmingham, UK; 3 Centre for Prognosis Research, School of Medicine, Keele University, Keele, UK; 4 Research Support Team, Leeds University Library, University of Leeds, Leeds, UK; 5 Geriatric Medicine, Bradford Teaching Hospitals NHS Foundation Trust, Bradford, UK; 6 Centre for Rehabilitation and Ageing Research, Academic Unit of Injury, Inflammation and Recovery Sciences, University of Nottingham, Nottingham, UK; 7 Health Care of Older People, Nottingham University Hospitals NHS Trust, Nottingham, UK

## Abstract

**Objective:**

To synthesise evidence of the effectiveness of community based complex interventions, grouped according to their intervention components, to sustain independence for older people.

**Design:**

Systematic review and network meta-analysis.

**Data sources:**

Medline, Embase, CINAHL, PsycINFO, CENTRAL, clinicaltrials.gov, and International Clinical Trials Registry Platform from inception to 9 August 2021 and reference lists of included studies.

**Eligibility criteria:**

Randomised controlled trials or cluster randomised controlled trials with ≥24 weeks’ follow-up studying community based complex interventions for sustaining independence in older people (mean age ≥65 years) living at home, with usual care, placebo, or another complex intervention as comparators.

**Main outcomes:**

Living at home, activities of daily living (personal/instrumental), care home placement, and service/economic outcomes at 12 months.

**Data synthesis:**

Interventions were grouped according to a specifically developed typology. Random effects network meta-analysis estimated comparative effects; Cochrane’s revised tool (RoB 2) structured risk of bias assessment. Grading of recommendations assessment, development and evaluation (GRADE) network meta-analysis structured certainty assessment.

**Results:**

The review included 129 studies (74 946 participants). Nineteen intervention components, including “multifactorial action from individualised care planning” (a process of multidomain assessment and management leading to tailored actions), were identified in 63 combinations. For living at home, compared with no intervention/placebo, evidence favoured multifactorial action from individualised care planning including medication review and regular follow-ups (routine review) (odds ratio 1.22, 95% confidence interval 0.93 to 1.59; moderate certainty); multifactorial action from individualised care planning including medication review without regular follow-ups (2.55, 0.61 to 10.60; low certainty); combined cognitive training, medication review, nutritional support, and exercise (1.93, 0.79 to 4.77; low certainty); and combined activities of daily living training, nutritional support, and exercise (1.79, 0.67 to 4.76; low certainty). Risk screening or the addition of education and self-management strategies to multifactorial action from individualised care planning and routine review with medication review may reduce odds of living at home. For instrumental activities of daily living, evidence favoured multifactorial action from individualised care planning and routine review with medication review (standardised mean difference 0.11, 95% confidence interval 0.00 to 0.21; moderate certainty). Two interventions may reduce instrumental activities of daily living: combined activities of daily living training, aids, and exercise; and combined activities of daily living training, aids, education, exercise, and multifactorial action from individualised care planning and routine review with medication review and self-management strategies. For personal activities of daily living, evidence favoured combined exercise, multifactorial action from individualised care planning, and routine review with medication review and self-management strategies (0.16, −0.51 to 0.82; low certainty). For homecare recipients, evidence favoured addition of multifactorial action from individualised care planning and routine review with medication review (0.60, 0.32 to 0.88; low certainty). High risk of bias and imprecise estimates meant that most evidence was low or very low certainty. Few studies contributed to each comparison, impeding evaluation of inconsistency and frailty.

**Conclusions:**

The intervention most likely to sustain independence is individualised care planning including medicines optimisation and regular follow-up reviews resulting in multifactorial action. Homecare recipients may particularly benefit from this intervention. Unexpectedly, some combinations may reduce independence. Further research is needed to investigate which combinations of interventions work best for different participants and contexts.

**Registration:**

PROSPERO CRD42019162195.

## Introduction

The global population aged over 65 years is expected to grow from 771 million people (10% of the population) in 2022 to 994 million by 2030 and 1.6 billion by 2050 (16%), necessitating change to healthcare systems.^1^ As the gap between life expectancy and healthy life expectancy grows, initiatives such as the World Health Organization’s Decade of Healthy Ageing seek to reverse this trend, maximising independence and social participation in later years as a shared global priority.^2^ ^3^ ^4^ This is important as an individual right, often diminished by ageism, but also to enable older people’s contribution to society and limit growing healthcare expenditure.^2^ ^5^ ^6^ ^7^

Community based complex interventions to support healthy ageing are diverse and may target individuals, their environment, or both. Such interventions typically target factors that contribute to health and wellbeing in older adults, aiming to maximise independence and quality of life. They involve a combination of approaches, which may be tailored to the needs and circumstances of the individual.

The 2023 Chief Medical Officer’s Report on Health in an Ageing Society identifies maximising independence for older people as a policy priority for England.^4^ ^8^ The report highlights an evidence gap regarding the effectiveness of complex interventions.^4^ Previous systematic reviews have indicated that, overall, such interventions probably have small but positive effects, despite some limitations in the underlying evidence.^9^ ^10^ ^11^ However, they have been unable to indicate which service models or components may be most effective. This field has seen further growth, and systematic review methods have developed strongly.^12^ ^13^ ^14^ Network meta-analysis extends traditional pairwise meta-analysis by comparing multiple interventions simultaneously in a single analysis, aiming to generate more precise results and rank them against each other to support policy and commissioning decisions.^15^ Network meta-analysis has not been used to evaluate community based complex interventions to sustain independence for older people. Therefore, we aimed to provide a rigorous, contemporary synthesis of trial evidence by using network meta-analysis to identify how interventions might best be configured to improve outcomes for older people and inform policy, commissioning, and delivery of evidence based services.

## Methods

This was a prospectively registered systematic review and network meta-analysis (PROSPERO CRD42019162195) that used Grading of Recommendations Assessment, Development and Evaluation (GRADE) for network meta-analysis to assess certainty^14^ ^16^ ^17^ ^18^ ^19^ ^20^ ^21^ and followed Preferred Reporting Items for Systematic Reviews and Meta-Analyses (PRISMA) 2020 and network meta-analysis guidelines.^22^ ^23^ We published the protocol before meta-analysis began.^24^ Only minor changes were made subsequently, detailed in appendix 1.

### Objectives

Our objectives were to identify randomised controlled trials and cluster randomised controlled trials of community based complex interventions to sustain independence in older people; synthesise evidence of their effectiveness for key outcomes in a meta-analysis of study level data; identify key intervention components and study level frailty to inform groupings for network meta-analysis and meta-regression; compare effectiveness of different intervention configurations by using network meta-analysis; and investigate the impact of frailty and pre-frailty by using meta-regression.

### Search strategy and selection criteria

Eligible studies were randomised controlled trials evaluating community based complex interventions to sustain independence in older people that met the criteria in table 1. DA searched the following databases and registers from inception between 9 and 11 August 2021: Cochrane Central Register of Controlled Trials (CENTRAL) (1992-); Medline (1946-); Embase and Embase Classic (1947-); CINAHL (1972-); PsycINFO (1806-); ClinicalTrials.gov (www.clinicaltrials.gov); and WHO International Clinical Trials Registry platform (https://trialsearch.who.int). Search strategies and their development are detailed in appendix 3. We also scanned reference lists of included reports (backward citation searches).

**Table 1:** Eligibility criteria for studies

Characteristic	Include	Exclude
Population	Older people (mean age ≥65 years) living at home at study entry	Participants living in residential care or nursing homes (care homes)^25^
Intervention	Initiated and mainly provided in the community	Interventions delivered in other settings (eg, outpatient, day hospital, intermediate care)
	Included two or more interacting components (intervention practices, structural elements, and contextual factors)	Interventions including only one discrete component (eg, exercise only)
	Targeted at the individual person, with provision of appropriate specialist care	Interventions not targeting the individual, such as general staff education or practice reorganisation
	Focused on sustaining (maintaining or improving) the person’s independence	Interventions not explicitly aimed at sustaining independence in activities of daily living
		Disease focused interventions (eg, for diabetes, depression)
		Falls prevention interventions
Comparators	Usual care, “placebo,” or attention control or a different complex intervention that met criteria	Non-complex interventions
Outcomes	Outcome domains did not form part of eligibility criteria	Outcome data were measured only before 24 weeks
Study design	Randomised controlled trials or cluster randomised controlled trials including all variants such as crossover, waiting list control, and stepped wedge designs	Post-crossover data due to likelihood of carryover. Studies for which only one unit (ie, individual or cluster) was randomised to an arm
Report characteristics	All reports regardless of publication status, date, or language	-

Duplicate records were removed with EndNote. Two reviewers independently used Rayyan (https://rayyan.ai/reviews) or Covidence (https://app.covidence.org/reviews) to evaluate eligibility of records (title and abstract) and, if potentially eligible, their reports (full text). We arranged translation as necessary. Disagreements were resolved by consensus, with guidance from the Project Management Group.

### Data collection

Two reviewers independently extracted data in a custom built Microsoft Access database, with data finalised automatically in case of agreement or resolved by a third reviewer. The main outcomes were living at home, activities of daily living (instrumental/personal), hospital admission, care home placement, homecare services usage, costs, and cost effectiveness. Additional outcomes were health status, depression, loneliness, falls, and mortality.

Data were extracted (including treatment effect estimates) and categorised into three timeframes: short term (around six months): 24 weeks to 9 months; medium term (around 12 months): >9 months to 18 months; and long term (around 24 months): >18 months. Medium term was our main timeframe.

Other data items collected (including design and participant details) are listed in appendix 4. We did not routinely seek missing data but sought to clarify ambiguities.

### Assessment of frailty

Two reviewers with extensive clinical-academic frailty expertise (AC and JGl) independently categorised study level frailty (robust, pre-frailty, frailty) on the basis of validated measures where available or participants’ characteristics and study inclusion criteria using the phenotype model as a framework.^26^

### Intervention classification

We grouped all eligible interventions (including comparators) in preparation for network meta-analysis in a three stage process. Firstly, one reviewer (MJ, NL, RR, LM, IP, or EP) coded and summarised each intervention against the Template for Intervention Description and Replication items,^27^ ^28^ and TC checked both coding and summaries, with disagreements resolved through discussion and involvement of the Project Management Group as necessary. Secondly, we generated categories of key intervention features through qualitative analysis, iteratively consolidating codes into categories. Thirdly, we grouped interventions on the basis of these categories, using a typology developed through discussion between reviewers, the Project Management Group, and experts including policy makers, commissioners, older people, and carers. The intervention groups became the network meta-analysis nodes.

### Risk of bias

Two reviewers independently assessed risk of bias in each result of interest from each study, using the revised Cochrane risk of bias tool for randomised trials (RoB 2).^12^ ^29^ Our effect of interest was assignment to the intervention (intention to treat). We rated risk of bias per domain and overall as low, some concerns, or high (serious or very serious concerns), through consensus between reviewers.

### Data synthesis

We did separate meta-analyses for each timeframe for living at home (dichotomous), personal activities of daily living (continuous), instrumental activities of daily living (continuous), and care home placement (dichotomous) and in the medium term for hospital admission (dichotomous), health status (continuous), and depression (continuous). We narratively synthesised other outcomes owing to a lack of data suitable for meta-analysis. We preferred adjusted effect estimates but calculated effect estimates from extracted data where necessary and possible, applying cluster adjustment where applicable.

We meta-analysed the effect estimates by using Stata modules including metan, mvmeta, and network.^30^ We synthesised dichotomous outcomes as log transformed odds ratios.^31^ For continuous outcomes, we used Hedges’ g standardised mean difference. We did random effects meta-analyses.^32^

Initially, for each outcome and timeframe, we did a separate meta-analysis for each type of intervention versus control, to provide summary effectiveness results and forest plots based only on direct evidence. We then used network meta-analysis to compare relative effectiveness of all intervention types concurrently, combining direct evidence with indirect evidence, which is based on a network of intermediate comparisons between intervention types.^15^ We did network meta-analysis (for each outcome and timeframe separately) by using a multivariate random effects meta-analysis framework via the network module in Stata using restricted maximum likelihood estimation.^30^ We produced summary (pooled) effect estimates for each pair of treatments in the network, with 95% confidence intervals and network plots. On the basis of the results, we calculated the ranking of intervention groups by using re-sampling methods. To facilitate interpretation, we transformed each summary odds ratio to a summary risk ratio by using the median risk in the reference comparator arms and corresponding absolute intervention risks and risk differences by using the highest and lowest risk among reference comparator arms (n≥100) as the assumed comparator risks.^33^ We re-expressed standardised mean differences as the mean difference by using a pooled standard deviation for a common measure of the outcome.

We examined the consistency assumption (that direct and indirect evidence are consistent with each other) for each treatment comparison where possible and across the whole network.^34^ ^35^ We summarised heterogeneity by the estimate of between study variance (τ), where possible.

We examined the effect of study level frailty on each intervention group effect where data allowed by using network meta-regression. We did sensitivity analyses excluding results rated as at very serious risk of bias. We examined funnel plots for small study effects if 10 or more studies were available.

We used the GRADE framework, adapted for network meta-analysis, to rate the certainty of the results of our network meta-analysis and presented summary of findings tables ordered by certainty and ranking.^14^ ^16^ ^17^ ^18^ ^19^ ^21^ We used plain language terms “probably” and “may” to indicate moderate and low certainty respectively and qualified the size and direction of point estimates (see appendix 5).^20^ ^36^ We followed the brief economic commentary framework to summarise and compare the principal economic findings reported by included studies.^37^

### Patient and public involvement

This review benefited from the involvement of our established Patient and Public Involvement Frailty Oversight Group in the Bradford Institute for Health Research. The group has a structure that provides connections to the whole spectrum of older people, with a focus on those living with frailty to enable meaningful, public involvement in our research projects.^38^ We consulted our Frailty Oversight Group throughout the development of the protocol and discussed plans in detail at the group’s quarterly meetings and at our annual consumer research conference. Group members helped to draft and revise the plain language summary for our funding application. Other examples of patient and public involvement include the selection of important outcomes and their prioritisation as main and additional outcomes. Frailty Oversight Group members emphasised that a wide range of outcomes were important to older people, with a particular focus on independence in addition to wellbeing, alongside service orientated outcomes. We also spent time discussing the intervention components that we had identified with group members. Through this work, we developed and refined our descriptions of the components and thus the findings. The plain language summary of our findings, developed with Frailty Oversight Group members, is presented in appendix 2.

## Results

### Study selection

**Fig 1:**
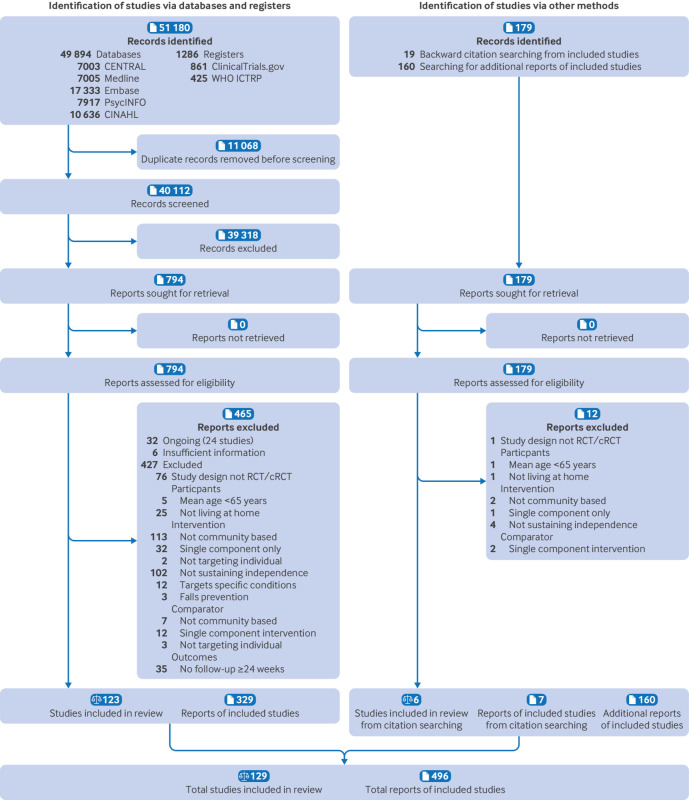
PRISMA flow diagram showing identification, selection, and inclusion of studies from databases, registers, and other sources.^23^ ICTRP=International Clinical Trials Registry Platform

Figure 1 shows the study selection process. Our database and register searches identified 51 180 records. After de-duplication, we screened 40 112 records and then assessed 794 reports for eligibility. Subsequently, we assessed 179 reports identified through searching for additional reports of included studies and backward citation searches. We excluded 477 reports; reasons are listed in appendix 6. We included 129 studies with 266 eligible intervention arms presented in 496 reports^39^ ^40^ ^41^ ^42^ ^43^ ^44^ ^45^ ^46^ ^47^ ^48^ ^49^ ^50^ ^51^ ^52^ ^53^ ^54^ ^55^ ^56^ ^57^ ^58^ ^59^ ^60^ ^61^ ^62^ ^63^ ^64^ ^65^ ^66^ ^67^ ^68^ ^69^ ^70^ ^71^ ^72^ ^73^ ^74^ ^75^ ^76^ ^77^ ^78^ ^79^ ^80^ ^81^ ^82^ ^83^ ^84^ ^85^ ^86^ ^87^ ^88^ ^89^ ^90^ ^91^ ^92^ ^93^ ^94^ ^95^ ^96^ ^97^ ^98^ ^99^ ^100^ ^101^ ^102^ ^103^ ^104^ ^105^ ^106^ ^107^ ^108^ ^109^ ^110^ ^111^ ^112^ ^113^ ^114^ ^115^ ^116^ ^117^ ^118^ ^119^ ^120^ ^121^ ^122^ ^123^ ^124^ ^125^ ^126^ ^127^ ^128^ ^129^ ^130^ ^131^ ^132^ ^133^ ^134^ ^135^ ^136^ ^137^ ^138^ ^139^ ^140^ ^141^ ^142^ ^143^ ^144^ ^145^ ^146^ ^147^ ^148^ ^149^ ^150^ ^151^ ^152^ ^153^ ^154^ ^155^ ^156^ ^157^ ^158^ ^159^ ^160^ ^161^ ^162^ ^163^ ^164^ ^165^ ^166^ ^167^; 90 studies contributed to network meta-analyses.

### Included study characteristics

The 129 studies assigned 74 946 participants (reported by 126 studies; 61% (44 817/72 877) women and 39% (28 060/72 877) men, from 123 studies). Studies were predominantly conducted in developed countries, and most participants were described as white. Nevertheless, the overall population included a broad range of demographic characteristics. Study populations included all frailty levels. Study characteristics are summarised in appendix 7. We judged most results to be at high risk of bias, primarily owing to missing outcome data (appendix 8).

### Intervention characteristics

We identified 19 separate components of included interventions (see box 1 and appendix 9), which were evaluated in 63 combinations including the absence of all these components, which we termed “available care.” “Homecare” was another common control group in populations in which all participants were receiving formal homecare. Homecare was one of 14 action components (further examples include education and exercise). Five other components primarily involved a tailoring process of ascertainment or assessment and planning with potential for subsequent multifactorial action, most often “multifactorial action” from care planning (a process of individualised multidomain assessment and management) with or without routine “review” (scheduled, regular follow-ups). Multifactorial action was further delineated according to the presence or absence of an embedded medication review and specific self-management strategies. Twenty six intervention groups (combinations) were evaluated in more than one study, and these are summarised in relation to Template for Intervention Description and Replication items in appendix 10.

Box 1 Nineteen identified components of community based complex interventions* intended to sustain independence in older people

**Action components**

“Activities of daily living training”
Providing “aids” and adaptations
“Alternative medicine”
“Care voucher” provision
“Cognitive training”
Health “education”
Physical “exercise”
Formal “homecare”
Engagement in “meaningful activities”
“Nutrition”(al) support
Psychological (mood) therapy (“psychology”)
“Social skills” training
Technology for communication and engagement (“telecoms”)
“Welfare” rights advice.

**Tailoring components**

“Multifactorial action” from individualised care planning
Routine “review”
“Medication review”
“Monitoring”
Routine “risk screening”

*Text in quotation marks is short version of name used in results

### Characteristics of network meta-analyses

**Fig 2:**
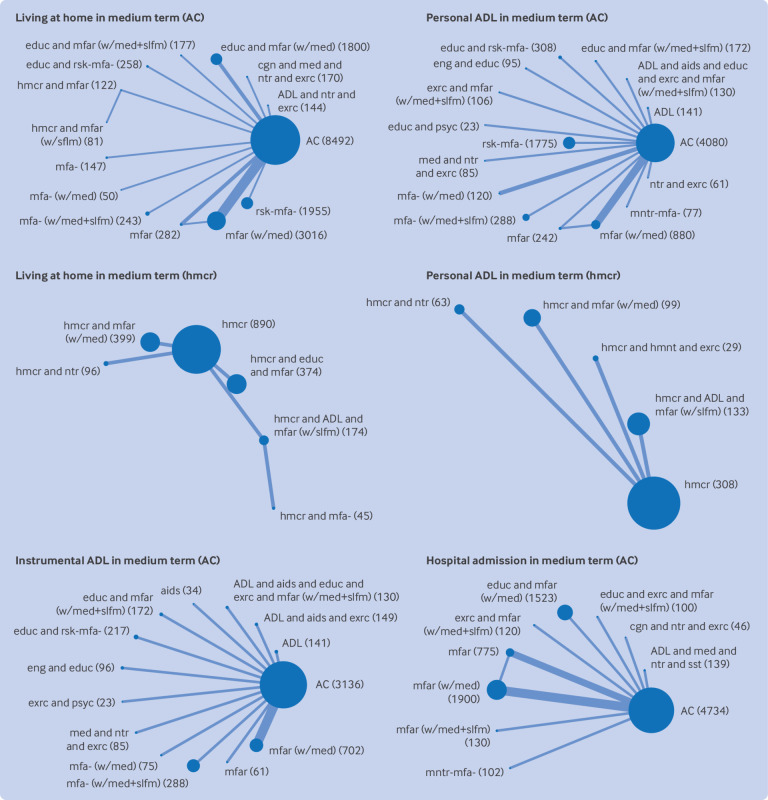
Network plots for analyses of main outcomes in medium term (~12 months) that yielded ≥1 finding of at least low certainty. AC indicates network including available care; hmcr indicates network including formal homecare. Each node is labelled with intervention group abbreviation and number of participants. Node size is proportionate to number of participants; edge thickness is proportionate to number of comparisons. Intervention and control group abbreviations are combination of: ADL=activities of daily living training; aids=provision of aids and adaptations; cgn=cognitive training; comm=technology for communication and engagement; educ=health education; eng=engagement in meaningful activities; exrc=physical exercise; hmcr=formal homecare; hmnt=alternative medicine; med=medication review; mfa=multifactorial action; mfar=multifactorial action and follow-on routine review; mntr-mfa=monitoring, which may trigger multifactorial action; ntr=nutritional support; psyc=psychological therapy; rsk-mfa=risk screening, which may trigger multifactorial action; sst=social skills training; vchr=care voucher provision; wlfr=welfare rights advice; w/med=with medication-review; w/slfm=with self-management strategies

**Fig 3:**
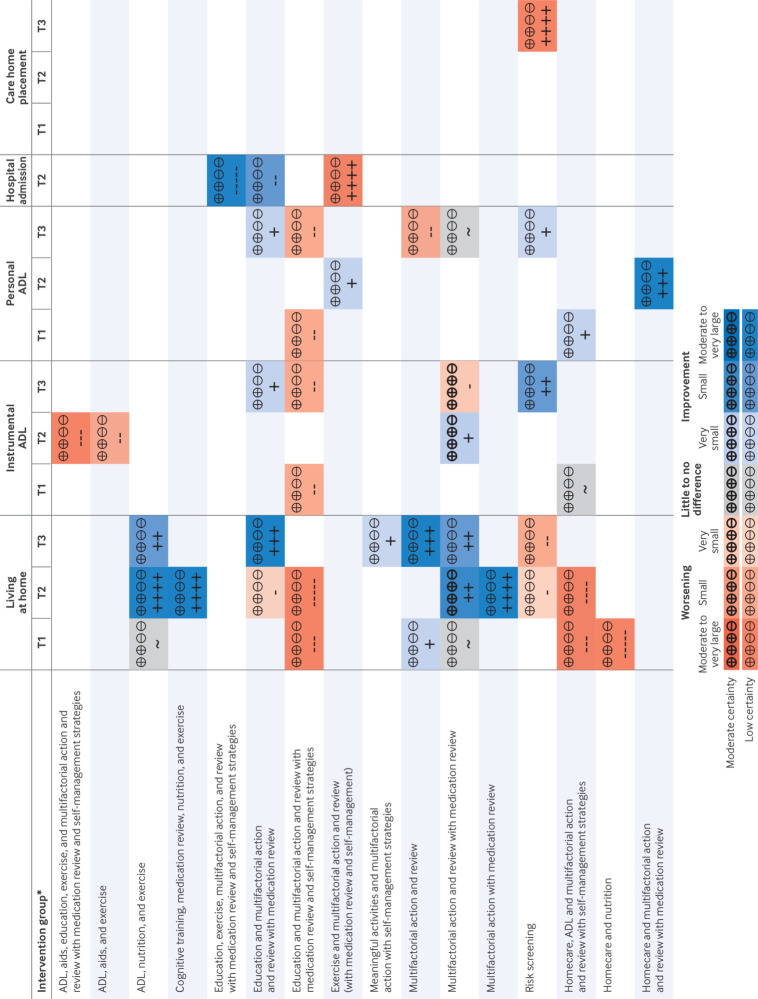
Summary of moderate and low certainty evidence for main outcomes synthesised with network meta-analysis. *In comparison with reference comparator. For intervention groups including homecare, reference comparator is homecare; for all other intervention groups, reference comparator is available care. Living at home (+ favoured); instrumental ADL (+ favoured); personal ADL (+ favoured); hospital admission (– favoured); care home placement (– favoured). ADL=activities of daily living; T1=short term timeframe (24 weeks to 9 months); T2=medium term timeframe (>9 months to 18 months; T3=long term timeframe (>18 months); +++++=very large increase; ++++=large increase; +++=moderate increase; ++= slight increase; +=very slight increase; ~=little to no difference; -=very slight reduction;--=slight reduction; ---=moderate reduction; ----=large reduction; -----=very large reduction. Blue shades indicate possible benefit; orange shades indicate possible harm; bold indicates moderate certainty evidence

Most networks were small and sparse, with few included studies contributing to most networks (fig 2). We found little evidence of inconsistency or small study effects, but the power to detect this was usually low. All outcomes except mortality needed to be analysed in two separate network meta-analyses, as the networks were disconnected: one with available care as the reference comparator (“available care network”) and one with homecare as the reference comparator (“homecare network”). Estimates are reported here only in comparison with the reference comparator. Comparison with available care is the effect of adding the intervention for a population who are not all receiving any particular care; comparison with homecare is similarly an alternative intervention for a population already in receipt of homecare without associated reablement or multifactorial action from care planning. Most estimates were of low certainty or very low certainty owing to risk of bias, imprecision, or their combination, and we do not detail very low certainty estimates below. Full results are presented in appendix 11 and summarised in figure 3.

### Living at home

**Fig 4:**
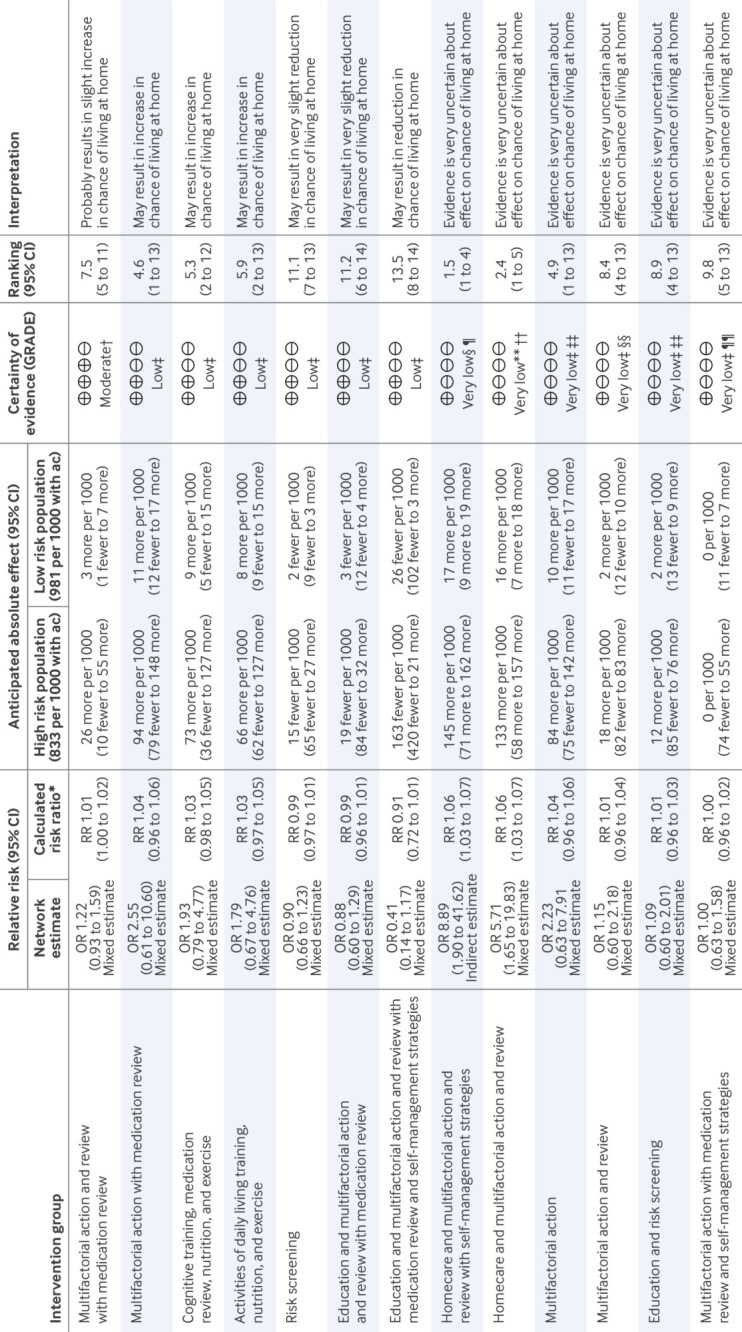
Living at home in medium term: comparisons with available care summary of findings table. Summary of findings table shows relative effects and anticipated absolute effects of each community based complex intervention type compared with available care (ac) for living at home outcome in medium term. Living at home is binary outcome, alternative outcome being either care home placement or death. Relative effects are odds ratio (OR) from network meta-analysis (NMA) and risk ratio (RR) calculated from odds ratio. OR>1 (or RR>1) favours listed community based complex intervention; OR<1 favours available care. Intervention types are ordered by certainty of evidence (high to very low) and ranking (highest to lowest). NMA included 21 studies, 14 nodes, and 16 937 participants in total. Follow-up ranged from 12 to 18 months. Heterogeneity was estimated as τ=8.56×10^-2^. Consistency assumption held. Mean rank of available care was 9.9 (95% CI 7 to 12). *Calculated from OR and assumed comparator risk of 0.935, median available care risk among these studies. †Serious concerns about imprecision as confidence interval (CI) crosses no effect line and includes substantial benefit. CI for absolute effect with high risk population also includes pre-specified definition of very small harm, but given that this was marginal and in light of small lower CI for RR (0.9955), this was not judged as very serious. Downgrade once. ‡Very serious concerns about imprecision as CI includes substantial benefit and substantial harm. Downgrade twice. §Very serious concerns about risk of bias owing to exclusion of participants in per protocol analysis and missing outcome data in indirect evidence via homecare and multifactorial action and review versus available care comparison. Downgrade twice. ¶Very serious concerns about imprecision as CI is very wide, no closed loop exists, and direct comparison is based on indirect evidence from 122 people in homecare and multifactorial action and review and 81 people in homecare and multifactorial action and review with self-management, which does not meet optimal information size. Already downgraded twice for risk of bias; downgrade once. **Very serious concerns about risk of bias owing to exclusion of participants in per protocol analysis and missing outcome data. Downgrade twice. ††Very serious concerns about imprecision as CI is very wide, no closed loop exists, and direct comparison is based on indirect evidence from 122 people in homecare and multifactorial action and review, which does not meet optimal information size. Already downgraded twice; downgrade once. ‡‡Serious concerns about risk of bias owing to missing outcome data. Downgrade once. §§Serious concerns about risk of bias owing to recruitment process of participants and missing outcome data in one study. Downgrade once. ¶¶Very serious concerns about risk of bias owing to randomisation process and missing outcome data. Already downgraded twice; downgrade once

For living at home in the medium term, the available care network included 21 studies (n=16 937) with 14 intervention groups (fig 4). We found moderate certainty evidence that “multifactorial action and review with medication review” probably results in a slight increase in the odds of living at home (odds ratio 1.22, 95% confidence interval 0.93 to 1.59). We found low certainty evidence that “multifactorial action with medication review” (odds ratio 2.55 (large), 0.61 to 10.60), “cognitive training, medication review, nutrition, and exercise” (1.93 (large), 0.79 to 4.77), and “activities of daily living training, nutrition, and exercise” (1.79 (large), 0.67 to 4.76) may result in an increase in the odds of living at home and that “risk screening” (0.90 (very small), 0.66 to 1.23), “education and multifactorial action and review with medication review” (0.88 (very small), 0.60 to 1.29), and “education, multifactorial action, and review with medication review and self-management strategies” (0.41 (very large), 0.14 to 1.17) may each result in some reduction in the odds of living at home. Other comparisons were very low certainty.

In the short term and long term timeframes, results were low certainty at best. For “multifactorial action and review with medication review” and “activities of daily living traning, nutrition, and exercise,” estimates were similarly of small increases in the long term but of little to no difference in the short term. For “education, multifactorial action, and review with medication review and self-management strategies” and “risk screening,” we found similar results in other timeframes (may reduce living at home), but for “education and multifactorial action and review with medication review,” we found contrasting evidence of an increase in living at home.

The homecare network for living at home was smaller (five studies; n=1978 in the medium term). In the short and medium term timeframes we found low certainty evidence that “homecare, activities of daily living traing, and multifactorial action and review with self-management strategies” may result in reductions (short term, odds ratio 0.63 (moderate), 0.31 to 1.26; medium term, 0.76 (large), 0.40 to 1.45) in the odds of living at home compared with “homecare” alone. In the short term, “homecare and nutrition” may result in reductions in the odds of living at home compared with “homecare” (odds ratio 0.34 (very large), 0.12 to 0.95). Other comparisons were of very low certainty.

### Instrumental activities of daily living

The medium term instrumental activities of daily living available care network included 16 studies (n=5309) with 14 intervention groups. We found moderate certainty evidence that “multifactorial action and review with medication review” was associated with very slightly increased independence in instrumental activities of daily living versus “available care” (standardised mean difference 0.11, 95% confidence interval 0.00 to 0.21). Two intervention groups may result in some reduction in instrumental activities of daily living: “activities of daily living training, aids, and exercise” and “activities of daily living training, aids, education, exercise, and multifactorial action and review with medication review and self-management strategies.” The findings for “multifactorial action and review with medication review” in the long term were contrasting, with moderate certainty evidence of a very slight reduction in instrumental activities of daily living (standardised mean difference −0.08, −0.21 to 0.05).

For the homecare network, we had one low certainty finding in the short term timeframe of little to no difference for “homecare, activities of daily living training and multifactorial action and review with self-management strategies,” with all other comparisons being of very low certainty.

### Personal activities of daily living

For personal activities of daily living, 20 trials (n=8583 participants) with 16 intervention groups contributed to the medium term available care network. One comparison was low certainty: “exercise and multifactorial action and review with medication review and self-management startegies” may result in a very slight increase in personal activities of daily living (standardised mean difference 0.16, −0.51 to 0.82).

The homecare network included four trials (n=632 participants) in the medium term. Here too, only one comparison with “homecare” was low certainty: “homecare and multifactorial action and review with medication review” may result in an increase in personal activities of daily living (standardised mean difference 0.60 (moderate), 0.32 to 0.88).

### Other main outcomes

For the service outcome of hospital admission, we found low certainty estimates of some reductions for “education, exercise, and multifactorial action and review with medication review and self-management strategies” and “education and multifactorial action and review with medication review” and of an increase for “exercise, multifactorial action, and review with medication review and self-management strategies.” For care home placement, all estimates were rated as very low certainty in the medium term. We found some evidence of both increases and decreases in use of homecare services with little pattern (not meta-analysed).

The summary of economic evidence included 39 studies (appendix 12). On the basis of the conclusions of 22 studies that did a full economic evaluation, five intervention groups seemed promising compared with a standard intervention or available care from an economic perspective: “activities of daily living training” (medium term time horizon); “homecare and multifactorial action and review with medication review and self-management strategies” (short term time horizon); “meaningful activities and education” (short and medium term time horizon); “multifactorial action and review with medication review” (short term but not medium or long term time horizon); and “exercise and multifactorial action with medication review” (long term time horizon).

### Additional outcomes

Additional outcomes are summarised in appendix 13. We found little evidence of any effect on self-reported health status, only low certainty beneficial findings regarding depression, and very little evidence regarding loneliness; more complex interventions were associated with less falling than with more falling (12 *v* 4 studies). For mortality, we had a large network of 65 studies (n=38 351) and 41 intervention groups. We found low certainty evidence of reductions for two intervention groups and increases for five intervention groups.

### Investigations of frailty and risk of bias

Across all outcomes, risk of bias sensitivity analyses produced very similar estimates to the main analyses. Funnel plots showed little evidence of asymmetry. Network meta-regressions were unable to estimate the effects of frailty for many comparisons, and those that were estimable had very wide confidence intervals.

## Discussion

### Principal findings

Our review has found evidence that “multifactorial action and review with medication review” probably improves the odds of living at home in the medium term timeframe (around 12 months). We also found that other complex interventions (“multifactorial action with medication review”; “cognitive training, medication review, nutrition, and exercise”; and “activities of daily living training, nutrition, and exercise”) may result in an increase in the odds of living at home in this timeframe. For 12 other intervention groups, we found low certainty evidence that they may improve or worsen at least one main outcome, but for other intervention groups evidence was either absent or very uncertain.

We identified moderate certainty evidence in three of our analyses of main outcomes, all related to “multifactorial action and review with medication review.” In comparison with “available care,” we found a probable slight increase in living at home in the medium term and a very slight increase in independence in instrumental activities of daily living in the medium term, but in contrast also a very slight reduction in instrumental activities of daily living in the long term (fig 3). The direct evidence for probable worsening came from studies that also contributed to the beneficial medium term finding (tables 28 and 32 and figures 8 and 14, appendix 11).^49^ ^131^ For homecare recipients, the addition of “multifactorial action and review with medication review” was associated with low certainty evidence of a moderate increase in personal activities of daily living in the medium term (fig 3).

### Strengths and limitations of review

We used rigorous methods to summarise the contemporary evidence on community based complex interventions for older people as an area of high strategic priority and global policy relevance. The evidence originated from a diverse population of older people, albeit mainly from high income regions. Population subgroups of various socioeconomic status, frailty levels, and ethnicity were included, and the ratio of women to men seemed typical for older populations.^1^ People with greater resource may be over-represented in many of these trials, given the voluntary nature of participation, which may have limited the effects of interventions and hence contributed to the small effect sizes presented here.

We developed a data driven grouping of interventions with expert guidance, so the groups were clearly specified to support practice, commissioning, and policy decisions. Our approach has resulted in a very clear typology of interventions, avoiding the historical problem of combining different interventions under unhelpful umbrella terms. We acknowledge that some clinical heterogeneity remains within intervention groups, particularly in the care available to different study populations. However, application of this typology led to the separation of evidence from populations in receipt of homecare and those that were not, reducing heterogeneity and improving the specificity of recommendations.

For consistency, we limited our review to interventions focused explicitly on independence in activities of daily living. Although this differs from preventing institutionalisation or activity limitation, multifactorial action interventions focused on each of these may work similarly. As such interventions can have multiple foci, differences may reflect intervention reporting rather than design. A less rigorous application of the criteria may have enabled us to pool similar evidence from more trials.^168^ ^169^

Many of the trials seemed to be well conducted under challenging circumstances, and most declared non-commercial support, limiting the risks of funding bias. However, most results were at some risk of bias owing to missing outcome data. This was often inevitable given the combination of a frail population, long timelines, self-reported outcomes, community based research, and our stringent application of the RoB 2 tool. Many of the identified risks of within study bias were towards no effect or favouring the control group, meaning that true intervention effects may have been underestimated.

Owing to the scarcity of both direct and indirect evidence, the power to examine heterogeneity and inconsistency was low in most networks. However, the assessments we were able to do generally indicated little heterogeneity or inconsistency. We were also unable to effectively investigate the effect of population frailty on intervention effects, because of a lack of comparisons that contained different frailty populations.

We carefully assessed certainty in the findings by using GRADE for network meta-analysis guidance, providing clarity for the reader about the strength of the results. For many estimates, certainty was reduced because the confidence intervals included both improvement and worsening. Therefore, some low certainty findings may represent the play of chance around no effect.

### Comparison with other studies

Whereas other reviews of similar literature have tended to identify positive effects, albeit small and with some limitations in the underlying evidence,^9^ ^10^ ^11^ this review has found evidence of both positive and negative effects, depending on the intervention type (fig 3). This difference is likely to reflect the broad pairwise pooling of comparisons in other reviews, with different combinations of intervention components, different comparators, and heterogeneous effects and time points. These other reviews also did not use GRADE to assess the certainty of the evidence or the RoB 2 tool to assess risk of bias and may therefore have been less likely to conclude that a statistically significant finding was nevertheless very uncertain.

Two recently published effectiveness trials have continued the trend of many previous studies in finding no significant difference in outcomes of interest.^170^ ^171^ Given that many of the effects estimated in this review were relatively small, future definitive trials of similar interventions will likely need to be much larger than most of those included to provide sufficient power to identify plausible effects on independence.

### Conclusions, policy implications, and future research

Overall, our best evidence of combinations associated with sustained independence are service models for older people that incorporate care planning leading to multifactorial action with embedded medication review. The strongest evidence also favoured services incorporating ongoing review of the older person. The combination of exercise and nutritional support was part of two additional favourable intervention groups. Given the overall evidence for their benefits, we recommend access to these interventions in services for older people.^172^ ^173^

Unexpectedly, we found evidence that some intervention combinations may reduce independence. This finding was not one that we anticipated but is not one that should be dismissed entirely, despite uncertainty in the evidence. Plausible mechanisms depend on the intervention group details but include invoking disengagement with the person’s health or with services or encouraging an individual to take on more than they can effectively manage. It could also be the case that certain events, such as care home placement or hospital admission, may be part of the best care strategy for an individual. Even deterioration in activities of daily living may be in response to provision of assistance for tasks that someone finds difficult or painful and otherwise unrewarding and may therefore be an acceptable trade-off. We recommend that practitioners, commissioners, and policy makers remain mindful of these possibilities when designing systems of care.

Despite a very large amount of primary research (129 studies; 74 946 participants) a degree of uncertainty exists about the effects of many types of intervention, although this does not mean that they are ineffective. Although methodological improvements in the primary research could further enhance knowledge, consideration of the scale of effort that would be needed to begin to complete this network of interventions is worthwhile. Therefore, whether embarking on a programme of large scale randomised controlled trials to further examine all interventions would be an effective use of research resources is unclear. Value of information analysis, which aims to quantify the value of new research to examine existing uncertainties, may be a helpful next step to prioritise which interventions to take forward to new trial based evaluation.^174^ Additionally, conducting individual participant data meta-analysis and realist synthesis may be useful to better explore the factors that may relate to benefit.^175^

## What is already known on this topic

Older people prioritise maintenance of independence
Previous systematic reviews have suggested that community based complex interventions may support independence for older people, but which are most effective is unclear
The lack of clear guidance about which services to implement has hampered translation of evidence into policy and practice

## What this study adds

Individualised care planning with tailored actions, including medicines optimisation and regular follow-ups, probably helps people to stay living at home
Although some complex interventions may sustain independence, others may reduce independence
Further evidence is needed about who benefits most from which kinds of interventions, which may be provided by individual participant data meta-analysis

## Data Availability

The data associated with this paper will be openly available indefinitely upon publication under a Creative Commons attribution license from the University of Leeds Data Repository. Summary effect estimates and findings from network meta-analyses: https://doi.org/10.5518/1377; risk of bias judgments: https://doi.org/10.5518/1386.

## Web extra

Extra material supplied by authors

Web appendix: Appendix 1-13

## Notes

Contributors: AC, TC, JGl, and RRi conceived of the study. AC, TC, JE, AF, JGl, MJ, NL, EP, and RRi designed the study. DA, TC, and NL developed the search strategy. DA executed the database and trial register searches. TC, JGr, MJ, NL, JM, EP, RRa, and RW selected the studies. RB, CB, TC, JGr, MH, MJ, NL, JM, LM, EP, IP, RRa, OT, and RW extracted data. AC and JGl assessed frailty. AC, TC, AE, AF, JGl, MJ, and NL did intervention grouping. CB, TC, MJ, NL, and MP assessed risk of bias. MJ liaised with patient and public involvement groups. RB, MB, and JE prepared and analysed effectiveness data. JE and RRi supervised the meta-analyses. TC and NL assessed the certainty of the evidence. TC and NL did economic and narrative synthesis. AC, TC, JE, AF, JGl, NL, RRi, and OT interpreted the findings. TC wrote the first draft of the manuscript with input from AC, JE, AF, and JGl. All authors contributed to critically reviewing or revising the manuscript. CB, MB, RB, AC, TC, JE, JGl, JGr, MH, MJ, NL, JM, LM, EP, IP, MP, RRa, OT, and RW collectively accessed and verified the underlying data reported in the manuscript. JE and NL contributed equally. TC is the guarantor. The corresponding author attests that all listed authors meet authorship criteria and that no others meeting the criteria have been omitted.

Transparency: The lead author (the manuscript's guarantor) affirms that the manuscript is an honest, accurate, and transparent account of the study being reported; that no important aspects of the study have been omitted; and that any discrepancies from the study as originally planned (and, if relevant, registered) have been explained.

Dissemination to participants and related patient and public communities: To ensure that we clearly communicate our findings with patients and member of the public, we spent time discussing the intervention components we had identified with Frailty Oversight Group members. Through this work, we developed and refined our descriptions of the components. Frailty Oversight Group members helped to draft and revise the plain language summary of our findings (see appendix 2). This will be included in the final report to be published in Health Technology Assessment,^176^ as well as on our website. We have produced short video presentations of our work that are also available. Our findings will be publicised on social media and at our regular research engagement events in the local community. As this systematic review was secondary research using aggregated, anonymised data, we do not have any contact details for the participants in the primary research to enable us to share the findings directly with them.

Provenance and peer review: Not commissioned; externally peer reviewed.
