# Supplementary material for: Community based complex interventions to sustain independence in older people: systematic review and network meta-analysis
Source: BMJ. 2024 Mar 21;384:e077764. doi: 10.1136/bmj-2023-077764 (PMC10955723; doi:10.1136/bmj-2023-077764)
Supplement: Supplementary file 1 — Web appendix: Appendix 1-13 [file crot077764.ww1.pdf]

COMMUNITY BASED COMPLEX INTERVENTIONS TO SUSTAIN INDEPENDENCE IN OLDER PEOPLE: SYSTEMATIC REVIEW AND NETWORK META-ANALYSIS

Authors: Thomas F Crocker, Joie Ensor, Natalie Lam, Magda Jordão, Ram Bajpai, Matthew Bond, Anne Forster, Richard Riley, Deirdre Andre, Caroline Brundle, Alison Ellwood, John Green, Matthew Hale, Lubena Mirza, Jessica Morgan, Ismail Patel, Eleftheria Patetsini, Matthew Prescott, Ridha Ramiz, Oliver Todd, Rebecca Walford, John Gladman, Andrew Clegg

BMJ 2024;384:e077764. https://doi.org/10.1136/bmj-2023-077764

SUPPLEMENTARY MATERIAL

Study registration

PROSPERO: CRD42019162195. Available from:
https://www.crd.york.ac.uk/PROSPERO/display_record.asp?ID=CRD42019162195

Funding details

This project was funded by the National Institute for Health Research (NIHR) Health Technology Assessment programme (NIHR128862) and will be published in full in Health Technology Assessment journal.[1] Sections of this appendix are reproduced from the full report. See the NIHR Journals Library website for further project information. Available from: https://dev.fundingawards.nihr.ac.uk/award/NIHR128862

Additional data

Crocker TF, Lam N, Ensor J, Jordão M, Bajpai R, Bond M, et al. (2023) Community-based complex interventions to sustain independence in older people systematic review and network meta-analysis: effect estimates and findings dataset. University of Leeds. [Dataset] https://doi.org/10.5518/1377

Crocker TF, Lam N, Jordão M, Brundle C, Prescott M, Forster A, et al. (2023) Community-based complex interventions to sustain independence in older people systematic review and network meta-analysis: risk-of-bias dataset. University of Leeds. [Dataset] https://doi.org/10.5518/1386

This work is made available under a Creative Commons Attribution 4.0 (CC BY 4.0) license, which permits you to copy, redistribute, remix, transform and build upon this work for any purpose, provided you properly cite the original work, give a link to the licence, and indicate if changes were made. See: https://creativecommons.org/licenses/by/4.0/.

Contents

Appendix 1. Differences from the published protocol 8
1.1 Changes to protocol following publication 8
1.2 Differences from the planned protocol in analyses conducted 8
Appendix 2. Plain language summary 9
Appendix 3. Search strategies and their development 10
3.1 Search strategy development process 10
3.2 Electronic search strategies 10
3.2.1 CENTRAL 10
3.2.2 MEDLINE 12
3.2.3 Embase 13
3.2.4 APA Psycinfo 16
3.2.5 CINAHL 17
3.2.6 Trial registers 22
Appendix 4. Data items 23
Appendix 5. Interpretation of the evidence 24
Appendix 6. Excluded reports 25
Appendix 7. Included studies and reports 35
Appendix 8. Risk of bias in results of interest 39
8.1 Study-level risk of bias for individually-randomised studies 39
8.1.1 Risk of bias due to the randomisation process (individual) 39
8.1.2 Risk of bias due to deviations from the intended interventions 39
8.2 Study-level risk of bias for cluster-randomised studies 39
8.2.1 Risk of bias due to the randomisation process (cluster) 39
8.2.2 Risk of bias due to identification or recruitment of participants into clusters 39
8.2.3 Risk of bias due to deviations from the intended interventions 39
8.3 Risk of bias in results of interest 40
8.4 Overall risk of bias 40
Appendix 9. Description of the components and aspects of components used to determine intervention groups, organised by topic 41
Appendix 10. TIDieR descriptions of intervention groups with more than one intervention 44
Group: ADL 44
Group: ADL, aids, education, exercise, multifactorial-action and review with medication review and self-management 45
Group: Aids 46
Group: Available care 47
Group: Education 49
Group: Education, exercise, multifactorial-action and review with medication review and self-management strategies 50
Group: Education, multifactorial-action and review with medication review 52
Group: Education, multifactorial-action and review with medication review and self-management strategies 53
Group: Education, multifactorial-action and review with self-management strategies. 55
Group: Exercise 56
Group: Exercise and psychology 57
Group: Homecare 58
Group: Homecare and multifactorial-action 60
Group: Homecare, ADL, multifactorial-action from care-planning and review with self-management strategies 61
Group: Homecare, multifactorial-action and review 62
Group: Homecare, multifactorial-action from care-planning and review with medication review 64
Group: Homecare, multifactorial-action and review with self-management strategies 65
Group: Meaningful activities and education 66
Group: Multifactorial-action 67

Group: Multifactorial-action and review ... 69
Group: Multifactorial-action and review with medication review ... 71
Group: Multifactorial-action and review with medication review and self-management strategies ... 73
Group: Multifactorial-action and review with self-management strategies ... 74
Group: Multifactorial-action with medication review ... 75
Group: Nutrition and exercise ... 76
Group: Risk-screening ... 77
Appendix 11. Results of network meta-analyses ... 79
11.1 Summary of network meta-analyses ... 79
11.2 Living at home ... 80
11.2.1 Living at home available care network, short-term timeframe ... 80
11.2.2 Living at home available care network, medium-term timeframe ... 83
11.2.3 Living at home available care network, long-term timeframe ... 86
11.2.4 Living at home homecare network, short-term timeframe ... 91
11.2.5 Living at home homecare network, medium-term timeframe ... 94
11.2.6 Living at home homecare network, long-term timeframe ... 97
11.3 Instrumental ADL ... 97
11.3.1 IADL available care network, short-term timeframe ... 97
11.3.2 IADL available care network, medium-term timeframe ... 101
11.3.3 IADL available care network, long-term timeframe ... 108
11.3.4 IADL homecare network, short-term timeframe ... 113
11.3.5 IADL homecare network, medium-term timeframe ... 116
11.3.6 IADL homecare network, long-term timeframe ... 120
11.4 Personal ADL ... 121
11.4.1 PADL available care network, short-term timeframe ... 121
11.4.2 PADL available care network, medium-term timeframe ... 125
11.4.3 PADL available care network, long-term timeframe ... 132
11.4.4 PADL homecare network, short-term timeframe ... 136
11.4.5 PADL homecare network, medium-term timeframe ... 139
11.4.6 PADL homecare network, long-term timeframe ... 143
11.5 Hospitalisation ... 143
11.5.1 Hospitalisation available care network (medium-term timeframe only) ... 143
11.5.2 Hospitalisation homecare network (medium-term timeframe only) ... 148
11.6 Care-home placement ... 148
11.6.1 Care-home placement available care network, short-term timeframe ... 148
11.6.2 Care-home placement available care network, medium-term timeframe ... 152
11.6.3 Care-home placement available care network, long-term timeframe ... 158
11.6.4 Care-home placement homecare network, short-term timeframe ... 162
11.6.5 Care-home placement homecare network, medium-term timeframe ... 163
11.6.6 Care-home placement homecare network, long-term timeframe ... 166
11.7 Health status ... 166
11.7.1 Health status available care network (medium-term timeframe only) ... 166
11.7.2 Health status homecare network (medium-term timeframe only) ... 172
11.8 Depression ... 173
11.8.1 Depression available care network (medium-term timeframe only) ... 173
11.8.2 Depression homecare network (medium-term timeframe only) ... 181
11.9 Mortality (medium-term timeframe only) ... 186
Appendix 12. Health economic evaluations and results ... 196
Appendix 13. Narrative and additional outcomes ... 216
References ... 222

Figures

Figure 1 - Summary of study-level risk of bias for the domains related to allocation and deviations from the intended intervention in the 113 studies with results of interest. Four studies had results at differing risk of bias in domain 2 so we have used the highest risk in this figure 39
Figure 2 - Summary of result-level risk of bias for the domains related to missing outcome data, measurement of the outcome and selection of the reported result in the 826 results of interest suitable for analyses. 40
Figure 3 - Pairwise meta-analysis for living at home: medium-term available care network (pooling comparisons with greater than one study reporting results) 86
Figure 4 - Comparison-adjusted funnel plot for living at home: medium-term available care network 86
Figure 5 - Pairwise meta-analysis for living at home: long-term available care network (pooling comparisons with greater than one study reporting results) 90
Figure 6 - Pairwise meta-analysis for living at home: short-term homecare network (pooling comparisons with greater than one study reporting results) 93
Figure 7 - Example of disconnected network for IADL short-term timeframe, showing separation between studies with available care (ac) comparator and homecare (hmcr) comparator 100
Figure 8 - Pairwise meta-analysis for IADL: medium-term available care network (pooling comparisons with greater than one study reporting results) 105
Figure 9 - Comparison-adjusted funnel plot for IADL: medium-term available care network 106
Figure 10 - Example of disconnected network for IADL medium-term timeframe, showing separation between studies with available care (ac) comparator and homecare (hmcr) comparator 106
Figure 11 - Rankogram showing comparative effectiveness of interventions for IADL medium-term available care network. Results based on a simulation of 1000 replications. 107
Figure 12 - Example of disconnected network for risk of bias sensitivity analysis for IADL medium-term timeframe, showing separation between studies with available care (ac) comparator and homecare (hmcr) comparator 107
Figure 13 - Network diagram for risk of bias analysis for IADL medium-term timeframe with available care (ac) comparator .. 108
Figure 14 - Pairwise meta-analysis for IADL: long-term available care network (pooling comparisons with greater than one study reporting results) 111
Figure 15 - Example of disconnected network for IADL long-term timeframe, showing separation between studies with available care (ac) comparator and homecare (hmcr) comparator 112
Figure 16 - Rankogram showing comparative effectiveness of interventions for IADL long-term available care network. Results based on a simulation of 1000 replications. 112
Figure 17 - Pairwise meta-analysis for IADL: medium-term homecare network (pooling comparisons with greater than one study reporting results) 119
Figure 18 - Rankogram showing comparative effectiveness of interventions for IADL medium-term homecare network. Results based on a simulation of 1000 replications. 120
Figure 19 - Rankogram showing comparative effectiveness of interventions for PADL short-term available care network. Results based on a simulation of 1000 replications. 124
Figure 20 - Rankogram showing comparative effectiveness of interventions for PADL medium-term available care network. Results based on a simulation of 1000 replications. 129
Figure 21 - Pairwise meta-analysis for PADL: medium-term available care network (pooling comparisons with greater than one study reporting results) 130
Figure 22 - Comparison-adjusted funnel plot for PADL medium-term timeframe 130
Figure 23 - Network plot for risk of bias analysis for PADL medium-term timeframe with available care (ac) comparator 131
Figure 24 - Rankogram showing comparative effectiveness of interventions for risk of bias sensitivity analysis for PADL medium-term available care network. Results based on a simulation of 1000 replications. 131
Figure 25 - Pairwise meta-analysis for PADL: long-term available care network (pooling comparisons with greater than one study reporting results) 135
Figure 26 - Rankogram showing comparative effectiveness of interventions for PADL long-term available care network. Results based on a simulation of 1000 replications. 135
Figure 27 - Rankogram showing comparative effectiveness of interventions for PADL short-term homecare network. Results based on a simulation of 1000 replications. 138
Figure 28 - Rankogram showing comparative effectiveness of interventions for PADL medium-term homecare network. Results based on a simulation of 1000 replications. 141
Figure 29 - Network plot for risk of bias analysis for PADL medium-term homecare network 142

Figure 30 - Rankogram showing comparative effectiveness of interventions for risk of bias sensitivity analysis for PADL medium-term homecare network. Results based on a simulation of 1000 replications. 142
Figure 31 - Pairwise meta-analysis for hospitalisation: medium-term available care network (pooling comparisons with greater than one study reporting results) 147
Figure 32 - Comparison-adjusted funnel plot for hospitalisation: medium-term available care network 148
Figure 33 - Pairwise meta-analysis for care-home placement: medium-term available care network (pooling comparisons with greater than one study reporting results) 156
Figure 34 - Comparison-adjusted funnel plot for care-home placement: medium-term available care network 157
Figure 35 - Pairwise meta-analysis for care-home placement: long-term available care network (pooling comparisons with greater than one study reporting results) 162
Figure 36 - Pairwise meta-analysis for Health status: medium-term available care network (pooling comparisons with greater than one study reporting results) 169
Figure 37 - Example of disconnected network for health status medium-term available care network 170
Figure 38 - Rankogram showing comparative effectiveness of interventions for Health Status medium-term available care network. Results based on a simulation of 1000 replications 170
Figure 39 - Network plot for risk of bias analysis for Health Status medium-term available care network 171
Figure 40 - Rankogram showing comparative effectiveness of interventions for risk of bias sensitivity analysis for Health Status medium-term available care network. Results based on a simulation of 1000 replications 172
Figure 41 - Pairwise meta-analysis for depression: medium-term available care network (pooling comparisons with greater than one study reporting results) 177
Figure 42 - Rankogram showing comparative effectiveness of interventions for depression medium-term available care network. Results based on a simulation of 1000 replications. 178
Figure 43 - Comparison-adjusted funnel plot for depression: medium-term available care network 178
Figure 44 - Network plot for risk of bias analysis for depression medium-term available care network 179
Figure 45 - Rankogram showing comparative effectiveness of interventions for risk of bias sensitivity analysis for depression medium-term available care network. Results based on a simulation of 1000 replications. 180
Figure 46 - Rankogram showing comparative effectiveness of interventions for depression medium-term homecare network. Results based on a simulation of 1000 replications. 184
Figure 47 - Network plot for risk of bias analysis for depression medium-term homecare network 185
Figure 48 - Rankogram showing comparative effectiveness of interventions for risk of bias sensitivity analysis for depression medium-term homecare network. Results based on a simulation of 1000 replications. 185
Figure 49 - Pairwise meta-analysis for mortality: medium-term network (pooling comparisons with greater than one study reporting results 195

Tables

Table 1 - CINAHL search strategy 17
Table 2 - Table of excluded reports 25
Table 3 - Summary characteristics of 129 included studies .[503-631] 35
Table 4 - Summary of network meta-analyses 79
Table 5 - Short-term living at home available-care network 80
Table 6 - Living at home in the short term: comparisons with available care summary of findings table. 81
Table 7 - Results of living at home: short-term available care network 82
Table 8 - Intervention rankings for living at home: short-term available care network 83
Table 9 - Medium-term living at home available-care network 83
Table 10 - Results of living at home: medium-term available care network 84
Table 11 - Intervention rankings for living at home: medium-term available care network 85
Table 12 - Long-term living at home available-care network 86
Table 13 - Living at home in the long term: comparisons with available care summary of findings table 87
Table 14 - Results of living at home: long-term available care network 89
Table 15 - Intervention rankings for living at home: long-term available care network 90
Table 16 - Short-term living at home homecare network 91
Table 17 - Living at home in the short term: comparisons with homecare summary of findings table 92
Table 18 - Results of living at home: short-term homecare network 93
Table 19 - Intervention rankings for living at home: short-term homecare network 93

Table 20 - Medium-term living at home homecare network 94
Table 21 - Living at home in the medium term: comparisons with homecare summary of findings table 95
Table 22 - Results of living at home: medium-term homecare network 96
Table 23 - Intervention rankings for living at home: medium-term homecare network 97
Table 24 - Short-term IADL available-care network 97
Table 25 - IADL in the short term: comparisons with available care summary of findings table 98
Table 26 - Results of IADL: short-term available care network 99
Table 27 - Intervention rankings for IADL: short-term available care network 100
Table 28 - Medium-term IADL available-care network 101
Table 29 - IADL in the medium term: comparisons with available care summary of findings table 102
Table 30 - Results of IADL: medium-term available care network 104
Table 31 - Intervention rankings for IADL: medium-term available care network 105
Table 32 - Long-term IADL available-care network 108
Table 33 - IADL in the long term: comparisons with available care summary of findings table 109
Table 34 - Results of IADL: long-term available care network 111
Table 35 - Intervention rankings for IADL: long-term available care network 111
Table 36 - Short-term IADL homecare network 113
Table 37 - IADL in the short term: comparisons with homecare summary of findings table 114
Table 38 - Results of IADL: short-term homecare network 116
Table 39 - Intervention rankings for IADL: short-term homecare network 116
Table 40 - Medium-term IADL homecare network 116
Table 41 - IADL in the medium term: comparisons with homecare summary of findings table 117
Table 42 - Results of IADL: medium-term homecare network 119
Table 43 - Intervention rankings for IADL: medium-term homecare network 119
Table 44 - Short-term PADL available-care network 121
Table 45 - PADL in the short term: comparisons with available care summary of findings table 122
Table 46 - Results of PADL: short-term available care network 123
Table 47 - Intervention rankings for PADL: short-term available care network 124
Table 48 - Medium-term PADL available-care network 125
Table 49 - PADL in the medium term: comparisons with available care summary of findings table 126
Table 50 - Results of PADL: medium-term available care network 128
Table 51 - Intervention rankings for PADL: medium-term available care network 129
Table 52 - Long-term PADL available-care network 132
Table 53 - PADL in the long term: comparisons with available care summary of findings table 133
Table 54 - Results of PADL: long-term available care network 134
Table 55 - Intervention rankings for PADL: long-term available care network 135
Table 56 - Short-term PADL homecare network 136
Table 57 - PADL in the short term: comparisons with homecare summary of findings table 137
Table 58 - Results of PADL: short-term homecare network 138
Table 59 - Intervention rankings for PADL: short-term homecare network 138
Table 60 - Medium-term PADL homecare network 139
Table 61 - PADL in the medium term: comparisons with homecare summary of findings table 140
Table 62 - Results of PADL: medium-term homecare network 141
Table 63 - Intervention rankings for PADL: medium-term homecare network 141
Table 64 - Intervention rankings for risk of bias analysis for PADL: medium-term homecare network 142
Table 65 - Results of risk of bias PADL: medium-term homecare network 143
Table 66 - Medium-term hospitalisation available-care network 143
Table 67 - Hospitalisation in the medium term: comparisons with available care summary of findings table. 144
Table 68 - Results of hospitalisation medium-term analysis 146
Table 69 - Intervention rankings for hospitalisation medium-term analysis 147

Table 70 - Short-term care-home placement available-care network ... 148
Table 71 - Care-home placement in the short term: comparisons with available care summary of findings table ... 149
Table 72 - Results of care-home placement: short-term available care network ... 150
Table 73 - Intervention rankings for care-home placement: short-term available care network ... 151
Table 74 - Medium-term care-home placement available-care network ... 152
Table 75 Care-home placement in the medium term: comparisons with available care summary of findings table ... 153
Table 76 - Results of care-home placement: medium-term available care network ... 155
Table 77 - Intervention rankings for care-home placement: medium-term available care network ... 156
Table 78 - Long-term care-home placement available-care network ... 158
Table 79 - Care-home placement in the long term: comparisons with available care summary of findings table. ... 159
Table 80 - Results of care-home placement: long-term available care network ... 161
Table 81 - Intervention rankings for care-home placement: long-term available care network ... 162
Table 82 - Medium-term care-home placement homecare network ... 163
Table 83. Care-home placement in the medium term: comparisons with homecare summary of findings table. ... 164
Table 84 - Results of care-home placement: medium-term homecare network ... 166
Table 85 - Intervention rankings for care-home placement: medium-term homecare network ... 166
Table 86 - Medium-term health status available-care network ... 166
Table 87 - Self-reported health in the medium term: comparisons with available care summary of findings table. ... 167
Table 88 - Results of Health status: medium-term available care network ... 168
Table 89 - Intervention rankings for Health status: medium-term available care network ... 169
Table 90 - Results of Risk of Bias analysis for Health Status: medium-term available care network ... 172
Table 91 - Medium-term depression available-care network ... 173
Table 92 - Depression in the medium term: comparisons with available care summary of findings table ... 174
Table 93 - Results of Depression: medium-term available care network ... 176
Table 94 - Intervention rankings for Depression: medium-term available care network ... 177
Table 95 - Intervention rankings for risk of bias analysis for depression: medium-term available care network ... 179
Table 96 - Results of risk of bias depression: medium-term available care network ... 181
Table 97 - Medium-term depression homecare network ... 181
Table 98 - Depression in the medium term: comparisons with homecare summary of findings table ... 182
Table 99 - Results of Depression: medium-term homecare network ... 183
Table 100 - Intervention rankings for Depression: medium-term homecare network ... 184
Table 101 - Intervention rankings for risk of bias analysis for depression: medium-term homecare network ... 185
Table 102 - Results of risk of bias depression: medium-term homecare network ... 186
Table 103 - Medium-term mortality ... 186
Table 104 - Mortality in the medium term: comparisons with available care summary of findings table ... 188
Table 105 - Results of mortality: medium-term network ... 192
Table 106 - Intervention rankings for mortality: medium-term network ... 194
Table 107 - Economic evaluation analysis and principal findings reported for the short-term time horizon ... 196
Table 108 - Economic evaluation analysis and principal findings reported for the medium-term time horizon ... 201
Table 109 - Economic evaluation analysis and principal findings reported for the long-term time horizon ... 209
Table 110 - Homecare usage results ... 217
Table 111 - Loneliness results ... 218
Table 112 - Falls results ... 219

Appendix 1. Differences from the published protocol

Our protocol was published in BMJ Open prior to analyses beginning.[2]

1.1 Changes to protocol following publication

We precisely specified the cutoffs for our timeframes, which were already specified as around 6, 12, 24 months. We selected the medium-term (around 12 months) timeframe as the main timeframe for summary reports.

We initially planned to conduct meta-analyses of both odds ratios (ORs) and risk ratios (RRs) for dichotomous outcomes where possible but decided not to calculate RRs in meta-analysis due to their limited portability.[3] We instead calculated an RR from the pooled OR to assist with interpretation.[4]

We initially planned to use GRADE[5,6] to assess certainty but added CINeMA[7,8] with the intention of making our assessments more systematic and reproducible. However, once we had performed our analyses, we discovered that the CINeMA software could not estimate imprecision, heterogeneity or incoherence for many of our networks. We therefore returned to our original plan of using GRADE.

We added methods based on the Cochrane Handbook to summarise economic evidence. Although costs and cost-effectiveness were specified as outcomes of interest, no methods were initially specified for handling economic evidence. It is rarely appropriate to conduct meta-analysis of cost and cost-effectiveness outcomes.[9]

1.2 Differences from the planned protocol in analyses conducted

95% CIs were calculated using the Wald based approach, but with inflated variances to account for uncertainty in the estimated variances, similar to the approach of Hartung-Knapp but using a normal approximation instead of a t-distribution.[10,11]

The software programs we used included EndNote and Covidence, in addition to Rayyan, for citation record management/study selection. We used Microsoft Access, Excel, and Word to compile the tables in the reports instead of GRADEPro and RevMan.

Due to the availability of data and sparsity of our networks we were unable to conduct all of our analyses as planned. This led to the following differences:

- We limited the analyses for some outcomes and additional analyses to the main timeframe (medium term) due to the escalating number of analyses caused by having a split network for almost all outcomes (available-care and homecare networks).
- Frailty analyses, where conducted, were based on study-level categorisation of frailty only as too few studies provided validated measures to conduct our planned initial frailty analyses.
- We conducted our risk of bias sensitivity analyses by excluding results rated very serious concerns about risk of bias as there were no low-risk results (our planned approach).
- We did not conduct planned sensitivity analyses for publication years or follow-up timeframes as there were too few trials to include.

Appendix 2. Plain language summary

Which community services are best for helping older people to be independent?

Key messages

- Individualised care planning, where medication is adjusted and there are regular follow-ups, probably helps people stay living at home.
- Due to a lack of robust evidence, the benefits and risks of most types of community services for older people are unclear.

What are community services for older people?

There are many kinds of community services for older people. For example, in some services, everyone is given exercise and dietary advice or an individualised care plan. These often aim to help older people age independently.

What was the study about?

Maintaining independence is important in later life.

We wanted to find out which community services work best:

- to help people stay living at home, and
- to do day-to-day activities independently.

We reviewed findings from previous studies that have tested different community services for older people. We combined these findings and compared different types of service with one another. We rated our confidence in the evidence.

What did we find?

We found 129 studies with 74,946 people. We found 63 different kinds of service have been studied. The studies were carried out in diverse populations around the world.

Individualised care planning, where medication is adjusted and there are regular follow-ups may help people age independently. It probably increases the chance of staying at home slightly. It may also help with doing day-to-day activities very slightly.

Exercise and dietary advice may also help people stay living at home.

However, there was some evidence that some services may reduce independence.

We do not know what effect most services have.

What are the limitations of the evidence?

We generally had little confidence in the evidence because studies were small, and information was missing.

How current is the evidence?

The evidence is up to date to August 2021.

Appendix 3. Search strategies and their development

The search contained the following concepts:
1. Older people or frailty
2. Home-based or community interventions
3. RCT filter
4. 1 AND 2 AND 3

Restrictions by publication status or language were not used.

3.1 Search strategy development process

Search terms were harvested by exploring three relevant systematic reviews and their included studies.[12-14] Their search strategies, as well as words and phrases in title, abstract and subject indexing were reviewed to find relevant search terms for inclusion. These terms were used to develop the initial draft search strategy. Extra search terms were found by reviewing results from that initial strategy. The PubMed PubReMiner (https://hgserver2.amc.nl/cgi-bin/miner/miner2.cgi) word frequency analysis tool was also used to find index terms and keyword terms for inclusion in the search strategy. For the concept 'home-based or community interventions', we included both broad and specific search terms as testing showed that this was necessary to capture all relevant interventions. We limited terms about geriatric nursing to community or home settings to increase the relevance of search results. Following testing, we also excluded some specific medical conditions in titles to increase the relevancy of the search results. For our MEDLINE search we added the Cochrane Collaboration highly sensitive filter, to identify randomised trials.[15] This was supplemented with a search filter developed to find Phase Three Trials not found by the Cochrane RCT filter.[16] For the Embase search we used the filter for finding randomised trials in Embase developed by the Cochrane Collaboration.[17] For the Psycinfo search we used a sensitive methodological search filter.[18] For the CINAHL search we developed our own search strategy to identify randomised trials. The strategies were peer reviewed by another information specialist prior to execution using the Peer Review of Electronic Search Strategies Checklist.[19]

3.2 Electronic search strategies

3.2.1 CENTRAL

Cochrane Central Register of Controlled Trials (CENTRAL) via Wiley interface was searched. The database coverage was 1992 to present and the database was searched on the 11th of August 2021

#1 ((frail* or prefrailty)):ti,ab,kw (Word variations have been searched) 4037

#2 MeSH descriptor: [Aged] explode all trees 213642

#3 MeSH descriptor: [Geriatrics] this term only 207

#4 (elderly or old* next people* or old* next person* or old* next wom?n* or old* next m?n* or old* next male* or old* next female* or old* next adult* or old* next age* or aging or geriatric* or senior next citizen* or seniors or pensioner* or veteran* or sexagenarian* or septuagenarian* or octogenarian* or nonagenarian* or centenarian*):ti,ab,kw 92534

#5 ((over Near/2 ("60" or "61" or "62" or "63" or "64" or "65" or "66" or "67" or "68" or "69" or "70" or "71" or "72" or "73" or "74" or "75" or "76" or "77" or "78" or "79" or "80" or "81" or "82" or "83" or "84" or "85" or "86" or "87" or "88" or "89" or "90" or "91" or "92" or "93" or "94" or "95" or "96" or "97" or "98" or "99" or "100") Near years)):ti,ab,kw (Word variations have been searched) 3277

#6 {or #1-#5} 283983

#7 MeSH descriptor: [Independent Living] this term only 544

#8 MeSH descriptor: [Community Health Nursing] explode all trees 345

#9 ("Community support services"):ti,ab,kw (Word variations have been searched) 23

#10 MeSH descriptor: [Managed Care Programs] explode all trees 502

#11 ("health maintenance organization*" or "health maintenance organisation*"):ti,ab,kw (Word variations have been searched) 627

#12 (HMO*):ti,ab,kw (Word variations have been searched) 494

#13 MeSH descriptor: [Social Work] this term only 184

#14 (social Near/3 services):ti,ab,kw (Word variations have been searched) 1417

#15 ("Voluntary services"):ti,ab,kw (Word variations have been searched) 14

#16 MeSH descriptor: [Home Nursing] this term only 282

#17 ("house call*"):ti,ab,kw (Word variations have been searched) 583

#18 (home near/5 visit*):ti,ab,kw (Word variations have been searched) 5140

#19 ((("general practice" or "primary care" or nurse* or group or "ambulatory clinic" or "geriatric clinic") near/3 visit*)):ti,ab,kw (Word variations have been searched) 4731

#20 MeSH descriptor: [Geriatric Assessment] this term only 1509

#21 (pharmac* near/2 visit):ti,ab,kw (Word variations have been searched) 278

#22 ((home or house) near/2 appointment*):ti,ab,kw (Word variations have been searched) 24

#23 ("Home Care Services"):ti,ab,kw (Word variations have been searched) 2257

#24 MeSH descriptor: [Home Care Services] this term only 1883

#25 MeSH descriptor: [Health Services for the Aged] this term only 456

#26 MeSH descriptor: [Home Health Nursing] explode all trees 7

#27 ("district nursing"):ti,ab,kw (Word variations have been searched) 115

#28 ("health visit*"):ti,ab,kw (Word variations have been searched) 186

#29 ("community matron"):ti,ab,kw (Word variations have been searched) 4

#30 (home Near/3 (intervention* or support* or assessment*)):ti,ab,kw (Word variations have been searched) 4926

#31 MeSH descriptor: [Home Health Nursing] this term only 7

#32 (((preventive* or preventative*) near/5 medicine)):ti,ab,kw (Word variations have been searched) 781

#33 MeSH descriptor: [Preventive Medicine] this term only 121

#34 ((preventive* or preventative*) near/3 (program* or intervent* or support* or care or service* or approach* or "case management" or measure* or OT or "occupational therapy" or assess*)):ti,ab,kw (Word variations have been searched) 6275

#35 {or #7-#34} 25735

#36 MeSH descriptor: [Geriatric Nursing] this term only 178

#37 ("geriatric nursing"):ti,ab,kw (Word variations have been searched) 274

#38 {or #36-#37} 274

#39 MeSH descriptor: [Community Health Services] this term only1061

#40 (community):ti,ab,kw 46478

#41 MeSH descriptor: [Community Health Nursing] explode all trees 345

#42 MeSH descriptor: [Community Pharmacy Services] this term only 271

#43 MeSH descriptor: [Home Care Services] this term only 1883

#44 MeSH descriptor: [Aftercare] this term only 661

#45 MeSH descriptor: [Primary Health Care] this term only 4388

#46 (domiciliary or ("social support" and home*) or ((homecare or medical) near/2 home) or (home and package*) or (outreach and home) or "(alternative setting" and home) or "home visit*" or "home manag*" or homecare or "home care" or "home therap*" or (model* adj1 home*) or "home program*" or "home monitor*"):ti,ab,kw (Word variations have been searched) 12652

#47 ("home-based" or homebased or homebound):ti,ab,kw (Word variations have been searched) 7510

#48 ((live or living or lived or dwell*) near/5 ("at home" or "own home" or "in home" or alone or independent*)):ti,ab,kw (Word variations have been searched) 3855

#49 ("Home care" or "primary care" or "primary healthcare" or "primary health care" or "community dwelling"):ti,ab,kw (Word variations have been searched) 31085

#50 {or #39-#49} 80654

#51 #38 AND #50 103

#52 #35 or #51 25779

#53 #6 and #52 8010

#54 (coronary heart disease or CHD or chronic obstructive pulmonary disease or COPD or kidney failure or CKD or Heart failure or diabetes or asthma or cancer or schizophrenia or severe mental illness*):ti 210929

#55 #53 NOT #54 7003

3.2.2 MEDLINE

MEDLINE(R) ALL was searched via OvidSP. The database coverage was 1946 to present and the database was searched on 9th of August 2021

1 randomized controlled trial.pt. (539556)

2 controlled clinical trial.pt. (94320)

3 randomized.ab. (529280)

4 placebo.ab. (220248)

5 clinical trials as topic.sh. (196870)

6 randomly.ab. (363058)

7 trial.ti. (244962)

8 or/1-7 (1384889)

9 exp animals/ not humans.sh. (4870600)

10 8 not 9 [Cochrane Highly Sensitive Search Strategy for identifying randomized trials in MEDLINE: sensitivity- and precision-maximizing version (2008 revision] (1274483)

11 Clinical Trial, Phase III/ (18797)

12 ("phase 3" or "phase3" or "phase III" or "P3" or "PIII").ti,ab,kw. (73139)

13 11 or 12 [search filter for phase three trials to supplement Cochrane HSSS, Cooper 2019] (79735)

14 10 or 13 [final RCT filter] (1318490)

15 (frail* or prefrailty).tw. (25865)

16 exp aged/ (3283911)

17 geriatrics/ (30590)

18 (elder* or older or old people* or old person* or old wom#n*1 or old m#n*1 or old male*1 or old female*1 or old adult*1 or old age* or aging or ageing or geriatric* or senior citizen* or seniors or pensioner* or veteran* or sexagenarian* or septuagenarian* or octogenarian* or nonagenarian* or centenarian*).tw,kf. (1385083)

19 (over adj2 ("60" or "61" or "62" or "63" or "64" or "65" or "66" or "67" or "68" or "69" or "70" or "71" or "72" or "73" or "74" or "75" or "76" or "77" or "78" or "79" or "80" or "81" or "82" or "83" or "84" or "85" or "86" or "87" or "88" or "89" or "90" or "91" or "92" or "93" or "94" or "95" or "96" or "97" or "98" or "99" or "100") adj years).tw. (21451)

20 or/15-19 [older or frail people] (4132930)

21 independent living/ (8001)

22 community health services/ (32391)

23 community health nursing/ (19684)

24 Community support services.tw. (173)

25 exp managed care programs/ (40081)

26 (health maintenance organi?ation* or HMO*).tw. (13817)

27 (Social adj3 services).tw. (10694)

28 Voluntary services.tw. (99)

29 *home nursing/ (5361)

30 House Calls/ (3846)

31 house call*.tw. (656)

32 (home adj5 visit*).tw. (12399)

33 ((general practice or primary care or nurse* or group or ambulatory clinic or geriatric clinic) adj3 visit*).tw. (9527)

34 *geriatric assessment/ (13906)

35 (pharmac* adj2 visit).tw. (212)

36 ((home or house) adj2 appointment*).tw. (52)

37 Home Care Services/ (34738)

38 Home care service*.tw. (1913)

39 *health services for the aged/ (14001)

40 home health nursing/ (364)

41 district nursing.tw. (667)

42 health visit*.ti. or health visit*.ab. /freq=2 (2285)

43 community matron*.ti. or community matron*.ab. /freq=2 (83)

44 (home adj3 (intervention* or support* or assessment*)).tw. (8887)

45 preventive health services/ (14024)

46 ((preventive* or preventative*) adj5 medicine).tw. (7306)

47 preventative medicine/ (11938)

48 ((preventive* or preventative*) adj3 (program* or intervent* or support* or care or service* or approach* or case management or measure* or OT or occupational therapy or assess*)).tw. (66507)

49 or/21-48 (283985)

50 geriatric nursing/ (13707)

51 geriatric nurs*.tw,kf. (1164)

52 or/50-51 [geriatric nursing] (14118)

53 community.ti,ab,kf. (539116)

54 community health services/ or community health nursing/ or community mental health services/ or community pharmacy services/ (74241)

55 "domiciliary care"/ (34738)

56 aftercare/ (10404)

57 primary health care/ (83064)

58 (domiciliary or (social support and home*) or ((homecare or medical) adj2 home) or (home and package*) or (outreach and home) or (alternative setting and home) or home visit* or home manag* or homecare or home care or home therap* or (model* adj1 home*) or home program* or home monitor*).tw. (58982)

59 ((live or living or lived or dwell*) adj5 ("at home" or "own home" or "in home" or alone or independent*)).tw. (17479)

60 (home-based or homebased or homebound).tw. (12811)

61 (Home care or primary care or primary health care or primary healthcare).tw. (163820)

62 or/53-61 [interventions in a community or home setting] (808717)

63 52 and 62 [geriatric nursing and interventions in a community or home setting] (2015)

64 49 or 63 [all interventions] (284694)

65 (coronary heart disease or CHD or chronic obstructive pulmonary disease or COPD or kidney failure or CKD or Heart failure or diabetes or asthma or cancer or schizophrenia or severe mental illness*).ti. (1592860)

66 64 not 65 [all intervenions excluding specific diseases in title] (267883)

67 14 and 20 and 66 [RCTS and older people and interventions] (7005)

3.2.3 Embase

Embase and Embase Classic via OvidSP was searched. The database coverage was 1947 to present and the database was search on the 9th of August 2021

1 randomized controlled trial/ (672319)

2 controlled clinical study/ (463974)

3 1 or 2 (860531)

4 random*.tw. (1703521)

5 randomization/ (91766)

6 intermethod comparison/ (273924)

7 placebo.tw. (332206)

8 (compare or compared or comparison).ti. (574408)

9 ((evaluated or evaluate or evaluating or assessed or assess) and (compare or compared)).ab. (2067060)

10 (open adj label).ti,ab. (89661)

11 ((double or single or doubly or singly) adj blind).tw. (227285)

12 parallel group$1.tw. (27916)

13 double blind procedure/ (188870)

14 (crossover or cross over).tw. (113362)

15 ((assign* or match or matched or allocation) adj5 (alternate or group$1 or intervention$1 or patient$1 or subject$1 or participant$1)).tw. (362240)

16 (assigned or allocated).tw. (427304)

17 (controlled adj7 (study or design or trial)).tw. (388612)

18 (volunteer or volunteers).tw. (265628)

19 human experiment/ (551078)

20 trial.ti. (343846)

21 or/4-20 (5189451)

22 21 or 3 (5347546)

23 (random* adj sampl* adj7 ("cross section*" or questionnaire$1 or survey* or database$1)).tw. not (comparative study/ or controlled study/ or randomi?ed controlled.tw. or randomly assigned.tw.) (8774)

24 Cross-sectional study/ not (randomized controlled trial/ or controlled clinical study/ or controlled study/ or randomi?ed controlled.tw. or control group$1.tw.) (277846)

25 (((case adj control*) and random*) not randomi?ed controlled).tw. (18755)

26 (Systematic review not (trial or study)).ti. (182362)

27 (nonrandom* not random*).tw. (17268)

28 "Random field*".tw. (2544)

29 (random cluster adj3 sampl*).tw. (1374)

30 (review.ab. and review.pt.) not trial.ti. (913087)

31 "we searched".ab. and (review.ti. or review.pt.) (37761)

32 "update review".ab. (119)

33 (databases adj4 searched).ab. (44421)

34 (rat or rats or mouse or mice or swine or porcine or murine or sheep or lambs or pigs or piglets or rabbit or rabbits or cat or cats or dog or dogs or cattle or bovine or monkey or monkeys or trout or marmoset$1).ti. and animal experiment/ (1116446)

35 Animal experiment/ not (human experiment/ or human/) (2346095)

36 or/23-35 (3759577)

37 22 not 36 [Cochrane Highly Sensitive Search Strategy for identifying controlled trials in Embase: (2018 revision); Ovid format (Glanville *et al.*, 2019b)] (4755948)

38 (frail* or prefrailty).tw. (39809)

39 aged/ (3370037)

40 very elderly/ (236950)

41 frail elderly/ (10922)

42 geriatrics/ (39915)

43 (elder* or older or old pele*ople* or old person* or old wom#n*1 or old m#n*1 or old ma1 or old female*1 or old adult*1 or old age* or aging or ageing or geriatric* or senior citizen* or seniors or pensioner* or veteran* or sexagenarian* or septuagenarian* or octogenarian* or nonagenarian* or centenarian*).tw,kw. (1838159)

44 (over adj2 ("60" or "61" or "62" or "63" or "64" or "65" or "66" or "67" or "68" or "69" or "70" or "71" or "72" or "73" or "74" or "75" or "76" or "77" or "78" or "79" or "80" or "81" or "82" or "83" or "84" or "85" or "86" or "87" or "88" or "89" or "90" or "91" or "92" or "93" or "94" or "95" or "96" or "97" or "98" or "99" or "100") adj years).tw. (33789)

45 or/38-44 [frail or elderly people] (4516350)

46 independent living/ (5523)

47	community care/ (61677)
48	community health nursing/ (26723)
49	Community support services.tw. (239)
50	(health maintenance organi?ation* or HMO*).tw. (16728)
51	(Social adj3 services).tw. (14016)
52	Voluntary services.tw. (148)
53	home visit/ (3712)
54	house call*.tw. (852)
55	(home adj5 visit*).tw. (17275)
56	((general practice or primary care or nurse* or group or ambulatory clinic or geriatric clinic) adj3 visit*).tw. (14158)
57	*geriatric assessment/ (6239)
58	(pharmac* adj2 visit).tw. (504)
59	((home or house) adj2 appointment*).tw. (107)
60	Home Care/ (66345)
61	Home care service*.tw. (2345)
62	*elderly care/ (21267)
63	district nursing.tw. (664)
64	health visit*.ti. or health visit*.ab. /freq=2 (2402)
65	community matron*.ti. or community matron*.ab. /freq=2 (82)
66	(home adj3 (intervention* or support* or assessment*)).tw. (12351)
67	preventive health service/ (30244)
68	((preventive* or preventative*) adj5 medicine).tw. (12282)
69	preventive medicine/ (29022)
70	((preventive* or preventative*) adj3 (program* or intervent* or support* or care or service* or approach* or case management or measure* or OT or occupational therapy or assess*)).tw. (88643)
71	or/46-70 [specific interventions] (376111)
72	geriatric nursing/ (12986)
73	geriatric nurs*.tw,kw. (1405)
74	or/72-73 [geriatric nursing] (13603)
75	community.tw,kw. (686753)
76	community health services/ or community health nursing/ or mental health service/ or "pharmacy (shop)"/ (144914)
77	aftercare/ (8598)
78	primary health care/ (70765)
79	(domiciliary or (social support and home*) or ((homecare or medical) adj2 home) or (home and package*) or (outreach and home) or (alternative setting and home) or home visit* or home manag* or homecare or home care or home therap* or (model* adj1 home*) or home program* or home monitor*).tw. (78824)
80	((live or living or lived or dwell*) adj5 ("at home" or "own home" or "in home" or alone or independent*)).tw. (24399)
81	(home-based or homebased or homebound).tw. (17773)
82	(Home care or primary care or primary healthcare or primary health care).tw. (217034)
83	or/75-82 [home or community setting] (1060004)
84	74 and 83 [geriatric nursing and home or community setting] (1864)
85	71 or 84 [all interventions] (376832)
86	(coronary heart disease or CHD or chronic obstructive pulmonary disease or COPD or kidney failure or CKD or Heart failure or diabetes or asthma or cancer or schizophrenia or severe mental illness*).ti. (2270105)
87	85 not 86 [all interventions except those mentioning specific diseases] (350036)

88 37 and 45 and 87 [RCT and elderly and Interventions] (17333)

3.2.4 APA Psycinfo

APA Psycinfo via OvidSP was searched. The database coverage was 1806 to present and the database was searched on the 9th of August 2021

1 (control: or random:).tw. or exp treatment/ [sensitive rct psycinfo search strategy Eady *et al.*, 2009] (1743140)

2 (frail* or prefrailty).tw. (5244)

3 exp aging/ (79898)

4 geriatric patients/ (13753)

5 geriatrics/ (11969)

6 (elder* or older or old people* or old person* or old wom#n*1 or old m#n*1 or old male*1 or old female*1 or old adult*1 or old age* or aging or geriatric* or senior citizen* or seniors or pensioner* or veteran* or sexagenarian* or septuagenarian* or octogenarian* or nonagenarian* or centenarian*).tw. (355903)

7 (over adj2 ("60" or "61" or "62" or "63" or "64" or "65" or "66" or "67" or "68" or "69" or "70" or "71" or "72" or "73" or "74" or "75" or "76" or "77" or "78" or "79" or "80" or "81" or "82" or "83" or "84" or "85" or "86" or "87" or "88" or "89" or "90" or "91" or "92" or "93" or "94" or "95" or "96" or "97" or "98" or "99" or "100") adj years).tw. (2391)

8 or/2-7 [frail or elderly people] (371975)

9 Self-Care Skills/ (4756)

10 community health/ (3653)

11 community services/ (17234)

12 social services/ (9557)

13 Community support services.tw. (219)

14 exp managed care/ (4567)

15 (health maintenance organi?ation* or HMO*).tw. (2449)

16 (Social adj3 services).tw. (11772)

17 Voluntary services.tw. (71)

18 home visiting programs/ (1861)

19 home care/ (6905)

20 house call*.tw. (106)

21 (home adj5 visit*).tw. (5619)

22 ((general practice or primary care or nurse* or group or ambulatory clinic or geriatric clinic) adj3 visit*).tw. (2716)

23 (pharmac* adj2 visit).tw. (23)

24 ((home or house) adj2 appointment*).tw. (12)

25 Independent Living Programs/ (408)

26 Home care service*.tw. (706)

27 district nursing.tw. (64)

28 health visit*.ti. or health visit*.ab. /freq=2 (342)

29 community matron*.ti. or community matron*.ab. /freq=2 (14)

30 (home adj3 (intervention* or support* or assessment*)).tw. (5172)

31 ((preventive* or preventative*) adj5 medicine).tw. (1085)

32 preventive medicine/ (2464)

33 ((preventive* or preventative*) adj3 (program* or intervent* or support* or care or service* or approach* or case management or measure* or OT or occupational therapy or assess*)).tw. (17107)

34 or/9-33 [interventions] (83270)

35 geriatric nursing.tw. (252)

36 (geriatrics/ or geriatric patients/) and nursing/ (639)

37 or/35-36 [geriatric nursing] (833)

38 community.tw. (275605)

39 community services/ or community health/ or community mental health services/ or pharmacy/ (28713)

40 (community healthcare or community health care).tw. (588)

41 home care/ (6905)

42 aftercare/ (1121)

43 primary health care/ (19284)

44 Public Health Service Nurses/ (658)

45 (domiciliary or (social support and home*) or ((homecare or medical) adj2 home) or (home and package*) or (outreach and home) or (alternative setting and home) or home visit* or home manag* or homecare or home care or home therap* or (model* adj1 home*) or home program* or home monitor*).tw. (19096)

46 ((live or living or lived or dwell*) adj5 ("at home" or "own home" or "in home" or alone or independent*)).tw. (10597)

47 (home-based or homebased or homebound).tw. (5823)

48 (Home care or primary care or primary health care or primary healthcare).tw. (44178)

49 or/38-48 [community or home based] (340141)

50 37 and 49 [geriatric nursing and community or home based] (174)

51 34 or 50 [all interventions] (83378)

52 (coronary heart disease or CHD or chronic obstructive pulmonary disease or COPD or kidney failure or CKD or Heart failure or diabetes or asthma or cancer or schizophrenia or severe mental illness*).ti. (116600)

53 51 not 52 [all interventions except specific diseases in title] (79888)

54 1 and 8 and 53 [RCT filter and elderly and all interventions except specific diseases in title] (7917)

3.2.5 CINAHL

CINAHL via EBSCOhost interface was searched. The database coverage was 1972 to present and the database was searched on the 9th of August 2021

Table 1 - CINAHL search strategy

#	Query	Limiters/Expanders	Last Run Via	Results
S46	S10 AND S18 and S45	Search modes - Boolean/Phrase	Interface - EBSCOhost Research Databases Search Screen - Advanced Search Database - CINAHL	10,636
S45	S43 NOT S44	Search modes - Boolean/Phrase	Interface - EBSCOhost Research Databases Search Screen - Advanced Search Database - CINAHL	106,016
S44	TI ("coronary heart disease" or CHD or "chronic obstructive pulmonary disease" or COPD or "kidney failure" or CKD or "Heart failure" or diabetes or asthma or cancer or schizophrenia or "severe mental illness*")	Search modes - Boolean/Phrase	Interface - EBSCOhost Research Databases Search Screen - Advanced Search Database - CINAHL	501,973
S43	S29 or S42	Search modes - Boolean/Phrase	Interface - EBSCOhost Research Databases Search Screen - Advanced Search Database - CINAHL	113,278

S42	S30 and S41	Search modes - Boolean/Phrase	Interface - EBSCOhost Research Databases Search Screen - Advanced Search Database - CINAHL	2,217
S41	S31 OR S32 OR S33 OR S34 OR S35 OR S36 OR S37 OR S38 OR S39 OR S40	Search modes - Boolean/Phrase	Interface - EBSCOhost Research Databases Search Screen - Advanced Search Database - CINAHL	687,262
S40	TX "Home care" or "primary care" or "primary health care" or "primary healthcare"	Search modes - Boolean/Phrase	Interface - EBSCOhost Research Databases Search Screen - Advanced Search Database - CINAHL	198,977
S39	TX "home-based" or homebased or homebound	Search modes - Boolean/Phrase	Interface - EBSCOhost Research Databases Search Screen - Advanced Search Database - CINAHL	9,035
S38	(MH "Community Health Services")	Search modes - Boolean/Phrase	Interface - EBSCOhost Research Databases Search Screen - Advanced Search Database - CINAHL	22,541
S37	TX ((live or living or lived or dwell*) N5 ("at home" or "own home" or "in home" or community or alone or independent*)	Search modes - Boolean/Phrase	Interface - EBSCOhost Research Databases Search Screen - Advanced Search Database - CINAHL	57,710
S36	TX domiciliary or ("social support" and home*) or ((homecare or medical) N2 home) or (home and package*) or (outreach and home) or (alternative setting and home) or home visit* or home manag* or homecare or "home care" or "home therap*" or (model* N1 home*) or "home program*" or "home monitor*")	Search modes - Boolean/Phrase	Interface - EBSCOhost Research Databases Search Screen - Advanced Search Database - CINAHL	68,720
S35	(MH "Primary Health Care")	Search modes - Boolean/Phrase	Interface - EBSCOhost Research Databases Search Screen - Advanced Search Database - CINAHL	67,490
S34	(MH "After Care")	Search modes - Boolean/Phrase	Interface - EBSCOhost Research Databases Search Screen - Advanced Search Database - CINAHL	16,366
S33	(MH "Community Health Nursing")	Search modes - Boolean/Phrase	Interface - EBSCOhost Research Databases Search Screen - Advanced Search Database - CINAHL	28,024

S32	(MH "Community Mental Health Services")	Search modes - Boolean/Phrase	Interface - EBSCOhost Research Databases<br>Search Screen - Advanced Search<br>Database - CINAHL	9,964
S31	TX community	Search modes - Boolean/Phrase	Interface - EBSCOhost Research Databases<br>Search Screen - Advanced Search<br>Database - CINAHL	479,269
S30	(MH "Gerontologic Nursing")	Search modes - Boolean/Phrase	Interface - EBSCOhost Research Databases<br>Search Screen - Advanced Search<br>Database - CINAHL	13,362
S29	S19 OR S20 OR S21 OR S22 OR S23 OR S24 OR S25 OR S26 OR S27 OR S28	Search modes - Boolean/Phrase	Interface - EBSCOhost Research Databases<br>Search Screen - Advanced Search<br>Database - CINAHL	111,533
S28	TX ((preventive* or preventative*) N3 (program* or intervent* or support* or care or service* or approach* or case management or measure* or OT or "occupational therapy" or assess*))	Search modes - Boolean/Phrase	Interface - EBSCOhost Research Databases<br>Search Screen - Advanced Search<br>Database - CINAHL	47,492
S27	(MH "Preventive Health Care")	Search modes - Boolean/Phrase	Interface - EBSCOhost Research Databases<br>Search Screen - Advanced Search<br>Database - CINAHL	21,369
S26	TX (home N3 (intervention* or support* or assessment*))	Search modes - Boolean/Phrase	Interface - EBSCOhost Research Databases<br>Search Screen - Advanced Search<br>Database - CINAHL	10,880
S25	TX "community matron*"	Search modes - Boolean/Phrase	Interface - EBSCOhost Research Databases<br>Search Screen - Advanced Search<br>Database - CINAHL	283
S24	TX "health visit*"	Search modes - Boolean/Phrase	Interface - EBSCOhost Research Databases<br>Search Screen - Advanced Search<br>Database - CINAHL	8,986
S23	TX "district nursing"	Search modes - Boolean/Phrase	Interface - EBSCOhost Research Databases<br>Search Screen - Advanced Search<br>Database - CINAHL	2,176
S22	MM "Home Health Care"	Search modes - Boolean/Phrase	Interface - EBSCOhost Research Databases<br>Search Screen - Advanced Search<br>Database - CINAHL	17,073

S21	(MH "Health Services for the Aged")	Search modes - Boolean/Phrase	Interface - EBSCOhost Research Databases<br>Search Screen - Advanced Search<br>Database - CINAHL	6,819
S20	(MH "Home Visits") or (MH "Community Living")	Search modes - Boolean/Phrase	Interface - EBSCOhost Research Databases<br>Search Screen - Advanced Search<br>Database - CINAHL	23,215
S19	TX "Community support services"	Search modes - Boolean/Phrase	Interface - EBSCOhost Research Databases<br>Search Screen - Advanced Search<br>Database - CINAHL	155
S18	S11 OR S12 OR S13 OR S14 OR S15 OR S16 OR S17	Search modes - Boolean/Phrase	Interface - EBSCOhost Research Databases<br>Search Screen - Advanced Search<br>Database - CINAHL	1,209,432
S17	TX (over N2 ("60" or "61" or "62" or "63" or "64" or "65" or "66" or "67" or "68" or "69" or "70" or "71" or "72" or "73" or "74" or "75" or "76" or "77" or "78" or "79" or "80" or "81" or "82" or "83" or "84" or "85" or "86" or "87" or "88" or "89" or "90" or "91" or "92" or "93" or "94" or "95" or "96" or "97" or "98" or "99" or "100") N1 years)	Search modes - Boolean/Phrase	Interface - EBSCOhost Research Databases<br>Search Screen - Advanced Search<br>Database - CINAHL	7,999
S16	TX (aging or ageing or geriatric* or gerontologic* or elderly or "senior citizen*" or seniors or pensioner* or veteran* or sexagenarian* or septuagenarian* or octogenarian* or nonagenarian* or centenarian*)	Search modes - Boolean/Phrase	Interface - EBSCOhost Research Databases<br>Search Screen - Advanced Search<br>Database - CINAHL	523,198
S15	TX ((older or elder*) N2 (person or people or adult* or patient* or m?n* or wom?n* or female* or male*))	Search modes - Boolean/Phrase	Interface - EBSCOhost Research Databases<br>Search Screen - Advanced Search<br>Database - CINAHL	194,690
S14	(MH "Aged+")	Search modes - Boolean/Phrase	Interface - EBSCOhost Research Databases<br>Search Screen - Advanced Search<br>Database - CINAHL	878,186
S13	(MH "Geriatrics")	Search modes - Boolean/Phrase	Interface - EBSCOhost Research Databases<br>Search Screen - Advanced Search<br>Database - CINAHL	5,708

S12	TX (frail*)	Search modes - Boolean/Phrase	Interface - EBSCOhost Research Databases Search Screen - Advanced Search Database - CINAHL	18,976
S11	TX (prefrailty)	Search modes - Boolean/Phrase	Interface - EBSCOhost Research Databases Search Screen - Advanced Search Database - CINAHL	160
S10	S1 OR S2 OR S3 OR S4 OR S5 OR S6 OR S7 OR S8 OR S9	Search modes - Boolean/Phrase	Interface - EBSCOhost Research Databases Search Screen - Advanced Search Database - CINAHL	1,157,497
S9	AB group or AB groups	Search modes - Boolean/Phrase	Interface - EBSCOhost Research Databases Search Screen - Advanced Search Database - CINAHL	795,542
S8	AB trial or AB Trials	Search modes - Boolean/Phrase	Interface - EBSCOhost Research Databases Search Screen - Advanced Search Database - CINAHL	299,002
S7	AB randomly	Search modes - Boolean/Phrase	Interface - EBSCOhost Research Databases Search Screen - Advanced Search Database - CINAHL	96,217
S6	AB (randomised or randomized)	Search modes - Boolean/Phrase	Interface - EBSCOhost Research Databases Search Screen - Advanced Search Database - CINAHL	221,475
S5	TX "randomised controlled trial*"	Search modes - Boolean/Phrase	Interface - EBSCOhost Research Databases Search Screen - Advanced Search Database - CINAHL	26,900
S4	TX "controlled clinical trial*"	Search modes - Boolean/Phrase	Interface - EBSCOhost Research Databases Search Screen - Advanced Search Database - CINAHL	10,403
S3	(MH "Clinical Trials")	Search modes - Boolean/Phrase	Interface - EBSCOhost Research Databases Search Screen - Advanced Search Database - CINAHL	177,904
S2	(MH "Randomized Controlled Trials")	Search modes - Boolean/Phrase	Interface - EBSCOhost Research Databases Search Screen - Advanced Search Database - CINAHL	117,892
S1	TX "randomized controlled trial*"	Search modes - Boolean/Phrase	Interface - EBSCOhost Research Databases Search Screen - Advanced	

Search
Database - CINAHL

3.2.6 Trial registers

To search Clinicaltrials.gov we used the advanced search interface, and searched the Conditions or Disease field using the following search terms: Frail Elderly Syndrome, frailty syndrome, Age-Related Atrophy ,Frailty, Old Age; Debility .The search yielded 861 records

For the International Clinical Trials Registry Platform (ICTRP) we used the advanced search interface, and used the search syntax older or elderly or frail in Title field and community or complex or independent or independence in Intervention field (with synonyms, all recruitment status).The search resulted in 425 records.

Appendix 4. Data items

In addition to outcomes, frailty status and intervention details as specified in the paper, we sought to extract data about:

- reports;
- funding sources;
- country;
- aims;
- RCT design;
- analysis details;
- participant:
    - description,
    - eligibility criteria,
    - number allocated in total and per group;
    - age,
    - gender,
    - living arrangement,
    - carer presence,
    - ethnicity,
    - frailty indicators and measures,
    - health status,
    - dependence and disabilities
    - comorbidities
    - cognitive status
    - mood status
- number of clusters if applicable;
- intervention name and role (experimental/control) per group; and
- further outcomes not specified as of interest for this review.

Appendix 5. Interpretation of the evidence

We described interpretation of the evidence following recent guidance,[20] providing an indication of size and direction if the evidence was not very low certainty. We used the terms ‘probably’ and ‘may’ to indicate moderate and low certainty respectively.[20,21] Where the point estimate was large but the evidence was low certainty we described only the direction in the text as we were not assessing certainty in the size of the estimate; in these cases we described the size alongside the statistical summary.

For effect sizes expressed as SMD we took 0.05 to be the lower bound for a very small effect, 0.16 to be a small effect, 0.38 to be a moderate effect, 0.76 to be a large effect, and 1.2 and above to be a very large effect, based on empirical evidence of effect sizes in gerontological research.[22] We re-expressed SMDs as MDs using a pooled SD for a common measure of the outcome from the included studies.[4]

We re-expressed ORs (and 95% CIs) as RRs using the median risk in the reference comparator arms, and as absolute effects (corresponding intervention risk and corresponding risk difference) for a high and low-risk population, using the highest and lowest risk among the reference comparator arms with more than 100 participants as the assumed comparator risks.[4] By reference to other commonly used interventions in fields such as stroke prevention and hypertension we noted that Number Needed to Treat to Benefit (NNTB) for major outcomes was often between 50 and 100, and sometimes larger.[23-25] Based on this we arbitrarily selected NNTB = 200 as a limit for important difference and used the corresponding risk difference for the high-risk population to define effect sizes as very small (5 per 1000), small (20 per 1000), moderate (40 per 1000), large (60 per 1000) or very large (100 or more per 1000).

Appendix 6. Excluded reports

Table 2 - Table of excluded reports

Excluded report	Reason for exclusion
ACTRN12606000042549[26]	Final planned follow-up was before 24 weeks.
ACTRN12616000521426[27]	The intervention was not initiated and provided in the community.
ACTRN12616001148460[28]	The comparator did not include two or more components (practices, structural elements and contextual factors). intervention
ACTRN12618001188224[29]	The intervention was not focused on sustaining the person's independence.
ACTRN12619000910101[30]	Final planned follow-up was before 24 weeks.
ACTRN12619001055190[31]	The intervention was targeted at specific conditions, rather than addressing independence more generally.
Adelman *et al.*[32]	The intervention was not targeted at the older person.
Ahmad *et al.*[33]	The intervention was not focused on sustaining the person's independence.
Aimonino Ricauda *et al.*[34]	The intervention was targeted at specific conditions, rather than addressing independence more generally.
Akihiro *et al.*[35]	The study was not an RCT/cRCT.
Albert *et al.*[36]	The intervention was not focused on sustaining the person's independence.
Anders *et al.*[37]	The intervention was not initiated and provided in the community.
Andrew *et al.*[38]	The study was not an RCT/cRCT.
Anonymous[39]	The study was not an RCT/cRCT.
Anonymous[40]	The intervention was not focused on sustaining the person's independence.
Anttila *et al.*[41]	The study was not an RCT/cRCT.
Applebaum *et al.*[42]	The intervention was not initiated and provided in the community.
Arendts *et al.*[43]	The intervention was not focused on sustaining the person's independence.
Aung *et al.*[44]	The intervention was not initiated and provided in the community.
Baker *et al.*[45]	The intervention was not focused on sustaining the person's independence.
Ball *et al.*[46]	The intervention did not include two or more components (practices, structural elements and contextual factors).
Ball *et al.*[47]	The intervention did not include two or more components (practices, structural elements and contextual factors).
Bandinelli *et al.*[48]	The intervention was not initiated and provided in the community.
Bardsley *et al.*[49]	The intervention was not focused on sustaining the person's independence.
Bauer[50]	The study was not an RCT/cRCT.
Bauer[51]	The study was not an RCT/cRCT.
Baumann *et al.*[52]	The study was not an RCT/cRCT.
Beck *et al.*[53]	The intervention was not initiated and provided in the community.
Beck *et al.*[54]	The intervention was not initiated and provided in the community.
Beck *et al.*[55]	The intervention was not initiated and provided in the community.
Beck *et al.*[56]	The intervention was not initiated and provided in the community.
Beland *et al.*[57]	The intervention was not initiated and provided in the community.
Belchior *et al.*[58]	The intervention did not include two or more components (practices, structural elements and contextual factors).
Belleville *et al.*[59]	The intervention was not focused on sustaining the person's independence.
Belqaid *et al.*[60]	The intervention was not initiated and provided in the community.
Berglund *et al.*[61]	The intervention was not initiated and provided in the community.
Bernabei *et al.*[62]	The intervention was not initiated and provided in the community.
Binder *et al.*[63]	The intervention was not initiated and provided in the community.
Blanchard *et al.*[64]	The participants were younger than 65 years on average.
Bondoc *et al.*[65]	The intervention was not initiated and provided in the community.
Bonnefoy *et al.*[66]	Final planned follow-up was before 24 weeks.
Bosch-Lenders *et al.*[67]	The intervention did not include two or more components (practices, structural elements and contextual factors).
Botoseneanu *et al.*[68]	The intervention was not initiated and provided in the community.
Boult *et al.*[69]	The intervention was not focused on sustaining the person's independence.
Brandon *et al.*[70]	The intervention was not focused on sustaining the person's independence.

Brazil *et al.*[71] NCT03902743[72]	According to the trial register, the study was still recruiting as of 31 August 2021.
Buford *et al.*[73]	The intervention was not initiated and provided in the community.
Buford *et al.*[74]	The intervention was not initiated and provided in the community.
Burke *et al.*[75]	The intervention was not focused on sustaining the person's independence.
Burton *et al.*[76]	The intervention was not focused on sustaining the person's independence.
Burton *et al.*[77]	The comparator did not include two or more components (practices, structural elements and contextual factors). intervention
Burton *et al.*[78]	The comparator did not include two or more components (practices, structural elements and contextual factors). intervention
Burton *et al.*[79]	The comparator did not include two or more components (practices, structural elements and contextual factors). intervention
Burton *et al.*[80]	The comparator did not include two or more components (practices, structural elements and contextual factors). intervention
Buss *et al.*[81]	The participants were not living at home.
Byles *et al.*[82]	The intervention was not focused on sustaining the person's independence.
Byles *et al.*[83]	The intervention was not focused on sustaining the person's independence.
Callahan *et al.*[84]	The intervention was not initiated and provided in the community.
Caplan *et al.*[85]	The intervention was not initiated and provided in the community.
Carrie *et al.*[86]	The intervention was not initiated and provided in the community.
Carrie *et al.*[87]	The intervention was not initiated and provided in the community.
Cartwright *et al.*[88]	The intervention was not focused on sustaining the person's independence.
Cavaillon[89]	The intervention was not initiated and provided in the community.
Cesari *et al.*[90]	The intervention was not initiated and provided in the community.
Chan *et al.*[91]	The intervention was not initiated and provided in the community.
Chin *et al.*[92]	Final planned follow-up was before 24 weeks.
Ching Wong *et al.*[93]	Final planned follow-up was before 24 weeks.
Choi *et al.*[94]	The intervention did not include two or more components (practices, structural elements and contextual factors).
Clarke *et al.*[95]	The intervention was not focused on sustaining the person's independence.
Coburn *et al.*[96]	The intervention was not focused on sustaining the person's independence.
Cochrane *et al.*[97]	The intervention was not initiated and provided in the community.
Cornu *et al.*[98]	Final planned follow-up was before 24 weeks.
Corrado[99]	The study was not an RCT/cRCT.
Crandall *et al.*[100]	Final planned follow-up was before 24 weeks.
Crews *et al.*[101]	The intervention was targeted at specific conditions, rather than addressing independence more generally.
Ctri *et al.*[102]	The study was not an RCT/cRCT.
Cucinotta *et al.*[103]	The intervention was not focused on sustaining the person's independence.
Cunliffe *et al.*[104]	The comparator was not initiated and provided in the community.
Cwirlej-Sozanska *et al.*[105]	The intervention was not focused on sustaining the person's independence.
Czaja *et al.*[106]	The intervention was not focused on sustaining the person's independence.
Daffner *et al.*[107]	Final planned follow-up was before 24 weeks.
Damanti *et al.*[108]	The intervention was not focused on sustaining the person's independence.
Dangour *et al.*[109]	The intervention was not focused on sustaining the person's independence.
Danilovich *et al.*[110]	The intervention did not include two or more components (practices, structural elements and contextual factors).
Danilovich *et al.*[111]	Final planned follow-up was before 24 weeks.
Dapp *et al.*[112]	The intervention was not initiated and provided in the community.
Dapp *et al.*[113]	The intervention was not initiated and provided in the community.
Dapp *et al.*[114]	The intervention was not initiated and provided in the community.
Dapp *et al.*[115]	The intervention was not initiated and provided in the community.
Dapp *et al.*[116]	The intervention was not initiated and provided in the community.
Dapp *et al.*[117]	The intervention was not initiated and provided in the community.
Dapp *et al.*[118]	The intervention was not initiated and provided in the community.

Dapp *et al.*[119]	The intervention was not initiated and provided in the community.
Darzins *et al.*[120]	The study was not an RCT/cRCT.
Datta *et al.*[121]	The intervention was not initiated and provided in the community.
De Luca *et al.*[122]	The comparator was not initiated and provided in the community.
de Souto Barreto *et al.*[123]	The intervention was not initiated and provided in the community.
De Vreede *et al.*[124]	The comparator did not include two or more components (practices, structural elements and contextual factors). intervention
de Vreede *et al.*[125]	The intervention did not include two or more components (practices, structural elements and contextual factors).
De Vriendt *et al.*[126]	Final planned follow-up was before 24 weeks.
de Vries *et al.*[127]	The participants were not living at home.
Delbaere *et al.*[128]	The study was not an RCT/cRCT.
Delrieu *et al.*[129]	The intervention was not initiated and provided in the community.
Denny *et al.*[130]	Only a conference abstract is available. We cannot confirm the length of follow-up period was at least 24 weeks.
Di Pollina *et al.*[131]	The intervention was not focused on sustaining the person's independence.
Docent *et al.*[132]	The study was not an RCT/cRCT.
Donelle *et al.*[133] ISRCTN79884651[134]	According to the trial register, the study recruitment ended in January 2020. The results were unavailable as of 31 August 2021 and intention to publish data was 31 March 2023.
Dotson *et al.*[135]	The intervention was not initiated and provided in the community.
DRKS00024638[136]	Only the trial register record is available. We cannot confirm whether the intervention was initiated and provided in the community.
Dunn *et al.*[137]	The intervention was not focused on sustaining the person's independence.
Eekhof *et al.*[138]	The study was not an RCT/cRCT.
Elliott *et al.*[139]	The intervention was not initiated and provided in the community.
Englund *et al.*[140]	The comparator did not include two or more components (practices, structural elements and contextual factors). intervention
Espeland *et al.*[141]	The intervention was not initiated and provided in the community.
Espeland *et al.*[142]	The intervention was not initiated and provided in the community.
Falvey *et al.*[143] NCT02905370[144]	According to the trial register, the study is still recruiting as of 31 August 2021.
Fasce *et al.*[145] NCT02052401[146]	Only the protocol and trial register records are available. We cannot confirm whether the participants aged 65 or over on average.
Feingold *et al.*[147]	The study was not an RCT/cRCT.
Feldman *et al.*[148]	The study was not an RCT/cRCT.
Feng *et al.*[149]	The intervention was not focused on sustaining the person's independence.
Ferrat *et al.*[150]	According to the trial register, the study was still recruiting as of 31 August 2021.
Ferreira[151]	Only a conference abstract of the protocol is available. We cannot confirm whether the study has a focus of sustaining independence.
Fielding *et al.*[152]	The intervention was not initiated and provided in the community.
Finkelstein *et al.*[153]	The intervention was targeted at specific conditions, rather than addressing independence more generally.
Finkelstein *et al.*[154]	The intervention was targeted at specific conditions, rather than addressing independence more generally.
Finkelstein *et al.*[155]	The intervention was targeted at specific conditions, rather than addressing independence more generally.
Fisher *et al.*[156]	The intervention was not focused on sustaining the person's independence.
Fletcher *et al.*[157]	The participants were not living at home.
Fletcher *et al.*[158]	The participants were not living at home.
Fontan[159]	The study was not an RCT/cRCT.
Fontan[160]	The study was not an RCT/cRCT.
Forbes[161]	The study was not an RCT/cRCT.
France *et al.*[162]	The study was not an RCT/cRCT.
Franse *et al.*[163]	The study was not an RCT/cRCT.
Frese *et al.*[164]	The study was not an RCT/cRCT.
Friedberg[165]	The study was not an RCT/cRCT.
Frieswijk *et al.*[166]	The intervention did not include two or more components (practices, structural elements and contextual factors).
Fritz *et al.*[167]	Final planned follow-up was before 24 weeks.

Gagnon *et al.*[168]	The comparator was not initiated and provided in the community.
Gasmann *et al.*[169]	The study was not an RCT/cRCT.
Geller *et al.*[170]	The intervention was not focused on sustaining the person's independence.
Giannini *et al.*[171]	The study was not an RCT/cRCT.
Gillette[172]	The intervention was not initiated and provided in the community.
Gillette-Guyonnet *et al.*[173]	The intervention was not initiated and provided in the community.
Ginis *et al.*[174]	The intervention was not initiated and provided in the community.
Giudici *et al.*[175]	The intervention was not initiated and provided in the community.
Giudici *et al.*[176]	The intervention was not initiated and provided in the community.
Godwin *et al.*[177]	The intervention was not focused on sustaining the person's independence.
Godwin *et al.*[178]	The intervention was not focused on sustaining the person's independence.
Golas *et al.*[179]	The intervention was not focused on sustaining the person's independence.
Goldberg *et al.*[180]	The intervention was not focused on sustaining the person's independence.
Gorenberg[181]	The study was not an RCT/cRCT.
Granbom *et al.*[182]	The intervention was not focused on sustaining the person's independence.
Green *et al.*[183]	The study was not an RCT/cRCT.
Groessl *et al.*[184]	The intervention was not initiated and provided in the community.
Gross *et al.*[185]	The intervention did not include two or more components (practices, structural elements and contextual factors).
Gross *et al.*[186]	The intervention did not include two or more components (practices, structural elements and contextual factors).
Gross *et al.*[187]	The intervention did not include two or more components (practices, structural elements and contextual factors).
Guerville *et al.*[188]	The intervention was not initiated and provided in the community.
Gunner-Svensson *et al.*[189]	The intervention was not focused on sustaining the person's independence.
Gunzelmann *et al.*[190]	The study was not an RCT/cRCT.
Guralnik *et al.*[191]	The intervention was not focused on sustaining the person's independence.
Guyonnet Sophie *et al.*[192]	The intervention was not initiated and provided in the community.
Hagen *et al.*[193]	The study was not an RCT/cRCT.
Hall[194]	The study was not an RCT/cRCT.
Hammar *et al.*[195]	The intervention was not initiated and provided in the community.
Hansen *et al.*[196]	The study was not an RCT/cRCT.
Hansen *et al.*[197]	The study was not an RCT/cRCT.
Hansen *et al.*[198]	The intervention was not focused on sustaining the person's independence.
Henderson *et al.*[199]	The intervention was not focused on sustaining the person's independence.
Henderson *et al.*[200]	The intervention was not focused on sustaining the person's independence.
Henderson *et al.*[201]	The intervention was not initiated and provided in the community.
Hernandez-Ascanio *et al.*[202]	The intervention was not focused on sustaining the person's independence.
Hinkka *et al.*[203]	The intervention was not initiated and provided in the community.
Hirani *et al.*[204]	The intervention was not focused on sustaining the person's independence.
Hitzel *et al.*[205]	The intervention was not initiated and provided in the community.
Hochhalter *et al.*[206]	The intervention was targeted at specific conditions, rather than addressing independence more generally.
Hooper *et al.*[207]	The intervention was not initiated and provided in the community.
Hopp *et al.*[208]	The intervention was not focused on sustaining the person's independence.
Hsieh *et al.*[209]	The intervention was not focused on sustaining the person's independence.
Hsieh *et al.*[210]	The intervention was not initiated and provided in the community.
Hsin *et al.*[211]	Final planned follow-up was before 24 weeks.
Hsu *et al.*[212]	The intervention was not initiated and provided in the community.
Hughes *et al.*[213]	The intervention was not initiated and provided in the community.
ISRCTN13927531[214]	According to the trial register, the overall trial end data is 31 May 2023.
ISRCTN16123291[215]	According to the trial register, the overall trial end data is 30 March 2023.
ISRCTN52788952[216]	The study was not an RCT/cRCT.

ISRCTN54268283[217]	According to the trial register, the study was still recruiting as of 31 August 2021.
ISRCTN57066881[218]	Final planned follow-up was before 24 weeks.
Jackson *et al.*[219]	The study was not an RCT/cRCT.
JPRN-UMIN000003877[220]	Final planned follow-up was before 24 weeks.
JPRN-UMIN000004767[221]	Final planned follow-up was before 24 weeks.
JPRN-UMIN000022992[222]	Final planned follow-up was before 24 weeks.
JPRN-UMIN000026448[223]	Final planned follow-up was before 24 weeks.
June *et al.*[224]	The study was not an RCT/cRCT.
Kallio *et al.*[225]	The study was not an RCT/cRCT.
Katula *et al.*[226]	The intervention was not initiated and provided in the community.
Kerr *et al.*[227]	The intervention was targeted at specific conditions, rather than addressing independence more generally.
Kerse *et al.*[228]	The intervention was not focused on sustaining the person's independence.
Kerse *et al.*[229]	The intervention was not focused on sustaining the person's independence.
Kim *et al.*[230]	The intervention was not focused on sustaining the person's independence.
Kim *et al.*[231]	The comparator did not include two or more components (practices, structural elements and contextual factors). intervention
King *et al.*[232]	The study was not an RCT/cRCT.
Kinney *et al.*[233]	The intervention was not focused on sustaining the person's independence.
Kivipelto *et al.*[234]	The intervention was not initiated and provided in the community.
Kivipelto *et al.*[235]	The study was not an RCT/cRCT.
Kivipelto *et al.*[236]	The intervention was not initiated and provided in the community.
Klompstra *et al.*[237]	The intervention was not initiated and provided in the community.
Kolbe-Alexander *et al.*[238]	The study was not an RCT/cRCT.
Kravitz *et al.*[239]	The study was not an RCT/cRCT.
Kristensson *et al.*[240]	The intervention was not focused on sustaining the person's independence.
Kwon *et al.*[241]	The intervention was not focused on sustaining the person's independence.
Latham *et al.*[242]	The intervention was not initiated and provided in the community.
Lewin *et al.*[243]	The study was not an RCT/cRCT.
Li *et al.*[244]	The intervention was not initiated and provided in the community.
Liang *et al.*[245]	The intervention was not initiated and provided in the community.
LIFE Study Investigators[246]	The intervention was not initiated and provided in the community.
Lihavainen *et al.*[247]	The participants were not living at home.
Lilamand *et al.*[248]	The intervention was not initiated and provided in the community.
Lim *et al.*[249]	The intervention was not initiated and provided in the community.
Lin *et al.*[250]	The intervention did not include two or more components (practices, structural elements and contextual factors).
Lin *et al.*[251]	The intervention did not include two or more components (practices, structural elements and contextual factors).
Liu *et al.*[252]	The intervention was not initiated and provided in the community.
Liu *et al.*[253]	The comparator did not include two or more components (practices, structural elements and contextual factors). intervention
Liu *et al.*[254]	Final planned follow-up was before 24 weeks.
Lohman *et al.*[255]	The intervention did not include two or more components (practices, structural elements and contextual factors).
Lorig *et al.*[256]	The intervention was not focused on sustaining the person's independence.
Luger *et al.*[257]	The intervention was not focused on sustaining the person's independence.
Lum *et al.*[258]	The intervention was not focused on sustaining the person's independence.
Lurie *et al.*[259]	The intervention was not targeted at the older person.
Lyndon *et al.*[260] ISRCTN74345449[261]	According to the trial register, the study results were unavailable as of 31 August 2021 and intention to publish data is 30 April 2022.
Mangin *et al.*[262]	The intervention was not focused on sustaining the person's independence.
Mankowski *et al.*[263]	The intervention was not initiated and provided in the community.
Marcusson *et al.*[264]	The study was not an RCT/cRCT.
Marsh *et al.*[265]	The intervention was not initiated and provided in the community.

Marsh *et al.*[266]	The intervention was not initiated and provided in the community.
Martin *et al.*[267]	The participants were not living at home.
Martin Lesende[268]	The study was not an RCT/cRCT.
Matthews *et al.*[269]	The intervention was not initiated and provided in the community.
Mayer *et al.*[270]	The intervention was not focused on sustaining the person's independence.
McDermott *et al.*[271]	The intervention was not initiated and provided in the community.
McDougall *et al.*[272]	The intervention was not focused on sustaining the person's independence.
McDowell *et al.*[273]	Final planned follow-up was before 24 weeks.
McEwan *et al.*[274]	The participants were not living at home.
McFarland[275]	The study was not an RCT/cRCT.
McMurdo *et al.*[276]	The comparator did not include two or more components (practices, structural elements and contextual factors). intervention
McWilliam *et al.*[277]	The intervention was not focused on sustaining the person's independence.
Meiling[278]	The study was not an RCT/cRCT.
Melin *et al.*[279]	The comparator was not initiated and provided in the community.
Melin *et al.*[280]	The comparator was not initiated and provided in the community.
Melin *et al.*[281]	The comparator was not initiated and provided in the community.
Merete Pedersen *et al.*[282]	The intervention was not initiated and provided in the community.
Meuleman[283]	The study was not an RCT/cRCT.
Meziere[284]	Final planned follow-up was before 24 weeks.
Miller *et al.*[285]	The comparator was not initiated and provided in the community.
Mohd Suffian *et al.*[286]	According to the trial register, the study has not started recruitment as of 31 August 2021.
Moller *et al.*[287]	The intervention was not focused on sustaining the person's independence.
Moon *et al.*[288]	The intervention was not initiated and provided in the community.
Mor *et al.*[289]	The participants were younger than 65 years on average.
Mortenson *et al.*[290]	Final planned follow-up was before 24 weeks.
Mortsiefer *et al.*[291] DRKS00015055[292]	According to the trial register, the study recruitment closed on 30 June 2021 and the study was ongoing as of 31 August 2021.
Mountain *et al.*[293]	The intervention was not focused on sustaining the person's independence.
Mugueta-Aguinaga *et al.*[294]	The intervention did not include two or more components (practices, structural elements and contextual factors).
Mugueta-Aguinaga *et al.*[295]	The intervention did not include two or more components (practices, structural elements and contextual factors).
NCT00452465[296]	The intervention was not focused on sustaining the person's independence.
NCT00672685[297]	The intervention was not initiated and provided in the community.
NCT01345032[298]	Final planned follow-up was before 24 weeks.
NCT02021565[299]	Final planned follow-up was before 24 weeks.
NCT02335177[300]	Final planned follow-up was before 24 weeks.
NCT02545257[301]	The intervention was not focused on sustaining the person's independence.
NCT02554838[302]	The intervention was targeted at specific conditions, rather than addressing independence more generally.
NCT02582138[303]	The intervention was not initiated and provided in the community.
NCT02847871[304]	The intervention was not initiated and provided in the community.
NCT02923843[305]	The intervention was not initiated and provided in the community.
NCT02942992[306]	The intervention did not include two or more components (practices, structural elements and contextual factors).
NCT03147625[307]	According to the trial register, the study recruitment status was unknown and no results were available as of 31 August 2021.
NCT03180606[308]	The study was not an RCT/cRCT.
NCT03212859[309]	The participants were not living at home.
NCT03336320[310]	The intervention was not focused on sustaining the person's independence.
NCT03342976[311]	The study was not an RCT/cRCT.
NCT03394495[312]	The intervention was not focused on sustaining the person's independence.
NCT03394534[313]	According to the trial register, the study was still recruiting as of 31 August 2021.
NCT03456128[314]	According to the trial register, the study was still recruiting as of 31 August 2021.

NCT03474380[315]	The intervention was not initiated and provided in the community.
NCT03568084[316]	The intervention did not include two or more components (practices, structural elements and contextual factors).
NCT03577002[317]	The intervention was not focused on sustaining the person's independence.
NCT03591055[318]	According to the trial register, the study recruitment status was unknown and not results were available as of 31 August 2021.
NCT03634033[319]	The comparator was not targeted at the older person.
NCT03649698[320]	According to the trial register, the study was still recruiting as of 31 August 2021.
NCT03797352[321]	The intervention was not focused on sustaining the person's independence.
NCT03814161[322]	The study was not an RCT/cRCT.
NCT03824106[323]	According to the trial register, the study has not started recruitment as of 31 August 2021.
NCT03952858[324]	The comparator did not include two or more components (practices, structural elements and contextual factors). intervention
NCT03979560[325]	According to the trial register, the study recruitment status was unknown and not results were available as of 31 August 2021.
NCT04076319[326]	The study was not an RCT/cRCT.
NCT04416815[327]	According to the trial register, the study was still recruiting as of 31 August 2021.
NCT04460742[328]	According to the trial register, the study was still recruiting as of 31 August 2021.
NCT04500366[329]	According to the trial register, the study was still recruiting as of 31 August 2021.
NCT04531852[330]	The intervention was not focused on sustaining the person's independence.
NCT04574271[331]	Only the trial register record is available. We cannot confirm whether the study is an RCT, and whether the intervention is initiated and provided in the community.
NCT04628754[332]	The intervention was not focused on sustaining the person's independence.
Nelson *et al.*[333]	The intervention was not focused on sustaining the person's independence.
Neumann *et al.*[334]	The intervention was not initiated and provided in the community.
Newbury *et al.*[335]	The study was not an RCT/cRCT.
Ngandu *et al.*[336]	The intervention was not initiated and provided in the community.
Nice[337]	The study was not an RCT/cRCT.
Nicklas *et al.*[338]	The intervention was not initiated and provided in the community.
Nielsen *et al.*[339]	The intervention was not focused on sustaining the person's independence.
O'Connell *et al.*[340]	The participants were younger than 65 years on average.
O'Connor *et al.*[341]	The intervention was targeted at specific conditions, rather than addressing independence more generally.
O'Connor *et al.*[342]	The intervention was targeted at specific conditions, rather than addressing independence more generally.
Oksman *et al.*[343]	The intervention was not focused on sustaining the person's independence.
Olesen *et al.*[344]	The intervention was not focused on sustaining the person's independence.
Oliva[345]	The intervention was not focused on sustaining the person's independence.
Ollonqvist *et al.*[346]	The intervention was not initiated and provided in the community.
Ollonqvist *et al.*[347]	The intervention was not initiated and provided in the community.
Olsson *et al.*[348]	The intervention was not focused on sustaining the person's independence.
Olsson Möller *et al.*[349]	The intervention was specifically a falls prevention programme.
Olsson Möller *et al.*[350]	The intervention was specifically a falls prevention programme.
Opdenacker *et al.*[351]	The intervention was not focused on sustaining the person's independence.
Opdenacker *et al.*[352]	The intervention was not focused on sustaining the person's independence.
Ory *et al.*[353]	The study was not an RCT/cRCT.
Osborn *et al.*[354]	The participants were not living at home.
Osborn *et al.*[355]	The participants were not living at home.
Osborn *et al.*[356]	The participants were not living at home.
Oswald *et al.*[357]	The study was not an RCT/cRCT.
Oswald *et al.*[358]	The study was not an RCT/cRCT.
Oswald *et al.*[359]	The study was not an RCT/cRCT.
Oswald *et al.*[360]	The study was not an RCT/cRCT.
Overbeek *et al.*[361]	The participants were not living at home.

Overbeek *et al.*[362]	The participants were not living at home.
Pacini *et al.*[363]	The intervention was not focused on sustaining the person's independence.
Palacholla *et al.*[364]	The intervention was not focused on sustaining the person's independence.
Pardessus *et al.*[365]	The intervention was not initiated and provided in the community.
Parsons *et al.*[366]	The participants were not living at home.
Peak *et al.*[367]	The study was not an RCT/cRCT.
Pedersen *et al.*[368]	Final planned follow-up was before 24 weeks.
Peri *et al.*[369]	According to the trial register, study recruitement was completed in February 2019 and the final follow-up is 4 years post-intervention; no results were avaiable as of 31 August 2021.
Perkel *et al.*[370]	The study was not an RCT/cRCT.
Perman *et al.*[371]	The study was not an RCT/cRCT.
Persson *et al.*[372]	Final planned follow-up was before 24 weeks.
Petersson *et al.*[373]	The study was not an RCT/cRCT.
Phillips *et al.*[374]	The intervention was not initiated and provided in the community.
Picarsic *et al.*[375]	The intervention was not initiated and provided in the community.
Prossegger *et al.*[376]	The intervention was not focused on sustaining the person's independence.
Rantanen *et al.*[377]	The intervention was not focused on sustaining the person's independence.
Rebok *et al.*[378]	The intervention did not include two or more components (practices, structural elements and contextual factors).
Rebok *et al.*[379]	The intervention did not include two or more components (practices, structural elements and contextual factors).
Rejeski *et al.*[380]	The intervention was not initiated and provided in the community.
Rejeski *et al.*[381]	The intervention was not initiated and provided in the community.
Reuben *et al.*[382]	The intervention was not focused on sustaining the person's independence.
Reuben[383]	The intervention was not initiated and provided in the community.
Rexroth *et al.*[384]	The intervention did not include two or more components (practices, structural elements and contextual factors).
Ribera *et al.*[385]	The study was not an RCT/cRCT.
Richardson *et al.*[386]	The intervention was not initiated and provided in the community.
Rietkerk *et al.*[387]	The study was not an RCT/cRCT.
Rikard *et al.*[388]	The intervention did not include two or more components (practices, structural elements and contextual factors).
Ristolainen *et al.*[389]	The intervention was not focused on sustaining the person's independence.
Rivas-Ruiz *et al.*[390] ISRCTN17143761[391]	According to the trial register, study recruitement was suspended; no results were avaiable as of 31 August 2021.
Robichaud *et al.*[392]	Final planned follow-up was before 24 weeks.
Rodrigues *et al.*[393]	The study was not an RCT/cRCT.
Rollins[394]	The study was not an RCT/cRCT.
Rosie *et al.*[395]	The comparator did not include two or more components (practices, structural elements and contextual factors). intervention
Rosstad *et al.*[396]	The participants were not living at home.
Rubenstein *et al.*[397]	The participants were younger than 65 years on average.
Ruikes *et al.*[398]	The study was not an RCT/cRCT.
Rydwik *et al.*[399]	The study was not an RCT/cRCT.
Rydwik *et al.*[400]	The study was not an RCT/cRCT.
Rydwik *et al.*[401]	The study was not an RCT/cRCT.
Saeterbakken *et al.*[402]	The intervention was not focused on sustaining the person's independence.
Sahlen *et al.*[403]	The study was not an RCT/cRCT.
Saito *et al.*[404]	The intervention was not focused on sustaining the person's independence.
Salem *et al.*[405]	The participants were younger than 65 years on average.
Sandberg *et al.*[406]	The intervention was not focused on sustaining the person's independence.
Sandberg *et al.*[407]	The intervention was not focused on sustaining the person's independence.
Sanders *et al.*[408]	The intervention was not focused on sustaining the person's independence.
Sanjuan *et al.*[409]	The participants were not living at home.
Santanasto *et al.*[410]	The intervention was not initiated and provided in the community.

Sato *et al.*[411]	The intervention did not include two or more components (practices, structural elements and contextual factors).
Schraeder *et al.*[412]	The intervention was not focused on sustaining the person's independence.
Scott *et al.*[413]	The study was not an RCT/cRCT.
Senior *et al.*[414]	The intervention was not initiated and provided in the community.
Sherwood[415]	The intervention was not initiated and provided in the community.
Sink *et al.*[416]	The intervention was not initiated and provided in the community.
Sisco *et al.*[417]	The intervention did not include two or more components (practices, structural elements and contextual factors).
Smeeth *et al.*[418]	The participants were not living at home.
Smeeth *et al.*[419]	The participants were not living at home.
Smith *et al.*[420]	Final planned follow-up was before 24 weeks.
Spoelstra *et al.*[421]	The comparator was not targeted at the older person.
Spoelstra *et al.*[422]	The comparator was not targeted at the older person.
Spoorenberg *et al.*[423]	The participants were not living at home.
Spoorenberg *et al.*[424]	The participants were not living at home.
Stathi *et al.*[425] Withall *et al.*[426] ISRCTN45627165[427]	According to the trial register, the overall trial end data was 31 May 2020; the intention to publish date was 22 June 2021, but results were unavailable as of 31 August 2021.
Steventon *et al.*[428]	The intervention was not focused on sustaining the person's independence.
Steventon *et al.*[429]	The intervention was not focused on sustaining the person's independence.
Stewart *et al.*[430]	The intervention was not focused on sustaining the person's independence.
Summers *et al.*[431]	The study was not an RCT/cRCT.
Tarazona-Santabalbina *et al.*[432]	The study was not an RCT/cRCT.
Taube *et al.*[433]	The intervention was not focused on sustaining the person's independence.
Teh *et al.*[434]	According to the trial register, the study data collection ended in September 2020; no results were available as of 31 August 2021.
Tennstedt *et al.*[435]	The intervention was not focused on sustaining the person's independence.
Tennstedt *et al.*[436]	The intervention did not include two or more components (practices, structural elements and contextual factors).
Thom *et al.*[437]	The intervention was not focused on sustaining the person's independence.
Tieland *et al.*[438]	The comparator did not include two or more components (practices, structural elements and contextual factors). intervention
Timonen *et al.*[439]	The intervention was not initiated and provided in the community.
Toivo *et al.*[440]	The intervention was not focused on sustaining the person's independence.
Toledano-González *et al.*[441]	The participants were not living at home.
Townsend *et al.*[442]	The intervention was not initiated and provided in the community.
Trombini-Souza *et al.*[443]	The intervention did not include two or more components (practices, structural elements and contextual factors).
Turunen *et al.*[444]	The intervention was not focused on sustaining the person's independence.
Uittenbroek *et al.*[445]	The participants were not living at home.
Uittenbroek *et al.*[446]	The participants were not living at home.
Ukawa *et al.*[447]	The intervention was not focused on sustaining the person's independence.
Ukawa *et al.*[448]	The intervention was not focused on sustaining the person's independence.
Ukawa *et al.*[449]	Final planned follow-up was before 24 weeks.
Ukawa *et al.*[450]	The intervention was not focused on sustaining the person's independence.
van de Sant *et al.*[451]	The participants were not living at home.
van den Helder *et al.*[452]	The intervention was not focused on sustaining the person's independence.
van den Helder *et al.*[453]	The comparator did not include two or more components (practices, structural elements and contextual factors). intervention
van Haaren[454]	The study was not an RCT/cRCT.
van Haaren[455]	The study was not an RCT/cRCT.
Vaz Fragoso *et al.*[456]	The intervention was not initiated and provided in the community.
Vaz Fragoso *et al.*[457]	The intervention was not initiated and provided in the community.
Vaz Fragoso *et al.*[458]	The intervention was not initiated and provided in the community.
Vellas *et al.*[459]	The intervention was not initiated and provided in the community.

Venturelli *et al.*[460]}	The participants were not living at home.
Vetter *et al.*[461]	The intervention was not focused on sustaining the person's independence.
Vienna[462]	Final planned follow-up was before 24 weeks.
von Renteln-Kruse *et al.*[463]	The intervention was not initiated and provided in the community.
Wadley *et al.*[464]	The intervention did not include two or more components (practices, structural elements and contextual factors).
Wagner *et al.*[465]	The intervention was specifically a falls prevention programme.
Walker *et al.*[466]	The intervention was targeted at specific conditions, rather than addressing independence more generally.
Walker *et al.*[467]	The intervention was not focused on sustaining the person's independence.
Wallace *et al.*[468]	The intervention did not include two or more components (practices, structural elements and contextual factors).
Wallen *et al.*[469]	The study was not an RCT/cRCT.
Wan *et al.*[470]	The intervention was not initiated and provided in the community.
Wang *et al.*[471]	The intervention was not initiated and provided in the community.
Wasson *et al.*[472]	The intervention was not initiated and provided in the community.
Watanabe *et al.*[473]	The intervention was not focused on sustaining the person's independence.
Watanabe *et al.*[474]	Final planned follow-up was before 24 weeks.
White *et al.*[475]	The participants were not living at home.
Whitehead *et al.*[476]	The intervention did not include two or more components (practices, structural elements and contextual factors).
Whitehead *et al.*[477]	The intervention did not include two or more components (practices, structural elements and contextual factors).
Wilber *et al.*[478]	The intervention was not focused on sustaining the person's independence.
Wilhelmson *et al.*[479]	The intervention was not initiated and provided in the community.
Williams *et al.*[480]	The intervention was not focused on sustaining the person's independence.
Williamson *et al.*[481]	The intervention was not initiated and provided in the community.
Willis *et al.*[482]	The intervention did not include two or more components (practices, structural elements and contextual factors).
Wilson *et al.*[483]	The participants were younger than 65 years on average.
Wolf *et al.*[484]	Final planned follow-up was before 24 weeks.
Wolinsky *et al.*[485]	The intervention did not include two or more components (practices, structural elements and contextual factors).
Wong *et al.*[486]	The intervention was not initiated and provided in the community.
Wong *et al.*[487]	The intervention was not focused on sustaining the person's independence.
Wong *et al.*[488]	Final planned follow-up was before 24 weeks.
Wooldridge *et al.*[489]	The study was not an RCT/cRCT.
Xie *et al.*[490]	Final planned follow-up was before 24 weeks.
Yao *et al.*[491]	The intervention was not initiated and provided in the community.
Yeo *et al.*[492]	The intervention was not initiated and provided in the community.
Yim *et al.*[493]	The intervention was not initiated and provided in the community.
Yoon *et al.*[494]	The intervention was not initiated and provided in the community.
Young *et al.*[495]	The study was not an RCT/cRCT.
Yu *et al.*[496]	The intervention was not focused on sustaining the person's independence.
Zaragoza[497]	The intervention did not include two or more components (practices, structural elements and contextual factors).
Zauszniewski *et al.*[498]	Final planned follow-up was before 24 weeks.
Zhu *et al.*[499]	The intervention was not initiated and provided in the community.
Zijlstra *et al.*[500]	The intervention was not focused on sustaining the person's independence.
Zillich *et al.*[501]	The intervention was not focused on sustaining the person's independence.
Zimmer *et al.*[502]	The intervention was not focused on sustaining the person's independence.

Appendix 7. Included studies and reports

Table 3 - Summary characteristics of 129 included studies .[503-631]

Study	Design	Enrolment began	Country	Population frailty	Population age, years	enrolled	Interventions	Controls	Funding
Alegria 2019[503,632,633]	RCT	2015	USA	P	60-64: 7%; ≥65: 93%	307	exrc & psyc	ac	NC
Arthanat 2019[504,634,635]	RCT	> 2005	USA	U	76 (7); ≥65	97	comm	ac	NC
Auvinen 2020[505,636-638]	RCT	2015	FIN	F	84 (7); ≥65	512	hmcr & med	hmcr	NC
Balaban 1988[506]	RCT	1981	USA	F	68; 17–99*; ≥65: 72%	198	mfa-(w/med)	ac	NC
Barenfeld 2018[507,639-642]	RCT	2012	SWE	all	74 (3); 70–84	131	educ	ac	NC
Bernabei 1998[508,643,644]	RCT	1995	ITA	F	81 (7); ≥65	200	hmcr & mfar(w/med)	hmcr	NC
Bleijenberg 2016[509,645-653]	cRCT	2010	NLD	P,F	74 (8); ≥60	m:39; 3092	rsk-mfa-; rsk-mfa-	ac	NC
Blom 2016[510,652,654,655]	cRCT	2009	NLD	all	~83 [79–87]; ≥75	m:59; 1379	mfa-(w/med+slfm)	ac	NC
Borrows 2013[511]	RCT	2008	GBR	U	70 (14); ≥16	36	aids	mfa-	NC
Botjes 2013[512,656,657]	RCT	2011	NLD	U	77 (7); ≥65	218	mfa-	ac	NC
Bouman 2008[513,658-662]	RCT	2002	NLD	P,F	76 (4); ≥70	330	mfar(w/med)	ac	NC
Brettschneider 2015[514,663-666]	RCT	2007	DEU	F	85 (4); ≥80	336	mfar(w/med)	ac	NC
Cameron 2013[515,667-676]	RCT	2008	AUS	F	83 (6); ≥70	241	exrc & mfar(w/med+slfm)	ac	NC
Carpenter 1990[516]	RCT	< 2006	GBR	all	≥75	539	rsk-m+fa-	ac	NC
Cesari 2014[517,677-682]	RCT	> 2005	FRA	U	73 (8); ≥60	?	mfar(w/med)	ac	NC
Challis 2004[518,683]	RCT	1998	GBR	F	82 (8); ≥60	256	mfar(w/med)	mfar	NC
Clark 1997[519,684-688]	RCT	1994	USA	R,P	74 (7); ≥60	361	eng & educ	ac	Mx
Clark 2012[520,689-694]	RCT	2004	USA	U	75 (8); 60–95	460	eng & educ	ac	NC
Coleman 1999[521]	cRCT	< 2006	USA	F	≥65	m:9; 169	educ & mfar(w/med+slfm)	ac	NC
Counsell 2007[522,695-699]	cRCT	2002	USA	U	72 (6); ≥65	m:164; 951	educ & mfar(w/med+slfm)	ac	NC
Cutchin 2009[523,700]	RCT	2008	USA	U	82 (5); ≥75	110	mfar	ac	NC
Dalby 2000[524,701]	RCT	< 2006	CAN	F	79 (6); ≥70	142	mfar(w/med)	ac	NC
de Craen 2006[525,702-704]	RCT	2000	NLD	all	≥85	402	mfa-	ac	NC
Dorresteijn 2016[526,705-708]	RCT	2009	NLD	U	78 (5); ≥70	389	ADL	ac	NC
Dupuy 2017[527,709]	RCT	> 2005	FRA	P,F	82 (2); ≥70	32	hmcr & aids & comm	hmcr	NC
Fabacher 1994[528]	RCT	< 2006	USA	all	73 (6); ≥70	254	mfar(w/med)	ac	NC
Fairhall 2015[529 710,711]	RCT	2013	AUS	P	82 (5); ≥70	230	mfar(w/med)	ac	NC
Faul 2009[530,712]	RCT	?	USA	R,P	77 (7); ≥65	81	educ & exrc & mfar(w/med+slfm); exrc & mfa-(w/med+slfm)		NC
Fernandez-Barres 2017[531,713,714]	RCT	2010	ESP	F	85 (7); ≥65	173	hmcr & ntr	hmcr	NC
Fischer 2009[532,715]	RCT	2004	DEU	all	67–80	4224	eng & mfa-(w/slfm)	ac	?
Ford 1971[533,716]	RCT	1963	USA	P,F	72; 50–94	300	mfar(w/med)	ac	NC
Fox 1997[534]	RCT	1994	USA	all	50-69: 53%; ≥70: 47%	237	mfar(w/med+slfm)	mfar(w/med)	NC
Fristedt 2019[535,717]	RCT	2015	SWE	F	85 (6); ≥75	62	hmcr & mfar(w/med)	hmcr	NC
Gene Huguet 2018[536]	RCT	2016	ESP	P	85 (5); ≥80	200	med & ntr & exrc	ac	NC
Gill 2002[537,718-721]	RCT	< 2006	USA	P,F	83 (5); ≥75	188	ADL & exrc	ac	NC
Giné-Garriga 2020[538,722-732]	RCT	2016	EEE	R	75 (6); ≥65	1360	exrc	ac	NC

Study	Design	Enrolment began	Country	Population frailty	Population age, years	enrolled	Interventions	Controls	Funding
Gitlin 2006[539,733-743]	RCT	2003	USA	P,F	79 (6); ≥70	319	ADL & aids & exrc	ac	NC
Grimmer 2013[540,744]	RCT	2014	AUS	U	≥65	?	mfa-	ac	?
Gustafson 2021[541,745,746]	RCT	2013	USA	all	77 (7); ≥65	390	aids & educ & comm	ac	Mx
Gustafsson 2013[542,639,747-754]	RCT	2007	SWE	all	~86; 80–97	491	educ & mfa-;<br>educ	ac	NC
Hall 1992[543]	RCT	1986	CAN	F	78 (7); ≥65	167	hmcr & mfar(w/slfm)	hmcr & mfar	?
Harari 2008[544 755-769]	RCT	2000	GBR	all	75 (6); ≥65	2503	mfar(w/med)	ac	NC
Hattori 2019[545,770]	RCT	2018	JPN	P,F	≥65	375	educ & mfar(w/slfm)	mfar	NC
Hay 1998[546,771]	RCT	< 2006	CAN	U	75 (6); ?	619	mfa-	ac;<br>ac	NC
Hebert 2001[547]	RCT	< 2006	CAN	P,F	80 (4); ≥75	503	mfar(w/med)	ac	NC
Henderson 2005[548,772]	cRCT	2002	AUS	R	82 (5); 75–94	m:16; 167	mfar	ac	NC
Hendriksen 1984[549,773-775]	RCT	1980	DNK	all	~78; 75–96	600	mfar	ac	NC
Hogg 2009[550,776-780]	RCT	2004	CAN	U	71; ≥50	241	mfar(w/med)	ac	NC
Holland 2005[551,781,782]	RCT	2001	USA	U	73 (5); ≥65	504	educ & exrc & mfar(w/slfm)	ac	NC
Howel 2019[552,783-785]	RCT	2011	GBR	all	71 (7); ≥60	755	wlfr	ac	NC
Imhof 2012[553,653]	RCT	2008	CHE	all	85 (4); ≥80	461	mfar	ac	NC
Jing 2018[554]	RCT	2016	CHN	F	75 (6); 60–85	80	psyc;<br>exrc & psyc		?
Jitapunkul 1998[555]	RCT	1993	THA	U	76 (6); ≥70	160	rsk-mfa-	ac	NC
Kerse 2014[556,786-790]	cRCT	2008	NZL	P,F	80 (5); ≥65*	m:60; 3893	rsk-mfa-	ac	NC
King 2012[557,791-793]	cRCT	2006	NZL	P,F	79 (7); ≥65	m:21; 186	hmcr & ADL & mfar(w/slfm)	hmcr	NC
Kono 2016[558,794,795]	RCT	2011	JPN	P	79 (6); ≥65	360	mfar(w/med)	mfar	NC
Kono 2004[559]	RCT	2000	JPN	P,F	83 (7); ≥65	119	mfar	ac	NC
Kono 2012[560,796-798]	RCT	2008	JPN	P	80 (7); ≥65	323	mfar	mfar	NC
Kukkonen-Harjula 2017[561,799-803]	RCT	2014	FIN	P,F	83 (6); ≥65	300	ADL & ntr & exrc	ac	NC
Lambotte 2018[562,804-811]	RCT	2017	BEL	P,F	75 (9); ≥60	871	mfar	ac	NC
Leung 2004[563,812]	RCT	2000	HKG	all	75 (7); ≥60	260	mfar(w/med)	ac	?
Leveille 1998[564,813,814]	RCT	1995	USA	U	77 (5); ≥70	201	educ & exrc & mfar(w/med+slfm)	ac	NC
Lewin 2013[565,815-817]	RCT	2005	AUS	F	82 (8); ≥65	750	hmcr & educ & mfar	hmcr	NC
Liddle 1996[566]	RCT	< 2006	AUS	U	82 (6); 69–94	105	aids & mfar	ac	NC
Liimatta 2019[567,818-820]	RCT	2013	FIN	R,P	81 (4); ≥75	422	exrc & mfa-(w/med)	ac	NC
Loh 2015[568,821,822]	cRCT	2014	MYS	U	68 (6); ≥60	m:8; 256	ntr & exrc	ac	NC
Lood 2015[569]	RCT	2012	SWE	R,P	76 (3); 71–85	40	educ	ac	NC
Mann J 2021[570,823-827]	cRCT	2018	AUS	all	~81 [77–85];<br>≥50*	m:14; 92	mfa-(w/med)	ac	NC
Mann WC 1999[571]	RCT	< 2006	USA	F	73 (8); ?	104	hmcr & aids	hmcr	NC
Markle-Reid 2006[572,828,829]	RCT	2001	CAN	F	≥75	288	hmcr & mfar(w/med+slfm)	hmcr & mfar	NC
Melis 2008[573,830-836]	RCT	2003	NLD	F	82 (6); 69–99	155	mfar(w/med)	ac	NC
Meng 2005[574,837-843]	RCT	1998	USA	F	80 (8); ?	1786	educ & vchr & mfar(w/med+slfm);<br>educ & mfar(w/med+slfm);<br>vchr	ac	NC
Messens 2014[575,844,845]	RCT	2011	EEE	P,F	≥65	208	aids & cgn & comm & mntr-mfa-	ac	NC
Metzelthin 2013[576,652,846-851]	cRCT	2009	NLD	F	77 (5); ≥70	m:12; 346	educ & mfar(w/med+slfm)	ac	NC
Moll van Charante 2016[577,852-862]	cRCT	2006	NLD	all	75 (3); ≥70	m:116; 3526	educ & mfar(w/slfm)	ac	NC

Study	Design	Enrolment began	Country	Population frailty	Population age, years	enrolled	Interventions	Controls	Funding
Monteserin Nadal 2008[578,863]	RCT	2004	ESP	all	80; 75–94	620	educ & rsk-mfa-	ac	NC
Morey 2006[579,864,865]	RCT	< 2006	USA	all	78 (5); 70–94	179	exrc; exrc	exrc	NC
Morey 2009[580,866-869]	RCT	2004	USA	U	78 (5); ≥70	400	exrc	ac	NC
Morgan 2019[581,870-872]	RCT	2014	GBR	P	65.3–88.1	51	exrc	ac	NC
Newbury 2001[582,873]	RCT	1998	AUS	U	80 (4); 75–91	100	mfa-(w/med)	ac	NC
Newcomer 2004[583,874,875]	RCT	2001	USA	U	≥65*	3079	educ & mfar(w/med)	ac	Mx
Ng 2015[584,876,877]	RCT	2009	SGP	P,F	70 (5); ≥65	246	cgn & ntr & exrc	ac	NC
Parsons J 2012[585,878,879]	cRCT	2007	NZL	P,F	78 (7); ≥55*	m:?; 205	hmcr & mfar(w/slfm)	hmcr & mfa-	NC
Parsons M 2017[586,880-883]	RCT	2003	NZL	F	83 (7); ≥55*	113	hmcr & ADL & mfar(w/slfm)	hmcr & mfa-	NC
Parsons M 2012[587,880-883]	cRCT	2003	NZL	F	81 (7); ≥55*	m:55; 351	hmcr & mfar	hmcr & mfa-	NC
Pathy 1992[588]	RCT	< 2006	GBR	all	73 (6); ≥65	725	rsk-mfa-	ac	NC
Phelan 2007[589]	cRCT	2002	USA	all	82 (5); ≥75	m:31; 874	mfar(w/med+slfm)	ac	NC
Ploeg 2010[590,884]	RCT	2004	CAN	P,F	81 (4); ≥75	719	educ & mfar(w/med)	ac	NC
Profener 2016[591,885-887]	RCT	2007	DEU	F	67–100	553	educ & mfar	ac	NC
Rockwood 2000[592,888]	RCT	< 2006	CAN	F	82 (7); ?	182	mfa-(w/med)	ac	NC
Romera-Liebana 2018[593,889,890]	RCT	2013	ESP	P,F	77 (7); ≥65	352	cgn & med & ntr & exrc	ac	NC
Rooijackers 2021[594,891-895]	cRCT	2017	NLD	F	82 (7); ≥65	m:10; 264	hmcr & ADL & mfar(w/slfm)	hmcr	NC
Rubenstein 2007[595]	RCT	< 2006	USA	F	74 (6); ≥65	792	mfar(w/med)	ac	NC
Ryvicker 2011[596,896]	cRCT	2005	USA	U	76 (13); ?	m:45; 3290	hmcr & mfar	hmcr & mfar	NC
Serra-Prat 2017[597,897]	RCT	2013	ESP	P	78 (5); ≥70	172	ntr & exrc	ac	NC
Shapiro 2002[598]	RCT	1998	USA	F	77; ?	108	hmcr & mfar	ac	NC
Sherman 2016[599,898]	cRCT	2006	SWE	all	≥75	m:16; 583	mfa-(w/med)	ac	NC
Siemonsma 2018[600,899,900]	RCT	2009	NLD	F	~84 [80-88]; ≥75	155	ADL	mfa-	NC
Stewart 2005[601,901,902]	RCT	2000	GBR	P,F	81 (7); ≥65	321	mfa-	mfa-	NC
Stuck 1995[602,903-907]	RCT	1988	USA	all	81 (4); ≥75	414	educ & mfar(w/med)	ac	NC
Stuck 2000[603,908-911]	RCT	1993	CHE	all	82 (5); ≥75	791	mfar(w/med)	ac	NC
Stuck 2015[604,766-769,912]	RCT	2000	CHE	R,P	75 (6); ≥65	2284	educ & mfar(w/med+slfm)	ac	NC
Suijker 2016[605,913-918]	cRCT	2010	NLD	F	≥70	m:24; 2283	mfar(w/med)	ac	NC
Szanton 2011[606,919,920]	RCT	2010	USA	P,F	78 (8); ≥65	40	ADL & aids & educ & exrc & mfar(w/med+slfm)	ac	NC
Szanton 2019[607,919,921-930]	RCT	2012	USA	P,F	76 (8); ≥65	300	ADL & aids & educ & exrc & mfar(w/med+slfm)	ac	NC
Takahashi 2012[608,931-937]	RCT	2009	USA	F	80 (8); ≥60	205	mntr-mfa-	ac	Mx
Teut 2013[609,938]	cRCT	2009	DEU	F	79 (11); ?	m:8; 58	hmcr & hmnt & exrc	hmcr	Mx
Thiel 2019[610,939,940]	RCT	2017	DEU	F	≥65	?	exrc & mfar(w/med)	ac	NC
Thomas 2007[611]	RCT	2001	CAN	P,F	81 (4); ≥75	520	mfar(w/med); mfar(w/med)	ac	?
Tomita 2007[612]	RCT	< 2006	USA	F	74 (5); ≥60	124	aids	ac	NC
Tulloch 1979[613]	RCT	1972	GBR	all	≥70	339	mfar(w/med)	ac	?
Tuntland 2015[614,941-943]	RCT	2012	NOR	U	79 (10); ≥18	61	hmcr & ADL & aids & mfa-(w/slfm)	hmcr & mfa-	NC
van der Pols-Vijlbrief 2017[615,944]	RCT	2013	NLD	F	83 (8); ≥65	155	hmcr & ntr & mfar	hmcr	NC
van Dongen 2020[616,945-948]	RCT	2016	NLD	all	75 (6); ≥65	168	ntr & exrc	ac	Mx
van Heuvelen 2005[617,949]	RCT	2001	NLD	P,F	≥65	233	exrc & psyc	ac	NC

Study	Design	Enrolment began	Country	Population frailty	Population age, years	enrolled	Interventions	Controls	Funding
van Hout 2010[618,950,951]	RCT	2002	NLD	F	81 (4); ≥75	658	mfar(w/med)	ac	NC
van Leeuwen 2015[619,952-956]	cRCT	2010	NLD	F	81 (8); ≥65	m:35; 1147	mfar(w/med+slfm)	ac	NC
van Lieshout 2018[620,957]	RCT	2011	NLD	P,F	74 (7); ≥65	710	ADL & med & ntr & sst	ac	NC
van Rossum 1993[621,958,959]	RCT	1988	NLD	all	≥75	580	mfar	ac	NC
Vass 2005[622,960-980]	cRCT	1999	DNK	all	≥75	m:34; 4060	mfar(w/med)	mfar	NC
Vetter 1984[623]	RCT	1980	GBR	all	≥70	1148	mfar	ac	NC
von Bonsdorff 2008[624,981-986]	RCT	2003	FIN	R	78 (2); ≥75	632	exrc	ac	NC
Wallace 1998[625,814]	RCT	< 2006	USA	all	72 (5); ≥65	100	exrc & mfar	ac	NC
Walters 2017[626,987,988]	RCT	2015	GBR	P	80 (7); 67–91	51	mfar(w/slfm)	ac	NC
Whitehead 2016[627,989,990]	RCT	2014	GBR	F	82 (11); ≥18	30	hmcr & ADL & aids & mfa-	hmcr & mfa-	NC
Williams 1992[628,991]	RCT	< 2006	GBR	all	≥75	470	mfar	mfa-	NC
Wolter 2013[629,992-994]	cRCT	2007	DEU	F	79; ?	m:69; 920	hmcr & mfar(w/med)	hmcr	NC
Wong 2019[630,995-998]	RCT	2016	HKG	all	78 (8); 60–105	540	mfar(w/slfm)	ac	NC
Yamada 2003[631]	RCT	1999	JPN	P,F	79 (7); ≥65	368	mfar(w/med)	ac	NC

Note that some reports provide information about multiple studies and are therefore cited more than once.

RCT: Randomised Controlled Trial; cRCT: cluster RCT.

Countries, territories, or areas of geographical interest, are indicated with ISO 3166-1 alpha-3 codes, except EEE to indicate a study in multiple European countries: AUS, Australia; BEL, Belgium; CAN, Canada; CHE, Switzerland; CHN, China; DEU, Germany; DNK, Denmark; ESP, Spain; FIN, Finland; FRA, France; GBR, United Kingdom; HKG, Hong Kong; ITA, Italy; JPN, Japan; MYS, Malaysia; NLD, Netherlands; NOR, Norway; NZL, New Zealand; SGP, Singapore; SWE, Sweden; THA, Thailand; USA, United States of America.

all: All frailty groups; R: Robust; P: Pre-frail; F: Frail, U, Unclassified.

Population age in years is provided as mean (SD), or alternatively ~median [IQR]; range, or minimum age, or percentage within age categories around 65 years based on available data.

* indicates that the minimum age criteria differed in conjunction with ethnicity,[556,585-587] medical condition,[583] both,[570] or living alone.[506]

m indicates the number of clusters assigned for cluster RCTs.

Intervention and control group abbreviations are a combination of the following:- ac: available care; ADL: activities of daily living training; aids: provision of aids and adaptions; cgn: cognitive training; comm: technology for communication and engagement; educ: health education; eng: engagement in meaningful activities; exrc: physical exercise; hmcr: formal homecare; hmnt: alternative medicine; med: medication-review; mfa: multifactorial-action; mfar: multifactorial-action and follow-on routine review; mntr-mfa: monitoring, which may trigger multifactorial-action; ntr: nutritional support; psyc: psychological therapy; rsk-mfa: risk screening, which may trigger multifactorial-action; sst: social skills training; vchr: care voucher provision; wlfr: welfare rights advice; w/med: with medication-review; w/slfm: with self-management.

Funding:- C: Commercial; Mx: Mixed; NC: Non-Commercial; ? Unclear

Appendix 8. Risk of bias in results of interest

We assessed risk of bias in 860 results of interest across 113 studies using version 2 of the Cochrane risk-of-bias tool for randomised trials (RoB2) [999-1002]. Thirty-four of the assessed results were unsuitable for inclusion in analyses and therefore we detail risk of bias in 826 results of interest here. The data associated with these assessments are available as supplementary data from https://doi.org/10.5518/1386 and the relevant data for each analysis is summarised in appendix 11.

Although we assessed risk of bias for each result of interest, we assessed the allocation domain per study and the judgments were the same across results per study in the 'deviations from the intended intervention' domain for all but four studies (described below). Therefore, we have summarised the risk of bias in these domains here on a per-study basis (see Figure 1). Risks of bias arising in the other domains varied according to the result assessed.

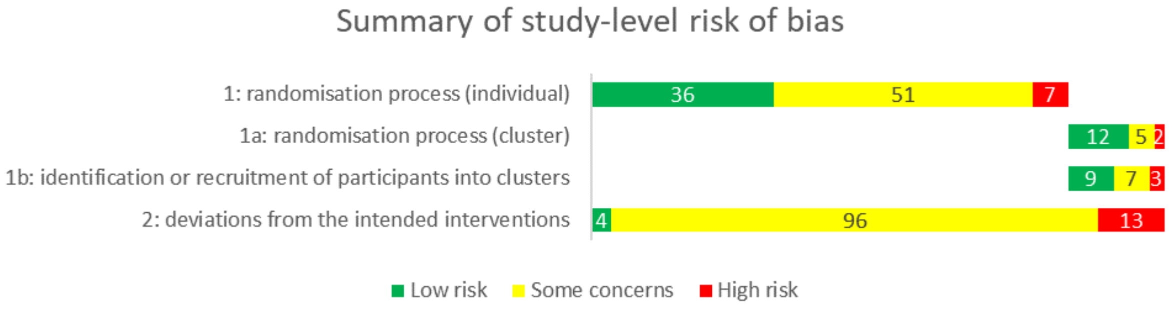

Figure 1 - Summary of study-level risk of bias for the domains related to allocation and deviations from the intended intervention in the 113 studies with results of interest. Four studies had results at differing risk of bias in domain 2 so we have used the highest risk in this figure.

8.1 Study-level risk of bias for individually-randomised studies

8.1.1 Risk of bias due to the randomisation process (individual)

Among individually-randomised studies, we judged the randomisation process to present a low risk of bias for 36 studies, some concerns for 51 studies and a high risk in seven studies. Of those at high risk of bias, we were concerned that allocation was predictable in three due to small-block randomisation [503,519,630], the process was reported to have been subverted in two [506,565], there was unexplained imbalance in baseline characteristics in one [612] and participants were allocated prior to recruitment in one [535].

8.1.2 Risk of bias due to deviations from the intended interventions

We judged the risk of bias due to deviations from the intended interventions to present some concerns in 80 individually-randomised studies. Two studies were judged to have low risk of bias because they had investigated whether the control group had deviated from their assigned intervention due to the trial context. Twelve studies presented a high risk of bias in this domain for at least one result: six due to post-randomisation exclusions [503,546,564,574,598,617], four due to risk or detection of contamination [507,544,566,604], one due to a combined risk of contamination and the reassignment of intervention participants to control following non-engagement [565], and one due to substantial modifications in the intervention from what was originally intended [506].

Four of the studies with some results in this domain at high risk of bias due to post-randomisation exclusions also reported results for other outcomes that we judged some concerns because participants were not excluded, we could incorporate those excluded in the analysis, or the number of exclusions was too small to substantially affect the results [546,564,574,598].

8.2 Study-level risk of bias for cluster-randomised studies

8.2.1 Risk of bias due to the randomisation process (cluster)

We judged the randomisation process in cluster-randomised trials to present low risk of bias in 12 studies, some concerns in five studies and high risk of bias in two studies. We judged that there was a high risk of bias in the randomisation process of Bleijenberg 2016 [509] because, despite reportedly being computer-randomised from a complete list stratified by cluster size, there was a substantial and unexplained imbalance in cluster size, education level and socioeconomic status. We reached the same judgment for Blom 2016 [510] where the cluster-randomisation process and an additional individual randomisation within the intervention arm were not detailed and there was also substantial and unexplained imbalance in cluster size.

8.2.2 Risk of bias due to identification or recruitment of participants into clusters

We judged the identification or recruitment of participants into clusters to present low risk of bias in nine studies, some concerns in seven studies and high risk of bias in three studies. In the three studies at high risk of bias, participant recruitment took place after cluster allocation; in two studies the recruiters and participants appeared to know the allocation prior to recruitment [548,589], while in Parsons J 2012 [585] this was unclear and we were concerned by imbalances in participant characteristics.

8.2.3 Risk of bias due to deviations from the intended interventions

We judged the risk of bias due to deviations from the intended interventions to present low risk of bias in two studies, some concerns in 16 studies and high risk of bias in one study. In the studies at low risk of bias it seemed unlikely that there were

deviations due to the trial context despite awareness of the intervention [548,570]. Sherman 2016 [599] was at high risk in this domain because participants who did not receive the intervention were excluded from the analysis.

8.3 Risk of bias in results of interest

Risks of bias arising in the other domains varied according to the result being assessed. These are summarised in Figure 2. For missing outcome data, differences particularly related to whether the outcome was continuous or dichotomous and the proportion of people experiencing the event, with rare, dichotomous outcomes more likely to be at higher risk. For bias in measurement of the outcome, differences largely related to whether the outcome was self-reported or sourced from records and whether the self-reporting was about the individual's perception (such as depressive symptoms) or memorable, observable events (such as hospitalisation). For bias in selection of the reported result, we very rarely had access to a sufficiently detailed analysis plan, so differences largely related to whether we had access to numbers of events and cases where there were no plausible alternative definitions for the measure such that the same data could not be recut into different groups, such as mortality as a defined outcome.

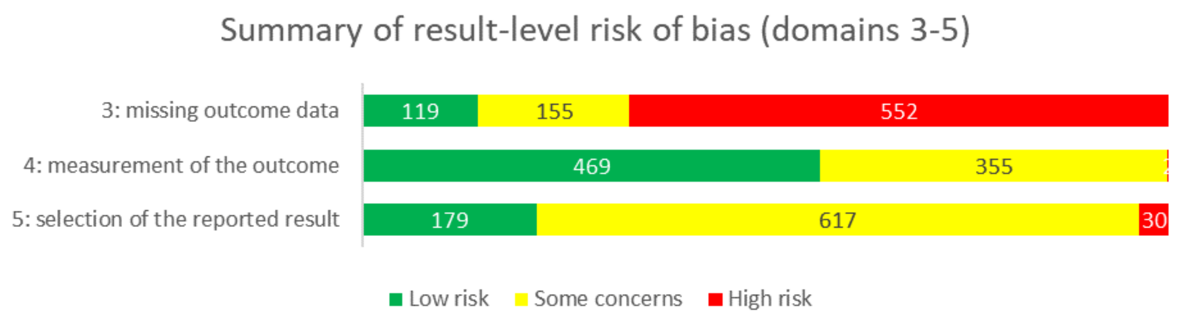

Figure 2 - Summary of result-level risk of bias for the domains related to missing outcome data, measurement of the outcome and selection of the reported result in the 826 results of interest suitable for analyses.

8.4 Overall risk of bias

Overall, no results of interest were judged to have low risk of bias, there were some concerns about 28%, with the remaining 72% at high risk of bias. We further judged those results at high risk of bias to present either serious concerns (53% of results) or very serious concerns (19% of results). Because there were differences in risk of bias by outcome, some outcomes were reported by more studies, and different studies report different outcomes, we have not presented a more detailed breakdown here. Results were included in analyses regardless of risk of bias, but we conducted sensitivity analyses excluding results for which we had very serious concerns. We judged whether to downgrade our certainty in the evidence for an effect estimate based on the contributions of results judged as serious concerns or very serious concerns about risk of bias.

Appendix 9. Description of the components and aspects of components used to determine intervention groups, organised by topic

Topic	Brief name (abbreviation)	Public-facing name	Plain language description
Activities	ADL (ADL)	Practise **day-to-day activities**	The person is offered support to practise carrying out day-to-day activities, for example dressing or taking the bus. The person may also be offered recommendations on how to carry out day-to-day activities safely or better. For example, this may include using appropriate footwear, removing loose rugs, cords, and clutter in walking paths or improvement of lighting. The person may receive an assessment to create a tailored day-to-day activities plan.
Activities	Aids (aids)	Get **equipment and technology** to support day-to-day activities	The person is offered equipment or technology to aid in day-to-day activities. This may include ramps, walking frames, grab rails, or a system of sensors that turn on the lights when the person gets up from the bed, for example. The person may receive an assessment to choose specific equipment or technology.
Activities	Meaningful activities (eng)	Identify and engage in **meaningful activities**	The person is offered support to identify and participate in activities that they find meaningful. Examples may include leisure activities, crafts, volunteering, but the focus is on the activities being ones that the person finds meaningful. The activities may be organized for the person, be done by the person alone, or be community activities that were already in place, for example.
Brain training	Cognitive training (cgn)	Do **brain training**	The person is offered training in thinking tasks such as memorising, paying attention or planning, among others. The training includes practical exercises and information about strategies to help thinking tasks.
Diet/nutrition	Nutrition (ntr)	Get **dietary advice and support**	The person is offered recommendations about diet and/or food supplements in group sessions or one-to-one. This is different from receiving information about nutrition as part of "Find out more information about health" because there is a greater focus on providing specialized nutrition/dietary advice and related activities. For example, the person may also participate in writing a food diary, cooking certain types of meals, and weight monitoring. They may be provided with particular foods or supplements. The person may receive an assessment to create a tailored nutrition plan.
Financial support	Care voucher (vchr)	Get a health and care **voucher**	The person is offered a voucher to pay for health and personal care services and support on how to use the voucher.
Financial support	Welfare (wlfr)	Get advice about **welfare services** with follow-up	The person is offered tailored advice about the welfare services and benefits they can access. This is based on an assessment. Afterwards, the person is offered support in putting the plan in practice and accessing the services and benefits they are entitled to.
General health information	Education (educ)	Find out more **information about health**	The person is offered information about a set of health topics. The topics may include many areas, for example, oral health, nutrition, physical activity. The information may also focus on areas that are more important for the person. The way the information is provided is more structured than the particular advice someone may receive as part of a clinical consultation with a health professional. The person may be offered information in group sessions or on one-to-one contact.

Topic	Brief name (abbreviation)	Public-facing name	Plain language description
Homecare	Homecare (hmcr)	Receive formal **home care**	The person is offered support services at home by health or care professionals. The services include, for example, nursing care or support with household tasks.
Individualised care	Medication review (med)	**Optimise my medication**	The person is offered recommendations to change medication. For example, someone may be on too many medicines and be recommended to stop some. The changes to the medication can be provided on their own or as part of a more complete assessment and recommendations (see “Take part in individualised care planning based on an assessment” for more details).
Individualised care	Monitoring (mntr)	Get care planning from health **monitoring** (including providing equipment)	If a health need is identified from monitoring, the person is offered an individualised care plan (see “Take part in **i**ndividualised care planning” for more details). To check for needs, the person participates in screening and monitoring of their bodily function, for example blood pressure, and heart rate. This happens at least weekly. The person is offered equipment to record their bodily function.
Individualised care	Multifactorial action (mfa)	Take part in **individualised care planning**	The person is offered an individualised care plan that includes recommendations for future action. The care plan is based on an assessment of the person’s needs and preferences and may include a variety of actions (related with physical exercise, diet, mood, etc.). The assessment structure may be set in advance or guided by the experience of a clinician. The person may receive support to carry out actions, for example, with referrals to certain services. The person may also receive support from a care coordinator, who helps to deal with different services and/or professionals.
Individualised care	Review [in relation to multifactorial action] (mfar)	Have **regular follow ups** [after individualised care planning]	The person is regularly followed up after receiving an individualised care plan based on an assessment. The follow up may include encouraging the person to carry out previous recommendations. The person may also be offered a new assessment of their needs and other relevant changes, and an updated individualised care plan.
Individualised care	Risk screening (rsk)	Get care planning following **screening** for possible health problems	A tool to indicate possible health problems is used routinely and, if indicated, the person is offered an individualised care plan (see “Take part in individualised care planning” for more details). The tool and the results that indicate problems are standardised, such as a questionnaire score or analysis of electronic health records.
Individualised care	Self-management [in multidomain assessment and care planning] (slfm)	Do activities to motivate taking good care of myself [when taking part in individualised care planning]	The person is engaged in conversations or activities designed to motivate them to care for themselves. The person may also be offered guided practice in some techniques, for example, to help them set up personal goals and solve problems.
Alternative medicine	Alternative medicine (hmnt)	Get alternative medicine	The person is offered alternative medicine such as homeopathic or naturopathic consultation and treatment.

Topic	Brief name (abbreviation)	Public-facing name	Plain language description
Physical exercise	Exercise (exrc)	Do physical **exercise**	The person is offered support to carry out physical exercise. The exercise may be on their own or in training sessions. This is different from receiving information about physical activity as part of “Find out more information about health” because there is a greater focus on providing specialized physical exercise advice and related activities. Physical exercises are activities done by a person to build up or maintain physical fitness (such as strength, balance, among others). The person may also receive an assessment to create a tailored exercise plan.
Social communication	Social skills (sst)	Practise **social interaction**	The person is offered information and support to improve their ability to relate with other people. This may include practising or discussing different ways of communicating.
Social communication	Telecoms (comm)	Get **technology for communication and engagement**	The person is offered technology to enable communication with friends, family, neighbours, or the community. For example, a mobile phone, or tablet, as well as applications such as email or social media. The person will usually receive support in using the applications.
Wellbeing	Psychology (psyc)	Get **wellbeing advice and support**	The person is offered support for their wellbeing in areas like feeling low and dealing with worries. The support includes information about how we usually think and feel, and information and activities to deal with what we think and feel, such as noticing and learning how to overcome unhelpful thoughts.

Appendix 10. TIDieR descriptions of intervention groups with more than one intervention

Group: ADL

There are two interventions in this group: Dorresteijn 2016[526], Siemonsma 2018[600]

TIDieR item	Description
**1. Brief name**	
**2. Why**	Goal: Both interventions had a focus on encouraging and enhancing independent living for older people. One was focused upon those who had a fear of falls which reduced and restricted their activity levels. This intervention also aimed to reduce burden on the healthcare services. The other intervention was focused upon increasing physical activity to prevent decline in a sustainable way.<br><br>Rationale: One intervention is based upon previous programme effectiveness. Both interventions have grounding in cognitive theories related to self-efficacy and control. The sustainability of the intervention was rationalised as likely due to embedding exercises within routine activity in one report. The other saw provision at home as beneficial to sustainability.
**3. What (materials)**	One intervention is vague in describing intervention material referring only to training materials for the providers of the intervention. The other intervention listed DVD's with case studies of challenges and solutions, printed materials including educational leaflets, checklists and worksheets, action planning documentation, standardised assessments and an evaluation questionnaire for participants.
**4. What (procedures)**	The descriptions of the processes for carrying out the interventions were varied. Both interventions mention an aspect of cognitive restructuring, motivational interviewing or confidence building. Training was provided in both interventions in a targeted and supervised way. One mentions how this training could be monitored and adapted over time and was to focus on daily tasks. One is focused on reducing fear of falling. One intervention mentions the input of caregivers. One also mentions accessibility to usual care by a multidisciplinary team.
**5. Who provided**	Both interventions were delivered by healthcare professionals with specialised intervention training. One was delivered by community/geriatric nurses. The other by physiotherapists. Usual care was provided by relevant professionals.
**6. How**	Provision was face-to-face and to individuals or with a significant other present. One intervention included input over the telephone; the other describes home based contact only.
**6b. How organised**	One report does not mention intervention organisation, the other places organisation on the facilitator and the participants' significant other to undertake activities.
**7. Where**	Both interventions were implemented in The Netherlands, and in the participants' home.
**8. When and how much**	Eligibility for the interventions varied. One intervention was accessible on referral. The other was available to people over 70 living in their own homes, identified by a postal screening questionnaire as having a fear of falling and fair to poor self-perceived health with a level of frailty.<br><br>The nature, duration and frequency of delivery varied. One intervention comprised seven sessions, three of which were face-to-face around 60-75 minutes in duration and four of which were over the telephone about 35 minutes in duration. The other intervention was delivered over a maximum of 18 sessions. One intervention duration was ten weeks, the other three months.
**9. Tailoring**	Both interventions were tailored to the needs, abilities and preferences of the individuals. One intervention aimed to provide tailored training on a feared activity of the participants' choice. The other intervention was tailored to the participants' home environment and was monitored and adapted throughout the programme.
**10. Modifications**	Neither report described modifications to the intervention.

**11. How well (planned)**	One report does not mention any plans for adherence or fidelity assessment. The other report conducted an evaluation of acceptability and feasibility by the participant. They also aimed to collect information on adherence to the intervention protocol, the time spent on delivery of the intervention and identify any barriers to implementation.
**12. How well (actual)**	One report did not undertake adherence or fidelity assessment. The other found that the intervention was perceived as feasible and acceptable by deliverers and participants. The intervention protocol was broadly adhered to. Action planning decreased over the duration of the intervention from over 70% to just above 50%. It was noted that training on a feared activity was problematic as this feared activity was often hard to identify.

Group: ADL, aids, education, exercise, multifactorial-action and review with medication review and self-management

There are two interventions in this group: Szanton 2011[606], Szanton 2019[607]

TIDieR item	Description
**1. Brief name**	
**2. Why**	Goal: Both interventions were targeted at both intrinsic (personal) and extrinsic (environmental) factors which contribute to disability in older people. In addition both interventions were targeted at those who were living on a low income. Both interventions had a focus on function, either by improvement in function or reducing functional difficulties. One intervention also mentioned reducing disability and the use of person centred goal setting to improve overall health, wellbeing and quality of life.<br><br>Rationale: Both reports mention the value of person centred approaches to care provision. One intervention refers to the need to address multiple factors which contribute to the decline of older people with a multi-component intervention, with consideration of the idea that such factors often interact to increase the impact on disability. This intervention also noted the need to reduce healthcare costs. The other intervention attributed the higher level of disability in lower income adults to a range of factors including environmental ones. This intervention was theoretically grounded and based on the success of piloting work.
**3. What (materials)**	Both interventions used a similar material base. A client/clinical assessment protocol, home modifications and assistive devices, letters and/or referrals from nurses to primary care providers. Additionally a DVD of Tai Chi exercises was provided in both interventions. One intervention also mentioned a health passport. The other intervention included the provision of a medication calendar and a Community Aging in Place - Advancing Better Living for Elders (CAPABLE) notebook to participants. In addition to the above one intervention described training materials for the providers, including a manual, audio tapes to record the sessions, a checklist to review the sessions and reminders regarding upcoming sessions.
**4. What (procedures)**	Both interventions included a multi-domain assessment with subsequent planning and arrangement of care based upon this. This assessment focused upon a range of domains including function, depression, pain, strength, medication and environmental factors among others. Physical exercise training and health related education provision on various topics including medication management, falls risk and self-management strategies were part of both interventions. The provision, fitting and relevant training on the use of adaptations to the environment was part of both interventions. Both interventions included access to relevant additional support such as Tai Chi training and mental health support for depression. Both interventions included routine reviewing and refinement of planning as well as access to usual care. One intervention also described the training and supervision of providers.
**5. Who provided**	Both interventions were delivered by nurses and occupational therapists. The adaptations were made by handymen. One intervention described input from a primary care provider and the other from relevant professionals of a multidisciplinary team as required.
**6. How**	The intervention was provided at home to individuals face-to-face. One intervention mentioned the collaborative development of care planning between providers and the inclusion of motivational interviewing to participants.

**6b. How organised**	In both interventions the care plans were designed to be delivered by a multidisciplinary team; occupational therapists and home modification co-ordinators organised and facilitated the housing adaptations. Both interventions involved planning documentation and appropriate letters and referrals to be sent by nurses. One intervention mentioned the staggering of intervention delivery to give participants time to engage with components. The other intervention used a secure share-site for the ease of sharing documentation across providers.
**7. Where**	Both interventions were undertaken in the USA, and were delivered at home.
**8. When and how much**	Both interventions targeted those who were from low income circumstances, with at least one limitation to activities of daily living and two limitations to instrumental activities of daily living. One intervention was location specific and participants were recruited from a waiting list for home based services. The participants were contacted by post.<br><br>The nature, duration and frequency of intervention delivery was similar across interventions, both involving around six visits from occupational therapists, four visits from nurses, each of around 60 to 90 minutes in duration. One intervention lasted six months, the other four months. Both interventions describe home visits to provide adaptations over as many visits as required.
**9. Tailoring**	Both interventions were tailored to the participant's goals, preferences, and risk level. This included the number and nature of visits as well as the development of strategies. One intervention described the tailoring of the training, the adaptations at home and the behavioural plan. The other intervention included a medical alert if polypharmacy was a significant concern.
**10. Modifications**	No modifications were mentioned in either report.
**11. How well (planned)**	One report did not mention the intention to measure fidelity or evaluate the intervention. The other included staff training, reminders for participants, supervised learning of exercises and the supervision of providers to improve adherence.
**12. How well (actual)**	One report did not mention report on fidelity or evaluate the intervention effectiveness. The other intervention noted that 92.8% of participants received at least eight sessions, less than 4% received less than three sessions, which had been defined as a minimum threshold for treatment. There was a mean of 9.1 visits per participant.

Group: Aids

There are two interventions in this group: Borrows 2013[511], Tomita 2007[612]

TIDieR item	Description
**1. Brief name**	
**2. Why**	Goal: One intervention had one clear goal, to reduce disability; whilst the other goal and rationale was focused upon decreasing dependence to sustain living at home, and to enable informed decision making by older people on equipment and products to maintain living at home.<br><br>Rationale: One report did not distinguish between the goal and rationale; however the implication was that independent living centres provide an opportunity to support informed decision making and safe use of aids and adaptations to maintain living at home. The other intervention was based on previous studies showing the benefit of assistive technology in sustaining living at home, additionally the technology of choice was based upon evidence due to ease of installation and use.
**3. What (materials)**	A range of materials were provided to participants in these interventions. One intervention was focused upon assistive technology, providing X10 Active Home kits including the necessary software, other standalone products, activity monitoring software and a computer and internet access as required. The other intervention was orientated to physical supportive equipment such as toileting and bathing equipment, medical equipment

	was also available on loan. Additionally this intervention provided information and advice on the safe use of equipment to maintain independence at home.
**4. What (procedures)**	Both interventions involved an assessment of needs; one specifies this as an assessment of both the individual and their home setting. One intervention included the installation of equipment, training on the safe use of this and ongoing support. The other intervention used the assessment to identify appropriate equipment which the participant was required to fit, although they could try out demonstration equipment at the independent living centre, which they received transportation to. Advice on other supportive service options was also identified during this assessment.
**5. Who provided**	One intervention was provided by an Occupational Therapist or nurse, with a geriatric nurse providing support. Equipment was fitted by a computer engineer. This report explicitly mentioned intervention specific training. The other intervention is provided by an Occupational Therapist assistant.
**6. How**	Both interventions were provided individually and face-to-face, however one was at home and provided additional support by telephone. The other intervention was provided in the independent living centre.
**6b. How organised**	Organisation was not always entirely clear in one intervention but stated that there was a cost limit of $400. The other intervention was organised by the British Red Cross.
**7. Where**	One intervention was undertaken in the USA and in the participant's home and the other in the independent living centre(s) in the UK.
**8. When and how much**	One intervention had clear eligibility criteria, participants had to be 60 years of age, living alone, have impairments to activities of daily living or instrumental activities of daily living, to be interested in technology and have no cognitive impairments. The other intervention simply mentioned access to be two weeks after randomisation.<br><br>The assessments for the intervention varied in length, one involved a 90 minute assessment, the other a 150 minute assessment. The technology intervention allowed for three to nine hours for engineer to install the equipment at the participant's home, with this intervention support was given as required.
**9. Tailoring**	Both reports describe the interventions being tailored to the needs, preferences, and also the safe capacity of the participant. One intervention mentioned follow up support being as required, the other mentioned training on the equipment being tailored.
**10. Modifications**	Neither report mention modification to the intervention.
**11. How well (planned)**	One report did not mention work to assess fidelity or adherence. The other intervention mentioned that fidelity to the intervention was promoted. Additionally this study collected data on the type of technology which was provided, as well as problems encountered and solutions to those problems.
**12. How well (actual)**	One study did not report on fidelity or adherence. The other reported that 100% of participants received software, although there was variation in what support items they accessed. Two years later 65% of participants were still using one or multiple pieces of assistive technology. Lack of use of the equipment was usually related to a failure of the equipment, either meaning functional failure, the equipment not meeting the needs of the participants or the participant's inability to use it.

Group: Available care

There are 98 interventions in this group: Alegria 2019[503], Arthanat 2019[504], Balaban 1988[506], Barenfeld 2018[507], Bleijenberg 2016[509], Blom 2016[510], Botjes 2013[512], Bouman 2008[513], Brettschneider 2015[514], Cameron 2013[515], Carpenter 1990[516], Cesari 2014[517], two arms of Clark 1997[519], Clark 2012[520], Coleman 1999[521], Counsell 2007[522], Cutchin 2009[523], Dalby 2000[524], de Craen 2006[525], Dorresteijn 2016[526], Fabacher 1994[528], Fairhall 2015[529], Fischer 2009[532], Ford 1971[533], Gene Huguet 2018[536], Gill 2002[537], Giné-Garriga 2020[538], Gitlin 2006[539], Grimmer 2013[540], Gustafson 2021[541], Gustafsson 2013[542], Harari 2008[544], two arms of Hay 1998[546], Hebert 2001[547], Henderson 2005[548], Hendriksen 1984[549], Hogg 2009[550], Holland 2005[551], Howel 2019[552], Imhof 2012[553], Jitapunkul 1998[555], Kerse 2014[556], Kono 2004[559], Kukkonen-Harjula 2017[561], Lambotte 2018[562], Leung 2004[563], Leveille 1998[564], Liddle 1996[566], Liimatta 2019[567], Loh 2015[568], Lood 2015[569], Mann J 2021[570], Melis 2008[573], Meng 2005[574], Messens 2014[575], Metzelthin 2013[576], Moll van Charante 2016[577], Monteserin Nadal 2008[578], Morey 2009[580], Morgan 2019[581], Newbury 2001[582],

Newcomer 2004[583], Ng 2015[584], Pathy 1992[588], Phelan 2007[589], Ploeg 2010[590], Profener 2016[591], Rockwood 2000[592], Romera-Liebana 2018[593], Rubenstein 2007[595], Serra-Prat 2017[597], Shapiro 2002[598], Sherman 2016[599], Stuck 1995[602], Stuck 2000[603], Stuck 2015[604], Suijker 2016[605], Szanton 2011[606], Szanton 2019[607], Takahashi 2012[608], Thiel 2019[610], Thomas 2007[611], Tomita 2007[612], Tulloch 1979[613], van Dongen 2020[616], van Heuvelen 2005[617], van Hout 2010[618], van Leeuwen 2015[619], van Lieshout 2018[620], van Rossum 1993[621], Vetter 1984[623], von Bonsdorff 2008[624], Wallace 1998[625], Walters 2017[626], Wong 2019[630], Yamada 2003[631]

TIDieR item	Description
**1. Brief name**	
**2. Why**	Around 22 reports included some rationalisation and goals in their description of the intervention. Four referred to an ageing or frail population living with unmet needs or in some stage of functional decline. Three others describe current care, including the need to limit costs, provide quality care and to compare standard primary care with the specialist care of geriatricians. Two reports indicated a need to promote independence in the older population. Five reports mentioned standardised care, whilst eight described access to actions that were not intended or anticipated to affect an individual's independence such as attention control, placebo, and assessments or social interaction.
**3. What (materials)**	Twenty-seven reports make some mention of materials required. Ten studies used various assessments, some of which were standardised. Eight described written materials provided to participants, a further study provided intervention materials to control participants at the end of the research process and another provided participants with placebo nutritional supplements. Four reports mentioned access to usual care equipment and services. At least ten reports described the sharing of information gleaned during assessment with other healthcare professionals through referrals etc. for ethical purposes. Materials for provider training and the assessment of fidelity were also mentioned in a small number of reports.
**4. What (procedures)**	A large majority of reports had some description of the procedure for the intervention. In 82 cases this included reference to usual available care, which a participant would access of their own accord. Ten reports described the assessment of participants, six mentioned social contact with the research team and referred to this as increased attention. Seven studies explained that identification of emergency needs required this information to be shared with other professionals as an ethical or moral obligation. Some studies provided non-active components to participants, in one case this was a placebo nutritional supplement, in five others this was written materials and eight interventions included peer contact, such as workshops or educational lectures.
**5. Who provided**	Almost half, around 43 reports, did not mention the providers. However, thirty-nine did refer to the provision of usual care by the expected professionals, while 15 mentioned the participants own GP or physician. It was not always clear if this was related to an aspect of the intervention which would be beyond usual care or not. Five reports explicitly referred to input from the research team. Additional providers mentioned were nurses, social workers, occupational therapists, non-trained or non-medical personnel, health educators and students, these last were usually when an intervention involved some non-active components such as placebo social interaction.
**6. How**	Sixty-four reports did not describe how 'available care' was delivered. At least thirteen of the remaining number referred to usual care being provided in the most appropriate way, for example in clinics and at home, through distanced or face-to-face methods. Some reports are less clear though a small percentage have face-to-face and individual contact for assessments, three used the postal service to provide information or collect assessments from participants, five conducted telephone calls and three had workshop or group sessions as part of a placebo, non-active component of the control.
**6b. How organised**	The majority, over 60, reports did not describe organisation. However around one third made some reference to organisation for funding. This was usually the nationally recognised approach to care funding in which the study was practising, be that state funded care or through varied insurance plans. GPs and primary care physicians were mentioned as involved in organisation in at least 14 reports. This was often in a gatekeeper role, recommending care and referring on to other services. Two reports mentioned explicit input in organisation of study including a nurse and a research assistant.
**7. Where**	All reports gave some indication of the location in which the studies were undertaken, though the country of one of these was unclear. Ninety-four studies were carried out in one country alone, whilst two were multi-site studies in four different countries. One study was carried out in Denmark, Northern Ireland, Germany and Spain: the other in Belgium, Spain, Ireland and Italy. Of the remaining studies the majority were also European, including 16 in The Netherlands, eight in the UK, four each in Germany, Spain and Finland. Three

	were carried out in Sweden, three in Switzerland and one each in France, Denmark and Belgium. A significant amount were also Northern American, including 26 in the USA and eight in Canada. Seven were caried out in Australia and one in New Zealand. Seven were undertaken in Asian countries, two in Japan, two in Hong Kong, one each in Thailand, Malaysia and Singapore.
**8. When and how much**	Most of the studies had set inclusion and exclusion criteria, although ten were not described at all. Fifty-nine studies involved those with identified specific needs, be that level of frailty, a diagnosis of a specific chronic condition or limitations to activities of daily living. Another common inclusion criterion was a minimum age limit. Fifty-three studies used age limits, usually just a minimum age, the lowest being 50 years and over, the highest being 85 years and over. Other common inclusion criteria were involvement in a service, which was mentioned in 26 reports, specific socio-economic factors, mentioned in at least nine reports, recent hospital attendance and involvement in a research cohort. Multiple studies excluded participants based on cognition and end of life status.<br><br>Few reports mentioned frequency of input given the nature of available care, however five did mention the contact of those administering assessments and ten described to some extent the nature of non-active components such as social telephone calls or workshop sessions.
**9. Tailoring**	Only ten of the 97 reports detailed any tailoring. Five mentioned that tailoring would be enacted by the participant themselves in line with their own care needs. Three studies explicitly described processes to access emergency services should the need be identified through the research process. Four studies had non-active or control components which involved tailoring, such as a social activity tailored to the participants preferences.
**10. Modifications**	Only one report mentioned modifications which related to reformation of service provision during the project.
**11. How well (planned)**	Very few reports detailed any steps to ensure fidelity to the intervention. Three of these were related to the recording and supervision of contacts participants had with providers to ensure delivery was as per protocol. Two reports also mentioned training of providers to ensure experimental intervention components were not administered to control participants, and one conducted inter-rater-reliability evaluation between experimental and control group's receipt of the assessment. At least two reports described steps taken to limit control participant access to the components of the experimental intervention, a further report detailed that any cross-contamination was measured. Two reports detailed steps to ensure participant compliance with attention control or placebo.
**12. How well (actual)**	Very few reports detailed success of delivery. Two reports noted that a substantial proportion of participants allocated to available care accessed components of the experimental intervention privately, while two others noted that participants accessed at least some aspects of the experimental intervention by some means. One reported on high levels of attrition, one explained that some participants had been referred to care due to initial assessments revealing emergency need. Three studies with placebo or attention-control components found compliance with these to be reasonably good. Inter-rater- reliability was 0.79-0.94 for the study which measured assessments between control and experimental groups across providers. One study noted that the intervention was delivered as intended.

Group: Education

There are three interventions in this group: Barenfeld 2018[507], Gustafsson 2013[542], Lood 2015[569]

TIDieR item	Description
**1. Brief name**	
**2. Why**	Goal: All three interventions had very similar goals, focused around the prevention or delay of deterioration in health and quality of life of older people. Two were focused upon the prevention of frailty and morbidity, one of these was also to support ageing in place. Two interventions were aiming to reduce the consumption of care. Two interventions were also targeting minority groups with language barriers.<br><br>Rationale: Person-centred care approaches were the core rationale for two of the interventions, as was the premise that peer learning would prove beneficial. All three interventions were based upon previous research,

	including RCT's of group education. In one intervention it was put forth that a multidisciplinary team was well placed to provide health education and benefit the health outcomes of older people.
**3. What (materials)**	All three interventions provided health advice information in a written format. One also provided the information in audio format. Two interventions provided information in different languages as well as the native language. One intervention also mentioned documentation materials and referrals as required. One other intervention described how usual care needs were to be met with regards to equipment provision.
**4. What (procedures)**	All three interventions worked with a group session format, where health and social care professionals delivered a specific session. Sessions provided education and a forum for peer discussion which was relevant to participants and required their input and exchange of experiences. All interventions provided group sessions then a follow up of one individual session at home. All interventions enabled access to usual care, including home care and medical services. One intervention also described how providers were supported, and one other described the input of interpreters.
**5. Who provided**	All three interventions were provided by a multidisciplinary team including an Occupational Therapist, a Nurse, a Physiotherapist and a Social Worker. Usual care was provided by a range of staff as required. One intervention also required the input of supportive staff such as translators.
**6. How**	All three interventions were delivered face-to-face in group sessions of four to six participants and then with one session delivered individually.
**6b. How organised**	Organisation for the intervention was described in varying detail. All three describe the input of the four key professionals and the participant. One intervention described the training of providers and some of the auditing processes, this intervention and one other also mention the funding from the state and the other describes input from the university. One intervention stated the importance of provider continuity.
**7. Where**	All three interventions were undertaken in Sweden, individual sessions were delivered at the home of the participant, however the location of the group session delivery was unclear in the reports.
**8. When and how much**	All three interventions require participants to not have existing support to carry out activities of daily living. Two interventions have a minimum age requirement of 70 years and to be a migrant to Sweden. The other intervention required participants to be classified as prefrail. It appears that the interventions were location specific.<br><br>All three interventions were delivered through four weekly sessions, lasting between one and half and two hours. The follow up individual sessions were delivered about two to three weeks after the final group session.
**9. Tailoring**	All three interventions describe tailoring to the needs of the participant. In the group sessions this involved pertinent discussion for the group and follow up was tailored to the individual's needs. The two interventions aimed at supporting migrants could tailor language as required.
**10. Modifications**	None of the reports described modifications to the interventions.
**11. How well (planned)**	All three reports described steps taken to improve adherence and monitor fidelity. All three developed their intervention with input from stakeholders including representatives of the participant group. One intervention also described training for providers and a priori approval of deviations from protocol. One other intervention implicated the use of consistent providers for continuity. The other intervention improved attendance by goal setting at registration and predefining minimum participation levels of 50% of meetings to be attended by participants.
**12. How well (actual)**	Intervention attendance was monitored and reported for all interventions. One intervention had 73% attendance at 3 or more sessions, one other had 99% attendance at 3 or more sessions while the other had 100% attendance at 3 or more sessions.

Group: Education, exercise, multifactorial-action and review with medication review and self-management strategies

There are two interventions in this group: Faul 2009[530], Leveille 1998[564]

TIDieR item	Description
**1. Brief name**	
**2. Why**	Goal: To reduce the risk of frailty, disability and dependence by enhancing existing care models with the promotion of self-management strategies.<br><br>Rationale: Both interventions were theoretically driven and evidence based. Drawing upon previous work implicating the benefits of such programmes with older people of reducing risk of decline by empowering and informing older people.
**3. What (materials)**	Both interventions developed care planning based on an assessment of the participant and advice sheets were provided to the participant. One intervention provided a self-management workbook and referrals to services. The other used standardised assessments, accessed existing care notes and also provided an exercise software programme. This intervention also used scripting for their telephone contact.
**4. What (procedures)**	Both interventions required a comprehensive assessment, although the content of the data collected was only described for one, which focused on function, mobility, mental health, medication and the home environment. A tailored care plan was developed in both interventions, according to the needs and preferences of the participant. This included a tailored exercise plan. Both interventions provided information on health behaviour. One intervention also explicitly mentions referrals on to mental health and substance misuse services. Both interventions describe follow up input and telephone contact. One intervention used peer support mentoring, for which training was undertaken.
**5. Who provided**	One intervention was provided by an interdisciplinary team, led by a physical therapist and working with a physical therapist student and a social work student. The other was overseen by a geriatric nurse practitioner and required the input from health mentors, lay leaders, primary care physicians, dietitians and social workers as required.
**6. How**	Both interventions have individual provision, one also used group sessions. Provision was face-to-face, both interventions were conducted within the home and used telephone contact, one also appears to have been in senior centres.
**6b. How organised**	Organisation varied, one intervention was overseen by the interdisciplinary team, students were supervised, aims were to forge community links. Reports were shared with participant's primary care physicians. The other intervention depended upon a number of large health providers for the development and implementation of the intervention as well as access to participants.
**7. Where**	Both interventions were provided at home, one was also provided in a senior centre. Both interventions were run in the USA.
**8. When and how much**	Inclusion and exclusion criteria varied between interventions. One had a minimum age of 65, requirement for participants to have a permanent address, to be literate and have a primary care physician. The other intervention required referral based on one or more chronic condition. One study excluded those living in long-term care, those with acute needs or recent serious health events, or those in receipt of home care. The other study excluded those living with dementia or with terminal conditions.<br><br>The interventions varied in number of visits: one conducted three, while the other conducted between one and eight. The duration of visits was mentioned in one study as 1-2 hours. One study had a requirement of eight phone calls, the other had between one and 22 calls. The duration and frequency of group sessions was noted for one study.
**9. Tailoring**	Both interventions required the tailoring of care and exercise routine planning based on needs and preference of the participant. One study had tailored referrals
**10. Modifications**	This was not mentioned in the reports.
**11. How well (planned)**	One report describes the use of training and supervision to intervention providers to ensure fidelity. The other intervention promoted home exercise sessions to improve compliance with this aspect of the intervention.

**12. How well (actual)**	This was not reported in detail for either study. One report mentioned that generic issues rather than self-management strategies were more commonly discussed in contact sessions. The other intervention found that participants were reasonably willing to attend sessions but participation at exercise classes was lower than anticipated.

Group: Education, multifactorial-action and review with medication review

There are three interventions in this group: Newcomer 2004[583], Ploeg 2010[590], Stuck 1995[602]

TIDieR item	Description
**1. Brief name**	
**2. Why**	Goal: All three interventions had a goal of reducing health resource use and thus lowering health care costs. Two interventions aimed to provide timely and comprehensive care and improve patient health. One of these and the remaining intervention aimed to prevent decline by reducing risk factors, increasing quality life years and improving health and wellbeing.<br><br>Rationale: Two interventions were based on previous research findings. Proactive and preventative approaches and appropriate use of health services and beneficial relationships with health care professionals were mentioned by these two interventions also. One of these interventions believed improved access and awareness of preventative health planning would be advantageous, whilst the other suggested that home-based care provision would be of benefit. The third intervention simply stated that the intervention would improve quality of life and reduce mortality compared to usual care.
**3. What (materials)**	All three interventions utilised referrals based upon need. Two used standardised screening measures. All three used types of recording and documentation, in care planning including one which described electronic records. Two interventions described the provision of information, one of these was about local community resources. One intervention provided aids and equipment as and when needed.
**4. What (procedures)**	All three interventions involved a multidomain assessment, referrals from this, as well as some form of care planning process following the assessment. Additionally, all three interventions provided educational materials in some form. Two reports described the monitoring process. Three reports described interaction to promote empowerment of participants, including coaching and encouragement from the providers. One intervention included communication from the participant to the primary care provider, one mentioned involvement of the family physician and one described the review process.
**5. Who provided**	All three interventions were primarily provided by Nurses, though each was described differently, one as a Nurse Case Manager, one as a Home Care Nurse and one as a Geriatric Nurse. Two reports mentioned the input of the Family Physician or Primary Care Physician, other input on these two interventions came from health care professionals as needed. Research Assistant input was required for one intervention.
**6. How**	Two interventions involved the initial assessment being undertaken face-to-face at the home of the participant. Follow up contact could be by telephone. In the other intervention initial screening was undertaken by post, with the option for telephone or face-to-face assessments if required.
**6b. How organised**	All three interventions described the bulk of organisation by the nurse provider and some input organisationally by various health care professionals as needed following referrals. Medication reviews were part of all interventions and two mention specialist input. One intervention required input from project geriatricians, another mentioned the participants taking an active role. Two mentioned input from the family doctor.
**7. Where**	Two interventions were undertaken in the USA and one in Canada.<br><br>Two interventions were undertaken at home, one predominantly involved self-assessment. Follow up care was provided in a variety of locations one was specifically at home, while one other described community care settings.

**8. When and how much**	Only one report described detailed inclusion criteria, while one invited participants from the voter registry. The one which described inclusion criteria included those who were enrolled on a specific health insurance programme for a minimum of a year, had a high risk of adverse health outcomes and were aged 80 and over or 65 and over with one chronic health condition.<br><br>The nature, duration and frequency of contact was very variable across the interventions. One involved the potential for daily contact for a period of time while others only had three required contacts. All three reports mentioned that contact was as per requirement but in addition to routine reviews.
**9. Tailoring**	All three interventions mentioned some tailoring to the need of the participant. One varied the mode of assessment (postal, telephone or face-to-face) to need. The frequency and nature of contact was tailored to need as were the referrals to services in all three interventions.
**10. Modifications**	Only one report described modification, this was to care protocols during the research project.
**11. How well (planned)**	One report did not describe any steps taken to measure fidelity or promote adherence. Two interventions described the documentation of adherence to treatment or appointment by participants and reasons for not adhering. The process of care was described as collected in one report as was physician co-operation in adherence.
**12. How well (actual)**	One intervention described how 42 participants were contacted to establish reasons for non-adherence. Another inferred that the bulk of participants received the minimum required visits (n=3), as three was the mean average number of visits received. In the other report detailed descriptions were made of adherence by both participants and the professionals involved. Five thousand six hundred and ninety-four recommendations were made across all participants with an average of 28.8 per participant, over half of these were not fully complied with although adherence was stable across the duration of the study. Major problems were more likely to be identified in the first year of involvement, while therapeutic and preventative recommendations were similar over time. Adherence was better from physicians than referrals to other professionals or community services or those requiring self-care.

Group: Education, multifactorial-action and review with medication review and self-management strategies

There are five interventions in this group: Coleman 1999[521], Counsell 2007[522], Meng 2005[574], Metzelthin 2013[576], Stuck 2015[604]

TIDieR item	Description
**1. Brief name**	
**2. Why**	Goal: The goals of the five interventions were closely aligned, though differently described. Three interventions were clearly seeking to improve the health, function and quality of life of older people, two of these sought to do this by identifying risk factors for decline, two of these three also sought to promote self-management of health. Of the other two interventions one sought to change how primary care was delivered, increasing ancillary support to manage unmet needs in the chronically ill. The other was focused on improving geriatric care, driving down costs and reducing long-term care admissions. Reducing care costs was mentioned by three reports in all.<br><br>Rationale: All studies were rationalised through previous research, three through existing study findings and four through reviews. At least three had also got a theoretical grounding, often in behaviour change theory. One had used intervention mapping from existing findings. One study was heavily grounded in the idea of person-centred care and the need to manage frequently undiagnosed geriatric syndromes. One study was also informed by policy recommendations.
**3. What (materials)**	A range of materials were used in the five interventions. Four reports describe training materials and protocols for providers. Two of these and the remaining other used treatment strategies for specific conditions. Two interventions utilised existing health records while one described their use of validated assessments. At least two reports described referrals and communication with professionals. One mentioned case management and care planning materials. Two interventions explained the input of the participant, one

	of these provided self-management videos to study participants, another used motivational interview materials.
**4. What (procedures)**	All five interventions were based around an initial assessment of needs, four of these were described as multidomain, one of which is formulated from electronic software designed to draw information from pre-existing patient records. Information from assessments was discussed by a multidisciplinary team in four interventions, three of which explicitly described planning from this assessment. Four interventions explained the review process and timing which varied. Three of these and the remaining other described the provision of self-management advice, though again the approach to delivering this varied. One intervention mentioned the process by which pharmacist input was implemented, though all assessments incorporated an aspect of medication review. One report described the provision of assistive devices, and one detailed the input of family members in care planning and post intervention care planning. Four reports detail usual care access.
**5. Who provided**	All five interventions had major input from nurses, four of these had GP or primary care physician input as well. Other professionals were involved to varying degrees including Physiotherapists, Occupational Therapists, Pharmacists and Social Workers. Multidisciplinary input was described to varying degrees as being when it was required. Three reports explicitly refer to the provision of specialist training on delivery of the intervention.
**6. How**	For one intervention provision was not reported. For the remaining four, all were provided face-to-face and at home with some provision over the telephone in all instances. Three interventions were provided individually, one of these with a family care giver if one was available.
**6b. How organised**	Organisation of the five interventions varied, although all five required organisational input from nurses to some degree. Three of these also required organisational input from GPs or primary care physicians, these same three place responsibility on the multidisciplinary team for organisation of sessions and ongoing care needs. Two reports described case conference sessions while one detailed the organisation of clinics.
**7. Where**	Three interventions were undertaken in the USA, one in The Netherlands and one in Switzerland. Four interventions took place in the participant's home, while the other was carried out in clinics and practice rooms at the health care facility undertaking the study.
**8. When and how much**	A variety of inclusion and exclusion criteria were listed for the studies. Three had minimum age limits, two of these being 65 years of age and over and one being 70 and over. Three studies required the participant to be identified as at risk of increase care needs, two included frailty status as part of their inclusion criteria. One included those with a reduced income level, while two others were limited to specific primary care practices. One study excluded those with limitations on basic activities of daily living, cognitive impairments or terminal diagnosis, while two others included those with some limitations on (instrumental) activities of daily living.
**9. Tailoring**	All five reports described how the intervention was tailored to the participants needs in line with their assessment. Three also incorporated the preferences of the participant. Four interventions provided contact levels ad hoc so these were also varied. The intervention with group sessions tailored discussion to the needs of the group.
**10. Modifications**	Only one intervention described modifications made during delivery. This was necessary due to changes to funding and reassignment of services. Steps were taken to ensure that delivery was as close to randomisation as possible.
**11. How well (planned)**	All five reports described steps taken to improve adherence and measure efficacy and fidelity to the intervention, although some to a minimal degree. Four reports detailed the use of trained providers and two of these also used ongoing supervision to improve fidelity. Two also used existing record keeping processes to improve adherence. Two interventions included a process evaluation, one of which had been supported by feasibility work. One other intervention had also undertaken feasibility studies.
**12. How well (actual)**	Studies had varied success with their intervention implementation. One study found that although no benefit could be shown in results, participants expressed satisfaction with the intervention. One other study reported high levels of adherence to meeting requirements and care planning, suggesting that non-adherence to care planning was often related to participant reluctance. A third study found that most aspects of the intervention were well complied with, and home visits were well received, however some aspects were not complied with by providers or participants. This was similarly seen in the fourth report, where implementing the full

	protocol was problematic, assessments were conducted but care planning not always successful. Though participants were most often considered as committed to the plans. For the final study, which was undertaken through self-assessment, over 85% of assessments were returned and almost 60% of participants remained in the project for a full two years, although some aspects of the intervention were not as successfully adhered to as others.

Group: Education, multifactorial-action and review with self-management strategies.

There are two interventions in this group: Hattori 2019[545], Moll van Charante 2016[577]

TIDieR item	Description
**1. Brief name**	
**2. Why**	Goal: One case was focussed on improving independence by encouraging self-management skills whilst the other case was focussed on reducing the incidence of dementia and cardiovascular disease, and the burden of functional disability in the elderly.<br><br>Rationale: Both cases were based on previous research. One case was based on the association between vascular and lifestyle risk of dementia and the potential to prevent dementia if risk factors are agreed. The other case on the other hand was based on effectiveness of multicomponent interventions
**3. What (materials)**	Varied devices used in both cases. Both cases used equipment to measure care goal activities. One case used original assessment for comprehensive clinical assessment, assessment sheet for self-management and booklet for preventing long-term care needs. The other case used detailed protocol which guided recommendations and referrals.
**4. What (procedures)**	Both cases started with comprehensive clinical assessments and a joint discussion of care goals and planning. Both cases involved training of staff during intervention provision.
**5. Who provided**	One case had intervention provided by a rehabilitation specialist such as an OT or physiotherapist with training by a care manager. The other case involved a practice nurse with supervision of a GP. Both cases had other professionals like dietitians, dental hygienists and other specialised health professionals also participating when required.
**6. How**	One case was delivered individually but the other case was presumed to be individually delivered.
**6b. How organised**	In all cases, mechanisms were in place to facilitate care coordination including meetings to discuss patients' goals with at least one case conference and a practice nurse under supervision of a GP who coordinated the intervention.
**7. Where**	One case was in The Netherlands in general practices organised in health centres and the other case was in Neyagawa, Osaka, Japan in a long-term care insurance system for people with mild to severe disability. One case was delivered face-to-face and the other case was presumed to be delivered face-to-face.
**8. When and how much**	In one case, the intervention was for five months which included one home-visit, up to 12 modules weekly lasting two to three hours and one review module. The other case was a nurse-led intervention every four months for six years and a total of 18 visits to the GP.
**9. Tailoring**	The care plan for both cases were tailored based on the participant's needs assessment and goals.
**10. Modifications**	Not mentioned in both cases.
**11. How well (planned)**	In both cases, measures were taken to promote fidelity through supervision and monitoring of the interventions. One case monitored the intervention through regular visits to the practice nurses. The other case did not state specifically who did the monitoring and supervision.

**12. How well (actual)**	One case had a high attendance rate with 76% attending at least one module and 66% attended at least seven modules. The other case had a relatively high drop-out rate with 544 participants receiving less than two visits per year before the end of study.

Group: Exercise

There are seven interventions in this group: Giné-Garriga 2020[538], three arms of Morey 2006[579], Morey 2009[580], Morgan 2019[581], von Bonsdorff 2008[624]

TIDieR item	Description
**1. Brief name**	
**2. Why**	Goal: Two reports did not discuss the goal of their intervention. The remaining five all stated that a primary goal was to increase physical activity, one also mentioned the reduction of sedentary behaviour. Further aims included the improvement of health, function, quality of life and retention of independence, the reduction in disability and need for supportive services are also mentioned. One intervention explicitly refers to long-term behaviour change.<br><br>Rationale: Six reports refer to a theoretical basis for behaviour change and motivational techniques. Two reports refer to reviews undertaken to ground their intervention, while three discuss evidence-based effectiveness. Two studies were based upon feasibility or previous interventions. The benefit of motivational support is highlighted in three reports and at least three used recommendations and guidelines for healthy physical activity to ground development of their intervention.
**3. What (materials)**	A variety of materials were used across interventions. A core component was the provision of written materials to the participant, in five interventions these were based on physical activity promotion, either exercise tips or advice about physical activity services in the area. Two interventions provided materials unrelated to physical activity such as general health promotion. Four interventions mention activity planning in their materials used. Access to care notes to include activity planning or to gather information as required in five interventions. Three studies provided pedometers to participants, two provided training equipment. Documentation to track activity was provided to participants in two studies. Progress reporting was mentioned in two interventions. Referrals were mentioned in one study. Three reports mention manuals and scripting or fidelity assessment in their reports.
**4. What (procedures)**	All seven interventions involve the development of a tailored plan to promote physical activity, five studies explicitly mention input from participants on the development of this. Two studies also describe a focus on strength and walking or balance training. A pedometer was provided in two interventions. Support to set and maintain goals is mentioned in five studies. Follow up support and review were also mentioned by at least five studies, although how this was provided (either by post, phone or face-to-face) is not always clear. The provision of health-related information is mentioned by three reports, three reports explicitly refer to behaviour change techniques. Usual care is mentioned as available in three interventions. One study mentions using referrals as part of the intervention.
**5. Who provided**	The intervention was provider by a range of individuals. Six reports detail the need for providers to be trained in the specifics of the intervention. Five interventions had input from primary care physicians or GPs and four from health counsellors. Two studies involved nurses or healthcare workers. Others involved in delivery included a qualified fitness instructor and a physiotherapist.
**6. How**	All seven interventions were provided individually and face-to-face. Five interventions also use telephone contact and two used the postal system. Group contact is mentioned in two interventions and one explicitly refers to the follow up process. One intervention preferred participants to have the support of a family member or loved one. Motivational interviewing and strategies for motivation, problem solving, goal setting and self-management are mentioned as key in five reports.
**6b. How organised**	One report does not mention how the intervention was organised. The six other interventions involve primary care providers or GP input, counsellors had an organisational role in three interventions and participant input was required in four interventions. A university, trained facilitators, the physiotherapist, and the local health

	and social services had input in one intervention. It was noted in three reports that the intervention was underway during a time of change in health promotion services.
**7. Where**	Interventions were delivered in a range of locations; although it was not always clear what was undertaken in each place. Veterans' health clinics are mentioned in fours interventions, primary care centres are mentioned in three, additionally, GP surgeries and leisure centres are identified locations in one intervention each.<br><br>Four interventions were delivered in the USA. The remaining three were in various European locations, one in the UK, one in Finland, and one was delivered in four countries including Denmark, Northern Ireland, Germany and Spain.
**8. When and how much**	The criteria for inclusion in the intervention were varied. Five interventions carried a minimum age, one was 65 years and over, the remaining four were 70 years and over. Four involved veterans only, one recruited old volunteers. Three studies assessed physical ability to safely take part and three required participants to have high levels of sedentary behaviour. Two studies required participants to not have dementia or at least be cognitively intact. Fours studies excluded those with high physical activity levels. Four excluded those with terminal diagnoses or specific health conditions.<br><br>The nature, duration and frequency of input varied over the interventions. One intervention provided 32 face-to-face exercise sessions, twice weekly for 16 weeks. While the majority (four) only mention one face-to-face contact. Telephone contact varied between three and 13 calls. One intervention mentions mailed updates. Intervention duration ranged from 16 weeks to two years.
**9. Tailoring**	All seven reports detail some level of tailoring in line with the participant's ability and capacity. One describes tailoring following progress by the participant. One suggests the involvement of friends of family is optional.
**10. Modifications**	Not mentioned in any of the reports.
**11. How well (planned)**	One study did not mention how they were promoting adherence or measuring implementation fidelity. Adherence to the intervention was promoted through the telephone contacts in at least two interventions. Involvement of the primary care provider was also seen as beneficial to adherence in one report. Implementation and fidelity to the intervention was measured and analysed in at least two interventions. One report explicitly mentions the use of qualitative approaches such as interviews to evaluate the intervention.
**12. How well (actual)**	One study did not mention how well the intervention was implemented. Four reports mention positive endorsement by the service provider. Three interventions report on flexibility with phone call delivery. Two interventions report that all participants received baseline input. One describes minimum dosage input being received by all participants. One report states that the anticipated duration of delivery was as intended. Call delivery was above 90% in one intervention, another reported that at least 302 of 318 participants received a minimum of four calls.

Group: Exercise and psychology

There are three interventions in this group: Alegria 2019[503], Jing 2018[554], van Heuvelen 2005[617]

TIDieR item	Description
**1. Brief name**	
**2. Why**	Goal: to improve physical and psychological health / reduce physical and mental disability.<br><br>Rationale: Previous demonstration of effectiveness of each component, including psychological benefits of physical exercise. The combination was expected to provide further additional benefits.
**3. What (materials)**	Some provided equipment and instructions for the exercises and materials to support the psychological tasks.

**4. What (procedures)**	All the interventions provided exercise sessions and psychological training.<br><br>One intervention additionally provided encouragement calls to continue practising.<br><br>One intervention additionally provided regular mood screening.<br><br>Staff were provided with training prior to intervention in two studies, and regular supervision in one.
**5. Who provided**	In some cases, specialists in exercise or psychological training provide the relevant component. In others the provider is a community health worker or nursing student.
**6. How**	Both physical exercise and psychological training were provided individually and in group sessions in different combinations.<br><br>The interventions included face-to-face contact as well as telephone calls. In one intervention, the two components were provided as parts of one session.
**6b. How organised**	Few details of organisation provided.
**7. Where**	China, The Netherlands and USA.<br><br>The intervention took place in community facilities and at the participant's home.
**8. When and how much**	Started in different circumstances:<br><br>(a) participants did not have cognitive impairment and were not very active;<br><br>(b) participants were housebound;<br><br>(c) participants had low mood and mild-moderate disability.<br><br>Physical exercise session frequency was between three times per week and once every two weeks for approximately 3 months. Sessions continued at greater or lesser frequency or not at all after this for an additional 6 weeks to 3 months.<br><br>Psychological training occurred for 18 weeks to 6 months, at a frequency of every 2 to 2.5 weeks for at least 3 months, with step-down to monthly training for the last 3 months in one.
**9. Tailoring**	The psychological training was tailored to individuals' problems in two interventions. Optional remote delivery was available in one intervention depending on participants' circumstances.
**10. Modifications**	Not mentioned
**11. How well (planned)**	One provided feedback on delivery, which was recorded. One encouraged participation by offering transport and sending newsletters. Encouragement calls or personal attention were also detailed in two interventions.
**12. How well (actual)**	Most participants did not attend all sessions in the two studies that reported details.

Group: Homecare

There are 12 interventions in this group: Auvinen 2020[505], Bernabei 1998[508], Dupuy 2017[527], Fernandez-Barres 2017[531], Fristedt 2019[535], King 2012[557], Lewin 2013[565], Mann WC 1999[571], Rooijackers 2021[594], Teut 2013[609], van der Pols-Vijlbrief 2017[615], Wolter 2013[629]

TIDieR item	Description

**1. Brief name**	
**2. Why**	Goal: Only three of 12 interventions mentioned an explicit Goal, where the goal was to provide care and support to older people enabling them to stay at home. One added the goal of continuity of care for older people.<br><br>Rationale: Ten reports made no mention of the rationale for the intervention, one mentioned the importance of responding to the needs of older people and the other identified that caregivers were an important resource for the care of older people.
**3. What (materials)**	Eight reports did not mention the materials used for the intervention. One intervention mentioned the use of fake sensors as this was a control group, another mentioned care plans to identify support needs, one mentioned likely equipment for carrying out usual care and one other provided participants with a healthy diet brochure.
**4. What (procedures)**	In all interventions there was some reference to the provision of usual care, including home care services. Two reports mention assessments being carried out as part of usual care practice and one of these interventions developed care plans from this assessment.
**5. Who provided**	Intervention provision was by a range of practitioners. In four interventions this was by nurses, one of these was supported by a doctor. Four other interventions were provided by paid care support workers. External co-ordination was mentioned in two other interventions. Five interventions mentioned the input of a range of health and social care professionals to carry out care as required.
**6. How**	Delivery was not always described. Eight interventions were delivered individually and ten face-to-face. One intervention mentioned that some group input may be part of some intervention input.
**6b. How organised**	In one report the organisation was not described. Of the remaining 11, four were organised by home care providers, two had external coordination, two had state input mentioned in reference to organisation. Other individuals mentioned include nurses, home care staff, care providers and nurses.
**7. Where**	One report does not state a location of provision. Of the remaining 11, eight were undertake in European locations, including Germany, Spain, The Netherlands, Finland, Sweden, Italy and France. One intervention was based in the USA, one in New Zealand and one in Australia. Whilst it was not always explicitly mentioned the nature of provision suggests that the intervention was provided in the participant's home.
**8. When and how much**	There were various inclusion and exclusion criteria for eligibility to the intervention. Ten reports mention that participants needed to be in receipt of usual care. Additionally age limits of age 65 and over or age 75 and over were prerequisites for eligibility in three instances. Additional requirements for inclusion were that no previous assessments were undertaken on the participant, that they had high level need or polypharmacy, that they were house bound, that they had a frailty level which indicated decline over the preceding six months, that they resided within a certain housing community or that they were undernourished. Only three interventions specified exclusion criteria, one excluded those with the highest level of need, two excluded those with cognitive impairments, one of these also excluded those who were terminally ill or bedbound.<br><br>The nature and frequency of contact was rarely described, for four reports it was and then it was assumed that this would vary according to need.
**9. Tailoring**	Four interventions mention that provision would be tailored according to the need of participants. The remaining eight do not describe tailoring.
**10. Modifications**	No reports mention modification to their intervention.
**11. How well (planned)**	No reports mention steps taken to improve implementation or adherence to the intervention.
**12. How well (actual)**	Not mentioned in many reports though one report found that home care staff promoted reablement principles that were not part of the intervention.

Group: Homecare and multifactorial-action

There are five interventions in this group: Parsons J 2012[585], Parsons M 2012[587], Parsons M 2017[586], Tuntland 2015[614], Whitehead 2016[627]

TIDieR item	Description
**1. Brief name**	
**2. Why**	Goal: Although the goals of the interventions were differently described the overall focus was to enable older people who were identified as requiring support to live at home, to maintain home living. Identification of the appropriate level of care appears to be a key aim across interventions. The promotion of health related quality of life, independence and social connectedness was mentioned as an explicit aim of one intervention. Rehabilitation as set at an appropriate level was mentioned by another. One other mentioned reducing care costs was an aim.<br><br>Rationale: Two reports did not explicitly state a rationale. One is somewhat ambiguous stating that there is a need for appropriate home care service provision. One intervention was based on evidence that older people often lose function when in hospital and those who do lose function often fail to regain it. This intervention suggests that home care has the potential to improve this situation. One intervention is based on Care Act guidelines around care provision and that a key component of this is empowerment and reablement.
**3. What (materials)**	Materials were minimally described in the reports. Three of the interventions described using standardised assessments. One mentioned the development of care planning with client input. Two others describe accessing services or equipment through referral systems as required.
**4. What (procedures)**	The process of the intervention was not always clearly described. Four interventions describe an assessment process; one of these is at a six week time point to identify continuing needs. Care planning following from the assessment is stated by one intervention. Four interventions explicitly refer to access to standard home care service provision and other healthcare services remaining in place. One intervention described little other than to indicate that the care package was designed to include input from family and community services.
**5. Who provided**	Assessments were conducted by needs assessors and processed by healthcare co-ordinators for two interventions. One of these also mentions the input of the research team. Home care aide input is mentioned explicitly by two interventions. The role of those undertaking assessments in not clearly specified in three interventions. One intervention mentions reablement workers and social care managers. Input from additional healthcare professionals as required is mentioned in the delivery of all five interventions.
**6. How**	It is not always clear how assessments were undertaken, two interventions mention this being an individual assessment but with no indication that it was face-to-face. Three interventions mention the provision of care being face-to-face and individual in nature. One other clearly states that provision of care is face-to-face and at home.
**6b. How organised**	A range of organisations and individuals were involved in the organisation of the interventions, three interventions utilised an assessment agency with needs assessors to undertake assessments. One of these interventions also had organisational input from the research team, home care co-ordinators and home care aides. Two interventions had healthcare organisation input, one of these reports mention funding by the health district board. One intervention mentions organisation by the relevant healthcare professional providing care. Another was organised by reablement workers for an initial six weeks, then an Occupational Therapist and home care service should continued care be required.
**7. Where**	Three interventions were implemented in New Zealand, one in Norway and one in the United Kingdom.<br><br>Four interventions mention delivery of the intervention at home.
**8. When and how much**	Eligibility to all five interventions was after referral to home care services. One of these required this to be on hospital discharge.

	The nature, duration and frequency of intervention input was not always mentioned. Two interventions note that input frequency and duration was varied. One states that there was no time limit to input. However one other conducted six weeks of reablement followed by homecare as required after this time point.
**9. Tailoring**	All five interventions were tailored in line with the needs of the individual. One mentions consideration of the preferences of the individual, whilst another was tailored to the effort given by the individual. Three mention flexibility over the duration of input.
**10. Modifications**	Four reports do not mention any modifications. One intervention required modifications to be made following changes to the recruitment approach of Occupational Therapists in the service.
**11. How well (planned)**	Three reports do not mention any plans for adherence or fidelity assessment. One report describes the collection and analysis of the care planning documentation. The other conducted a cost analysis identifying the number of contacts, the provision of equipment and individual reported additional service use.
**12. How well (actual)**	Three reports did not undertake adherence or fidelity assessments. One intervention found that 15% of care plans documented individualised activity related to functional improvement. The other intervention remarked on changes to the intervention due to changes in the recruitment of staff.

Group: Homecare, ADL, multifactorial-action from care-planning and review with self-management strategies

There are three interventions in this group: King 2012[557], Parsons M 2017[586], Rooijackers 2021[594]

TIDieR item	Description
**1. Brief name**	
**2. Why**	Goal: The three interventions all aimed to improve, restore, promote or maintain independence or function. One intervention also had a goal of improving wellbeing of participants. All three interventions desired improved service provision. One intervention aimed to reduce admission to long-term care.<br><br>Rationale: All three interventions were based on previous studies or existing models of working which showed benefit to older people. Studies mentioned a theoretical basis in the evidence for restorative approaches and social theories.
**3. What (materials)**	All three interventions used a range of assessments over a number of domains, one explicitly referred to psychological, social and physical components. Two reports detailed training materials for staff and one intervention mentioned goal setting documentation, action planning documentation and exercise booklets for participants.
**4. What (procedures)**	All three interventions required a multidomain assessment to be undertaken, all three were also co-ordinated by a nurse. Goal setting, care planning and tailored exercise planning is also part of all three interventions. Regular review, referrals and staff training are each mentioned as part of one intervention. Usual care is available across all interventions.
**5. Who provided**	Registered nurses and support workers are involved in the provision of all three interventions. One intervention involved multidisciplinary input as well. Training was important for providers of all three interventions.
**6. How**	All three interventions were provided face-to-face, three mention this being on an individual basis. One intervention mentions telephone contact as well.
**6b. How organised**	All three interventions appear to have nurse co-ordinators as a core organisational feature. Support worker input is key across all three interventions as well. Hospital staff were involved in one intervention, a physiotherapist or occupational therapist in another. Funding is through healthcare insurance for one intervention while one other mentions input from charitable services.
**7. Where**	All three interventions were delivered at home. Two interventions mention involvement of one key healthcare provider.

	Two studies were undertaken in New Zealand, one in The Netherlands.
**8. When and how much**	All three interventions required participants to be in receipt of home care. One had an age limit of 65 years and over. One recruited those with high levels of need placing them at risk of long-term care admission. One study placed a language restriction on participants. Only one study excluded those with serious or terminal illness or cognitive impairment.<br><br>The duration and frequency of contact varied across the three interventions. Contact ranged from multiple daily contacts to a minimum of once a fortnight. One mentions four-six months of input, and one mentions 12 months duration. Two interventions mention reassessment, one at 12 months one at six.
**9. Tailoring**	All three interventions included tailored care planning according to the assessment of participants. Two mention this being in conjunction with the participant. One describes the adaptation of visits according to need.
**10. Modifications**	This was not mentioned by any of the reports.
**11. How well (planned)**	One study did not mention any attempt to assess implementation or fidelity. The two other studies both delivered training and support to intervention providers. One study undertook feasibility work. One promoted adherence thorough prompts to providers. One report details a process evaluation through the collection of documents about records and qualitative methods.
**12. How well (actual)**	One study did not mention any attempt to assess implementation or fidelity. A variety of findings were reported by the remaining two studies. One was delivered during the expected timeframe, follow up calls were received between 70-89% of the time, although over 50% of initial assessments did not identify tasks. The other intervention describes barriers to implementation such as low staffing and resistance from clients, whilst additional funding and digital care planning facilitated implementation. Compliance measured as 73%-86% for attendance at over half of the meetings, over 50% of assignments were completed by team members. Staff were noted as perceiving change as positive due to the intervention.

Group: Homecare, multifactorial-action and review

There are six interventions in this group: Hall 1992[543], Markle-Reid 2006[572], Parsons M 2012[587], two arms of Ryvicker 2011[596], Shapiro 2002[598]

TIDieR item	Description
**1. Brief name**	
**2. Why**	Goal: The six interventions had a number of goals, four of which promoted the improvement of function in older people with some level of dependency through home care to enable independent living for as long as possible. Two reports indicated that reducing service duplication and integration of services by promoting better across service communication as additional aims. Improving the home care service by optimising the role and enhancing retention was an aim of one other intervention. One other intervention indicated the early input of interventions to promote proactive care as a goal.<br><br>Rationale: one report did not describe the rationale for their intervention. Two interventions suggested that integrated care approaches appear beneficial to support the holistic care neds of older people. Two interventions were based on previous research. One intervention suggested that that are barriers to providing successful home care and this impacts on home care worker retention and the outcomes of those using the service. One other report identified the potential that home care provision has to improve the wellbeing of older people.
**3. What (materials)**	Two reports did not describe the materials used in their intervention. The remaining four all described a variety of assessments for older people, some of these were routinely undertaken in usual care, others were specifically developed for the intervention. One intervention also accessed medical records to complete assessments. One intervention developed guidance called 'Five Promises' to aid with communication

	between older people and the staff providing care. One intervention also described documentation used in the supervision of staff who delivered the intervention.
**4. What (procedures)**	All six interventions used an assessment to identify needs, one of these specifically involved patients' preferences. Care-planning was explicitly developed from this assessment in four interventions, one involving family caregivers in this process. A further four describe the review process, with one again including patients preferences in this. Services were arranged as part of three interventions. Training and supervision are described in the reports of two interventions. Additionally, access to usual care is noted in four reports.
**5. Who provided**	A range of professionals were involved in the provision of the interventions; many by multiple individuals and roles. Nurses were involved in three interventions and case workers in three as well. Access to a multidisciplinary team was mentioned in four interventions, though this was sometimes in conducting assessments and at other times in carrying out care. A personal support worker was mentioned in one intervention. Another mentioned the need to ensure providers were trained in intervention delivery.
**6. How**	One report did not describe how the intervention was delivered. Four interventions were delivered face-to-face, four at home and four individually (although these were not always the same four). One intervention also used telephone contact and another required input from a caregiver.
**6b. How organised**	Organisation was variable. One intervention required little planning and organisation. Two interventions relied upon case managers to organise the intervention, clinicians were involved in care planning for three interventions. Nurse input was described as key in three interventions as was the home care team in two of these. The family caregiver had some input in one intervention.
**7. Where**	Five of the six interventions were undertaken in North America; two in Canada, three in the USA. The remaining intervention was carried out in New Zealand. Four of the six reports described delivery at home, one mentioned specific care settings, whilst the other does not state the location of intervention delivery.
**8. When and how much**	All six interventions commenced following on from assessment indicating that the older person required home care. Two of the interventions required evidence on the assessment for capacity for improvement, whilst one other required a specific level of ill-being. One intervention had a minimum age requirement of 75 years of age.<br><br>The nature, frequency and duration of the interventions was not well described, four reports described an initial assessment and then all six refer to review of this assessment, although for some this was at specific time points and others it was ongoing or as required. Only one report described duration and this was 18 months long.
**9. Tailoring**	All six interventions were designed to be tailored to the needs and capacity of the participant identified at assessment. Two interventions tailored according to participant's wishes also. One intervention was tailored to the caregiver as well; this intervention also tailored the supportive contact according to need.
**10. Modifications**	Four reports did not mention any modifications. One mentioned that there were changes to the criteria to assess support changed during implementation, impacting on the service provision. One report mentioned that there were changes made to the hours provided by home care over the duration of the intervention.
**11. How well (planned)**	Only two reports described steps taken to assess fidelity and adherence, the remaining four did not. The two that did carried out a survey on the intervention, used both standardised intervention materials and training materials for providers. They both also conducted interviews with delivery team managers and carried out observations of meetings.
**12. How well (actual)**	Four interventions were not assessed for fidelity. One report described components of the intervention were widely accepted, although some components were not considered feasible the 'Five Promises' guidance was seen to show benefit. Training was seen to be inconsistent and Clinician and commitment to support the intervention was variable. The other report described how there was little capacity to implement the intervention.

Group: Homecare, multifactorial-action from care-planning and review with medication review

There are three interventions in this group: Bernabei 1998[508], Fristedt 2019[535], Wolter 2013[629]

TIDieR item	Description
**1. Brief name**	
**2. Why**	Goal: All three interventions had a goal of reducing institutional admissions be that to hospital or long-term care, one also sought to reduce the cost of providing care to older people. Two reports mentioned the aim of improving function, one report implied this would improve quality of life for older people. One report describes the need to improve communication between services.<br><br>Rationale: One report mentioned the need to integrate social and medical services to provide clarity over their purpose, this intervention was also grounded in policy recommendations and the wishes of older people themselves. The two other interventions were developed following on from previous research showing the benefit of such interventions. One of these was part of a government policy to improve quality of care, the other was to identify deficits in current care processes.
**3. What (materials)**	All three interventions included assessments of need, though these varied in type they were designed to cover multiple domains of need, with a requirement for sufficient data to develop a care plan. Two interventions required access to existing medical records, one of which also required agreement for care planning with the participants GP. Two interventions mention equipment required by staff such as transportation and a laptop in one intervention, and a protocol for conducting assessments in another.
**4. What (procedures)**	All three interventions conducted multidomain assessments. Two explicitly refer to care planning from this. One intervention describes the monitoring process following assessment in detail while the other two mention reviewing. Two interventions mentioned the staff training process and one describes the need for agreement of care plans with the participants care providers. All interventions included access to usual care services, although one does replace some existing service provision with the intervention multidisciplinary team.
**5. Who provided**	One intervention is provided by a case manager and the participants GP as well as a specialist trained multidisciplinary team. A multidisciplinary team provides one other intervention, while the third is provided by home care nurses and staff. All reports mentioned the input of multidisciplinary team members as per usual care needs.
**6. How**	All interventions were provided individually and face-to-face. One intervention mentioned that some of the services accessed may be in different locations and in group contexts as was relevant. Another intervention required collaboration with participants and/or their relative(s).
**6b. How organised**	The interventions were organised differently. Although two interventions relied upon some level of state involvement. One of these involved input from existing services, case managers and the GP and used weekly sessions to discuss the intervention implementation. The other of these the geriatrician for the intervention took over the primary care responsibilities for the participants. The third intervention was organised by the home care service and nurses.
**7. Where**	All three interventions were undertaken in Europe, one in Germany, one in Sweden and one in Italy. All three were implemented at home. Two reports mentioned the system of care in which they were operating, this was varied, and integration of services was mixed.
**8. When and how much**	Two interventions were accessible to participants upon receipt of home care services, although one of these specified no previous assessment and planning to be undertaken. The other interventions criteria were participants being over 75 years or age and having a level frailty which indicated decline over the previous six months.<br><br>The nature, duration and frequency of input was not described in detail by the reports. One intervention lasted for 12 months with input over alternate months; another intervention had an assessment visit then varied amounts of contact over the following 15 weeks. The other report does not mention input explicitly, although reassessment is mentioned.

**9. Tailoring**	All interventions could be tailored to the needs of the participant, one was also tailored to the needs of the relatives. Two interventions also mentioned the provision of support from providers being tailored to needs and wishes of participants.
**10. Modifications**	There were no mentions of modifications to the interventions in any reports.
**11. How well (planned)**	Two reports did not mention any steps taken to improve fidelity or measure adherence. However, one explicitly mentioned that staff providing the intervention received training and advice, and support was on hand to improve fidelity.
**12. How well (actual)**	Two reports did not describe fidelity or implementation of the intervention. The other report however described how the intervention saw an increase in the level of care planning and in keeping care plans up to date. Although, implementation of the intervention varied between providers, some being able to implement well or rapidly (optimal) and others unable to implement even over a longer period of time (sub-optimal). Nurses feedback indicated that a year was needed to implement. Further analysis indicated that there were certain factors associated with improved implementation including services with higher levels of qualified staff, staff having lower workloads, and smaller services were more likely to implement well.

Group: Homecare, multifactorial-action and review with self-management strategies

There are two interventions in this group: Hall 1992[543], Parsons J 2012[585]

TIDieR item	Description
**1. Brief name**	
**2. Why**	Goals: The goals of the two interventions were broadly aligned. One intervention stated assisting frail older people to live at home for longer and sustain their total wellbeing as a goal, the other focused on the restoration and maintenance of function as well as engagement with community services. Both interventions focused on the empowerment of older people to take control of their own lives, one intervention also wished to change the philosophy of home care provision from increasing dependence to promoting independence.<br><br>Rationale: one study was based on previous work which showed a gap in existing home care interventions. The other intervention was also based on existing evidence, suggesting that hospitalised older people often lose function and then do not regain this once back at home, identifying home care as having potential to improve this situation.
**3. What (materials)**	One intervention used a protocol to guide care planning formulation and then referrals to services. The other intervention developed and used a specific tool called Towards Achieving Realistic Goals in Elders tool (TARGET), standardised assessments care planning and client reviewing are also mentioned as materials in this report.
**4. What (procedures)**	Both interventions use multidomain assessments the planning and arrangement/organisation of care and a regular review component. One intervention stated that review was monthly. Both interventions also used supported goal-setting and had access to usual care, and home care specific to their needs based on standard existing assessments. One intervention also mentions the training of deliverers ahead of providing the intervention.
**5. Who provided**	The interventions were provided by different teams. One used nurses to conduct the assessment and carry out care. The other used trained needs assessors, home care coordinators, and home care aides as well as the research team in the delivery of their intervention. Both reports mention access to healthcare professionals as needed.
**6. How**	Both interventions are provided to individuals, however only one states that this is face-to-face, the nature of the assessment for the other intervention is unclear.
**6b. How organised**	Organisation of the intervention also differed. One is organised by a nurse who provides referrals based on their assessment then community services arrange relevant services. The other intervention requires the assessment agency to conduct needs assessments, the home care agency to coordinate this, and the

	coordinators to plan and review the relevant care to be provided by home care aides. The research team are also mentioned as having organisational input in one intervention.
**7. Where**	One intervention was conducted in Canada, the other in New Zealand
**8. When and how much**	Eligibility for both interventions was on enrolment or referral for personalised care at home. In both cases this was based on standardised assessments identifying this need, visits and support were according to need. One intervention mentioned specific review at three and 12+ months.
**9. Tailoring**	In both cases care planning was tailored to need and with preferences identified by the participant based on their multidomain assessment. One intervention also mentions that usual care was also based on the need of the client.
**10. Modifications**	Modifications were not mentioned in either report.
**11. How well (planned)**	One intervention did not mention any plans for adherence or fidelity assessment. In the other intervention support plans and details of services accessed were collected and analysed, the number of reviews undertaken by home care coordinators was also gathered.
**12. How well (actual)**	One intervention did not report on adherence or fidelity. The other intervention identified almost 2/3rds of planning included activity targeting functional improvement. However, the review process was not increased by use of TARGET. 85% of participants engaged in goal setting and 10 referrals to allied health professionals were made.

Group: Meaningful activities and education

There are two interventions in this group: Clark 1997[519], Clark 2012[520]

TIDieR item	Description
**1. Brief name**	
**2. Why**	Goal: The Interventions were both designed to benefit the physical, psychological and functional health of older people. One also mentioned attending to cognitive health as well. Both interventions had the aim of reducing decline; this was to be targeted through engaging people in meaningful activities. One study mentioned education to inform better health practice in older people. The other intervention mentioned that they targeted an ethnically diverse population, and a desire to embed the intervention in everyday routine.<br><br>Rationale: Both interventions were developed based on previous study, additionally both interventions mention occupation specifically as part of successful ageing. One of the interventions rationalised development through an occupational science theoretical basis, acknowledging that occupation is socially generative and productive. Previous study had been used to select components of this intervention for evidence based benefit. The other intervention indicated that activity and lifestyle are modifiable factors for targeting change.
**3. What (materials)**	One report did not specify the materials they provided although it was noted that they were culturally adapted for the population. The other intervention provided educational materials including '25 ways to stay healthy' which was developed by participants, a life redesign journal and an instructional video on crime prevention.
**4. What (procedures)**	Both interventions consisted of the same procedures. This was to provide educational sessions on various topics to groups of older people. Additionally individual education sessions were provided which could be tailored to the participant. Interventions provided opportunities to take part in activity sessions. Functional training was available to enable easier engagement with activities and usual care was also noted as available to participants.
**5. Who provided**	Both interventions were provided by occupational therapists, particularly trained in supporting older people. These were able to speak appropriate languages as required. One intervention also mentioned a session being delivered by a police officer.

**6. How**	Both interventions were provided face-to-face and using both group and individual settings. Interventions aimed to facilitate peer interaction and were using psychological approaches to ensure intervention efficacy.
**6b. How organised**	One intervention report mentioned the funding source as the National Institute of Health and the American Occupational Therapy Foundation. The other intervention suggested that continuity in provision was key, and that money was available to compensate participants for taking part in activities.
**7. Where**	Both interventions were undertaken in the USA. The individual components of the interventions were provided at home. The group sessions were provided at community based sites for one intervention although the location of the group sessions in the other intervention is unspecified.
**8. When and how much**	One intervention was available to older people living in specific locations following a health assessment by a physician. The other intervention was undertaken with people over 60 years of age, recruited through various approaches within a specific location, for example targeting senior housing and community centres. Both interventions were aiming to reach culturally and ethnically diverse populations where there was an assumed health disparity.<br><br>Both interventions provided two hourly group sessions once a week, one provided hourly individual sessions once a month, the other made up to ten hours of individual sessions available, this intervention lasted six months the other nine.
**9. Tailoring**	Both interventions were tailored according to the needs and activity preferences of the individuals. These activities could be adapted over the intervention period. Both interventions could be tailored to the language of the participant.
**10. Modifications**	Neither report detailed modifications made to the intervention.
**11. How well (planned)**	Both reports detail efforts to maintain fidelity and assess adherence to the intervention. Both reports describe training to the providers. One intervention also asked participants to refrain from speaking to each other about their activity involvement to avoid contamination across activities. One intervention took steps to ensure that providers were continuous across the intervention delivery. In addition reminders were sent about activities taking place and contamination across activity provision was measured.
**12. How well (actual)**	For one intervention 65% of the participants attended at least half of the sessions. For the other intervention on average, participants attended 56% of the scheduled sessions. Whilst 17% of individuals did not attend any intervention sessions. There was some cultural variation in attendance, conflict was seen across participants but this was well managed by intervention providers.

Group: Multifactorial-action

There are nine interventions in this group: Borrows 2013[511], Botjes 2013[512], de Craen 2006[525], Grimmer 2013[540], Hay 1998[546], Siemonsma 2018[600], two arms of Stewart 2005[601], Williams 1992[628]

TIDieR item	Description
**1. Brief name**	
**2. Why**	Goal: One report did not mention an explicit goal of their intervention, the remaining eight had a variable focus around promoting independence or preventing and/or delaying dependence or functional decline in older people. For three interventions this was also to maintain living at home, and for another this was to ensure that older people could continue to contribute to society. Three interventions had an additional aim of reducing the cost of care provision. One desired the promotion of health and wellbeing for older people.<br><br>Rationale: There were mixed rationales for the interventions, one report did not state a rationale. Four interventions were based on previous research or evidence in the literature. One of these was also theoretically driven by the perceived benefit of patient involvement in care decision making. Three other interventions were based on the perceived benefit of their approach, be that by utilising specific staff expertise or by implementing specific ways of working. Further rationalisations were evident including the

	need for working proactively, which was mentioned twice, adherence to policy recommendations or that certain patient groups were specifically at risk.
**3. What (materials)**	One report did not mention the materials that were used in their intervention. For six interventions assessment documentation, be that electronic or paper based, was described. Three interventions mentioned the provision of appropriate therapy, equipment or adaptations. Two interventions described communication with health care professionals and participants in their materials. One study required access to patient clinical records and referrals. The provision of information to participants was mentioned in one report; one other describes the use of a protocol to guide care. As one intervention was based on the internet, a computer and internet access were required for this.
**4. What (procedures)**	All nine interventions described an assessment of need, for four reports this was described in more detail as being multidomain. Six interventions describe the implementation of appropriate recommendations, referrals, therapy or adaptations in line with this assessment. One of these six and one other mentioned care or action planning following assessment. Three interventions required the participants to have input on the solutions to their care needs. Three also mention access to usual care. Two reports mentioned processes designed to sustain the programme by developing community partnerships and improved communication between different support services.
**5. Who provided**	Eight of the nine interventions were provided by professionals. Three of these were occupational therapist led, one of these may have been an occupational therapist assistant at times. One intervention was physiotherapist led while another was provided by physiotherapists and occupational therapists. One was led by a research nurse, one by a health visitor and one by a social worker. The remaining intervention was conducted online however there was a volunteer on hand to provide support if required, although their background was not specified.
**6. How**	Not all reports clearly describe how the intervention was delivered. In six reports it was evident that provision was face-to-face, and in five this was individually provided. One intervention was provided both face-to-face and over the telephone. Another intervention was less clear in detailing how it was provided, although the setting appeared to be clinically based. One intervention was conducted online with the option of support for those struggling to complete the assessment questionnaire.
**6b. How organised**	For one intervention organisation was not mentioned, for the remaining eight interventions there was varied input. State or local authority input was mentioned in three reports. The relevant provider such as the occupational therapist, social worker, research nurse or health visitor was responsible for organisation in most instances. Established care providers and GP's were also involved in three interventions. Additionally, the older person was seen to have some responsibility for organisation in two interventions.
**7. Where**	Seven interventions were undertaken in Europe, four in the UK, three in The Netherlands. One intervention was implemented in Australia and one in Canada. Six interventions were carried out at the participant's home, one mentioned attending clinic and one a location befitting the participants therapy requirements.
**8. When and how much**	The eligibility requirements for inclusion were varied, though four had a minimum age requirement, this ranged from 65 and over to 85 and over. Five specified that participants had to have an evident need or referral to services. Two were following hospital discharge, although one was upon discharge from emergency services and the other from inpatient care. One intervention required participants to have a level of frailty, although the assessment was unspecified, one recruited through a pre-existing cohort. Another specified that participants have mental capacity to be included.<br><br>The nature, frequency of input and duration of the interventions was rarely described in any detail. Contacts ranged from one to 18 occasions. The duration of the intervention was only mentioned in three reports ranging from three weeks to six months.
**9. Tailoring**	All interventions involved tailoring to participants need. Two also mentioned tailoring of support level according to need and two others mentioned attending to the preferences of the participant.
**10. Modifications**	None of the reports described modifications to their interventions.
**11. How well (planned)**	Five interventions did not report taking steps to measure or promote fidelity or adherence. One intervention conducted a process evaluation of the experience of the intervention by participants. One documented

	compliance with referrals. Whilst two others promoted adherence and interest with regular contact and meetings.
**12. How well (actual)**	Four reports do not describe the success of intervention implementation. One intervention found that 60/109 participants were able to take part, and of these over 90% received a care plan. Non-compliance was explored and found to be related to lack of support or access to computer equipment. Although the experience was seen to be beneficial at times some of the suggestions were not welcomed by participants. In one other intervention 66/147 showed need, of which approximately 50% accepted support offered, of those who did not the proposed solutions was not seen as likely to help by participants. Another intervention identified that compliance for first appointments was quite good although uptake dropped off after this, however healthcare professionals rated compliance with the intervention as high for those who did attend appointments. For the two interventions which promoted adherence and interest with regular contact and meetings these were poorly attended.

Group: Multifactorial-action and review

There are 15 interventions in this group: Challis 2004[518], Cutchin 2009[523], Hattori 2019[545], Henderson 2005[548], Hendriksen 1984[549], Imhof 2012[553], Kono 2004[559], two arms of Kono 2012[560], Kono 2016[558], Lambotte 2018[562], van Rossum 1993[621], Vass 2005[622], Vetter 1984[623], Williams 1992[628]

TIDieR item	Description
**1. Brief name**	
**2. Why**	Goal: Not all reports identified a goal of their intervention, however the majority did. Goals most often focused upon older people, improvement of their health and function being a goal of seven interventions, quality of life and wellbeing a part of three of these and an aim of a further two. Supporting independent living was an aim of two, promoting self-care a focus of one other. The identification of needs was a goal of five interventions, one simply stating that older people had needs, two others identifying that these were often unmet and of a medical and social nature, two others suggested early identification of these needs was key. Other primary goals were to reduce health resource use in six interventions, in particular for long-term admissions in two reports. Four interventions also identified that accessing care and support was vital for older people.<br><br>Rationale: Not all reports were rationalised, and one was unclear in rationale. Eight interventions were based on previous research which showed benefit, four in particular focusing on proactive and preventative approaches to care. Evidence that unmet needs lead to acute care admissions was the foundation for two interventions. Two interventions had grounding in theory. One intervention was policy informed, one suggested that social care could manage many needs of older people better than primary care providers. One of the reports highlighted that they had developed their intervention collaboratively.
**3. What (materials)**	Four interventions did not describe any materials they provided. Seven described the assessment documentation they used, five used referrals and communications to other services. Four others mentioned documentation relating to summaries of the assessments, such as care plans or feedback to participants and families. Four interventions described the use of protocols, instructions, or manuals by providers. One intervention developed and used a coding system to aid with carrying out the intervention. One also described the loan of assistive equipment to participants.
**4. What (procedures)**	All interventions consisted of some kind of assessment, some described these in some detail, others used specific validated assessments, but all 15 were defined as covering multiple domains, such as physical, social, psychological and cognitive aspects. Six of these assessments resulted in the production of care plans in collaboration with participants, one of these also included family in care planning. Seven interventions provided information and advice to participants, five also provided referrals on to other services. Thirteen reports described the review process in some detail. Twelve interventions explicitly mentioned that access to usual care would be sustained for participants.
**5. Who provided**	The interventions were provided by a range of individuals. Eight interventions were provided by more than one person, the remaining seven appeared to be unidisciplinary. Ten interventions had some Nurse input although the specialisms of the Nurses was varied, including Community Psychiatric Nurses, Advanced Practise Nurses, Public Health Nurses and Community Care or District Nurses. Social services personnel

	were involved in four interventions and Health Visitors involved in two. Occupational Therapists involved in two others, one of these also involved a Physiotherapist. Other professional input came from a medical student, a geriatric specialist, a Care Manager and a GP. Four reports also described the specific training given to intervention providers.
**6. How**	Although provision was not always described, face-to-face delivery was implied in all reports. At least 11 interventions were provided to the participant at home, and 14 appeared to have had some individual provision. One intervention also notes that some group delivery may have occurred depending on the nature of the recommendations made to the participant. Six interventions also used telephone calls to contact participants, however one of these interventions required the telephone call to be initiated by the participant or their family member. One intervention also posted out recommendations and required the participant to act on those recommendations. One intervention explicitly stated that providers were continuous for each participant, to aid in building a rapport.
**6b. How organised**	Organisation of the intervention was by a range of individuals, however in at least 12 instances this was the professional providing the intervention, be that a Nurse, Occupational Therapist, Physiotherapist, Health Visitor or other professional. Funding was described in four reports, this came from the state and in one case was supported by research funds. Although many interventions describe input of multiple professionals including GPs, four explain that decision making was to be unidisciplinary. Four interventions mentioned that the participant had to take responsibility for organising care in line with recommendations.
**7. Where**	Thirteen interventions were described as being provided in the participant's home.<br><br>Eight interventions were undertaken in Europe, three in the UK, two in Denmark, one in Switzerland, one in Belgium and one in The Netherlands. One intervention was undertaken in Australia, another in the USA and five others were carried out in Japan.
**8. When and how much**	For one intervention it was not stated when input commenced. For the other reports inclusion and exclusion criteria were wide ranging. Five interventions were to start following assessment which indicated a specific level of need. Four were specific to location but that the participants own accommodation, their GP surgery or upon their discharge from hospital. One required their participants to be registered with the welfare authority. Seven had minimum age requirements, these ranged from 60 and over to 80 and over. One required a specific level of frailty in their participants and four required a level of ADL limitation but with capacity to ambulate. Participants were excluded if they had a severe cognitive impairment or dementia in two studies and were at the end of their life in of these. One other report excluded those who had used welfare services in the preceding three months in three studies.<br><br>The nature, frequency and duration of input was varied across interventions and was not always clear. The longest visits were up to two hours in length. The longest intervention duration was three years, while the shortest stated was nine months. Input over this duration was varied, 12 visits was the most contacts a participant could expect to have though most interventions ranged between quarterly and bi-annually contacts. Other contacts included scheduled and ad hoc telephone calls. One intervention appeared to have also included ad hoc visits as required and upon request.
**9. Tailoring**	One report did not mention any tailoring to the intervention, the remaining 14 all indicated tailoring of provision was in response to the participants needs assessment. Five interventions considered the preferences and wishes of the participant, one of these also considered family input. Four interventions tailored additional contact to need. One report mentioned that participants had the right to decline recommendations.
**10. Modifications**	None of the reports described any modifications made to the intervention.
**11. How well (planned)**	Seven reports did not describe if or how they took steps to improve or measure adherence to, or the efficacy of, their intervention. Three reports described supervision, training and monitoring of providers. At least three described the role of a detailed protocol and system of working in consistent delivery by different providers. One study ensured that providers could raise questions and queries to enhance their practice and one other carried out quality assessments on the data collection process. At least two documented contacts and actions made during planning. One intervention was piloted, and one other conducted qualitative investigation alongside the trial.

**12. How well (actual)**	Seven reports did not describe how well their intervention was implemented. Four interventions examined participant compliance with visits, all four suggested this was at least 60%, one was as high as 98%, although one other study found that the number of visits per participant varied widely in their intervention. Actions and recommendations from their assessment were measured in three studies, compliance was over 50% in two studies, however for the other intervention, almost 80% of the time no recorded action was made in a visit. Consistency in provision was considered good in one study, in another a provider left and had to be replaced and in a third the intervention delivery was very varied across the two providers.

Group: Multifactorial-action and review with medication review

There are 24 interventions in this group: Bouman 2008[513], Brettschneider 2015[514], Cesari 2014[517], Challis 2004[518], Dalby 2000[524], Fabacher 1994[528], Fairhall 2015[529], Ford 1971[533], Fox 1997[534], Harari 2008[544], Hebert 2001[547], Hogg 2009[550], Kono 2016[558], Leung 2004[563], Melis 2008[573], Rubenstein 2007[595], Stuck 2000[603], Suijker 2016[605], two arms of Thomas 2007[611], Tulloch 1979[613], van Hout 2010[618], Vass 2005[622], Yamada 2003[631]

TIDieR item	Description
**1. Brief name**	
**2. Why**	Goal: Most interventions had the goal of maintaining and improving the function of older people, additional aims included improving quality of life and reducing negative outcomes. Reducing the costs associated with health resource use and long-term care admissions were also identified goals of half of the interventions. Six interventions explicitly described the importance of identification of health needs over a number of physical, psychological and social domains. Behaviour change and self-management promotion was mentioned by four reports. Other goals of interventions included reducing care-giver burden, promoting appropriate health care access and increasing human interaction within care provision.<br><br>Rationale: An ageing population and current complexity with the identification of risk, as well as the prevalence of unmet need and treatable conditions. Furthermore, variations in screening and appropriate service provision were seen as important to address by interventions. The majority of interventions claimed to be based upon previous evidence regarding the benefits of multidisciplinary screening, multidisciplinary or specialist input on outcomes for older people. More than half of the studies were based on previous study findings and/or pilot work. Home visits were believed to be key to success in at least 11 interventions. The importance of the nurse role was mentioned by two reports. Other rationalisation included the perceived benefits of behaviour change techniques and care-giver support.
**3. What (materials)**	One study did not report on the materials used. Over 20 interventions described the use of assessment tools which covered a range of domains. The delivery of these appears to have been by a healthcare professional in most cases, although one was posted to participants and their caregivers to complete. Care note access was required in nine interventions. Over half of interventions mention access to referrals. Eight interventions communicated recommendations to GPs, five to participants. Four interventions listed training for staff and five provided guidelines. Three interventions refer to the provision of resource information. Participants were provided with equipment for the monitoring of health conditions in one intervention. One intervention explicitly refers to equipment used to assess a participant for use by a healthcare professional. Recording documentation which was used by health care professionals, the research team and the participant was mentioned in six reports.
**4. What (procedures)**	All interventions involved multi-domain assessments although one was carried out as self-assessment by post; the majority were undertaken by trained healthcare staff. A range of domains were incorporated including, among others, physical health, cognition, mental health, medication and social aspects. All interventions develop some sort of care planning; nine interventions explicitly refer to consultation and agreement on this planning with the participant. Reviewing of the planning was mentioned across the interventions, however the way in which this took place was varied, sometimes with face-to-face contact at home while others placed telephone calls. Actions from the assessment and planning were often related to referrals on to other services, and/or the provision of the information and advice, be that to the participant or caregiver or other healthcare staff. Five interventions explicitly refer to the need for participants to take actions themselves. Nine describe support from others to sustain the recommendations and actions. Multidisciplinary discussion was mentioned by at least three interventions. Access to usual care was described as maintained in at least 13 interventions.
**5. Who provided**	Nurses, including those with more general and specific skill sets were the main implementers of the intervention in 17 descriptions. Geriatrician input was part of eight interventions; GPs were significant contributors to five interventions. Other professionals defined as involved included social workers and

	physicians' assistants. Multidisciplinary input was often described in the interventions as accessed when required; this would be from dietitians, physiotherapists, pharmacists and health visitors among others.
**6. How**	Intervention provision was primarily to the participant on an individual basis. Twenty-one interventions describe face-to face contact, 16 are explicitly at the participant's own home. Nine explicitly describe telephone contact with participants at some point in the process, be that initial assessment or follow-up. Two studies were conducted in clinics and two others were primarily utilising routine care note data. One was conducted through self-assessment by post. Three interventions describe the need for family or caregiver involvement.
**6b. How organised**	Organisation of the intervention was not always clear or explicitly stated. The intervention was organised by a range of individuals most frequently, in at least 14 cases, this was by a nurse. Having the input of a range of individuals was mentioned in eight reports; GP input was mentioned in twelve reports, although at times this was suggested as not required. The participant or their caregiver was expected to co-ordinate their response to the assessment in at least four interventions. Geriatricians took a lead role in organisation for three interventions.
**7. Where**	At least some of the intervention was provided to the participants at their home in 21 described studies. Health care settings including rehabilitation centres and clinics were also delivery sites in at least seven reports.<br><br>Eleven interventions were provided in European countries, including The Netherlands, the UK, Germany, France, Denmark and Switzerland. The USA and Canada were the location of a further nine interventions. Three interventions were provided in Asian locations, Hong Kong and Japan. Australia was the site of one intervention.
**8. When and how much**	Not all reports described the inclusion and exclusion criteria for involvement. Studies varied significantly on how they recruited and involved participants. Some used age as a limitation, however this varied from 50 years and over to 80 years and over. Some studies included those who had been recently discharged from hospital or were awaiting other service input. Some studies used assessment of frailty level or disability as an inclusion criterion. Some studies excluded individuals who were severely ill or living with dementia or severe cognitive impairments. Other exclusions were based on the intervention being supported by the GP or geographical limitations.<br><br>The frequency, duration and nature of input across interventions was highly variable. In some reports this was unclear. Some interventions provided a minimum of one contact at assessment only, whilst others provided a range of contacts based on need. Length of involvement in the intervention was also varied, from a minimum of seven weeks to four years; most interventions were around 1-2 years. Frequency of contact ranged from bi-weekly to annual input. Visit length was described in at least nine reports, the duration of visits being between 20 minutes and two hours. The nature of the follow up interaction was less formally described and often appeared to be tailored.
**9. Tailoring**	All intervention reports described some level of tailoring. Twenty-three interventions reflected tailoring to the needs identified for the participant during their assessment. Most of this included the number and duration of contacts. Nine interventions described collaboration with the participant, whilst four had a preference for input from caregivers or family as well. Four interventions mention contribution from GP's or Pharmacists as and when required. Flexibility about the location of the intervention delivery was also mentioned in two reports.
**10. Modifications**	Only one intervention described a required modification, this was due to a lack of equipment and the need to adjust the aim of the intervention.
**11. How well (planned)**	Approaches to measuring how well the intervention worked were described in 16 reports, the remaining eight did not have descriptions of this. To promote adherence and fidelity seven studies describe training and supervision of providers, three studies implemented follow up contact, three had used piloting work to improve the feasibility of the intervention, two used other pre-existing groups to enhance the intervention, two used family input to promote compliance, one used a small team of nurses to promote good relationships, one used goal setting approaches and a postal questionnaire included stamped addressed envelopes to promote questionnaire return. Various approaches to measuring adherence were described while around ten studies just described this generally, five mention specific documentation on assessment or follow-up visits and discussions with participants, three describe analysis of the recommendations, and three refer to the collection of barriers and facilitators. Only one report described evaluation of the intervention by the participant.
**12. How well (actual)**	Eight studies did not report on how well the interventions actually worked. This information was compiled in variable ways including a measurement of compliance. Full compliance was reported for seven studies, varying between 13% and 90%. Partial compliance was reported for eight studies, varying between 42% and 97%. Three studies reported on the number of problems identified. Two collected information on the time

	spent by nurses at visits, or the number of visits undertaken. One study reported on sustainability over time. A number of other studies described barriers to their intervention including resistance from other clinicians in three studies, a lack of motivation to change or disagreement from participants was mentioned by two studies, logistic issues in one study, feasibility perception in one study, lack of financial resources for participants to act on recommendations in one study, and variability in the provider working-style in one study. Three studies described variation in adherence to the recommendations, for example medication change had a higher adherence rate than changing smoking/alcohol use behaviours.

Group: Multifactorial-action and review with medication review and self-management strategies

There are three interventions in this group: Fox 1997[534], Phelan 2007[589], van Leeuwen 2015[619]

TIDieR item	Description
**1. Brief name**	
**2. Why**	Goal: The goals of the three interventions were broadly aligned and similar in nature. All three sought to improve health and reduce disability and poor health outcomes. One intervention was aiming to increase adherence to healthy behaviour advice. While the two others wanted to improve the quality of care and reduce hospitalisations. One of these interventions was also seeking to improve quality of life and reduce carer burden.<br><br>Rationale: Three interventions were rationalised on the premise that provision of health information may promote better self-care. Two interventions were based on evidence of success from similar approaches, one of these and one other had a behaviour change theory to ground the intervention development. One intervention was also based on policy recommendations, one on the idea that professionals trained in geriatric care were best placed to advise on supporting older people. The third suggested that there were benefits to early intervention and integrated care between various professionals and their patients.
**3. What (materials)**	There were a number of materials used in delivery of the interventions. All three interventions described care planning documentation. One also mentioned documentation to collect health history, a food and fluid diary, equipment to collect physiological data, various health advice materials and referrals. This and one other describe materials used to record meeting information. Two interventions described heath assessments in some detail relating to validation and standardisation. One of these also ensured that the patient and GP received documentation of care planning.
**4. What (procedures)**	All three interventions utilised a multidomain assessment. All three also had a follow up or review procedure, although how this was conducted varied. Two interventions provided individualised health information and advice, one of these also described risk identification, referrals and behaviour change or motivational sessions. Two interventions created action or care plans, one described how specialist input from geriatricians and geropharmacists was enacted, including family caregiver involvement. Two reports detailed access to usual care.
**5. Who provided**	All three interventions had input from nurses, though these came from various specialisms including public health and geriatrics. Two interventions involved various gerontological specialists including geriatricians, and one also involved a geropharmacist. One intervention included a primary care practitioner.
**6. How**	All three interventions were provided face-to-face and individually with additional telephone contact.
**6b. How organised**	All three interventions were organised by team members, generally led by the nurses. Patient input was required in organisation of the intervention as well. The geriatric team described were required to support with organisation for two interventions. Two reports mentioned funding, one was by the state and the other was by a large health organisation.
**7. Where**	Two interventions took place in the USA, and one in The Netherlands. Two interventions were undertaken in clinics or community hubs. The other took place at the participant's home.
**8. When and how much**	Two interventions had minimum age limits; one was aged 60 years and over, this intervention was targeted at those with lower wealth and utilising the public health service for the first time. The other intervention limited by age was open to those aged 75 and over and using a particular health organisation. The third study targeted those who were identified as frail coexistent with polypharmacy.<br><br>The nature and duration of input was somewhat varied across interventions, though all included an initial assessment. The review period was not always stated, however for one intervention this was every six months. One intervention required referrals to be enacted within three months.

**9. Tailoring**	All three interventions tailored planning in line with the needs and preferences of the participant. The frequency of follow up visits or review processes was also tailored in two interventions, specifically with an additional visit at three months on one study should this be required.
**10. Modifications**	No reports described modifications to the interventions.
**11. How well (planned)**	All three studies took some steps to increase adherence and fidelity. One intervention documented the recommendations made, and the implementation of these. One other engaged with supportive measures for providers to support with troubleshooting. The third intervention standardised processes to improve adherence, and measured implementation at the participant, provider and organisational level. In addition to this the third intervention also undertook qualitative work to identify barriers and facilitators to implementation.
**12. How well (actual)**	All three reports described how well their intervention was delivered to some extent. One found that around ¾ of participants were at least moderately adherent to the intervention recommendations, economic limits were identified as a barrier to adherence. Another intervention found almost ¾ of those invited received a visit, on average participants received two visits and six phone calls. The third intervention found that adherence for some components increased over time, while others decreased. Additionally, there was some variation in delivery between different providers. Of the providers who received the training, the motivational interview training was seen to be beneficial to practice, however the training on the assessment was not.

Group: Multifactorial-action and review with self-management strategies

There are two interventions in this group: Walters 2017[626], Wong 2019[630]

TIDieR item	Description
**1. Brief name**	
**2. Why**	Goal: Both interventions aimed to support older people to live independently through addressing health and social problems proactively.<br><br>Rationale: Evidence bases such as literature reviews and stakeholder opinions identified that multifaceted strategies would optimise self-management change. Additionally, a range of theories and approaches exist for promoting successful ageing, self-efficacy, care management and behaviour change among others.
**3. What (materials)**	A range of materials were required for the interventions. One was manualised and used a range of health educational materials, equipment for exercise and planning documentation. The other used a structured assessment, and health educational materials, promotion of self-management through identification with older celebrities, and referral systems.
**4. What (procedures)**	The interventions both used care planning to identify relevant services and referrals. Self-efficacy and behaviour change techniques were used to promote monitoring and self-care. Additionally, regular routine follow up and review as well as access to all standardised care were features of both procedures. One intervention explicitly emphasised exercise, education and environmental change (i.e., home adaptation) as part of the assessment process, but this was not provided to all participants.
**5. Who provided**	One intervention was provided by a non-specialist support worker with training in behaviour change techniques. The other intervention used intervention-trained nurse case managers and community workers under the supervision of the nurse case managers.
**6. How**	Both interventions were focused on face-to-face interaction at assessment. One intervention explicitly involved a family carer in this. Other contacts could be undertaken by remote methods such as telephone or video calling. Techniques to promote self-care were focused on self-efficacy and behaviour change approaches.
**6b. How organised**	The interventions involved the nurse or support worker organising care in conjunction with the participant, in relation to the care planning. With one intervention this was explicitly reviewed and modified as required.
**7. Where**	One intervention was undertaken in a district of Hong Kong, the other in two regions of the UK.<br><br>The intervention was carried out at home.
**8. When and how much**	With one intervention, participants were eligible for involvement if they were 60 years or more and not engaged in other health or social programmes. The other intervention recruited those who were 65 years or more and classified as mildly frail.

	The number, frequency and duration of visits differed between the interventions. Face-to-face contacts ranged from 30-120 minutes, with an expectation of a minimum of six contacts. Telephone contacts were mentioned as being 6-12 minutes long by one intervention.
**9. Tailoring**	The interventions were tailored based on the co-developed care plan which identified the participants' needs, goals and wishes. One intervention also tailored the behaviour change technique to the participant.
**10. Modifications**	Not mentioned
**11. How well (planned)**	For both interventions, fidelity and adherence were promoted through training providers in intervention delivery and recording and documenting the contact sessions with participants. One intervention also included case conference meetings, the other involved consultation with stakeholders to facilitate intervention delivery.
**12. How well (actual)**	Only one study reported on actual adherence. For this intervention, delivery was largely as intended with coverage of a range of domains and tailored goals identified. 96% of participants identified at least one goal, fidelity to the intervention at appointments was assessed at 72.1%, attendance at appointments was 91.3%.

Group: Multifactorial-action with medication review

There are five interventions in this group: Balaban 1988[506], Mann J 2021[570], Newbury 2001[582], Rockwood 2000[592], Sherman 2016[599]

TIDieR item	Description
**1. Brief name**	
**2. Why**	Goal: Although goals were varied common aims were to identify those who were at risk of having unmet needs, often including social needs, could benefit from additional care and support with a goal of improving wellbeing, reducing admissions to hospital and retaining functional independent living in the community. Some interventions utilised goal setting and tailoring approaches to improve the likelihood to success.<br><br>Rationale: Evidence indicates there is unmet need in the older population which may lead to higher level resource use. Identification of those at risk and person-centred planning may be an appropriate preventative measure in improving health outcomes for older people as well as reducing admissions to hospital.
**3. What (materials)**	Materials required were not mentioned in one study. However, for the remaining four a range of approaches were used to undertake assessments. Some assessments were completed using routinely collected data, all involved Nurse or clinician visits to carry out a physical assessment and questionnaires. The process was usually documented in participants' patient records and relevant prescriptions and referrals to services were made. One intervention used goal setting as part of the process.
**4. What (procedures)**	Procedures were different across the interventions although all carried out an assessment of needs, usually this was explicitly undertaken at the participant's home, this was primarily focused upon medical and social needs, however a psychosocial and functional approach was taken with one assessment. Assessments were usually undertaken by Nurses, sometimes with multidisciplinary input as well. Medication checks were included in all five assessments. One intervention took a person-centred approach and explicitly incorporated the wishes of the participant, another intervention also undertook goal setting at assessment. The provision of the recommended care was sometimes the role of the participants own GP, other times this was provided as part of the intervention. In one intervention it was unclear who would act on recommendations made. Follows were mentioned as part of two intervention procedures.
**5. Who provided**	Primarily interventions were provided by nurses, some of whom were specialised in geriatric care. Two interventions involved geriatricians in the assessment phase. One used a programme physician.<br><br>One intervention explicitly referred to the involvement of physiotherapists, Occupational Therapists, Social Workers, Dietitians, Audiologists and Speech and Language Therapists as part of the care carried out following assessment. Other interventions relied on GPs to enact required care.
**6. How**	Only one study mentions how participants were initially contact this was by letter and telephone. All studies refer to contact with clinicians, for most interventions this was at the participant's home and presumably therefore was face-to-face.
**6b. How organised**	In four interventions there was significant nurse input. Although with one study it was not clear who was in charge of the care planning process, this was usually undertaken by a nurse, with support from physicians or

	specialist geriatricians in two studies. The recommendations were at times carried out as part of the intervention and other times were sent to the participant's GP.
**7. Where**	A range of international locations were involved, including Australia, Sweden and Canada.<br><br>Interaction with participants was usually at home or in a primary care facility.
**8. When and how much**	Identification of participants varied. Three studies involved people based on age, those 75 and over for two of these and the other involved those aged 70 plus, or 50 plus who appeared at risk due to physical and/or social needs limiting their access to services or increasing risk of ill health. The two remaining interventions were access based on their risk of decline related to health or social illbeing.<br><br>All studies had a minimum of one visit, the remaining contact was based on needs identified.
**9. Tailoring**	All reports mention some elements of tailoring, given that assessments were aiming to identify specific needs. The need for follow up care and recommendations were mentioned as tailored in four of the interventions. The timing and location of assessment (and if necessary, the follow up) was also mentioned as flexible in three reports.
**10. Modifications**	Only one intervention mentioned modifications - the nature of these was not specified.
**11. How well (planned)**	Three reports explicitly refer to training to enhance fidelity, additionally two of these also used reliability checks on the assessments made. One intervention had also been part of a feasibility pilot.
**12. How well (actual)**	Studies varied in reporting how well the intervention worked. Two made no reference to implementation effectiveness. One stated that on average participants who were able received on average 2.0-3.8 visits. Another reported on inter-rater reliability of assessments being between 0.79-0.94 across assessors. One other intervention was reported as carried out as planned and the process was straightforward.

Group: Nutrition and exercise

There are three interventions in this group: Loh 2015[568], Serra-Prat 2017[597], van Dongen 2020[616]

TIDieR item	Description
**1. Brief name**	
**2. Why**	Goal: The long-term aim of the three interventions was to improve frailty status, physical functioning and/or reduce loss of independence. For one intervention good oral care was implicated.<br><br>Rationale: Previous research implicates muscle wastage as a contributing factor to frailty; insufficient or poor diet also contributes to this health state. Evidence supports the use of multicomponent nutritional and exercise programme in enhancing physical functioning.
**3. What (materials)**	The interventions primarily used a combination of leaflets and educational information such as DVDs describing or promoting physical exercise and providing nutritional advice. In addition to this some interventions provided referrals to nutritional units, training sessions and checklists relating to physical exercise and nutritional exercise undertaken, and oral care advice. One intervention provided cash rewards for involvement in sessions.
**4. What (procedures)**	A range of processes were seen across the interventions. Screening and identification of particular risk was seen in one of the interventions. All interventions involved an exercise session with provision of an exercise programme to be undertaken at home, one of the interventions provided a more tailored programme. Nutritional advice provision was more varied, involving screening and referral, workshops and a tailored diet provision or group advice sessions. Interventions provided a range of other activities designed to promote adherence including phone calls, training and support for healthcare professionals, goal setting and peer engagement.
**5. Who provided**	Who provided the intervention was not always clear. When stated, a range of healthcare professionals were seen to be involved. Nutritional advice was provided by dietitians or nutritionists, physical activity training was provided by physiotherapists or trained fitness instructors. Other professionals, including health promotion employees were involved to facilitate involvement.
**6. How**	Although not always clear in the reporting, physical training and nutritional exercise appears to have been provided face-to-face. Some of these sessions were group or workshop based. Some sessions had motivational techniques built in. Additional educational supplements were supplied. Telephone calls were provided to enhance adherence and for additional consultation purposes.

**6b. How organised**	This was either not mentioned or somewhat unclear in reporting for two interventions, suggesting involvement by various disciplines in executing relevant aspects such as the nutritional assessment, overseen by the research nurse. One intervention was partially coordinated by care sport collaborators who connected primary care services and the sports sector.
**7. Where**	The interventions were carried out in Spain, The Netherlands and Malaysia. In some reports there is little detail about the locations of the intervention, it is suggested that primary care centres were used. Two of the interventions detailed either the use of local sports settings and/or community facilities.
**8. When and how much**	Only two interventions provide details relating to eligibility, one intervention was aimed at those 60 and over, the other stipulated 70 and over with prefrailty.<br><br>There was variation in the number frequency, duration and nature of contact across the interventions. The exercise component varied in input from one session with recommendation to follow an exercise plan at home, to 24 weeks of sessions which decreased from hourly bi-weekly sessions to weekly sessions. The nutritional component varied from input only upon referral to 6, 30-minute sessions. The intervention which provided oral care advice included 2 sessions.
**9. Tailoring**	This was not always reported upon. One intervention provided referral to dietary services if their nutritional assessment showed a risk. One intervention provided tailoring to all components including tailored exercise programmes and dietary advice. Additionally, participants could choose to attend additional activities that were offered.
**10. Modifications**	Not mentioned in any of the reports
**11. How well (planned)**	The reporting of this varied across interventions. From a planned process evaluation to detail attendance, satisfaction, enablers and barriers to involvement, to attendance records for physical activity and dietary intake and/or telephone contact to monitor compliance.
**12. How well (actual)**	This was not reported for all interventions. For one study 47.5% were considered to have adhered at 12 month follow up. One intervention found that attendance was high at intensive support sessions (first 12 weeks), between 98.8% and 83.6%, but lower at (later) moderate support sessions, between 59.8% and 56.1%. Protein intake improved from baseline to 12 weeks and still remained higher than baseline at 24 weeks follow up.

Group: Risk-screening

There are six interventions in this group: two arms of Bleijenberg 2016[509], Carpenter 1990[516], Jitapunkul 1998[555], Kerse 2014[556], Pathy 1992[588]

TIDieR item	Description
**1. Brief name**	
**2. Why**	Goal: to preserve daily functioning and enhance their quality of life and maintain community living. Two interventions clearly mention the identification of those at risk of decline or with unmet needs<br><br>Rationale: based on evidence that there are older people living with unmet needs and identification of those at risk and with unmet needs through appropriate screening targeted action planning can be achieved in other similar programmes and pilot work.
**3. What (materials)**	A range of screening assessments or electronic patient records were used in the identification of risk and unmet needs including frailty measures, at least one of these assessments was explicitly by postal self-report.<br><br>Guidelines on the appropriate prescription of aids, medication or referral to health and social services following assessment varied across the interventions.
**4. What (procedures)**	Identification through the screening of patient records or using questionnaires and assessments either delivered by a range of individuals, from volunteers to trained health care professionals, for at least one intervention this was undertaken through self-assessment by the older person.<br><br>Identification of those deemed at risk or with unmet need resulted in a protocol to be enacted for accessing appropriate care. This was usually through needs based tailored referrals to health and social care services, prescriptions and access to aids.
**5. Who provided**	Screening assessments were undertaken by a range of individuals from volunteers to nurses, non-professionals and trained staff. One study mentioned interpretation of the screening assessment by a trained

	nurse. All but one intervention detailed that the reports generated were to be acted upon by the participant's GP or a geriatrician. Other health and social care professionals were to be involved with enactment of care as was relevant.
**6. How**	For one intervention this was not described. For at least three interventions the initial assessment is done at distance, usually by post. One described an at home face-to-face assessment. The follow up of any required care based on the screening assessments was explicitly to be undertaken individually and at home by relevant clinicians in two interventions. Location and type of follow-up care is less clear in two further interventions, and not mentioned in the remaining two.
**6b. How organised**	Organisation of the screening process was by a range of individuals or was unstated for some of the interventions. Organisation of the subsequent care was often undertaken by the participants GP or members of the GP practice such as health visitors or nurses. The interventions varied as to whether the care was unidisciplinary or multidisciplinary. One intervention explicitly refers to organisation by the research team and facilitation by the district health board.
**7. Where**	Four of the interventions were based in Europe, two in the UK, two in The Netherlands. One was in New Zealand and one in Thailand.<br><br>Two interventions do not specify a location for carrying out the intervention, one states that some assessment will be carried out at home, three others mention that the intervention is based in the participant's home.
**8. When and how much**	Eligibility for intervention involvement varied across studies, one did not mention a minimum age, two recruited at 60 years and over with an indication of multimorbidity, polypharmacy or lack of contact with services, one recruited at 65 and over, one at 75 and over, whilst another recruited at varying ages depending on ethnicity.<br><br>Repetition of the screening process was mentioned as being annual in one study or every three years in another.<br><br>Input from services according to need varied, four interventions explicitly mention follow up support being needs based, ranging from a minimum of one visit to quarterly visits for three years to as required.
**9. Tailoring**	One intervention did not mention any tailoring. The remaining five mention tailoring based on the assessments undertaken. Additional tailoring to the specific needs and required input by service for individuals including the nature and frequency of follow up visits and contact was also mentioned.
**10. Modifications**	Only one report describes modifications which were required due to reforms to geriatric services
**11. How well (planned)**	Only two reports describe approaches to maintain fidelity. One refers to use of manualised training of the staff involved, the other refers to use of manualised training and supervision of staff, collection of information on barriers and facilitators of the intervention and the undertaking of a 6 week pilot study.
**12. How well (actual)**	This was not mentioned by two of the reports. The feasibility of the interventions was variable and information relating to this differs. One intervention, while perceived as feasible by staff, only managed to deliver follow up care to a third of those assessed as in need or at risk. Referral rates in the study group exceeded the control group until the final year of the study for one intervention. One study saw assessment completion and return rates of 88%. Another study reported that 40% of those screened were not in need of visits.

Appendix 11. Results of network meta-analyses

11.1 Summary of network meta-analyses

Table 4 - Summary of network meta-analyses

Outcome	Reference comparator	Timeframe	Analysis	Total studies	Total nodes	Total participants	Heterogeneity (τ)	Wald test (p)	Node splitting (p)
Living at home	Available care	Short	Main	8	8	4013	inestimable	0.24	all > 0.05
〃	〃	Medium	Main	21	14	16,937	8.56×10^{-2}	0.80	all > 0.05
〃	〃	〃	Sensitivity (RoB)	17	11	15,457	7.71×10^{-2}	0.51	all > 0.05
〃	〃	〃	Frailty meta-regression	18	12	13,418	3.86×10^{-7}	0.70	all > 0.05
〃	〃	Long	Main	13	10	14,843	9.92×10^{-7}	not tested	not tested
〃	Homecare	Short	Main	4	4	704	2.85×10^{-7}	not tested	not tested
〃	〃	Medium	Main	5	6	1978	inestimable	not tested	not tested
〃	〃	〃	Sensitivity (RoB)	4	5	1234	inestimable	not tested	not tested
IADL	Available care	Short	Main	6	7	1155	inestimable	not tested	not tested
〃	〃	Medium	Main	16	14	5309	6.91×10^{-8}	not tested	not tested
〃	〃	〃	Sensitivity (RoB)	11	9	3369	1.39×10^{-8}	not tested	not tested
〃	〃	〃	Frailty meta-regression	15	13	4997	1.22×10^{-7}	not tested	not tested
〃	〃	Long	Main	6	5	1727	5.31×10^{-6}	not tested	not tested
〃	Homecare	Short	Main	4	5	970	inestimable	not tested	not tested
〃	〃	Medium	Main	6	5	1401	1.41×10^{-1}	not tested	not tested
PADL	Available care	Short	Main	8	9	4075	inestimable	not tested	not tested
〃	〃	Medium	Main	20	16	8583	3.10×10^{-1}	0.47	all > 0.05
〃	〃	〃	Sensitivity (RoB)	14	11	3613	3.35×10^{-1}	not tested	not tested
〃	〃	〃	Frailty meta-regression	18	15	8182	6.64×10^{-7}	0.99	all > 0.05
〃	〃	Long	Main	7	7	2095	3.87×10^{-8}	0.74	all > 0.05
〃	Homecare	Short	Main	4	5	775	inestimable	0.90	all > 0.05
〃	〃	Medium	Main	4	5	632	inestimable	0.41	all > 0.05
〃	〃	〃	Sensitivity (RoB)	3	4	368	inestimable	0.78	all > 0.05
Hospitalisation	Available care	Medium	Main	15	10	9569	1.57×10^{-6}	0.38	all > 0.05
〃	〃	〃	Sensitivity (RoB)	11	8	6896	1.94×10^{-5}	0.30	all > 0.05
〃	〃	〃	Frailty meta-regression	13	8	6314	1.58×10^{-7}	0.79	all > 0.05
Care-home placement	Available care	Short	Main	7	8	3672	inestimable	not tested	not tested
〃	〃	Medium	Main	20	14	16,055	2.44×10^{-1}	0.82	all > 0.05
〃	〃	〃	Sensitivity (RoB)	15	11	12,326	3.15×10^{-1}	0.81	all > 0.05
〃	〃	〃	Frailty meta-regression	17	12	12,648	2.05×10^{-5}	0.88	all > 0.05
〃	〃	Long	Main	14	10	13,638	5.18×10^{-6}	not tested	not tested
〃	Homecare	Medium	Main	4	5	1567	inestimable	not tested	not tested
Health status	Available care	Medium	Main	8	7	2631	9.95×10^{-2}	not tested	not tested
〃	〃	〃	Sensitivity (RoB)	7	6	1787	9.95×10^{-2}	not tested	not tested
〃	〃	〃	Frailty meta-regression	8	7	2631	3.16×10^{-2}	not tested	not tested

Outcome	Reference comparator	Timeframe	Analysis	Total studies	Total nodes	Total participants	Heterogeneity (τ)	Wald test (p)	Node splitting (p)
Depression	Available care	Medium	Main	15	13	7245	1.05×10^{-1}	0.16	all > 0.05
〃	〃	〃	Sensitivity (RoB)	10	9	2893	8.64×10^{-6}	0.66	all > 0.05
〃	〃	〃	Frailty meta-regression	13	12	7046	5.42×10^{-3}	0.99	all > 0.05
〃	Homecare	Medium	Main	6	7	996	inestimable	0.25	all > 0.05
〃	〃	〃	Sensitivity (RoB)	3	4	368	inestimable	0.79	all > 0.05
Mortality	Available care†	Medium	Main	65	41	38,351	9.53×10^{-2}	0.30	all > 0.05
〃	〃	〃	Sensitivity (RoB)	46	26	30,425	1.08×10^{-1}	0.39	all > 0.05
〃	〃 †	〃	Frailty meta-regression	55	37	32,272	1.49×10^{-1}	0.20	all > 0.05
〃	Homecare	Medium	Sensitivity (RoB)	11	10	2479	3.23×10^{-7}	0.91	all > 0.05

IADL: instrumental activities of daily living. PADL: personal activities of daily living. Reference comparator was either available care or homecare as the network was split for all outcome-timeframe combinations except mortality in the medium term. Timeframes:- short: 24 weeks to 9 months; medium: >9 months to 18 months; long: >18 months. Heterogeneity (τ) was inestimable when a network contained only a single study measuring each comparison, and therefore a common-effect model was fitted. Tests of the consistency assumption (Wald test and node splitting) could not be tested when the network contained no loops; a 'consistency' model was fitted, setting the inconsistency parameter in the model to zero for all comparisons.

† For mortality in the medium term, homecare studies were not disconnected from the available care network in the main analysis and frailty meta-regression, so all were compared with available care. Therefore there is not a separate homecare network for either of these analyses. The sensitivity analysis for risk of bias removed the linking comparison and therefore we conducted separate available care and homecare analyses.

11.2 Living at home

11.2.1 Living at home available care network, short-term timeframe

Table 5 - Short-term living at home available-care network

Study	Frailty	n	Experimental group	Control group	ROB D1	D2	D3	D4	D5	Overall
**Challis 2004**[518]	frail	256	mfar(w/med)	mfar	-	-	+	+	+	-
**Imhof 2012**[553]	all	448	mfar	ac	-	-	+	+	-	-
**Kukkonen-Harjula 2017**[561]	pre-frail and frail	292	ADL & ntr & exrc	ac	+	-	+	+	-	-
**Liddle 1996**[566]	unclassifiable	105	aids & mfar	ac	-	x	+	+	-	xx
**Metzelthin 2013**[576]	frail	341	educ & mfar(w/med+slfm)	ac	-/-	-	+	+	-	-
**Suijker 2016**[605]	frail	2031	mfar(w/med)	ac	+/-	-	-	+	-	-
**Szanton 2011**[606]	pre-frail and frail	39	ADL&aids&educ&exrc& mfar(w/med+slfm)	ac	-	-	+	+	-	-
**Wong 2019**[630]	all	501	mfar(w/slfm)	ac	x	-	-	+	-	x

Table 6 - Living at home in the short term: comparisons with available care summary of findings table.

Population: Older people

Interventions: Community-based complex interventions

Comparator: Available care (ac)

Outcome: living at home

Timeframe: short term; range of follow up 24 weeks to 6 months

Setting: Community

Total studies: 8

Total participants: 4013

Comparator rank: Mean 4.7, 95% CI 2 to 7

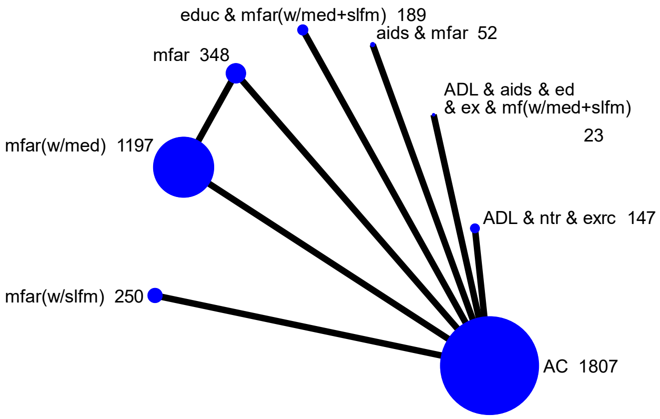

	Relative effect (95% CI)		Anticipated absolute effect (95% CI)						
			High-risk population (952 per 1000 with ac)		Low-risk population (980 per 1000 with ac)				
Intervention group	Network summary estimate	Calculated risk ratio[a]	With intervention	Difference	With intervention	Difference	Certainty of the evidence (GRADE)	Ranking (95% CI)	Interpretation
Multifactorial-action and review (mfar)	OR 1.34 (0.75 to 2.39) Mixed estimate	RR 1.01 (0.99 to 1.02)	964 per 1000 (937 to 979)	12 more per 1000 (15 fewer to 27 more)	985 per 1000 (974 to 992)	5 more per 1000 (6 fewer to 12 more)	⊕⊕⊖⊖ Low[b]	3.1 (1 to 6)	may result in a very slight increase in chance of living at home
ADL, nutrition and exercise (ADL & ntr & exrc)	OR 1.01 (0.25 to 4.13) Mixed estimate	RR 1.00 (0.92 to 1.02)	953 per 1000 (831 to 988)	1 more per 1000 (121 fewer to 36 more)	980 per 1000 (924 to 995)	0 per 1000 (56 fewer to 15 more)	⊕⊕⊖⊖ Low[b]	4.5 (1 to 8)	may result in little to no difference in chance of living at home
Multifactorial-action and review with medication-review (mfar(w/med))	OR 0.95 (0.58 to 1.55) Mixed estimate	RR 1.00 (0.98 to 1.01)	949 per 1000 (920 to 969)	3 fewer per 1000 (32 fewer to 17 more)	979 per 1000 (966 to 987)	1 fewer per 1000 (14 fewer to 7 more)	⊕⊕⊖⊖ Low[b]	5.0 (2 to 7)	may result in little to no difference in chance of living at home
Education, multifactorial-action and review with medication-review and self-management strategies (educ & mfar(w/med+slfm))	OR 0.52 (0.13 to 2.06) Mixed estimate	RR 0.98 (0.85 to 1.01)	912 per 1000 (725 to 976)	40 fewer per 1000 (227 fewer to 24 more)	962 per 1000 (867 to 990)	18 fewer per 1000 (113 fewer to 10 more)	⊕⊕⊖⊖ Low[b]	6.2 (2 to 8)	may result in a reduction in chance of living at home
Aids, multifactorial-action and review (aids & mfar)	OR 3.06 (0.31 to 30.42) Mixed estimate	RR 1.02 (0.94 to 1.03)	984 per 1000 (859 to 998)	32 more per 1000 (93 fewer to 46 more)	993 per 1000 (938 to 999)	13 more per 1000 (42 fewer to 19 more)	⊕⊖⊖⊖ Very low[b,c]	2.2 (1 to 7)	the evidence is very uncertain about the effect on chance of living at home
Multifactorial-action and review with self-management strategies (mfar(w/slfm))	OR 1.34 (0.56 to 3.25) Mixed estimate	RR 1.01 (0.98 to 1.02)	964 per 1000 (917 to 985)	12 more per 1000 (35 fewer to 33 more)	985 per 1000 (965 to 994)	5 more per 1000 (15 fewer to 14 more)	⊕⊖⊖⊖ Very low[b,d]	3.4 (1 to 7)	the evidence is very uncertain about the effect on chance of living at home

ADL, aids, education, exercise, multifactorial-action and review with medication-review and self-management strategies (ADL & aids & ed & ex & mf(w/med+slfm))	OR 0.18 (0.01 to 3.69) Mixed estimate	RR 0.89 (0.24 to 1.02)	779 per 1000 (145 to 987)	173 fewer per 1000 (807 fewer to 35 more)	897 per 1000 (295 to 994)	83 fewer per 1000 (685 fewer to 14 more)	⊕⊖⊖⊖ Very low[e]	7.0 (1 to 8)	the evidence is very uncertain about the effect on chance of living at home

a: Calculated from OR and an assumed comparator risk of 0.972, the median available care risk among these studies.

b: very serious concerns about imprecision as confidence interval includes substantial benefit and substantial harm. Downgrade twice.

c: very serious concerns about risk of bias due to significant contamination in both groups, particularly in the intervention arm where serious deviations from the intended interventions happened. Already downgraded twice for imprecision, therefore downgrade once.

d: serious concerns about risk of bias due to randomisation process. Downgrade once.

e: extremely serious concerns about imprecision as confidence interval is extremely wide. Downgrade three levels.

Table 7 - Results of living at home: short-term available care network

**mfar(w/slfm)**							1.34 (0.56,3.25)
1.42 (0.52,3.90)	**mfar(w/med)**	0.62 (0.38,1.02)					1.11 (0.63,1.95)
1.00 (0.35,2.89)	0.71 (0.45,1.11)	**mfar**					0.87 (0.35,2.19)
2.57 (0.50,13.10)	1.81 (0.42,7.76)	2.56 (0.58,11.33)	**educ & mfar(w/med+slfm)**				0.52 (0.13,2.06)
0.44 (0.04,5.14)	0.31 (0.03,3.24)	0.44 (0.04,4.68)	0.17 (0.01,2.48)	**aids & mfar**			3.06 (0.31,30.42)
7.58 (0.32,178.38)	5.34 (0.25,115.36)	7.55 (0.34,165.59)	2.95 (0.11,82.25)	17.24 (0.38,774.20)	**ADL&aids&ed&ex&mf(w/med+slfm)**		0.18 (0.01,3.69)
1.33 (0.25,6.97)	0.93 (0.21,4.14)	1.32 (0.29,6.04)	0.52 (0.07,3.67)	3.02 (0.20,44.55)	0.18 (0.01,4.95)	**ADL & ntr & exrc**	1.01 (0.25,4.13)
1.34 (0.56,3.25)	0.95 (0.58,1.55)	1.34 (0.75,2.39)	0.52 (0.13,2.06)	3.06 (0.31,30.42)	0.18 (0.01,3.69)	1.01 (0.25,4.13)	**ac**

Lower left triangle presents the findings (OR with 95% CI) of the network meta-analysis. Upper right triangle presents the findings (OR with 95% CI) of pairwise meta-analyses. An OR>1 favours the upper left intervention; an OR<1 favours the lower right intervention. Within the table, comparisons between treatments should be read from left to right (i.e. treatment 1 versus treatment 2). The estimate effect measure (OR and their 95% CI) is in the cell in common between the row- and column-defining treatment.

Table 8 - Intervention rankings for living at home: short-term available care network

Treatment	SUCRA	Pr(Best)	Mean Rank	LCI Rank	UCI Rank
aids & mfar	82.8	63.9	2.2	1	7
mfar	70.3	9.2	3.1	1	6
mfar(w/slfm)	66.0	13.0	3.4	1	7
adl & ntr & exrc	50.5	8.2	4.5	1	8
ac	47.7	0.4	4.7	2	7
mfar(w/med)	43.5	0.2	5.0	2	7
educ & mfar(w/med+slfm)	25.5	1.4	6.2	2	8
adl&aids&ed&ex&mf(w/med+slfm)	13.6	3.7	7.0	1	8

SUCRA values (0–100) and mean ranks are presented, based on 1000 simulations. Higher SUCRAs and lower mean ranks indicate better performing interventions. Pr(Best) gives the probability of each specific intervention being ranked best intervention, based on 1000 simulations.

11.2.2 Living at home available care network, medium-term timeframe

Table 9 - Medium-term living at home available-care network

Study	Frailty	n	Experimental group	Control group	ROB D1	D2	D3	D4	D5	Overall
**Blom 2016[510]**	all	1105	mfa-(w/med+slfm)	ac	x/+	-	x	+	-	xx
**Dalby 2000[524]**	frail	139	mfar(w/med)	ac	-	-	+	+	+	-
**Fabacher 1994[528]**	all	229	mfar(w/med)	ac	-	-	x	+	+	x
**Hall 1992[543]**	frail	167	hmcr & mfar(w/slfm)	hmcr & mfar	-	-	+	+	+	-
**Harari 2008[544]**	all	2425	mfar(w/med)	ac	+	x	+	+	-	x
**Hay 1998[546]**	unclassifiable	485	mfa-	ac	-	-	x	+	-	x
**Hebert 2001[547]**	pre-frail and frail	494	mfar(w/med)	ac	-	-	+	+	-	-
**Henderson 2005[548]**	robust	136	mfar	ac	+/x	+	x	+	-	xx
**Kerse 2014[556]**	pre-frail and frail	3712	rsk-mfa-	ac	+/+	-	+	+	-	-
**Kono 2004[559]**	pre-frail and frail	117	mfar	ac	-	-	+	+	-	-
**Kono 2016[558]**	pre-frail	313	mfar(w/med)	mfar	+	-	-	+	+	-
**Kukkonen-Harjula 2017[561]**	pre-frail and frail	287	ADL & ntr & exrc	ac	+	-	+	+	-	-
**Metzelthin 2013[576]**	frail	325	educ & mfar(w/med+slfm)	ac	-/-	-	-	+	-	-
**Monteserin Nadal 2008[578]**	all	516	educ & rsk-mfa-	ac	-	-	x	+	+	x
**Newbury 2001[582]**	unclassifiable	100	mfa-(w/med)	ac	-	-	+	+	-	-
**Newcomer 2004[583]**	unclassifiable	2934	educ & mfar(w/med)	ac	-	-	+	+	-	-
**Ploeg 2010[590]**	pre-frail and frail	665	educ & mfar(w/med)	ac	+	-	x	+	-	x
**Romera-Liebana 2018[593]**	pre-frail and frail	342	cgn & med & ntr & exrc	ac	+	-	+	+	-	-
**Shapiro 2002[598]**	frail	72	hmcr & mfar	ac	-	x	x	+	-	xx
**Suijker 2016[605]**	frail	1873	mfar(w/med)	ac	+/-	-	-	+	-	-
**van Hout 2010[618]**	frail	501	mfar(w/med)	ac	+	-	x	+	-	x

n: number of participants. ROB: risk of bias. D#: Domain #. D1: risk of bias arising from the randomisation process (individual); or, for cluster trials, risk of bias arising from the randomisation process / risk of bias arising from the identification or recruitment of participants into clusters. D2: risk of bias due to deviations from the intended interventions (effect of assignment to the intervention). D3: risk of bias due to missing outcome data. D4: risk of bias in measurement of the outcome. D5: risk of bias in selection of the reported result. +: low risk of bias; -: some concerns; x: high risk of bias / serious concerns; xx: very serious concerns (overall risk of bias only). all: robust, pre-frail and frail.

Table 10 - Results of living at home: medium-term available care network

**rsk-mfa-**													0.90 (0.70,1.17)
0.74 (0.49,1.11)	**mfar(w/med)**	1.22 (0.36, 4.07)											1.22 (0.96, 1.56)
0.79 (0.39,1.60)	1.06 (0.55,2.06)	**mfar**											1.18 (0.50, 2.79)
0.90 (0.52,1.57)	1.22 (0.72,2.07)	1.15 (0.52,2.53)	**mfa-(w/med+slfm)**										1.00 (0.65, 1.53)
0.35 (0.08,1.52)	0.48 (0.11,2.04)	0.45 (0.09,2.15)	0.39 (0.09,1.75)	**mfa-(w/med)**									2.55 (0.62, 10.49)
0.41 (0.11,1.49)	0.55 (0.15,2.00)	0.52 (0.12,2.13)	0.45 (0.12,1.73)	1.14 (0.17,7.69)	**mfa-**								2.23 (0.64, 7.82)
0.10 (0.02,0.49)	0.14 (0.03,0.66)	0.13 (0.02,0.69)	0.11 (0.02,0.56)	0.29 (0.04,2.34)	0.25 (0.03,1.85)	**hmcr & mfar(w/slfm)**	1.56 (0.63, 3.82)						
0.16 (0.04,0.57)	0.21 (0.06,0.76)	0.20 (0.05,0.81)	0.18 (0.05,0.66)	0.45 (0.07,2.96)	0.39 (0.07,2.30)	1.56 (0.62,3.88)	**hmcr & mfar**						5.71 (1.67, 19.60)
0.83 (0.42,1.63)	1.12 (0.58,2.17)	1.05 (0.43,2.54)	0.92 (0.43,1.96)	2.33 (0.50,10.98)	2.04 (0.50,8.31)	8.13 (1.55,42.75)	5.23 (1.31,20.88)	**educ & rsk-mfa-**					1.09 (0.61, 1.96)
2.22 (0.74,6.69)	3.00 (1.01,8.93)	2.82 (0.82,9.73)	2.46 (0.78,7.79)	6.26 (1.06,36.96)	5.48 (1.05,28.53)	21.84 (3.36,141.97)	14.04 (2.74,71.90)	2.68 (0.79,9.10)	**educ & mfar(w/med+slfm)**				0.41 (0.14, 1.16)
1.03 (0.63,1.68)	1.39 (0.85,2.27)	1.31 (0.61,2.79)	1.14 (0.63,2.07)	2.90 (0.66,12.69)	2.54 (0.68,9.53)	10.12 (2.06,49.65)	6.51 (1.77,23.91)	1.24 (0.61,2.55)	0.46 (0.15,1.43)	**educ & mfar(w/med)**			0.93 (0.55, 1.58)
0.47 (0.18,1.21)	0.63 (0.25,1.61)	0.59 (0.20,1.79)	0.52 (0.19,1.42)	1.32 (0.24,7.11)	1.15 (0.24,5.45)	4.59 (0.77,27.46)	2.95 (0.64,13.73)	0.56 (0.19,1.67)	0.21 (0.05,0.85)	0.45 (0.17,1.21)	**cgn & med & ntr & exrc**		1.93 (0.80, 4.69)
0.50 (0.18,1.40)	0.68 (0.25,1.87)	0.64 (0.20,2.06)	0.56 (0.19,1.64)	1.42 (0.25,8.00)	1.24 (0.25,6.15)	4.96 (0.80,30.82)	3.19 (0.66,15.50)	0.61 (0.19,1.93)	0.23 (0.05,0.96)	0.49 (0.17,1.40)	1.08 (0.29,4.08)	**ADL & ntr & exrc**	1.79 (0.68, 4.69)
0.90 (0.66,1.23)	1.22 (0.93,1.59)	1.15 (0.60,2.18)	1.00 (0.63,1.58)	2.55 (0.61,10.60)	2.23 (0.63,7.91)	8.89 (1.90,41.63)	5.71 (1.65,19.83)	1.09 (0.60,2.01)	0.41 (0.14,1.17)	0.88 (0.60,1.29)	1.93 (0.79,4.77)	1.79 (0.67,4.76)	**ac**

Lower left triangle presents the findings (OR with 95% CI) of the network meta-analysis. Upper right triangle presents the findings (OR with 95% CI) of pairwise meta-analyses. A OR>1 favours the upper left intervention; a OR<1 favours the lower right intervention. Within the table, comparisons between treatments should be read from left to right (i.e. treatment 1 versus treatment 2). The estimate effect measure (OR and their 95% CI) is in the cell in common between the row- and column-defining treatment.

Table 11 - Intervention rankings for living at home: medium-term available care network

Treatment	SUCRA	Pr(Best)	Mean Rank	LCI Rank	UCI Rank
hmcr & mfar(w/slfm)	95.8	70.5	1.5	1	4
hmcr & mfar	89.1	12.5	2.4	1	5
mfa-(w/med)	72.1	8.7	4.6	1	13
mfa-	69.7	5.7	4.9	1	13
cgn & med & ntr & exrc	66.6	0.6	5.3	2	12
ADL & ntr & exrc	62.5	1.9	5.9	2	13
mfar(w/med)	49.7	0.0	7.5	5	11
mfar	42.7	0.1	8.4	4	13
educ & rsk-mfa-	39.1	0.0	8.9	4	13
mfa-(w/med+slfm)	32.5	0.0	9.8	5	13
ac	31.8	0.0	9.9	7	12
rsk-mfa-	22.6	0.0	11.1	7	13
educ & mfar(w/med)	21.9	0.0	11.2	6	14
educ & mfar(w/med+slfm)	3.9	0.0	13.5	8	14

SUCRA values (0–100) and mean ranks are presented, based on 1000 simulations. Higher SUCRAs and lower mean ranks indicate better performing interventions. Pr(Best) gives the probability of each specific intervention being ranked best intervention, based on 1000 simulations.

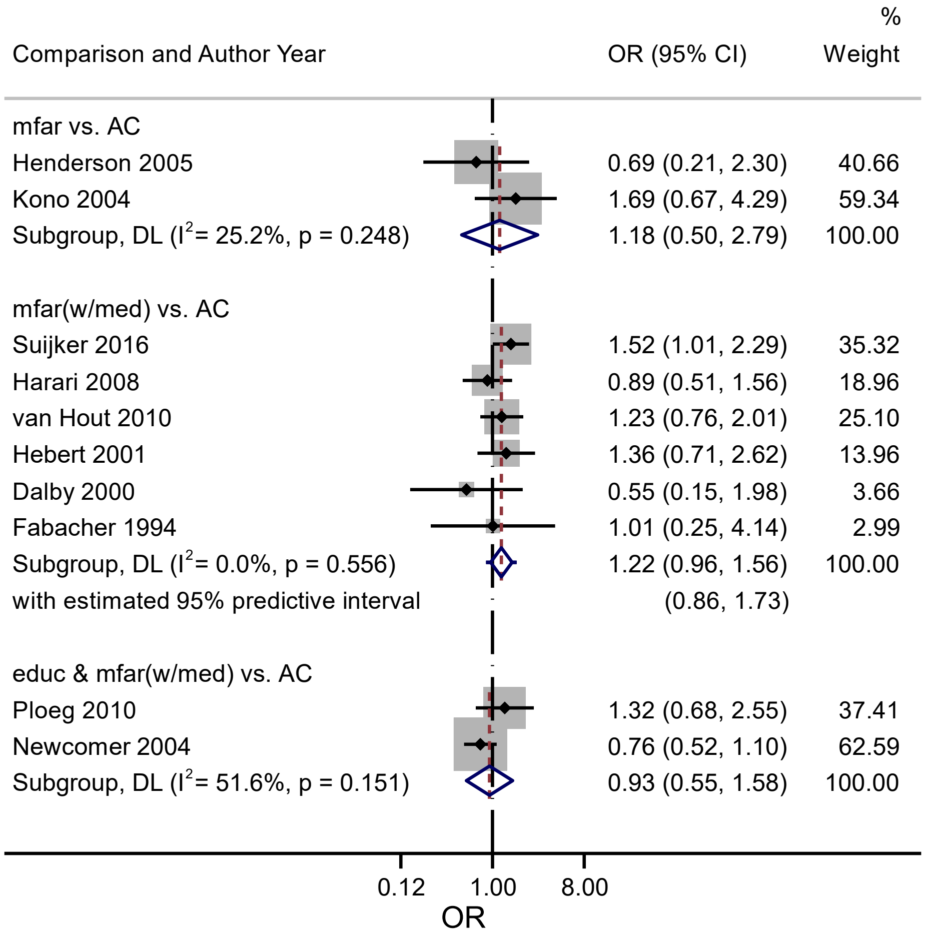

Figure 3 - Pairwise meta-analysis for living at home: medium-term available care network (pooling comparisons with greater than one study reporting results)

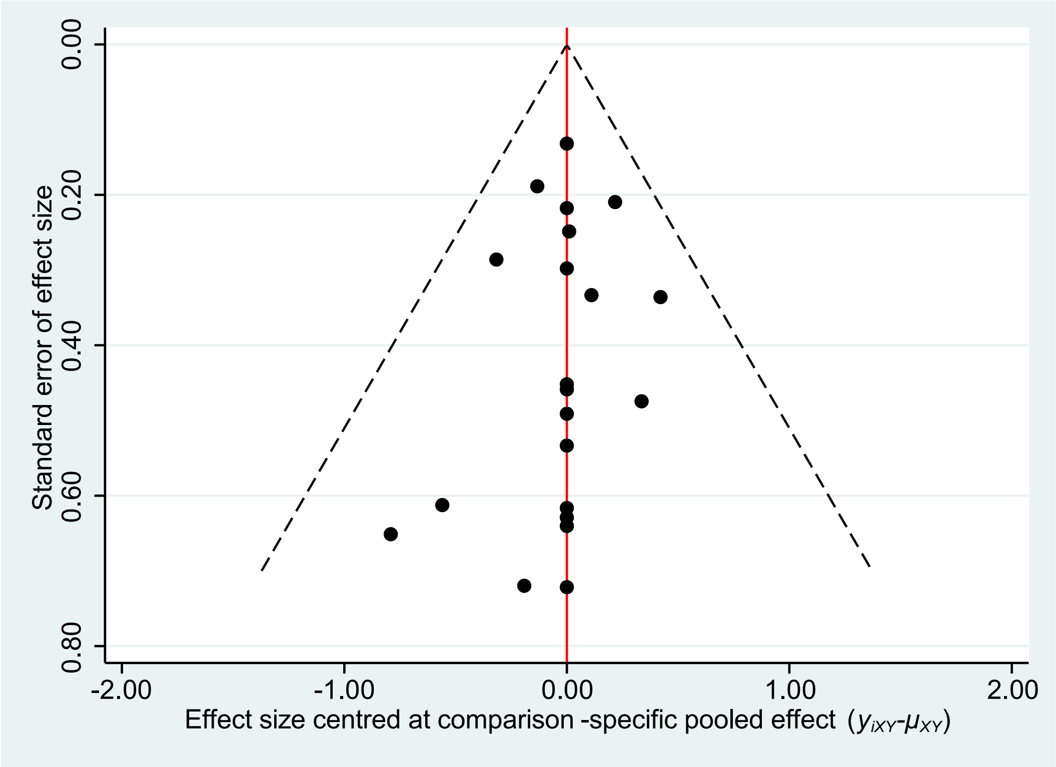

Figure 4 - Comparison-adjusted funnel plot for living at home: medium-term available care network

11.2.3 Living at home available care network, long-term timeframe

Table 12 - Long-term living at home available-care network

					ROB					
**Study**	**Frailty**	**n**	**Experimental group**	**Control group**	**D1**	**D2**	**D3**	**D4**	**D5**	**Overall**
**Carpenter 1990**[516]	all	515	rsk-mfa-	ac	-	-	-	+	-	-
**Fischer 2009**[532]	all	4165	eng & mfa-(w/slfm)	ac	+	-	-	+	+	-
**Ford 1971**[533]	pre-frail and frail	300	mfar(w/med)	ac	+	-	+	+	+	-
**Hay 1998**[546]	unclassifiable	386	mfa-	ac	-	-	x	+	-	x
**Kerse 2014**[556]	pre-frail and frail	3629	rsk-mfa-	ac	+/+	-	-	+	-	-
**Kono 2016**[558]	pre-frail	302	mfar(w/med)	mfar	+	-	-	+	+	-
**Kukkonen-Harjula 2017**[561]	pre-frail and frail	299	ADL & ntr & exrc	ac	+	-	+	+	+	-
**Metzelthin 2013**[576]	frail	315	educ & mfar(w/med+slfm)	ac	-/-	-	x	+	-	x
**Stuck 1995**[602]	all	414	educ & mfar(w/med)	ac	+	-	+	+	+	-
**Stuck 2015**[604]	robust and pre-frail	2154	educ & mfar(w/med+slfm)	ac	+	-	x	+	+	x
**Suijker 2016**[605]	frail	1955	mfar(w/med)	ac	+/-	-	-	+	-	-
**Tomita 2007**[612]	frail	110	aids	ac	x	-	x	+	-	xx
**Tulloch 1979**[613]	all	299	mfar(w/med)	ac	-	-	-	+	-	-

n: number of participants. ROB: risk of bias. D#: Domain #. D1: risk of bias arising from the randomisation process (individual); or, for cluster trials, risk of bias arising from the randomisation process / risk of bias arising from the identification or recruitment of participants into clusters. D2: risk of bias due to deviations from the intended interventions (effect of assignment to the intervention). D3: risk of bias due to missing outcome data. D4: risk of bias in measurement of the outcome. D5: risk of bias in selection of the reported result. +: low risk of bias; -: some concerns; x: high risk of bias / serious concerns; xx: very serious concerns (overall risk of bias only). all: robust, pre-frail and frail.

Table 13 - Living at home in the long term: comparisons with available care summary of findings table

Population: Older people

Interventions: Community-based complex interventions

Comparator: Available care (ac)

Outcome: living at home

Timeframe: long term; range of follow up 24 to 43 months

Setting: Community

Total studies: 13

Total participants: 14,843

Comparator rank: Mean 7.5, 95% CI 5 to 9

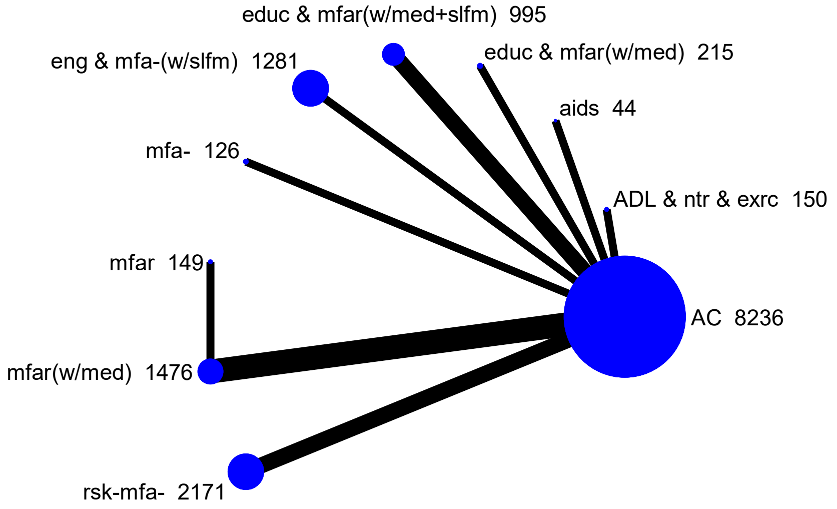

	Relative effect (95% CI)		Anticipated absolute effect (95% CI)						
			High-risk population (607 per 1000 with ac)		Low-risk population (932 per 1000 with ac)				
Intervention group	Network estimate	Calculated risk ratio[a]	With intervention	Difference	With intervention	Difference	Certainty of the evidence (GRADE)	Ranking (95% CI)	Interpretation
Multifactorial-action and review (mfar)	OR 1.29 (0.63 to 2.63) Indirect estimate	RR 1.04 (0.90 to 1.13)	665 per 1000 (492 to 803)	58 more per 1000 (115 fewer to 196 more)	946 per 1000 (896 to 973)	14 more per 1000 (36 fewer to 41 more)	⊕⊕⊖⊖ Low[b]	5.0 (1 to 10)	may result in an increase in chance of living at home
Education, multifactorial-action and review with medication-review (educ & mfar(w/med))	OR 1.23 (0.72 to 2.10) Mixed estimate	RR 1.04 (0.93 to 1.11)	654 per 1000 (525 to 764)	47 more per 1000 (82 fewer to 157 more)	944 per 1000 (908 to 966)	12 more per 1000 (24 fewer to 34 more)	⊕⊕⊖⊖ Low[b]	5.2 (2 to 10)	may result in an increase in chance of living at home
Multifactorial-action and review with medication-review (mfar(w/med))	OR 1.17 (0.94 to 1.47) Mixed estimate	RR 1.03 (0.99 to 1.06)	645 per 1000 (592 to 694)	38 more per 1000 (15 fewer to 87 more)	942 per 1000 (928 to 953)	10 more per 1000 (4 fewer to 21 more)	⊕⊕⊖⊖ Low[b]	5.2 (3 to 9)	may result in a slight increase in chance of living at home
ADL, nutrition and exercise (ADL & ntr & exrc)	OR 1.15 (0.64 to 2.05) Mixed estimate	RR 1.02 (0.91 to 1.10)	639 per 1000 (499 to 760)	32 more per 1000 (108 fewer to 153 more)	940 per 1000 (898 to 966)	8 more per 1000 (34 fewer to 34 more)	⊕⊕⊖⊖ Low[b]	5.8 (2 to 10)	may result in a slight increase in chance of living at home
Meaningful-activities and multifactorial-action with self-management strategies (eng & mfa-(w/slfm))	OR 1.03 (0.85 to 1.25) Mixed estimate	RR 1.01 (0.97 to 1.04)	614 per 1000 (567 to 658)	7 more per 1000 (40 fewer to 51 more)	934 per 1000 (921 to 945)	2 more per 1000 (11 fewer to 13 more)	⊕⊕⊖⊖ Low[b]	6.9 (3 to 10)	may result in a very slight increase in chance of living at home

Risk-screening (rsk-mfa-)	OR 0.91 (0.77 to 1.07) Mixed estimate	RR 0.98 (0.95 to 1.01)	584 per 1000 (543 to 624)	23 fewer per 1000 (64 fewer to 17 more)	926 per 1000 (913 to 936)	6 fewer per 1000 (19 fewer to 4 more)	⊕⊕⊖⊖ Low[b]	8.8 (6 to 10)	may result in a slight reduction in chance of living at home
Aids (aids)	OR 2.64 (1.02 to 6.88) Mixed estimate	RR 1.13 (1.00 to 1.19)	803 per 1000 (611 to 914)	196 more per 1000 (4 more to 307 more)	973 per 1000 (933 to 990)	41 more per 1000 (1 more to 58 more)	⊕⊖⊖⊖ Very low[c,d]	1.8 (1 to 7)	the evidence is very uncertain about the effect on chance of living at home
Multifactorial-action (mfa-)	OR 2.13 (0.85 to 5.33) Mixed estimate	RR 1.11 (0.97 to 1.18)	767 per 1000 (568 to 892)	160 more per 1000 (39 fewer to 285 more)	967 per 1000 (921 to 986)	35 more per 1000 (11 fewer to 54 more)	⊕⊖⊖⊖ Very low[b,e]	2.5 (1 to 9)	the evidence is very uncertain about the effect on chance of living at home
Education, multifactorial-action and review with medication-review and self-management strategies (educ & mfar(w/med+slfm))	OR 1.08 (0.78 to 1.49) Mixed estimate	RR 1.01 (0.95 to 1.06)	625 per 1000 (547 to 697)	18 more per 1000 (60 fewer to 90 more)	937 per 1000 (915 to 953)	5 more per 1000 (17 fewer to 21 more)	⊕⊖⊖⊖ Very low[b,e]	6.3 (3 to 10)	the evidence is very uncertain about the effect on chance of living at home

a: Calculated from OR and an assumed comparator risk of 0.816, the median available care risk among these studies.

b: very serious concerns about imprecision as confidence interval includes substantial benefit and substantial harm. Downgrade twice.

c: very serious concerns about risk of bias due to randomisation process and missing outcome data. Downgrade twice.

d: serious concerns about imprecision as no closed loop and direct comparison is based on 110 persons which does not meet optimal information size. Downgrade once (already downgraded twice for risk of bias).

e: serious concerns about risk of bias due to missing outcome data. Downgrade once.

Table 14 - Results of living at home: long-term available care network

**rsk-mfa-**									0.91 (0.77, 1.07)
0.77 (0.58,1.02)	**mfar(w/med)**	0.91 (0.46, 1.81)							1.17 (0.94, 1.47)
0.71 (0.34,1.48)	0.91 (0.46,1.81)	**mfar**							
0.43 (0.17,1.09)	0.55 (0.21,1.42)	0.60 (0.19,1.94)	**mfa-**						2.13 (0.85, 5.33)
0.88 (0.68,1.14)	1.14 (0.85,1.54)	1.25 (0.60,2.63)	2.07 (0.81,5.29)	**eng & mfa- (w/slfm)**					1.03 (0.85, 1.25)
0.84 (0.58,1.21)	1.09 (0.73,1.61)	1.19 (0.54,2.61)	1.97 (0.74,5.21)	0.95 (0.65,1.39)	**educ & mfar (w/med+slfm)**				1.02 (0.64, 1.63)
0.74 (0.42,1.30)	0.96 (0.54,1.72)	1.05 (0.43,2.57)	1.74 (0.60,5.03)	0.84 (0.47,1.48)	0.88 (0.47,1.65)	**educ & mfar (w/med)**			1.23 (0.72, 2.10)
0.34 (0.13,0.91)	0.44 (0.17,1.19)	0.49 (0.15,1.61)	0.81 (0.21,3.03)	0.39 (0.15,1.03)	0.41 (0.15,1.12)	0.46 (0.15,1.39)	**aids**		2.64 (1.02, 6.88)
0.79 (0.43,1.44)	1.02 (0.55,1.90)	1.12 (0.45,2.81)	1.85 (0.63,5.48)	0.90 (0.49,1.65)	0.94 (0.49,1.82)	1.07 (0.49,2.35)	2.30 (0.75,7.03)	**ADL & ntr & exrc**	1.15 (0.64, 2.05)
0.91 (0.77,1.07)	1.17 (0.94,1.47)	1.29 (0.63,2.63)	2.13 (0.85,5.33)	1.03 (0.85,1.25)	1.08 (0.78,1.49)	1.23 (0.72,2.10)	2.64 (1.02,6.88)	1.15 (0.64,2.05)	**ac**

Lower left triangle presents the findings (OR with 95% CI) of the network meta-analysis. Upper right triangle presents the findings (OR with 95% CI) of pairwise meta-analyses. A OR>1 favours the upper left intervention; a OR<1 favours the lower right intervention. Within the table, comparisons between treatments should be read from left to right (i.e. treatment 1 versus treatment 2). The estimate effect measure (OR and their 95% CI) is in the cell in common between the row- and column-defining treatment.

Table 15 - Intervention rankings for living at home: long-term available care network

Treatment	SUCRA	Pr(Best)	Mean Rank	LCI Rank	UCI Rank
aids	91.1	58.3	1.8	1	7
mfa-	83	33.1	2.5	1	9
mfar	55.8	4.8	5	1	10
educ & mfar(w/med)	53.2	2.2	5.2	2	10
mfar(w/med)	53.4	0.3	5.2	3	9
ADL & ntr & exrc	46.5	1.2	5.8	2	10
educ & mfar(w/med+slfm)	41.6	0.1	6.3	3	10
eng & mfa-(w/slfm)	34.9	0	6.9	3	10
ac	27.6	0	7.5	5	9
rsk-mfa-	12.9	0	8.8	6	10

SUCRA values (0–100) and mean ranks are presented, based on 1000 simulations. Higher SUCRAs and lower mean ranks indicate better performing interventions. Pr(Best) gives the probability of each specific intervention being ranked best intervention, based on 1000 simulations.

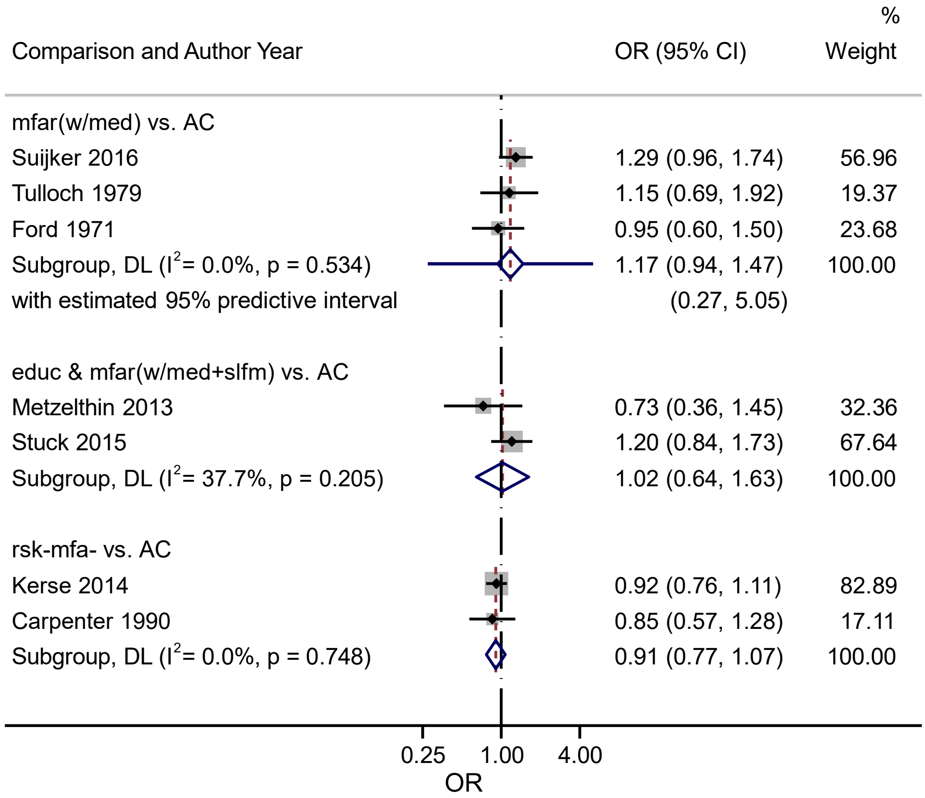

Figure 5 - Pairwise meta-analysis for living at home: long-term available care network (pooling comparisons with greater than one study reporting results)

11.2.4 Living at home homecare network, short-term timeframe

Table 16 - Short-term living at home homecare network

Study	Frailty	n	Experimental group	Control group	ROB D1	D2	D3	D4	D5	Overall
**Fernandez-Barres 2017**[531]	frail	163	hmcr & ntr	hmcr	+	-	-	+	-	-
**King 2012**[557]	pre-frail and frail	174	hmcr & ADL & mfar(w/slfm)	hmcr	+/+	-	+	+	-	-
**Parsons M 2017**[586]	frail	104	hmcr & ADL & mfar(w/slfm)	hmcr & mfa-	-	-	x	+	+	x
**Rooijackers 2021**[594]	frail	263	hmcr & ADL & mfar(w/slfm)	hmcr	+/-	-	+	+	-	-

n: number of participants. ROB: risk of bias. D#: Domain #. D1: risk of bias arising from the randomisation process (individual); or, for cluster trials, risk of bias arising from the randomisation process / risk of bias arising from the identification or recruitment of participants into clusters. D2: risk of bias due to deviations from the intended interventions (effect of assignment to the intervention). D3: risk of bias due to missing outcome data. D4: risk of bias in measurement of the outcome. D5: risk of bias in selection of the reported result. +: low risk of bias; -: some concerns; x: high risk of bias / serious concerns; xx: very serious concerns (overall risk of bias only). all: robust, pre-frail and frail.

Table 17 - Living at home in the short term: comparisons with homecare summary of findings table

Population: Older people

Interventions: Community-based complex interventions

Comparator: homecare (hmcr)

Outcome: living at home

Timeframe: short term; range of follow up 6 to 7 months

Setting: Community

Total studies: 4

Total participants: 704

Comparator rank: Mean 1.1, 95% CI

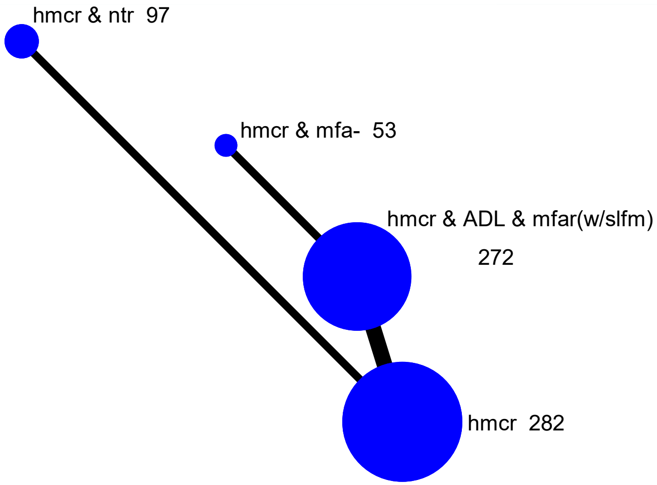

	Relative effect (95% CI)		Anticipated absolute effect (95% CI)						
			High-risk population (923 per 1000 with hmcr)		Low-risk population (953 per 1000 with hmcr)				
Intervention group	Network estimate	Calculated risk ratio[a]	With intervention	Difference	With intervention	Difference	Certainty of the evidence (GRADE)	Ranking (95% CI)	Interpretation
Homecare, ADL, multifactorial-action and review with self-management strategies (hmcr & ADL & mfar(w/slfm))	OR 0.63 (0.31 to 1.26) Mixed estimate	RR 0.96 (0.86 to 1.02)	882 per 1000 (789 to 938)	41 fewer per 1000 (134 fewer to 15 more)	927 per 1000 (863 to 962)	26 fewer per 1000 (90 fewer to 9 more)	⊕⊕⊖⊖ Low[b]	2.1 (1 to 3)	may result in a reduction in chance of living at home
Homecare and nutrition (hmcr & ntr)	OR 0.34 (0.12 to 0.95) Mixed estimate	RR 0.87 (0.64 to 1.00)	801 per 1000 (588 to 919)	122 fewer per 1000 (335 fewer to 4 fewer)	872 per 1000 (707 to 951)	81 fewer per 1000 (246 fewer to 2 fewer)	⊕⊕⊖⊖ Low[c]	3.2 (2 to 4)	may result in a reduction in chance of living at home
Homecare and multifactorial-action (hmcr & mfa-)	OR 0.26 (0.09 to 0.77) Indirect estimate	RR 0.82 (0.56 to 0.98)	756 per 1000 (512 to 902)	167 fewer per 1000 (411 fewer to 21 fewer)	840 per 1000 (639 to 940)	113 fewer per 1000 (314 fewer to 13 fewer)	⊕⊖⊖⊖ Very low[d,e]	3.6 (3 to 4)	the evidence is very uncertain about the effect on chance of living at home

a: Calculated from OR and an assumed comparator risk of 0.924, the median available care risk among these studies.

b: very serious concerns about imprecision as confidence interval includes substantial benefit and substantial harm. Downgrade twice.

c: very serious concerns about imprecision as the optimal information size is not met. The confidence interval is very wide: OR CI ratio 8.0; 588 to 919 per 1000 in the high-risk population. There is no closed loop and the direct comparison is based on evidence from 163 persons. Downgrade twice.

d: serious concerns about risk of bias due to missing outcome data. Downgrade once.

e: very serious concerns about imprecision as the optimal information size is not met. The confidence interval is very wide: OR CI ratio 8.8; 512 to 902 per 1000 in the high-risk population. There is no direct evidence, the indirect evidence coming from the comparison of hmcr & mfa- vs hmcr & ADL & mfar(w/slfm) with 104 participants. Downgrade twice.

Table 18 - Results of living at home: short-term homecare network

**hmcr & ntr**			0.34 (0.12,0.95)
1.30 (0.29,5.85)	**hmcr & mfa-**	0.41 (0.18,0.95)	
0.54 (0.15,1.88)	0.41 (0.18,0.95)	**hmcr & ADL & mfar(w/slfm)**	0.63 (0.31,1.26)
0.34 (0.12,0.95)	0.26 (0.09,0.77)	0.63 (0.31,1.26)	**hmcr**

Lower left triangle presents the findings (OR with 95% CI) of the network meta-analysis. Upper right triangle presents the findings (OR with 95% CI) of pairwise meta-analyses. A OR>1 favours the upper left intervention; a OR<1 favours the lower right intervention. Within the table, comparisons between treatments should be read from left to right (i.e. treatment 1 versus treatment 2). The estimate effect measure (OR and their 95% CI) is in the cell in common between the row- and column-defining treatment.

Table 19 - Intervention rankings for living at home: short-term homecare network

Treatment	SUCRA	Pr(Best)	Mean Rank	LCI Rank	UCI Rank
hmcr	95.7	87.4	1.1	1	2
hmcr & ADL & mfar(w/slfm)	64.4	10.4	2.1	1	3
hmcr & ntr	26.2	2.1	3.2	1	4
hmcr & mfa-	13.6	0.1	3.6	3	4

SUCRA values (0–100) and mean ranks are presented, based on 1000 simulations. Higher SUCRAs and lower mean ranks indicate better performing interventions. Pr(Best) gives the probability of each specific intervention being ranked best intervention, based on 1000 simulations.

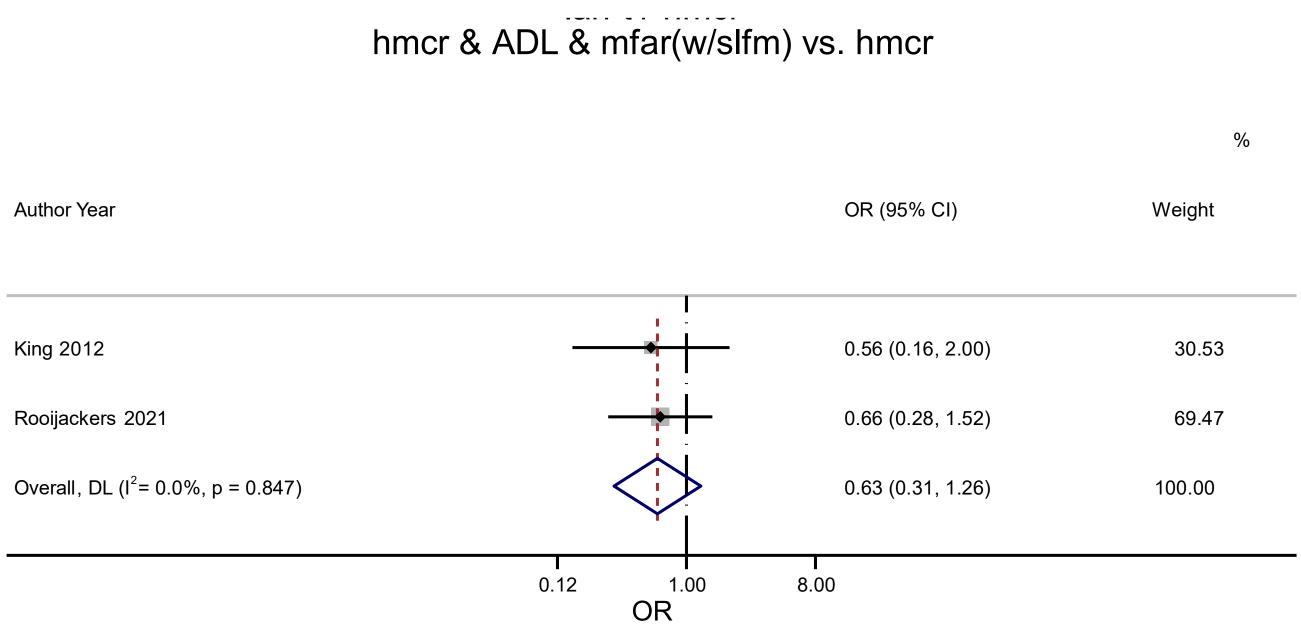

Figure 6 - Pairwise meta-analysis for living at home: short-term homecare network (pooling comparisons with greater than one study reporting results)

11.2.5 Living at home homecare network, medium-term timeframe

Table 20 - Medium-term living at home homecare network

Study	Frailty	n	Experimental group	Control group	ROB D1	D2	D3	D4	D5	Overall
**Fernandez-Barres 2017**[531]	frail	156	hmcr & ntr	hmcr	+	-	x	+	-	x
**Lewin 2013**[565]	frail	744	hmcr & educ & mfar	hmcr	x	x	+	+	-	xx
**Parsons M 2017**[586]	frail	87	hmcr & ADL & mfar(w/slfm)	hmcr & mfa-	-	-	x	+	+	x
**Rooijackers 2021**[594]	frail	259	hmcr & ADL & mfar(w/slfm)	hmcr	+/-	-	+	+	-	-
**Wolter 2013**[629]	frail	732	hmcr & mfar(w/med)	hmcr	+/-	-	x	+	-	x

n: number of participants. ROB: risk of bias. D#: Domain #. D1: risk of bias arising from the randomisation process (individual); or, for cluster trials, risk of bias arising from the randomisation process / risk of bias arising from the identification or recruitment of participants into clusters. D2: risk of bias due to deviations from the intended interventions (effect of assignment to the intervention). D3: risk of bias due to missing outcome data. D4: risk of bias in measurement of the outcome. D5: risk of bias in selection of the reported result. +: low risk of bias; -: some concerns; x: high risk of bias / serious concerns; xx: very serious concerns (overall risk of bias only).

Table 21 - Living at home in the medium term: comparisons with homecare summary of findings table

Population: Older people

Interventions: Community-based complex interventions

Comparator: homecare (hmcr)

Outcome: living at home

Timeframe: medium term; range of follow up 12 to 13 months

Setting: Community

Total studies: 5

Total participants: 1978

Comparator rank: Mean 3.0, 95% CI 1 to 5

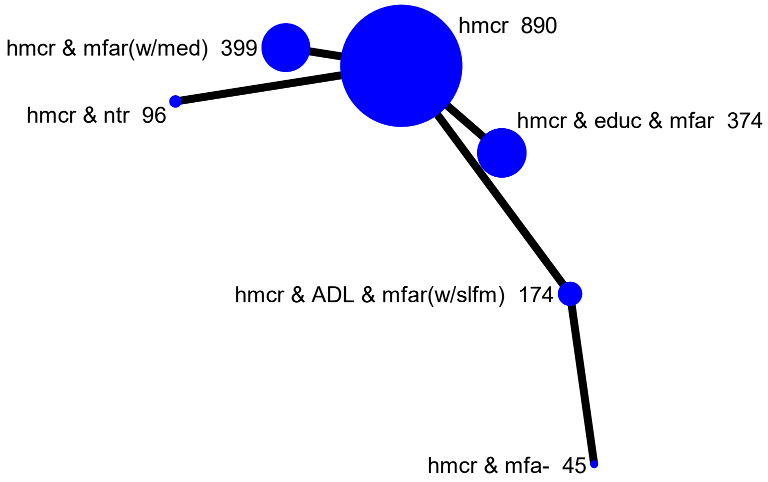

	Relative effect (95% CI)		Anticipated absolute effect (95% CI)						
			High-risk population (649 per 1000 with hmcr)		Low-risk population (843 per 1000 with hmcr)				
Intervention group	Network estimate	Calculated risk ratio[a]	With intervention	Difference	With intervention	Difference	Certainty of the evidence (GRADE)	Ranking (95% CI)	Interpretation
Homecare, ADL, multifactorial-action and review with self-management strategies (hmcr & ADL & mfar(w/slfm))	OR 0.76 (0.40 to 1.45) Mixed estimate	RR 0.92 (0.72 to 1.09)	585 per 1000 (426 to 728)	64 fewer per 1000 (223 fewer to 79 more)	804 per 1000 (683 to 886)	39 fewer per 1000 (160 fewer to 43 more)	⊕⊕⊖⊖ Low[b]	3.8 (1 to 6)	may result in a reduction in chance of living at home
Homecare, education, multifactorial-action and review (hmcr & educ & mfar)	OR 1.17 (0.85 to 1.59) Mixed estimate	RR 1.04 (0.96 to 1.11)	683 per 1000 (612 to 747)	34 more per 1000 (37 fewer to 98 more)	862 per 1000 (821 to 895)	19 more per 1000 (22 fewer to 52 more)	⊕⊖⊖⊖ Very low[b,c]	1.8 (1 to 4)	the evidence is very uncertain about the effect on chance of living at home
Homecare, multifactorial-action and review with medication-review (hmcr & mfar(w/med))	OR 1.11 (0.82 to 1.51) Mixed estimate	RR 1.03 (0.94 to 1.10)	672 per 1000 (601 to 736)	23 more per 1000 (48 fewer to 87 more)	856 per 1000 (814 to 890)	13 more per 1000 (29 fewer to 47 more)	⊕⊖⊖⊖ Very low[b,d]	2.1 (1 to 4)	the evidence is very uncertain about the effect on chance of living at home
Homecare and multifactorial-action (hmcr & mfa-)	OR 0.51 (0.17 to 1.49) Indirect estimate	RR 0.80 (0.45 to 1.09)	485 per 1000 (243 to 734)	164 fewer per 1000 (406 fewer to 85 more)	732 per 1000 (483 to 889)	111 fewer per 1000 (360 fewer to 46 more)	⊕⊖⊖⊖ Very low[b,d]	5.1 (1 to 6)	the evidence is very uncertain about the effect on chance of living at home
Homecare and nutrition (hmcr & ntr)	OR 0.50 (0.23 to 1.07) Mixed estimate	RR 0.79 (0.54 to 1.02)	480 per 1000 (301 to 664)	169 fewer per 1000 (348 fewer to 15 more)	729 per 1000 (556 to 852)	114 fewer per 1000 (287 fewer to 9 more)	⊕⊖⊖⊖ Very low[b,d]	5.2 (2 to 6)	the evidence is very uncertain about the effect on chance of living at home

a: Calculated from OR and an assumed comparator risk of 0.738, the median available care risk among these studies.

b: very serious concerns about imprecision as confidence interval includes substantial benefit and substantial harm. Downgrade twice.

c: very serious concerns about risk of bias due to randomisation process and missing outcome data. Already downgraded twice for imprecision, downgrade once.

d: serious concerns about risk of bias due to missing outcome data. Downgrade once.

Table 22 - Results of living at home: medium-term homecare network

**hmcr & ntr**					0.50 (0.23,1.07)
0.45 (0.20,1.03)	**hmcr & mfar(w/med)**				1.11 (0.82,1.51)
0.98 (0.26,3.67)	2.18 (0.71,6.65)	**hmcr & mfa-**		0.67 (0.28,1.58)	
0.43 (0.19,0.98)	0.95 (0.61,1.47)	0.44 (0.14,1.33)	**hmcr & educ & mfar**		1.17 (0.85,1.59)
0.66 (0.24,1.78)	1.45 (0.71,2.96)	0.67 (0.28,1.58)	1.53 (0.75,3.13)	**hmcr & ADL & mfar(w/slfm)**	0.76 (0.40,1.45)
0.50 (0.23,1.07)	1.11 (0.82,1.51)	0.51 (0.17,1.49)	1.17 (0.85,1.59)	0.76 (0.40,1.45)	**hmcr**

Lower left triangle presents the findings (OR with 95% CI) of the network meta-analysis. Upper right triangle presents the findings (OR with 95% CI) of pairwise meta-analyses. A OR>1 favours the upper left intervention; a OR<1 favours the lower right intervention. Within the table, comparisons between treatments should be read from left to right (i.e. treatment 1 versus treatment 2). The estimate effect measure (OR and their 95% CI) is in the cell in common between the row- and column-defining treatment.

Table 23 - Intervention rankings for living at home: medium-term homecare network

Treatment	SUCRA	Pr(Best)	Mean Rank	LCI Rank	UCI Rank
hmcr & educ & mfar	83.9	50.5	1.8	1	4
hmcr & mfar(w/med)	77.7	33.2	2.1	1	4
hmcr	60.3	3.6	3.0	1	5
hmcr & ADL & mfar(w/slfm)	43.4	7.9	3.8	1	6
hmcr & mfa-	18.7	3.9	5.1	1	6
hmcr & ntr	16.0	0.9	5.2	2	6

SUCRA values (0–100) and mean ranks are presented, based on 1000 simulations. Higher SUCRAs and lower mean ranks indicate better performing interventions. Pr(Best) gives the probability of each specific intervention being ranked best intervention, based on 1000 simulations.

11.2.6 Living at home homecare network, long-term timeframe

No results as there were too few comparisons to conduct network meta-analysis.

11.3 Instrumental ADL

11.3.1 IADL available care network, short-term timeframe

Table 24 - Short-term IADL available-care network

Study	Frailty	n	Experimental group	Control group	ROB D1	D2	D3	D4	D5	Overall
**Clark 1997**[519]	robust and pre-frail	304	eng & educ	ac	x	-	x	-	x	xx
**Gitlin 2006**[539]	pre-frail and frail	300	ADL & aids & exrc	ac	+	-	x	-	-	x
**Metzelthin 2013**[576]	frail	316	educ & mfar(w/med+slfm)	ac	-/-	-	x	-	-	x
**Morgan 2019**[581]	pre-frail	47	exrc	ac	+	-	-	x	-	x
**Rockwood 2000**[592]	frail	148	mfa-(w/med)	ac	-	-	x	-	-	x
**Szanton 2011**[606]	pre-frail and frail	40	ADL&aids&educ&exrc& mfar(w/med+slfm)	ac	-	-	x	-	-	x

n: number of participants. ROB: risk of bias. D#: Domain #. D1: risk of bias arising from the randomisation process (individual); or, for cluster trials, risk of bias arising from the randomisation process / risk of bias arising from the identification or recruitment of participants into clusters. D2: risk of bias due to deviations from the intended interventions (effect of assignment to the intervention). D3: risk of bias due to missing outcome data. D4: risk of bias in measurement of the outcome. D5: risk of bias in selection of the reported result. +: low risk of bias; -: some concerns; x: high risk of bias / serious concerns; xx: very serious concerns (overall risk of bias only). all: robust, pr

e-frail and frail.

Table 25 - IADL in the short term: comparisons with available care summary of findings table

Population: Older people

Interventions: Community-based complex interventions

Comparator: Available care (ac)

Outcome: independence in instrumental activities of daily living

Timeframe: short term; range of follow up 24 weeks to 9 months

Setting: Community

Total studies: 6

Total participants: 1155

Comparator rank: Mean 4.4, 95% CI 2 to 6

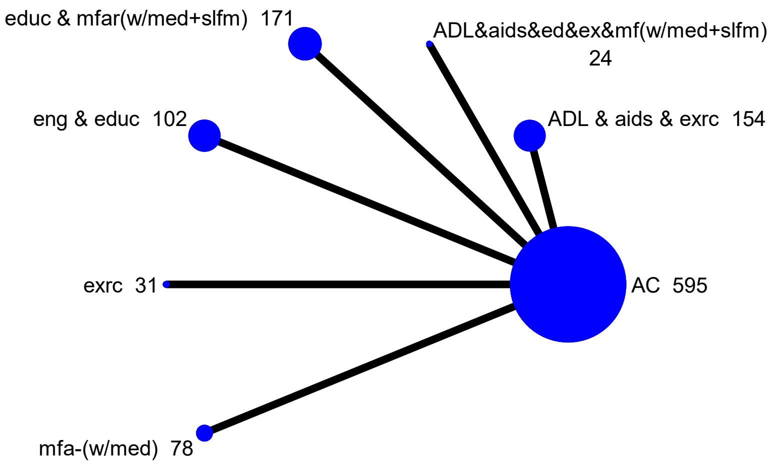

Intervention group	Anticipated absolute effect (95% CI)		Certainty of the evidence (GRADE)	Ranking (95% CI)	Interpretation
	SMD	MD (Lawton IADL 0 to 8)[a]			
Education, multifactorial-action and review with medication-review and self-management (educ & mfar(w/med+slfm))	SMD 0.22 lower (0.45 lower to 0.00) Mixed estimate	MD 0.59 lower (1.17 lower to 0.01 lower)	⊕⊕⊖⊖ **Low**[b,c]	6.5 (5 to 7)	may result in a slight reduction in IADL independence
ADL, aids, education, exercise, multifactorial-action and review with medication-review and self-management (ADL&aids&ed&ex&mf(w/med+slfm))	SMD 0.38 higher (0.26 lower to 1.01 higher) Mixed estimate	MD 0.98 higher (0.69 lower to 2.66 higher)	⊕⊖⊖⊖ **Very low**[b,d]	1.9 (1 to 7)	the evidence is very uncertain about the effect on IADL independence
ADL training, aids-adaptations and physical exercise (ADL & aids & exrc)	SMD 0.14 higher (0.09 lower to 0.36 higher) Mixed estimate	MD 0.36 higher (0.24 lower to 0.95 higher)	⊕⊖⊖⊖ **Very low**[b,d]	2.8 (1 to 6)	the evidence is very uncertain about the effect on IADL independence
Meaningful-activities and education (eng & educ)	SMD 0.06 higher (0.18 lower to 0.30 higher) Mixed estimate	MD 0.16 higher (0.46 lower to 0.79 higher)	⊕⊖⊖⊖ **Very low**[e,f]	3.5 (1 to 6)	the evidence is very uncertain about the effect on IADL independence
Exercise (exrc)	SMD 0.00 (0.60 lower to 0.60 higher) Mixed estimate	MD 0.00 (1.58 lower to 1.58 higher)	⊕⊖⊖⊖ **Very low**[d,g]	4.2 (1 to 7)	the evidence is very uncertain about the effect on IADL independence
Multifactorial-action with medication-review (mfa-(w/med))	SMD 0.05 lower (0.37 lower to 0.27 higher) Mixed estimate	MD 0.13 lower (0.98 lower to 0.71 higher)	⊕⊖⊖⊖ **Very low**[b,d]	4.8 (2 to 7)	the evidence is very uncertain about the effect on IADL independence

a: calculated from the estimated SMD using a standard deviation of 2.62, the pooled standard deviation across intervention groups reporting the Lawton IADL.

b: serious concerns about risk of bias due to missing outcome data. Downgrade once.

c: serious concerns about imprecision as no closed loop and direct comparison is based on 316 persons which does not meet optimal information size. Downgrade once.

d: very serious concerns about imprecision as confidence interval includes substantial benefit and harm (SMD +/- 0.05). Downgrade twice.

e: very serious concerns about risk of bias due to randomisation process, missing outcome data, and reported results were not analysed according to allocation. Downgrade twice.

f: very serious concerns about imprecision as confidence interval includes substantial benefit and harm (SMD +/- 0.05). Already downgraded twice for risk of bias, downgrade once.

g: serious concerns about risk of bias due to ceiling effect in the outcome measurement for a substantial proportion of participants. Downgrade once.

Table 26 - Results of IADL: short-term available care network

**mfa-(w/med)**						-0.05 (-0.37,0.27)
-0.05 (-0.74,0.63)	**exrc**					0.00 (-0.60,0.60)
-0.11 (-0.51,0.29)	-0.06 (-0.71,0.59)	**eng & educ**				0.06 (-0.18,0.30)
0.17 (-0.22,0.56)	0.22 (-0.42,0.87)	0.29 (-0.04,0.61)	**educ & mfar(w/med+slfm)**			-0.22 (-0.45,-0.00)
-0.43 (-1.14,0.29)	-0.38 (-1.25,0.50)	-0.31 (-0.99,0.37)	-0.60 (-1.28,0.08)	**ADL&aids&ed&ex&mf(w/med+slfm)**		0.38 (-0.26,1.01)
-0.19 (-0.58,0.21)	-0.14 (-0.78,0.51)	-0.07 (-0.40,0.25)	-0.36 (-0.68,-0.04)	0.24 (-0.44,0.92)	**ADL & aids & exrc**	0.14 (-0.09,0.36)
-0.05 (-0.37,0.27)	-0.00 (-0.60,0.60)	0.06 (-0.18,0.30)	-0.22 (-0.45,-0.00)	0.38 (-0.26,1.01)	0.14 (-0.09,0.36)	**ac**

Lower left triangle presents the findings (SMD with 95% CI) of the network meta-analysis. Upper right triangle presents the findings (SMD with 95% CI) of pairwise meta-analyses. A SMD>1 favours the upper left intervention; a SMD<1 favours the lower right intervention. Within the table, comparisons between treatments should be read from left to right (i.e. treatment 1 versus treatment 2). The estimate effect measure (SMD and their 95% CI) is in the cell in common between the row- and column-defining treatment.

Table 27 - Intervention rankings for IADL: short-term available care network

Treatment	SUCRA	Pr(Best)	Mean Rank	LCI Rank	UCI Rank
ADL&aids&ed&ex&mf(w/med+slfm)	85.6	65.7	1.9	1	7
ADL & aids & exrc	70.3	13.2	2.8	1	6
Eng & educ	57.8	4.8	3.5	1	6
Exrc	46.7	13.7	4.2	1	7
available care	44.1	0.1	4.4	3	6
Mfa-(w/med)	36.6	2.4	4.8	1	7
Educ & mfar(w/med+slfm)	8.9	0.1	6.5	5	7

SUCRA values (0–100) and mean ranks are presented, based on 1000 simulations. Higher SUCRAs and lower mean ranks indicate better performing interventions. Pr(Best) gives the probability of each specific intervention being ranked best intervention, based on 1000 simulations.

Disconnected Network

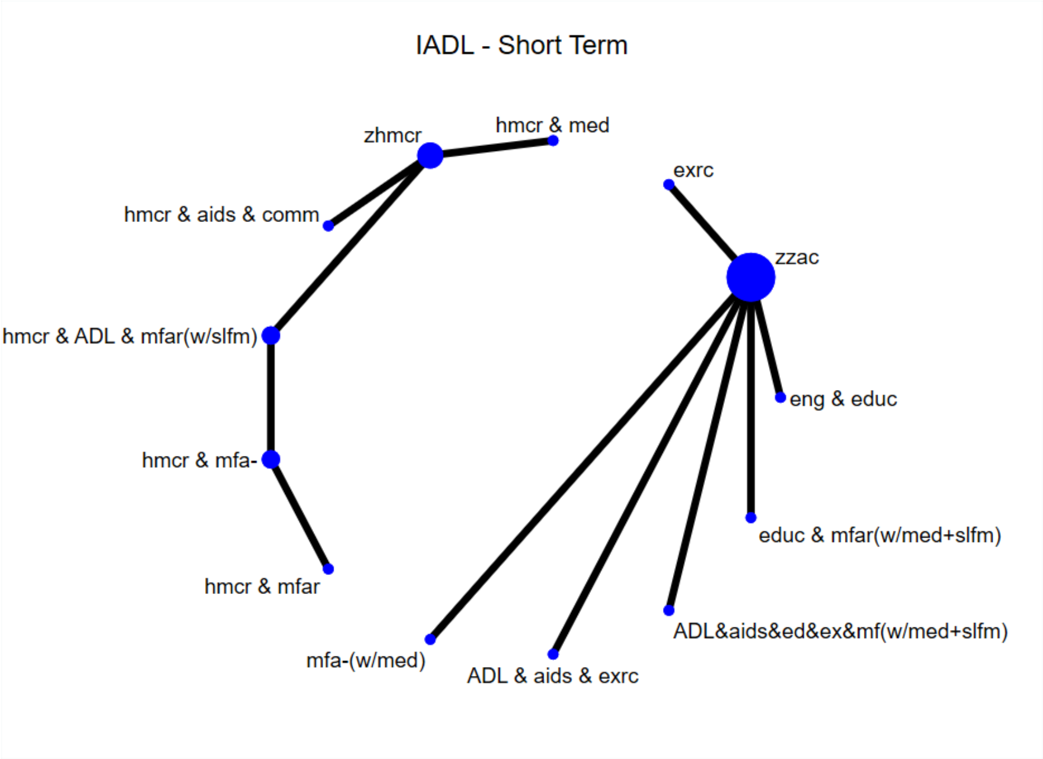

Figure 7 - Example of disconnected network for IADL short-term timeframe, showing separation between studies with available care (ac) comparator and homecare (hmcr) comparator

11.3.2 IADL available care network, medium-term timeframe

Table 28 - Medium-term IADL available-care network

Study	Frailty	n	Experimental group	Control group	ROB D1	D2	D3	D4	D5	Overall
**Blom 2016[510]**	all	1379	mfa-(w/med+slfm)	ac	x/+	-	x	-	-	xx
**Bouman 2008[513]**	pre-frail and frail	293	mfar(w/med)	ac	+	-	x	-	-	x
**Brettschneider 2015[514]**	frail	265	mfar(w/med)	ac	-	-	x	-	+	x
**Clark 1997[519]**	robust and pre-frail	282	eng & educ	ac	x	-	x	-	x	xx
**Dorresteijn 2016[526]**	unclassifiable	312	ADL	ac	+	-	x	-	-	x
**Fabacher 1994[528]**	all	195	mfar(w/med)	ac	-	-	x	-	-	x
**Gene Huguet 2018[536]**	pre-frail	173	med & ntr & exrc	ac	-	-	x	-	-	x
**Gitlin 2006[539]**	pre-frail and frail	285	ADL & aids & exrc	ac	+	-	x	-	-	x
**Henderson 2005[548]**	robust	124	mfar	ac	+/x	+	x	+	-	xx
**Metzelthin 2013[576]**	frail	317	educ & mfar(w/med+slfm)	ac	-/-	-	x	-	-	x
**Monteserin Nadal 2008[578]**	all	430	educ & rsk-mfa-	ac	-	-	x	-	-	x
**Rockwood 2000[592]**	frail	145	mfa-(w/med)	ac	-	-	x	-	-	x
**Rubenstein 2007[595]**	frail	694	mfar(w/med)	ac	-	-	-	-	-	-
**Szanton 2019[607]**	pre-frail and frail	260	ADL&aids&educ&exrc& mfar(w/med+slfm)	ac	+	-	x	-	-	x
**Tomita 2007[612]**	frail	78	aids	ac	x	-	x	-	-	xx
**van Heuvelen 2005[617]**	pre-frail and frail	77	exrc & psyc	ac	-	x	x	-	-	xx

n: number of participants. ROB: risk of bias. D#: Domain #. D1: risk of bias arising from the randomisation process (individual); or, for cluster trials, risk of bias arising from the randomisation process / risk of bias arising from the identification or recruitment of participants into clusters. D2: risk of bias due to deviations from the intended interventions (effect of assignment to the intervention). D3: risk of bias due to missing outcome data. D4: risk of bias in measurement of the outcome. D5: risk of bias in selection of the reported result. +: low risk of bias; -: some concerns; x: high risk of bias / serious concerns; xx: very serious concerns (overall risk of bias only). all: robust, pre-frail and frail.

Table 29 - IADL in the medium term: comparisons with available care summary of findings table

Population: Older people

Interventions: Community-based complex interventions

Comparator: Available care (ac)

Outcome: independence in instrumental activities of daily living

Timeframe: medium term; range of follow up 10 to 18 months

Setting: Community

Total studies: 16

Total participants: 5309

Comparator rank: Mean 7.2, 95% CI 5 to 9

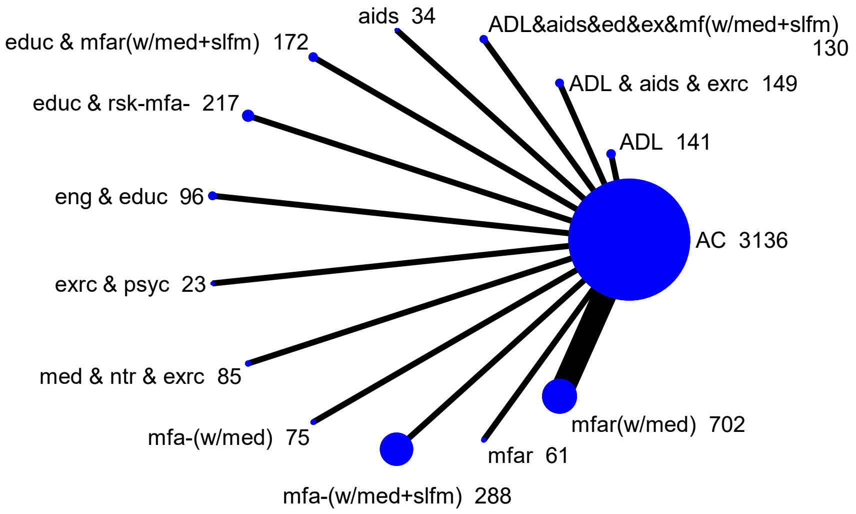

Intervention group	Anticipated absolute effect (95% CI): SMD	Anticipated absolute effect (95% CI): MD (Lawton IADL 0 to 8)[a]	Certainty of the evidence (GRADE)	Ranking (95% CI)	Interpretation
Multifactorial-action and review with medication-review (mfar(w/med))	SMD 0.11 higher (0.00 to 0.21 higher) Mixed estimate	MD 0.28 higher (0.01 higher to 0.55 higher)	⊕⊕⊕⊖ **Moderate**[b]	4.4 (2 to 7)	probably results in a very slight increase in IADL independence
ADL, aids and exercise (ADL & aids & exrc)	SMD 0.19 lower (0.42 lower to 0.04 higher) Mixed estimate	MD 0.50 lower (1.11 lower to 0.11 higher)	⊕⊕⊖⊖ **Low**[b,c]	11.2 (5 to 13)	may result in a slight reduction in IADL independence
ADL, aids, education, exercise, multifactorial-action and review with medication-review and self-management (ADL&aids&ed&ex&mf(w/med+slfm))	SMD 0.56 lower (0.81 lower to 0.31 lower) Mixed estimate	MD 1.47 lower (2.12 lower to 0.82 lower)	⊕⊕⊖⊖ **Low**[b,d]	13.9 (13 to 14)	may result in a reduction in IADL independence
Multifactorial-action and review (mfar)	SMD 0.50 higher (0.15 higher to 0.86 higher) Mixed estimate	MD 1.32 higher (0.38 higher to 2.26 higher)	⊕⊖⊖⊖ **Very low**[e,f]	1.2 (1 to 4)	the evidence is very uncertain about the effect on IADL independence
Medication-review, nutrition and exercise (med & ntr & exrc)	SMD 0.21 higher (0.08 lower to 0.51 higher) Mixed estimate	MD 0.56 higher (0.22 lower to 1.34 higher)	⊕⊖⊖⊖ **Very low**[b,g]	3.2 (1 to 10)	the evidence is very uncertain about the effect on IADL independence
ADL (ADL)	SMD 0.10 higher (0.12 lower to 0.33 higher) Mixed estimate	MD 0.27 higher (0.31 lower to 0.86 higher)	⊕⊖⊖⊖ **Very low**[b,g]	4.9 (2 to 10)	the evidence is very uncertain about the effect on IADL independence

Multifactorial-action with medication-review (mfa-(w/med))	SMD 0.02 higher (0.30 lower to 0.35 higher) Mixed estimate	MD 0.06 higher (0.79 lower to 0.92 higher)	⊕⊝⊝⊝ **Very low**[b,g]	6.7 (2 to 13)	the evidence is very uncertain about the effect on IADL independence
Education and risk-screening (educ & rsk-mfa-)	SMD 0.00 (0.19 lower to 0.19 higher) Mixed estimate	MD 0.00 (0.50 lower to 0.50 higher)	⊕⊝⊝⊝ **Very low**[b,g]	7.1 (3 to 12)	the evidence is very uncertain about the effect on IADL independence
Meaningful-activities and education (eng & educ)	SMD 0.01 lower (0.26 lower to 0.23 higher) Mixed estimate	MD 0.03 lower (0.68 lower to 0.61 higher)	⊕⊝⊝⊝ **Very low**[h,i]	7.5 (2 to 13)	the evidence is very uncertain about the effect on IADL independence
Exercise and psychology (exrc & psyc)	SMD 0.12 lower (0.60 lower to 0.37 higher) Mixed estimate	MD 0.30 lower (1.58 lower to 0.98 higher)	⊕⊝⊝⊝ **Very low**[i,j]	8.9 (2 to 14)	the evidence is very uncertain about the effect on IADL independence
Multifactorial-action with medication-review and self-management (mfa-(w/med+slfm))	SMD 0.07 lower (0.20 lower to 0.06 higher) Mixed estimate	MD 0.19 lower (0.53 lower to 0.15 higher)	⊕⊝⊝⊝ **Very low**[i,k]	9.2 (5 to 12)	the evidence is very uncertain about the effect on IADL independence
Aids (aids)	SMD 0.15 lower (0.60 lower to 0.30 higher) Mixed estimate	MD 0.39 lower (1.56 lower to 0.78 higher)	⊕⊝⊝⊝ **Very low**[i,k]	9.6 (2 to 14)	the evidence is very uncertain about the effect on IADL independence
Education, multifactorial-action and review with medication-review and self-management (educ & mfar(w/med+slfm))	SMD 0.13 lower (0.35 lower to 0.09 higher) Mixed estimate	MD 0.34 lower (0.92 lower to 0.24 higher)	⊕⊝⊝⊝ **Very low**[b,g]	10.1 (5 to 13)	the evidence is very uncertain about the effect on IADL independence

a: calculated from the estimated SMD using a standard deviation of 2.62, the pooled standard deviation across intervention groups reporting the Lawton IADL.

b: serious concerns about risk of bias due to missing outcome data. Downgrade once.

c: serious concerns about imprecision as confidence interval crosses the no effect line and includes substantial harm. Downgrade once.

d: serious concerns about imprecision as no closed loop and direct comparison is based on evidence from 260 persons which does not meet optimal information size. Downgrade once.

e: serious concerns about imprecision as no closed loop and direct comparison is based on evidence from 124 persons which does not meet optimal information size. Downgrade once.

f: very serious concerns about risk of bias due to recruitment of participants and missing outcome data. Downgrade twice.

g: very serious concerns about imprecision as confidence interval includes substantial benefit and harm (SMD +/- 0.05). Downgrade twice.

h: very serious concerns about risk of bias due to the randomisation process, missing outcome data and selection of the reported result. Downgrade twice.

i: very serious concerns about imprecision as confidence interval includes substantial benefit and harm (SMD +/- 0.05). Already downgraded twice for risk of bias, downgrade once.

j: very serious concerns about risk of bias due to excluding participants in per-protocol analysis and missing outcome data. Downgrade twice.

k: very serious concerns about risk of bias due to the randomisation process and missing outcome data. Downgrade twice.

Table 30 - Results of IADL: medium-term available care network

**mfar(w/med)**													0.11 (-0.00, 0.22)
-0.40 (-0.77,-0.03)	**mfar**												0.50 (0.15, 0.86)
0.18 (0.01,0.35)	0.58 (0.20,0.96)	**mfa-(w/med+slfm)**											-0.07 (-0.20, 0.06)
0.08 (-0.26,0.42)	0.48 (-0.00,0.96)	-0.10 (-0.45,0.25)	**mfa-(w/med)**										0.02 (-0.30, 0.35)
-0.11 (-0.42,0.21)	0.29 (-0.18,0.76)	-0.29 (-0.61,0.04)	-0.19 (-0.63,0.25)	**med & ntr & exrc**									0.21 (-0.08, 0.51)
0.22 (-0.28,0.72)	0.62 (0.01,1.23)	0.04 (-0.46,0.55)	0.14 (-0.45,0.73)	0.33 (-0.24,0.90)	**exrc & psyc**								-0.12 (-0.60, 0.37)
0.12 (-0.15,0.39)	0.52 (0.08,0.95)	-0.06 (-0.34,0.22)	0.04 (-0.37,0.44)	0.23 (-0.16,0.61)	-0.10 (-0.65,0.44)	**eng & educ**							-0.01 (-0.26, 0.23)
0.11 (-0.11,0.32)	0.50 (0.10,0.91)	-0.07 (-0.30,0.16)	0.02 (-0.35,0.40)	0.21 (-0.14,0.57)	-0.12 (-0.64,0.41)	-0.01 (-0.32,0.30)	**educ & rsk-mfa-**						0.00 (-0.19, 0.19)
0.24 (-0.01,0.48)	0.63 (0.21,1.06)	0.06 (-0.20,0.31)	0.15 (-0.24,0.55)	0.34 (-0.03,0.72)	0.01 (-0.52,0.55)	0.12 (-0.21,0.45)	0.13 (-0.16,0.42)	**educ & mfar(w/med +slfm)**					-0.13 (-0.35, 0.09)
0.26 (-0.20,0.72)	0.65 (0.08,1.23)	0.07 (-0.39,0.54)	0.17 (-0.38,0.73)	0.36 (-0.18,0.90)	0.03 (-0.63,0.70)	0.14 (-0.37,0.65)	0.15 (-0.34,0.64)	0.02 (-0.48,0.52)	**aids**				-0.15 (-0.60, 0.30)
0.67 (0.40,0.94)	1.07 (0.63,1.50)	0.49 (0.21,0.77)	0.59 (0.18,1.00)	0.78 (0.39,1.16)	0.45 (-0.10,0.99)	0.55 (0.20,0.90)	0.56 (0.25,0.87)	0.43 (0.10,0.76)	0.41 (-0.10,0.92)	**ADL&aids&ed&ex&mf(w/med+slfm)**			-0.56 (-0.81, -0.31)
0.30 (0.04,0.55)	0.70 (0.27,1.12)	0.12 (-0.15,0.38)	0.22 (-0.18,0.62)	0.41 (0.03,0.79)	0.08 (-0.47,0.62)	0.18 (-0.16,0.52)	0.19 (-0.11,0.49)	0.06 (-0.26,0.38)	0.04 (-0.46,0.55)	-0.37 (-0.71,-0.03)	**ADL & aids & exrc**		-0.19 (-0.42, 0.04)
0.00 (-0.24,0.25)	0.40 (-0.02,0.82)	-0.18 (-0.44,0.08)	-0.08 (-0.48,0.31)	0.11 (-0.26,0.48)	-0.22 (-0.76,0.32)	-0.12 (-0.45,0.22)	-0.10 (-0.40,0.19)	-0.24 (-0.55,0.08)	-0.25 (-0.75,0.25)	-0.67 (-1.00,-0.33)	-0.30 (-0.62,0.03)	**ADL**	0.10 (-0.12, 0.33)
0.11 (0.00,0.21)	0.50 (0.15,0.86)	-0.07 (-0.20,0.06)	0.02 (-0.30,0.35)	0.21 (-0.08,0.51)	-0.12 (-0.60,0.37)	-0.01 (-0.26,0.23)	0.00 (-0.19,0.19)	-0.13 (-0.35,0.09)	-0.15 (-0.60,0.30)	-0.56 (-0.81,-0.31)	-0.19 (-0.42,0.04)	0.10 (-0.12,0.33)	**ac**

Lower left triangle presents the findings (SMD with 95% CI) of the network meta-analysis. Upper right triangle presents the findings (SMD with 95% CI) of pairwise meta-analyses. A SMD>1 favours the upper left intervention; a SMD<1 favours the lower right intervention. Within the table, comparisons between treatments should be read from left to right (i.e. treatment 1 versus treatment 2). The estimate effect measure (SMD and their 95% CI) is in the cell in common between the row- and column-defining treatment.

Table 31 - Intervention rankings for IADL: medium-term available care network

Treatment	SUCRA	Pr(Best)	Mean Rank	LCI Rank	UCI Rank
mfar	98.3	86.3	1.2	1	4
Med & ntr & exrc	83.1	8.7	3.2	1	10
Mfar(w/med)	73.9	0.6	4.4	2	7
ADL	70.2	1.1	4.9	2	10
Mfa-(w/med)	55.9	1.1	6.7	2	13
Educ & rsk-mfa-	52.7	0.2	7.1	3	12
ac	52.4	0.0	7.2	5	9
Eng & educ	50.3	0.2	7.5	2	13
Exrc & psyc	38.9	1.1	8.9	2	14
Mfa-(w/med+slfm)	37.3	0.0	9.2	5	12
aids	33.9	0.7	9.6	2	14
Educ & mfar(w/med+slfm)	30.4	0.0	10.1	5	13
ADL & aids & exrc	21.8	0.0	11.2	5	13
ADl&aids&ed&ex&mf(w/med+slfm)	0.9	0.0	13.9	13	14

SUCRA values (0–100) and mean ranks are presented, based on 1000 simulations. Higher SUCRAs and lower mean ranks indicate better performing interventions. Pr(Best) gives the probability of each specific intervention being ranked best intervention, based on 1000 simulations.

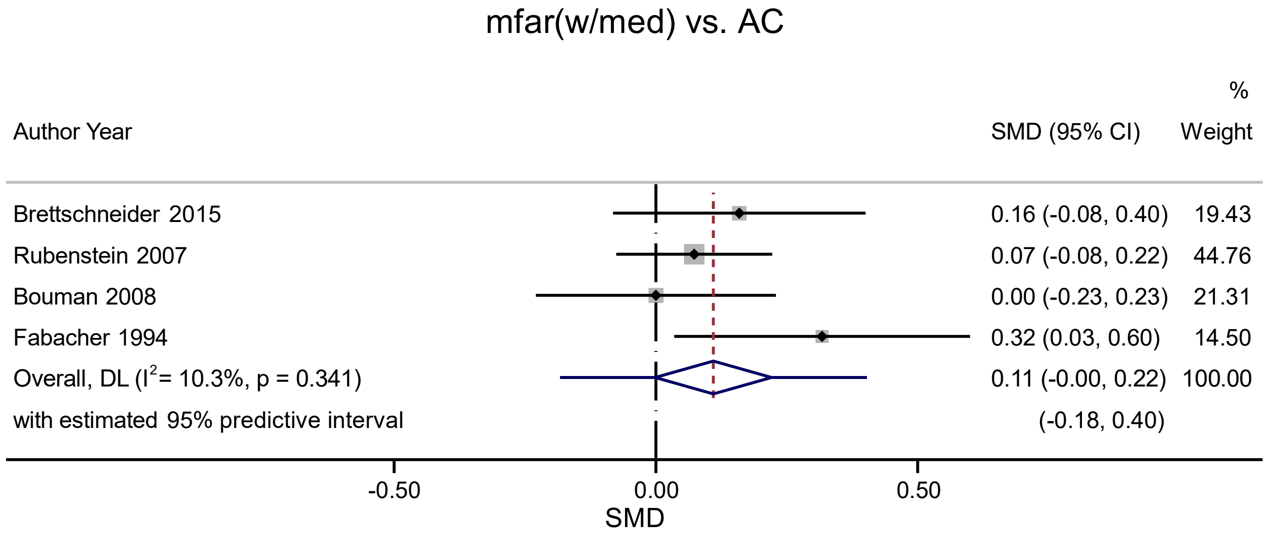

Figure 8 - Pairwise meta-analysis for IADL: medium-term available care network (pooling comparisons with greater than one study reporting results)

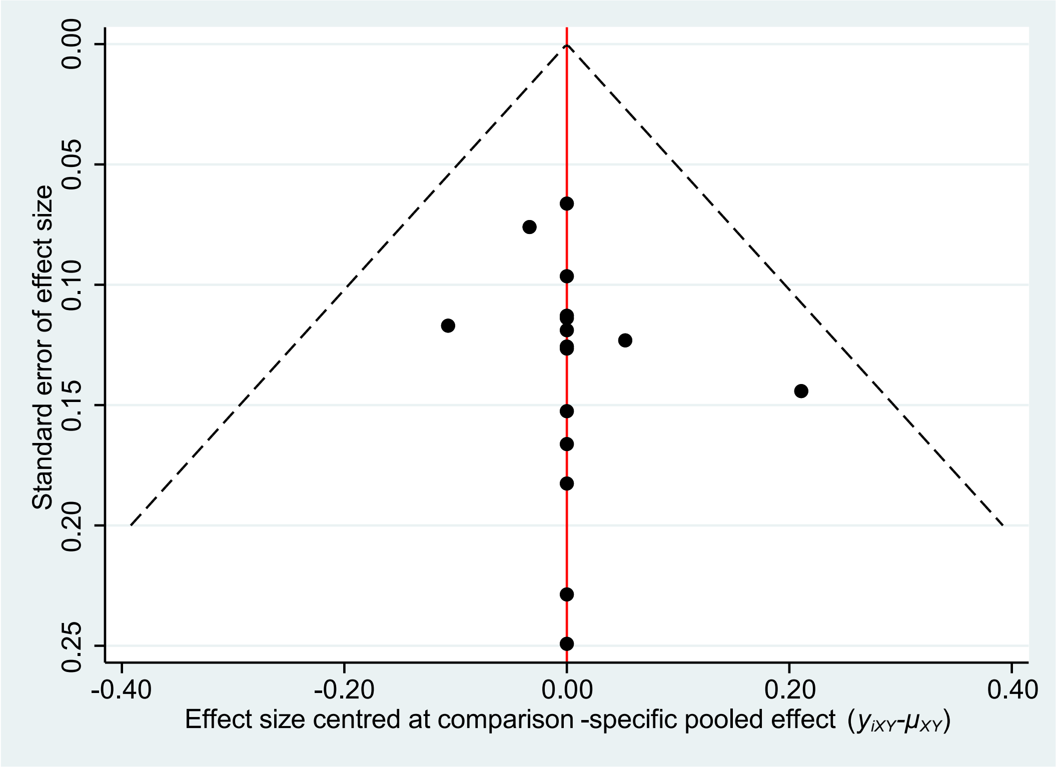

Figure 9 - Comparison-adjusted funnel plot for IADL: medium-term available care network

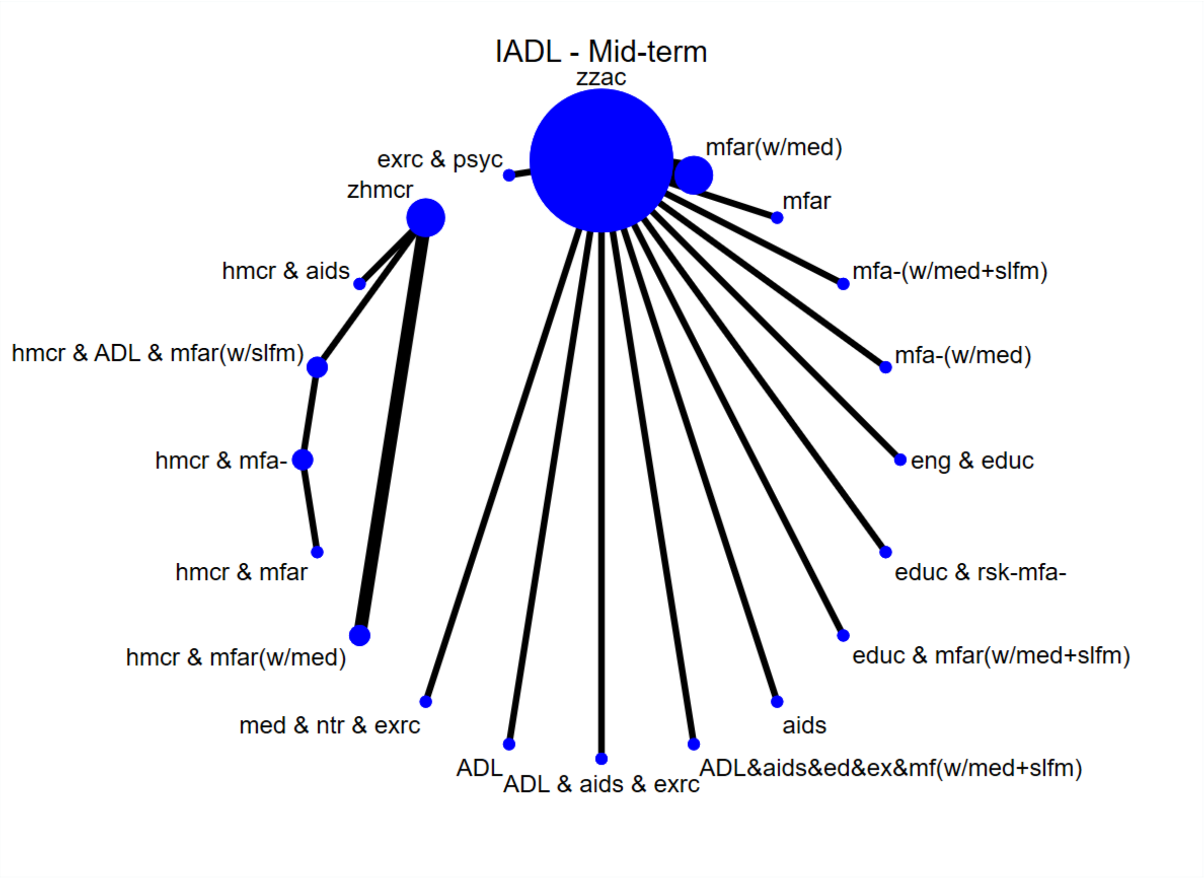

Figure 10 - Example of disconnected network for IADL medium-term timeframe, showing separation between studies with available care (ac) comparator and homecare (hmcr) comparator

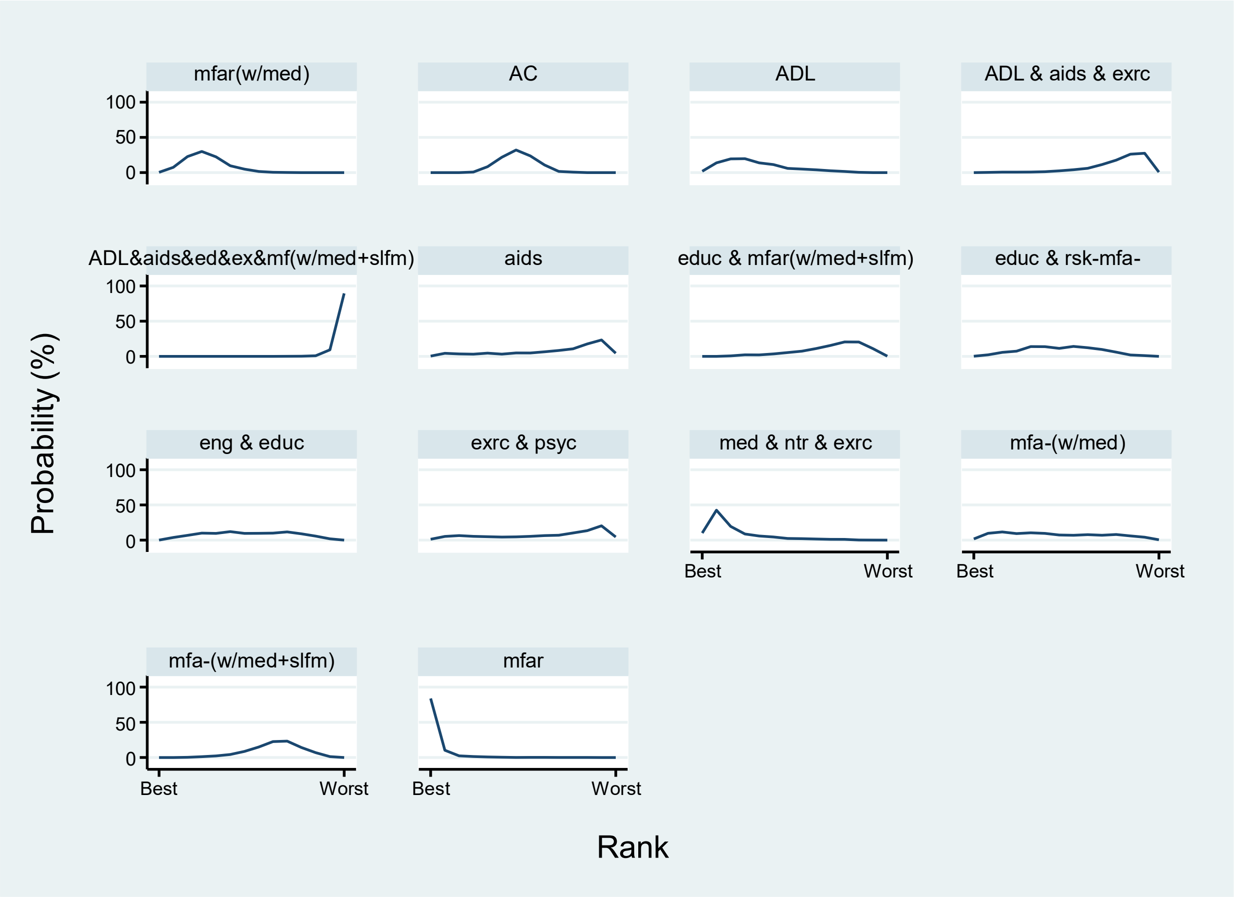

Figure 11 - Rankogram showing comparative effectiveness of interventions for IADL medium-term available care network. Results based on a simulation of 1000 replications.

Risk of Bias

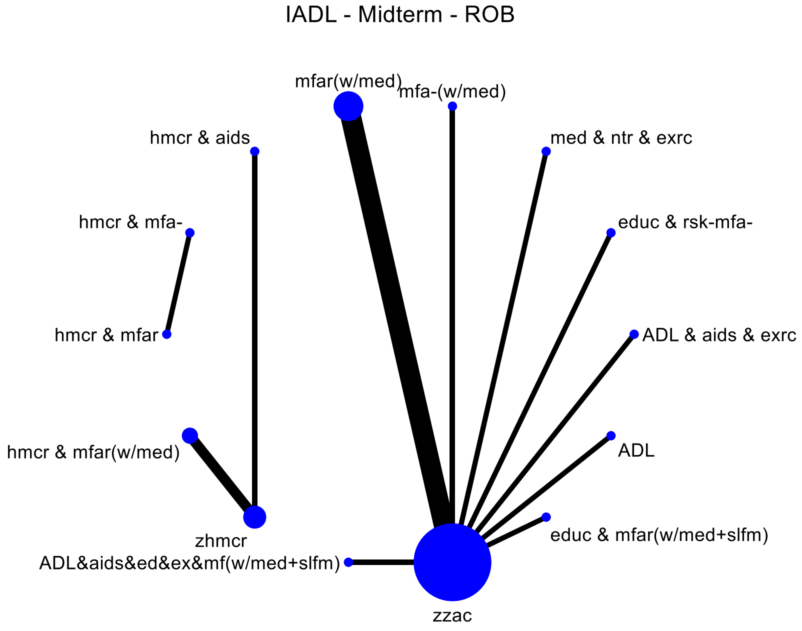

Figure 12 - Example of disconnected network for risk of bias sensitivity analysis for IADL medium-term timeframe, showing separation between studies with available care (ac) comparator and homecare (hmcr) comparator

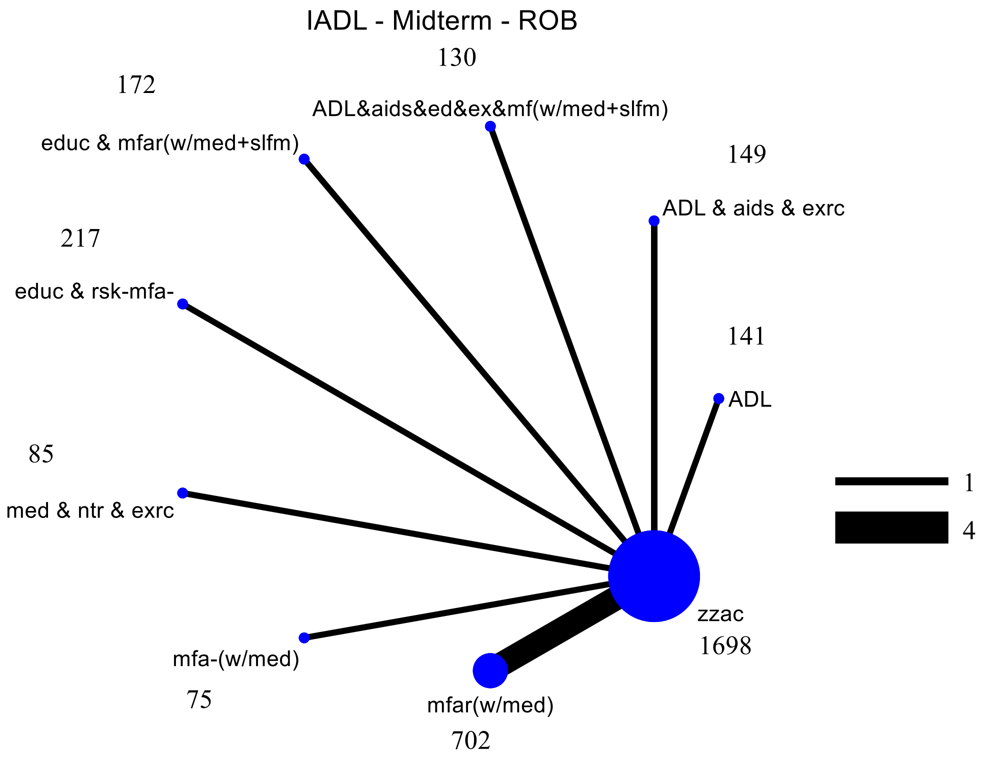

Figure 13 - Network diagram for risk of bias analysis for IADL medium-term timeframe with available care (ac) comparator

11.3.3 IADL available care network, long-term timeframe

Table 32 - Long-term IADL available-care network

					ROB					
**Study**	**Frailty**	**n**	**Experimental group**	**Control group**	**D1**	**D2**	**D3**	**D4**	**D5**	**Overall**
**Bouman 2008**[513]	pre-frail and frail	293	mfar(w/med)	ac	+	-	x	-	-	x
**Jitapunkul 1998**[555]	unclassifiable	116	rsk-mfa-	ac	-	-	-	-	-	-
**Metzelthin 2013**[576]	frail	316	educ & mfar(w/med+slfm)	ac	-/-	-	x	-	-	x
**Rubenstein 2007**[595]	frail	607	mfar(w/med)	ac	-	-	-	-	-	-
**Stuck 1995**[602]	all	317	educ & mfar(w/med)	ac	+	-	-	-	-	-
**Tomita 2007**[612]	frail	78	aids	ac	x	-	x	-	-	xx

n: number of participants. ROB: risk of bias. D#: Domain #. D1: risk of bias arising from the randomisation process (individual); or, for cluster trials, risk of bias arising from the randomisation process / risk of bias arising from the identification or recruitment of participants into clusters. D2: risk of bias due to deviations from the intended interventions (effect of assignment to the intervention). D3: risk of bias due to missing outcome data. D4: risk of bias in measurement of the outcome. D5: risk of bias in selection of the reported result. +: low risk of bias; -: some concerns; x: high risk of bias / serious concerns; xx: very serious concerns (overall risk of bias only). all: robust, pre-frail and frail.

Table 33 - IADL in the long term: comparisons with available care summary of findings table

Population: Older people

Interventions: Community-based complex interventions

Comparator: Available care (ac)

Outcome: instrumental activities of daily living (higher is better)

Timeframe: long term; range of follow up 24 to 36 months

Setting: Community

Total studies: 6

Total participants: 1727

Comparator rank: Mean 3.4, 95% CI 2 to 5

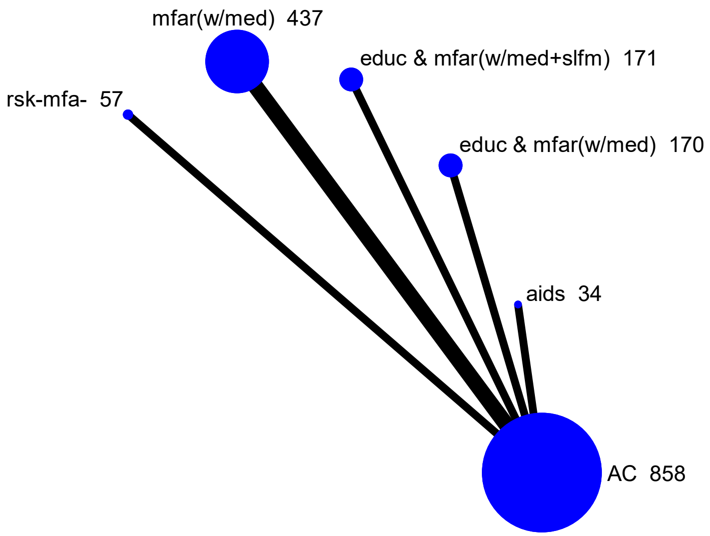

Intervention group	Anticipated absolute effect (95% CI): SMD	Anticipated absolute effect (95% CI): MD (Lawton IADL 0 to 8)[a]	Certainty of the evidence (GRADE)	Ranking (95% CI)	Interpretation
Multifactorial-action and review with medication-review (mfar(w/med))	SMD 0.08 lower (0.21 lower to 0.05 higher) Mixed estimate	MD 0.21 lower (0.56 lower to 0.13 higher)	⊕⊕⊕⊝ **Moderate**[b]	4.5 (3 to 6)	probably results in a very slight reduction in IADL
Risk-screening (rsk-mfa-)	SMD 0.23 higher (0.13 lower to 0.60 higher) Mixed estimate	MD 0.61 higher (0.35 lower to 1.57 higher)	⊕⊕⊝⊝ **Low**[c]	1.7 (1 to 5)	may result in a slight increase in IADL
Education, multifactorial-action and review with medication-review (educ & mfar(w/med))	SMD 0.14 higher (0.08 lower to 0.36 higher) Mixed estimate	MD 0.37 higher (0.21 lower to 0.95 higher)	⊕⊕⊝⊝ **Low**[c]	2.1 (1 to 5)	may result in a very slight increase in IADL
Education, multifactorial-action and review with medication-review and self-management (educ & mfar(w/med+slfm))	SMD 0.21 lower (0.44 lower to 0.01 higher) Mixed estimate	MD 0.56 lower (1.14 lower to 0.02 higher)	⊕⊕⊝⊝ **Low**[b,d]	5.5 (4 to 6)	may result in a slight reduction in IADL
Aids (aids)	SMD 0.03 lower (0.48 lower to 0.42 higher) Mixed estimate	MD 0.07 lower (1.25 lower to 1.10 higher)	⊕⊝⊝⊝ **Very low**[e,f]	3.8 (1 to 6)	the evidence is very uncertain about the effect on IADL

a: calculated from the estimated SMD using a standard deviation of 2.62, the pooled standard deviation across intervention groups reporting the Lawton IADL.

b: serious concerns about imprecision as confidence interval crosses the no effect line and includes substantial harm. Downgrade once.

c: very serious concerns about imprecision as confidence interval includes substantial benefit and harm (SMD +/- 0.05). Downgrade twice.

d: serious concerns about risk of bias due to missing outcome data. Downgrade once.

e: very serious concerns about risk of bias due to randomisation process and missing outcome data. Downgrade twice.

f: very serious concerns about imprecision as confidence interval includes substantial benefit and harm (SMD +/- 0.05). Already downgraded twice for risk of bias, downgrade once.

Table 34 - Results of IADL: long-term available care network

**rsk-mfa-**					0.23 (-0.13,0.60)
0.32 (-0.07,0.70)	**mfar(w/med)**				-0.08 (-0.21,0.05)
0.45 (0.02,0.87)	0.13 (-0.13,0.39)	**educ & mfar(w/med+slfm)**			-0.21 (-0.44,0.01)
0.09 (-0.33,0.52)	-0.22 (-0.48,0.03)	-0.35 (-0.67,-0.04)	**educ & mfar(w/med)**		0.14 (-0.08,0.36)
0.26 (-0.32,0.84)	-0.05 (-0.52,0.41)	-0.19 (-0.69,0.31)	0.17 (-0.33,0.67)	**aids**	-0.03 (-0.48,0.42)
0.23 (-0.13,0.60)	-0.08 (-0.21,0.05)	-0.21 (-0.44,0.01)	0.14 (-0.08,0.36)	-0.03 (-0.48,0.42)	**ac**

Lower left triangle presents the findings (SMD with 95% CI) of the network meta-analysis. Upper right triangle presents the findings (SMD with 95% CI) of pairwise meta-analyses. A SMD>1 favours the upper left intervention; a SMD<1 favours the lower right intervention. Within the table, comparisons between treatments should be read from left to right (i.e. treatment 1 versus treatment 2). The estimate effect measure (SMD and their 95% CI) is in the cell in common between the row- and column-defining treatment.

Table 35 - Intervention rankings for IADL: long-term available care network

Treatment	SUCRA	Pr(Best)	Mean Rank	LCI Rank	UCI Rank
rsk-mfa-	86.5	61.9	1.7	1	5
educ & mfar(w/med)	77.3	25.5	2.1	1	5
ac	52.5	0.4	3.4	2	5
aids	44.3	11.9	3.8	1	6
mfar(w/med)	29.7	0.1	4.5	3	6
educ & mfar(w/med+slfm)	9.7	0.2	5.5	4	6

SUCRA values (0–100) and mean ranks are presented, based on 1000 simulations. Higher SUCRAs and lower mean ranks indicate better performing interventions. Pr(Best) gives the probability of each specific intervention being ranked best intervention, based on 1000 simulations.

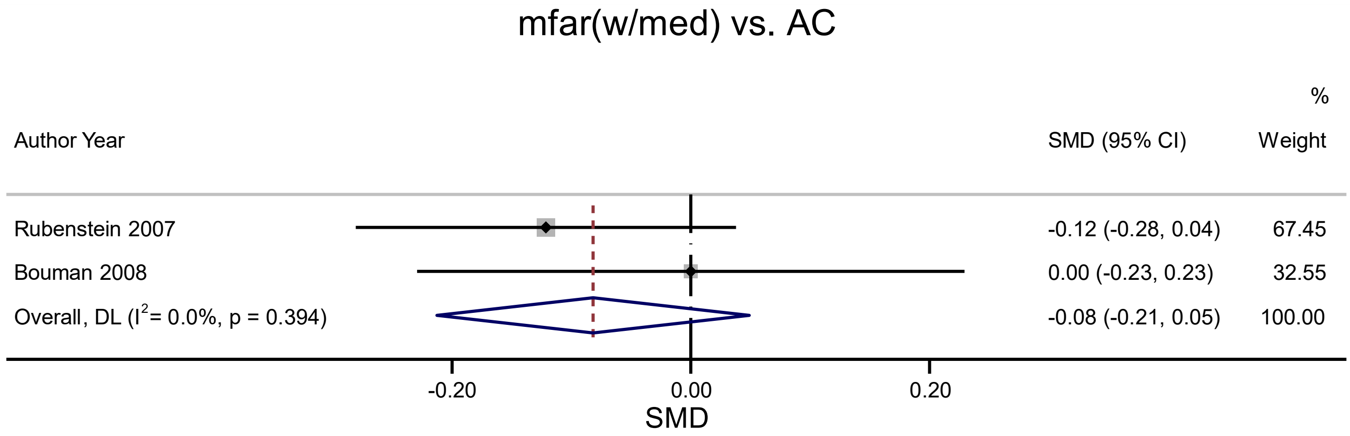

Figure 14 - Pairwise meta-analysis for IADL: long-term available care network (pooling comparisons with greater than one study reporting results)

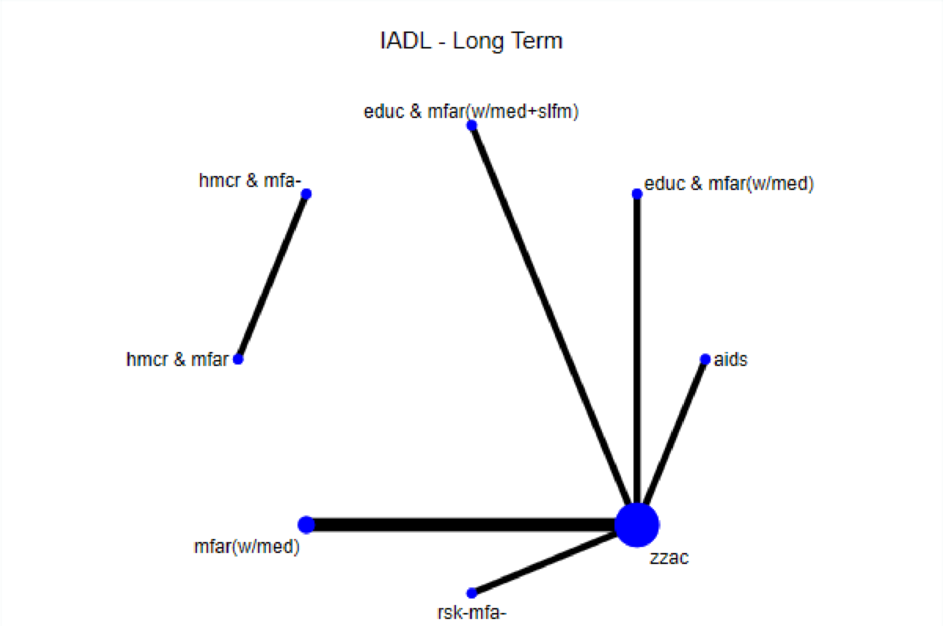

Figure 15 - Example of disconnected network for IADL long-term timeframe, showing separation between studies with available care (ac) comparator and homecare (hmcr) comparator

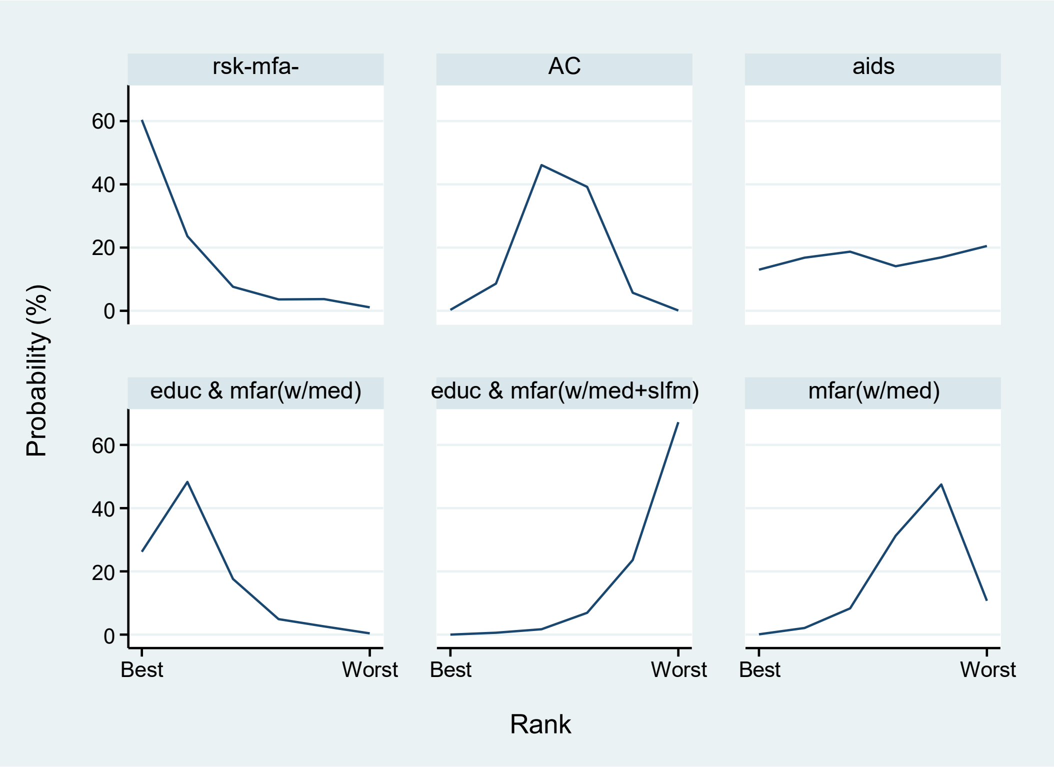

Figure 16 - Rankogram showing comparative effectiveness of interventions for IADL long-term available care network. Results based on a simulation of 1000 replications.

11.3.4 IADL homecare network, short-term timeframe

Table 36 - Short-term IADL homecare network

Study	Frailty	n	Experimental group	Control group	ROB					
					D1	D2	D3	D4	D5	Overall
**Auvinen 2020**[505]	frail	449	hmcr & med	hmcr	+	-	-	-	x	x
**King 2012**[557]	pre-frail and frail	157	hmcr & ADL & mfar(w/slfm)	hmcr	+/+	-	-	-	-	-
**Parsons M 2012**[587]	frail	251	hmcr & mfar	hmcr & mfa-	+/-	-	-	-	-	-
**Parsons M 2017**[586]	frail	113	hmcr & ADL & mfar(w/slfm)	hmcr & mfa-	-	-	x	-	-	x

n: number of participants. ROB: risk of bias. D#: Domain #. D1: risk of bias arising from the randomisation process (individual); or, for cluster trials, risk of bias arising from the randomisation process / risk of bias arising from the identification or recruitment of participants into clusters. D2: risk of bias due to deviations from the intended interventions (effect of assignment to the intervention). D3: risk of bias due to missing outcome data. D4: risk of bias in measurement of the outcome. D5: risk of bias in selection of the reported result. +: low risk of bias; -: some concerns; x: high risk of bias / serious concerns; xx: very serious concerns (overall risk of bias only). all: robust, pre-frail and frail.

Table 37 - IADL in the short term: comparisons with homecare summary of findings table

Population: Older people

Interventions: Community-based complex interventions

Comparator: Formal homecare (hmcr)

Outcome: independence in instrumental activities of daily living (higher is better)

Timeframe: short term; range of follow up 6 to 7 months

Setting: Community

Total studies: 4

Total participants: 970

Comparator rank: Mean 2.5, 95% CI 1 to 4

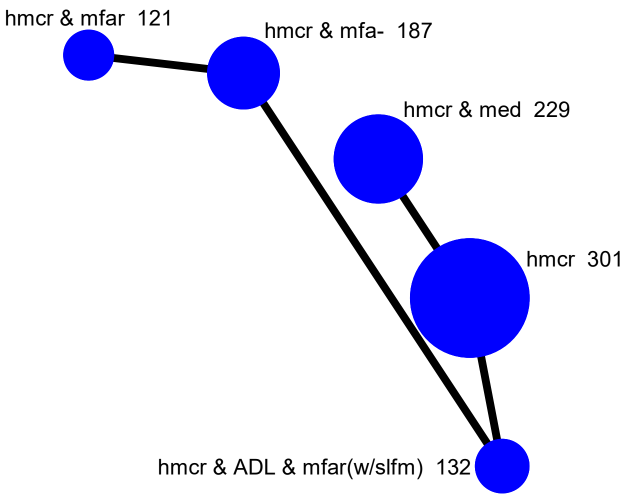

Intervention group	Anticipated absolute effect (95% CI): SMD	Anticipated absolute effect (95% CI): MD (Lawton IADL 0 to 8)[a]	Certainty of the evidence (GRADE)	Ranking (95% CI)	Interpretation
Homecare, ADL, multifactorial-action and review with self-management (hmcr & ADL & mfar(w/slfm))	SMD 0.02 lower (0.33 lower to 0.29 higher) Mixed estimate	MD 0.06 lower (0.88 lower to 0.76 higher)	⊕⊕⊖⊖ **Low**[b]	2.8 (1 to 5)	may result in little to no difference in IADL independence
Homecare, multifactorial-action and review (hmcr & mfar)	SMD 0.04 higher (0.51 lower to 0.58 higher) Indirect estimate	MD 0.09 higher (1.33 lower to 1.52 higher)	⊕⊖⊖⊖ **Very low**[b,c]	2.4 (1 to 5)	the evidence is very uncertain about the effect on IADL independence
Homecare and multifactorial-action (hmcr & mfa-)	SMD 0.07 lower (0.55 lower to 0.42 higher) Indirect estimate	MD 0.18 lower (1.44 lower to 1.09 higher)	⊕⊖⊖⊖ **Very low**[b,d]	3.4 (1 to 5)	the evidence is very uncertain about the effect on IADL independence
Homecare and medication-review (hmcr & med)	SMD 0.13 lower (0.31 lower to 0.06 higher) Mixed estimate	MD 0.33 lower (0.82 lower to 0.15 higher)	⊕⊖⊖⊖ **Very low**[b,e]	3.9 (1 to 5)	the evidence is very uncertain about the effect on IADL independence

a: calculated from the estimated SMD using a standard deviation of 2.62, the pooled standard deviation across intervention groups reporting the Lawton IADL.

b: very serious concerns about imprecision as confidence interval includes substantial benefit and harm (SMD +/- 0.05). Downgrade twice.

c: serious concerns about risk of bias due to missing outcome data in indirect evidence via the homecare, ADL, multifactorial-action and review with self-management vs homecare and multifactorial-action comparison. Downgrade once.

d: serious concerns about risk of bias due to missing outcome data. Downgrade once.

e: serious concerns about risk of bias because multiple analyses were conducted but the results from only one analysis were reported. Downgrade once.

Table 38 - Results of IADL: short-term homecare network

**hmcr & mfar**	0.10 (-0.14,0.35)			
0.10 (-0.14,0.35)	**hmcr & mfa-**		-0.05 (-0.41,0.32)	
0.16 (-0.41,0.74)	0.06 (-0.46,0.58)	**hmcr & med**		-0.13 (-0.31,0.06)
0.06 (-0.39,0.50)	-0.05 (-0.41,0.32)	-0.11 (-0.47,0.26)	**hmcr & ADL & mfar(w/slfm)**	-0.02 (-0.33,0.29)
0.04 (-0.51,0.58)	-0.07 (-0.55,0.42)	-0.13 (-0.31,0.06)	-0.02 (-0.33,0.29)	**hmcr**

Lower left triangle presents the findings (SMD with 95% CI) of the network meta-analysis. Upper right triangle presents the findings (SMD with 95% CI) of pairwise meta-analyses. A SMD>1 favours the upper left intervention; a SMD<1 favours the lower right intervention. Within the table, comparisons between treatments should be read from left to right (i.e. treatment 1 versus treatment 2). The estimate effect measure (SMD and their 95% CI) is in the cell in common between the row- and column-defining treatment.

Table 39 - Intervention rankings for IADL: short-term homecare network

Treatment	SUCRA	Pr(Best)	Mean Rank	LCI Rank	UCI Rank
hmcr & mfar	64.7	41.5	2.4	1	5
hmcr	62.8	30.4	2.5	1	5
hmcr & ADL & mfar(w/s)	54.5	17.1	2.8	1	5
hmcr & mfa-	39.2	6.4	3.4	1	5
hmcr & med	28.7	4.6	3.9	1	5

SUCRA values (0–100) and mean ranks are presented, based on 1000 simulations. Higher SUCRAs and lower mean ranks indicate better performing interventions. Pr(Best) gives the probability of each specific intervention being ranked best intervention, based on 1000 simulations.

11.3.5 IADL homecare network, medium-term timeframe

Table 40 - Medium-term IADL homecare network

					ROB					
**Study**	**Frailty**	**n**	**Experimental group**	**Control group**	**D1**	**D2**	**D3**	**D4**	**D5**	**Overall**
**Bernabei 1998**[508]	frail	199	hmcr & mfar(w/med)	hmcr	-	-	x	-	-	x
**Mann WC 1999**[571]	frail	90	hmcr & aids	hmcr	-	-	x	+	-	x
**Parsons M 2012**[587]	frail	251	hmcr & mfar	hmcr & mfa-	+/-	-	x	-	-	x
**Parsons M 2017**[586]	frail	113	hmcr & ADL & mfar(w/slfm)	hmcr & mfa-	-	-	x	-	-	xx
**Rooijackers 2021**[594]	frail	264	hmcr & ADL & mfar(w/slfm)	hmcr	+/-	-	x	+	x	xx
**Wolter 2013**[629]	frail	484	hmcr & mfar(w/med)	hmcr	+/-	-	x	+	-	x

n: number of participants. ROB: risk of bias. D#: Domain #. D1: risk of bias arising from the randomisation process (individual); or, for cluster trials, risk of bias arising from the randomisation process / risk of bias arising from the identification or recruitment of participants into clusters. D2: risk of bias due to deviations from the intended interventions (effect of assignment to the intervention). D3: risk of bias due to missing outcome data. D4: risk of bias in measurement of the outcome. D5: risk of bias in selection of the reported result. +: low risk of bias; -: some concerns; x: high risk of bias / serious concerns; xx: very serious concerns (overall risk of bias only). all: robust, pre-frail and frail.

Table 41 - IADL in the medium term: comparisons with homecare summary of findings table

Population: Older people

Interventions: Community-based complex interventions

Comparator: homecare (hmcr)

Outcome: independence in instrumental activities of daily living

Timeframe: medium term; range of follow up 12 to 18 months

Setting: Community

Total studies: 6

Total participants: 1401

Comparator rank: Mean 4.7, 95% CI 2 to 6

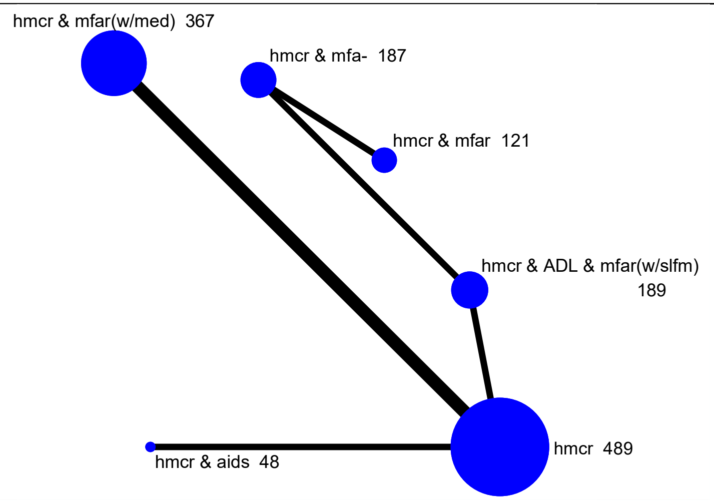

Intervention group	Anticipated absolute effect (95% CI)		Certainty of the evidence (GRADE)	Ranking (95% CI)	Interpretation
	SMD	MD (Lawton IADL 0 to 8)[a]			
Homecare and aids(hmcr & aids)	SMD 0.27 higher (0.23 lower to 0.77 higher) Mixed estimate	MD 0.71 higher (0.60 lower to 2.02 higher)	⊕⊖⊖⊖ **Very low**[b,c]	2.5 (1 to 6)	the evidence is very uncertain about the effect on IADL independence
Homecare, multifactorial-action and review (hmcr & mfar)	SMD 0.18 higher (0.52 lower to 0.88 higher) Indirect estimate	MD 0.47 higher (1.35 lower to 2.30 higher)	⊕⊖⊖⊖ **Very low**[d,e]	3.0 (1 to 6)	the evidence is very uncertain about the effect on IADL independence
Homecare, ADL training, multifactorial-action and review with self-management (hmcr & ADL & mfar(w/slfm))	SMD 0.16 higher (0.21 lower to 0.53 higher) Mixed estimate	MD 0.41 higher (0.55 lower to 1.38 higher)	⊕⊖⊖⊖ **Very low**[e,f]	3.1 (1 to 6)	the evidence is very uncertain about the effect on IADL independence
Homecare, multifactorial-action and review with medication-review (hmcr & mfar(w/med))	SMD 0.15 higher (0.11 lower to 0.41 higher) Mixed estimate	MD 0.38 higher (0.30 lower to 1.06 higher)	⊕⊖⊖⊖ **Very low**[b,c]	3.2 (1 to 6)	the evidence is very uncertain about the effect on IADL independence
Homecare and multifactorial-action (hmcr & mfa-)	SMD 0.01 lower (0.60 lower to 0.58 higher) Indirect estimate	MD 0.02 lower (1.57 lower to 1.52 higher)	⊕⊖⊖⊖ **Very low**[e,g]	4.5 (2 to 6)	the evidence is very uncertain about the effect on IADL independence

a: calculated from the estimated SMD using a standard deviation of 2.62, the pooled standard deviation across intervention groups reporting the Lawton IADL.

b: serious concerns about risk of bias due to missing outcome data. Downgrade once.

c: very serious concerns about imprecision as confidence interval includes substantial benefit and harm (SMD +/- 0.05). Downgrade twice.

d: very serious concerns about risk of bias because of missing data and multiple analyses being conducted but the results from only one analysis reported in the indirect evidence via the comparisons of homecare and multifactorial-action (hmcr & mfa-) vs homecare, ADL, multifactorial-action and review with self-management (hmcr & ADL & mfar(w/slfm)); and, homecare, ADL, multifactorial-action and review with self-management (hmcr & ADL & mfar(w/slfm)) vs homecare (hmcr). Downgrade twice.

e: very serious concerns about imprecision as confidence interval includes substantial benefit and harm (SMD +/- 0.05). Already downgraded twice for risk of bias, downgrade once.

f: very serious concerns about risk of bias because of missing data and multiple analyses being conducted but the results from only one analysis reported. Downgrade twice.

g: very serious concerns about risk of bias because of missing data and multiple analyses being conducted but the results from only one analysis reported in the in indirect evidence via homecare, ADL, multifactorial-action and review with self-management (hmcr & ADL & mfar(w/slfm)) vs homecare (hmcr). Downgrade twice.

Table 42 - Results of IADL: medium-term homecare network

**hmcr & mfar(w/med)**					0.15 (-0.11,0.40)
-0.03 (-0.78,0.71)	**hmcr & mfar**	0.19 (-0.06,0.44)			
0.16 (-0.49,0.80)	0.19 (-0.18,0.56)	**hmcr & mfa-**		-0.17 (-0.54,0.20)	
-0.13 (-0.69,0.44)	-0.09 (-0.95,0.76)	-0.28 (-1.05,0.49)	**hmcr & aids**		0.27 (-0.14,0.69)
-0.01 (-0.46,0.44)	0.02 (-0.57,0.61)	-0.17 (-0.63,0.29)	0.11 (-0.51,0.73)	**hmcr & ADL & mfar(w/slfm)**	0.16 (-0.08,0.40)
0.15 (-0.11,0.41)	0.18 (-0.52,0.88)	-0.01 (-0.60,0.58)	0.27 (-0.23,0.77)	0.16 (-0.21,0.53)	**hmcr**

Table 43 - Intervention rankings for IADL: medium-term homecare network

Treatment	SUCRA	Pr(Best)	Mean Rank	LCI Rank	UCI Rank
hmcr & aids	69	41.2	2.6	1	6
hmcr & mfar	61.1	31.5	2.9	1	6
hmcr & ADL & mfar(w/slfm)	58.5	11.7	3.1	1	6
hmcr & mfar(w/med)	55.5	13.1	3.2	1	6
hcmr & mfa-	30.2	2.2	4.5	2	6
hmcr	25.7	0.3	4.7	2	6

SUCRA values (0–100) and mean ranks are presented, based on 1000 simulations. Higher SUCRAs and lower mean ranks indicate better performing interventions. Pr(Best) gives the probability of each specific intervention being ranked best intervention, based on 1000 simulations.

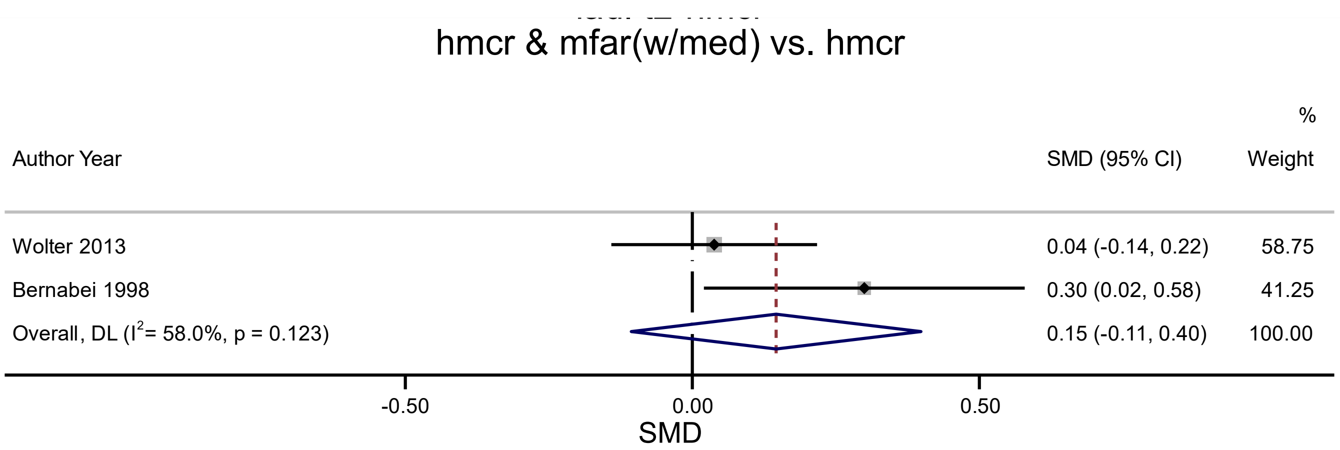

Figure 17 - Pairwise meta-analysis for IADL: medium-term homecare network (pooling comparisons with greater than one study reporting results)

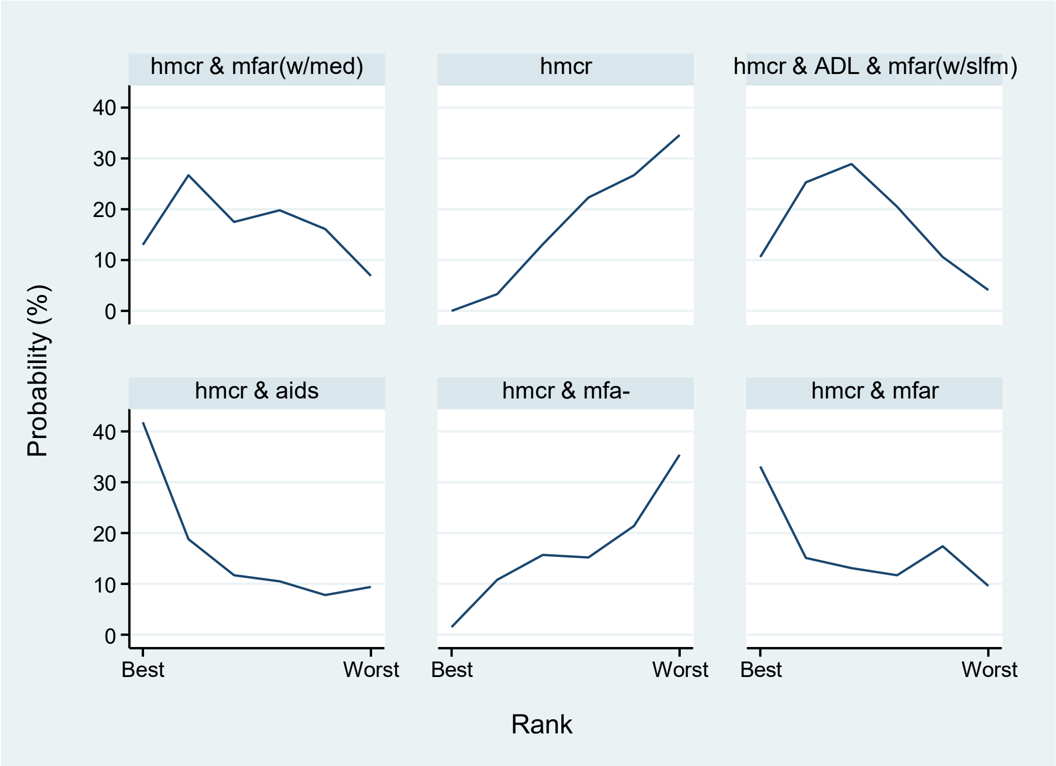

Figure 18 - Rankogram showing comparative effectiveness of interventions for IADL medium-term homecare network. Results based on a simulation of 1000 replications.

11.3.6 IADL homecare network, long-term timeframe

No results as there were too few comparisons to conduct network meta-analysis.

11.4 Personal ADL

11.4.1 PADL available care network, short-term timeframe

Table 44 - Short-term PADL available-care network

Study	Frailty	n	Experimental group	Control group	ROB					
					D1	D2	D3	D4	D5	Overall
**Bleijenberg 2016**[509]	pre-frail and frail	2754	rsk-mfa-	ac	x/+	-	x	+	-	xx
**Clark 1997**[519]	robust and pre-frail	303	eng & educ	ac	x	-	x	-	x	xx
**Gitlin 2006**[539]	pre-frail and frail	300	ADL & aids & exrc	ac	+	-	x	-	-	x
**Metzelthin 2013**[576]	frail	316	educ & mfar(w/med+slfm)	ac	-/-	-	x	-	-	x
**Rockwood 2000**[592]	frail	148	mfa-(w/med)	ac	-	-	x	-	-	x
**Szanton 2011**[606]	pre-frail and frail	40	ADL&aids&educ&exrc& mfar(w/med+slfm)	ac	-	-	x	-	-	x
**Takahashi 2012**[608]	frail	166	mntr-mfa-	ac	-	-	x	-	-	x
**Walters 2017**[626]	pre-frail	48	mfar(w/slfm)	ac	+	-	x	-	x	xx

n: number of participants. ROB: risk of bias. D#: Domain #. D1: risk of bias arising from the randomisation process (individual); or, for cluster trials, risk of bias arising from the randomisation process / risk of bias arising from the identification or recruitment of participants into clusters. D2: risk of bias due to deviations from the intended interventions (effect of assignment to the intervention). D3: risk of bias due to missing outcome data. D4: risk of bias in measurement of the outcome. D5: risk of bias in selection of the reported result. +: low risk of bias; -: some concerns; x: high risk of bias / serious concerns; xx: very serious concerns (overall risk of bias only). all: robust, pre-frail and frail.

Table 45 - PADL in the short term: comparisons with available care summary of findings table

Population: Older people

Interventions: Community-based complex interventions

Comparator: Available care (ac)

Outcome: independence in personal activities of daily living

Timeframe: short term; range of follow up 24 weeks to 9 months

Setting: Community

Total studies: 8

Comparator rank: Mean 5.6, 95% CI 4 to 8

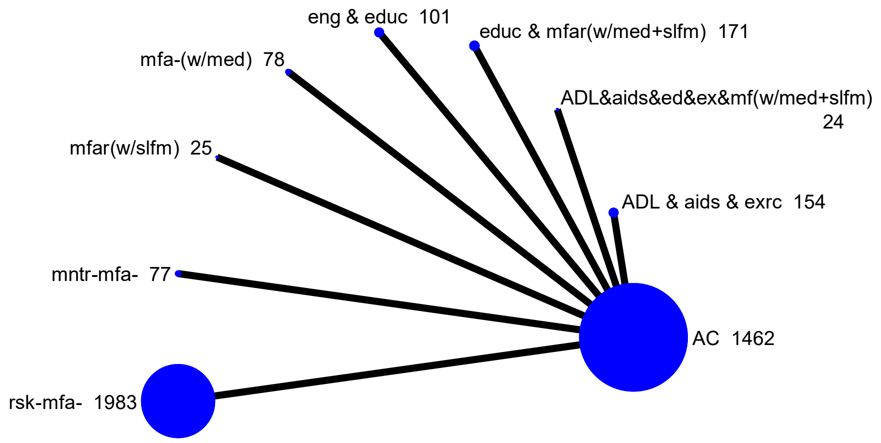

Intervention group	Anticipated absolute effect (95% CI) SMD	MD (Barthel Index 0 to 100)[a]	Certainty of the evidence (GRADE)	Ranking (95% CI)	Interpretation
Education, multifactorial-action and review with medication-review and self-management (educ & mfar(w/med+slfm))	SMD 0.25 lower (0.47 lower to 0.03 lower) Mixed estimate	MD 7.34 lower (13.91 lower to 0.76 lower)	⊕⊕⊖⊖ Low[b,c]	8.4 (6 to 9)	may result in a slight reduction in PADL independence
ADL, aids, education, exercise, multifactorial-action and review with medication-review and self-management (ADL&aids&ed&ex&mf(w/med+slfm))	SMD 0.87 higher (0.21 higher to 1.54 higher) Mixed estimate	MD 25.81 higher (6.17 higher to 45.46 higher)	⊕⊖⊖⊖ Very low[b,d]	1.4 (1 to 2)	the evidence is very uncertain about the effect on PADL independence
Multifactorial-action and review with self-management (mfar(w/slfm))	SMD 0.67 higher (0.09 higher to 1.26 higher) Mixed estimate	MD 19.94 higher (2.67 higher to 37.21 higher)	⊕⊖⊖⊖ Very low[e,f]	1.8 (1 to 4)	the evidence is very uncertain about the effect on PADL independence
ADL, aids and exercise (ADL & aids & exrc)	SMD 0.14 higher (0.09 lower to 0.36 higher) Mixed estimate	MD 4.03 higher (2.68 lower to 10.74 higher)	⊕⊖⊖⊖ Very low[b,g]	3.7 (2 to 7)	the evidence is very uncertain about the effect on PADL independence
Risk-screening (rsk-mfa-)	SMD 0.03 higher (0.06 lower to 0.11 higher) Mixed estimate	MD 0.75 higher (1.71 lower to 3.21 higher)	⊕⊖⊖⊖ Very low[h,i]	4.9 (3 to 7)	the evidence is very uncertain about the effect on PADL independence
Meaningful-activities and education (eng & educ)	SMD 0.00 (0.24 lower to 0.24 higher) Mixed estimate	MD 0.00 (7.07 lower to 7.07 higher)	⊕⊖⊖⊖ Very low[i,j]	5.5 (3 to 9)	the evidence is very uncertain about the effect on PADL independence
Monitoring (mntr-mfa-)	SMD 0.09 lower (0.40 lower to 0.21 higher) Mixed estimate	MD 2.76 lower (11.79 lower to 6.28 higher)	⊕⊖⊖⊖ Very low[b,d]	6.7 (3 to 9)	the evidence is very uncertain about the effect on PADL independence
Multifactorial-action with medication-review (mfa-(w/med))	SMD 0.11 lower (0.44 lower to 0.21 higher) Mixed estimate	MD 3.38 lower (12.94 lower to 6.18 higher)	⊕⊖⊖⊖ Very low[b,d]	7.0 (3 to 9)	the evidence is very uncertain about the effect on PADL independence

a: calculated from the estimated SMD using a standard deviation of 29.6, the pooled standard deviation across intervention groups reporting the Barthel Index.

b: serious concerns about risk of bias due to missing outcome data. Downgrade once.

c: serious concerns about imprecision as there is no closed loop and direct evidence is based on 316 persons which does not meet optimal information size. Downgrade once.

d: very serious concerns about imprecision as there is no closed loop and direct evidence is based on 40 persons which does not meet optimal information size. Downgrade twice.

e: very serious concerns about risk of bias because of missing data and multiple analyses being conducted but the results from only one analysis reported. Downgrade twice.

f: very serious concerns about imprecision as there is no closed loop and direct evidence is based on 48 persons which does not meet optimal information size. Already downgraded twice, downgrade once.

g: very serious concerns about imprecision as confidence interval includes substantial benefit and harm (SMD +/- 0.05). Downgrade twice.

h: very serious concerns about risk of bias due to randomisation process and missing outcome data. Downgrade twice.

i: very serious concerns about imprecision as confidence interval includes substantial benefit and harm (SMD +/- 0.05). Already downgraded twice for risk of bias, downgrade once.

j: very serious concerns about risk of bias due to randomisation process, missing outcome data, and reported results not being analysed according to allocation. Downgrade twice.

Table 46 - Results of PADL: short-term available care network

**rsk-mfa-**								0.03 (-0.06,0.11)
0.12 (-0.20,0.43)	**mntr-mfa-**							-0.09 (-0.40,0.21)
-0.65 (-1.24,-0.06)	-0.77 (-1.42,-0.11)	**mfar(w/slfm)**						0.67 (0.09,1.26)
0.14 (-0.19,0.47)	0.02 (-0.42,0.47)	0.79 (0.12,1.45)	**mfa-(w/med)**					-0.11 (-0.44,0.21)
0.03 (-0.23,0.28)	-0.09 (-0.48,0.29)	0.67 (0.04,1.30)	-0.11 (-0.52,0.29)	**eng & educ**				0.00 (-0.24,0.24)
0.27 (0.04,0.51)	0.15 (-0.22,0.53)	0.92 (0.30,1.55)	0.13 (-0.26,0.53)	0.25 (-0.08,0.57)	**educ & mfar(w/med+slfm)**			-0.25 (-0.47,-0.03)
-0.84 (-1.51,-0.17)	-0.96 (-1.69,-0.23)	-0.20 (-1.08,0.69)	-0.98 (-1.72,-0.24)	-0.87 (-1.58,-0.16)	-1.12 (-1.82,-0.42)	**ADL&aids&ed&ex&mf(w/med+slfm)**		0.87 (0.20,1.53)
-0.11 (-0.35,0.13)	-0.23 (-0.61,0.15)	0.54 (-0.09,1.16)	-0.25 (-0.64,0.14)	-0.14 (-0.47,0.19)	-0.38 (-0.70,-0.07)	0.73 (0.03,1.43)	**ADL & aids & exrc**	0.14 (-0.09,0.36)
0.03 (-0.06,0.11)	-0.09 (-0.40,0.21)	0.67 (0.09,1.26)	-0.11 (-0.44,0.21)	0.00 (-0.24,0.24)	-0.25 (-0.47,-0.03)	0.87 (0.20,1.53)	0.14 (-0.09,0.36)	**ac**

Lower left triangle presents the findings (SMD with 95% CI) of the network meta-analysis. Upper right triangle presents the findings (SMD with 95% CI) of pairwise meta-analyses. A SMD>1 favours the upper left intervention; a SMD<1 favours the lower right intervention. Within the table, comparisons between treatments should be read from left to right (i.e. treatment 1 versus treatment 2). The estimate effect measure (SMD and their 95% CI) is in the cell in common between the row- and column-defining treatment.

Table 47 - Intervention rankings for PADL: short-term available care network

Treatment	SUCRA	Pr(Best)	Mean Rank	LCI Rank	UCI Rank
ADL&aids&ed&ex&mf(w/med+slfm)	95.1	67.2	1.4	1	2
mfar(w/slfm)	89.8	32.3	1.8	1	4
ADL & aids & exrc	66.3	0.3	3.7	2	7
rsk-mfa-	50.8	0.0	4.9	3	7
eng & educ	44.0	0.1	5.5	3	9
ac	42.5	0.0	5.6	4	8
mntr-mfa-	28.3	0.0	6.7	3	9
mfa-(w/med)	25.6	0.1	7.0	3	9
educ & mfar(w/med+slfm)	7.5	0.0	8.4	6	9

SUCRA values (0–100) and mean ranks are presented, based on 1000 simulations. Higher SUCRAs and lower mean ranks indicate better performing interventions. Pr(Best) gives the probability of each specific intervention being ranked best intervention, based on 1000 simulations.

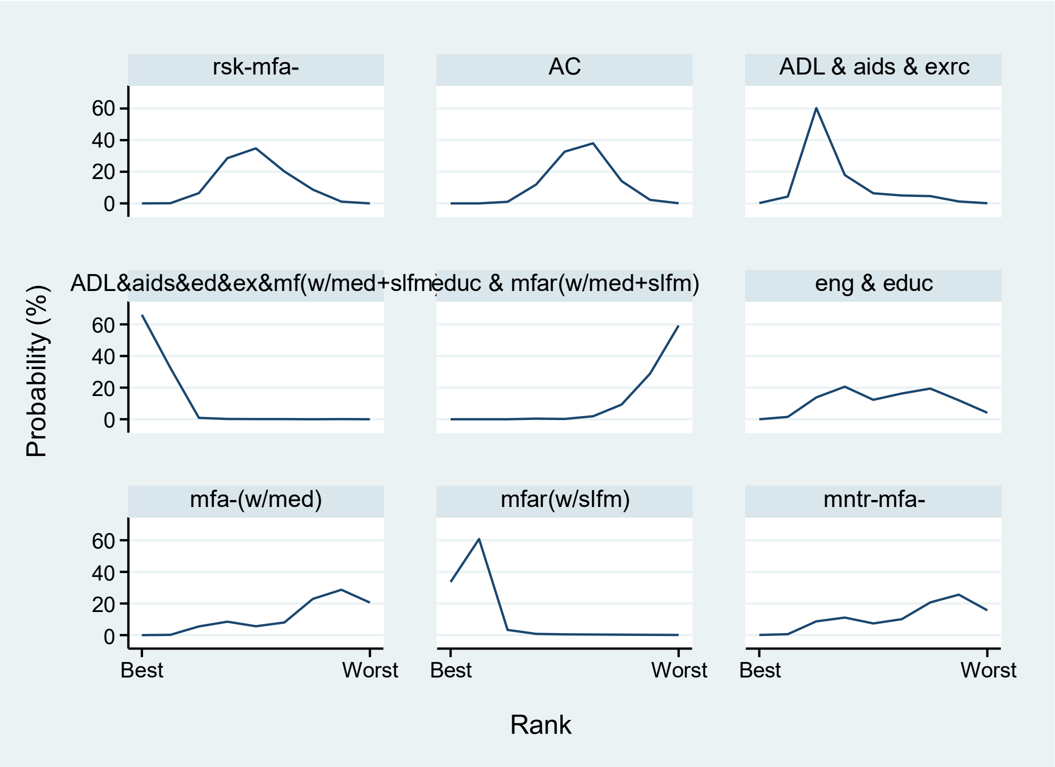

Figure 19 - Rankogram showing comparative effectiveness of interventions for PADL short-term available care network. Results based on a simulation of 1000 replications.

11.4.2 PADL available care network, medium-term timeframe

Table 48 - Medium-term PADL available-care network

					ROB					
**Study**	**Frailty**	**n**	**Experimental group**	**Control group**	**D1**	**D2**	**D3**	**D4**	**D5**	**Overall**
**Bleijenberg 2016[509]**	pre-frail and frail	2489	rsk-mfa-	ac	x/+	-	x	+	-	xx
**Blom 2016[510]**	all	1379	mfa-(w/med+slfm)	ac	x/+	-	x	-	-	xx
**Bouman 2008[513]**	pre-frail and frail	293	mfar(w/med)	ac	+	-	x	-	-	x
**Brettschneider 2015[514]**	frail	262	mfar(w/med)	ac	-	-	x	-	+	x
**Cameron 2013[515]**	frail	214	exrc & mfar(w/med+slfm)	ac	+	-	-	-	+	-
**Clark 1997[519]**	robust and pre-frail	281	eng & educ	ac	x	-	x	-	x	xx
**Dorresteijn 2016[526]**	unclassifiable	312	ADL	ac	+	-	x	-	-	x
**Fabacher 1994[528]**	all	195	mfar(w/med)	ac	-	-	x	-	-	x
**Gene Huguet 2018[536]**	pre-frail	173	med & ntr & exrc	ac	-	-	x	-	-	x
**Henderson 2005[548]**	robust	124	mfar	ac	+/x	+	x	+	-	xx
**Kono 2016[558]**	pre-frail	360	mfar(w/med)	mfar	+	-	x	-	-	x
**Metzelthin 2013[576]**	frail	317	educ & mfar(w/med+slfm)	ac	-/-	-	x	-	-	x
**Monteserin Nadal 2008[578]**	all	620	educ & rsk-mfa-	ac	-	-	x	x	-	xx
**Newbury 2001[582]**	unclassifiable	89	mfa-(w/med)	ac	-	-	x	-	-	x
**Rockwood 2000[592]**	frail	145	mfa-(w/med)	ac	-	-	x	-	-	x
**Rubenstein 2007[595]**	frail	694	mfar(w/med)	ac	-	-	-	-	-	-
**Serra-Prat 2017[597]**	pre-frail	133	ntr & exrc	ac	-	-	x	-	-	x
**Szanton 2019[607]**	pre-frail and frail	260	ADL&aids&educ&exrc& mfar(w/med+slfm)	ac	+	-	x	-	-	x
**Takahashi 2012[608]**	frail	166	mntr-mfa-	ac	-	-	x	-	-	x
**van Heuvelen 2005[617]**	pre-frail and frail	77	exrc & psyc	ac	-	x	x	-	-	xx

n: number of participants. ROB: risk of bias. D#: Domain #. D1: risk of bias arising from the randomisation process (individual); or, for cluster trials, risk of bias arising from the randomisation process / risk of bias arising from the identification or recruitment of participants into clusters. D2: risk of bias due to deviations from the intended interventions (effect of assignment to the intervention). D3: risk of bias due to missing outcome data. D4: risk of bias in measurement of the outcome. D5: risk of bias in selection of the reported result. +: low risk of bias; -: some concerns; x: high risk of bias / serious concerns; xx: very serious concerns (overall risk of bias only). all: robust, pre-frail and frail.

Table 49 - PADL in the medium term: comparisons with available care summary of findings table

Population: Older people

Interventions: Community-based complex interventions

Comparator: Available care (ac)

Outcome: personal activities of daily living (higher is better)

Timeframe: medium term; range of follow up 10 to 18 months

Setting: Community

Total studies: 20

Total participants: 8583

Comparator rank: Mean 9.2, 95% CI 6 to 12

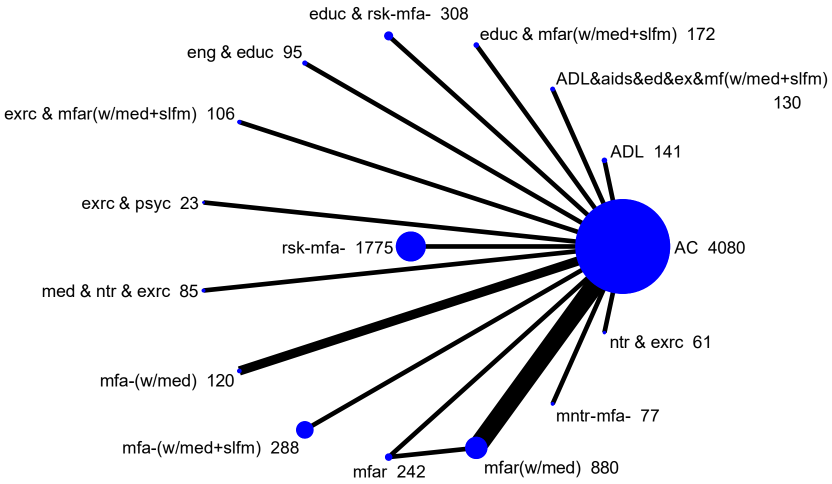

Intervention group	Anticipated absolute effect (95% CI)		Certainty of the evidence (GRADE)	Ranking (95% CI)	Interpretation
	**SMD**	**MD (Barthel Index 0 to 100)[a]**			
Exercise, multifactorial-action and review with medication-review and self-management (exrc & mfar(w/med+slfm))	SMD 0.16 higher (0.51 lower to 0.82 higher) Mixed estimate	MD 4.68 higher (15.01 lower to 24.37 higher)	⊕⊕⊖⊖ **Low**[b]	6.6 (1 to 16)	may result in a very slight increase in PADL independence
Multifactorial-action with medication-review (mfa-(w/med))	SMD 0.51 higher (0.00 to 1.02 higher) Mixed estimate	MD 15.08 higher (0.14 lower to 30.30 higher)	⊕⊖⊖⊖ **Very low**[c,d,e]	2.9 (1 to 10)	the evidence is very uncertain about the effect on PADL independence
Medication-review, nutrition and exercise (med & ntr & exrc)	SMD 0.31 higher (0.37 lower to 0.99 higher) Mixed estimate	MD 9.27 higher (10.81 lower to 29.36 higher)	⊕⊖⊖⊖ **Very low**[b,c]	5.3 (1 to 15)	the evidence is very uncertain about the effect on PADL independence
ADL (ADL)	SMD 0.22 higher (0.42 lower to 0.87 higher) Mixed estimate	MD 6.64 higher (12.55 lower to 25.84 higher)	⊕⊖⊖⊖ **Very low**[b,c]	6.3 (1 to 15)	the evidence is very uncertain about the effect on PADL independence
Risk-screening (rsk-mfa-)	SMD 0.13 higher (0.48 lower to 0.75 higher) Mixed estimate	MD 3.86 higher (14.34 lower to 22.06 higher)	⊕⊖⊖⊖ **Very low**[f,g]	7.4 (1 to 16)	the evidence is very uncertain about the effect on PADL independence
ADL, aids, education, exercise, multifactorial-action and review with medication-review and self-management (ADL&aids&ed&ex&mf(w/med+slfm))	SMD 0.07 higher (0.59 lower to 0.73 higher) Mixed estimate	MD 2.07 higher (17.33 lower to 21.47 higher)	⊕⊖⊖⊖ **Very low**[b,c]	8.1 (1 to 16)	the evidence is very uncertain about the effect on PADL independence
Multifactorial-action and review with medication-review (mfar(w/med))	SMD 0.05 higher (0.26 lower to 0.36 higher) Mixed estimate	MD 1.40 higher (7.71 lower to 10.51 higher)	⊕⊖⊖⊖ **Very low**[b,c]	8.4 (3 to 14)	the evidence is very uncertain about the effect on PADL independence

Exercise and psychology (exrc & psyc)	SMD 0.00 (0.78 lower to 0.78 higher) Mixed estimate	MD 0.00 (23.09 lower to 23.09 higher)	⊕⊖⊖⊖ **Very low**[g,h]	9.0 (1 to 16)	the evidence is very uncertain about the effect on PADL independence
Nutrition and exercise (ntr & exrc)	SMD 0.00 (0.70 lower to 0.70 higher) Mixed estimate	MD 0.00 (20.65 lower to 20.65 higher)	⊕⊖⊖⊖ **Very low**[b,c]	9.1 (1 to 16)	the evidence is very uncertain about the effect on PADL independence
Multifactorial-action with medication-review and self-management (mfa-(w/med+slfm))	SMD 0.04 lower (0.66 lower to 0.58 higher) Mixed estimate	MD 1.18 lower (19.60 lower to 17.24 higher)	⊕⊖⊖⊖ **Very low**[f,g]	9.5 (2 to 16)	the evidence is very uncertain about the effect on PADL independence
Education and risk-screening (educ & rsk-mfa-)	SMD 0.03 lower (0.66 lower to 0.60 higher) Mixed estimate	MD 0.95 lower (19.56 lower to 17.66 higher)	⊕⊖⊖⊖ **Very low**[g,i]	9.6 (2 to 16)	the evidence is very uncertain about the effect on PADL independence
Meaningful-activities and education (eng & educ)	SMD 0.05 lower (0.71 lower to 0.61 higher) Mixed estimate	MD 1.42 lower (20.87 lower to 18.03 higher)	⊕⊖⊖⊖ **Very low**[g,j]	10.1 (2 to 16)	the evidence is very uncertain about the effect on PADL independence
Monitoring (mntr-mfa-)	SMD 0.17 lower (0.85 lower to 0.51 higher) Mixed estimate	MD 5.14 lower (25.30 lower to 15.02 higher)	⊕⊖⊖⊖ **Very low**[b,c]	10.9 (3 to 16)	the evidence is very uncertain about the effect on PADL independence
Multifactorial-action and review (mfar)	SMD 0.14 lower (0.65 lower to 0.36 higher) Mixed estimate	MD 4.21 lower (19.14 lower to 10.72 higher)	⊕⊖⊖⊖ **Very low**[b,c]	11.2 (3 to 16)	the evidence is very uncertain about the effect on PADL independence
Education, multifactorial-action and review with medication-review and self-management (educ & mfar(w/med+slfm))	SMD 0.27 lower (0.92 lower to 0.38 higher) Mixed estimate	MD 7.91 lower (27.09 lower to 11.27 higher)	⊕⊖⊖⊖ **Very low**[b,c]	12.5 (3 to 16)	the evidence is very uncertain about the effect on PADL independence

a: calculated from the estimated SMD using a standard deviation of 29.6, the pooled standard deviation across intervention groups reporting the Barthel Index.

b: very serious concerns about imprecision as confidence interval includes substantial benefit and harm (SMD +/- 0.05). Downgrade twice.

c: serious concerns about risk of bias due to missing outcome data. Downgrade once.

d: serious concerns about imprecision as confidence interval crosses the no effect line and includes substantial benefit. Downgrade once.

e: serious concerns about inconsistency (heterogeneity) between studies as confidence intervals do not overlap. Downgrade once.

f: very serious concerns about risk of bias due to randomisation process and missing outcome data. Downgrade twice.

g: very serious concerns about imprecision as confidence interval includes substantial benefit and harm (SMD +/- 0.05). Already downgraded twice for risk of bias, downgrade once.

h: very serious concerns about risk of bias due to excluding participants in per-protocol analysis and missing outcome data. Downgrade twice.

i: very serious concerns about risk of bias due to missing data and ceiling effect in the outcome measurement for a substantial proportion of participants. Downgrade twice.

j: very serious concerns about risk of bias due to randomisation process, missing outcome data, and reported results were not analysed according to allocation. Downgrade twice.

Table 50 - Results of PADL: medium-term available care network

**rsk-mfa-**															0.13 (0.04, 0.22)
0.13 (-0.80,1.06)	**ntr & exrc**														0.00 (-0.34, 0.34)
0.30 (-0.61,1.22)	0.17 (-0.80,1.15)	**mntr-mfa-**													-0.17 (-0.48, 0.13)
0.08 (-0.60,0.77)	-0.05 (-0.81,0.71)	-0.22 (-0.97,0.53)	**mfar(w/med)**	0.03 (-0.18, 0.24)											0.11 (0.00, 0.21)
0.27 (-0.52,1.07)	0.14 (-0.72,1.00)	-0.03 (-0.88,0.81)	0.19 (-0.31,0.68)	**mfar**											-0.33 (-0.69, 0.02)
0.17 (-0.70,1.04)	0.04 (-0.89,0.97)	-0.13 (-1.05,0.79)	0.09 (-0.61,0.78)	-0.10 (-0.90,0.70)	**mfa-(w/med+slfm)**										-0.04 (-0.17, 0.09)
-0.38 (-1.18,0.42)	-0.51 (-1.37,0.36)	-0.68 (-1.53,0.17)	-0.46 (-1.06,0.14)	-0.65 (-1.37,0.07)	-0.55 (-1.35,0.26)	**mfa-(w/med)**									0.55 (-0.59, 1.70)
-0.18 (-1.10,0.73)	-0.31 (-1.29,0.66)	-0.49 (-1.45,0.47)	-0.27 (-1.01,0.48)	-0.46 (-1.30,0.39)	-0.35 (-1.27,0.57)	0.20 (-0.65,1.05)	**med & ntr & exrc**								0.31 (0.01, 0.61)
0.13 (-0.86,1.12)	-0.00 (-1.05,1.05)	-0.17 (-1.21,0.86)	0.05 (-0.79,0.89)	-0.14 (-1.07,0.79)	-0.04 (-1.04,0.96)	0.51 (-0.42,1.44)	0.31 (-0.72,1.35)	**exrc & psyc**							0.00 (-0.49, 0.49)
-0.03 (-0.93,0.88)	-0.16 (-1.12,0.80)	-0.33 (-1.28,0.62)	-0.11 (-0.84,0.62)	-0.30 (-1.13,0.53)	-0.20 (-1.11,0.71)	0.35 (-0.49,1.19)	0.16 (-0.79,1.10)	-0.16 (-1.18,0.87)	**exrc & mfar(w/med +slfm)**						0.16 (-0.11, 0.43)
0.18 (-0.72,1.08)	0.05 (-0.91,1.00)	-0.13 (-1.07,0.82)	0.10 (-0.63,0.82)	-0.09 (-0.92,0.73)	0.01 (-0.90,0.91)	0.56 (-0.28,1.39)	0.36 (-0.58,1.30)	0.05 (-0.97,1.07)	0.21 (-0.73,1.14)	**eng & educ**					-0.05 (-0.30, 0.20)
0.16 (-0.72,1.04)	0.03 (-0.91,0.97)	-0.14 (-1.07,0.78)	0.08 (-0.62,0.78)	-0.11 (-0.91,0.69)	-0.01 (-0.89,0.88)	0.54 (-0.27,1.35)	0.35 (-0.58,1.27)	0.03 (-0.97,1.03)	0.19 (-0.72,1.10)	-0.02 (-0.92,0.89)	**educ & rsk-mfa-**				-0.03 (-0.19, 0.13)
0.40 (-0.49,1.29)	0.27 (-0.68,1.22)	0.09 (-0.84,1.03)	0.31 (-0.40,1.03)	0.13 (-0.69,0.94)	0.23 (-0.67,1.12)	0.78 (-0.05,1.60)	0.58 (-0.36,1.52)	0.27 (-0.75,1.28)	0.43 (-0.50,1.35)	0.22 (-0.70,1.14)	0.24 (-0.67,1.14)	**educ & mfar(w/med +slfm)**			-0.27 (-0.49, -0.05)
0.06 (-0.84,0.96)	-0.07 (-1.03,0.89)	-0.24 (-1.19,0.70)	-0.02 (-0.75,0.70)	-0.21 (-1.04,0.61)	-0.11 (-1.01,0.79)	0.44 (-0.39,1.27)	0.24 (-0.70,1.19)	-0.07 (-1.09,0.95)	0.09 (-0.84,1.02)	-0.12 (-1.04,0.81)	-0.10 (-1.01,0.80)	-0.34 (-1.26,0.58)	**ADL&aids& ed&ex&mf(w/med+slfm)**		0.07 (-0.17, 0.31)
-0.09 (-0.99,0.80)	-0.22 (-1.18,0.73)	-0.40 (-1.34,0.54)	-0.18 (-0.89,0.54)	-0.37 (-1.19,0.45)	-0.26 (-1.16,0.63)	0.28 (-0.54,1.11)	0.09 (-0.85,1.03)	-0.22 (-1.24,0.79)	-0.07 (-0.99,0.86)	-0.27 (-1.19,0.65)	-0.26 (-1.16,0.65)	-0.49 (-1.41,0.42)	-0.15 (-1.08,0.77)	**ADL**	0.22 (0.00, 0.45)
0.13 (-0.48,0.74)	0.00 (-0.70,0.70)	-0.17 (-0.85,0.51)	0.05 (-0.26,0.35)	-0.14 (-0.65,0.36)	-0.04 (-0.66,0.58)	0.51 (-0.01,1.02)	0.31 (-0.36,0.99)	0.00 (-0.78,0.78)	0.16 (-0.51,0.82)	-0.05 (-0.70,0.61)	-0.03 (-0.66,0.60)	-0.27 (-0.91,0.38)	0.07 (-0.58,0.72)	0.22 (-0.42,0.87)	**ac**

Lower left triangle presents the findings (SMD with 95% CI) of the network meta-analysis. Upper right triangle presents the findings (SMD with 95% CI) of pairwise meta-analyses. A SMD>1 favours the upper left intervention; a SMD<1 favours the lower right intervention. Within the table, comparisons between treatments should be read from left to right (i.e. treatment 1 versus treatment 2). The estimate effect measure (SMD and their 95% CI) is in the cell in common between the row- and column-defining treatment.

Table 51 - Intervention rankings for PADL: medium-term available care network

Treatment	SUCRA	Pr(Best)	Mean Rank	LCI Rank	UCI Rank
mfa-(w/med)	87.6	34.3	2.9	1	10
med & ntr & exrc	71.5	18.4	5.3	1	15
ADL	64.8	11	6.3	1	15
exrc & mfar(w/med+slfm)	62.5	9.7	6.6	1	16
rsk-mfa-	57.6	6.1	7.4	1	16
ADL&aids&ed&ex&mf(w/med+slfm)	52.4	4.2	8.1	1	16
mfar(w/med)	50.8	0.1	8.4	3	14
exrc & psyc	46.4	5.3	9	1	16
ntr & exrc	45.9	3.8	9.1	1	16
ac	45.1	0	9.2	6	12
mfa-(w/med+slfm)	43	1.9	9.5	2	16
educ & rsk-mfa-	42.9	1.5	9.6	2	16
eng & educ	39.4	1.7	10.1	2	16
mntr-mfa-	34.2	1	10.9	3	16
mfar	32.2	0.2	11.2	3	16
educ & mfar(w/med+slfm)	23.6	0.8	12.5	3	16

SUCRA values (0–100) and mean ranks are presented, based on 1000 simulations. Higher SUCRAs and lower mean ranks indicate better performing interventions. Pr(Best) gives the probability of each specific intervention being ranked best intervention, based on 1000 simulations.

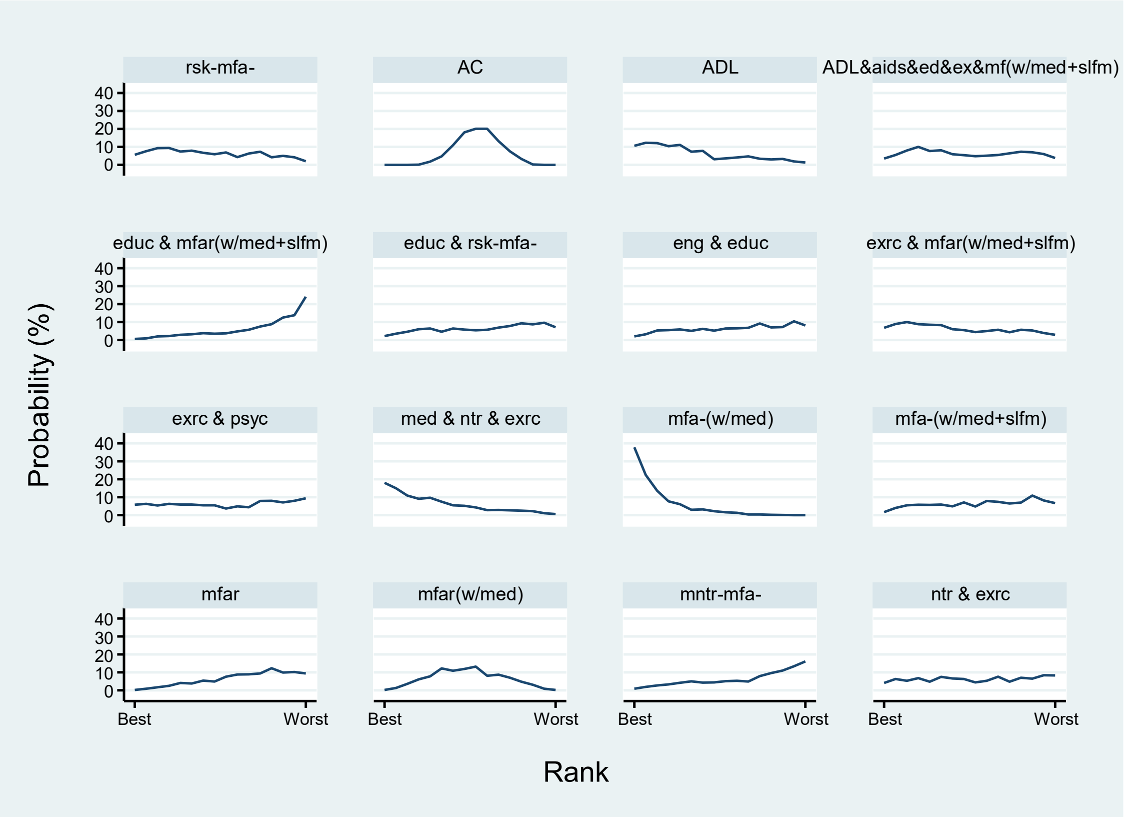

Figure 20 - Rankogram showing comparative effectiveness of interventions for PADL medium-term available care network. Results based on a simulation of 1000 replications.

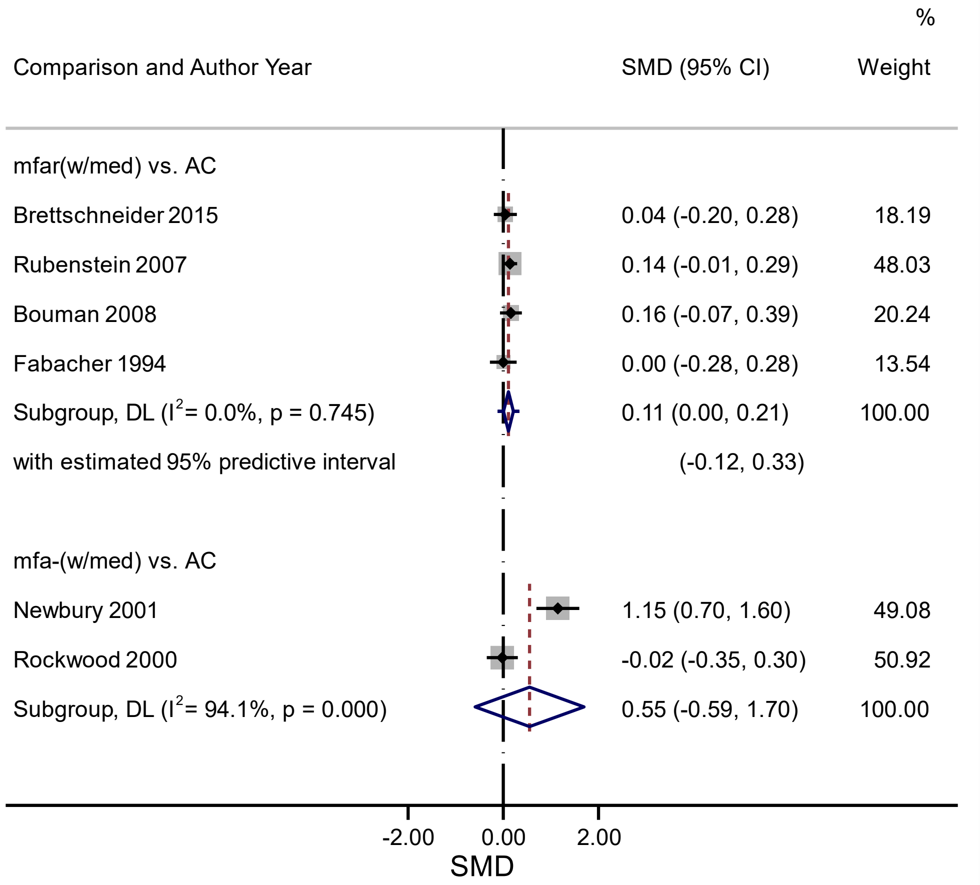

Figure 21 - Pairwise meta-analysis for PADL: medium-term available care network (pooling comparisons with greater than one study reporting results)

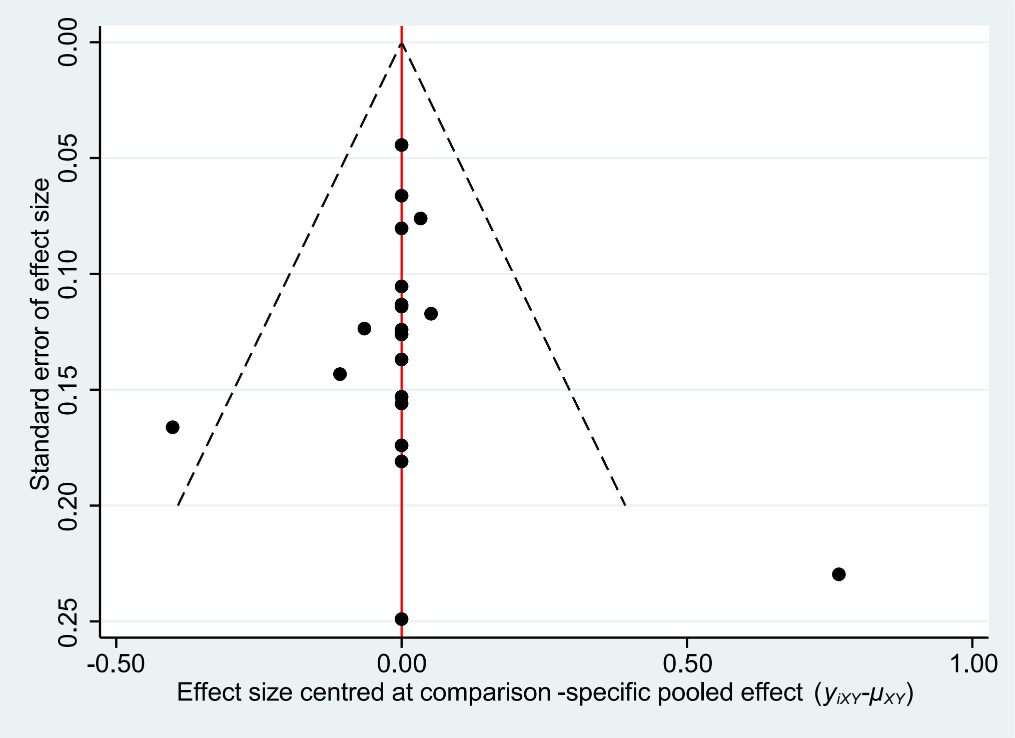

Figure 22 - Comparison-adjusted funnel plot for PADL medium-term timeframe

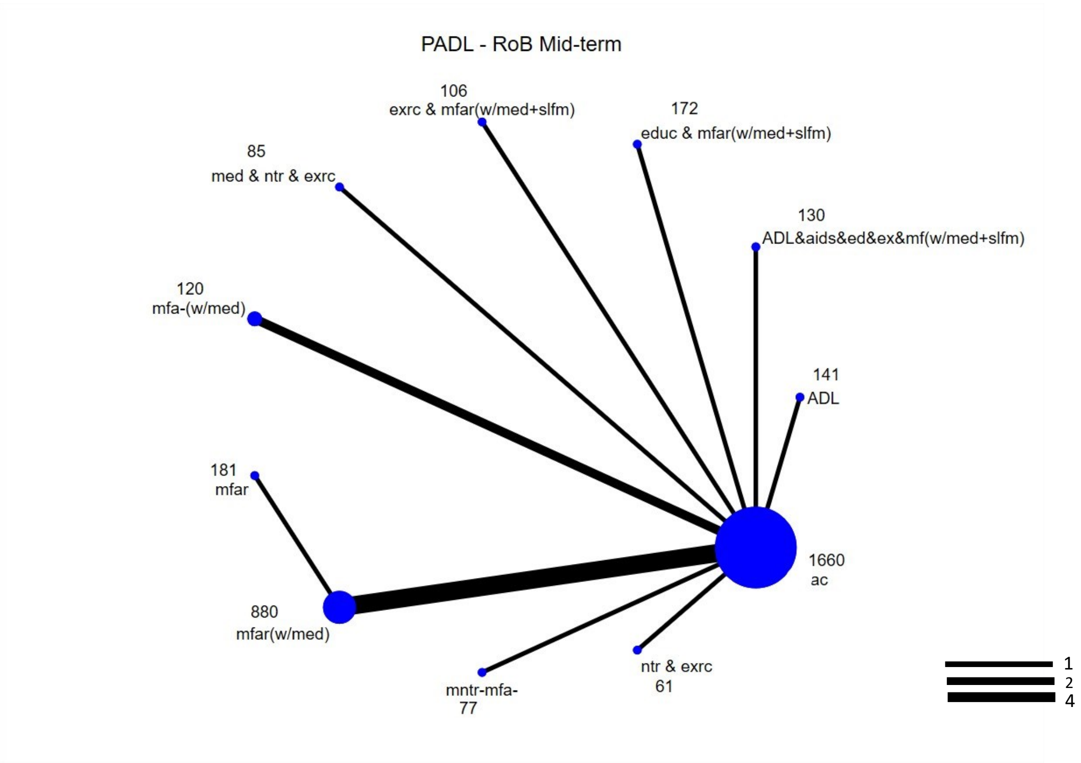

Figure 23 - Network plot for risk of bias analysis for PADL medium-term timeframe with available care (ac) comparator

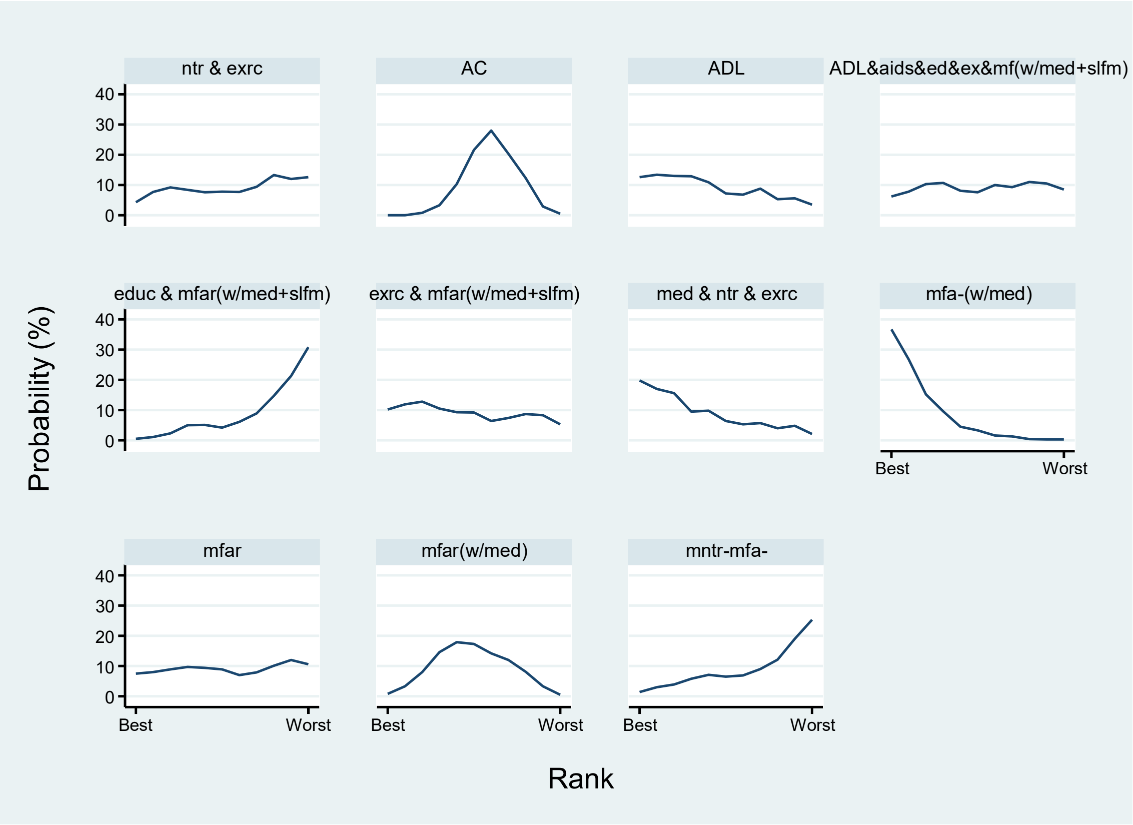

Figure 24 - Rankogram showing comparative effectiveness of interventions for risk of bias sensitivity analysis for PADL medium-term available care network. Results based on a simulation of 1000 replications.

11.4.3 PADL available care network, long-term timeframe

Table 52 - Long-term PADL available-care network

Study	Frailty	n	Experimental group	Control group	ROB D1	ROB D2	ROB D3	ROB D4	ROB D5	ROB Overall
**Balaban 1988**[506]	frail	86	mfa-(w/med)	ac	x	x	x	-	-	xx
**Bouman 2008**[513]	pre-frail and frail	293	mfar(w/med)	ac	+	-	x	-	-	x
**Jitapunkul 1998**[555]	unclassifiable	116	rsk-mfa-	ac	-	-	-	-	-	-
**Kono 2016**[558]	pre-frail	360	mfar(w/med)	mfar	+	-	x	-	-	x
**Metzelthin 2013**[576]	frail	316	educ & mfar(w/med+slfm)	ac	-/-	-	x	-	-	x
**Rubenstein 2007**[595]	frail	607	mfar(w/med)	ac	-	-	-	-	-	-
**Stuck 1995**[602]	all	317	educ & mfar(w/med)	ac	+	-	-	-	-	-

n: number of participants. ROB: risk of bias. D#: Domain #. D1: risk of bias arising from the randomisation process (individual); or, for cluster trials, risk of bias arising from the randomisation process / risk of bias arising from the identification or recruitment of participants into clusters. D2: risk of bias due to deviations from the intended interventions (effect of assignment to the intervention). D3: risk of bias due to missing outcome data. D4: risk of bias in measurement of the outcome. D5: risk of bias in selection of the reported result. +: low risk of bias; -: some concerns; x: high risk of bias / serious concerns; xx: very serious concerns (overall risk of bias only). all: robust, pre-frail and frail.

Table 53 - PADL in the long term: comparisons with available care summary of findings table

Population: Older people

Interventions: Community-based complex interventions

Comparator: Available care (ac)

Outcome: Independence in personal activities of daily living (PADL)

Timeframe: long term; range of follow up 24 to 36 months

Setting: Community

Total studies: 7

Total participants: 2095

Comparator rank: Mean 3.0, 95% CI 1 to 5

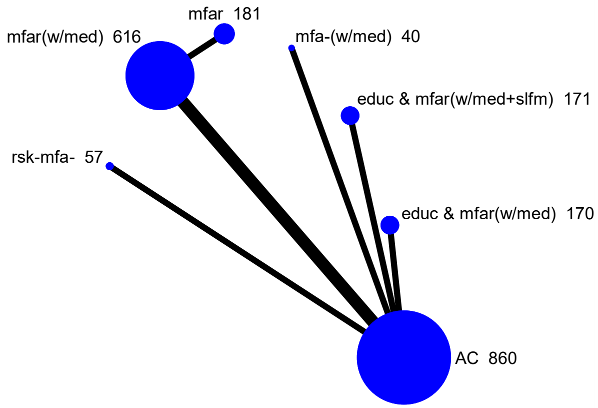

Intervention group	Anticipated absolute effect (95% CI) SMD	Anticipated absolute effect (95% CI) MD (Barthel Index 0 to 100)[a]	Certainty of the evidence (GRADE)	Ranking (95% CI)	Interpretation
Education, multifactorial-action and review with medication-review (educ & mfar(w/med))	SMD 0.11 higher (0.11 lower to 0.33 higher) Mixed estimate	MD 3.21 higher (3.33 lower to 9.75 higher)	⊕⊕⊖⊖ **Low**[b]	1.9 (1 to 5)	may result in a very slight increase in PADL independence
Risk-screening (rsk-mfa-)	SMD 0.06 higher (0.30 lower to 0.43 higher) Mixed estimate	MD 1.85 higher (8.92 lower to 12.63 higher)	⊕⊕⊖⊖ **Low**[b]	2.5 (1 to 6)	may result in a very slight increase in PADL independence
Multifactorial-action and review with medication-review (mfar(w/med))	SMD 0.03 lower (0.16 lower to 0.10 higher) Mixed estimate	MD 0.79 lower (4.66 lower to 3.08 higher)	⊕⊕⊖⊖ **Low**[b]	3.5 (1 to 5)	may result in little to no difference in PADL independence
Education, multifactorial-action and review with medication-review and self-management (educ & mfar(w/med+slfm))	SMD 0.27 lower (0.50 lower to 0.05 lower) Mixed estimate	MD 8.07 lower (14.65 lower to 1.49 lower)	⊕⊕⊖⊖ **Low**[c,d]	5.8 (4 to 7)	may result in a slight reduction in PADL independence
Multifactorial-action and review (mfar)	SMD 0.37 lower (0.62 lower to 0.13 lower) Indirect estimate	MD 11.06 lower (18.34 lower to 3.79 lower)	⊕⊕⊖⊖ **Low**[c,e]	6.5 (5 to 7)	may result in a slight reduction in PADL independence
Multifactorial-action with medication-review mfa-(w/med)	SMD 0.17 lower (0.60 lower to 0.25 higher) Mixed estimate	MD 5.09 lower (17.66 lower to 7.48 higher)	⊕⊖⊖⊖ **Very low**[f,g]	4.8 (1 to 7)	the evidence is very uncertain about the effect on PADL independence

a: calculated from the estimated SMD using a standard deviation of 29.6, the pooled standard deviation across intervention groups reporting the Barthel Index.

b: very serious concerns about imprecision as confidence interval includes substantial benefit and harm (SMD +/- 0.05). Downgrade twice.

c: serious concerns about risk of bias due to missing outcome data. Downgrade once.

d: serious concerns about imprecision as no closed loop and direct comparison is based on 316 persons which does not meet optimal information size. Downgrade once.

e: serious concerns about imprecision as no closed loop and comparison is based on 360 persons in link between multifactorial-action and review and multifactorial-action and review with medication-review which does not meet optimal information size. Downgrade once.

f: very serious concerns about risk of bias due to randomisation process, deviations from the intended interventions, and missing data. Downgrade twice.

g: very serious concerns about imprecision as confidence interval includes substantial benefit and harm (SMD +/- 0.05). Already downgraded twice for risk of bias, downgrade once.

Table 54 - Results of PADL: long-term available care network

**rsk-mfa-**						0.06 (-0.30, 0.43)
0.09 (-0.30,0.48)	**mfar(w/med)**	0.35 (0.14, 0.56)				-0.03 (-0.16, 0.10)
0.44 (-0.00,0.88)	0.35 (0.14,0.56)	**mfar**				
0.23 (-0.32,0.79)	0.15 (-0.30,0.59)	-0.20 (-0.69,0.29)	**mfa-(w/med)**			-0.17 (-0.60, 0.25)
0.34 (-0.09,0.76)	0.25 (-0.01,0.50)	-0.10 (-0.43,0.23)	0.10 (-0.38,0.58)	**educ & mfar(w/med+slfm)**		-0.27 (-0.50, -0.05)
-0.05 (-0.47,0.38)	-0.14 (-0.39,0.12)	-0.48 (-0.81,-0.15)	-0.28 (-0.76,0.20)	-0.38 (-0.69,-0.07)	**educ & mfar(w/med)**	0.11 (-0.11, 0.33)
0.06 (-0.30,0.43)	-0.03 (-0.16,0.10)	-0.37 (-0.62,-0.13)	-0.17 (-0.60,0.25)	-0.27 (-0.50,-0.05)	0.11 (-0.11,0.33)	**AC**

Lower left triangle presents the findings (SMD with 95% CI) of the network meta-analysis. Upper right triangle presents the findings (SMD with 95% CI) of pairwise meta-analyses. A SMD>1 favours the upper left intervention; a SMD<1 favours the lower right intervention. Within the table, comparisons between treatments should be read from left to right (i.e. treatment 1 versus treatment 2). The estimate effect measure (SMD and their 95% CI) is in the cell in common between the row- and column-defining treatment.

Table 55 - Intervention rankings for PADL: long-term available care network

Treatment	SUCRA	Pr(Best)	Mean Rank	LCI Rank	UCI Rank
educ & mfar(w/med)	85.4	46.2	1.9	1	5
rsk-mfa-	74.8	38.7	2.5	1	6
ac	65.9	3.4	3.0	1	5
mfar(w/med)	58.9	4.5	3.5	1	5
mfa-(w/med)	36.8	7.2	4.8	1	7
educ & mfar(w/med+slfm)	20.1	0.0	5.8	4	7
mfar	8.2	0.0	6.5	5	7

SUCRA values (0–100) and mean ranks are presented, based on 1000 simulations. Higher SUCRAs and lower mean ranks indicate better performing interventions. Pr(Best) gives the probability of each specific intervention being ranked best intervention, based on 1000 simulations.

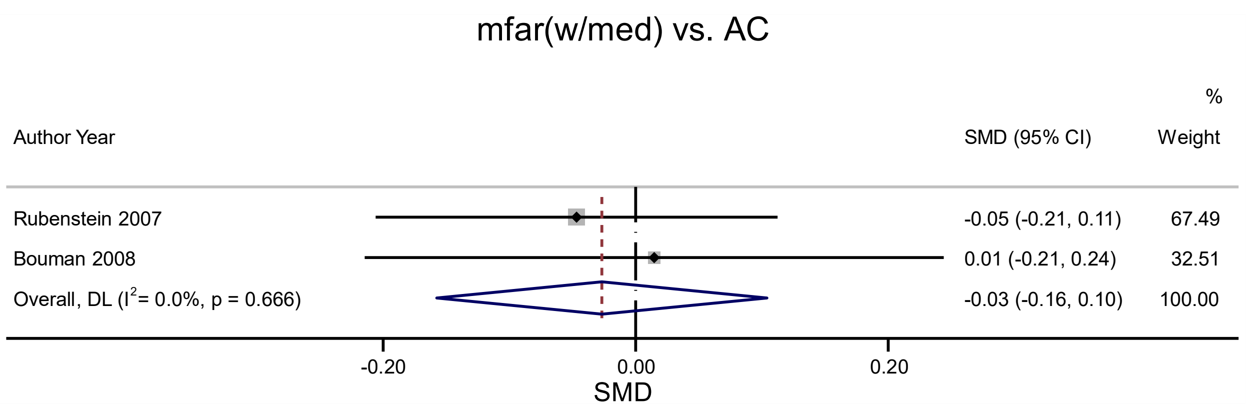

Figure 25 - Pairwise meta-analysis for PADL: long-term available care network (pooling comparisons with greater than one study reporting results)

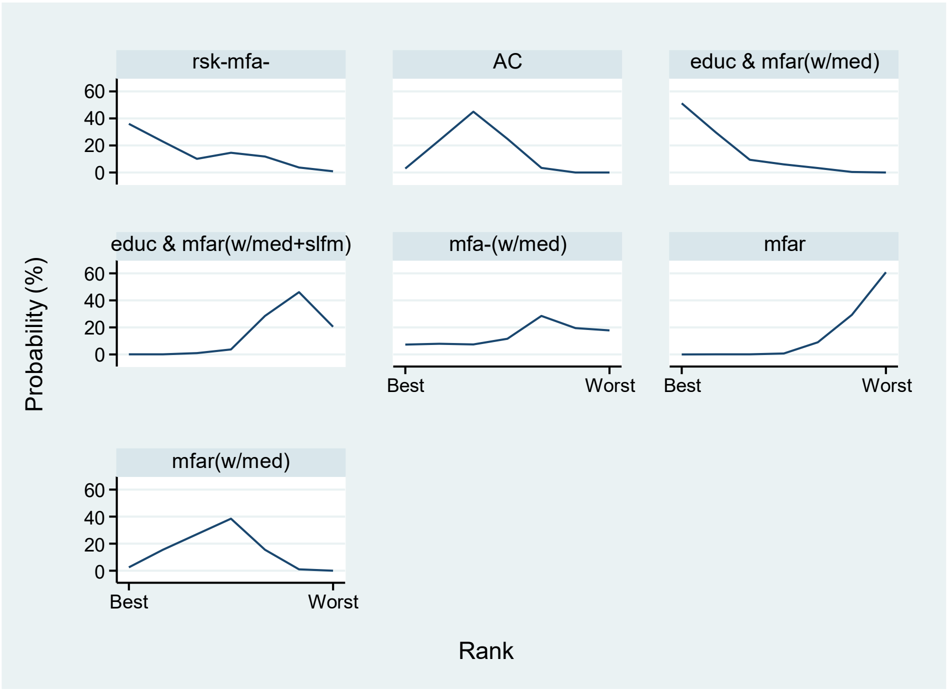

Figure 26 - Rankogram showing comparative effectiveness of interventions for PADL long-term available care network. Results based on a simulation of 1000 replications.

11.4.4 PADL homecare network, short-term timeframe

Table 56 - Short-term PADL homecare network

Study	Frailty	n	Experimental group	Control group	ROB D1	D2	D3	D4	D5	Overall
**Auvinen 2020**[505]	frail	449	hmcr & med	hmcr	+	-	-	-	x	x
**Fernandez-Barres 2017**[531]	frail	111	hmcr & ntr	hmcr	+	-	x	-	-	x
**King 2012**[557]	pre-frail and frail	157	hmcr & ADL & mfar(w/slfm)	hmcr	+/+	-	-	-	-	-
**Teut 2013**[609]	frail	58	hmcr & hmnt & exrc	hmcr	+/+	-	x	-	-	x

n: number of participants. ROB: risk of bias. D#: Domain #. D1: risk of bias arising from the randomisation process (individual); or, for cluster trials, risk of bias arising from the randomisation process / risk of bias arising from the identification or recruitment of participants into clusters. D2: risk of bias due to deviations from the intended interventions (effect of assignment to the intervention). D3: risk of bias due to missing outcome data. D4: risk of bias in measurement of the outcome. D5: risk of bias in selection of the reported result. +: low risk of bias; -: some concerns; x: high risk of bias / serious concerns; xx: very serious concerns (overall risk of bias only). all: robust, pre-frail and frail.

Table 57 - PADL in the short term: comparisons with homecare summary of findings table

Population: Older people

Interventions: Community-based complex interventions

Comparator: Homecare (hmcr)

Outcome: Independence in personal activities of daily living (PADL)

Timeframe: short term; range of follow up 6 to 7 months

Setting: Community

Total studies: 4

Total participants: 775

Comparator rank: Mean 3.4, 95% CI 2 to 5

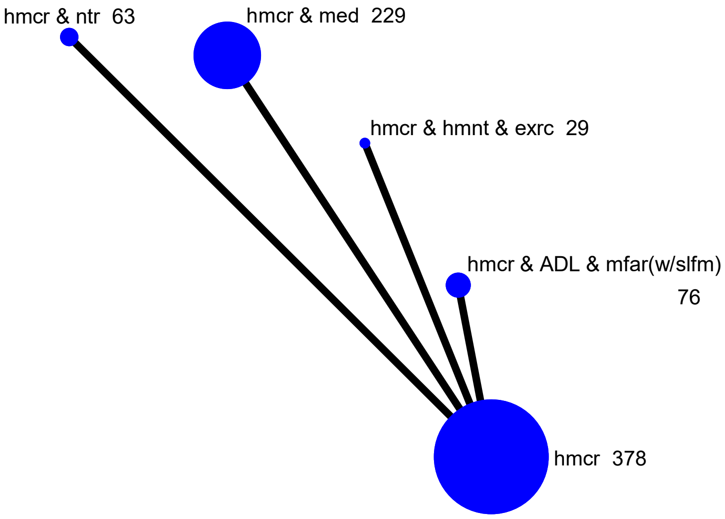

Intervention group	Anticipated absolute effect (95% CI): SMD	Anticipated absolute effect (95% CI): MD (Barthel Index 0 to 100)[a]	Certainty of the evidence (GRADE)	Ranking (95% CI)	Interpretation
Homecare, ADL, multifactorial-action and review with self-management (hmcr & ADL & mfar(w/slfm))	SMD 0.11 higher (0.20 lower to 0.43 higher) Mixed estimate	MD 3.32 higher (5.95 lower to 12.59 higher)	⊕⊕⊖⊖ **Low**[b]	2.4 (1 to 5)	may result in a very slight increase in PADL independence
Homecare and nutrition (hmcr & ntr)	SMD 0.13 higher (0.24 lower to 0.51 higher) Mixed estimate	MD 3.92 higher (7.20 lower to 15.05 higher)	⊕⊖⊖⊖ **Very low**[b,c]	2.3 (1 to 5)	the evidence is very uncertain about the effect on PADL independence
Homecare, alternative-medicine and exercise (hmcr & hmnt & exrc)	SMD 0.03 higher (0.48 lower to 0.55 higher) Mixed estimate	MD 0.91 higher (14.32 lower to 16.15 higher)	⊕⊖⊖⊖ **Very low**[b,c]	3.0 (1 to 5)	the evidence is very uncertain about the effect on PADL independence
Homecare and medication-review (hmcr & med)	SMD 0.05 lower (0.23 lower to 0.14 higher) Mixed estimate	MD 1.44 lower (6.92 lower to 4.03 higher)	⊕⊖⊖⊖ **Very low**[b,d]	3.9 (2 to 5)	the evidence is very uncertain about the effect on PADL independence

a: calculated from the estimated SMD using a standard deviation of 29.6, the pooled standard deviation across intervention groups reporting the Barthel Index.

b: very serious concerns about imprecision as confidence interval includes substantial benefit and harm (SMD +/- 0.05). Downgrade twice.

c: serious concerns about risk of bias due to missing outcome data. Downgrade once.

d: serious concerns about risk of bias because multiple analyses were conducted but the results from only one analysis were reported. Downgrade once.

Table 58 - Results of PADL: short-term homecare network

**hmcr & ntr**				0.13 (-0.24,0.51)
0.18 (-0.24,0.60)	**hmcr & med**			-0.05 (-0.23,0.14)
0.10 (-0.54,0.74)	-0.08 (-0.63,0.47)	**hmcr & hmnt & exrc**		0.03 (-0.48,0.55)
0.02 (-0.47,0.51)	-0.16 (-0.52,0.20)	-0.08 (-0.68,0.52)	**hmcr & ADL & mfar(w/slfm)**	0.11 (-0.20,0.43)
0.13 (-0.24,0.51)	-0.05 (-0.23,0.14)	0.03 (-0.48,0.55)	0.11 (-0.20,0.43)	**hmcr**

Lower left triangle presents the findings (SMD with 95% CI) of the network meta-analysis. Upper right triangle presents the findings (SMD with 95% CI) of pairwise meta-analyses. A SMD>1 favours the upper left intervention; a SMD<1 favours the lower right intervention. Within the table, comparisons between treatments should be read from left to right (i.e. treatment 1 versus treatment 2). The estimate effect measure (SMD and their 95% CI) is in the cell in common between the row- and column-defining treatment.

Table 59 - Intervention rankings for PADL: short-term homecare network

Treatment	SUCRA	Pr(Best)	Mean Rank	LCI Rank	UCI Rank
hmcr & ntr	66.7	37.9	2.3	1	5
hmcr & ADL & mfar(w/slfm)	66	29.2	2.4	1	5
hmcr & hmnt & exrc	50.1	28.5	3	1	5
hmcr	40.4	1.8	3.4	2	5
hmcr & med	26.8	2.6	3.9	2	5

SUCRA values (0–100) and mean ranks are presented, based on 1000 simulations. Higher SUCRAs and lower mean ranks indicate better performing interventions. Pr(Best) gives the probability of each specific intervention being ranked best intervention, based on 1000 simulations.

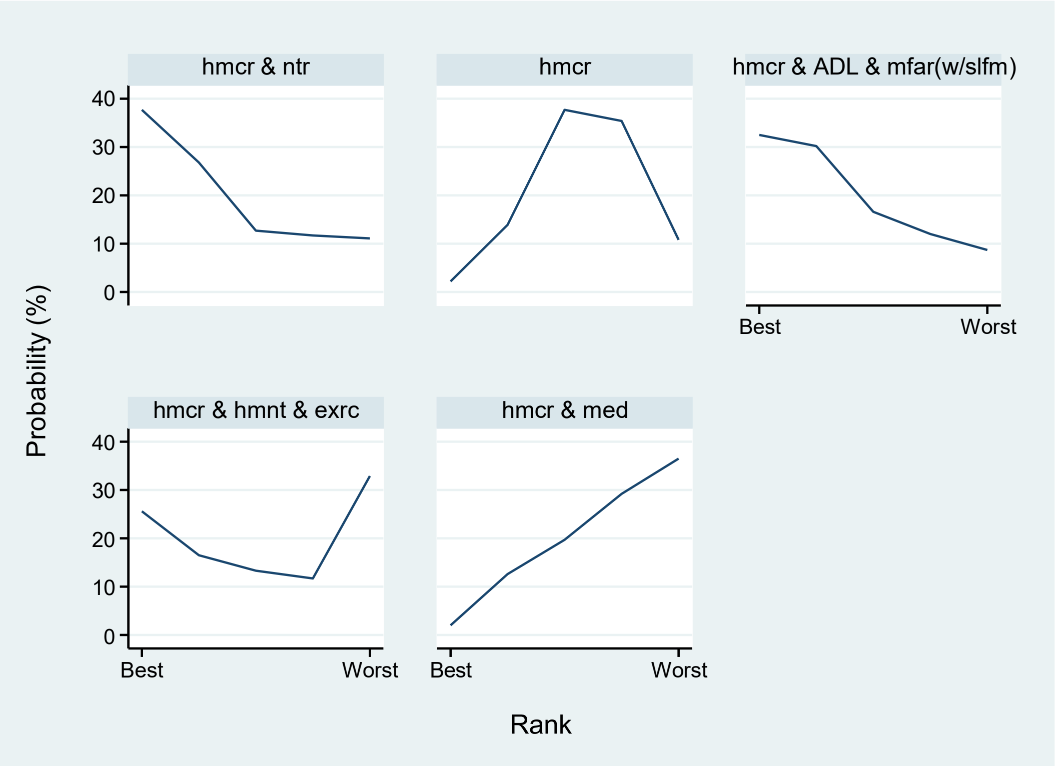

Figure 27 - Rankogram showing comparative effectiveness of interventions for PADL short-term homecare network. Results based on a simulation of 1000 replications.

11.4.5 PADL homecare network, medium-term timeframe

Table 60 - Medium-term PADL homecare network

Study	Frailty	n	Experimental group	Control group	ROB					
					D1	D2	D3	D4	D5	Overall
**Bernabei 1998**[508]	frail	199	hmcr & mfar(w/med)	hmcr	-	-	x	-	-	x
**Fernandez-Barres 2017**[531]	frail	111	hmcr & ntr	hmcr	+	-	x	-	-	x
**Rooijackers 2021**[594]	frail	264	hmcr & ADL & mfar(w/slfm)	hmcr	+/-	-	x	+	x	xx
**Teut 2013**[609]	frail	58	hmcr & hmnt & exrc	hmcr	+/+	-	x	-	-	x

n: number of participants. ROB: risk of bias. D#: Domain #. D1: risk of bias arising from the randomisation process (individual); or, for cluster trials, risk of bias arising from the randomisation process / risk of bias arising from the identification or recruitment of participants into clusters. D2: risk of bias due to deviations from the intended interventions (effect of assignment to the intervention). D3: risk of bias due to missing outcome data. D4: risk of bias in measurement of the outcome. D5: risk of bias in selection of the reported result. +: low risk of bias; -: some concerns; x: high risk of bias / serious concerns; xx: very serious concerns (overall risk of bias only). all: robust, pre-frail and frail.

Table 61 - PADL in the medium term: comparisons with homecare summary of findings table

Population: Older people

Interventions: Community-based complex interventions

Comparator: Formal homecare (hmcr)

Outcome: personal activities of daily living (higher is better)

Timeframe: medium term; follow up at 12 months

Setting: Community

Total studies: 4

Total participants: 632

Comparator rank: Mean 4.4, 95% CI 3 to 5

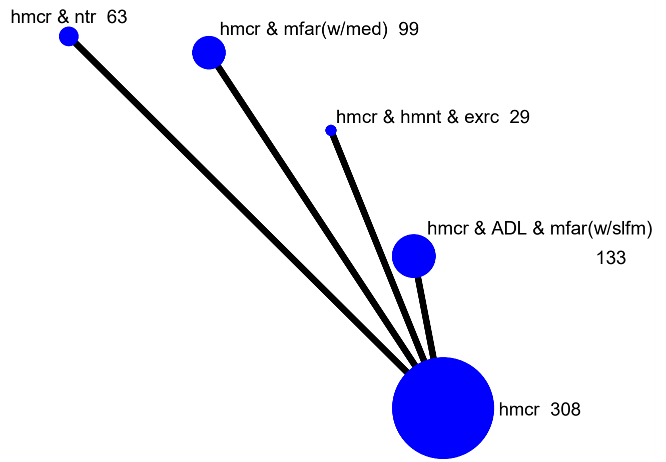

Intervention group	Anticipated absolute effect (95% CI): SMD	Anticipated absolute effect (95% CI): MD (Barthel Index 0 to 100)[a]	Certainty of the evidence (GRADE)	Ranking (95% CI)	Interpretation
Homecare, multifactorial-action and review with medication-review (hmcr & mfar(w/med))	SMD 0.60 higher<br>(0.32 higher to 0.88 higher)<br>Mixed estimate	MD 17.74 higher<br>(9.32 higher to 26.15 higher)	⊕⊕⊖⊖<br>**Low**[b,c]	1.1<br>(1 to 2)	may result in an increase in PADL
Homecare and nutrition (hmcr & ntr)	SMD 0.23 higher<br>(0.15 lower to 0.60 higher)<br>Mixed estimate	MD 6.70 higher<br>(4.45 lower to 17.85 higher)	⊕⊖⊖⊖<br>**Very low**[b,d]	2.7<br>(1 to 5)	the evidence is very uncertain about the effect on PADL
Homecare, ADL, multifactorial-action and review with self-management (hmcr & ADL & mfar(w/slfm))	SMD 0.12 higher<br>(0.13 lower to 0.36 higher)<br>Mixed estimate	MD 3.42 higher<br>(3.73 lower to 10.57 higher)	⊕⊖⊖⊖<br>**Very low**[e,f]	3.4<br>(2 to 5)	the evidence is very uncertain about the effect on PADL
Homecare, alternative-medicine and exercise (hmcr & hmnt & exrc)	SMD 0.10 higher<br>(0.42 lower to 0.61 higher)<br>Mixed estimate	MD 2.83 higher<br>(12.41 lower to 18.08 higher)	⊕⊖⊖⊖<br>**Very low**[b,d]	3.4<br>(1 to 5)	the evidence is very uncertain about the effect on PADL

a: calculated from the estimated SMD using a standard deviation of 29.6, the pooled standard deviation across intervention groups reporting the Barthel Index.

b: serious concerns about risk of bias due to missing outcome data. Downgrade once.

c: serious concerns about imprecision as no closed loop and direct evidence is based on 99 persons in homecare, multifactorial-action and review with medication-review which does not meet optimal information size. Downgrade once.

d: very serious concerns about imprecision as confidence interval includes substantial benefit and harm (SMD +/- 0.05). Downgrade twice.

e: very serious concerns about risk of bias due to missing outcome data and because reported results were not analysed in accordance with the protocol. Downgrade twice.

f: very serious concerns about imprecision as confidence interval includes substantial benefit and harm (SMD +/- 0.05). Already downgraded twice for risk of bias, downgrade once.

Table 62 - Results of PADL: medium-term homecare network

**hmcr & ntr**				0.23 (-0.15,0.60)
-0.37 (-0.84,0.10)	**hmcr & mfar(w/med)**			0.60 (0.31,0.88)
0.13 (-0.51,0.77)	0.50 (-0.08,1.09)	**hmcr & hmnt & exrc**		0.10 (-0.42,0.61)
0.11 (-0.34,0.56)	0.48 (0.11,0.86)	-0.02 (-0.59,0.55)	**hmcr & ADL & mfar(w/slfm)**	0.12 (-0.13,0.36)
0.23 (-0.15,0.60)	0.60 (0.31,0.88)	0.10 (-0.42,0.61)	0.12 (-0.13,0.36)	**hmcr**

Lower left triangle presents the findings (SMD with 95% CI) of the network meta-analysis. Upper right triangle presents the findings (SMD with 95% CI) of pairwise meta-analyses. A SMD>1 favours the upper left intervention; a SMD<1 favours the lower right intervention. Within the table, comparisons between treatments should be read from left to right (i.e. treatment 1 versus treatment 2). The estimate effect measure (SMD and their 95% CI) is in the cell in common between the row- and column-defining treatment.

Table 63 - Intervention rankings for PADL: medium-term homecare network

Treatment	SUCRA	Pr(Best)	Mean Rank	LCI Rank	UCI Rank
hmcr & mfar(w/med)	97	89	1.1	1	2
hmcr & ntr	57.5	5.9	2.7	1	5
hmcr & ADL & mfar(w/slfm)	41.2	0.3	3.4	2	5
hmcr & hmnt & exrc	39	4.8	3.4	1	5
hmcr	15.3	0	4.4	3	5

SUCRA values (0–100) and mean ranks are presented, based on 1000 simulations. Higher SUCRAs and lower mean ranks indicate better performing interventions. Pr(Best) gives the probability of each specific intervention being ranked best intervention, based on 1000 simulations.

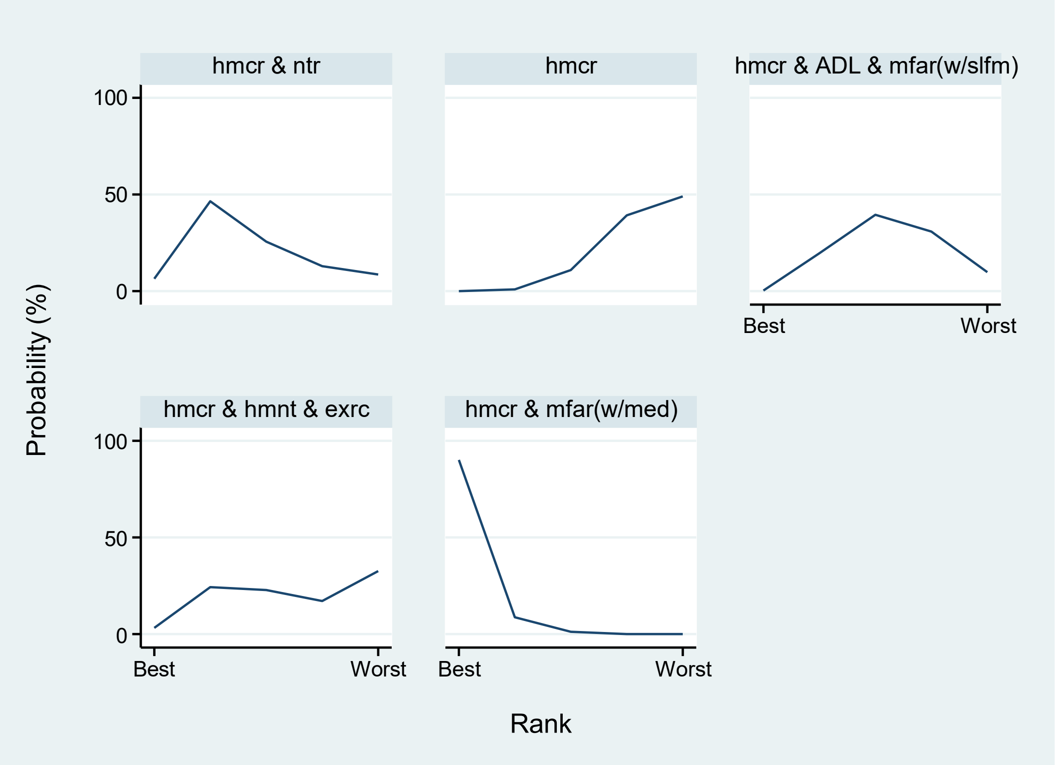

Figure 28 - Rankogram showing comparative effectiveness of interventions for PADL medium-term homecare network. Results based on a simulation of 1000 replications.

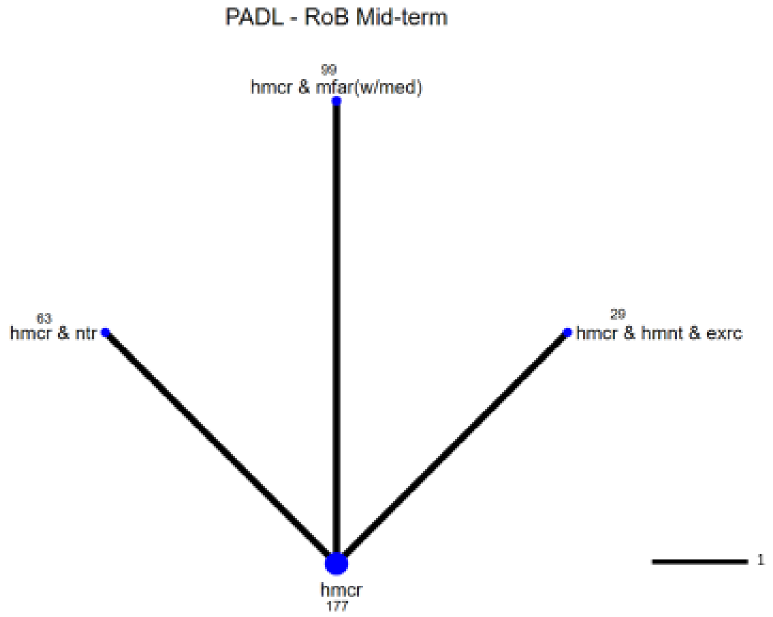

Figure 29 - Network plot for risk of bias analysis for PADL medium-term homecare network

Table 64 - Intervention rankings for risk of bias analysis for PADL: medium-term homecare network

Intervention group	SUCRA	PrBest	Mean Rank	95% CI for true rank
hmcr & mfar(w/med)	96.1	89.4	1.1	1 - 2
hmcr & ntr	53.1	5.6	2.4	1 - 4
hmcr & hmnt & exrc	35.2	5	2.9	1 - 4
hmcr	15.6	0	3.5	2 - 4

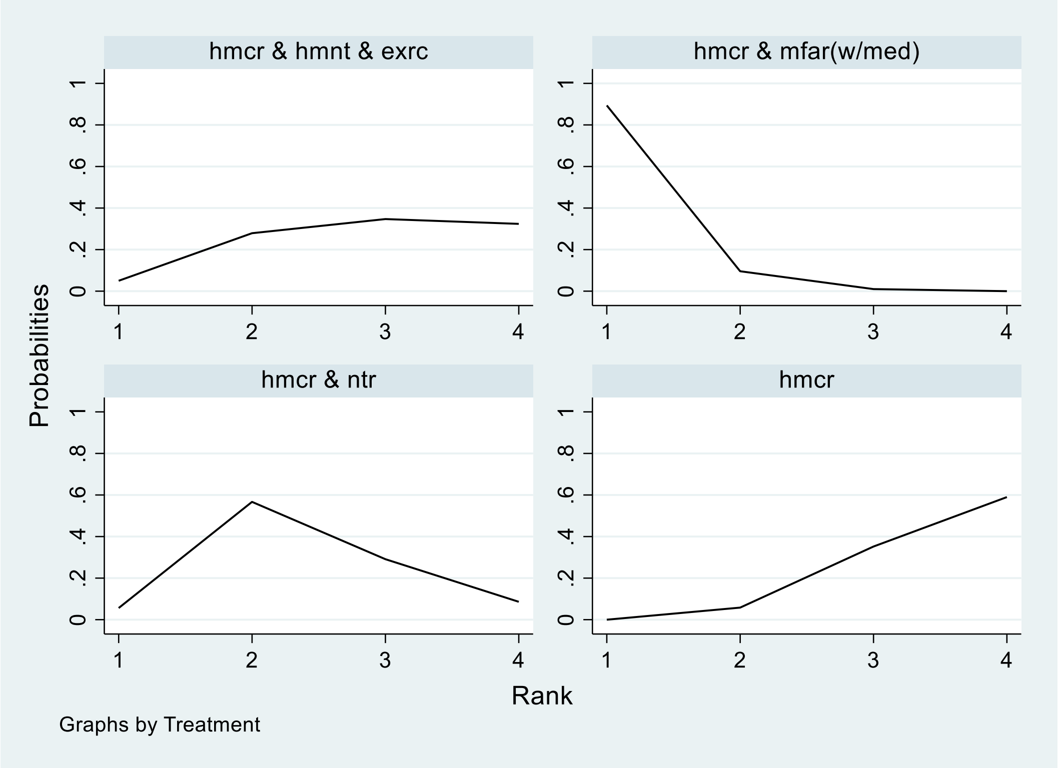

Figure 30 - Rankogram showing comparative effectiveness of interventions for risk of bias sensitivity analysis for PADL medium-term homecare network. Results based on a simulation of 1000 replications.

Table 65 - Results of risk of bias PADL: medium-term homecare network

**hmcr**	-0.23 (-0.60,0.15)	**-0.60 (-0.88,-0.32)**	-0.10 (-0.61,0.42)
-0.23 (-0.60,0.15)	**hmcr & ntr**		
**-0.60 (-0.88,-0.32)**	-0.37 (-0.84,0.10)	**hmcr & mfar(w/med)**	
-0.10 (-0.61,0.42)	0.13 (-0.51,0.77)	0.50 (-0.08,1.09)	**hmcr & hmnt & exrc**

11.4.6 PADL homecare network, long-term timeframe

No results as there were too few comparisons to conduct network meta-analysis.

11.5 Hospitalisation

11.5.1 Hospitalisation available care network (medium-term timeframe only)

Table 66 - Medium-term hospitalisation available-care network

					ROB					
**Study**	**Frailty**	**n**	**Experimental group**	**Control group**	**D1**	**D2**	**D3**	**D4**	**D5**	**Overall**
**Bouman 2008**[513]	pre-frail and frail	330	mfar(w/med)	ac	+	-	+	+	-	-
**Cameron 2013**[515]	frail	241	exrc & mfar(w/med+slfm)	ac	+	-	+	+	+	-
**Harari 2008**[544]	all	1969	mfar(w/med)	ac	+	x	x	+	+	xx
**Henderson 2005**[548]	robust	124	mfar	ac	+/x	+	x	+	-	xx
**Hendriksen 1984**[549]	all	543	mfar	ac	-	-	x	+	-	x
**Kono 2016**[558]	pre-frail	305	mfar(w/med)	mfar	+	-	x	+	-	x
**Leveille 1998**[564]	unclassifiable	200	educ & exrc & mfar(w/med+slfm)	ac	+	-	+	+	-	-
**Newcomer 2004**[583]	unclassifiable	3055	educ & mfar(w/med)	ac	-	-	-	+	-	-
**Ng 2015**[584]	pre-frail and frail	92	cgn & ntr & exrc	ac	+	-	x	+	-	x
**Phelan 2007**[589]	all	299	mfar(w/med+slfm)	ac	-/x	-	x	+	-	xx
**Rubenstein 2007**[595]	frail	694	mfar(w/med)	ac	-	-	x	+	-	x
**Takahashi 2012**[608]	frail	205	mntr-mfa-	ac	-	-	x	+	+	x
**van Hout 2010**[618]	frail	651	mfar(w/med)	ac	+	-	-	+	-	-
**van Lieshout 2018**[620]	pre-frail and frail	281	ADL & med & ntr & sst	ac	-	-	x	+	-	xx
**van Rossum 1993**[621]	all	580	mfar	ac	-	-	-	+	-	-

n: number of participants. ROB: risk of bias. D#: Domain #. D1: risk of bias arising from the randomisation process (individual); or, for cluster trials, risk of bias arising from the randomisation process / risk of bias arising from the identification or recruitment of participants into clusters. D2: risk of bias due to deviations from the intended interventions (effect of assignment to the intervention). D3: risk of bias due to missing outcome data. D4: risk of bias in measurement of the outcome. D5: risk of bias in selection of the reported result. +: low risk of bias; -: some concerns; x: high risk of bias / serious concerns; xx: very serious concerns (overall risk of bias only). all: robust, pre-frail and frail.

Table 67 - Hospitalisation in the medium term: comparisons with available care summary of findings table.

Population: Older people

Interventions: Community-based complex interventions

Comparator: Available care (ac)

Outcome: hospitalisation

Timeframe: medium term; range of follow up 12 to 18 months

Setting: Community

Total studies: 15

Total participants: 9569

Comparator rank: Mean 4.4, 95% CI 5 to 8

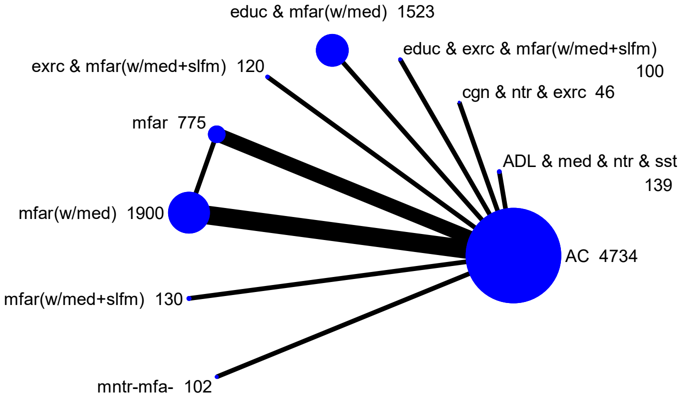

	Relative effect (95% CI)		Anticipated absolute effect (95% CI)						
			High-risk population (520 per 1000 with ac)		Low-risk population (118 per 1000 with ac)				
**Intervention**	**Network estimate**	**Calculated risk ratio[a]**	**With intervention**	**Difference**	**With intervention**	**Difference**	**Certainty of the evidence (GRADE)**	**Ranking (95% CI)**	**Interpretation**
Education, exercise, multifactorial-action and review with medication-review and self-management strategies (educ & exrc & mfar(w/med+slfm))	OR 0.53 (0.25 to 1.12) Mixed estimate	RR 0.59 (0.30 to 1.09)	365 per 1000 (213 to 549)	155 fewer per 1000 (307 fewer to 29 more)	66 per 1000 (32 to 131)	52 fewer per 1000 (86 fewer to 13 more)	⊕⊕⊖⊖ Low[b]	1.4 (5 to 10)	may result in a reduction in chance of being hospitalised
Education, multifactorial-action and review with medication-review (educ & mfar(w/med))	OR 0.92 (0.78 to 1.09) Mixed estimate	RR 0.94 (0.82 to 1.07)	499 per 1000 (457 to 542)	21 fewer per 1000 (63 fewer to 22 more)	110 per 1000 (94 to 127)	8 fewer per 1000 (24 fewer to 9 more)	⊕⊕⊖⊖ Low[c]	3.3 (5 to 9)	may result in a slight reduction in chance of being hospitalised
Exercise, multifactorial-action and review with medication-review and self-management strategies (exrc & mfar(w/med+slfm))	OR 1.34 (0.80 to 2.24) Mixed estimate	RR 1.24 (0.84 to 1.75)	592 per 1000 (465 to 708)	72 more per 1000 (55 fewer to 188 more)	152 per 1000 (97 to 231)	34 more per 1000 (21 fewer to 113 more)	⊕⊕⊖⊖ Low[d]	7.0 (1 to 8)	may result in an increase in chance of being hospitalised
Multifactorial-action and review (mfar)	OR 0.81 (0.62 to 1.06) Mixed estimate	RR 0.85 (0.67 to 1.05)	467 per 1000 (400 to 535)	53 fewer per 1000 (120 fewer to 15 more)	98 per 1000 (76 to 124)	20 fewer per 1000 (42 fewer to 6 more)	⊕⊖⊖⊖ Very low[c,e]	2.4 (6 to 10)	the evidence is very uncertain about the effect on chance of being hospitalised
Multifactorial-action and review with medication-review (mfar(w/med))	OR 1.10 (0.95 to 1.28) Mixed estimate	RR 1.08 (0.96 to 1.20)	544 per 1000 (507 to 581)	24 more per 1000 (13 fewer to 61 more)	129 per 1000 (113 to 146)	11 more per 1000 (5 fewer to 28 more)	⊕⊖⊖⊖ Very low[c,e]	5.7 (3 to 8)	the evidence is very uncertain about the effect on chance of being hospitalised
Multifactorial-action and review with medication-review and self-management strategies (mfar(w/med+slfm))	OR 1.37 (0.76 to 2.50) Mixed estimate	RR 1.27 (0.80 to 1.86)	598 per 1000 (450 to 731)	78 more per 1000 (70 fewer to 211 more)	155 per 1000 (92 to 251)	37 more per 1000 (26 fewer to 133 more)	⊕⊖⊖⊖ Very low[f,g]	6.7 (1 to 9)	the evidence is very uncertain about the effect on chance of being hospitalised

Monitoring (mntr-mfa-)	OR 1.39 (0.80 to 2.42) Mixed estimate	RR 1.28 (0.84 to 1.83)	602 per 1000 (466 to 724)	82 more per 1000 (54 fewer to 204 more)	157 per 1000 (97 to 244)	39 more per 1000 (21 fewer to 126 more)	⊕⊖⊖⊖ Very low[h,i]	7.0 (1 to 9)	the evidence is very uncertain about the effect on chance of being hospitalised
ADL, medication-review, nutrition and social-skills (ADL & med & ntr & sst)	OR 1.70 (0.93 to 3.09) Mixed estimate	RR 1.46 (0.95 to 2.09)	648 per 1000 (503 to 770)	128 more per 1000 (17 fewer to 250 more)	185 per 1000 (111 to 293)	67 more per 1000 (7 fewer to 175 more)	⊕⊖⊖⊖ Very low[j,k]	8.1 (1 to 7)	the evidence is very uncertain about the effect on chance of being hospitalised
Cognitive training, nutrition and exercise (cgn & ntr & exrc)	OR 3.30 (0.63 to 17.30) Mixed estimate	RR 2.16 (0.69 to 3.67)	781 per 1000 (405 to 949)	261 more per 1000 (115 fewer to 429 more)	306 per 1000 (78 to 698)	188 more per 1000 (40 fewer to 580 more)	⊕⊖⊖⊖ Very low[h,l]	9.0 (1 to 9)	the evidence is very uncertain about the effect on chance of being hospitalised

a: Calculated from OR and an assumed comparator risk of 0.228, the median available care risk among these studies.

b: very serious concerns about imprecision as confidence interval includes substantial benefit and harm, and as no closed loop and direct comparison is based on 35 events from 200 persons which does not meet optimal information size. Downgrade twice.

c: very serious concerns about imprecision as confidence interval includes substantial benefit and harm. Downgrade twice.

d: very serious concerns about imprecision as confidence interval includes substantial harm and harm, and as no closed loop and direct comparison is based on 140 events from 241 persons which does not meet optimal information size. Downgrade twice.

e: serious concerns about risk of bias mainly due to missing outcome data among the studies. Downgrade once.

f: very serious concerns about risk of bias due to the recruitment process of participants and missing data. Downgrade twice.

g: very serious concerns about imprecision as confidence interval includes substantial benefit and harm, and as no closed loop and direct comparison is based on 52 events from 299 persons which does not meet optimal information size. Already downgraded twice for risk of bias, downgrade once.

h: serious concerns about risk of bias due to missing outcome data. Downgrade once.

i: very serious concerns about imprecision as confidence interval includes substantial benefit and harm, and as no closed loop and direct comparison is based on 98 events from 205 persons which does not meet optimal information size. Downgrade twice.

j: very serious concerns about risk of bias due to substantial number of missing outcome data. Downgrade twice.

k: very serious concerns about imprecision as confidence interval includes substantial benefit and harm, and as no closed loop and direct comparison is based on 55 events from 281 persons which does not meet optimal information size. Already downgraded twice for risk of bias, downgrade once.

l: very serious concerns about imprecision as confidence interval includes substantial benefit and harm, and as no closed loop and direct comparison is based on 8 events from 95 persons which does not meet optimal information size. Downgrade twice.

Table 68 - Results of hospitalisation medium-term analysis

**mntr-mfa-**									1.39 (0.80, 2.42)
1.01 (0.45, 2.29)	**mfar(w/med+slfm)**								1.38 (0.76, 2.50)
1.26 (0.71, 2.23)	1.25 (0.67, 2.31)	**mfar(w/med)**	1.07 (0.59, 1.97)						1.12 (0.96, 1.30)
1.72 (0.93, 3.19)	1.70 (0.88, 3.29)	1.36 (1.01, 1.83)	**mfar**						0.76 (0.56, 1.03)
1.04 (0.49, 2.21)	1.03 (0.47, 2.26)	0.82 (0.48, 1.40)	0.60 (0.34, 1.08)	**exrc & mfar(w/med+slfm)**					1.34 (0.80, 2.24)
1.51 (0.85, 2.69)	1.49 (0.80, 2.78)	1.20 (0.96, 1.50)	0.88 (0.64, 1.21)	1.46 (0.85, 2.50)	**educ & mfar(w/med)**				0.92 (0.78, 1.09)
2.63 (1.04, 6.67)	2.60 (0.99, 6.78)	2.08 (0.97, 4.48)	1.53 (0.69, 3.39)	2.53 (1.02, 6.28)	1.74 (0.81, 3.75)	**educ & exrc & mfar(w/med+slfm)**			0.53 (0.25, 1.12)
0.42 (0.07, 2.42)	0.42 (0.07, 2.43)	0.33 (0.06, 1.76)	0.25 (0.05, 1.31)	0.41 (0.07, 2.30)	0.28 (0.05, 1.48)	0.16 (0.03, 0.99)	**cgn & ntr & exrc**		3.30 (0.63, 17.30)
0.82 (0.36, 1.85)	0.81 (0.35, 1.89)	0.65 (0.35, 1.20)	0.48 (0.25, 0.92)	0.79 (0.36, 1.74)	0.54 (0.29, 1.01)	0.31 (0.12, 0.82)	1.94 (0.33, 11.31)	**ADL & med & ntr & sst**	1.70 (0.93, 3.09)
1.39 (0.80, 2.42)	1.38 (0.76, 2.50)	1.10 (0.95, 1.28)	0.81 (0.62, 1.06)	1.34 (0.80, 2.24)	0.92 (0.78, 1.09)	0.53 (0.25, 1.12)	3.30 (0.63, 17.30)	1.70 (0.93, 3.09)	**AC**

Lower left triangle presents the findings (OR with 95% CI) of the network meta-analysis. Upper right triangle presents the findings (OR with 95% CI) of pairwise meta-analyses. A OR<1 favours the upper left intervention; a OR>1 favours the lower right intervention. Within the table, comparisons between treatments should be read from left to right (i.e. treatment 1 versus treatment 2). The estimate effect measure (OR and their 95% CI) is in the cell in common between the row- and column-defining treatment.

Table 69 - Intervention rankings for hospitalisation medium-term analysis

Treatment	SUCRA	Pr(Best)	Mean Rank	LCI rank	UCI rank
educ & exrc & mfar(w/med+slfm)	95.5	83.5	1.4	1	6
mfar	84.6	11.2	2.4	1	5
educ & mfar(w/med)	74.2	1.3	3.3	2	6
ac	62.6	0	4.4	3	6
mfar(w/med)	48.1	0	5.7	3	8
mfar(w/med+slfm)	36.3	0.9	6.7	2	10
mntr-mfa-	33.1	0.9	7	3	10
exrc & mfar(w/med+slfm)	33.6	0.5	7	2	10
ADL & med & ntr & sst	20.7	0.2	8.1	4	10
cgn & ntr & exrc	11.3	1.5	9	2	10

SUCRA values (0–100) and mean ranks are presented, based on 1000 simulations. Higher SUCRAs and lower mean ranks indicate better performing interventions. Pr(Best) gives the probability of each specific intervention being ranked best intervention, based on 1000 simulations.

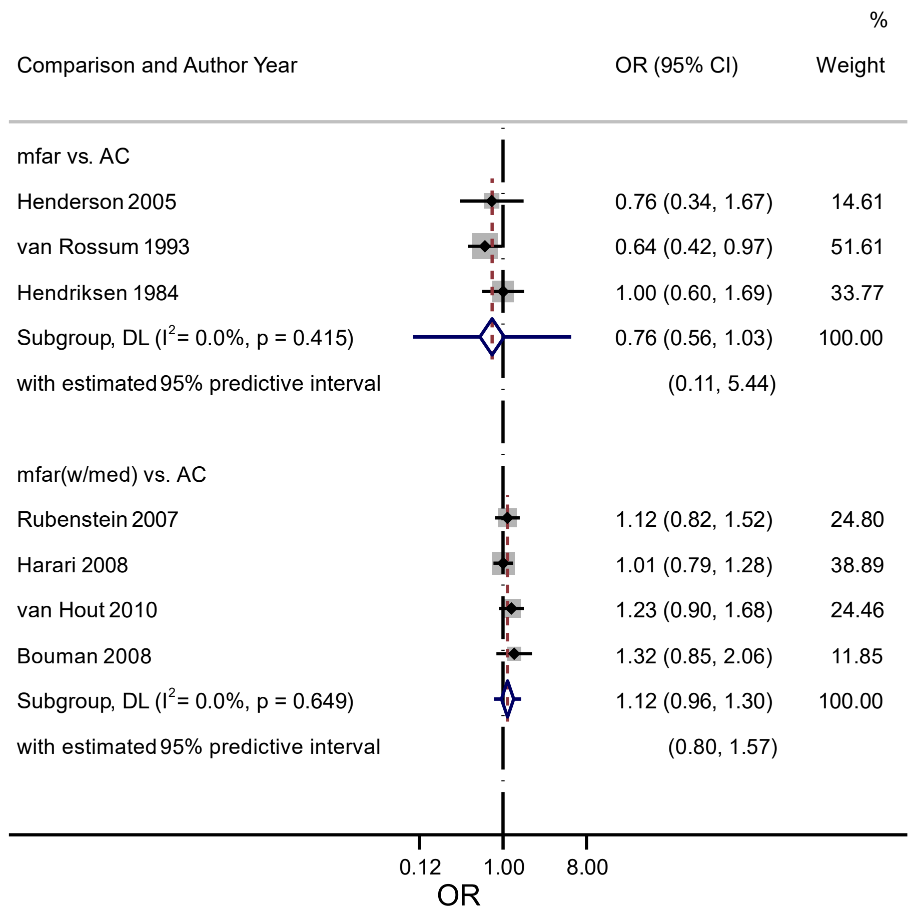

Figure 31 - Pairwise meta-analysis for hospitalisation: medium-term available care network (pooling comparisons with greater than one study reporting results)

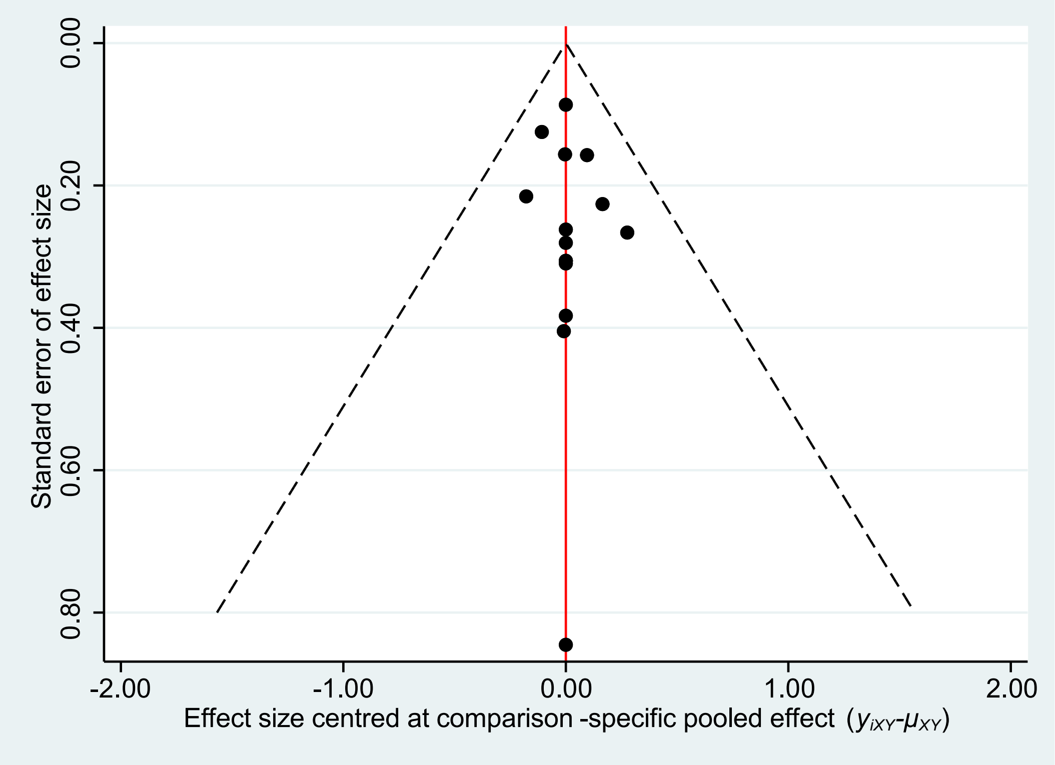

Figure 32 - Comparison-adjusted funnel plot for hospitalisation: medium-term available care network

11.5.2 Hospitalisation homecare network (medium-term timeframe only)

No results as there were too few comparisons to conduct network meta-analysis.

11.6 Care-home placement

11.6.1 Care-home placement available care network, short-term timeframe

Table 70 - Short-term care-home placement available-care network

Study	Frailty	n	Experimental group	Control group	ROB					
					D1	D2	D3	D4	D5	Overall
**Imhof 2012[553]**	all	436	mfar	ac	-	-	x	+	-	x
**Kukkonen-Harjula 2017[561]**	pre-frail and frail	284	ADL & ntr & exrc	ac	+	-	x	+	-	x
**Liddle 1996[566]**	unclassifiable	102	aids & mfar	ac	-	x	x	+	-	xx
**Metzelthin 2013[576]**	frail	335	educ & mfar(w/med+slfm)	ac	-/-	-	x	+	-	x
**Suijker 2016[605]**	frail	1983	mfar(w/med)	ac	+/-	-	x	+	-	x
**Szanton 2011[606]**	pre-frail and frail	38	ADL&aids&educ&exrc& mfar(w/med+slfm)	ac	-	-	+	+	-	-
**Wong 2019[630]**	all	494	mfar(w/slfm)	ac	x	-	x	+	-	xx

n: number of participants. ROB: risk of bias. D#: Domain #. D1: risk of bias arising from the randomisation process (individual); or, for cluster trials, risk of bias arising from the randomisation process / risk of bias arising from the identification or recruitment of participants into clusters. D2: risk of bias due to deviations from the intended interventions (effect of assignment to the intervention). D3: risk of bias due to missing outcome data. D4: risk of bias in measurement of the outcome. D5: risk of bias in selection of the reported result. +: low risk of bias; -: some concerns; x: high risk of bias / serious concerns; xx: very serious concerns (overall risk of bias only). all: robust, pre-frail and frail.

Table 71 - Care-home placement in the short term: comparisons with available care summary of findings table

Population: Older people

Interventions: Community-based complex interventions

Comparator: Available care (ac)

Outcome: care-home placement

Timeframe: short term; range of follow up 24 weeks to 6 months

Setting: Community

Total studies: 7

Total participants: 3672

Comparator rank: Mean 4.6, 95% CI 3 to 7

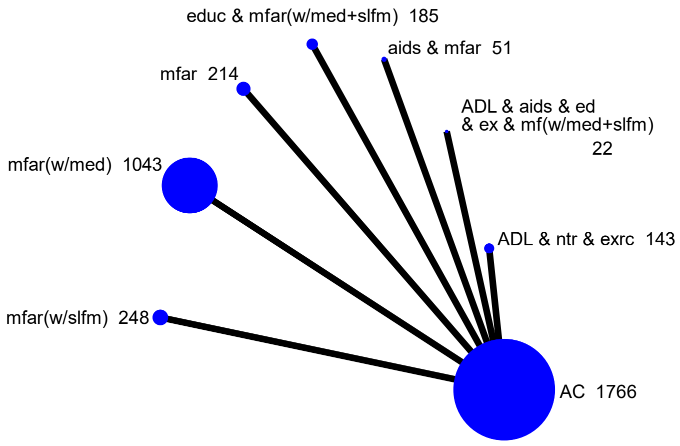

	Relative effect (95% CI)		Anticipated absolute effect (95% CI)						
			High-risk population (28 per 1000 with ac)		Low-risk population (2 per 1000 with ac)				
Intervention group	Network estimate	Calculated risk ratio[a]	With intervention	Difference	With intervention	Difference	Certainty of the evidence (GRADE)	Ranking (95% CI)	Interpretation
Multifactorial-action and review with medication-review (mfar(w/med))	OR 0.77 (0.17 to 3.50) Mixed estimate	RR 0.78 (0.17 to 3.44)	22 per 1000 (5 to 92)	6 fewer per 1000 (23 fewer to 64 more)	2 per 1000 (0 to 7)	0 per 1000 (2 fewer to 5 more)	⊕⊖⊖⊖ Very low[b,c]	2.3 (1 to 7)	the evidence is very uncertain about the effect on care-home placement
Aids, multifactorial-action and review (aids & mfar)	OR 4.02 (0.18 to 89.76) Mixed estimate	RR 3.94 (0.18 to 55.36)	104 per 1000 (5 to 721)	76 more per 1000 (23 fewer to 693 more)	8 per 1000 (0 to 152)	6 more per 1000 (2 fewer to 150 more)	⊕⊖⊖⊖ Very low[d,e]	3.1 (1 to 8)	the evidence is very uncertain about the effect on care-home placement
Multifactorial-action and review (mfar)	OR 2.46 (0.25 to 23.86) Mixed estimate	RR 2.43 (0.25 to 20.57)	66 per 1000 (7 to 407)	38 more per 1000 (21 fewer to 379 more)	5 per 1000 (1 to 46)	3 more per 1000 (1 fewer to 44 more)	⊕⊖⊖⊖ Very low[b,c]	4.0 (1 to 7)	the evidence is very uncertain about the effect on care-home placement
ADL, nutrition and exercise (ADL & ntr & exrc)	OR 0.99 (0.34 to 2.87) Mixed estimate	RR 0.99 (0.34 to 2.83)	28 per 1000 (10 to 76)	0 per 1000 (18 fewer to 48 more)	2 per 1000 (1 to 6)	0 per 1000 (1 fewer to 4 more)	⊕⊖⊖⊖ Very low[b,c]	4.6 (1 to 8)	the evidence is very uncertain about the effect on care-home placement
Multifactorial-action and review with self-management strategies (mfar(w/slfm))	OR 0.18 (0.01 to 3.75) Mixed estimate	RR 0.18 (0.01 to 3.68)	5 per 1000 (0 to 98)	23 fewer per 1000 (28 fewer to 70 more)	0 per 1000 (0 to 7)	2 fewer per 1000 (2 fewer to 5 more)	⊕⊖⊖⊖ Very low[e,f]	4.6 (2 to 7)	the evidence is very uncertain about the effect on care-home placement
Education, multifactorial-action and review with medication-review and self-management strategies (educ & mfar(w/med+slfm))	OR 0.33 (0.01 to 8.21) Mixed estimate	RR 0.33 (0.01 to 7.82)	9 per 1000 (0 to 191)	19 fewer per 1000 (28 fewer to 163 more)	1 per 1000 (0 to 16)	1 fewer per 1000 (2 fewer to 14 more)	⊕⊖⊖⊖ Very low[b,c]	6.2 (2 to 8)	the evidence is very uncertain about the effect on care-home placement
ADL, aids, education, exercise, multifactorial-action and review with medication-review and self-management strategies (ADL&aids&educ&exrc& mfar(w/med+slfm))	OR 0.99 (0.02 to 50.04) Mixed estimate	RR 0.99 (0.02 to 37.25)	28 per 1000 (1 to 590)	0 per 1000 (27 fewer to 562 more)	2 per 1000 (0 to 91)	0 per 1000 (2 fewer to 89 more)	⊕⊖⊖⊖ Very low[g]	6.6 (2 to 9)	the evidence is very uncertain about the effect on care-home placement

a: Calculated from OR and an assumed comparator risk of 0.007, the median available care risk among these studies.

b: serious concerns about risk of bias due to missing outcome data. Downgrade once.

c: very serious concerns about imprecision as confidence interval includes substantial benefit and substantial harm. Downgrade twice.

d: very serious concerns about risk of bias due to deviations from the intended interventions and missing data. Downgrade twice.

e: very serious concerns about imprecision as confidence interval includes substantial benefit and substantial harm. Already downgraded twice for risk of bias, downgrade once.

f: very serious concerns about risk of bias due to randomisation process and missing outcome data. Downgrade twice.

g: extremely serious concerns about imprecision as confidence interval is extremely wide. Downgrade three levels.

Table 72 - Results of care-home placement: short-term available care network

**mfar(w/slfm)**							0.99 (0.34,2.87)
5.51 (0.22,137.74)	**mfar(w/med)**						0.18 (0.01,3.75)
1.28 (0.20,8.10)	0.23 (0.01,6.90)	**mfar**					0.77 (0.17,3.50)
0.40 (0.03,4.97)	0.07 (0.00,3.25)	0.32 (0.02,4.83)	**educ & mfar(w/med+slfm)**				2.46 (0.25,23.86)
3.03 (0.10,90.43)	0.55 (0.01,46.17)	2.37 (0.07,83.33)	7.51 (0.15,388.32)	**aids & mfar**			0.33 (0.01,8.21)
0.25 (0.01,6.56)	0.04 (0.00,3.44)	0.19 (0.01,6.08)	0.61 (0.01,28.63)	0.08 (0.00,7.14)	**ADL&aids&ed&ex&mf(w/med+slfm)**		4.02 (0.18,89.76)
1.01 (0.02,58.78)	0.18 (0.00,26.13)	0.79 (0.01,52.76)	2.49 (0.03,232.77)	0.33 (0.00,53.33)	4.08 (0.03,609.32)	**ADL & ntr & exrc**	0.99 (0.02,50.04)
0.99 (0.34,2.87)	0.18 (0.01,3.75)	0.77 (0.17,3.50)	2.46 (0.25,23.86)	0.33 (0.01,8.21)	4.02 (0.18,89.76)	0.99 (0.02,50.04)	**ac**

Lower left triangle presents the findings (OR with 95% CI) of the network meta-analysis. Upper right triangle presents the findings (OR with 95% CI) of pairwise meta-analyses. A OR<1 favours the upper left intervention; a OR>1 favours the lower right intervention. Within the table, comparisons between treatments should be read from left to right (i.e. treatment 1 versus treatment 2). The estimate effect measure (OR and their 95% CI) is in the cell in common between the row- and column-defining treatment.

Table 73 - Intervention rankings for care-home placement: short-term available care network

Treatment	SUCRA	Pr(Best)	Mean Rank	LCI Rank	UCI Rank
mfar(w/med)	81.7	47.4	2.3	1	7
aids & mfar	70.6	30.9	3.1	1	8
mfar	56.7	4.7	4.0	1	7
mfar(w/slfm)	47.9	0.8	4.6	2	7
ac	48.6	0.1	4.6	3	7
ADL & ntr & exrc	48.5	13.5	4.6	1	8
educ & mfar(w/med+slfm)	25.8	0.9	6.2	2	8
ADL&aids&ed&mfar(w/med+slfm)	20.2	1.7	6.6	2	9

SUCRA values (0–100) and mean ranks are presented, based on 1000 simulations. Higher SUCRAs and lower mean ranks indicate better performing interventions. Pr(Best) gives the probability of each specific intervention being ranked best intervention, based on 1000 simulations.

11.6.2 Care-home placement available care network, medium-term timeframe

Table 74 - Medium-term care-home placement available-care network

Study	Frailty	n	Experimental group	Control group	ROB					
					D1	D2	D3	D4	D5	Overall
**Blom 2016**[510]	all	1000	mfa-(w/med+slfm)	ac	x/+	-	x	+	-	xx
**Fabacher 1994**[528]	all	221	mfar(w/med)	ac	-	-	x	+	-	x
**Hall 1992**[543]	frail	155	hmcr & mfar(w/slfm)	hmcr & mfar	-	-	x	+	-	x
**Harari 2008**[544]	all	2377	mfar(w/med)	ac	+	x	x	+	+	xx
**Hay 1998**[546]	unclassifiable	470	mfa-	ac	-	-	x	+	-	x
**Hebert 2001**[547]	pre-frail and frail	464	mfar(w/med)	ac	-	-	x	+	-	x
**Henderson 2005**[548]	robust	130	mfar	ac	+/x	+	x	+	-	xx
**Kerse 2014**[556]	pre-frail and frail	3565	rsk-mfa-	ac	+/+	-	x	+	-	x
**Kono 2004**[559]	pre-frail and frail	107	mfar	ac	-	-	x	+	-	x
**Kono 2016**[558]	pre-frail	305	mfar(w/med)	mfar	+	-	x	+	-	x
**Kukkonen-Harjula 2017**[561]	pre-frail and frail	272	ADL & ntr & exrc	ac	+	-	x	+	-	x
**Metzelthin 2013**[576]	frail	313	educ & mfar(w/med+slfm)	ac	-/-	-	x	+	-	x
**Monteserin Nadal 2008**[578]	all	474	educ & rsk-mfa-	ac	-	-	x	+	-	x
**Newbury 2001**[582]	unclassifiable	94	mfa-(w/med)	ac	-	-	x	+	-	x
**Newcomer 2004**[583]	unclassifiable	2845	educ & mfar(w/med)	ac	-	-	x	+	-	x
**Ploeg 2010**[590]	pre-frail and frail	645	educ & mfar(w/med)	ac	+	-	x	+	-	x
**Romera-Liebana 2018**[593]	pre-frail and frail	324	cgn & med & ntr & exrc	ac	+	-	x	+	-	x
**Shapiro 2002**[598]	frail	67	hmcr & mfar	ac	-	-	x	+	-	xx
**Suijker 2016**[605]	frail	1784	mfar(w/med)	ac	+/-	-	x	+	-	x
**van Hout 2010**[618]	frail	443	mfar(w/med)	ac	+	-	x	+	-	x

n: number of participants. ROB: risk of bias. D#: Domain #. D1: risk of bias arising from the randomisation process (individual); or, for cluster trials, risk of bias arising from the randomisation process / risk of bias arising from the identification or recruitment of participants into clusters. D2: risk of bias due to deviations from the intended interventions (effect of assignment to the intervention). D3: risk of bias due to missing outcome data. D4: risk of bias in measurement of the outcome. D5: risk of bias in selection of the reported result. +: low risk of bias; -: some concerns; x: high risk of bias / serious concerns; xx: very serious concerns (overall risk of bias only). all: robust, pre-frail and frail.

Table 75 Care-home placement in the medium term: comparisons with available care summary of findings table

Population: Older people

Interventions: Community-based complex interventions

Comparator: Available care (ac)

Outcome: care-home placement

Timeframe: medium term; range of follow up 12 to 18 months

Setting: Community

Total studies: 20

Total participants: 16,055

Comparator rank: Mean 9.2, 95% CI 6 to 12

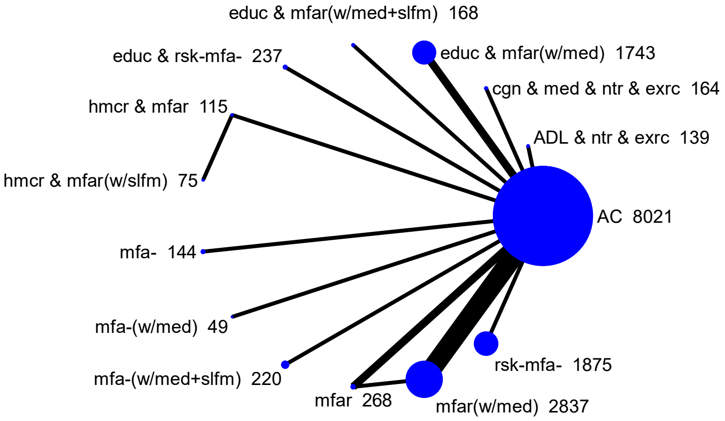

Intervention group	Relative effect (95% CI): Network estimate	Relative effect (95% CI): Calculated risk ratio[a]	High-risk population (50 per 1000 with ac): With intervention	High-risk population (50 per 1000 with ac): Difference	Low-risk population (1 per 1000 with ac)[b]: With intervention	Low-risk population (1 per 1000 with ac)[b]: Difference	Certainty of the evidence (GRADE)	Ranking (95% CI)	Interpretation
Homecare, multifactorial-action and review with self-management strategies (hmcr & mfar(w/slfm))	OR 0.07 (0.01 to 0.53) Indirect estimate	RR 0.07 (0.01 to 0.53)	4 per 1000 (0 to 27)	46 fewer per 1000 (50 fewer to 23 fewer)	0 per 1000 (0 to 1)	1 fewer per 1000 (1 fewer to 0)	⊕⊖⊖⊖ Very low[c,d]	1.6 (1 to 5)	the evidence is very uncertain about the effect on care-home placement
Homecare, multifactorial-action and review (hmcr & mfar)	OR 0.18 (0.04 to 0.78) Mixed estimate	RR 0.18 (0.04 to 0.78)	9 per 1000 (2 to 39)	41 fewer per 1000 (48 fewer to 11 fewer)	0 per 1000 (0 to 1)	1 fewer per 1000 (1 fewer to 0)	⊕⊖⊖⊖ Very low[e,f]	2.9 (1 to 7)	the evidence is very uncertain about the effect on care-home placement
Multifactorial-action (mfa-)	OR 0.32 (0.02 to 6.48) Mixed estimate	RR 0.32 (0.02 to 5.81)	17 per 1000 (1 to 254)	33 fewer per 1000 (49 fewer to 204 more)	0 per 1000 (0 to 6)	1 fewer per 1000 (1 fewer to 5 more)	⊕⊖⊖⊖ Very low[g,h]	5.3 (1 to 14)	the evidence is very uncertain about the effect on care-home placement
Multifactorial-action and review (mfar)	OR 0.53 (0.20 to 1.39) Mixed estimate	RR 0.53 (0.20 to 1.38)	27 per 1000 (10 to 68)	23 fewer per 1000 (40 fewer to 18 more)	1 per 1000 (0 to 1)	0 per 1000 (1 fewer to 0)	⊕⊖⊖⊖ Very low[g,h]	5.7 (2 to 12)	the evidence is very uncertain about the effect on care-home placement
Education and risk-screening (educ & rsk-mfa-)	OR 0.59 (0.13 to 2.72) Mixed estimate	RR 0.60 (0.13 to 2.63)	30 per 1000 (7 to 125)	20 fewer per 1000 (43 fewer to 75 more)	1 per 1000 (0 to 3)	0 per 1000 (1 fewer to 2 more)	⊕⊖⊖⊖ Very low[g,h]	6.6 (2 to 14)	the evidence is very uncertain about the effect on care-home placement
Cognitive training, medication-review, nutrition and exercise (cgn & med & ntr & exrc)	OR 0.65 (0.10 to 4.17) Mixed estimate	RR 0.65 (0.10 to 3.91)	33 per 1000 (5 to 180)	17 fewer per 1000 (45 fewer to 130 more)	1 per 1000 (0 to 4)	0 per 1000 (1 fewer to 3 more)	⊕⊖⊖⊖ Very low[g,h]	7.2 (1 to 14)	the evidence is very uncertain about the effect on care-home placement
Multifactorial-action and review with medication-review (mfar(w/med))	OR 0.81 (0.42 to 1.57) Mixed estimate	RR 0.81 (0.42 to 1.55)	41 per 1000 (22 to 76)	9 fewer per 1000 (28 fewer to 26 more)	1 per 1000 (0 to 2)	0 per 1000 (1 fewer to 1 more)	⊕⊖⊖⊖ Very low[g,h]	7.7 (4 to 12)	the evidence is very uncertain about the effect on care-home placement

Multifactorial-action with medication-review (mfa-(w/med))	OR 0.91 (0.12 to 7.18) Mixed estimate	RR 0.92 (0.12 to 6.35)	46 per 1000 (6 to 274)	4 fewer per 1000 (44 fewer to 224 more)	1 per 1000 (0 to 7)	0 per 1000 (1 fewer to 6 more)	⊕⊖⊖⊖ Very low[g,h]	8.3 (2 to 14)	the evidence is very uncertain about the effect on care-home placement
ADL, nutrition and exercise (ADL & ntr & exrc)	OR 0.96 (0.13 to 7.29) Mixed estimate	RR 0.96 (0.13 to 6.44)	48 per 1000 (7 to 277)	2 fewer per 1000 (43 fewer to 227 more)	1 per 1000 (0 to 7)	0 per 1000 (1 fewer to 6 more)	⊕⊖⊖⊖ Very low[g,h]	8.6 (2 to 14)	the evidence is very uncertain about the effect on care-home placement
Multifactorial-action with medication-review and self-management strategies (mfa-(w/med+slfm))	OR 1.01 (0.40 to 2.58) Mixed estimate	RR 1.01 (0.40 to 2.49)	51 per 1000 (21 to 119)	1 more per 1000 (29 fewer to 69 more)	1 per 1000 (0 to 3)	0 per 1000 (1 fewer to 2 more)	⊕⊖⊖⊖ Very low[i,j]	9.3 (4 to 14)	the evidence is very uncertain about the effect on care-home placement
Risk-screening (rsk-mfa-)	OR 1.15 (0.62 to 2.13) Mixed estimate	RR 1.14 (0.62 to 2.08)	57 per 1000 (31 to 101)	7 more per 1000 (19 fewer to 51 more)	1 per 1000 (1 to 2)	0 per 1000 (0 to 1 more)	⊕⊖⊖⊖ Very low[g,h]	10.0 (6 to 14)	the evidence is very uncertain about the effect on care-home placement
Education, multifactorial-action and review with medication-review (educ & mfar(w/med))	OR 1.23 (0.61 to 2.49) Mixed estimate	RR 1.22 (0.61 to 2.41)	61 per 1000 (31 to 116)	11 more per 1000 (19 fewer to 66 more)	1 per 1000 (1 to 2)	0 per 1000 (0 to 1 more)	⊕⊖⊖⊖ Very low[g,h]	10.4 (5 to 14)	the evidence is very uncertain about the effect on care-home placement
Education, multifactorial-action and review with medication-review and self-management strategies (educ & mfar(w/med+slfm))	OR 2.19 (0.39 to 12.29) Mixed estimate	RR 2.14 (0.40 to 9.93)	103 per 1000 (20 to 393)	53 more per 1000 (30 fewer to 343 more)	2 per 1000 (0 to 12)	1 more per 1000 (1 fewer to 11 more)	⊕⊖⊖⊖ Very low[g,h]	12.1 (4 to 14)	the evidence is very uncertain about the effect on care-home placement

a: Calculated from OR and an assumed comparator risk of 0.021, the median available care risk among these studies.

b: 1 per 1000 was given as low risk but two available care groups had lower risks than this.

c: very serious concerns about risk of bias due to missing outcome data in the indirect evidence via homecare, multifactorial-action and review (hmcr & mfar) vs available care (ac) comparison. Downgrade twice.

d: serious concerns about imprecision as no closed loop and direct evidence is based on 11 events and 155 persons in homecare, multifactorial-action and review (hmcr & mfar) which do not meet optimal information size. Downgrade once.

e: very serious concerns about risk of bias due to missing outcome data. Downgrade twice.

f: serious concerns about imprecision as no closed loop and direct evidence is based on 14 events and 67 persons in homecare, multifactorial-action and review (hmcr & mfar) which do not meet optimal information size. Downgrade once.

g: serious concerns about risk of bias due to missing outcome data. Downgrade once.

h: very serious concerns about imprecision as confidence interval includes substantial benefit and substantial harm. Downgrade twice.

i: very serious concerns about risk of bias due to randomisation process and missing outcome data. Downgrade twice.

j: very serious concerns about imprecision as confidence interval includes substantial benefit and substantial harm. Already downgraded twice for risk of bias, downgrade once.

Table 76 - Results of care-home placement: medium-term available care network

**rsk-mfa-**													1.15 (0.77, 1.70)
1.41 (0.57, 3.50)	**mfar(w/med)**	1.97 (0.18, 22.00)											0.79 (0.42, 1.47)
2.17 (0.69, 6.83)	1.53 (0.50, 4.66)	**mfar**											0.55 (0.21, 1.48)
1.13 (0.37, 3.47)	0.80 (0.26, 2.51)	0.52 (0.14, 2.00)	**mfa- (w/med+slfm)**										1.01 (0.46, 2.26)
1.25 (0.15, 10.77)	0.89 (0.10, 7.70)	0.58 (0.06, 5.63)	1.11 (0.12, 10.63)	**mfa-(w/med)**									0.91 (0.12, 6.78)
3.58 (0.17, 77.33)	2.53 (0.12, 55.13)	1.65 (0.07, 38.98)	3.17 (0.14, 73.90)	2.86 (0.07, 109.58)	**mfa-**								0.32 (0.02, 6.23)
17.08 (1.98, 147.40)	12.08 (1.38, 105.43)	7.88 (0.81, 76.98)	15.10 (1.57, 145.41)	13.63 (0.74, 251.66)	4.77 (0.12, 182.97)	**hmcr & mfar(w/slfm)**	0.38 (0.10, 1.47)						
6.40 (1.30, 31.61)	4.53 (0.90, 22.70)	2.96 (0.51, 17.18)	5.66 (0.99, 32.31)	5.11 (0.41, 64.21)	1.79 (0.06, 50.84)	0.38 (0.09, 1.60)	**hmcr & mfar**						0.18 (0.04, 0.72)
1.93 (0.37, 9.96)	1.36 (0.26, 7.15)	0.89 (0.15, 5.39)	1.70 (0.29, 10.14)	1.54 (0.12, 19.89)	0.54 (0.02, 15.64)	0.11 (0.01, 1.46)	0.30 (0.04, 2.49)	**educ & rsk-mfa-**					0.59 (0.14, 2.52)
0.52 (0.08, 3.26)	0.37 (0.06, 2.34)	0.24 (0.03, 1.74)	0.46 (0.07, 3.28)	0.42 (0.03, 6.12)	0.15 (0.00, 4.67)	0.03 (0.00, 0.45)	0.08 (0.01, 0.79)	0.27 (0.03, 2.70)	**educ & mfar(w/med+slfm)**				2.19 (0.42, 11.48)
0.93 (0.36, 2.39)	0.66 (0.25, 1.75)	0.43 (0.13, 1.42)	0.83 (0.26, 2.66)	0.75 (0.08, 6.57)	0.26 (0.01, 5.72)	0.05 (0.01, 0.48)	0.15 (0.03, 0.74)	0.48 (0.09, 2.59)	1.79 (0.28, 11.50)	**educ & mfar(w/med)**			1.14 (0.34, 3.82)
1.77 (0.25, 12.67)	1.25 (0.17, 9.07)	0.82 (0.10, 6.69)	1.57 (0.19, 12.62)	1.42 (0.09, 22.80)	0.50 (0.01, 17.05)	0.10 (0.01, 1.68)	0.28 (0.03, 2.98)	0.92 (0.08, 10.21)	3.39 (0.27, 43.01)	1.90 (0.26, 13.96)	**cgn & med & ntr & exrc**		0.65 (0.11, 3.92)
1.20 (0.14, 10.03)	0.85 (0.10, 7.18)	0.55 (0.06, 5.25)	1.06 (0.11, 9.91)	0.96 (0.05, 17.27)	0.33 (0.01, 12.61)	0.07 (0.00, 1.27)	0.19 (0.02, 2.30)	0.62 (0.05, 7.87)	2.29 (0.16, 32.93)	1.28 (0.15, 11.03)	0.68 (0.04, 10.66)	**ADL & ntr & exrc**	0.96 (0.13, 6.89)
1.15 (0.62, 2.13)	0.81 (0.42, 1.57)	0.53 (0.20, 1.39)	1.01 (0.40, 2.58)	0.91 (0.12, 7.18)	0.32 (0.02, 6.48)	0.07 (0.01, 0.53)	0.18 (0.04, 0.78)	0.59 (0.13, 2.72)	2.19 (0.39, 12.29)	1.23 (0.61, 2.49)	0.65 (0.10, 4.17)	0.96 (0.13, 7.29)	**ac**

Lower left triangle presents the findings (OR with 95% CI) of the network meta-analysis. Upper right triangle presents the findings (OR with 95% CI) of pairwise meta-analyses. A OR<1 favours the upper left intervention; a OR>1 favours the lower right intervention. Within the table, comparisons between treatments should be read from left to right (i.e. treatment 1 versus treatment 2). The estimate effect measure (OR and their 95% CI) is in the cell in common between the row- and column-defining treatment.

Table 77 - Intervention rankings for care-home placement: medium-term available care network

Treatment	SUCRA	Pr(Best)	Mean Rank	LCI Rank	UCI Rank
hmcr & mfar(w/slfm)	95.1	65.1	1.6	1	5
hmcr & mfar	85.2	4.6	2.9	1	7
mfa-	67	19.9	5.3	1	14
mfar	63.9	0.5	5.7	2	12
educ & rsk-mfa-	56.6	1.7	6.6	2	14
cgn & med & ntr & exrc	52.6	3.2	7.2	1	14
mfar(w/med)	48.1	0	7.7	4	12
mfa-(w/med)	43.6	2.8	8.3	2	14
adl & ntr & exrc	41.3	2.1	8.6	2	14
ac	37	0	9.2	6	12
mfa-(w/med+slfm)	36.5	0	9.3	4	14
rsk-mfa-	30.6	0	10	6	14
educ & mfar(w/med)	27.6	0	10.4	5	14
educ & mfar(w/med+slfm)	15	0.1	12.1	4	14

SUCRA values (0–100) and mean ranks are presented, based on 1000 simulations. Higher SUCRAs and lower mean ranks indicate better performing interventions. Pr(Best) gives the probability of each specific intervention being ranked best intervention, based on 1000 simulations.

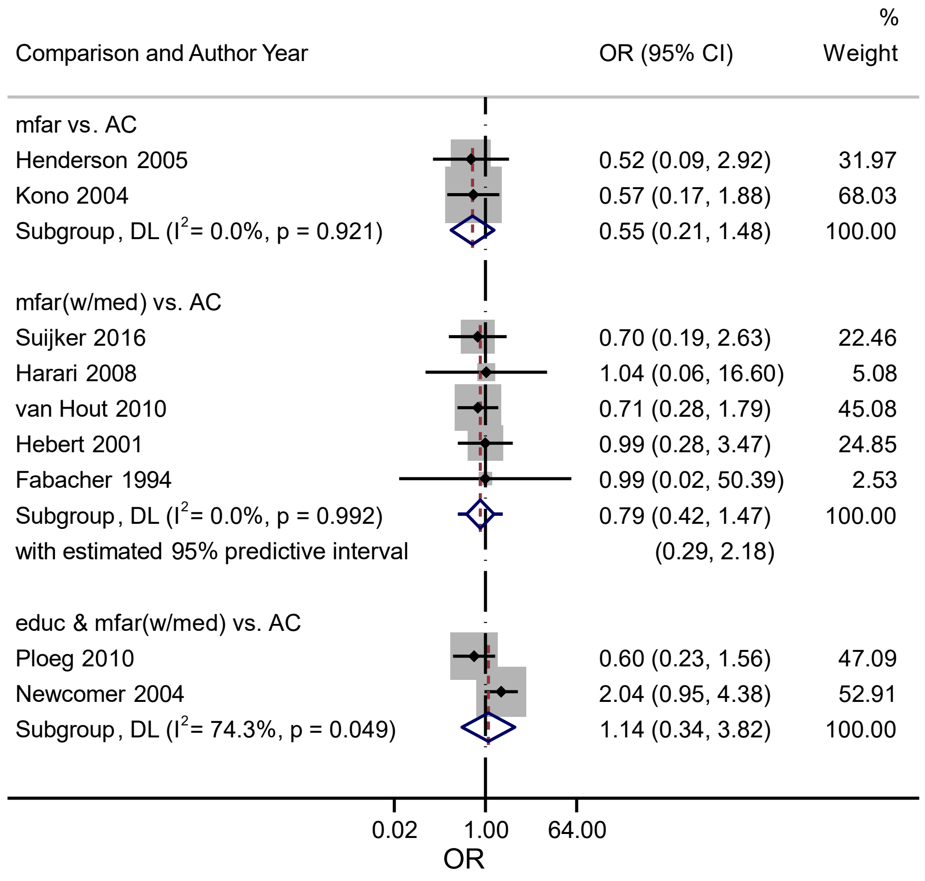

Figure 33 - Pairwise meta-analysis for care-home placement: medium-term available care network (pooling comparisons with greater than one study reporting results)

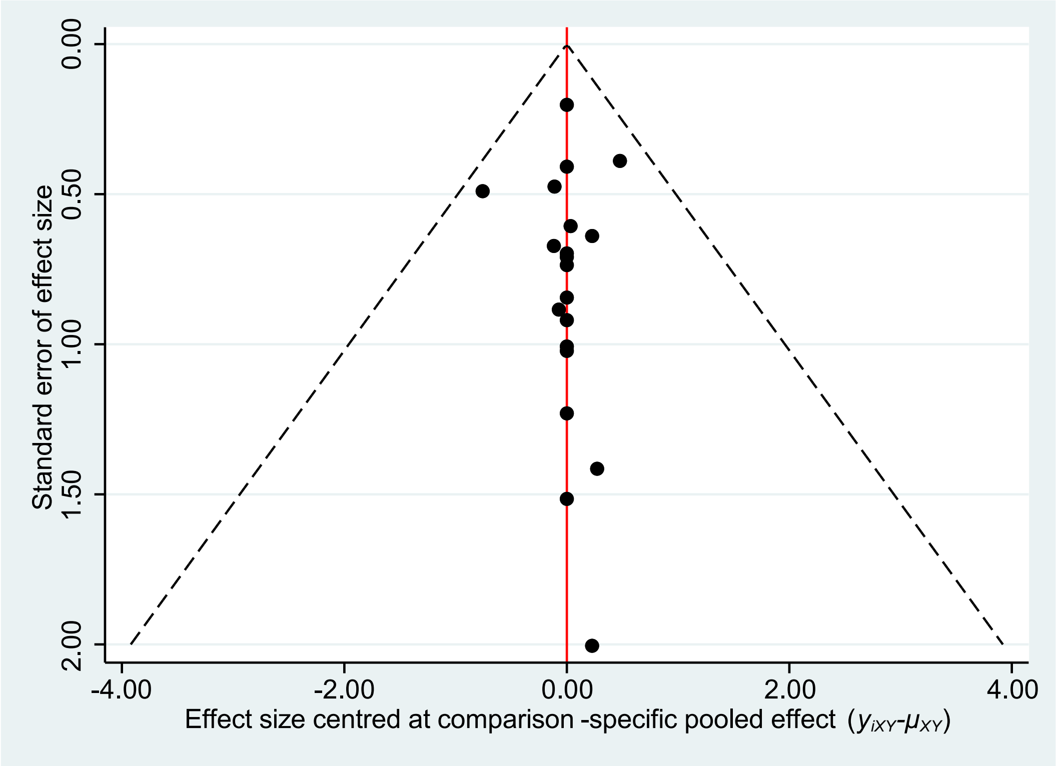

Figure 34 - Comparison-adjusted funnel plot for care-home placement: medium-term available care network

11.6.3 Care-home placement available care network, long-term timeframe

Table 78 - Long-term care-home placement available-care network

					ROB					
**Study**	**Frailty**	**n**	**Experimental group**	**Control group**	**D1**	**D2**	**D3**	**D4**	**D5**	**Overall**
**Carpenter 1990**[516]	all	395	rsk-mfa-	ac	-	-	x	+	-	x
**Fischer 2009**[532]	all	3700	eng & mfa-(w/slfm)	ac	+	-	x	+	-	x
**Ford 1971**[533]	pre-frail and frail	213	mfar(w/med)	ac	+	-	x	+	-	x
**Hay 1998**[546]	unclassifiable	359	mfa-	ac	-	-	x	+	-	x
**Kerse 2014**[556]	pre-frail and frail	3305	rsk-mfa-	ac	+/+	-	x	+	-	x
**Kono 2016**[558]	pre-frail	280	mfar(w/med)	mfar	+	-	x	+	-	x
**Kukkonen-Harjula 2017**[561]	pre-frail and frail	262	ADL & ntr & exrc	ac	+	-	x	+	+	x
**Metzelthin 2013**[576]	frail	290	educ & mfar(w/med+slfm)	ac	-/-	-	x	+	-	x
**Stuck 1995**[602]	all	364	educ & mfar(w/med)	ac	+	-	x	+	-	x
**Stuck 2015**[604]	robust and pre-frail	2045	educ & mfar(w/med+slfm)	ac	+	x	x	+	+	xx
**Suijker 2016**[605]	frail	1776	mfar(w/med)	ac	+/-	-	x	+	-	x
**Thomas 2007**[611]	pre-frail and frail	341	mfar(w/med)	ac	-	-	-	+	-	-
**Tomita 2007**[612]	frail	85	aids	ac	x	-	x	+	-	xx
**Tulloch 1979**[613]	all	223	mfar(w/med)	ac	-	-	x	+	-	x

n: number of participants. ROB: risk of bias. D#: Domain #. D1: risk of bias arising from the randomisation process (individual); or, for cluster trials, risk of bias arising from the randomisation process / risk of bias arising from the identification or recruitment of participants into clusters. D2: risk of bias due to deviations from the intended interventions (effect of assignment to the intervention). D3: risk of bias due to missing outcome data. D4: risk of bias in measurement of the outcome. D5: risk of bias in selection of the reported result. +: low risk of bias; -: some concerns; x: high risk of bias / serious concerns; xx: very serious concerns (overall risk of bias only). all: robust, pre-frail and frail.

Table 79 - Care-home placement in the long term: comparisons with available care summary of findings table.

Population: Older people

Interventions: Community-based complex interventions

Comparator: Available care (ac)

Outcome: care-home placement

Timeframe: long term; range of follow up 24 to 48 months

Setting: Community

Total studies: 14

Total participants: 13638

Comparator rank: Mean 5.4, 95% CI 3 to 8

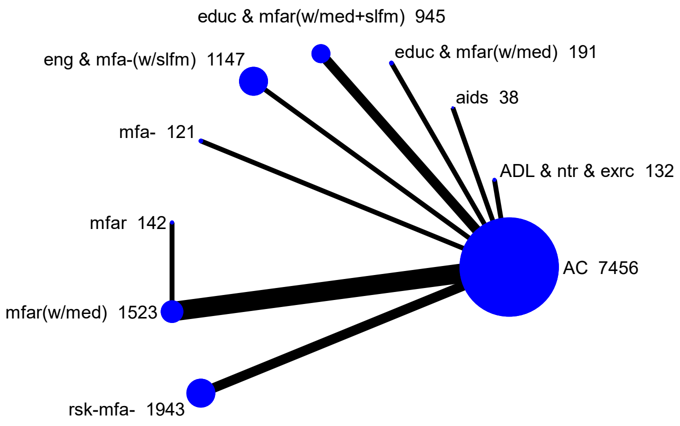

	Relative effect (95% CI)		Anticipated absolute effect (95% CI)						
			High-risk population (200 per 1000 with ac)		Low-risk population (7 per 1000 with ac)				
Intervention group	Network estimate	Calculated risk ratio[a]	With intervention	Difference	With intervention	Difference	Certainty of the evidence (GRADE)	Ranking (95% CI)	Interpretation
Risk-screening (rsk-mfa-)	OR 1.41 (1.06 to 1.88) Mixed estimate	RR 1.39 (1.06 to 1.82)	261 per 1000 (209 to 319)	61 more per 1000 (9 more to 119 more)	10 per 1000 (7 to 13)	3 more per 1000 (0 to 6 more)	⊕⊕⊖⊖ Low[b,c]	8.4 (6 to 10)	may result in an increase in care-home placement
Multifactorial-action and review (mfar)	OR 0.41 (0.07 to 2.26) Indirect estimate	RR 0.42 (0.08 to 2.16)	93 per 1000 (18 to 361)	107 fewer per 1000 (182 fewer to 161 more)	3 per 1000 (1 to 16)	4 fewer per 1000 (6 fewer to 9 more)	⊕⊖⊖⊖ Very low[d,e]	3.1 (1 to 10)	the evidence is very uncertain about the effect on care-home placement
Aids (aids)	OR 0.40 (0.04 to 3.97) Mixed estimate	RR 0.41 (0.04 to 3.58)	90 per 1000 (10 to 498)	110 fewer per 1000 (190 fewer to 298 more)	3 per 1000 (0 to 27)	4 fewer per 1000 (7 fewer to 20 more)	⊕⊖⊖⊖ Very low[f,g]	3.3 (1 to 10)	the evidence is very uncertain about the effect on care-home placement
ADL, nutrition and exercise (ADL & ntr & exrc)	OR 0.79 (0.32 to 1.98) Mixed estimate	RR 0.80 (0.32 to 1.91)	165 per 1000 (73 to 331)	35 fewer per 1000 (127 fewer to 131 more)	6 per 1000 (2 to 14)	1 fewer per 1000 (5 fewer to 7 more)	⊕⊖⊖⊖ Very low[b,e]	4.6 (1 to 10)	the evidence is very uncertain about the effect on care-home placement
Education, multifactorial-action and review with medication-review (educ & mfar(w/med))	OR 0.77 (0.25 to 2.33) Mixed estimate	RR 0.78 (0.26 to 2.22)	161 per 1000 (60 to 369)	39 fewer per 1000 (140 fewer to 169 more)	5 per 1000 (2 to 16)	2 fewer per 1000 (5 fewer to 9 more)	⊕⊖⊖⊖ Very low[b,e]	4.6 (1 to 10)	the evidence is very uncertain about the effect on care-home placement
Multifactorial-action (mfa-)	OR 0.65 (0.07 to 6.34) Mixed estimate	RR 0.66 (0.07 to 5.30)	140 per 1000 (17 to 613)	60 fewer per 1000 (183 fewer to 413 more)	5 per 1000 (0 to 43)	2 fewer per 1000 (7 fewer to 36 more)	⊕⊖⊖⊖ Very low[b,e]	4.7 (1 to 10)	the evidence is very uncertain about the effect on care-home placement
Multifactorial-action and review with medication-review (mfar(w/med))	OR 1.08 (0.72 to 1.62) Mixed estimate	RR 1.08 (0.73 to 1.58)	212 per 1000 (153 to 288)	12 more per 1000 (47 fewer to 88 more)	8 per 1000 (5 to 11)	1 more per 1000 (2 fewer to 4 more)	⊕⊖⊖⊖ Very low[b,e]	6.2 (3 to 10)	the evidence is very uncertain about the effect on care-home placement

Meaningful-activities and multifactorial-action with self-management strategies (eng & mfa-(w/slfm))	OR 1.21 (0.79 to 1.86) Mixed estimate	RR 1.20 (0.79 to 1.80)	232 per 1000 (165 to 317)	32 more per 1000 (35 fewer to 117 more)	8 per 1000 (6 to 13)	1 more per 1000 (1 fewer to 6 more)	⊕⊖⊖⊖ Very low[b,e]	7.3 (3 to 10)	the evidence is very uncertain about the effect on care-home placement
Education, multifactorial-action and review with medication-review and self-management strategies (educ & mfar(w/med+slfm))	OR 1.26 (0.67 to 2.37) Mixed estimate	RR 1.25 (0.68 to 2.25)	240 per 1000 (144 to 372)	40 more per 1000 (56 fewer to 172 more)	9 per 1000 (5 to 16)	2 more per 1000 (2 fewer to 9 more)	⊕⊖⊖⊖ Very low[g,h]	7.4 (3 to 10)	the evidence is very uncertain about the effect on care-home placement

a: Calculated from OR and an assumed comparator risk of 0.037, the median available care risk among these studies.

b: serious concerns about risk of bias due to missing outcome data. Downgrade once.

c: serious concerns about inconsistency (heterogeneity) between studies as their point estimates indicate benefit and harm respectively. Although confidence intervals overlap it is only moderate. $I^2 = 56\%$ Downgrade once.

d: serious concerns about risk of bias due to missing outcome data in the indirect evidence via multifactorial-action and review with medication-review vs available care comparison. Downgrade once.

e: very serious concerns about imprecision as confidence interval includes substantial benefit and substantial harm. Downgrade twice.

f: very serious concerns about risk of bias due to randomisation process and missing outcome data. Downgrade twice.

g: very serious concerns about imprecision as confidence interval includes substantial benefit and harm. Already downgraded twice for risk of bias, downgrade once.

h: very serious concerns about risk of bias due to deviations from the intended interventions and missing data. Downgrade twice.

Table 80 - Results of care-home placement: long-term available care network

**rsk-mfa-**									0.75 (0.10, 5.72)
1.31 (0.80,2.14)	**mfar(w/med)**	2.63 (0.50, 13.80)							1.08 (0.70, 1.65)
3.44 (0.61,19.37)	2.63 (0.50,13.80)	**mfar**							
2.16 (0.22,21.36)	1.65 (0.16,16.64)	0.63 (0.04,10.78)	**mfa-**						0.65 (0.07, 6.34)
1.16 (0.70,1.95)	0.89 (0.49,1.61)	0.34 (0.06,1.97)	0.54 (0.05,5.46)	**eng & mfa- (w/slfm)**					1.21 (0.79, 1.86)
1.12 (0.56,2.23)	0.86 (0.41,1.81)	0.33 (0.05,2.00)	0.52 (0.05,5.48)	0.96 (0.45,2.06)	**educ & mfar(w/med+slfm)**				1.26 (0.67, 2.37)
1.83 (0.58,5.77)	1.40 (0.43,4.57)	0.53 (0.07,4.08)	0.85 (0.07,10.66)	1.57 (0.48,5.17)	1.64 (0.46,5.88)	**educ & mfar(w/med)**			0.77 (0.25, 2.33)
3.56 (0.35,36.28)	2.72 (0.26,28.26)	1.03 (0.06,18.19)	1.65 (0.06,41.96)	3.05 (0.29,31.84)	3.18 (0.29,34.70)	1.94 (0.15,25.06)	**aids**		0.40 (0.04, 3.97)
1.78 (0.68,4.65)	1.36 (0.50,3.71)	0.52 (0.07,3.59)	0.82 (0.07,9.57)	1.53 (0.56,4.21)	1.59 (0.52,4.84)	0.97 (0.23,4.10)	0.50 (0.04,5.98)	**ADL & ntr & exrc**	0.79 (0.32, 1.98)
1.41 (1.06,1.88)	1.08 (0.72,1.62)	0.41 (0.07,2.26)	0.65 (0.07,6.34)	1.21 (0.79,1.86)	1.26 (0.67,2.37)	0.77 (0.25,2.33)	0.40 (0.04,3.97)	0.79 (0.32,1.98)	**ac**

Lower left triangle presents the findings (OR with 95% CI) of the network meta-analysis. Upper right triangle presents the findings (OR with 95% CI) of pairwise meta-analyses. A OR<1 favours the upper left intervention; a OR>1 favours the lower right intervention. Within the table, comparisons between treatments should be read from left to right (i.e. treatment 1 versus treatment 2). The estimate effect measure (OR and their 95% CI) is in the cell in common between the row- and column-defining treatment.

Table 81 - Intervention rankings for care-home placement: long-term available care network

Treatment	SUCRA	Pr(Best)	Mean Rank	LCI Rank	UCI Rank
mfar	76.5	31.2	3.1	1	10
aids	74.2	37.6	3.3	1	10
adl & ntr & exrc	60.5	3.7	4.6	1	10
educ & mfar(w/med)	60.2	6	4.6	1	10
mfa-	58.8	21.2	4.7	1	10
ac	50.7	0	5.4	3	8
mfar(w/med)	42.4	0.1	6.2	3	10
eng & mfa-(w/slfm)	29.9	0	7.3	3	10
educ & mfar(w/med+slfm)	29.1	0.2	7.4	3	10
rsk-mfa	17.7	0	8.4	6	10

SUCRA values (0–100) and mean ranks are presented, based on 1000 simulations. Higher SUCRAs and lower mean ranks indicate better performing interventions. Pr(Best) gives the probability of each specific intervention being ranked best intervention, based on 1000 simulations.

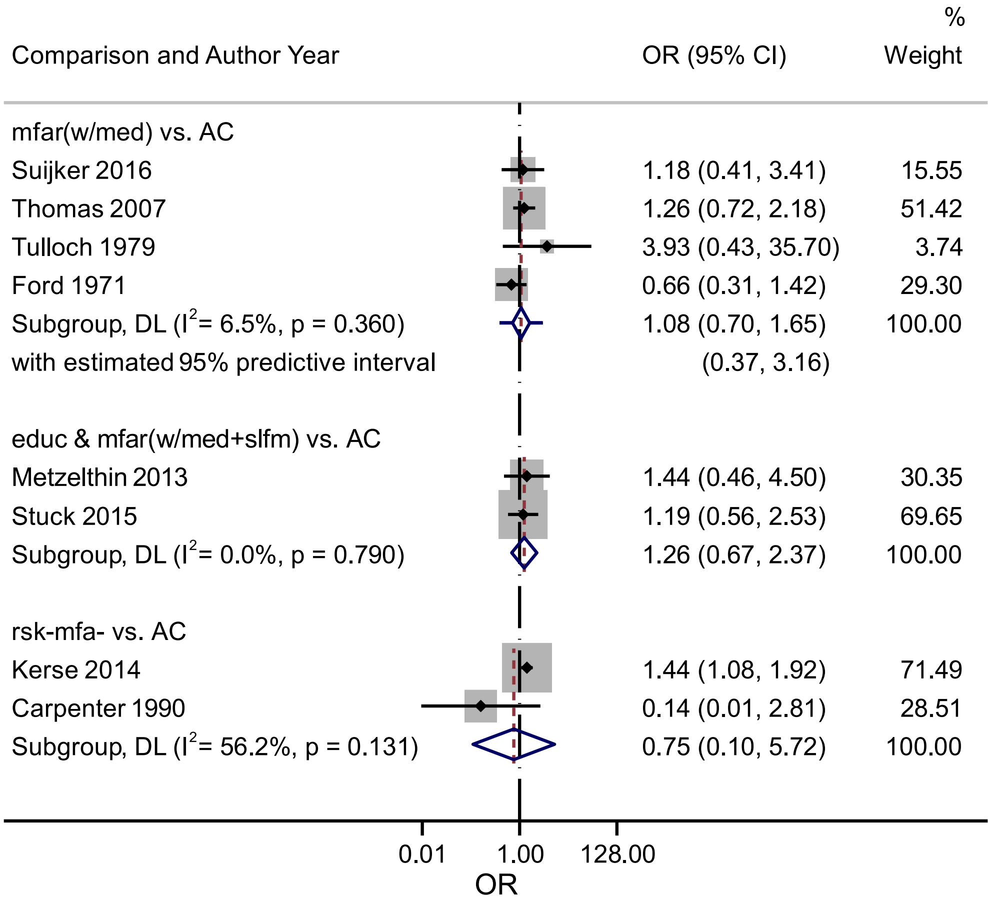

Figure 35 - Pairwise meta-analysis for care-home placement: long-term available care network (pooling comparisons with greater than one study reporting results)

11.6.4 Care-home placement homecare network, short-term timeframe

No results as there were too few comparisons to conduct network meta-analysis.

11.6.5 Care-home placement homecare network, medium-term timeframe

Table 82 - Medium-term care-home placement homecare network

					ROB					
**Study**	**Frailty**	**n**	**Experimental group**	**Control group**	**D1**	**D2**	**D3**	**D4**	**D5**	**Overall**
**Fernandez-Barres 2017**[531]	frail	129	hmcr & ntr	hmcr	+	-	x	+	-	x
**Lewin 2013**[565]	frail	607	hmcr & educ & mfar	hmcr	x	x	x	+	-	xx
**Rooijackers 2021**[594]	frail	232	hmcr & ADL & mfar(w/slfm)	hmcr	+/-	-	x	+	-	x
**Wolter 2013**[629]	frail	599	hmcr & mfar(w/med)	hmcr	+/-	-	x	+	-	x

n: number of participants. ROB: risk of bias. D#: Domain #. D1: risk of bias arising from the randomisation process (individual); or, for cluster trials, risk of bias arising from the randomisation process / risk of bias arising from the identification or recruitment of participants into clusters. D2: risk of bias due to deviations from the intended interventions (effect of assignment to the intervention). D3: risk of bias due to missing outcome data. D4: risk of bias in measurement of the outcome. D5: risk of bias in selection of the reported result. +: low risk of bias; -: some concerns; x: high risk of bias / serious concerns; xx: very serious concerns (overall risk of bias only). all: robust, pre-frail and frail.

Table 83. Care-home placement in the medium term: comparisons with homecare summary of findings table.

Population: Older people

Interventions: Community-based complex interventions

Comparator: Homecare (hmcr)

Outcome: care-home placement

Timeframe: medium term; range of follow up 12 to 13 months

Setting: Community

Total studies: 4

Total participants: 1567

Comparator rank: Mean 2.9, 95% CI 1 to 5

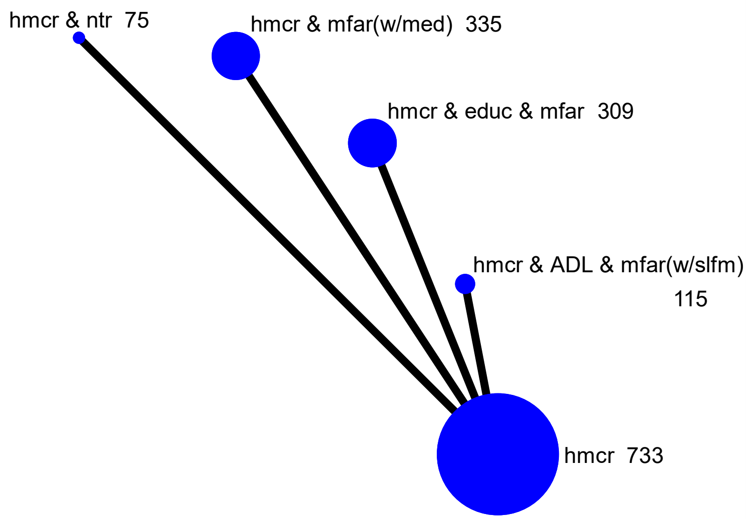

Intervention group	Relative effect (95% CI): Network estimate	Relative effect (95% CI): Calculated risk ratio[a]	High-risk population (182 per 1000 with hmcr): With intervention	High-risk population (182 per 1000 with hmcr): Difference	Low-risk population (85 per 1000 with hmcr): With intervention	Low-risk population (85 per 1000 with hmcr): Difference	Certainty of the evidence (GRADE)	Ranking (95% CI)	Interpretation
Homecare, education, multifactorial-action and review (hmcr & educ & mfar)	OR 0.86 (0.55 to 1.35) Mixed estimate	RR 0.88 (0.59 to 1.29)	161 per 1000 (110 to 231)	21 fewer per 1000 (72 fewer to 49 more)	74 per 1000 (49 to 111)	11 fewer per 1000 (36 fewer to 26 more)	⊕⊖⊖⊖ Very low[b,c]	2.2 (1 to 5)	the evidence is very uncertain about the effect on care-home placement
Homecare, ADL, multifactorial-action and review with self-management strategies (hmcr & ADL & mfar(w/slfm))	OR 0.91 (0.35 to 2.33) Mixed estimate	RR 0.92 (0.39 to 1.97)	168 per 1000 (73 to 341)	14 fewer per 1000 (109 fewer to 159 more)	78 per 1000 (32 to 178)	7 fewer per 1000 (53 fewer to 93 more)	⊕⊖⊖⊖ Very low[d,e]	2.6 (1 to 5)	the evidence is very uncertain about the effect on care-home placement
Homecare, multifactorial-action and review with medication-review (hmcr & mfar(w/med))	OR 1.12 (0.75 to 1.70) Mixed estimate	RR 1.11 (0.77 to 1.55)	200 per 1000 (142 to 274)	18 more per 1000 (40 fewer to 92 more)	95 per 1000 (65 to 136)	10 more per 1000 (20 fewer to 51 more)	⊕⊖⊖⊖ Very low[d,e]	3.6 (1 to 5)	the evidence is very uncertain about the effect on care-home placement

Homecare and nutrition (hmcr & ntr)	OR 1.37 (0.48 to 3.98) Mixed estimate	RR 1.31 (0.51 to 2.83)	234 per 1000 (96 to 470)	52 more per 1000 (86 fewer to 288 more)	113 per 1000 (42 to 270)	28 more per 1000 (43 fewer to 185 more)	⊕⊖⊖⊖ Very low[d,e]	3.8 (1 to 5)	the evidence is very uncertain about the effect on care-home placement

a: Calculated from OR and an assumed comparator risk of 0.136, the median available care risk among these studies.

b: very serious concerns about risk of bias due to randomisation process, participants were not analysed according to allocation, and missing outcome data. Downgrade twice.

c: very serious concerns about imprecision as confidence interval includes substantial benefit and substantial harm. Already downgraded twice for risk of bias, downgrade once.

d: serious concerns about risk of bias due to missing outcome data. Downgrade once.

e: very serious concerns about imprecision as confidence interval includes substantial benefit and substantial harm. Downgrade twice.

Table 84 - Results of care-home placement: medium-term homecare network

**hmcr & ntr**				1.37 (0.48,3.98)
1.22 (0.39,3.82)	**hmcr & mfar(w/med)**			1.13 (0.75,1.70)
1.59 (0.50,5.03)	1.30 (0.71,2.38)	**hmcr & educ & mfar**		0.86 (0.55,1.35)
1.51 (0.37,6.25)	1.24 (0.44,3.45)	0.95 (0.34,2.69)	**hmcr & ADL & mfar(w/slfm)**	0.91 (0.35,2.33)
1.37 (0.48,3.98)	1.13 (0.75,1.70)	0.86 (0.55,1.35)	0.91 (0.35,2.33)	**hmcr**

Lower left triangle presents the findings (OR with 95% CI) of the network meta-analysis. Upper right triangle presents the findings (OR with 95% CI) of pairwise meta-analyses. A OR<1 favours the upper left intervention; a OR>1 favours the lower right intervention. Within the table, comparisons between treatments should be read from left to right (i.e. treatment 1 versus treatment 2). The estimate effect measure (OR and their 95% CI) is in the cell in common between the row- and column-defining treatment.

Table 85 - Intervention rankings for care-home placement: medium-term homecare network

Treatment	SUCRA	Pr(Best)	Mean Rank	LCI Rank	UCI Rank
hmcr & educ & mfar	71.2	36.4	2.2	1	5
hmcr & ADL & mfar(w/slfm)	60.1	38.1	2.6	1	5
hmcr	52.5	6.8	2.9	1	5
hmcr & mfar(w/med)	35.8	4.9	3.6	1	5
hmcr & ntr	30.4	13.8	3.8	1	5

SUCRA values (0–100) and mean ranks are presented, based on 1000 simulations. Higher SUCRAs and lower mean ranks indicate better performing interventions. Pr(Best) gives the probability of each specific intervention being ranked best intervention, based on 1000 simulations.

11.6.6 Care-home placement homecare network, long-term timeframe

No results as there were too few comparisons to conduct network meta-analysis.

11.7 Health status

11.7.1 Health status available care network (medium-term timeframe only)

Table 86 - Medium-term health status available-care network

Study	Frailty	n	Experimental group	Control group	ROB D1	D2	D3	D4	D5	Overall
**Blom 2016**[510]	all	844	mfa-(w/med+slfm)	ac	x/+	-	x	-	+	xx
**Bouman 2008**[513]	pre-frail and frail	293	mfar(w/med)	ac	+	-	x	-	-	x
**Brettschneider 2015**[514]	frail	278	mfar(w/med)	ac	-	-	x	-	+	x
**Cameron 2013**[515]	frail	215	exrc & mfar(w/med+slfm)	ac	+	-	-	-	+	-
**Serra-Prat 2017**[597]	pre-frail	133	ntr & exrc	ac	-	-	x	-	-	x
**Szanton 2019**[607]	pre-frail and frail	260	ADL&aids&educ&exrc& mfar(w/med+slfm)	ac	+	-	x	-	-	x
**Takahashi 2012**[608]	frail	166	mntr-mfa-	ac	-	-	x	-	+	x
**Thomas 2007**[611]	pre-frail and frail	442	mfar(w/med)	ac	-	-	x	-	-	x

n: number of participants. ROB: risk of bias. D#: Domain #. D1: risk of bias arising from the randomisation process (individual); or, for cluster trials, risk of bias arising from the randomisation process / risk of bias arising from the identification or recruitment of participants into clusters. D2: risk of bias due to deviations from the intended interventions (effect of assignment to the intervention). D3: risk of bias due to missing outcome data. D4: risk of bias in measurement of the outcome. D5: risk of bias in selection of the reported result. +: low risk of bias; -: some concerns; x: high risk of bias / serious concerns; xx: very serious concerns (overall risk of bias only). all: robust, pre-frail and frail.

Table 87 - Self-reported health in the medium term: comparisons with available care summary of findings table.

Population: Older people

Interventions: Community-based complex interventions

Comparator: Available care (ac)

Outcome: self-reported health (single question)

Timeframe: medium term; range of follow up 12 to 18 months

Setting: Community

Total studies: 8

Total participants: 2631

Comparator rank: Mean 4.4, 95% CI 2 to 6

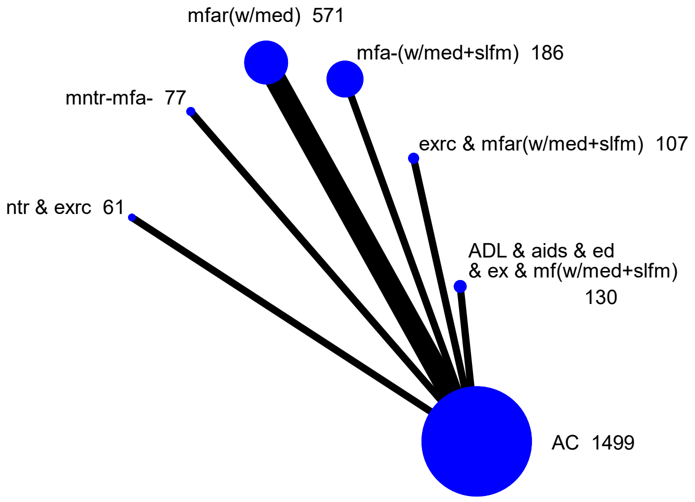

Intervention group	Anticipated absolute effect (95% CI) SMD	Anticipated absolute effect (95% CI) MD (EQ-VAS, 0 to 100)[a]	Certainty of the evidence (GRADE)	Ranking (95% CI)	Interpretation
Exercise, multifactorial-action and review with medication-review and self-management strategies (exrc & mfar(w/med+slfm))	SMD 0.01 lower (0.34 lower to 0.32 higher) Mixed estimate	MD 0.20 lower (6.94 lower to 6.53 higher)	⊕⊕⊖⊖ Low[b]	4.3 (1 to 7)	may result in little to no difference in self-reported health
Multifactorial-action and review with medication-review (mfar(w/med))	SMD 0.11 higher (0.06 lower to 0.28 higher) Mixed estimate	MD 2.24 higher (1.22 lower to 5.71 higher)	⊕⊖⊖⊖ Very low[b,c]	2.6 (1 to 6)	the evidence is very uncertain about the effect on self-reported health
Multifactorial-action with medication-review and self-management strategies (mfa-(w/med+slfm))	SMD 0.07 higher (0.18 lower to 0.32 higher) Mixed estimate	MD 1.43 higher (3.67 lower to 6.53 higher)	⊕⊖⊖⊖ Very low[d,e]	3.2 (1 to 7)	the evidence is very uncertain about the effect on self-reported health
Nutrition and exercise (ntr & exrc)	SMD 0.07 higher (0.33 lower to 0.46 higher) Mixed estimate	MD 1.43 higher (6.73 lower to 9.38 higher)	⊕⊖⊖⊖ Very low[b,c]	3.4 (1 to 7)	the evidence is very uncertain about the effect on self-reported health
ADL, aids, education, exercise, multifactorial-action and review with medication-review and self-management strategies (ADL & aids & ed & ex & mf(w/med+slfm))	SMD 0.06 lower (0.38 lower to 0.25 higher) Mixed estimate	MD 1.22 lower (7.75 lower to 5.10 higher)	⊕⊖⊖⊖ Very low[b,c]	4.9 (1 to 7)	the evidence is very uncertain about the effect on self-reported health
Monitoring (mntr-mfa-)	SMD 0.11 lower (0.47 lower to 0.26 higher) Mixed estimate	MD 2.24 lower (9.59 lower to 5.30 higher)	⊕⊖⊖⊖ Very low[b,c]	5.3 (1 to 7)	the evidence is very uncertain about the effect on self-reported health

a: calculated from the estimated SMD using a standard deviation of 20.4, the pooled standard deviation across all intervention groups reporting EQ-VAS (0 to 100) included in this NMA.

b: very serious concerns about imprecision as confidence interval includes substantial benefit and harm (SMD +/- 0.05). Downgrade twice.

c: serious concerns about risk of bias due to missing outcome data. Downgrade once.

d: very serious concerns about risk of bias due to missing outcome data and uncertainty about the randomisation procedure combined with a large imbalance in cluster sizes.

e: very serious concerns about imprecision as confidence interval includes substantial benefit and harm (SMD +/- 0.05). Already downgraded twice for risk of bias, downgrade once.

Table 88 - Results of Health status: medium-term available care network

**ntr & exrc**						0.07 (-0.27,0.41)
0.17 (-0.36,0.71)	**mntr-mfa-**					-0.11 (-0.41,0.20)
-0.04 (-0.47,0.38)	-0.21 (-0.61,0.19)	**mfar(w/med)**				0.11 (-0.06,0.28)
-0.00 (-0.47,0.46)	-0.18 (-0.62,0.27)	0.04 (-0.27,0.34)	**mfa-(w/med+slfm)**			0.07 (-0.09,0.23)
0.08 (-0.44,0.59)	-0.10 (-0.59,0.40)	0.12 (-0.25,0.49)	0.08 (-0.34,0.50)	**exrc & mfar(w/med+slfm)**		-0.01 (-0.28,0.26)
0.13 (-0.37,0.63)	-0.04 (-0.52,0.44)	0.17 (-0.18,0.53)	0.14 (-0.27,0.54)	0.05 (-0.40,0.51)	**ADL&aids&ed&ex&mf(w/med+slfm)**	-0.06 (-0.31,0.18)
0.07 (-0.33,0.46)	-0.11 (-0.47,0.26)	0.11 (-0.06,0.28)	0.07 (-0.18,0.32)	-0.01 (-0.34,0.32)	-0.06 (-0.38,0.25)	**ac**

Lower left triangle presents the findings (SMD with 95% CI) of the network meta-analysis. Upper right triangle presents the findings (SMD with 95% CI) of pairwise meta-analyses. A SMD>1 favours the upper left intervention; a SMD<1 favours the lower right intervention. Within the table, comparisons between treatments should be read from left to right (i.e. treatment 1 versus treatment 2). The estimate effect measure (SMD and their 95% CI) is in the cell in common between the row- and column-defining treatment.

Table 89 - Intervention rankings for Health status: medium-term available care network

Treatment	SUCRA	Pr(Best)	Mean Rank	LCI Rank	UCI Rank
mfar(w/med)	74.1	24.4	2.6	1	6
mfa-(w/med+slfm)	64	22.3	3.2	1	7
ntr & exrc	59.2	27.4	3.4	1	7
exrc & mfar(w/med+slfm)	44.7	12.3	4.3	1	7
ac	44.1	0.2	4.4	2	6
adl&aids&ed&ex&mf(w/med+slfm)	35	6.6	4.9	1	7
mntr-mfa-	28.9	6.8	5.3	1	7

SUCRA values (0–100) and mean ranks are presented, based on 1000 simulations. Higher SUCRAs and lower mean ranks indicate better performing interventions. Pr(Best) gives the probability of each specific intervention being ranked best intervention, based on 1000 simulations.

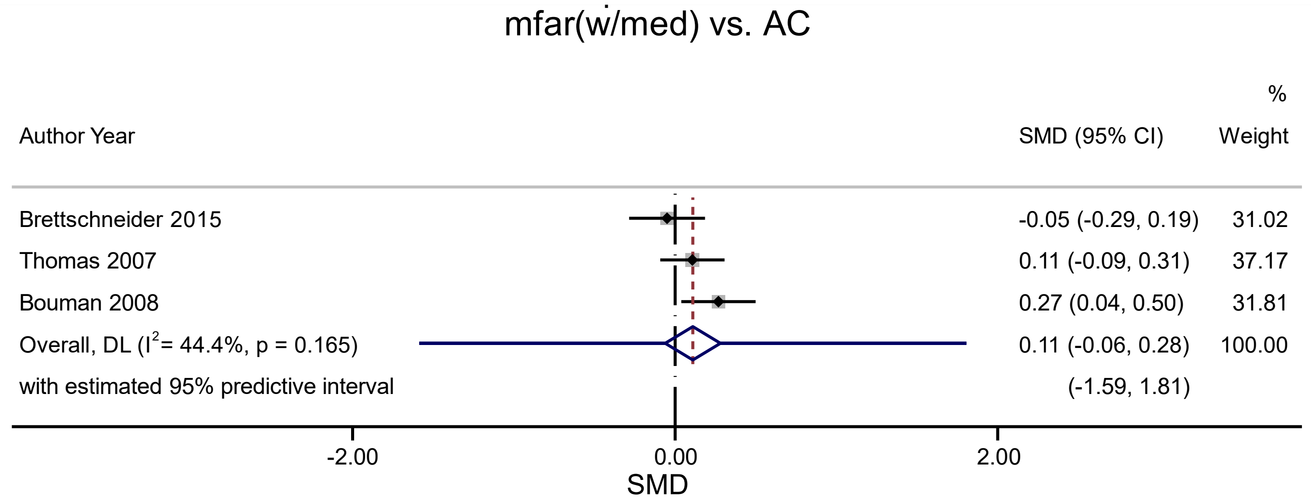

Figure 36 - Pairwise meta-analysis for Health status: medium-term available care network (pooling comparisons with greater than one study reporting results)

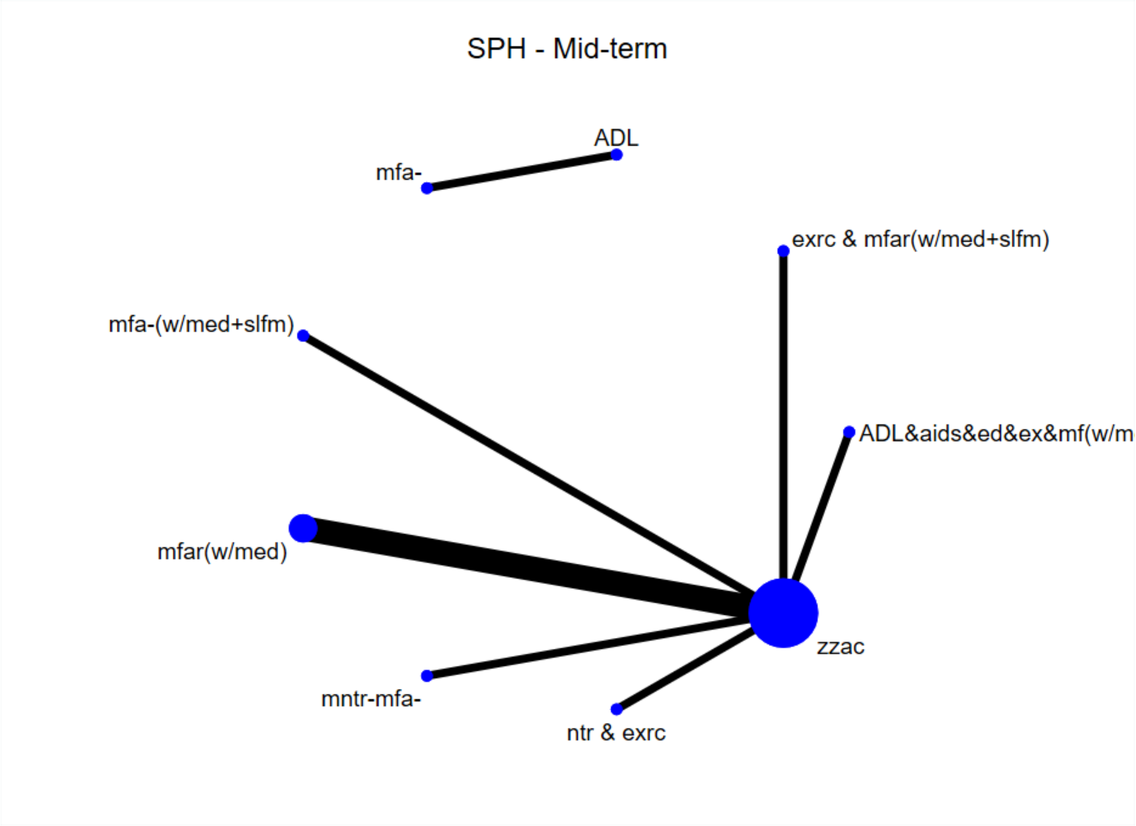

Figure 37 - Example of disconnected network for health status medium-term available care network

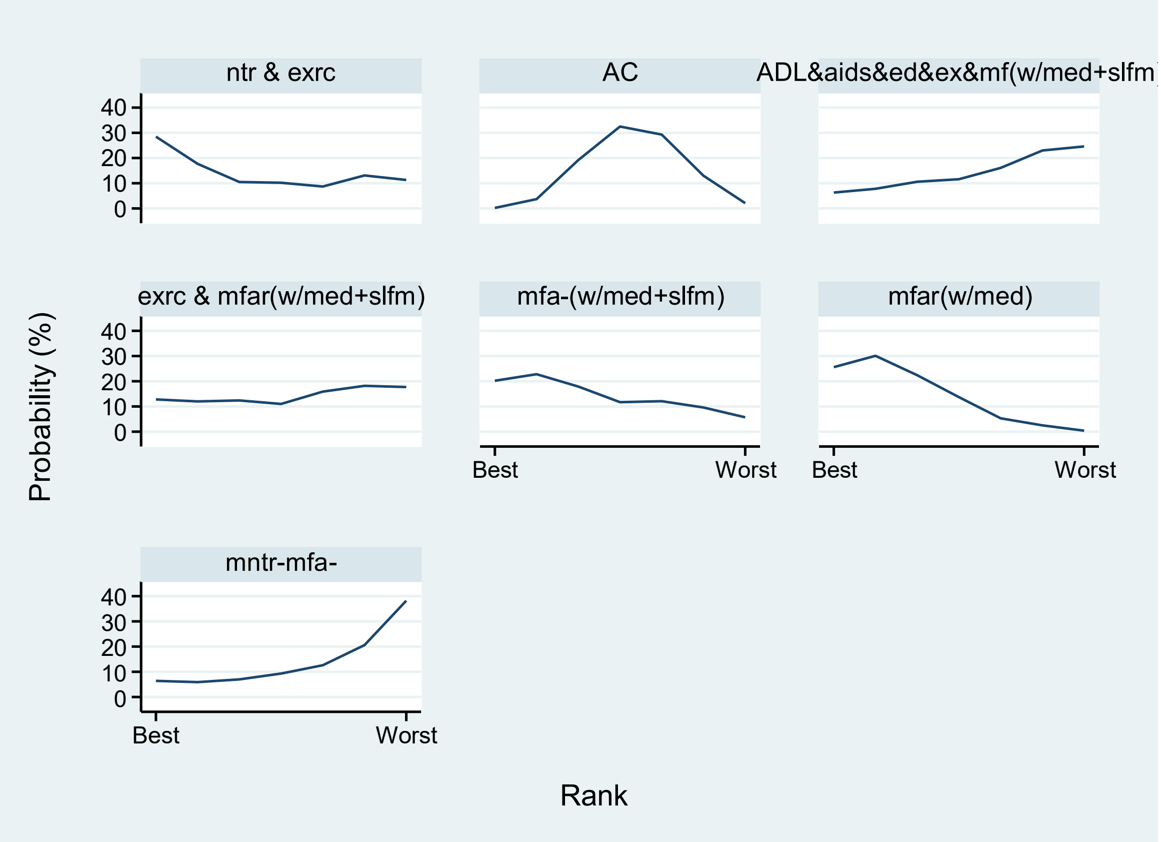

Figure 38 - Rankogram showing comparative effectiveness of interventions for Health Status medium-term available care network. Results based on a simulation of 1000 replications

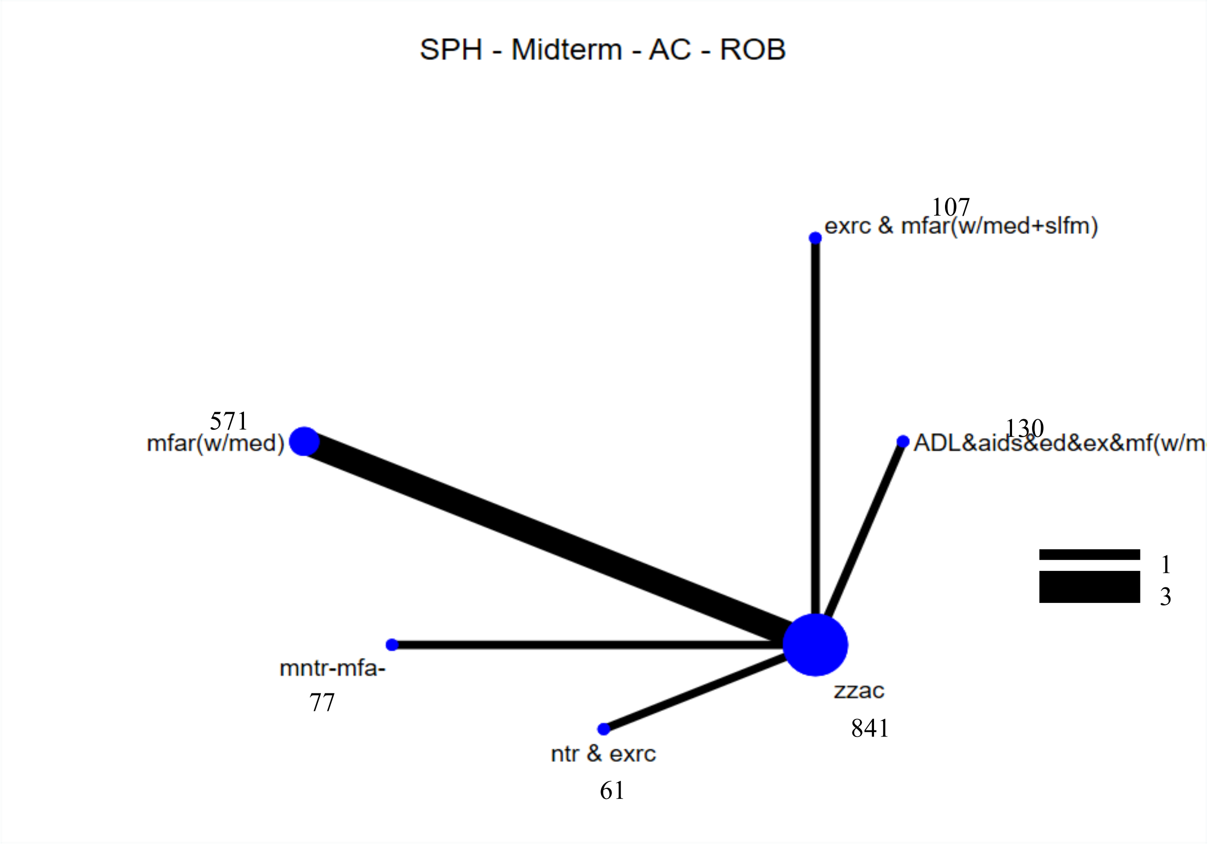

Figure 39 - Network plot for risk of bias analysis for Health Status medium-term available care network

Table 90 - Results of Risk of Bias analysis for Health Status: medium-term available care network

**ntr & exrc**					0.07 (-0.27, 0.41)
0.17 (-0.36,0.71)	**mntr-mfa-**				-0.11 (-0.41, 0.20)
-0.04 (-0.47,0.38)	-0.21 (-0.61,0.19)	**mfar(w/med)**			0.11 (-0.06, 0.28)
0.08 (-0.44,0.59)	-0.10 (-0.59,0.40)	0.12 (-0.25,0.49)	**exrc & mfar(w/med+slfm)**		-0.01 (-0.28, 0.26)
0.13 (-0.37,0.63)	-0.04 (-0.52,0.44)	0.17 (-0.18,0.53)	0.05 (-0.40,0.51)	**ADL&aids&ed&ex&mf(w/med+slfm)**	-0.06 (-0.31, 0.18)
0.07 (-0.33,0.46)	-0.11 (-0.47,0.26)	0.11 (-0.06,0.28)	-0.01 (-0.34,0.32)	-0.06 (-0.38,0.25)	**AC**

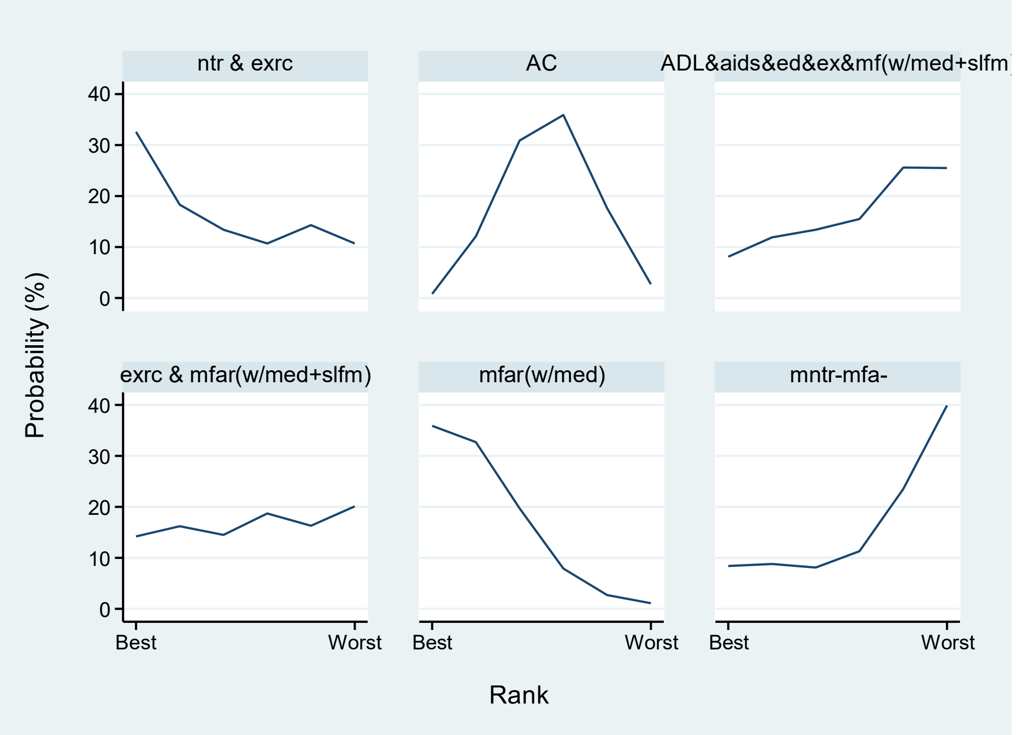

Figure 40 - Rankogram showing comparative effectiveness of interventions for risk of bias sensitivity analysis for Health Status medium-term available care network. Results based on a simulation of 1000 replications

11.7.2 Health status homecare network (medium-term timeframe only)

No results as there were too few comparisons to conduct network meta-analysis.

11.8 Depression

11.8.1 Depression available care network (medium-term timeframe only)

Table 91 - Medium-term depression available-care network

Study	Frailty	n	Experimental group	Control group	ROB D1	D2	D3	D4	D5	Overall
**Bleijenberg 2016**[509]	pre-frail and frail	2489	rsk-mfa-	ac	x/+	-	x	+	-	xx
**Blom 2016**[510]	all	1379	mfa-(w/med+slfm)	ac	x/+	-	x	-	-	xx
**Bouman 2008**[513]	pre-frail and frail	293	mfar(w/med)	ac	+	-	x	-	-	x
**Cameron 2013**[515]	frail	214	exrc & mfar(w/med+slfm)	ac	+	-	-	-	+	-
**Clark 1997**[519]	robust and pre-frail	283	eng & educ	ac	x	-	x	-	x	xx
**Cutchin 2009**[523]	unclassifiable	110	mfar	ac	-	-	x	-	-	x
**Gustafson 2021**[541]	all	390	aids & educ & comm	ac	+	-	x	-	-	x
**Henderson 2005**[548]	robust	124	mfar	ac	+/x	+	x	+	-	xx
**Kono 2016**[558]	pre-frail	360	mfar(w/med)	mfar	+	-	x	-	-	x
**Metzelthin 2013**[576]	frail	317	educ & mfar(w/med+slfm)	ac	-/-	-	x	-	-	x
**Newbury 2001**[582]	unclassifiable	89	mfa-(w/med)	ac	-	-	x	-	-	x
**Rubenstein 2007**[595]	frail	694	mfar(w/med)	ac	-	-	-	-	-	-
**Szanton 2019**[607]	pre-frail and frail	260	ADL&aids&educ&exrc& mfar(w/med+slfm)	ac	+	-	x	-	-	x
**Takahashi 2012**[608]	frail	166	mntr-mfa-	ac	-	-	x	-	+	x
**van Heuvelen 2005**[617]	pre-frail and frail	77	exrc & psyc	ac	-	x	x	-	-	xx

n: number of participants. ROB: risk of bias. D#: Domain #. D1: risk of bias arising from the randomisation process (individual); or, for cluster trials, risk of bias arising from the randomisation process / risk of bias arising from the identification or recruitment of participants into clusters. D2: risk of bias due to deviations from the intended interventions (effect of assignment to the intervention). D3: risk of bias due to missing outcome data. D4: risk of bias in measurement of the outcome. D5: risk of bias in selection of the reported result. +: low risk of bias; -: some concerns; x: high risk of bias / serious concerns; xx: very serious concerns (overall risk of bias only). all: robust, pre-frail and frail.

Table 92 - Depression in the medium term: comparisons with available care summary of findings table

Population: Older people

Interventions: Community-based complex interventions

Comparator: Available care (ac)

Outcome: Depression

Timeframe: medium term; range of follow up 44 weeks to 18 months

Setting: Community

Total studies: 15

Total participants: 7245

Comparator rank: Mean 7.6, 95% CI 5 to 11

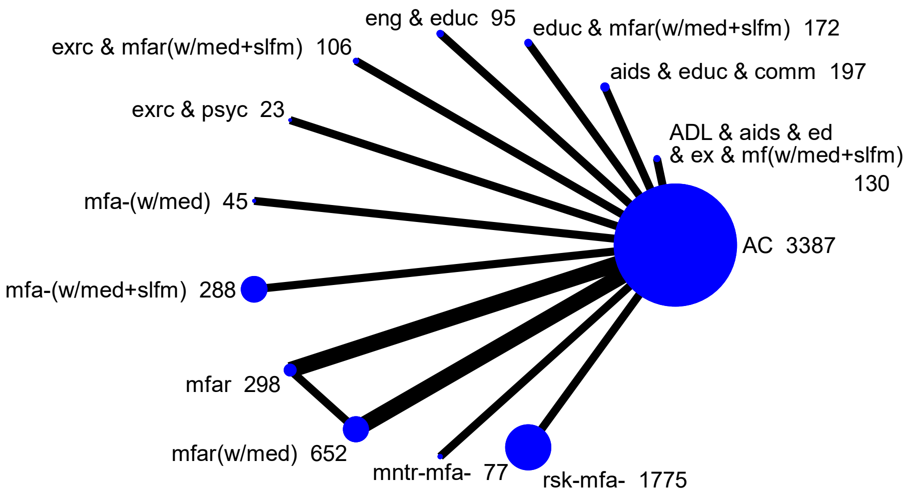

Intervention group	Anticipated absolute effect (95% CI): SMD	Anticipated absolute effect (95% CI): MD (Geriatric Depression Scale (GDS 15))[a]	Certainty of the evidence (GRADE)	Ranking (95% CI)	Interpretation
Exercise, multifactorial-action and review with medication-review and self-management strategies (exrc & mfar(w/med+slfm))	SMD 0.11 lower (0.45 lower to 0.23 higher) Mixed estimate	MD 0.35 lower (1.41 lower to 0.72 higher)	⊕⊕⊖⊖ **Low**[b]	4.9 (1 to 12)	may result in a very slight reduction in symptoms of depression
Meaningful-activities and education (eng & educ)	SMD 0.13 lower (0.46 lower to 0.19 higher) Mixed estimate	MD 0.42 lower (1.44 lower to 0.59 higher)	⊕⊖⊖⊖ **Very low**[c,d]	4.7 (1 to 12)	the evidence is very uncertain about the effect on symptoms of depression
Risk-screening (rsk-mfa-)	SMD 0.09 lower (0.31 lower to 0.14 higher) Mixed estimate	MD 0.28 lower (0.98 lower to 0.43 higher)	⊕⊖⊖⊖ **Very low**[d,e]	5.3 (1 to 11)	the evidence is very uncertain about the effect on symptoms of depression
Multifactorial-action and review with medication-review (mfar(w/med))	SMD 0.07 lower (0.25 lower to 0.12 higher) Mixed estimate	MD 0.21 lower (0.80 lower to 0.37 higher)	⊕⊖⊖⊖ **Very low**[b,f]	5.6 (1 to 11)	the evidence is very uncertain about the effect on symptoms of depression
Aids, education and telecoms (aids & educ & comm)	SMD 0.05 lower (0.33 lower to 0.24 higher) Mixed estimate	MD 0.15 lower (1.05 lower to 0.76 higher)	⊕⊖⊖⊖ **Very low**[b,f]	6.2 (1 to 13)	the evidence is very uncertain about the effect on symptoms of depression
Exercise and psychology (exrc & psyc)	SMD 0.06 lower (0.59 lower to 0.47 higher) Mixed estimate	MD 0.20 lower (1.87 lower to 1.47 higher)	⊕⊖⊖⊖ **Very low**[d,g]	6.2 (1 to 13)	the evidence is very uncertain about the effect on symptoms of depression

ADL, aids, education, exercise, multifactorial-action and review with medication-review and self-management strategies (ADL & aids & ed & ex & mf(w/med+slfm))	SMD 0.01 lower (0.33 lower to 0.31 higher) Mixed estimate	MD 0.03 lower (1.03 lower to 0.97 higher)	⊕⊖⊖⊖ **Very low**[b,f]	7.2 (1 to 13)	the evidence is very uncertain about the effect on symptoms of depression
Monitoring (mntr-mfa-)	SMD 0.00 (0.37 lower to 0.37 higher) Mixed estimate	MD 0.00 (1.16 lower to 1.16 higher)	⊕⊖⊖⊖ **Very low**[b,f]	7.3 (1 to 13)	the evidence is very uncertain about the effect on symptoms of depression
Multifactorial-action with medication-review and self-management strategies (mfa-(w/med+slfm))	SMD 0.00 (0.24 lower to 0.24 higher) Mixed estimate	MD 0.00 (0.77 lower to 0.77 higher)	⊕⊖⊖⊖ **Very low**[d,h]	7.5 (2 to 13)	the evidence is very uncertain about the effect on symptoms of depression
Multifactorial-action and review (mfar)	SMD 0.04 higher (0.20 lower to 0.28 higher) Mixed estimate	MD 0.13 higher (0.63 lower to 0.88 higher)	⊕⊖⊖⊖ **Very low**[b,f]	8.6 (2 to 13)	the evidence is very uncertain about the effect on symptoms of depression
Multifactorial-action with medication-review (mfa-(w/med))	SMD 0.11 higher (0.35 lower to 0.58 higher) Mixed estimate	MD 0.36 higher (1.10 lower to 1.82 higher)	⊕⊖⊖⊖ **Very low**[b,f]	9.3 (1 to 13)	the evidence is very uncertain about the effect on symptoms of depression
Education, multifactorial-action and review with medication-review and self-management strategies (educ & mfar(w/med+slfm))	SMD 0.17 higher (0.13 lower to 0.47 higher) Mixed estimate	MD 0.53 higher (0.42 lower to 1.48 higher)	⊕⊖⊖⊖ **Very low**[b,f]	10.6 (3 to 13)	the evidence is very uncertain about the effect on symptoms of depression

a: calculated from the estimated SMD using a standard deviation of 3.15, the pooled standard deviation across intervention groups reporting the GDS-15 in the medium term.

b: very serious concerns about imprecision as confidence interval includes substantial benefit and harm (SMD +/- 0.05). Downgrade twice.

c: very serious concerns about risk of bias due to randomisation process, missing outcome data, and reported results were not analysed according to allocation. Downgrade twice.

d: very serious concerns about imprecision as confidence interval includes substantial benefit and harm (SMD +/- 0.05). Already downgraded twice for risk of bias, downgrade once.

e: very serious concerns about risk of bias due to randomisation process and missing outcome data. Downgrade twice.

f: serious concerns about risk of bias due to missing outcome data. Downgrade once.

g: very serious concerns about risk of bias due to excluding participants in per-protocol analysis and missing outcome data. Downgrade twice.

h: very serious concerns about risk of bias due to randomisation process and missing outcome data. Downgrade twice.

Table 93 - Results of Depression: medium-term available care network

**rsk-mfa-**												-0.09<br>(-0.18, -0.00)
-0.09<br>(-0.52,0.34)	**mntr-mfa-**											0.00<br>(-0.31, 0.31)
-0.02<br>(-0.31,0.27)	0.07<br>(-0.34,0.48)	**mfar(w/med)**	0.00<br>(-0.21, 0.21)									-0.13<br>(-0.26, -0.00)
-0.13<br>(-0.46,0.20)	-0.04<br>(-0.48,0.40)	-0.11<br>(-0.33,0.12)	**mfar**									0.15<br>(-0.19, 0.49)
-0.09<br>(-0.42,0.24)	-0.00<br>(-0.44,0.44)	-0.07<br>(-0.37,0.24)	0.04<br>(-0.30,0.38)	**mfa-<br>(w/med+slfm)**								0.00<br>(-0.13, 0.13)
-0.20<br>(-0.72,0.31)	-0.11<br>(-0.71,0.48)	-0.18<br>(-0.68,0.32)	-0.07<br>(-0.60,0.45)	-0.11<br>(-0.64,0.41)	**mfa-(w/med)**							0.11<br>(-0.30, 0.53)
-0.03<br>(-0.60,0.55)	0.06<br>(-0.58,0.71)	-0.00<br>(-0.57,0.56)	0.10<br>(-0.48,0.68)	0.06<br>(-0.52,0.65)	0.18<br>(-0.53,0.88)	**exrc & psyc**						-0.06<br>(-0.55, 0.43)
0.02<br>(-0.38,0.43)	0.11<br>(-0.39,0.61)	0.04<br>(-0.34,0.43)	0.15<br>(-0.26,0.57)	0.11<br>(-0.31,0.53)	0.23<br>(-0.35,0.80)	0.05<br>(-0.58,0.68)	**exrc &<br>mfar(w/med+sl<br>fm)**					-0.11<br>(-0.38, 0.16)
0.05<br>(-0.35,0.44)	0.13<br>(-0.35,0.62)	0.07<br>(-0.30,0.44)	0.17<br>(-0.23,0.58)	0.13<br>(-0.27,0.54)	0.25<br>(-0.32,0.81)	0.07<br>(-0.55,0.69)	0.02<br>(-0.44,0.49)	**eng & educ**				-0.13<br>(-0.38, 0.11)
-0.26<br>(-0.63,0.12)	-0.17<br>(-0.64,0.31)	-0.24<br>(-0.59,0.12)	-0.13<br>(-0.51,0.26)	-0.17<br>(-0.56,0.22)	-0.05<br>(-0.61,0.50)	-0.23<br>(-0.84,0.38)	-0.28<br>(-0.73,0.18)	-0.30<br>(-0.74,0.14)	**educ &<br>mfar(w/med+sl<br>fm)**			0.17<br>(-0.05, 0.39)
-0.04<br>(-0.40,0.32)	0.05<br>(-0.42,0.51)	-0.02<br>(-0.36,0.32)	0.09<br>(-0.29,0.46)	0.05<br>(-0.33,0.42)	0.16<br>(-0.38,0.71)	-0.02<br>(-0.62,0.59)	-0.06<br>(-0.51,0.38)	-0.09<br>(-0.52,0.34)	0.21<br>(-0.20,0.63)	**aids & educ &<br>comm**		-0.05<br>(-0.24, 0.15)
-0.08<br>(-0.47,0.31)	0.01<br>(-0.48,0.50)	-0.06<br>(-0.43,0.31)	0.05<br>(-0.35,0.45)	0.01<br>(-0.39,0.41)	0.12<br>(-0.44,0.69)	-0.05<br>(-0.67,0.56)	-0.10<br>(-0.57,0.36)	-0.12<br>(-0.58,0.33)	0.18<br>(-0.26,0.62)	-0.04<br>(-0.47,0.39)	**ADL&aids&ed<br>&ex&mf(w/me<br>d+slfm)**	-0.01<br>(-0.25, 0.23)
-0.09<br>(-0.31,0.14)	-0.00<br>(-0.37,0.37)	-0.07<br>(-0.25,0.12)	0.04<br>(-0.20,0.28)	-0.00<br>(-0.24,0.24)	0.11<br>(-0.35,0.58)	-0.06<br>(-0.59,0.47)	-0.11<br>(-0.45,0.23)	-0.13<br>(-0.46,0.19)	0.17<br>(-0.13,0.47)	-0.05<br>(-0.33,0.24)	-0.01<br>(-0.33,0.31)	**ac**

Lower left triangle presents the findings (SMD with 95% CI) of the network meta-analysis. Upper right triangle presents the findings (SMD with 95% CI) of pairwise meta-analyses. A SMD<1 favours the upper left intervention; a SMD>1 favours the lower right intervention. Within the table, comparisons between treatments should be read from left to right (i.e. treatment 1 versus treatment 2). The estimate effect measure (SMD and their 95% CI) is in the cell in common between the row- and column-defining treatment.

Table 94 - Intervention rankings for Depression: medium-term available care network

Treatment	SUCRA	Pr(Best)	Mean Rank	LCI Rank	UCI Rank
eng & educ	69.2	20.2	4.7	1	12
exrc & mfar(w/med+slfm)	67.7	18.7	4.9	1	12
rsk-mfa-	64.2	7.7	5.3	1	11
mfar(w/med)	62.1	2.9	5.6	1	11
aids & educ & comm	56.4	6.9	6.2	1	13
exrc & psyc	56.4	21.1	6.2	1	13
ADL&aids&ed&ex&mf(w/med+slfm)	48.4	5.7	7.2	1	13
mntr-mfa-	47.7	8.6	7.3	1	13
mfa-(w/med+slfm)	45.5	2.3	7.5	2	13
ac	45.1	0	7.6	5	11
mfar	36.5	1.3	8.6	2	13
mfa-(w/med)	31.1	4.2	9.3	1	13
educ & mfar(w/med+slfm)	19.7	0.4	10.6	3	13

SUCRA values (0–100) and mean ranks are presented, based on 1000 simulations. Higher SUCRAs and lower mean ranks indicate better performing interventions. Pr(Best) gives the probability of each specific intervention being ranked best intervention, based on 1000 simulations.

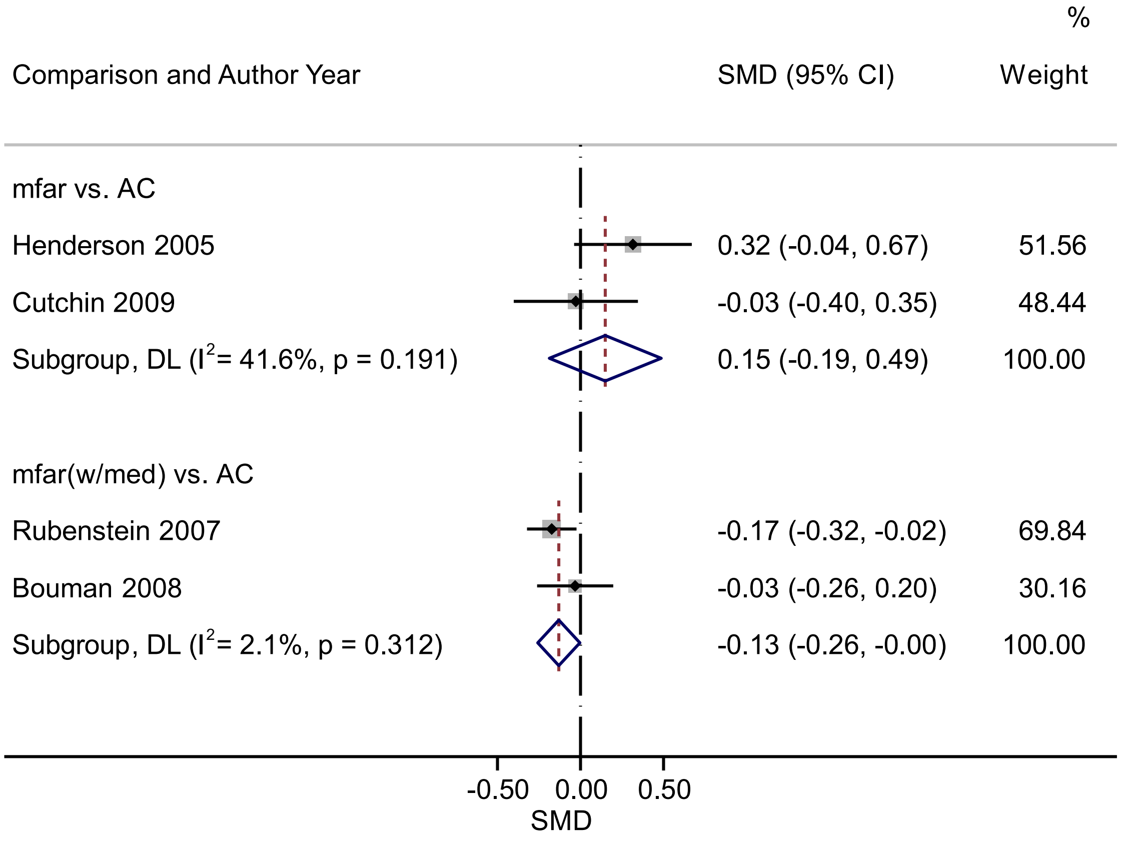

Figure 41 - Pairwise meta-analysis for depression: medium-term available care network (pooling comparisons with greater than one study reporting results)

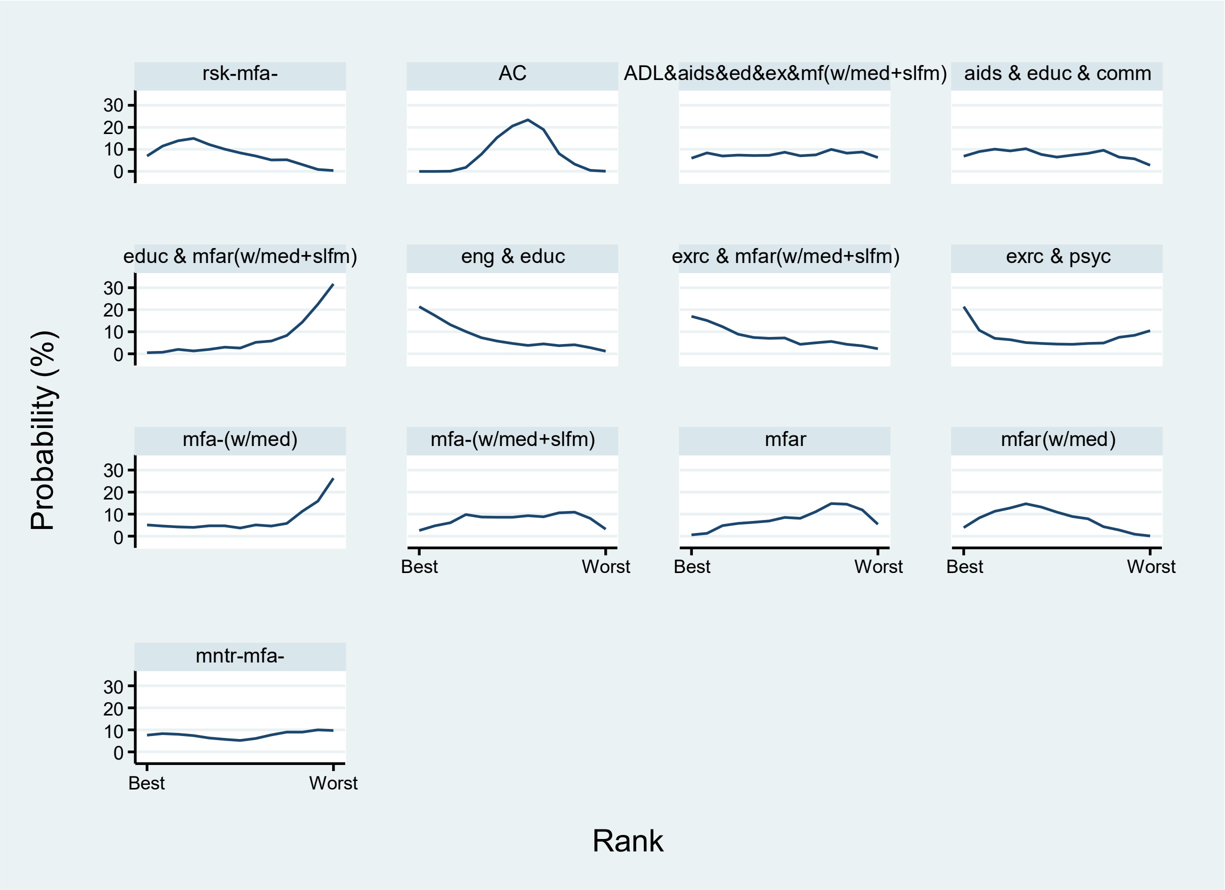

Figure 42 - Rankogram showing comparative effectiveness of interventions for depression medium-term available care network. Results based on a simulation of 1000 replications.

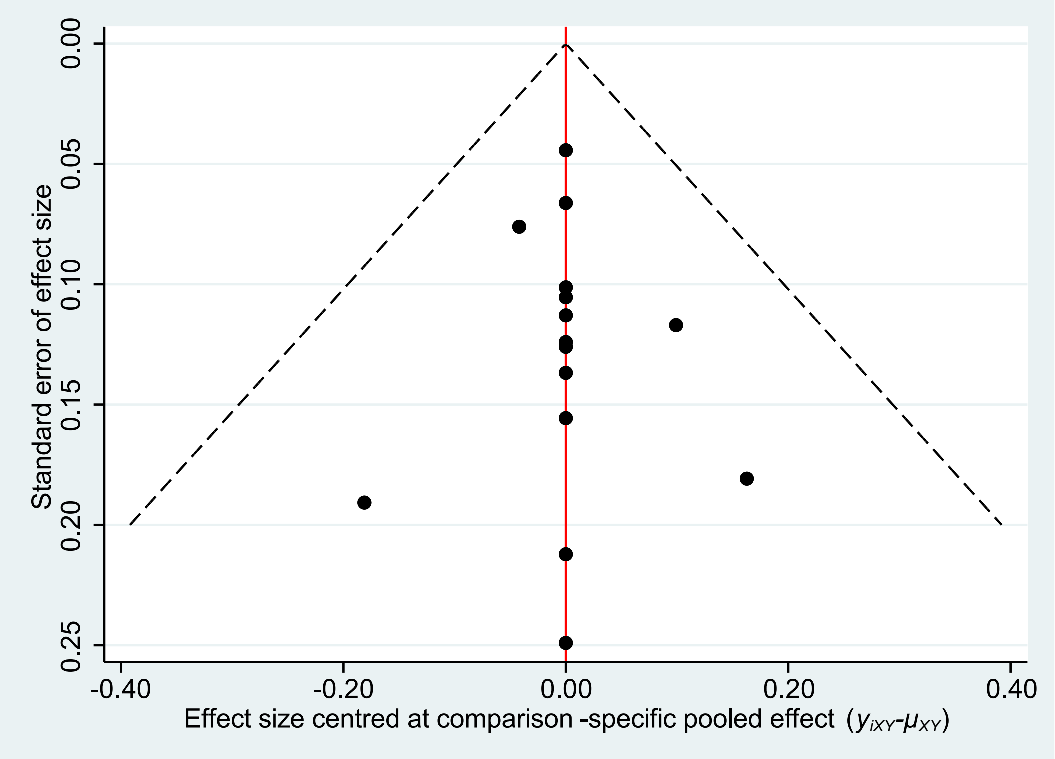

Figure 43 - Comparison-adjusted funnel plot for depression: medium-term available care network

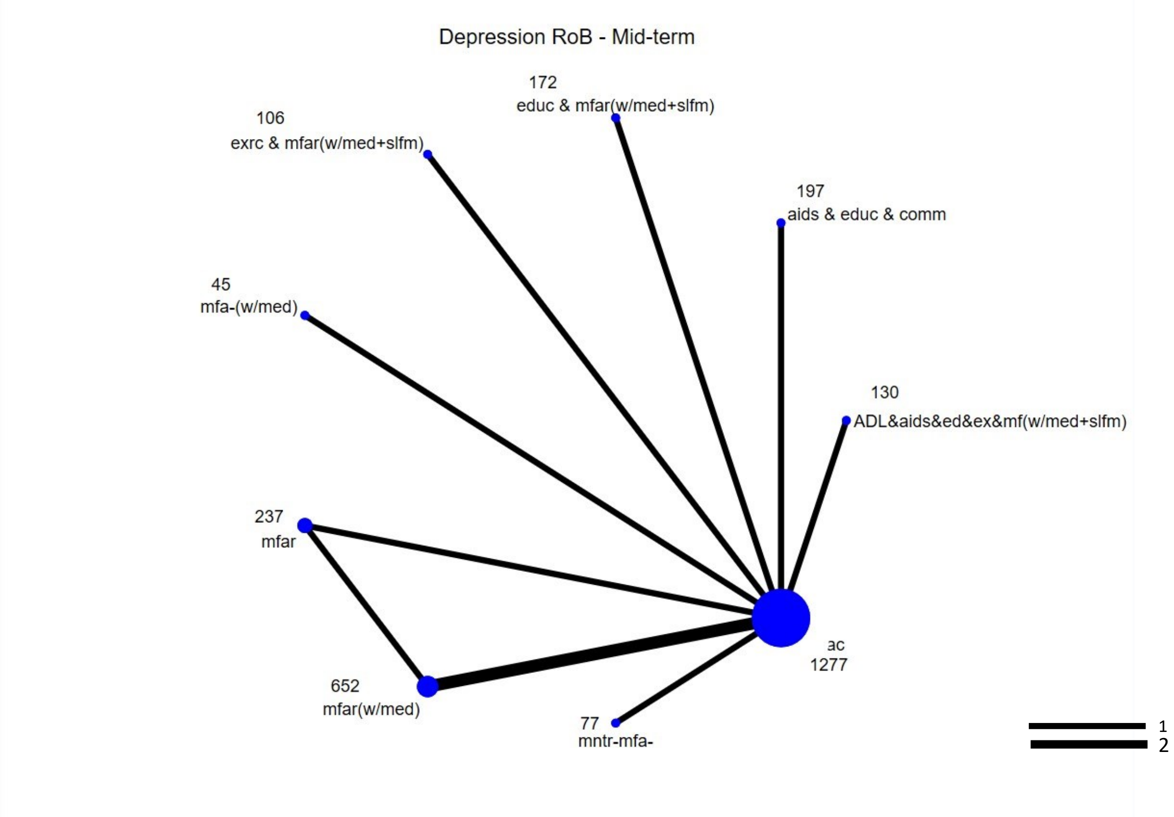

Figure 44 - Network plot for risk of bias analysis for depression medium-term available care network

Table 95 - Intervention rankings for risk of bias analysis for depression: medium-term available care network

Treatment	SUCRA	PrBest	Mean Rank	95% CI for true rank
mfar(w/med)	77.8	16.0	2.8	1 - 6
exrc & mfar(w/med+slfm)	69.9	30.4	3.4	1 - 8
mfar	69.0	17.9	3.5	1 - 8
aids & educ & comm	56.7	8.9	4.5	1 - 8
ADL&aids&ed&ex&mf(w/med+slfm)	47.9	9.0	5.2	1 - 9
mntr-mfa-	46.1	10.4	5.3	1 - 9
ac	42.0	0.0	5.6	3 - 8
mfa-(w/med)	27.5	7.1	6.8	1 - 9
educ & mfar(w/med+slfm)	13.1	0.3	8.0	4 - 9

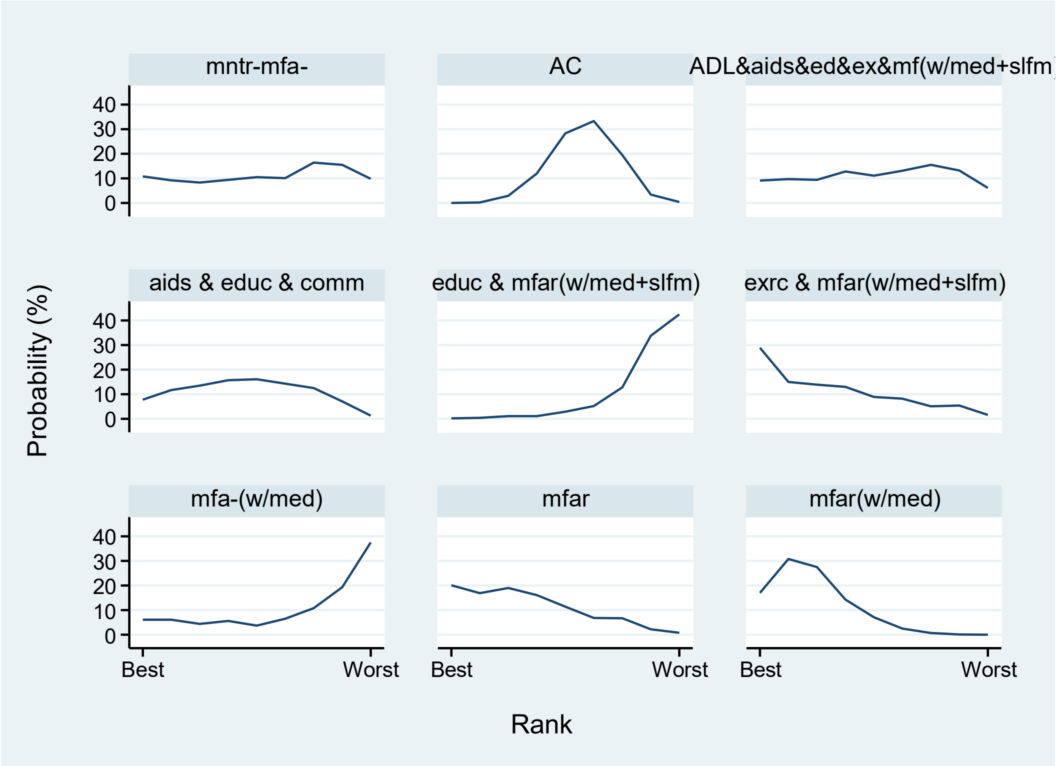

Figure 45 - Rankogram showing comparative effectiveness of interventions for risk of bias sensitivity analysis for depression medium-term available care network. Results based on a simulation of 1000 replications.

Table 96 - Results of risk of bias depression: medium-term available care network

**mntr-mfa-**								0.00 (-0.31, 0.31)
0.12 (-0.20,0.45)	**mfar(w/med)**	0.00 (-0.21, 0.21)						-0.13 (-0.26, -0.00)
0.10 (-0.27,0.47)	-0.02 (-0.21,0.16)	**mfar**						-0.03 (-0.40, 0.35)
-0.11 (-0.63,0.40)	-0.24 (-0.67,0.20)	-0.22 (-0.68,0.25)	**mfa-(w/med)**					0.11 (-0.30, 0.53)
0.11 (-0.30,0.52)	-0.01 (-0.31,0.28)	0.01 (-0.33,0.35)	0.23 (-0.27,0.72)	**exrc & mfar(w/med+slfm)**				-0.11 (-0.38, 0.16)
-0.17 (-0.55,0.21)	-0.29 (-0.54,-0.04)	-0.27 (-0.57,0.03)	-0.05 (-0.52,0.42)	-0.28 (-0.63,0.07)	**educ & mfar(w/med+slfm)**			0.17 (-0.05, 0.39)
0.05 (-0.32,0.41)	-0.08 (-0.31,0.16)	-0.05 (-0.34,0.23)	0.16 (-0.30,0.62)	-0.06 (-0.40,0.27)	0.21 (-0.08,0.51)	**aids & educ & comm**		-0.05 (-0.24, 0.15)
0.01 (-0.38,0.40)	-0.11 (-0.38,0.16)	-0.09 (-0.41,0.23)	0.12 (-0.36,0.61)	-0.10 (-0.46,0.26)	0.18 (-0.15,0.51)	-0.04 (-0.35,0.28)	**ADL&aids&ed&ex& mf(w/med+slfm)**	-0.01 (-0.25, 0.23)
-0.00 (-0.31,0.31)	-0.12 (-0.24,-0.00)	-0.10 (-0.30,0.10)	0.11 (-0.30,0.53)	-0.11 (-0.38,0.16)	0.17 (-0.05,0.39)	-0.05 (-0.24,0.15)	-0.01 (-0.25,0.23)	**AC**

11.8.2 Depression homecare network (medium-term timeframe only)

Table 97 - Medium-term depression homecare network

					ROB					
**Study**	**Frailty**	**n**	**Experimental group**	**Control group**	**D1**	**D2**	**D3**	**D4**	**D5**	**Overall**
**Bernabei 1998**[508]	frail	199	hmcr & mfar(w/med)	hmcr	-	-	x	-	-	x
**Fernandez-Barres 2017**[531]	frail	111	hmcr & ntr	hmcr	+	-	x	-	-	x
**Parsons M 2012**[587]	frail	251	hmcr & mfar	hmcr & mfa-	+/-	-	x	-	-	x
**Parsons M 2017**[586]	frail	113	hmcr & ADL & mfar(w/slfm)	hmcr & mfa-	-	-	x	-	-	xx
**Rooijackers 2021**[594]	frail	264	hmcr & ADL & mfar(w/slfm)	hmcr	+/-	-	x	+	x	xx
**Teut 2013**[609]	frail	58	hmcr & hmnt & exrc	hmcr	+/+	-	x	-	-	x

n: number of participants. ROB: risk of bias. D#: Domain #. D1: risk of bias arising from the randomisation process (individual); or, for cluster trials, risk of bias arising from the randomisation process / risk of bias arising from the identification or recruitment of participants into clusters. D2: risk of bias due to deviations from the intended interventions (effect of assignment to the intervention). D3: risk of bias due to missing outcome data. D4: risk of bias in measurement of the outcome. D5: risk of bias in selection of the reported result. +: low risk of bias; -: some concerns; x: high risk of bias / serious concerns; xx: very serious concerns (overall risk of bias only).

Table 98 - Depression in the medium term: comparisons with homecare summary of findings table

Population: Older people

Interventions: Community-based complex interventions

Comparator: Homecare (hmcr)

Outcome: Depression

Timeframe: medium term; follow up at 12 months

Setting: Community

Total studies: 6

Total participants: 996

Comparator rank: Mean 5.2, 95% CI 3 to 7

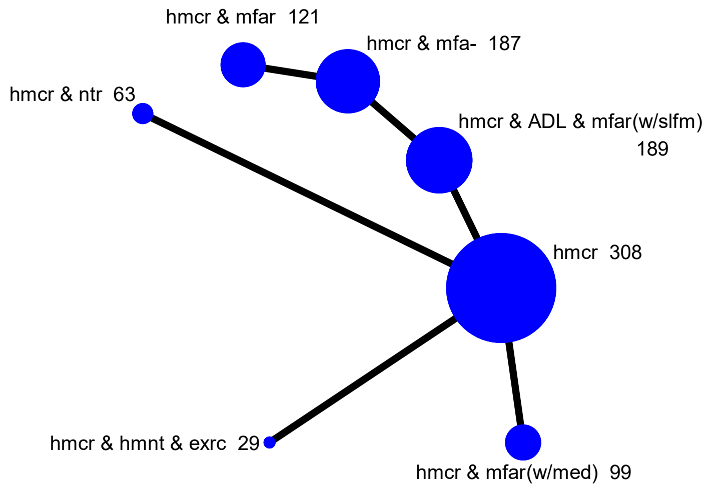

Intervention group	Anticipated absolute effect (95% CI)		Certainty of the evidence (GRADE)	Ranking (95% CI)	Interpretation
	SMD	MD (Geriatric Depression Scale (GDS 15))[a]			
Homecare, multifactorial-action and review with medication-review (hmcr & mfar(w/med))	SMD 0.38 lower (0.66 lower to 0.10 lower) Mixed estimate	MD 1.20 lower (2.08 lower to 0.31 lower)	⊕⊕⊖⊖ **Low**[b,c]	1.7 (1 to 4)	may result in a slight reduction in symptoms of depression
Homecare and nutrition (hmcr & ntr)	SMD 0.24 lower (0.62 lower to 0.14 higher) Mixed estimate	MD 0.76 lower (1.95 lower to 0.43 higher)	⊕⊖⊖⊖ **Very low**[b,d]	2.8 (1 to 7)	the evidence is very uncertain about the effect on symptoms of depression
Homecare, ADL, multifactorial-action and review with self-management strategies (hmcr & ADL & mfar(w/slfm))	SMD 0.09 lower (0.33 lower to 0.16 higher) Mixed estimate	MD 0.27 lower (1.03 lower to 0.49 higher)	⊕⊖⊖⊖ **Very low**[e,f]	4.0 (2 to 7)	the evidence is very uncertain about the effect on symptoms of depression
Homecare and multifactorial-action (hmcr & mfa-)	SMD 0.09 lower (0.53 lower to 0.35 higher) Indirect estimate	MD 0.28 lower (1.67 lower to 1.11 higher)	⊕⊖⊖⊖ **Very low**[d,g]	4.0 (1 to 6)	the evidence is very uncertain about the effect on symptoms of depression
Homecare, alternative-medicine and exercise (hmcr & hmnt & exrc)	SMD 0.06 lower (0.58 lower to 0.45 higher) Mixed estimate	MD 0.20 lower (1.82 lower to 1.42 higher)	⊕⊖⊖⊖ **Very low**[b,d]	4.4 (1 to 7)	the evidence is very uncertain about the effect on symptoms of depression
Homecare, multifactorial-action and review (hmcr & mfar)	SMD 0.10 higher (0.40 lower to 0.61 higher) Indirect estimate	MD 0.32 higher (1.27 lower to 1.92 higher)	⊕⊖⊖⊖ **Very low**[d,h]	5.9 (2 to 7)	the evidence is very uncertain about the effect on symptoms of depression

a: calculated from the estimated SMD using a standard deviation of 3.15, the pooled standard deviation across all intervention groups reporting the GDS-15 in the medium term.

b: serious concerns about risk of bias due to missing outcome data. Downgrade once.

c: serious concerns about imprecision as no closed loop and direct evidence is based on 99 persons in homecare, multifactorial-action and review with medication-review (hmcr & mfar(w/med)) which does not meet optimal information size. Downgrade once.

d: very serious concerns about imprecision as confidence interval includes substantial benefit and harm (SMD +/- 0.05). Downgrade twice.

e: very serious concerns about risk of bias due to missing outcome data and the reported results were not analysed in accordance with the protocol. Downgrade twice.

f: very serious concerns about imprecision as confidence interval includes substantial benefit and harm (SMD +/- 0.05). Already downgraded twice for risk of bias, downgrade once.

g: very serious concerns about risk of bias due to missing outcome data and the reported results were not analysed in accordance with the protocol in the indirect evidence via homecare, ADL, multifactorial-action and review with self-management strategies (hmcr & ADL & mfar(w/slfm)) vs homecare (hmcr) comparison. Downgrade twice.

h: very serious concerns about risk of bias due to missing outcome data and the reported results were not analysed in accordance with the protocol in the indirect evidence via homecare, ADL, multifactorial-action and review with self-management strategies (hmcr & ADL & mfar(w/slfm)) vs homecare (hmcr) comparison and via homecare, ADL, multifactorial-action and review with self-management strategies (hmcr & ADL & mfar(w/slfm)) vs homecare and multifactorial-action (hmcr & mfa-) comparison. Downgrade twice.

Table 99 - Results of Depression: medium-term homecare network

**hmcr & ntr**						-0.24 (-0.62,0.14)
0.14 (-0.33,0.61)	**hmcr & mfar(w/med)**					-0.38 (-0.66,-0.10)
-0.34 (-0.97,0.29)	-0.48 (-1.06,0.10)	**hmcr & mfar**	0.19 (-0.06,0.44)			
-0.15 (-0.73,0.43)	-0.29 (-0.81,0.23)	0.19 (-0.06,0.44)	**hmcr & mfa-**		-0.00 (-0.37,0.36)	
-0.18 (-0.82,0.46)	-0.32 (-0.90,0.27)	0.17 (-0.56,0.89)	-0.03 (-0.70,0.65)	**hmcr & hmnt & exrc**		-0.06 (-0.58,0.45)
-0.16 (-0.60,0.29)	-0.29 (-0.66,0.08)	0.19 (-0.26,0.63)	-0.00 (-0.37,0.36)	0.02 (-0.55,0.59)	**hmcr & ADL & mfar(w/slfm)**	-0.09 (-0.33,0.16)
-0.24 (-0.62,0.14)	-0.38 (-0.66,-0.10)	0.10 (-0.40,0.61)	-0.09 (-0.53,0.35)	-0.06 (-0.58,0.45)	-0.09 (-0.33,0.16)	**hmcr**

Lower left triangle presents the findings (SMD with 95% CI) of the network meta-analysis. Upper right triangle presents the findings (SMD with 95% CI) of pairwise meta-analyses. A SMD>1 favours the upper left intervention; a SMD<1 favours the lower right intervention. Within the table, comparisons between treatments should be read from left to right (i.e. treatment 1 versus treatment 2). The estimate effect measure (SMD and their 95% CI) is in the cell in common between the row- and column-defining treatment.

Table 100 - Intervention rankings for Depression: medium-term homecare network

Treatment	SUCRA	Pr(Best)	Mean Rank	LCI Rank	UCI Rank
hmcr & mfar(w/med)	89.1	58.8	1.7	1	4
hmcr & ntr	69.7	22.3	2.8	1	7
hmcr & mfa-	50.0	7.2	4	1	6
hmcr & ADL & mfar(w/slfm)	49.4	1.3	4	2	7
hmcr & hmnt & exrc	43.9	9.9	4.4	1	7
hmcr	30.2	0	5.2	3	7
hmcr & mfar	17.8	0.5	5.9	2	7

SUCRA values (0–100) and mean ranks are presented, based on 1000 simulations. Higher SUCRAs and lower mean ranks indicate better performing interventions. Pr(Best) gives the probability of each specific intervention being ranked best intervention, based on 1000 simulations.

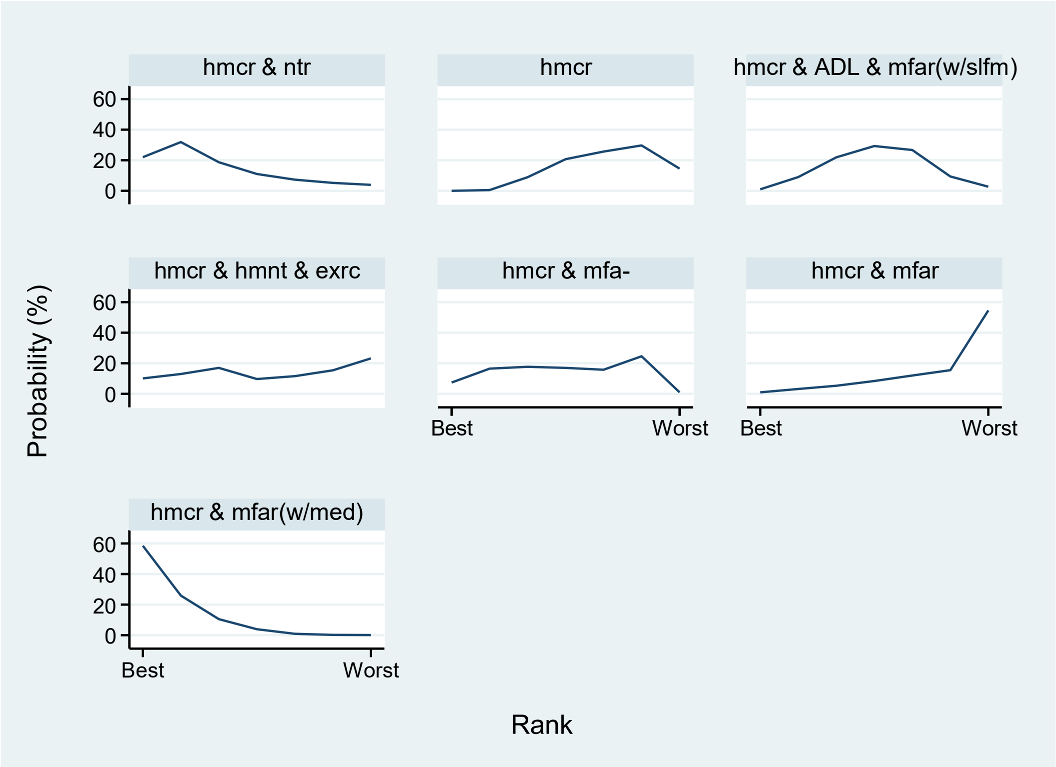

Figure 46 - Rankogram showing comparative effectiveness of interventions for depression medium-term homecare network. Results based on a simulation of 1000 replications.

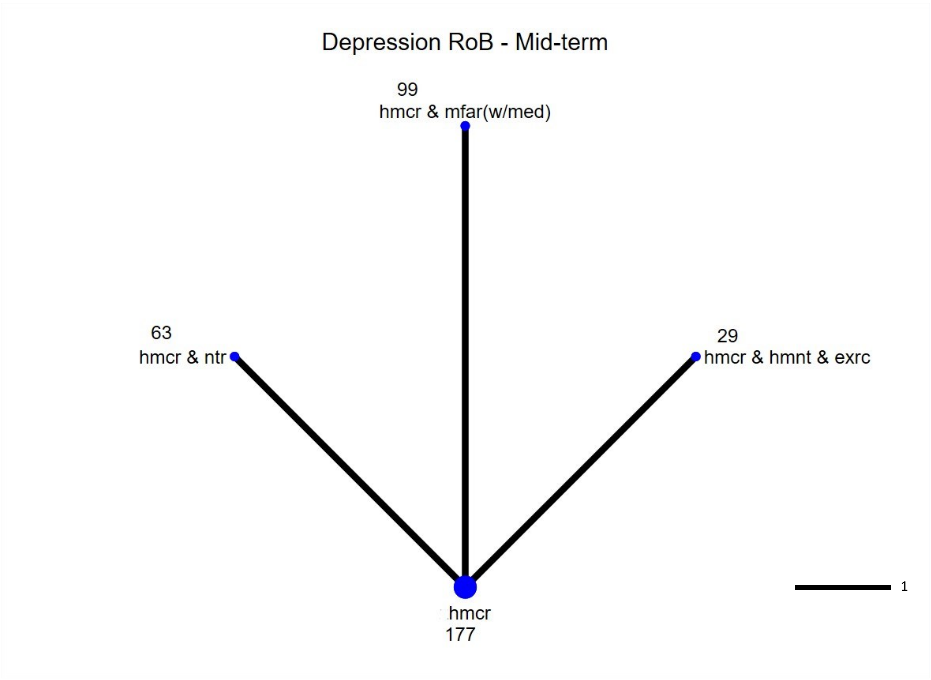

Figure 47 - Network plot for risk of bias analysis for depression medium-term homecare network

Table 101 - Intervention rankings for risk of bias analysis for depression: medium-term homecare network

Treatment	SUCRA	PrBest	Mean Rank	95% CI for true rank
hmcr & mfar(w/med)	86.1	64.2	1.4	1 - 3
hmcr & ntr	62.1	24.2	2.1	1 - 4
hmcr & hmnt & exrc	34.6	11.6	3	1 - 4
hmcr	17.2	0	3.5	2 - 4

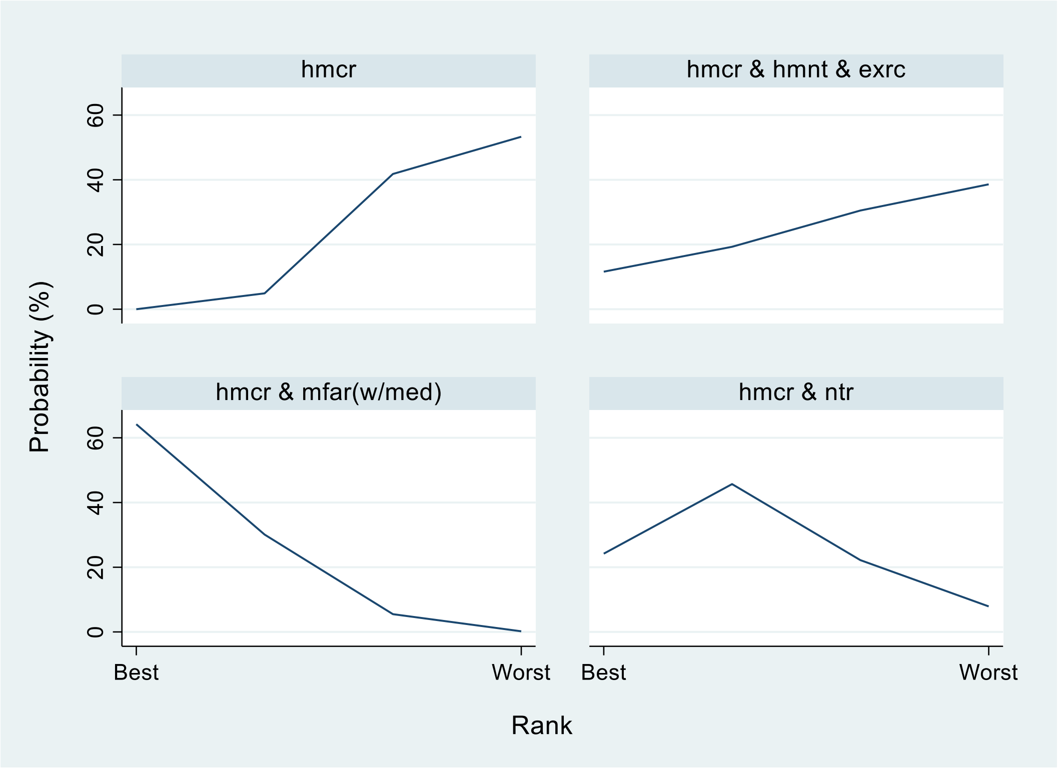

Figure 48 - Rankogram showing comparative effectiveness of interventions for risk of bias sensitivity analysis for depression medium-term homecare network. Results based on a simulation of 1000 replications.

Table 102 - Results of risk of bias depression: medium-term homecare network

**hmcr**	0.24 (-0.14,0.62)	0.38 (0.10,0.66)	0.06 (-0.45,0.58)
0.24 (-0.14,0.62)	**hmcr & ntr**		
0.38 (0.10,0.66)	0.14 (-0.33,0.61)	**hmcr & mfar(w/med)**	
0.06 (-0.45,0.58)	-0.18 (-0.82,0.46)	-0.32 (-0.90,0.27)	**hmcr & hmnt & exrc**

11.9 Mortality (medium-term timeframe only)

Table 103 - Medium-term mortality

Study	Frailty	n	Experimental group	Control group	ROB D1	D2	D3	D4	D5	Overall
**Alegria 2019**[503]	pre-frail	267	exrc & psyc	ac	x	x	x	+	-	xx
**Auvinen 2020**[505]	frail	494	hmcr & med	hmcr	+	-	x	+	+	x
**Barenfeld 2018**[507]	all	125	educ	ac	-	x	x	+	-	xx
**Bernabei 1998**[508]	frail	199	hmcr & mfar(w/med)	hmcr	-	-	+	+	+	-
**Bleijenberg 2016**[509]	pre-frail and frail	3092	rsk-mfa-	ac	x/+	-	+	+	-	x
**Blom 2016**[510]	all	1095	mfa-(w/med+slfm)	ac	x/+	-	x	+	-	xx
**Borrows 2013**[511]	unclassifiable	33	aids	mfa-	+	-	x	+	-	x
**Bouman 2008**[513]	pre-frail and frail	311	mfar(w/med)	ac	+	-	x	+	+	x
**Brettschneider 2015**[514]	frail	278	mfar(w/med)	ac	-	-	x	+	+	x
**Cameron 2013**[515]	frail	238	exrc & mfar(w/med+slfm)	ac	+	-	-	+	+	-
**Coleman 1999**[521]	frail	164	educ & mfar(w/med+slfm)	ac	-/-	-	x	+	-	x
**Counsell 2007**[522]	unclassifiable	853	educ & mfar(w/med+slfm)	ac	+/-	-	x	+	+	x
**Dalby 2000**[524]	frail	139	mfar(w/med)	ac	-	-	+	+	+	-
**de Craen 2006**[525]	all	335	mfa-	ac	+	-	x	+	-	x
**Dorresteijn 2016**[526]	unclassifiable	389	ADL	ac	+	-	+	+	-	-
**Fabacher 1994**[528]	all	229	mfar(w/med)	ac	-	-	x	+	+	x
**Fernandez-Barres 2017**[531]	frail	147	hmcr & ntr	hmcr	+	-	x	+	-	x
**Fristedt 2019**[535]	frail	62	hmcr & mfar(w/med)	hmcr	x	-	+	+	+	x
**Gill 2002**[537]	pre-frail and frail	188	ADL & exrc	ac	-	-	+	+	+	-
**Gitlin 2006**[539]	pre-frail and frail	319	ADL & aids & exrc	ac	+	-	+	+	+	-
**Gustafsson 2013**[542]	all	288	educ & mfa-	ac	-	-	x	+	-	x
**Hall 1992**[543]	frail	167	hmcr & mfar(w/slfm)	hmcr & mfar	-	-	+	+	+	-
**Harari 2008**[544]	all	2423	mfar(w/med)	ac	+	x	x	+	+	xx
**Hay 1998**[546]	unclassifiable	484	mfa-	ac	-	-	x	+	-	x
**Hebert 2001**[547]	pre-frail and frail	494	mfar(w/med)	ac	-	-	-	+	-	-
**Henderson 2005**[548]	robust	130	mfar	ac	+/x	+	x	+	-	xx
**Hendriksen 1984**[549]	all	572	mfar	ac	-	-	+	+	+	-
**Hogg 2009**[550]	unclassifiable	240	mfar(w/med)	ac	-	-	-	+	-	-
**Holland 2005**[551]	unclassifiable	493	educ & exrc & mfar(w/slfm)	ac	+	-	x	+	-	x
**Howel 2019**[552]	all	725	wlfr	ac	+	+	x	+	+	x
**Kerse 2014**[556]	pre-frail and frail	3687	rsk-mfa-	ac	+/+	-	x	+	-	x
**Kono 2004**[559]	pre-frail and frail	117	mfar	ac	-	-	-	+	-	-
**Kono 2016**[558]	pre-frail	351	mfar(w/med)	mfar	+	-	x	+	+	x
**Kukkonen-Harjula 2017**[561]	pre-frail and frail	287	ADL & ntr & exrc	ac	+	-	x	+	-	x
**Leveille 1998**[564]	unclassifiable	191	educ & exrc & mfar(w/med+slfm)	ac	+	-	x	+	-	x
**Lewin 2013**[565]	frail	750	hmcr & educ & mfar	hmcr	x	x	+	+	+	xx
**Liimatta 2019**[567]	robust and pre-frail	422	exrc & mfa-(w/med)	ac	-	-	+	+	+	-
**Mann WC 1999**[571]	frail	100	hmcr & aids	hmcr	-	-	-	+	-	-
**Meng 2005**[574]	frail	599	vchr	ac	-	x	x	+	x	xx
**Metzelthin 2013**[576]	frail	321	educ & mfar(w/med+slfm)	ac	-/-	-	x	+	-	x
**Monteserin Nadal 2008**[578]	all	516	educ & rsk-mfa-	ac	-	-	x	+	+	x
**Morey 2009**[580]	unclassifiable	362	exrc	ac	-	-	x	+	-	x
**Newbury 2001**[582]	unclassifiable	100	mfa-(w/med)	ac	-	-	+	+	+	-
**Newcomer 2004**[583]	unclassifiable	2934	educ & mfar(w/med)	ac	-	-	x	+	-	x
**Ng 2015**[584]	pre-frail and frail	95	cgn & ntr & exrc	ac	+	-	x	+	+	x
**Parsons M 2012**[587]	frail	196	hmcr & mfar	hmcr & mfa-	+/-	-	x	+	-	x
**Parsons M 2017**[586]	frail	75	hmcr & ADL & mfar(w/slfm)	hmcr & mfa-	-	-	x	+	+	x
**Ploeg 2010**[590]	pre-frail and frail	713	educ & mfar(w/med)	ac	+	-	-	+	-	-
**Rockwood 2000**[592]	frail	182	mfa-(w/med)	ac	-	-	+	+	+	-
**Romera-Liebana 2018**[593]	pre-frail and frail	342	cgn & med & ntr & exrc	ac	+	-	x	+	-	x
**Rooijackers 2021**[594]	frail	252	hmcr & ADL & mfar(w/slfm)	hmcr	+/-	-	-	+	-	-
**Rubenstein 2007**[595]	frail	792	mfar(w/med)	ac	-	-	+	+	+	-

**Serra-Prat 2017[597]**	pre-frail	172	ntr & exrc	ac	-	-	+	+	-	-
**Shapiro 2002[598]**	frail	58	hmcr & mfar	ac	-	x	x	+	-	xx
**Siemonsma 2018[600]**	frail	118	ADL	mfa-	-	-	x	+	-	x
**Suijker 2016[605]**	frail	2283	mfar(w/med)	ac	+/-	-	+	+	+	-
**Szanton 2019[607]**	pre-frail and frail	273	ADL&aids&educ&exrc& mfar(w/med+slfm)	ac	+	-	x	+	-	x
**Takahashi 2012[608]**	frail	186	mntr-mfa-	ac	-	-	x	+	-	x
**Teut 2013[609]**	frail	55	hmcr & hmnt & exrc	hmcr	+/+	-	-	+	-	-
**van Hout 2010[618]**	frail	651	mfar(w/med)	ac	+	-	+	+	+	-
**van Rossum 1993[621]**	all	580	mfar	ac	-	-	+	+	+	-
**Vass 2005[622]**	all	4060	mfar(w/med)	mfar	+/+	-	+	+	+	-
**Williams 1992[628]**	all	470	mfar	mfa-	-	-	+	+	+	-
**Wolter 2013[629]**	frail	732	hmcr & mfar(w/med)	hmcr	+/-	-	x	+	-	x
**Yamada 2003[631]**	pre-frail and frail	356	mfar(w/med)	ac	+	-	x	+	-	x

n: number of participants. ROB: risk of bias. D#: Domain #. D1: risk of bias arising from the randomisation process (individual); or, for cluster trials, risk of bias arising from the randomisation process / risk of bias arising from the identification or recruitment of participants into clusters. D2: risk of bias due to deviations from the intended interventions (effect of assignment to the intervention). D3: risk of bias due to missing outcome data. D4: risk of bias in measurement of the outcome. D5: risk of bias in selection of the reported result. +: low risk of bias; -: some concerns; x: high risk of bias / serious concerns; xx: very serious concerns (overall risk of bias only). all: robust, pre-frail and frail.

Table 104 - Mortality in the medium term: comparisons with available care summary of findings table

Population: Older people

Interventions: Community-based complex interventions

Comparator: Available care (ac)

Outcome: Mortality

Timeframe: medium term; range of follow up 12 to 18 months

Setting: Community

Total studies: 65

Total participants: 38,351

Comparator rank: Mean 26.0, 95% CI 18 to 31

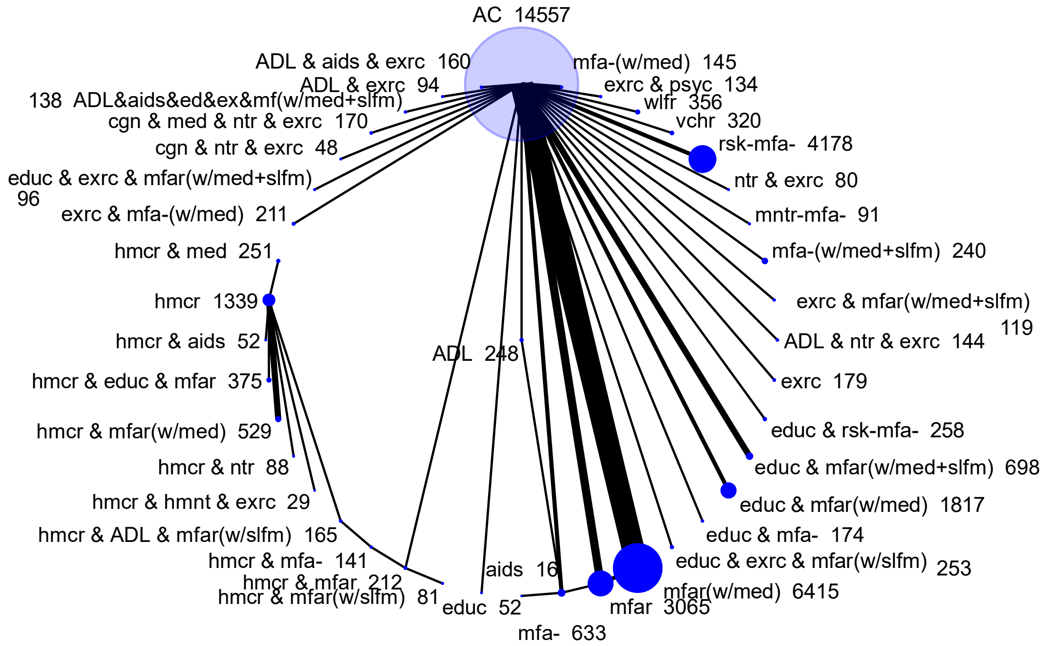

Intervention group	Relative effect (95% CI)		Anticipated absolute effect (95% CI)				Certainty of the evidence (GRADE)	Ranking (95% CI)	Interpretation
			High-risk population (202 per 1000 with ac)		Low-risk population (7 per 1000 with ac)				
	Network estimate	Calculated risk ratio[a]	With intervention	Difference	With intervention	Difference			
ADL, aids and exercise (ADL & aids & exrc)	OR 0.16 (0.03 to 0.71) Mixed estimate	RR 0.17 (0.03 to 0.72)	39 per 1000 (8 to 152)	163 fewer per 1000 (194 fewer to 50 fewer)	1 per 1000 (0 to 5)	6 fewer per 1000 (7 fewer to 2 fewer)	⊕⊕⊖⊖ Low[b]	8.6 (1 to 19)	may result in a reduction in mortality
Multifactorial-action and review (mfar)	OR 0.88 (0.66 to 1.18) Mixed estimate	RR 0.88 (0.67 to 1.17)	182 per 1000 (143 to 230)	20 fewer per 1000 (59 fewer to 28 more)	7 per 1000 (5 to 9)	1 fewer per 1000 (3 fewer to 1 more)	⊕⊕⊖⊖ Low[c]	22.9 (12 to 31)	may result in a slight reduction in mortality
ADL (ADL)	OR 1.03 (0.44 to 2.43) Mixed estimate	RR 1.03 (0.45 to 2.29)	206 per 1000 (100 to 380)	5 more per 1000 (102 fewer to 179 more)	8 per 1000 (3 to 18)	0 per 1000 (4 fewer to 10 more)	⊕⊕⊖⊖ Low[c]	25.9 (12 to 37)	may result in a very slight increase in mortality
Exercise, multifactorial-action and review with medication-review and self-management strategies (exrc & mfar(w/med+slfm))	OR 1.22 (0.50 to 3.01) Mixed estimate	RR 1.21 (0.51 to 2.77)	235 per 1000 (112 to 432)	34 more per 1000 (89 fewer to 230 more)	9 per 1000 (4 to 22)	2 more per 1000 (4 fewer to 15 more)	⊕⊕⊖⊖ Low[c]	28.6 (11 to 38)	may result in a slight increase in mortality
Multifactorial-action with medication-review (mfa-(w/med))	OR 1.23 (0.50 to 3.04) Mixed estimate	RR 1.22 (0.51 to 2.80)	237 per 1000 (112 to 434)	35 more per 1000 (89 fewer to 233 more)	9 per 1000 (4 to 22)	2 more per 1000 (4 fewer to 15 more)	⊕⊕⊖⊖ Low[c]	28.8 (14 to 38)	may result in a slight increase in mortality

Exercise and multifactorial-action with medication-review (exrc & mfa-(w/med))	OR 1.51 (0.25 to 9.20) Mixed estimate	RR 1.48 (0.26 to 6.83)	276 per 1000 (59 to 699)	74 more per 1000 (142 fewer to 497 more)	11 per 1000 (2 to 64)	4 more per 1000 (6 fewer to 57 more)	⊕⊕⊖⊖ Low[c]	29.7 (8 to 41)	may result in an increase in mortality
ADL and exercise (ADL & exrc)	OR 1.53 (0.41 to 5.70) Mixed estimate	RR 1.50 (0.42 to 4.75)	279 per 1000 (94 to 590)	77 more per 1000 (108 fewer to 388 more)	11 per 1000 (3 to 41)	4 more per 1000 (4 fewer to 33 more)	⊕⊕⊖⊖ Low[c]	30.9 (11 to 40)	may result in an increase in mortality
Homecare and aids (hmcr & aids)	OR 0.07 (0.00 to 1.57) Indirect estimate	RR 0.07 (0.00 to 1.53)	17 per 1000 (0 to 284)	184 fewer per 1000 (202 fewer to 82 more)	1 per 1000 (0 to 12)	7 fewer per 1000 (7 fewer to 4 more)	⊕⊖⊖⊖ Very Low[d,e]	5.1 (1 to 27)	the evidence is very uncertain about the effect on mortality
Homecare, multifactorial-action and review with medication-review (hmcr & mfar(w/med))	OR 0.10 (0.01 to 1.64) Indirect estimate	RR 0.10 (0.01 to 1.60)	25 per 1000 (3 to 293)	177 fewer per 1000 (199 fewer to 91 more)	1 per 1000 (0 to 12)	7 fewer per 1000 (7 fewer to 5 more)	⊕⊖⊖⊖ Very low[d,e]	6.0 (1 to 27)	the evidence is very uncertain about the effect on mortality
Homecare, education, multifactorial-action and review (hmcr & educ & mfar)	OR 0.12 (0.01 to 1.93) Indirect estimate	RR 0.12 (0.01 to 1.86)	29 per 1000 (3 to 328)	172 fewer per 1000 (199 fewer to 126 more)	1 per 1000 (0 to 14)	7 fewer per 1000 (7 fewer to 7 more)	⊕⊖⊖⊖ Very low[d,e]	7.7 (1 to 30)	the evidence is very uncertain about the effect on mortality
Homecare (hmcr)	OR 0.13 (0.01 to 1.97) Indirect estimate	RR 0.13 (0.01 to 1.89)	32 per 1000 (3 to 332)	170 fewer per 1000 (199 fewer to 131 more)	1 per 1000 (0 to 14)	6 fewer per 1000 (7 fewer to 7 more)	⊕⊖⊖⊖ Very low[d,e]	8.1 (2 to 29)	the evidence is very uncertain about the effect on mortality
Exercise (exrc)	OR 0.17 (0.02 to 1.40) Mixed estimate	RR 0.18 (0.02 to 1.38)	41 per 1000 (5 to 261)	160 fewer per 1000 (197 fewer to 60 more)	1 per 1000 (0 to 10)	6 fewer per 1000 (7 fewer to 3 more)	⊕⊖⊖⊖ Very low[d,e]	9.6 (1 to 34)	the evidence is very uncertain about the effect on mortality
Homecare, multifactorial-action and review (hmcr & mfar)	OR 0.16 (0.02 to 1.58) Mixed estimate	RR 0.17 (0.02 to 1.54)	39 per 1000 (5 to 285)	163 fewer per 1000 (197 fewer to 84 more)	1 per 1000 (0 to 12)	6 fewer per 1000 (7 fewer to 4 more)	⊕⊖⊖⊖ Very low[g,h]	9.6 (2 to 28)	the evidence is very uncertain about the effect on mortality
Homecare and medication-review (hmcr & med)	OR 0.16 (0.01 to 2.68) Indirect estimate	RR 0.17 (0.01 to 2.50)	39 per 1000 (3 to 404)	163 fewer per 1000 (199 fewer to 202 more)	1 per 1000 (0 to 20)	6 fewer per 1000 (7 fewer to 12 more)	⊕⊖⊖⊖ Very low[d,e]	10.8 (2 to 34)	the evidence is very uncertain about the effect on mortality
Homecare, multifactorial-action and review with self-management strategies (hmcr & mfar(w/slfm))	OR 0.18 (0.01 to 2.26) Indirect estimate	RR 0.19 (0.01 to 2.15)	43 per 1000 (3 to 363)	158 fewer per 1000 (199 fewer to 162 more)	1 per 1000 (0 to 17)	6 fewer per 1000 (7 fewer to 9 more)	⊕⊖⊖⊖ Very low[d,e]	10.8 (1 to 34)	the evidence is very uncertain about the effect on mortality
Nutrition and exercise (ntr & exrc)	OR 0.22 (0.01 to 4.78) Mixed estimate	RR 0.23 (0.01 to 4.12)	53 per 1000 (3 to 547)	149 fewer per 1000 (199 fewer to 345 more)	2 per 1000 (0 to 34)	6 fewer per 1000 (7 fewer to 27 more)	⊕⊖⊖⊖ Very low[i]	13.0 (1 to 39)	the evidence is very uncertain about the effect on mortality
Homecare, ADL, multifactorial-action and review with self-management strategies (hmcr & ADL & mfar(w/slfm))	OR 0.22 (0.02 to 3.01) Indirect estimate	RR 0.23 (0.02 to 2.77)	53 per 1000 (5 to 432)	149 fewer per 1000 (197 fewer to 230 more)	2 per 1000 (0 to 22)	6 fewer per 1000 (7 fewer to 15 more)	⊕⊖⊖⊖ Very low[d,e]	13.1 (5 to 35)	the evidence is very uncertain about the effect on mortality
Cognitive training, nutrition and exercise (cgn & ntr & exrc)	OR 0.32 (0.01 to 8.09) Mixed estimate	RR 0.33 (0.01 to 6.22)	75 per 1000 (3 to 671)	127 fewer per 1000 (199 fewer to 470 more)	2 per 1000 (0 to 57)	5 fewer per 1000 (7 fewer to 50 more)	⊕⊖⊖⊖ Very low[c,f]	15.6 (1 to 40)	the evidence is very uncertain about the effect on mortality
Homecare and multifactorial-action (hmcr & mfa-)	OR 0.31 (0.03 to 3.46) Indirect estimate	RR 0.32 (0.03 to 3.13)	73 per 1000 (8 to 466)	129 fewer per 1000 (194 fewer to 265 more)	2 per 1000 (0 to 25)	5 fewer per 1000 (7 fewer to 18 more)	⊕⊖⊖⊖ Very low[g,h]	15.9 (7 to 37)	the evidence is very uncertain about the effect on mortality
ADL, nutrition and exercise (ADL & ntr & exrc)	OR 0.48 (0.16 to 1.46) Mixed estimate	RR 0.49 (0.17 to 1.43)	108 per 1000 (39 to 269)	93 fewer per 1000 (163 fewer to 68 more)	4 per 1000 (1 to 11)	4 fewer per 1000 (6 fewer to 3 more)	⊕⊖⊖⊖ Very low[c,f]	16.1 (4 to 33)	the evidence is very uncertain about the effect on mortality
Cognitive training, medication-review, nutrition and exercise (cgn & med & ntr & exrc)	OR 0.49 (0.18 to 1.35) Mixed estimate	RR 0.50 (0.19 to 1.33)	110 per 1000 (43 to 254)	91 fewer per 1000 (158 fewer to 53 more)	4 per 1000 (1 to 10)	4 fewer per 1000 (6 fewer to 3 more)	⊕⊖⊖⊖ Very low[c,f]	16.3 (4 to 32)	the evidence is very uncertain about the effect on mortality
Homecare, alternative-medicine and exercise (hmcr & hmnt & exrc)	OR 0.31 (0.01 to 7.00) Indirect estimate	RR 0.32 (0.01 to 5.58)	73 per 1000 (3 to 639)	129 fewer per 1000 (199 fewer to 437 more)	2 per 1000 (0 to 50)	5 fewer per 1000 (7 fewer to 42 more)	⊕⊖⊖⊖ Very low[d,e]	16.8 (2 to 40)	the evidence is very uncertain about the effect on mortality

Education, exercise, multifactorial-action and review with medication-review and self-management strategies (educ & exrc & mfar(w/med+slfm))	OR 0.49<br>(0.04 to 5.53)<br>Mixed estimate	RR 0.50<br>(0.04 to 4.64)	110 per 1000<br>(10 to 583)	91 fewer per 1000<br>(192 fewer to 381 more)	4 per 1000<br>(0 to 40)	4 fewer per 1000<br>(7 fewer to 32 more)	⊕⊖⊖⊖<br>Very low[e,f]	17.9<br>(1 to 40)	the evidence is very uncertain about the effect on mortality
Homecare and nutrition (hmcr & ntr)	OR 0.35<br>(0.02 to 6.49)<br>Indirect estimate	RR 0.36<br>(0.02 to 5.27)	81 per 1000<br>(5 to 621)	120 fewer per 1000<br>(197 fewer to 419 more)	3 per 1000<br>(0 to 46)	5 fewer per 1000<br>(7 fewer to 39 more)	⊕⊖⊖⊖<br>Very low[d,e]	17.9<br>(6 to 39)	the evidence is very uncertain about the effect on mortality
Multifactorial-action and review with medication-review (mfar(w/med))	OR 0.86<br>(0.71 to 1.05)<br>Mixed estimate	RR 0.87<br>(0.72 to 1.05)	178 per 1000<br>(152 to 210)	23 fewer per 1000<br>(50 fewer to 8 more)	6 per 1000<br>(5 to 8)	1 fewer per 1000<br>(2 fewer to 0)	⊕⊖⊖⊖<br>Very low[c,j]	22.1<br>(13 to 29)	the evidence is very uncertain about the effect on mortality
Welfare-advice (wlfr)	OR 0.80<br>(0.29 to 2.22)<br>Mixed estimate	RR 0.81<br>(0.30 to 2.11)	168 per 1000<br>(68 to 359)	34 fewer per 1000<br>(133 fewer to 158 more)	6 per 1000<br>(2 to 16)	1 fewer per 1000<br>(5 fewer to 9 more)	⊕⊖⊖⊖<br>Very low[c,f]	22.7<br>(7 to 37)	the evidence is very uncertain about the effect on mortality
Multifactorial-action (mfa-)	OR 0.89<br>(0.56 to 1.43)<br>Mixed estimate	RR 0.89<br>(0.57 to 1.40)	183 per 1000<br>(124 to 265)	18 fewer per 1000<br>(78 fewer to 64 more)	7 per 1000<br>(4 to 11)	1 fewer per 1000<br>(3 fewer to 3 more)	⊕⊖⊖⊖<br>Very low[c,f]	23.2<br>(11 to 34)	the evidence is very uncertain about the effect on mortality
Aids (aids)	OR 0.95<br>(0.05 to 17.30)<br>Indirect estimate	RR 0.95<br>(0.05 to 10.24)	193 per 1000<br>(12 to 814)	8 fewer per 1000<br>(189 fewer to 612 more)	7 per 1000<br>(0 to 114)	0 per 1000<br>(7 fewer to 107 more)	⊕⊖⊖⊖<br>Very low[e,k]	24.6<br>(2 to 41)	the evidence is very uncertain about the effect on mortality
Multifactorial-action with medication-review and self-management strategies (mfa-(w/med+slfm))	OR 1.00<br>(0.59 to 1.68)<br>Mixed estimate	RR 1.00<br>(0.60 to 1.63)	202 per 1000<br>(130 to 298)	0 per 1000<br>(72 fewer to 96 more)	7 per 1000<br>(4 to 12)	0 per 1000<br>(3 fewer to 5 more)	⊕⊖⊖⊖<br>Very low[l,m]	25.5<br>(14 to 35)	the evidence is very uncertain about the effect on mortality
Education and risk-screening (educ & rsk-mfa)	OR 1.00<br>(0.52 to 1.93)<br>Mixed estimate	RR 1.00<br>(0.53 to 1.86)	202 per 1000<br>(116 to 328)	0 per 1000<br>(86 fewer to 126 more)	7 per 1000<br>(4 to 14)	0 per 1000<br>(4 fewer to 7 more)	⊕⊖⊖⊖<br>Very low[c,f]	25.9<br>(11 to 36)	the evidence is very uncertain about the effect on mortality
Care voucher (vchr)	OR 1.02<br>(0.59 to 1.79)<br>Mixed estimate	RR 1.02<br>(0.60 to 1.73)	205 per 1000<br>(130 to 311)	3 more per 1000<br>(72 fewer to 110 more)	8 per 1000<br>(4 to 13)	0 per 1000<br>(3 fewer to 6 more)	⊕⊖⊖⊖<br>Very low[m,n]	25.9<br>(14 to 36)	the evidence is very uncertain about the effect on mortality
Risk-screening (rsk-mfa-)	OR 1.03<br>(0.76 to 1.37)<br>Mixed estimate	RR 1.03<br>(0.77 to 1.35)	206 per 1000<br>(161 to 257)	5 more per 1000<br>(41 fewer to 55 more)	8 per 1000<br>(6 to 10)	0 per 1000<br>(2 fewer to 3 more)	⊕⊖⊖⊖<br>Very low[c,o]	26.4<br>(16 to 34)	the evidence is very uncertain about the effect on mortality
Education (educ)	OR 1.41<br>(0.09 to 23.20)<br>Mixed estimate	RR 1.39<br>(0.09 to 11.96)	262 per 1000<br>(22 to 854)	61 more per 1000<br>(179 fewer to 653 more)	10 per 1000<br>(1 to 148)	3 more per 1000<br>(7 fewer to 140 more)	⊕⊖⊖⊖<br>Very low[h,p]	27.2<br>(3 to 41)	the evidence is very uncertain about the effect on mortality
Education, exercise, multifactorial-action and review with self-management strategies (educ & exrc & mfar(w/slfm))	OR 1.19<br>(0.31 to 4.54)<br>Mixed estimate	RR 1.18<br>(0.32 to 3.95)	231 per 1000<br>(73 to 534)	29 more per 1000<br>(129 fewer to 332 more)	9 per 1000<br>(2 to 33)	1 more per 1000<br>(5 fewer to 25 more)	⊕⊖⊖⊖<br>Very low[c,f]	27.8<br>(8 to 39)	the evidence is very uncertain about the effect on mortality
Education, multifactorial-action and review with medication-review (educ & mfar(w/med))	OR 1.10<br>(0.73 to 1.67)<br>Mixed estimate	RR 1.10<br>(0.74 to 1.62)	217 per 1000<br>(156 to 297)	16 more per 1000<br>(46 fewer to 95 more)	8 per 1000<br>(5 to 12)	1 more per 1000<br>(2 fewer to 5 more)	⊕⊖⊖⊖<br>Very low[c,f]	27.8<br>(17 to 36)	the evidence is very uncertain about the effect on mortality
Education and multifactorial-action (educ & mfa-)	OR 1.32<br>(0.23 to 7.39)<br>Mixed estimate	RR 1.30<br>(0.24 to 5.82)	250 per 1000<br>(55 to 651)	48 more per 1000<br>(147 fewer to 449 more)	10 per 1000<br>(2 to 52)	2 more per 1000<br>(6 fewer to 45 more)	⊕⊖⊖⊖<br>Very low[c,f]	28.4<br>(7 to 40)	the evidence is very uncertain about the effect on mortality
Education, multifactorial-action and review with medication-review and self-management strategies (educ & mfar(w/med+slfm))	OR 1.15<br>(0.66 to 2.01)<br>Mixed estimate	RR 1.14<br>(0.67 to 1.93)	225 per 1000<br>(143 to 337)	23 more per 1000<br>(59 fewer to 135 more)	9 per 1000<br>(5 to 15)	1 more per 1000<br>(3 fewer to 7 more)	⊕⊖⊖⊖<br>Very low[c,f]	28.4<br>(16 to 36)	the evidence is very uncertain about the effect on mortality
Exercise and psychology (exrc & psyc)	OR 4.06<br>(0.44 to 37.10)<br>Mixed estimate	RR 3.59<br>(0.45 to 14.68)	506 per 1000<br>(100 to 904)	305 more per 1000<br>(102 fewer to 702 more)	29 per 1000<br>(3 to 217)	22 more per 1000<br>(4 fewer to 209 more)	⊕⊖⊖⊖<br>Very low[m,q]	35.9<br>(15 to 41)	the evidence is very uncertain about the effect on mortality
Monitoring (mntr-mfa-)	OR 4.49<br>(1.41 to 14.30)<br>Mixed estimate	RR 3.91<br>(1.39 to 9.15)	531 per 1000<br>(262 to 783)	330 more per 1000<br>(61 more to 582 more)	32 per 1000<br>(10 to 96)	25 more per 1000<br>(3 more to 89 more)	⊕⊖⊖⊖<br>Very low[c,f]	38.5<br>(32 to 41)	the evidence is very uncertain about the effect on mortality

ADL, aids, education, exercise, multifactorial-action and review with medication-review and self-management strategies (ADL & aids & ed & ex & mf(w/med+slfm))	OR 8.25 (1.01 to 67.40) Mixed estimate	RR 6.31 (1.01 to 17.69)	676 per 1000 (203 to 944)	474 more per 1000 (2 more to 743 more)	58 per 1000 (7 to 335)	51 more per 1000 (0 to 327 more)	⊕⊖⊖⊖ Very low[e,f]	38.9 (23 to 41)	the evidence is very uncertain about the effect on mortality

a: Calculated from OR and an assumed comparator risk of 0.042, the median available care risk among these studies.

b: very serious concerns about imprecision as confidence interval is wide and direct evidence is based on 14 events from 319 persons. Downgrade twice.

c: very serious concerns about imprecision as confidence interval includes substantial benefit and substantial harm. Downgrade twice.

d: serious concerns about risk of bias due to excluding participants in per-protocol analysis and missing outcome data in indirect evidence. Downgrade once.

e: extremely serious concerns about imprecision as confidence interval is extremely wide. Downgrade twice (would be three downgrades except for additional downgrades for risk of bias).

f: serious concerns about risk of bias due to missing outcome data. Downgrade once.

g: very serious concerns about risk of bias due to excluding participants in per-protocol analysis and missing outcome data. Downgrade twice.

h: extremely serious concerns about imprecision as confidence interval is extremely wide. Downgrade once (would be three downgrades except for additional downgrades for risk of bias).

i: extremely serious concerns about imprecision as confidence interval is extremely wide and direct evidence is based on 2 events from 172 persons. Downgrade three levels.

j: serious concerns about inconsistency (heterogeneity) between studies as confidence intervals do not overlap. Downgrade once.

k: serious concerns about risk of bias due to missing outcome data in indirect evidence. Downgrade once.

l: very serious concerns about risk of bias due to randomisation process and missing outcome data. Downgrade twice.

m: very serious concerns about imprecision as confidence interval includes substantial benefit and substantial harm. Already downgraded twice for risk of bias, downgrade once.

n: very serious concerns about risk of bias due to excluding participants from analyses, missing data, and selective reporting results. Downgrade twice.

o: serious concerns about risk of bias due to randomisation process and missing outcome data in each of the two studies respectively. Downgrade once.

p: very serious concerns about risk of bias due to contamination between the intervention arms and missing outcome data. Downgrade twice.

q: very serious concerns about risk of bias due to randomisation process, excluding participants in per-protocol analysis, and missing outcome data. Downgrade twice.

Table 105 - Results of mortality: medium-term network

Please note: the results of mortality: medium-term network are too large to fit on one page. Please see https://doi.org/10.5518/1377

**wlfr**																																								0.80 (0.30, 2.18)
0.78 (0.25, 2.49)	**vchr**																																							1.02 (0.61, 1.73)
0.78 (0.27, 2.25)	1.00 (0.53, 1.87)	**rsk-mfa-**																																						1.03 (0.79, 1.33)
3.57 (0.14, 89.42)	4.56 (0.20, 101.89)	4.56 (0.21, 98.30)	**ntr & exrc**																																					0.22 (0.01, 4.75)
0.18 (0.04, 0.83)	0.23 (0.06, 0.83)	0.23 (0.07, 0.75)	0.05 (0.00, 1.32)	**mntr-mfa-**																																				4.49 (1.43, 14.10)
0.93 (0.33, 2.62)	1.19 (0.66, 2.15)	1.19 (0.83, 1.70)	0.26 (0.01, 5.58)	5.21 (1.61, 16.89)	**mfar(w/med)**	0.99 (0.73, 1.36)																																		0.88 (0.66, 1.17)
0.91 (0.32, 2.63)	1.17 (0.62, 2.19)	1.17 (0.77, 1.77)	0.26 (0.01, 5.52)	5.11 (1.55, 16.89)	0.98 (0.74, 1.30)	**mfar**			0.74 (0.44, 1.24)																															1.07 (0.70, 1.64)
0.80 (0.26, 2.52)	1.03 (0.48, 2.20)	1.03 (0.56, 1.87)	0.23 (0.01, 5.00)	4.49 (1.26, 16.02)	0.86 (0.49, 1.51)	0.88 (0.48, 1.60)	**mfa-(w/med+slfm)**																																	1.00 (0.61, 1.63)
0.65 (0.17, 2.54)	0.83 (0.29, 2.40)	0.83 (0.32, 2.15)	0.18 (0.01, 4.42)	3.64 (0.84, 15.85)	0.70 (0.28, 1.77)	0.71 (0.27, 1.85)	0.81 (0.29, 2.30)	**mfa-(w/med)**																																0.72 (0.08, 6.49)
0.90 (0.29, 2.76)	1.15 (0.56, 2.38)	1.15 (0.66, 1.99)	0.25 (0.01, 5.56)	5.04 (1.44, 17.60)	0.97 (0.59, 1.57)	0.99 (0.62, 1.57)	1.12 (0.56, 2.26)	1.38 (0.50, 3.80)	**mfa-**																									0.94 (0.05, 16.37)					0.83 (0.23, 3.04)	0.53 (0.26, 1.11)
2.31 (0.10, 51.08)	2.95 (0.15, 57.93)	2.95 (0.16, 55.80)	0.65 (0.01, 44.50)	12.91 (0.55, 300.52)	2.48 (0.13, 46.55)	2.53 (0.13, 47.81)	2.87 (0.15, 56.12)	3.54 (0.17, 75.75)	2.56 (0.13, 49.63)	**hmcr & ntr**										2.77 (1.04, 7.35)																				
4.58 (0.29, 72.02)	5.86 (0.43, 80.42)	5.86 (0.45, 77.05)	1.28 (0.02, 69.26)	25.66 (1.54, 426.33)	4.93 (0.38, 64.21)	5.02 (0.38, 66.03)	5.71 (0.42, 77.84)	7.04 (0.47, 106.34)	5.09 (0.38, 68.76)	1.99 (0.22, 17.94)	**hmcr & mfar(w/slfm)**		1.07 (0.33, 3.45)																											
7.89 (0.41, 152.04)	10.08 (0.59, 171.42)	10.08 (0.62, 164.91)	2.21 (0.04, 137.64)	44.17 (2.18, 896.57)	8.48 (0.52, 137.31)	8.64 (0.53, 140.93)	9.83 (0.58, 166.02)	12.12 (0.65, 225.78)	8.77 (0.52, 147.29)	3.42 (1.18, 9.91)	1.72 (0.23, 12.70)	**hmcr & mfar(w/med)**								0.80 (0.57, 1.11)																				
4.89 (0.41, 58.64)	6.25 (0.61, 64.47)	6.25 (0.64, 61.45)	1.37 (0.03, 61.62)	27.37 (2.14, 349.21)	5.25 (0.54, 51.14)	5.35 (0.54, 52.65)	6.09 (0.59, 62.35)	7.51 (0.65, 86.21)	5.43 (0.54, 55.01)	2.12 (0.33, 13.49)	1.07 (0.32, 3.50)	0.62 (0.12, 3.09)	**hmcr & mfar**	0.53 (0.23, 1.21)																										0.16 (0.02, 1.57)
2.60 (0.19, 35.92)	3.33 (0.28, 39.82)	3.33 (0.29, 38.06)	0.73 (0.01, 36.00)	14.58 (1.00, 213.19)	2.80 (0.25, 31.70)	2.85 (0.25, 32.62)	3.24 (0.27, 38.53)	4.00 (0.30, 52.92)	2.89 (0.25, 34.02)	1.13 (0.22, 5.86)	0.57 (0.13, 2.44)	0.33 (0.08, 1.29)	0.53 (0.23, 1.24)	**hmcr & mfa-**					1.40 (0.52, 3.77)																					
4.97 (0.25, 98.47)	6.34 (0.36, 111.17)	6.34 (0.38, 106.91)	1.39 (0.02, 88.42)	27.79 (1.33, 580.40)	5.34 (0.32, 89.16)	5.44 (0.32, 91.61)	6.18 (0.36, 107.67)	7.63 (0.40, 145.84)	5.52 (0.32, 95.18)	2.15 (0.69, 6.74)	1.08 (0.14, 8.34)	0.63 (0.32, 1.24)	1.02 (0.19, 5.33)	1.91 (0.46, 7.95)	**hmcr & med**					1.29 (0.76, 2.18)																				
2.62 (0.10, 70.15)	3.34 (0.14, 80.13)	3.34 (0.14, 77.37)	0.73 (0.01, 58.18)	14.65 (0.52, 411.56)	2.81 (0.12, 64.58)	2.87 (0.12, 66.30)	3.26 (0.14, 77.66)	4.02 (0.15, 104.25)	2.91 (0.12, 68.72)	1.13 (0.19, 6.78)	0.57 (0.05, 6.69)	0.33 (0.07, 1.53)	0.54 (0.06, 4.62)	1.00 (0.14, 7.29)	0.53 (0.11, 2.58)	**hmcr & hmnt & exrc**				2.44 (0.56, 10.65)																				
6.71 (0.35, 129.70)	8.57 (0.50, 146.26)	8.57 (0.52, 140.61)	1.88 (0.03, 117.31)	37.56 (1.84, 764.82)	7.21 (0.44, 117.25)	7.35 (0.45, 120.49)	8.36 (0.49, 141.65)	10.31 (0.55, 192.04)	7.45 (0.44, 125.21)	2.91 (1.00, 8.50)	1.46 (0.20, 10.85)	0.85 (0.49, 1.48)	1.37 (0.27, 6.88)	2.58 (0.65, 10.17)	1.35 (0.68, 2.70)	2.56 (0.55, 11.95)	**hmcr & educ & mfar**			0.95 (0.67, 1.36)																				
10.95 (0.43, 275.95)	13.98 (0.62, 314.52)	13.98 (0.64, 303.44)	3.07 (0.04, 232.40)	61.26 (2.32, 1620.34)	11.76 (0.55, 253.23)	11.99 (0.55, 260.02)	13.63 (0.61, 304.78)	16.81 (0.69, 409.82)	12.16 (0.55, 269.63)	4.75 (0.89, 25.26)	2.39 (0.22, 25.77)	1.39 (0.34, 5.60)	2.24 (0.29, 17.56)	4.20 (0.64, 27.51)	2.20 (0.51, 9.46)	4.18 (0.56, 31.00)	1.63 (0.40, 6.64)	**hmcr & aids**		0.58 (0.15, 2.21)																				
3.66 (0.22, 60.73)	4.67 (0.32, 68.00)	4.67 (0.33, 65.22)	1.02 (0.02, 57.42)	20.47 (1.17, 359.11)	3.93 (0.28, 54.36)	4.00 (0.29, 55.89)	4.55 (0.32, 65.83)	5.62 (0.35, 89.74)	4.06 (0.28, 58.16)	1.59 (0.43, 5.84)	0.80 (0.14, 4.69)	0.46 (0.18, 1.17)	0.75 (0.20, 2.78)	1.40 (0.51, 3.84)	0.74 (0.73, 2.03)	1.40 (0.25, 7.71)	0.54 (0.21, 1.39)	0.33 (0.07, 1.63)	**hmcr & ADL & mfar(w/slfm)**	1.75 (0.77, 3.98)																				
6.38 (0.34, 120.06)	8.16 (0.49, 135.22)	8.16 (0.51, 129.94)	1.79 (0.03, 109.44)	35.73 (1.80, 708.27)	6.86 (0.43, 108.35)	6.99 (0.44, 111.34)	7.95 (0.48, 130.95)	9.81 (0.54, 177.69)	7.09 (0.43, 115.74)	2.77 (1.02, 7.48)	1.39 (0.20, 9.91)	0.81 (0.55, 1.18)	1.31 (0.27, 6.22)	2.45 (0.66, 9.11)	1.29 (0.73, 2.25)	2.44 (0.55, 10.77)	0.95 (0.64, 1.42)	0.58 (0.15, 2.24)	1.75 (0.75, 4.06)	**hmcr**																				
																					**exrc & psyc**	**exrc & mfar(w/med+slfm)**	**exrc & mfa-(w/med)**	**exrc**	**educ & rsk-mfa-**	**educ & mfar(w/med+slfm)**	**educ & mfar(w/med)**	**educ & mfa-**	**educ & exrc & mfar(w/slfm)**	**educ & exrc & mfar(w/med+slfm)**	**educ**	**cgn & ntr & exrc**	**cgn & med & ntr & exrc**	**aids**	**ADL&aids&ed&ex&mf(w/med+slfm)**	**ADL & ntr & exrc**	**ADL & exrc**	**ADL & aids & exrc**	**ADL**	**ac**

wlfr	vchr	rsk-mfa-	ntr & exrc	mntr-mfa-	mfar(w/med)	mfar	mfa-(w/med+slfm)	mfa-(w/med)	mfa-	hmcr & ntr	hmcr & mfar(w/slfm)	hmcr & mfar(w/med)	hmcr & mfar	hmcr & mfa-	hmcr & med	hmcr & hmnt & exrc	hmcr & educ & mfar	hmcr & aids	hmcr & ADL & mfar(w/slfm)	hmcr																				
0.20 (0.02, 2.25)	0.25 (0.03, 2.47)	0.25 (0.03, 2.35)	0.06 (0.00, 2.41)	1.11 (0.09, 13.44)	0.21 (0.02, 1.96)	0.22 (0.02, 2.01)	0.25 (0.03, 2.39)	0.30 (0.03, 3.31)	0.22 (0.02, 2.11)	0.09 (0.00, 3.36)	0.04 (0.00, 1.27)	0.03 (0.00, 0.87)	0.04 (0.00, 0.96)	0.08 (0.00, 2.01)	0.04 (0.00, 1.42)	0.08 (0.00, 3.48)	0.03 (0.00, 1.03)	0.02 (0.00, 0.79)	0.05 (0.00, 1.67)	0.03 (0.00, 1.06)	**exrc & psyc**																			4.06 (0.45, 36.83)
0.66 (0.17, 2.55)	0.84 (0.29, 2.42)	0.84 (0.33, 2.16)	0.18 (0.01, 4.45)	3.67 (0.85, 15.94)	0.71 (0.28, 1.77)	0.72 (0.28, 1.85)	0.82 (0.29, 2.31)	1.01 (0.28, 3.61)	0.73 (0.26, 2.01)	0.28 (0.01, 6.08)	0.14 (0.01, 2.16)	0.08 (0.00, 1.54)	0.13 (0.01, 1.54)	0.25 (0.02, 3.33)	0.13 (0.01, 2.52)	0.25 (0.01, 6.50)	0.10 (0.01, 1.82)	0.06 (0.00, 1.46)	0.18 (0.01, 2.86)	0.10 (0.01, 1.86)	3.32 (0.30, 36.21)	**exrc & mfar(w/med+slfm)**																		1.22 (0.51, 2.95)
0.53 (0.07, 4.24)	0.68 (0.10, 4.51)	0.68 (0.11, 4.25)	0.15 (0.00, 5.20)	2.98 (0.35, 25.54)	0.57 (0.09, 3.53)	0.58 (0.09, 3.64)	0.66 (0.10, 4.36)	0.82 (0.11, 6.18)	0.59 (0.09, 3.83)	0.23 (0.01, 7.20)	0.12 (0.01, 2.67)	0.07 (0.00, 1.86)	0.11 (0.01, 1.98)	0.20 (0.01, 4.19)	0.11 (0.00, 3.03)	0.20 (0.01, 7.54)	0.08 (0.00, 2.19)	0.05 (0.00, 1.71)	0.15 (0.01, 3.51)	0.08 (0.00, 2.25)	2.69 (0.15, 46.96)	0.81 (0.11, 6.12)	**exrc & mfa-(w/med)**																	1.51 (0.25, 9.11)
4.84 (0.45, 51.51)	6.18 (0.68, 56.18)	6.19 (0.72, 53.39)	1.36 (0.03, 56.48)	27.09 (2.39, 307.69)	5.20 (0.61, 44.41)	5.30 (0.61, 45.74)	6.03 (0.67, 54.30)	7.43 (0.73, 75.56)	5.38 (0.60, 47.88)	2.10 (0.06, 78.59)	1.06 (0.04, 29.61)	0.61 (0.02, 20.39)	0.99 (0.04, 22.29)	1.86 (0.07, 46.84)	0.97 (0.03, 33.22)	1.85 (0.04, 81.62)	0.72 (0.02, 24.06)	0.44 (0.01, 18.51)	1.32 (0.05, 38.87)	0.76 (0.02, 24.70)	24.51 (1.13, 530.54)	7.38 (0.73, 74.86)	9.09 (0.55, 149.36)	**exrc**																0.17 (0.02, 1.39)
0.80 (0.24, 2.69)	1.02 (0.43, 2.43)	1.03 (0.50, 2.11)	0.22 (0.01, 5.13)	4.49 (1.18, 17.03)	0.86 (0.43, 1.71)	0.88 (0.43, 1.80)	1.00 (0.43, 2.31)	1.23 (0.40, 3.77)	0.89 (0.40, 2.00)	0.35 (0.02, 6.98)	0.18 (0.01, 2.46)	0.10 (0.01, 1.77)	0.16 (0.02, 1.74)	0.31 (0.03, 3.78)	0.16 (0.01, 2.89)	0.31 (0.01, 7.49)	0.12 (0.01, 2.08)	0.07 (0.00, 1.68)	0.22 (0.01, 3.27)	0.13 (0.01, 2.13)	4.06 (0.40, 40.85)	1.22 (0.40, 3.73)	1.51 (0.22, 10.33)	0.17 (0.02, 1.55)	**educ & rsk-mfa-**															1.00 (0.53, 1.88)
0.70 (0.22, 2.23)	0.89 (0.40, 1.97)	0.89 (0.47, 1.68)	0.20 (0.01, 4.38)	3.91 (1.08, 14.17)	0.75 (0.41, 1.36)	0.76 (0.41, 1.44)	0.87 (0.40, 1.87)	1.07 (0.37, 3.11)	0.78 (0.37, 1.62)	0.30 (0.02, 5.96)	0.15 (0.01, 2.09)	0.09 (0.01, 1.50)	0.14 (0.01, 1.48)	0.27 (0.02, 3.21)	0.14 (0.01, 2.47)	0.27 (0.01, 6.40)	0.10 (0.01, 1.78)	0.06 (0.00, 1.44)	0.19 (0.01, 2.78)	0.11 (0.01, 1.82)	3.54 (0.36, 34.66)	1.06 (0.37, 3.07)	1.31 (0.20, 8.72)	0.14 (0.02, 1.31)	0.87 (0.37, 2.07)	**educ & mfar(w/med+slfm)**														1.14 (0.66, 1.97)
0.73 (0.24, 2.17)	0.93 (0.46, 1.85)	0.93 (0.56, 1.54)	0.20 (0.01, 4.45)	4.07 (1.19, 13.92)	0.78 (0.49, 1.23)	0.80 (0.48, 1.32)	0.90 (0.47, 1.76)	1.12 (0.41, 3.01)	0.81 (0.43, 1.50)	0.32 (0.02, 6.05)	0.16 (0.01, 2.12)	0.09 (0.01, 1.53)	0.15 (0.01, 1.49)	0.28 (0.02, 3.25)	0.15 (0.01, 2.50)	0.28 (0.01, 6.51)	0.11 (0.01, 1.80)	0.07 (0.00, 1.46)	0.20 (0.01, 2.82)	0.11 (0.01, 1.84)	3.68 (0.39, 34.92)	1.11 (0.41, 2.98)	1.36 (0.21, 8.73)	0.15 (0.02, 1.32)	0.91 (0.42, 1.97)	1.04 (0.52, 2.09)	**educ & mfar(w/med)**													1.11 (0.76, 1.62)
0.61 (0.08, 4.50)	0.78 (0.13, 4.76)	0.78 (0.14, 4.47)	0.17 (0.01, 5.71)	3.41 (0.43, 27.22)	0.65 (0.12, 3.71)	0.67 (0.12, 3.83)	0.76 (0.13, 4.59)	0.94 (0.13, 6.55)	0.68 (0.11, 4.04)	0.26 (0.01, 7.89)	0.13 (0.01, 2.91)	0.08 (0.00, 2.03)	0.12 (0.01, 2.15)	0.23 (0.01, 4.56)	0.12 (0.00, 3.31)	0.23 (0.01, 8.27)	0.09 (0.00, 2.39)	0.06 (0.00, 1.87)	0.17 (0.01, 3.83)	0.10 (0.00, 2.45)	3.08 (0.19, 50.95)	0.93 (0.13, 6.49)	1.14 (0.09, 13.92)	0.13 (0.01, 1.96)	0.76 (0.12, 4.81)	0.87 (0.14, 5.34)	0.84 (0.14, 4.93)	**educ & mfa-**												1.32 (0.24, 7.31)
0.67 (0.13, 3.62)	0.86 (0.20, 3.68)	0.86 (0.22, 3.40)	0.19 (0.01, 5.32)	3.77 (0.64, 22.20)	0.72 (0.19, 2.81)	0.74 (0.19, 2.91)	0.84 (0.20, 3.54)	1.04 (0.21, 5.21)	0.75 (0.18, 3.10)	0.29 (0.01, 7.31)	0.15 (0.01, 2.65)	0.09 (0.00, 1.87)	0.14 (0.01, 1.92)	0.26 (0.02, 4.11)	0.14 (0.01, 3.05)	0.26 (0.01, 7.74)	0.10 (0.00, 2.20)	0.06 (0.00, 1.74)	0.18 (0.01, 3.50)	0.11 (0.00, 2.26)	3.41 (0.26, 45.36)	1.03 (0.20, 5.16)	1.27 (0.13, 12.04)	0.14 (0.01, 1.73)	0.84 (0.19, 3.74)	0.97 (0.23, 4.13)	0.93 (0.23, 3.77)	1.11 (0.12, 9.83)	**educ & exrc & mfar(w/slfm)**											1.19 (0.32, 4.48)
1.64 (0.12, 22.71)	2.09 (0.17, 25.19)	2.09 (0.18, 24.08)	0.46 (0.01, 22.73)	9.17 (0.62, 134.81)	1.76 (0.15, 20.06)	1.79 (0.16, 20.63)	2.04 (0.17, 24.37)	2.52 (0.19, 33.47)	1.82 (0.15, 21.52)	0.71 (0.02, 31.79)	0.36 (0.01, 12.15)	0.21 (0.01, 8.29)	0.34 (0.01, 9.27)	0.63 (0.02, 19.34)	0.33 (0.01, 13.49)	0.63 (0.01, 32.77)	0.24 (0.01, 9.78)	0.15 (0.00, 7.45)	0.45 (0.01, 15.91)	0.26 (0.01, 10.06)	8.30 (0.31, 221.05)	2.50 (0.19, 33.17)	3.08 (0.15, 63.43)	0.34 (0.01, 8.57)	2.04 (0.17, 25.20)	2.35 (0.19, 28.27)	2.26 (0.19, 26.40)	2.69 (0.14, 52.74)	2.43 (0.15, 38.79)	**educ & exrc & mfar(w/med+slfm)**										0.49 (0.04, 5.49)
0.57 (0.03, 11.19)	0.73 (0.04, 12.62)	0.73 (0.04, 12.14)	0.16 (0.00, 10.06)	3.18 (0.15, 65.93)	0.61 (0.04, 10.12)	0.62 (0.04, 10.40)	0.71 (0.04, 12.23)	0.87 (0.05, 16.56)	0.63 (0.04, 10.81)	0.25 (0.00, 14.16)	0.12 (0.00, 5.51)	0.07 (0.00, 3.72)	0.12 (0.00, 4.27)	0.22 (0.01, 8.84)	0.11 (0.00, 6.05)	0.22 (0.00, 14.46)	0.08 (0.00, 4.39)	0.05 (0.00, 3.30)	0.16 (0.00, 7.20)	0.09 (0.00, 4.52)	2.88 (0.08, 102.14)	0.87 (0.05, 16.42)	1.07 (0.04, 29.96)	0.12 (0.00, 3.98)	0.71 (0.04, 12.59)	0.81 (0.05, 14.16)	0.78 (0.05, 13.27)	0.93 (0.03, 25.04)	0.84 (0.04, 18.80)	0.35 (0.01, 14.09)	**educ**									1.41 (0.09, 23.10)
2.51 (0.08, 74.26)	3.21 (0.12, 85.11)	3.21 (0.13, 82.26)	0.70 (0.01, 60.13)	14.05 (0.45, 435.06)	2.70 (0.11, 68.68)	2.75 (0.11, 70.49)	3.13 (0.12, 82.50)	3.86 (0.13, 110.46)	2.79 (0.11, 73.02)	1.09 (0.01, 85.15)	0.55 (0.01, 33.79)	0.32 (0.00, 22.55)	0.51 (0.01, 26.59)	0.96 (0.02, 54.58)	0.51 (0.01, 36.58)	0.96 (0.01, 86.08)	0.37 (0.01, 26.59)	0.23 (0.00, 19.69)	0.69 (0.01, 43.97)	0.39 (0.01, 27.42)	12.71 (0.25, 638.04)	3.83 (0.13, 109.49)	4.72 (0.12, 191.36)	0.52 (0.01, 24.94)	3.13 (0.12, 84.63)	3.59 (0.14, 95.48)	3.46 (0.13, 89.79)	4.12 (0.11, 160.62)	3.72 (0.11, 123.01)	1.53 (0.03, 87.01)	4.42 (0.06, 317.95)	**cgn & ntr & exrc**								0.32 (0.01, 8.05)
1.64 (0.39, 6.94)	2.10 (0.66, 6.72)	2.10 (0.73, 6.08)	0.46 (0.02, 11.57)	9.20 (1.96, 43.15)	1.77 (0.62, 5.00)	1.80 (0.62, 5.21)	2.05 (0.65, 6.45)	2.53 (0.65, 9.88)	1.83 (0.59, 5.62)	0.71 (0.03, 15.82)	0.36 (0.02, 5.65)	0.21 (0.01, 4.02)	0.34 (0.03, 4.04)	0.63 (0.05, 8.72)	0.33 (0.02, 6.58)	0.63 (0.02, 16.87)	0.25 (0.01, 4.75)	0.15 (0.01, 3.79)	0.45 (0.03, 7.48)	0.26 (0.01, 4.85)	8.33 (0.73, 95.22)	2.51 (0.64, 9.78)	3.09 (0.39, 24.67)	0.34 (0.03, 3.62)	2.05 (0.61, 6.91)	2.35 (0.73, 7.55)	2.26 (0.75, 6.81)	2.70 (0.36, 20.04)	2.44 (0.45, 13.14)	1.00 (0.07, 13.93)	2.89 (0.15, 57.06)	0.66 (0.02, 19.41)	**cgn & med & ntr & exrc**							0.49 (0.18, 1.33)
0.84 (0.04, 18.30)	1.08 (0.06, 20.74)	1.08 (0.06, 19.96)	0.24 (0.00, 16.04)	4.72 (0.21, 107.72)	0.91 (0.05, 16.60)	0.92 (0.05, 16.85)	1.05 (0.05, 20.09)	1.30 (0.06, 27.07)	0.94 (0.05, 16.47)	0.37 (0.01, 22.60)	0.18 (0.00, 8.84)	0.11 (0.00, 5.97)	0.17 (0.00, 6.87)	0.32 (0.01, 14.19)	0.17 (0.00, 9.66)	0.32 (0.00, 23.02)	0.13 (0.00, 7.02)	0.08 (0.00, 5.25)	0.23 (0.00, 11.53)	0.13 (0.00, 7.23)	4.27 (0.11, 164.55)	1.29 (0.06, 26.90)	1.59 (0.05, 48.55)	0.17 (0.00, 6.41)	1.05 (0.05, 20.67)	1.21 (0.06, 23.29)	1.16 (0.06, 21.81)	1.39 (0.05, 40.61)	1.25 (0.05, 30.65)	0.51 (0.01, 22.63)	1.49 (0.03, 83.97)	0.34 (0.00, 25.91)	0.51 (0.02, 11.15)	**aids**						
0.10 (0.01, 1.00)	0.12 (0.01, 1.09)	0.12 (0.01, 1.04)	0.03 (0.00, 1.11)	0.54 (0.05, 6.00)	0.10 (0.01, 0.86)	0.11 (0.01, 0.89)	0.12 (0.01, 1.06)	0.15 (0.02, 1.47)	0.11 (0.01, 0.93)	0.04 (0.00, 1.55)	0.02 (0.00, 0.58)	0.01 (0.00, 0.40)	0.02 (0.00, 0.44)	0.04 (0.00, 0.92)	0.02 (0.00, 0.65)	0.04 (0.00, 1.61)	0.01 (0.00, 0.47)	0.01 (0.00, 0.36)	0.03 (0.00, 0.76)	0.02 (0.00, 0.49)	0.49 (0.02, 10.41)	0.15 (0.02, 1.46)	0.18 (0.01, 2.92)	0.02 (0.00, 0.40)	0.12 (0.01, 1.10)	0.14 (0.02, 1.23)	0.13 (0.02, 1.14)	0.16 (0.01, 2.42)	0.14 (0.01, 1.74)	0.06 (0.00, 1.47)	0.17 (0.01, 5.68)	0.04 (0.00, 1.83)	0.06 (0.01, 0.61)	0.12 (0.00, 4.15)	**ADL&aids&ed&ex&mf(w/med+slfm)**					8.25 (1.02, 66.86)
1.68 (0.37, 7.58)	2.14 (0.62, 7.45)	2.14 (0.68, 6.79)	0.47 (0.02, 12.17)	9.39 (1.88, 46.89)	1.80 (0.58, 5.59)	1.84 (0.58, 5.81)	2.09 (0.61, 7.15)	2.58 (0.61, 10.82)	1.86 (0.56, 6.25)	0.73 (0.03, 16.66)	0.37 (0.02, 5.97)	0.21 (0.01, 4.24)	0.34 (0.03, 4.29)	0.64 (0.04, 9.24)	0.34 (0.02, 6.94)	0.64 (0.02, 17.73)	0.25 (0.01, 5.00)	0.15 (0.01, 3.99)	0.46 (0.03, 7.91)	0.26 (0.01, 5.12)	8.49 (0.71, 101.15)	2.56 (0.61, 10.71)	3.15 (0.38, 26.39)	0.35 (0.03, 3.85)	2.09 (0.57, 7.63)	2.40 (0.69, 8.37)	2.31 (0.70, 7.58)	2.75 (0.35, 21.47)	2.49 (0.43, 14.21)	1.02 (0.07, 14.76)	2.95 (0.14, 60.17)	0.67 (0.02, 20.39)	1.02 (0.22, 4.63)	1.99 (0.09, 44.60)	17.24 (1.60, 186.01)	**ADL & ntr & exrc**				0.48 (0.16, 1.44)
0.52 (0.10, 2.75)	0.67 (0.16, 2.78)	0.67 (0.17, 2.56)	0.15 (0.01, 4.08)	2.93 (0.51, 16.86)	0.56 (0.15, 2.12)	0.57 (0.15, 2.20)	0.65 (0.16, 2.67)	0.80 (0.16, 3.95)	0.58 (0.14, 2.34)	0.23 (0.01, 5.60)	0.11 (0.01, 2.03)	0.07 (0.00, 1.43)	0.11 (0.01, 1.47)	0.20 (0.01, 3.15)	0.11 (0.00, 2.34)	0.20 (0.01, 5.94)	0.08 (0.00, 1.69)	0.05 (0.00, 1.34)	0.14 (0.01, 2.68)	0.08 (0.00, 1.73)	2.65 (0.20, 34.68)	0.80 (0.16, 3.91)	0.98 (0.11, 9.18)	0.11 (0.01, 1.32)	0.65 (0.15, 2.83)	0.75 (0.18, 3.12)	0.72 (0.18, 2.85)	0.86 (0.10, 7.50)	0.78 (0.12, 5.06)	0.32 (0.02, 5.03)	0.92 (0.04, 20.29)	0.21 (0.01, 6.81)	0.32 (0.06, 1.68)	0.62 (0.03, 15.00)	5.38 (0.45, 64.01)	0.31 (0.06, 1.75)	**ADL & exrc**			1.53 (0.42, 5.62)
5.17 (0.83, 32.34)	6.61 (1.30, 33.51)	6.61 (1.40, 31.24)	1.45 (0.05, 44.16)	28.96 (4.26, 196.69)	5.56 (1.19, 25.88)	5.67 (1.20, 26.77)	6.44 (1.29, 32.29)	7.95 (1.35, 46.78)	5.75 (1.17, 28.35)	2.24 (0.08, 60.84)	1.13 (0.06, 22.22)	0.66 (0.03, 15.60)	1.06 (0.07, 16.26)	1.99 (0.11, 34.68)	1.04 (0.04, 25.47)	1.98 (0.06, 64.15)	0.77 (0.03, 18.40)	0.47 (0.02, 14.48)	1.41 (0.07, 29.32)	0.81 (0.03, 18.85)	26.19 (1.78, 384.82)	7.88 (1.34, 46.33)	9.72 (0.91, 103.59)	1.07 (0.08, 14.74)	6.45 (1.22, 33.96)	7.41 (1.46, 37.63)	7.12 (1.47, 34.58)	8.50 (0.85, 84.92)	7.67 (1.01, 58.42)	3.16 (0.18, 55.36)	9.10 (0.38, 221.01)	2.06 (0.06, 73.43)	3.15 (0.50, 19.72)	6.13 (0.23, 162.98)	53.18 (3.96, 713.37)	3.09 (0.47, 20.41)	9.89 (1.32, 73.98)	**ADL & aids & exrc**		0.16 (0.03, 0.70)
0.78 (0.21, 2.93)	0.99 (0.36, 2.76)	0.99 (0.40, 2.45)	0.22 (0.01, 5.21)	4.35 (1.03, 18.38)	0.83 (0.35, 2.00)	0.85 (0.35, 2.06)	0.97 (0.36, 2.64)	1.19 (0.34, 4.13)	0.86 (0.36, 2.08)	0.34 (0.02, 7.11)	0.17 (0.01, 2.52)	0.10 (0.01, 1.80)	0.16 (0.01, 1.79)	0.30 (0.02, 3.88)	0.16 (0.01, 2.95)	0.30 (0.01, 7.60)	0.12 (0.01, 2.13)	0.07 (0.00, 1.71)	0.21 (0.01, 3.34)	0.12 (0.01, 2.17)	3.93 (0.37, 42.19)	1.18 (0.34, 4.10)	1.46 (0.20, 10.80)	0.16 (0.02, 1.60)	0.97 (0.33, 2.85)	1.11 (0.40, 3.10)	1.07 (0.41, 2.76)	1.28 (0.19, 8.75)	1.15 (0.23, 5.65)	0.47 (0.04, 6.20)	1.37 (0.07, 25.59)	0.31 (0.01, 8.76)	0.47 (0.12, 1.79)	0.92 (0.05, 18.46)	7.99 (0.83, 77.23)	0.46 (0.11, 1.89)	1.49 (0.31, 7.12)	0.15 (0.03, 0.86)	**ADL**	1.01 (0.35, 2.92)
0.80 (0.29, 2.22)	1.02 (0.59, 1.79)	1.03 (0.76, 1.37)	0.22 (0.01, 4.78)	4.49 (1.41, 14.31)	0.86 (0.71, 1.05)	0.88 (0.66, 1.18)	1.00 (0.59, 1.68)	1.23 (0.50, 3.04)	0.89 (0.56, 1.43)	0.35 (0.02, 6.49)	0.18 (0.01, 2.26)	0.10 (0.01, 1.64)	0.16 (0.02, 1.58)	0.31 (0.03, 3.46)	0.16 (0.01, 2.68)	0.31 (0.01, 7.00)	0.12 (0.01, 1.93)	0.07 (0.00, 1.57)	0.22 (0.02, 3.01)	0.13 (0.01, 1.97)	4.06 (0.44, 37.12)	1.22 (0.50, 3.01)	1.51 (0.25, 9.20)	0.17 (0.02, 1.40)	1.00 (0.52, 1.93)	1.15 (0.66, 2.01)	1.10 (0.73, 1.67)	1.32 (0.23, 7.39)	1.19 (0.31, 4.54)	0.49 (0.04, 5.53)	1.41 (0.09, 23.24)	0.32 (0.01, 8.09)	0.49 (0.18, 1.35)	0.95 (0.05, 17.35)	8.25 (1.01, 67.42)	0.48 (0.16, 1.46)	1.53 (0.41, 5.70)	0.16 (0.03, 0.71)	1.03 (0.44, 2.43)	**ac**

Please note: the results of mortality: medium-term network are too large to fit on one page. Please see https://doi.org/10.5518/1377

Table 106 - Intervention rankings for mortality: medium-term network

Treatment	SUCRA	Pr(Best)	Mean Rank	LCI Rank	UCI Rank
hmcr & aids	89.7	29.2	5.1	1	27
hmcr & mfar(w/med)	87.5	7.5	6	1	27
hmcr & educ & mfar	83.2	2.2	7.7	1	30
hmcr	82.1	0.4	8.1	2	29
ADL & aids & exrc	81.1	8.4	8.6	1	19
exrc	78.5	12.2	9.6	1	34
hmcr & mfar	78.5	1.2	9.6	2	28
hmcr & med	75.6	0.5	10.8	2	34
hmcr & mfar(w/slfm)	75.4	4.3	10.8	1	34
ntr & exrc	69.9	14.1	13	1	39
hmcr & ADL & mfar(w/slfm)	69.8	0.1	13.1	5	35
cgn & ntr & exrc	63.5	12.1	15.6	1	40
hmcr & mfa-	62.8	0	15.9	7	37
ADL & ntr & exrc	62.1	0.2	16.1	4	33
cgn & med & ntr & exrc	61.7	0	16.3	4	32
hmcr & hmnt & exrc	60.5	0.9	16.8	2	40
educ & exrc & mfar(w/med+slfm)	57.7	3.1	17.9	1	40
hmcr & ntr	57.6	0.2	17.9	6	39
mfar(w/med)	47.4	0	22.1	13	29
wlfr	45.7	0	22.7	7	37
mfar	45.3	0	22.9	12	31
mfa-	44.4	0	23.2	11	34
aids	41	2.2	24.6	2	41
mfa-(w/med+slfm)	38.6	0	25.5	14	35
ADL	37.8	0	25.9	12	37
educ & rsk-mfa-	37.8	0	25.9	11	36
vchr	37.7	0	25.9	14	36
ac	37.5	0	26	18	31
rsk-mfa-	36.5	0	26.4	16	34
educ	34.6	1.1	27.2	3	41
educ & exrc & mfar(w/slfm)	33	0.1	27.8	8	39
educ & mfar(w/med)	32.9	0	27.8	17	36
educ & mfar(w/med+slfm)	31.6	0	28.4	16	36
educ & mfa-	31.4	0	28.4	7	40
exrc & mfar(w/med+slfm)	31.1	0	28.6	11	38
mfa-(w/med)	30.5	0	28.8	14	38
exrc & mfa-(w/med)	28.2	0	29.7	8	41
ADL & exrc	25.2	0	30.9	11	40
exrc & psyc	12.7	0	35.9	15	41
mntr-mfa-	6.2	0	38.5	32	41
ADL&aids&ed&ex&mf(w/med+slfm)	5.4	0	38.9	23	41

SUCRA values (0–100) and mean ranks are presented, based on 1000 simulations. Higher SUCRAs and lower mean ranks indicate better performing interventions. Pr(Best) gives the probability of each specific intervention being ranked best intervention, based on 1000 simulations.

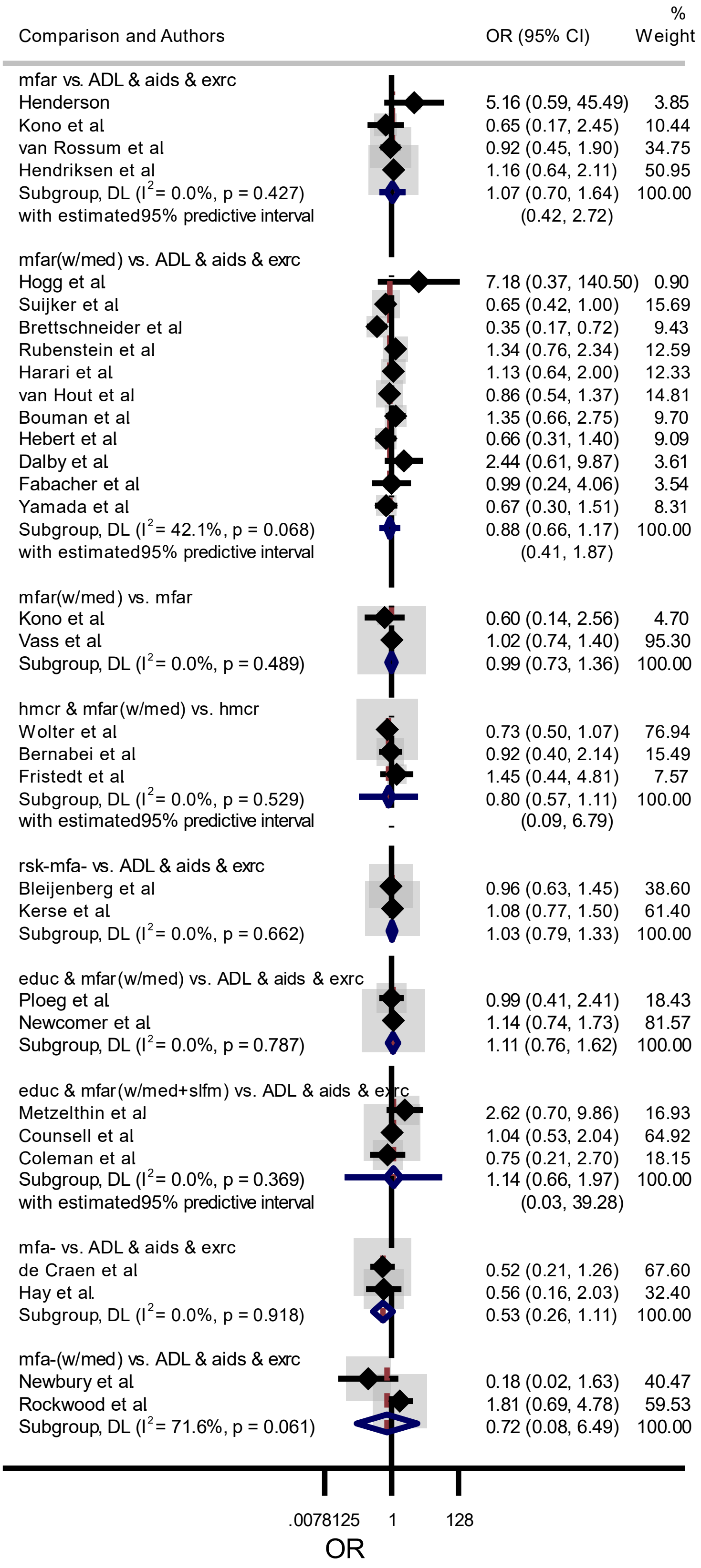

Figure 49 - Pairwise meta-analysis for mortality: medium-term network (pooling comparisons with greater than one study reporting results

Appendix 12. Health economic evaluations and results

Table 107 - Economic evaluation analysis and principal findings reported for the short-term time horizon

Study	Intervention 1 (Int1) *(Experimental intervention)*	Intervention 2 (Int2) *(comparator)*	Perspective	Time horizon (months)	Evaluation[a] type: Cost analysis	Evaluation[a] type: Cost-effectiveness analysis	Evaluation[a] type: Cost-utility analysis	Evaluation[a] type: Cost-benefit analysis	Cost items category[a]: Health sector costs	Cost items category[a]: Other sector costs	Cost items category[a]: Patient and family costs	Cost items category[a]: Productivity impacts	Intervention cost items	Currency / Price year / Principal findings / Conclusion of study	Study conclusion[b]
Clark 1997[519]	eng & educ<br>n = 51/51	ac<br>n = 112/112	Soc (inc. HSCS)	9	●		●		●	●<br>Paid caregiver only	●<br>Unpaid caregiver only		Int1: Staff time spent on preparation, contacts, and travelling;<br>Int2: Staff time spent on preparation and contacts	**Currency / Price year**: USD$, 1995; annual discount rate of 3% for costs<br>**Total costs** (annualised mean per person):<br>***(during treatment) Int1***: $4741 (SD $11,654), ***Int2***: $4723 (SD $11,321)<br>**Conclusion**: "Preventive OT demonstrated cost-effectiveness in conjunction with a trend toward decreased medical expenditures."<br>**Notes:** Post-treatment total costs and ICER were estimated at 15 months.	✓
Clark 2012[520]	eng & educ<br>n = 232/232	ac<br>n = 228/228	HSCS	6	●		●		●<br>Experimental intervention costs only				Salaries of intervention therapists	**Currency / Price year**: USD$, 2007-8<br>**Experimental intervention costs** (mean per person): $783 (approx. £472.5)<br>**ICER** (per QALY): $41,218 (approx. £24,868), a number within the range that is often considered cost-effective by the UK NICE.<br>**Conclusion**: "A lifestyle-oriented occupational therapy intervention has beneficial effects for ethnically diverse older people recruited from a wide array of community settings. Because the intervention is cost-effective and is applicable on a wide-scale basis, it has the potential to help reduce health decline and promote well-being in older people."	✓
Melis 2008[573]	mfar(w/med)<br>n = 85/88	ac<br>n = 66/67	HSCS	6	●	●		●	●	●			Staff time spent on	**Currency / Price year**: EUR€, 2005<br>**Total cost** (mean per person): ***Int1***: €9713(SD €10,205), ***Int2***: €8952(SD €9757);	✓

													Consult-ation, phone calls, traveling, and adminis-tration	***mean difference***[c]: €761 (95% CI €-3336 to €4687)<br>**Experimental intervention costs** (mean per person): €998 (95% CI €888 to 1108)<br>**ICER** (per successful treatment[d]): €3418 (95% CI €-21,458 to 45,362)<br>**CE plane**: 34.6% ICERs in southeast quadrant[e]<br>**WTP**: ICER is roughly €3500 per successful treatment. The new treatment is cost-effective at a WTP of €34,000.<br>**Conclusion**: "The results of this economic evaluation suggest that DGIP is an effective addition to primary care for frail older people at a reasonable cost."	
Tuntland 2015[614]	hmcr & ADL & aids & mfa- (w/slfm)<br>n = 25/31	hmcr & mfa-<br>n = 21/30	HSCS	9	●	●[f]		●	●<br>Home visits from healthcare professionals only	●<br>Home care only			Staff time spent on home visits	**Currency / Price year:** NOK, (assumed) 2012-4<br>**Total costs** (mean per person, 3-9 months post-intervention phase only):<br>***Int1***: 6470.82 (SD 10,559.00) NOK, ***Int2***: 13914.31 (SD 28,926.05) NOK;<br>***mean difference***[c]: -7443.23 NOK<br>**Intervention costs** (mean per person): ***Int1***: 6322.78 (SD 4101.98) NOK, ***Int2***: 7456.77 (SD 12,952.97) NOK;<br>***mean difference***: -1134.00 NOK<br>**Conclusions**: "We conclude that reablement stands out as a promising intervention, not only because it seems to decrease expenditure, but also because older adults feel they improve their performance and satisfaction in daily life activities. The combination of lower costs and higher effects is the kind of policy measure that will be of interest to policy-makers. Reablement is a more cost-effective intervention compared with usual care. Reablement has a potentially large effect on the demand for compensating home-based care services. Policy-makers should therefore consider implementing reablement on a larger scale."	✓
Stewart 2005[601]	mfa-<br>n = 160/160	mfa-<br>n =161/161	HSCS	8	●	●	●		●	●<br>Also social services, equipment and adaptations	●<br>Out-of-pocket expenses only, e.g., non-prescription medications, travel, additional household costs.		Not specified but analysed as part of total costs	**Currency / Price year**: GBP£ (£1 = US $1.59 = EUR €1.47), 2001; reported no discounting<br>Total costs (mean per person):<br>***Int1***: £4379 (SD £4173), ***Int2***: £3837 (SD £4736); ***mean difference***[c]: £543 (95% CI -434 to 1519)<br>**CEA curve**: At best, occupational therapy assessment would improve outcomes at a cost of £14,000 per QALY. The probability of such an outcome was <50% (similar results presented for distribution of values of	✗

														ICER based on Community Dependence Index (CDI) from bootstrap estimation).<br>**Conclusions**: "From a policy perspective, the lack of difference in clinical and cost-effectiveness means that either a social work or an occupational therapy service is successful in making care assessments that enable an older person to remain in their own home."	
van der Pols-Vijlbrief 2017[615]	hmcr & ntr & mfar<br>n = 79/79[f]	hmcr<br>n = 76/76[g]	Soc (inc. HSCS)	6	●		●	●	●	●	●<br>Informal care only		Implementing action plan, staff time needed for support and visits	**Currency / Price year**: EUR€, 2014; reported no discounting<br>**Total costs** (mean per person):<br>***Int1:*** €2770 (SE €347), ***Int2***: €3044 (SE €325);<br>***mean differenc***[c]: €-274, 95% CI €-1111 to €782.<br>**Experimental intervention costs** (mean, per person): €41 (SE €0.47)<br>**ICER** (per QALY): €-32173<br>**CE plane:** 55% cost-effect pairs in southeast quadrant, 21% northeast, 18% in southwest, 6% in northwest quadrants[e].<br>**CEA curve**: Probability of cost-effectiveness 0.80 at a WTP of €20,000/QALY gained.<br>**Conclusion**: "The intervention cannot be considered cost-effective in comparison with usual care for […] QALYs gained. [It] showed stronger effects in participants who completed the intervention and actually executed the recommendations given. Therefore, future studies should take into account motivation and capability as potential key factors for a successful intervention."	✗
Wong 2019[630]	mfar(w/slfm)<br>n = 230/271	ac<br>n = 229/269	HSCS	6	●		●	●	●		●<br>Private GP visits only		(Int1) Staff time on staff training, staff time on intervention delivery (home visits, calls, adminis-tration), equipment; (Int2) Staff time on staff training, staff time on intervention delivery (calls)	**Currency / Price year**: HKD$, 2018<br>**Total costs** (mean per person):<br>***Int1***: $3979, ***Int2***: $3623;<br>***mean difference***[c]: $356; 95% CI $272 to $440.<br>**Intervention costs** (mean per person):<br>***Int1***: $1263, ***Int2***: $68<br>**ICER** (per QALY): $109,453 (95% CI $83,719 to $135,189)<br>**CE plane**: 12.0% ICERs in the southeast quadrant[e].<br>**CEA curve**: The preventive self-care health management program had a 53.2% likelihood of being cost-effective when considering the NICE threshold ($200,000/QALY), and a 53.4% likelihood of being	~

														cost-effective compared to WHO (Hong Kong GDP/capita, $381,780)<br>**Conclusion**: "The results provide some evidence to suggest that the addition of a home-based, preventive self-care health management program may have effects on cost outcomes for community-dwelling older adults in Hong Kong."	
Challis 2004 2004[518]	mfar(w/med)<br>n =129/129	mfar<br>n =127/127	Soc (inc. HSCS)	6	●				●	●	●<br>Personal consumption, housing, informal care only		Not specified but analysed as part of total costs	**Currency / Price year**: GBP£, 2000-1<br>**Total costs** (mean per week alive, per person):<br>***Int1***: £607, ***Int2***: £641<br>**Conclusion**: "Overall, the costs of care for those receiving the assessment were no greater with NHS costs actually lower. The potential benefits in involving specialist clinicians in the assessment process include identifying previously undiagnosed conditions and enhancing care managers' decision making[; this] could be provided at a modest marginal cost."	✓
Markle-Reid 2006[572]	hmcr & mfar(w/med +slfm)<br>n = 120/144	hmcr & mfar<br>n = 122/144	Soc (inc. HSCS)	6	●				●	●	●<br>Out of pocket expenses	●<br>Number of days off work	Not specified but analysed as part of total costs	**Currency / Price year**: CAD$ (CAN $1 = USD $0.641, GBP £0.445 and EUR €0.717), (assumed) 2001-2<br>**Total costs:** No statistically significant difference between the two groups<br>**Conclusion**: "Home based nursing health promotion, proactively provided to frail older people with chronic health needs, enhances quality of life while not increasing the overall costs of health care. The results underscore the need to re-invest in nursing services for health promotion for older clients receiving home care."	✓
Walters 2017[626]	mfar(w/slfm)<br>n = 25/26[f]	ac<br>n = 24/25[g]	HSCS and Soc (inc. HSCS)[h]	6	●				●	●	●<br>Transport, privately paid home help, informal care, benefits received, social outings only		Training costs (staff time on training, oncosts, overheads), staff time on supervision, time on service delivery (appoint-ments, adminis-tration, travelling), consum-ables supplied to clients	**Currency / Price year**: GBP£, 2015-6<br>**Total costs** (mean per person):<br>(health services only) ***Int1***: £1650 (SE £908) (95% CI £-179 to £3478), ***Int2***: £2575 (£927) (95% CI £707 to £4445)<br>(care and support services only): ***Int1***: £1563, ***Int2***: £3632<br>**Experimental intervention costs** (mean, per person): £307<br>**Budget impact analysis**: If the NHS was to commission, and assuming delivered by a NHS band 6 staff member, savings are £907 per patient in secondary care and £16 in community care; equal a net saving to a NHS commissioner of £616 per patient, with greater number of patients reducing the per-patient cost.<br>If the local government was to commission, the total cost saving of home-based social care is £170 and a total	~

														additional cost of £297 for accommodation; resulting in a total additional cost to local government of £434 per patient.<br>**Conclusion**: "The intervention was delivered at modest cost. Although there were promising findings in terms of its potential for cost-effectiveness, this was a small study and it would be premature to recommend more widespread implementation, except as part of a larger-scale evaluation. The budget impact assessment provides preliminary information to Clinical Commissioning Groups and/or local authorities on the potential costs and benefits to their local budget should they wish to implement this service."	

approx., approximately; CAD$, Canadian dollar; CDI, Community Dependence Index; CE, cost-effectiveness; CEA curve, cost-effectiveness acceptability curve; CI, confidence interval ; CUA, cost-utility analysis; EUR€, Euro; GBP£, British pound; GDP, gross domestic product; GP, general practitioner; HSCS, health and social care system; HKD$, Hong Kong dollar; ICER, incremental cost-effectiveness ratio; n, number of participants analysed out of the number randomised; NICE, National Institute of Health and Clinical Excellence; NOK, Norwegian krone; QALY, quality-adjusted life year; SD, standard deviation; SE, standard error; society including healthcare services; USD$, US dollar; WHO, World Health Organization; WTP, willingness to pay

Intervention and control group abbreviations are a combination of the following:- ac: available care; ADL: activities of daily living training; aids: provision of aids and adaptions; cgn: cognitive training; comm: technology for communication and engagement; educ: health education; eng: engagement in meaningful activities; exrc: physical exercise; hmcr: formal homecare; hmnt: alternative medicine; med: medication review; mfa: multifactorial action; mfar: multifactorial action and follow-on routine review; mntr-mfa: monitoring, which may trigger multifactorial action; ntr: nutritional support; psyc: psychological therapy; rsk-mfa: risk screening, which may trigger multifactorial action; sst: social skills training; vchr: care voucher provision; wlfr: welfare rights advice; w/med: with medication review; w/slfm: with self-management.

a ● indicates the category was included in the study evaluation

b ✓ indicates that experimental intervention was clearly concluded as a more cost-effective, lower-cost alternative, or recommended by the study authors; ✗ indicates that experimental intervention was explicitly not recommended by the study authors; ~ indicates that no definite conclusion was drawn by the study authors.

c Mean difference = Intervention 1 (Int1) group value minus Intervention 2 (Int2) group value

d Study authors considered the treatment a success if a patient's MOS-20MH score increased by more than 10 points and the GARS-3 score declined no more than 4.5 points.

e CE plane regions: southeast quadrant represents less costs and more effects (superior), northeast quadrant represents higher costs and more effects, southwest quadrant represents less costs and less effects, northwest quadrant represents higher costs and less effect (inferior)

f Cost-effectiveness was evaluated with two outcomes which were not of interest of this review: ICER - COPM performance in daily life activities; ICER - COPM satisfaction with performance in daily life activities.

g Imputation used to replace missing values

h Two separate perspectives were used in the evaluation.

Table 108 - Economic evaluation analysis and principal findings reported for the medium-term time horizon

Study	Intervention 1 (Int1) *(Experimental intervention)*	Intervention 2 (Int2) *(comparator)*	Perspective	Time horizon (months)	Evaluation type[a]: Cost analysis	Cost-effectiveness analysis	Cost-utility analysis	Cost-benefit analysis	Cost items category[a]: Health sector costs	Other sector costs	Patient and family costs	Productivity impacts	Intervention cost items	Currency / Price year / Principal findings / Conclusion of study	Study conclusion[b]
Dorresteijn 2016[526]	ADL<br><br>n = 130/194	ac<br><br>n = 159/195	Soc (inc. HSCS)	12	●	●[c]	●		●		●<br>(nursing) home-care, formal and informal care, aids, and in-home modifications only		Materials used, salaries of the facilitators, costs of training sessions for the facilitators, etc.	**Currency / Price year**: EUR€, 2011<br>**Total costs** (mean per person): ***Int1***: €7890 (SD €6450), ***Int2***: €8094 (SD €7466)<br>**ICER** (per QALY): base case: €-9586 (dominant), healthcare perspective: €−14,018 (dominant), per-protocol: €−159,846 (dominant), without outliners: €−35,330 (dominant)<br>**CE plane**: (QALY base case) 57% in southeast, 38% northeast, 1% southwest, 3% northwest quadrants[d].<br>(Sensitivity analyses) Overall, the probability of the cost-effectiveness of AMB-Home increased if participants received five or more sessions compared to usual care (per-protocol), decreased when costs were taken only from a healthcare perspective, and without outliers was rather similar to the base case analyses.<br>**Conclusion**: "The programme is likely to be cost-effective, and therefore a useful addition to current geriatric care, particularly for those persons who are not able or willing to attend group programmes."	✓
Clark 1997[519]	eng & educ<br><br>n = 51/51	ac<br><br>n = 112/112	Soc (inc. HSCS)	15	●		●		●	●<br>Paid caregiver only	●<br>Unpaid caregiver only		Int1: Staff time spent on preparation, contacts, and travelling;<br><br>Int2: Staff time spent on preparation and contacts	**Currency / Price year**: USD$, 1995; annual discount rate of 3% for costs<br>**Total costs** (annualised mean per person): ***(15 months, post-treatment) Int1***: $4145 (SD $10,801), ***Int2***: $5218 (SD $9588)<br>**Intervention costs** (mean per person): ***Int1***: $548, ***Int2***: $68<br>**ICER** (per QALY)**:** $10,666 (95% CI $6,747 to $25,430)<br>**Conclusion**: "Preventive OT demonstrated cost-effectiveness in conjunction with a trend toward decreased medical expenditures."<br>**Notes:** Costs during intervention phase estimated at 9 months	✓

Cameron 2013[515]	exrc & mfar(w/med+ slfm)<br><br>n = 119/120	ac<br><br>n = 119/121	HSCS	12	●	●[e]	●	●[e]	●	●<br>Also transport			Staff time spent on assessments and intervention delivery, materials and equipment in intervention delivery, aids and adaptions	**Currency / Price year**: AUD$, 2011<br>**Total costs** (mean per person): ***Int1*** $25,030 (SD $29,827), ***Int2*** $22,885 (SD $32354);<br>***mean difference***[f]: $2145 (95% CI $-5698 to $10,221)<br>**Experimental intervention costs** (mean per person): $1528.52<br>**ICER:** (per QALY) taking uncertainty into account, the bootstrapped replicates indicated that 10.8% probability of being cost saving across the entire participant population, 17.8% probability of saving in the very frail subgroup, and 8.2% probability in the frail subgroup<br>**Conclusion**: "For frail older people residing in the community, a 12-month multifactorial intervention provided good value for money, particularly for the very frail, where it has a high probability of being cost saving as well as effective."[e]	✓[e]
Brettschneider 2015[514]	mfar(w/med)<br><br>n = 133/150	ac<br><br>n = 145/155	Soc (inc. HSCS)	18	●		●	●	●<br>Also medical devices, dentures	●	●<br>Informal care, transport, in-home modifications only		Staff training cost, staff time spent on assessments, case conferences and home visits, participant's travel costs	**Currency / Price year**: EUR€, 2008; reported no discounting<br>**Total costs** (mean per person): ***Int1***: €20,195 (SD €21,689), ***Int2***: €21,028 (SD €24,384); ***adjusted mean difference***[f]: €4400.52 (SE €3019.61)<br>**Experimental intervention costs** (mean per person): €73 (SD €22)<br>**CEA curve**: 15% probability of an ICER <€50,000/QALY for preventive home visits.<br>At a WTP of €0/QALY the probability of cost-effectiveness of preventive home visits was 7%, while at a WTP of €250,000/QALY the probability was 39%.<br>**Conclusions**: "The evaluated preventive home visits programme is unlikely to be cost-effective."	✗
Hogg 2009[550]	mfar(w/med)<br><br>n = 74/120	ac<br><br>n = 78/121	HSCS[g]	15	●	●[h]		●	●<br>Also personal service support				Staff time spent on intervention delivery and adminis-tration, medical supplies, overheads	**Currency / Price year**: CAD$, (assumed) 2004-6<br>**Total costs** (mean, per person):<br>***Int1***: $12,923, ***Int2***: $9222;<br>***mean difference***[f]: $3701 (95% CI $385 to $7024)<br>**Experimental intervention costs** (mean, per person): $3802<br>**Conclusion**: "By any of the metrics used, the APTCare intervention was not cost-effective, at least not in a population for which baseline quality of care was high."	✗
Suijker 2016[605]	mfar(w/med)<br><br>n = 1209/1209[i]	ac<br><br>n = 1074/1074[i]	HSCS	12	●	●	●		●	●			Staff time spent on training, postal screening, visits, and planning treatment plans	**Currency / Price year**: EUR€, 2010 prices adjusted for 2016<br>**Total costs** (mean per person):<br>***Int1***: €6518 (SE €472), ***Int2***: €5214 (SE €338);<br>***mean difference***[f]: €1457 (95% CI €572 to €2537)<br>**Experimental intervention costs** (mean per person): €168	✗

														**ICERs**: (per 1 point of modified Katz-ADL index) €21,884<br>(per QALY) €287,879<br>**CE plane**: 79% of the modified Katz-ADL cost-effect pairs and majority of the QALY cost-effect pairs in the northeast quadrant[d]<br>**CEA curve**: maximum probability of the intervention being cost-effective was 14% at a WTP of €50,000 per one point improvement on the modified Katz-ADL index score, and 4% at a WTP of €50,000/QALY gained; less than 1% probability of cost-effectiveness at a WTP of €0 per modified Katz-ADL point or QALY.<br>**Conclusion**: “The current intervention was not cost-effective compared to usual care to prevent or postpone new disabilities over a one-year period. Based on these findings, implementation of the evaluated multifactorial nurse-led care model is not to be recommended.”	
Gitlin 2006[539]	ADL & aids & exrc<br><br>n = 160/160	ac<br><br>n = 159/159	HSCS[j]	12	●	●		●		●<br>Experimental intervention costs only			Staff time spent on training and with participants, materials, travel mileage, home modifications	**Currency / Price year**: USD$, 2003 (adjusted to 2010 values); reported not discounted;<br>Model 1 (base case) reports on estimated costs of delivering ABLE in a home care agency. Model 2 (base case + 10%) accounts for a potential variation in the cost of delivering ABLE in a real world setting.<br>**Experimental intervention costs** (mean per person): ***base case***: $942, ***base case + 10% model***: $1036<br>**ICER** (cost per one additional year of life): ***base case model***: $13,179, ***base case +10% model***: $14,800<br>**CEA curve**: Probability of the intervention being cost-effective is greater than 50% of the time as long as a purchaser is willing to pay more than $13,000 for one additional year of life under the base case model; or $14,800 under the base case +10% model.<br>**Conclusion**: “This economic evaluation suggests that investment in this program may be worthwhile depending on one’s willingness to pay. However, confidence intervals varied widely due to small effect in reducing mortality.”	~
Kukkonen-Harjula 2017[561]	ADL & ntr & exrc<br><br>n = 150/150	ac<br><br>n = 149/149	HSCS	12	●		●		●	●			Physio-therapist visits	**Currency / Price year**: EUR€, 2018 (service costs valued at 2011 and corrected for inflation)<br>**Total costs** (mean, pyrs):<br>***(12-month intervention period) Int1***: €33,839 (SE €2167), ***Int2***: €21,151 (SE €2185);<br>***mean ratio***: 1.60 (95% CI 1.23 to 1.98)<br>**CE plane**: For the first 12 months, for costs and QALYs, all participants lay in the northeast quadrant[b], implying that the intervention was more effective but more costly than usual care.	~

														**Conclusion**: "The exercise investment was costly, but the costs were gained back in decreased utilization of health care and social services in the exercise frail subgroup over 24 months."	
Blom 2016[510]	mfa-(w/med +slfm)<br><br>n = 288/288[i]	ac<br><br>n = 1091/1091[i]	Soc (inc. HCS)	12	●		●		●	●	●<br>Informal care only		Staff training costs (course development, materials, time), staff time spent on assessments and formulating care plans, materials, participants' time invested in the intervention	**Currency / Price year**: EUR€, 2013; reported no discounting<br>**Total costs** (mean per person):<br>***Int1***: €18761, ***Int2***: €20066;<br>***mean difference***[f]: €-1305 (95% CI €-16,349 to €13,744)<br>**Experimental intervention costs**: €236-370 per care plan (mean cost for a GP practice conducting 25 or 10 plans respectively, cost is lower for more plans in a practice).<br>**For reasonable WTP**: values above €10,000 per QALY, both policies are about 50% likely to be preferred.<br>**Conclusion**: "The care plan costs were low compared to (the variability of) the total costs during the 1-year follow-up period, and were not significantly different between groups. Due to the uncertainty in WTP, the economic preference for care is undecided."	~
Parsons M 2017[586]	hmcr & ADL & mfar(w/slfm)<br><br>n = 15/56	hmcr & mfa-<br><br>n = 12/57	HSCS	12	●	●		●	●	●<br>Also social worker, volunteer services, modifications, transport, carer support	●<br>Costs to the older people (items not specified)		Not specified but analysed as part of total costs	**Currency / Price year**: NZD$ 2006<br>**Total costs** (mean per person): ***Int1***: $46,256, ***Int2***: $32,413<br>***mean difference***[f]: $13,842.66<br>**ICERs** (mean per person):<br>(each day residential care avoided): $880.57<br>(each day deceased avoided): $392.27<br>(each day in community gained): $271.26<br>**Sensitivity analysis**: Results can be quite sensitive to changes in the average resource use and changes in living and survival status outcomes.<br>**Conclusion**: "While the cost of the initiative was more than the cost for usual care, the initiative had the result of increasing the amount of time spent in the community relative to usual care over a 12-month period, by decreasing the time spent in residential care and the time spent deceased.<br>"Community FIRST may appear much more expensive for the outcome it achieves (among the three initiatives as part of the ASPIRE project), but this is because it faced greater challenges with its sampled participants."	~
Parsons M 2012[587]	hmcr & mfar<br><br>n = 116/169	hmcr & mfa-<br><br>n = 117/182	HSCS	12	●	●		●	●	●<br>Also social worker, volunteer services, modifications, transport, carer support	●<br>Costs to the older people (items not specified)		Not specified but analysed as part of total costs	**Currency / Price year**: NZD$, 2006<br>**Total costs** (mean per person): ***Int1***: $13,936, ***Int2***: $13,779;<br>***mean differenc***[f]: $157.49<br>**ICERs** (mean per person):<br>(each day residential care avoided): $22.84<br>(each day deceased avoided): $190.74	~

														(each day in community gained): $20.13<br>**Sensitivity analysis**: Results can be quite sensitive to changes in the average resource use and changes in living and survival status outcomes.<br>**Conclusion**: Over 12 months, “the cost of the initiative was more than the cost for usual care, [but it increased time remaining at home comparatively], by [reducing] time spent in residential care [or] deceased.”<br>“Our base case results suggest that COSE costs an additional $20 per person over a 12-month period for each extra day spent in the community relative to usual care.”	
Leveille 1998[564]	educ & exrc & mfar(w/med+ slfm)<br><br>n = 100/101	ac<br><br>n = 100/100	HSCS	12	●				●<br>Hospital in-patient charges only	●<br>Experimental intervention costs only			Salaries of intervention team	**Currency / Price year** (assumed): USD$, mid-1990s<br>**Hospital charges** (mean per person): ***mean difference***[f]: savings of approx. $1200 in Int1<br>**Experimental intervention costs** (mean per person annually): approx. £300<br>**Conclusion**: “The estimated cost savings, based on the absolute reduction in the number of inpatient days by intervention participants, were substantial. […] These findings in regard to inpatient costs alone are very encouraging and suggest a sizeable cost benefit to healthcare insurers from this approach to disability prevention.”	✓
Bleijenberg el al. 2016[509]	UPRIM screening<br><br>rsk-mfa-<br><br>n = 790/790<br><br>UPRIM+U-CARE<br><br>rsk-mfa-<br><br>n = 1446/1446	ac<br><br>n = 856/856	HSCS	12	●				●<br>Items not specified				Not specified but analysed as part of total costs	**Currency / Price year**: EUR€, (around) 2010-2<br>**Total costs** (mean per person):<br>***Int1: (U-PRIM)*** €6651, (***U-PRIM+U-CARE)*** €6825, ***Int2***: €7601.<br>**Conclusions**: “U-PRIM and U-PRIM+U-CARE resulted in better preservation of daily functioning in older patients and has a high probability of being cost-effective compared with usual care.”	✓
Mann WC 1999[571]	hmcr & aids<br><br>n = 52/52	hmcr<br><br>n = 49/52	HSCS	18	●				●	●<br>Also case manager visits			AT and EI only	**Currency / Price year**: (assumed) USD$, mid-1990s<br>**Total costs** (mean per person):<br>***Int1***: $14,172 (SD $13,761), ***Int2***: $31,610 (SD $42,239)<br>**Intervention costs on AT-EIs** (mean per person):<br>***Int1***: $2620, ***Int2***: $443<br>**Conclusion**: “The frail elderly persons in this trial experienced functional decline over time. Results indicate rate of decline can be slowed, and	✓

														institutional and certain in-home personnel costs reduced through a systematic approach to providing AT and EIs.”	
Bernabei 1998[508]	hmcr & mfar(w/med)<br><br>n = 99/100	hmcr<br><br>n = 100/100	HSCS	12	●				●	●			Salaries of intervention team	**Currency / Price year**: GBP£ and USD$, (assumed) 1995<br>**Total costs**: savings of £1125 ($1800) per year of follow up in Int1, 23% less than Int2, mainly from reductions in nursing home and hospital expenses<br>**Conclusion**: “Integrated social and medical care with case management programmes may provide a cost-effective approach to reduce admission to institutions and functional decline in older people living in the community.”	✓
Ploeg 2010[590]	educ & mfar(w/med)<br><br>n = 350/361	ac<br><br>n = 343/358	HSCS	12	●				●	●			Not specified but analysed as part of total costs	**Currency / Price year**: CAD$, (assumed 2004-6)<br>**Total costs** (mean per person):<br>***Int1***: $7779 (SD $7980), ***Int2***: $8096 (SD $9582); ***mean difference***[f]: $-165 (£107; €118; USD$162) (95% CI $-16545 to $16214)<br>**Conclusion**: “A preventive primary care outreach intervention for older Canadian adults at risk of functional decline had no effect on QALYs, costs of health and social services, functional status, self-rated health, or mortality.<br>“The results of this study do not support adoption of this preventive primary care intervention for this target population of high-risk older adults.”	✗
Hay 1998[546]	mfa-<br><br>n = 75/209	Usual care (CG1: no baseline; CG2: assessed at baseline)<br><br>CG1: ac<br><br>CG2: ac<br><br>CG1<br><br>n = 103/207<br><br>CG2<br><br>n = 86/203	Soc (inc. HSCS)	12	●				●	●	●<br>Out-of-pocket expenses only	●<br>Loss of income or work days only	Not specified, not included in total costs analysis.	**Currency / Price year** (assumed): CAD$, 1991-5<br>**Total costs** (mean per person annually):<br>***(during treatment) Int1***: $4001, ***CG1***: $1555, ***CG2***: $2587<br>**Conclusion**: “While the study provided an opportunity for these interventions, there was no demonstrable benefit in terms of cost or health status. There were no significant differences in health system use costs, although the experimental group showed higher use the year they were being treated and a marked decrease in the second year. Differences in hospitalization rates account for this variation.”	✗

Counsell 2007[522]	educ & mfar(w/med+slfm)<br><br>n = 474/474	ac<br><br>n = 477/477	HSCS	12	●				●				Salaries and benefits for personnel, mileage reimburse-ment, pager and cellphone costs, home visit bags, and office supplies	**Currency / Price year**: USD$, (assumed 2002-4)<br>**Total costs** (mean per person):<br>***(12 months) Int1***: $7917 (SD $10,457), ***Int2***: $6163 (SD $10,044)<br>**Experimental intervention costs** (mean per person annually): ***all***: $1260, ***high risk***: $1432, ***low risk***: $1207<br>**Conclusion**: "In patients at high risk of hospitalization, the GRACE intervention is cost neutral from the healthcare delivery system perspective. A cost-effectiveness analysis is needed to guide decisions about implementation in low-risk patients."	~
Newcomer 2004[583]	educ & mfar(w/med)<br><br>n = 1523/1537	ac<br><br>n = 1532/154[illegible]	HSCS[k]	12	●				●<br>Hospital in-patient charges only				Not specified	**Currency / Price year** (assumed): USD$, 2001-3<br>**Hospital charges** (mean per person monthly):<br>***Int1***: $2002 (SD $9895), ***Int2***: $2102 (SD $15,227);<br>***mean change***[e] (increase from baseline): ***Int1***: $1110 (SD $10,300), ***Int2***: $1071 (SD $15,597).<br>**Conclusion**: "Regardless of the approach taken to quantify or standardize service use or expenditures, the unadjusted findings were consistent: There was no statistically significant treatment effect evident among the study outcomes."	~

approx., approximately; AT and EI, assistive technology and home environmental interventions; CAD$, Canadian dollar; CE, cost-effectiveness; CEA curve, cost-effectiveness acceptability curve; CI , confidence interval; EUR€, Euro; GBP£, British pound; HSCS, health and social care system; ICER, incremental cost-effectiveness ratio; n= number of participants analysed out of the number randomised; NZD$, New Zealand dollar; pyrs, per person-years; QALY, quality-adjusted life year; SD, standard deviation; SE, standard error; Soc (inc. HSCS), societal perspective including health and social care system; USD$, US dollar; WTP, willingness to pay

Intervention and control group abbreviations are a combination of the following:- ac: available care; ADL: activities of daily living training; aids: provision of aids and adaptions; cgn: cognitive training; comm: technology for communication and engagement; educ: health education; eng: engagement in meaningful activities; exrc: physical exercise; hmcr: formal homecare; hmnt: alternative medicine; med: medication review; mfa: multifactorial action; mfar: multifactorial action and follow-on routine review; mntr-mfa: monitoring, which may trigger multifactorial action; ntr: nutritional support; psyc: psychological therapy; rsk-mfa: risk screening, which may trigger multifactorial action; sst: social skills training; vchr: care voucher provision; wlfr: welfare rights advice; w/med: with medication review; w/slfm: with self-management.

a ● indicates the category was included in the study evaluation

b ✓ indicates that experimental intervention was clearly concluded as a more cost-effective, lower-cost alternative, or recommended by the study authors; ✗ indicates that experimental intervention was explicitly not recommended by the study authors; ~ indicates that no definite conclusion was drawn by the study authors.

c Cost-effectiveness was evaluated with Falls Efficacy Scale-International (FES-I) which is not an outcome of interest.

d CE plane regions: southeast quadrant represents less costs and more effects (superior), northeast quadrant represents higher costs and more effects, southwest quadrant represents less costs and less effects, northwest quadrant represents higher costs and less effect (inferior)

e Cost-effectiveness was evaluated with extra number of patients experiencing transition out of frailty which is not an outcome of interest.

f Mean difference = Intervention 1 (Int1) group value minus Intervention 2 (Int2) group value

g Specific payer's perspective mentioned: From the perspective the provincial Ministry of Health

h Cost-effectiveness was evaluated with quality of care which is not an outcome of interest.

i Imputation used to replace missing values

j Specific payer’s perspective mentioned: From the perspective of a homecare agency

k Specific payer’s perspective mentioned: From the perspective of Medicare, USA

Table 109 - Economic evaluation analysis and principal findings reported for the long-term time horizon

Study	Intervention 1 (Int1) *(Experimental intervention)*	Intervention 2 (Int2) *(comparator)*	Perspective	Time horizon (months)	Evaluation type[a]				Cost items category[a]				Intervention cost item	Currency / Price year / Principal findings / Conclusion of study	Study conclusion[b]
					Cost analysis	Cost-effectiveness analysis	Cost-utility analysis	Cost-benefit analysis	Health sector costs	Other sector costs	Patient and family costs	Productivity impacts			
Liimatta 2019[567]	exrc & mfa-(w/med)<br><br>n = 211/211[c]	ac<br><br>n = 211/211[c]	HSCS	24	●		●		●	●			Unit costs of home visits	**Currency / Price year**: EUR€, 2013-2014 (service costs valued at 2011 and corrected for inflation)<br>**Total costs** (mean, pyrs):<br>***Int1***: €7310 (SE €849), ***Int2***: €8277 (SE €1089); ***mean difference***[d]: €-967 (95% CI €-3766 to €1633); ***mean ratio***: 0.84 (95% CI 0.55 to 1.13)<br>**Experimental intervention costs** (mean, per person): €382<br>**CE plane**: 60% ICERs per QALY lie in the dominant (southeast) quadrant[e]<br>**Conclusion**: "The intervention appeared to have positive effects on health-related quality of life without accruing additional costs."	✓
Metzelthin 2013[576]	educ & mfar(w/med+slfm)<br><br>n = 103/193	ac<br><br>n = 91/153	Soc (inc. HSCS)	24	●	●	●		●	●	●<br>Informal care, aids and in-home modifications only		Intervention materials, training activities, postal screening, and staff time spent on home visit assessments, treatment plans, delivering interventions	**Currency / Price year**: EUR€, 2010<br>**Total costs** (mean per person): ***Int1***: €26503 (SD €27273), ***Int2***: €20,550 (SD €18891); ***mean difference***[d]: €5953 (95% CI €-633 to €12538)<br>**Experimental intervention costs** (mean per person): €728<br>**ICERs** (per GARS score): €1920,<br>(per QALY UK tariff): €150616, without intervention costs: €132195, QALY Dutch tariff: €285428<br>**CE plane**: (QALY UK tariff) 2% in southeast, 19% northeast, 2% southwest, 77% northwest quadrants[e].<br>(GARS): 1% in southeast, 2% northeast, 3% southwest, 95% northwest quadrants[e].<br>**Sensitivity analyses:** did not reveal other results<br>**Conclusion**: "The intervention under study led to an increase in healthcare utilisation and related costs without providing any beneficial effects. This study adds to the scarce amount of evidence	✗

														regarding cost-effectiveness of proactive primary care in community-dwelling frail older people.”	
Bouman 2008[513]	mfar(w/med)<br><br>n =139/160 (analysed for CE; 160 analysed for costs)	ac<br><br>n =154/170 (analysed for CE; 170 analysed for costs)	HSCS	24	●	●			●	●<br>Also aids, in-home modifications			Staff salaries, staff travel costs, staff training activities	**Currency / Price year**: EUR€, base year 2003, or otherwise discounted at 4%<br>**Total cost** (mean per person):<br>***Int1***: €15679, ***Int2***: €15229;<br>***mean difference***[d]: €450 (95% CI €-3780 to €4680)<br>**Experimental intervention costs** (mean per person): €753<br>**ICER** (per self-rated health score): bootstrap analysis showed a 10% chance that the intervention was cost-effective<br>**Conclusion:** “The home visiting program did not appear to have any effect on the health care use of older people with poor health and had a low chance of being cost-effective. [… T]hese visits are probably not beneficial for such persons [in this] or comparable settings …”	✗
Howel 2019[552]	wlfr<br><br>n = 381/381	ac<br><br>n = 374/374	HSCS	24	●		●	●		●<br>Welfare rights advice services only			Time spent on home visit, telephone calls, letter/email writing, administration, and travel costs	**Currency / Price year**: GBP£, 2013-4 discounted at 1.5% for second year<br>**Experimental intervention costs** (mean per person): £43.76; 38% were travel costs<br>**ICER** (per QALY): £1914<br>**CEA curve**: 63% probability that the intervention would be cost-effective, should society be willing to pay £20000/QALY gained. These results were robust to changes in the discount rate and higher costs associated with the delivery of the intervention.<br>**Conclusion**: “We found no effects on health outcomes; fewer participants than anticipated received additional benefit entitlements, and participants were more affluent than expected. Our findings do not support delivery of domiciliary welfare rights advice to achieve the health outcomes assessed in this population. However, better intervention targeting may reveal worthwhile health impacts.”	✗
Kukkonen-Harjula 2017[561]	ADL & ntr & exrc<br><br>n = 150/150	ac<br><br>n = 149/149	HSCS	24	●		●		●	●			Physio-therapist visits (specific cost items not provided)	**Currency / Price year**: EUR€, 2018 (service costs valued at 2011 and corrected for inflation)<br>**Total costs** (mean, pyrs):<br>***(0-24 months including post-intervention) Int1***: €23961 (SE €2198), ***Int2***: € 29428 (SE €2282);<br>***mean ratio***: 1.23 (95% CI 0.95 to 1.50)<br>**Conclusion**: “The exercise investment was costly, but the costs were gained back in decreased	~

														utilization of health care and social services in the exercise frail subgroup over 24 months."	
Vass 2005[622]	mfar(w/med)<br><br>n = 2092/2104[c]	mfar<br><br>n = 1942/1956[c]	HSCS	36	●	●		●	●<br>Included all resources used recorded in routine healthcare databases, which include dental care, aids and applications	●<br>Included all resources used recorded in routine social services databases	●<br>Patient co-payments for care and prescription only		Staff training programme, GP services, staff time on home visits, transport, administration, breaks, meetings.	**Currency / Price year**: EUR€ (€1=7.46 Danish crowns), 2001-2002 prices converted to 2005 values; reported as undiscounted, and in present values using a 3% and 6% discount rate a year.<br>**Total costs** (mean per person):<br>(75-year-old) ***Int1***: €12899 (SE €605.36), ***Int2***: €13778 (SE €587.94);<br>***mean difference***[d]: €-879 (95% CI €−2534 to €776); discounted 3%: €-855 (95% CI €-2455 to €744)<br>(80-year-old) ***Int1***: €17773 (SE €1332.17), ***Int2***: €17059 (SE €1180.97);<br>***mean difference***[d]: €714 (95% CI €−2779 to 4207); discounted 3%: €694 (95% CI €−2684 to 4071)<br>**ICERs** (per active life-year): (75-year-old) mean total costs were the same in the groups as well as the mean number of active life-years. Thus it was not relevant to calculate ICERs.<br>(80-year-old, costs and active life-years discounted 3%) €3522 per active life year gained; **Sensitivity analyses**: €2906 to €6294 per active life-year gained among the 80-year-olds<br>**WTP**: Probability that Int1 intervention being more cost effective than Int2:<br>(75-year-old) did not increase 86% for ceiling ratios up to €60000 per active life-year gained.<br>(80-year-old) increased to 93% if a decision maker is willing to pay at least €20000 per active life-year gained, and to 98% if they are willing to pay at least €27000 per active life-year gained.<br>**Conclusion**: "Neither the differences in the total costs nor […] the effectiveness measure were statistically significant. The estimates […] fell into a range where no definite conclusions can be drawn regarding cost-effectiveness. [It] depends on the decision makers' […] willingness to pay for an active life-year in elderly persons."	~
Lewin 2013[565]	hmcr & educ & mfar<br><br>n = 375/375	hmcr<br><br>n = 375/375	HSCS	24	●				●	●			Not specified but analysed as part of total costs	**Currency / Price year**: AUD$, (assumed) 2007-8<br>**Total costs** (mean per person): ***Int1***: $19888, ***Int2***: $22757<br>**Conclusion**: "Given the projected increase in numbers of older people in Australia over the next 40 years, the incorporation of intensive restorative services into the Gateway proposed for the reformed Australian aged care system (Commonwealth of Australia 2012) could result in very substantial savings at a whole of population level. Careful targeting of older people to maximise the cost-effectiveness of restorative interventions warrants further investigation."	✓

Hay 1998[546]	mfa-<br><br>n = 75/209	Usual care (CG1: no baseline; CG2: assessed at baseline)<br><br>CG1: ac<br><br>CG2: ac<br><br>CG1<br><br>n = 103/207<br><br>CG2<br><br>n = 86/203	Soc (inc. HSCS)	24	●				●	●	●<br>Out-of-pocket expenses only	●<br>Loss of income or work days only	Not specified, not included in total costs analysis.	**Currency / Price year** (assumed): CAD$, 1991-5<br>**Total costs** (mean per person annually):<br>***(post-treatment) Int1***: $1600, ***CG1***: $1041, ***CG2***: $2458<br>**Conclusion**: "While the study provided an opportunity for these interventions, there was no demonstrable benefit in terms of cost or health status. There were no significant differences in health system use costs, although the experimental group showed higher use the year they were being treated and a marked decrease in the second year. Differences in hospitalization rates account for this variation."	χ
van Rossum 1993[621]	mfar<br><br>n = 292/292	ac<br><br>n = 288/288	HSCS	36	●				●	●			Staff time spent on home visits, travelling, preparing the visits, administration	**Currency / Price year**: NLGƒ (ƒ1 = approx. GBP£0.29 and USD$0.51), (assumed) 1988-92<br>**Total costs** (mean per person):<br>***Int1***: ƒ20080, ***Int2***: ƒ19321; ***mean difference***[c]: +4%<br>**Experimental intervention costs** (total): ƒ393981<br>**Conclusion**: "From a financial point of view, the visits were not beneficial: apart from the reduced hospital costs, the 'gains' in favour of the intervention group were only marginal (home nursing care and nursing home). The increased costs in the intervention group with respect to most community services and homes for the elderly balanced the reduction of hospital costs. Preventive home visits are not beneficial for the general population of elderly people living at home but might be effective when restricted to subjects with poor health."	χ
Counsell 2007[522]	educ & mfar(w/med+ slfm)<br><br>n = 436/474	ac<br><br>n = 440/477	HSCS	24 & 36	●				●				Salaries and benefits for personnel, mileage reimburse-ment, pager and cellphone costs, home visit bags, and office supplies	**Currency / Price year**: USD$, (assumed 2002-4)<br>**Total costs** (mean per person):<br>***(24 months, cumulated) Int1***: $14348 (SD $15008), high risk: $17713 (SD $16776), low risk: $13307 (SD $14286), ***Int2***: $11834 (SD $15567), high risk: $18776 (SD $19472), low risk: $9654 (SD $13429)<br>***(24-36 months, post-intervention) Int1***: $5045 (SD $9684), high risk: $5088 (SD $7481), low risk: $5032 (SD $10258), ***Int2***: $4732 (SD $10012), high risk: $6575 (SD $9030), low risk: $4217 (SD $10222)<br>**Conclusion**: "In patients at high risk of hospitalization, the GRACE intervention is cost	~

														neutral from the healthcare delivery system perspective. A cost-effectiveness analysis is needed to guide decisions about implementation in low-risk patients.”	
Coleman 1999[521]	educ & mfar(w/med+ slfm)<br><br>n = 96/96	ac<br><br>n = 73/73	HSCS	24	●				●				Not specified but analysed as part of total costs	**Currency / Price year** (assumed): USD$, mid-1990s<br>**Total costs** (mean per person annually):<br>***Int1***: $9535, ***Int2***: $10116<br>**Conclusion**: “Costs of medical care including frequency of hospitalization, hospital days, emergency and ambulatory visits, and total costs of care were not significantly different between intervention and control groups.”	~
Stuck 2000[603]	mfar(w/med)<br><br>n = 775/791 from both groups	ac	HSCS	36	●				●	●			In-Home visits	**Currency / Price year**: SFr (SFr 1 = approx. USD$0.60), 1995<br>**Total costs** (per person annually):<br>***mean difference***: SFr 1500 (USD$900) more in Int1 than Int2<br>**Subgroup analysis**: Despite the similar health status of subjects, fewer health problems in Int1 participants were identified by 1 (nurse c) of 3 nurses. Among low-risk subjects visited by the 2 nurses (ZIP codes A & B), the PHVs resulted in net cost savings in the third year (SFr 2336 (USD$1403) per person per year), but not among those visited by nurse C.<br>**Experimental intervention costs** (mean per person): SFr 460 (USD$276)<br>**Conclusion**: “In the subgroup with favourable outcomes (i.e., among low-risk subjects in ZIP codes A and B), [… t]he program resulted in additional costs [near the start] of the intervention, but in the third year, the additional […] costs […] were more than offset by savings in nursing home costs…”	~
Kono 2012[560]	mfar	mfar	HSCS	24	●				●	●			Preventive home visits (specific cost items not provided)	**Currency / Price year**: JP¥ (1 USD$ = ¥104.5 in 2008, = ¥94.6 in 2009)<br>**Total costs:** (mean per person):	~

	n = 161/161	n = 162/162								Also visiting nursing care, aids and home modifications				***Int1***: ¥2016606 (SE ¥161432; approx. USD$20166, SE $1,614), ***Int2***: ¥2287450 (SE ¥200535; approx. USD$22875, SE $2005)<br>**Experimental intervention costs** (mean per person per year): ¥5000 (approx. USD$50)<br>**Conclusion**: "The total LTC costs over 2 years in the intervention group were higher than in the control group (non-significant), and the intervention group utilised significantly more community and institutional LTC services than the control group over the period 7 months to 15 months after the intervention started.<br>"The present second analysis of randomized controlled trial showed that a preventive home visit program can reduce health care costs, primarily from reduced hospitalizations, in addition to providing other major benefits.<br>"The results suggest that a preventive home visit program might be ineffective on functional and psychosocial status among ambulatory frail elders overall, although it might significantly improve ADLs, IADLs and depression for those with ADL dependency."	
Kono 2016[558]	mfar(w/med)<br><br>n = 149/179	mfar<br><br>n = 157/181	HSCS	36	●					●<br>Also visiting nursing care			Not specified but analysed as part of total costs	**Currency / Price year**: credit (1 credit = JP¥ 10.0 - 10.70 at 2014)<br>**Total costs** (mean per person):<br>***Int1***: 3507 (SD 5400) credits, ***Int2***: 3562 (SD 5066) credits<br>**Conclusion**: "No statistically significant differences in total LTC service costs per person over 36 months between groups were obtained.<br>"We conclude that our PHV program with rigorous recommendations, based on the systematic structured assessment of care-needs, could be beneficially applied in clinical practice for the prevention of functional decline among ambulatory frail elderly people living at home."	~

approx., approximately; CAD$, Canadian dollar; CE, cost-effectiveness; CEA curve, cost-effectiveness acceptability curve; CI, confidence interval; CUA, cost-utility analysis; EUR€, Euro; GBP£, British pound; GP, general practitioner; HSCS, health and social care system; ICER, incremental cost-effectiveness ratio; n, number of participants analysed out of the number randomised; JP¥, Japanese yen; LTC, long-term Care; PHV, preventive home visit; pyrs, per person-years; QALY, quality-adjusted life year; SD, standard deviation; SE, standard error; SFr, Swiss francs; Soc, societal/ society; Soc (inc. HSCS), societal perspective including health and social care system; USD$, US dollar; WTP, willingness to pay

Intervention and control group abbreviations are a combination of the following:- ac: available care; ADL: activities of daily living training; aids: provision of aids and adaptions; cgn: cognitive training; comm: technology for communication and engagement; educ: health education; eng: engagement in meaningful activities; exrc: physical exercise; hmcr: formal homecare; hmnt: alternative medicine; med: medication review; mfa: multifactorial action; mfar: multifactorial action and follow-on routine review; mntr-mfa: monitoring, which may trigger multifactorial action; ntr: nutritional support; psyc: psychological therapy; rsk-mfa: risk screening, which may trigger multifactorial action; sst: social skills training; vchr: care voucher provision; wlfr: welfare rights advice; w/med: with medication review; w/slfm: with self-management.

a ● indicates the category was included in the study evaluation

b ✓ indicates that experimental intervention was clearly concluded as a more cost-effective, lower-cost alternative, or recommended by the study authors; ✗ indicates that experimental intervention was explicitly not recommended by the study authors; ~ indicates that no definite conclusion was drawn by the study authors.

c Imputation used to replace missing values

d Mean difference = Intervention 1 (Int1) group value minus Intervention 2 (Int2) group value

e CE plane regions: southeast quadrant represents less costs and more effects (superior), northeast quadrant represents higher costs and more effects, southwest quadrant represents less costs and less effects, northwest quadrant represents higher costs and less effect (inferior)

Appendix 13. Narrative and additional outcomes

Comparisons of homecare services usage were reported by sixteen studies (Table 110). Among results for which there were no serious concerns about risk of bias, compared with available care there was lower usage of homecare with three complex interventions and higher usage of homecare with one complex intervention. One other study with no serious concerns had ambivalent results. Results for two further studies were ambivalent (serious concerns). We had serious concerns about the risk of bias in results from eight studies and very serious concerns about the risk of bias in two studies.

For our additional outcomes there was little evidence of any effect on self-reported health status (appendix 11.7), only low certainty beneficial findings regarding depression (appendix 11.8), very little evidence regarding loneliness (Table 111), and more complex interventions were associated with less falling than more falling (12 studies vs 4 studies; Table 112). For mortality there was a large network of 65 studies (n = 38,351) and 41 intervention groups. There was low-certainty evidence of reductions for two, and increases for five, intervention groups (appendix 11.9).

Table 110 - Homecare usage results

Study	Intervention 1	Intervention 2	Outcome measure	Timepoint	Group 1 result	Group 2 result	Comparison	RoB assessment
Bouman 2008[513]	mfar(w/med)	ac	Homecare - personal care only (hours)	24 months	n=160; mean (SD): 42.06 (124.25); range: 0 to 907	n=170; mean (SD): 34.09 (109.12); range: 0 to 1130		No serious concerns
Bouman 2008[513]	mfar(w/med)	ac	Homecare - domestic care only (hours)	24 months	n=160; mean (SD): 86.65 (143.99); range: 0 to 627	n=170; mean (SD): 81.79 (138.47)		No serious concerns
King 2012[557]	hmcr & ADL & mfar(w/slfm)	hmcr	Homecare (visits per month)	7 months	n=87; mean: 5.8	n=85; mean: 6.3	P = 0.4	No serious concerns
King 2012[557]	hmcr & ADL & mfar(w/slfm)	hmcr	Homecare (hours per visit)	7 months	n=87; mean: 1.5	n=85; mean: 1.4	P = 0.2	No serious concerns
Kukkonen-Harjula 2017[561]	ADL & ntr & exrc	ac	Homecare (visits/ per person-years)	12 months	n=150; mean ± SE: 117.2 ± 19.3	n=149; mean ± SE: 160.5 ± 24.8	IRR: 0.73 (95% CI 0.47 to 1.14)	No serious concerns
Kukkonen-Harjula 2017[561]	ADL & ntr & exrc	ac	Homecare (visits/ per person-years)	24 months	n=150; mean ± SE: 141.2 ± 22.6	n=149; mean ± SE: 185.2 ± 27.1	IRR: 0.76 (95% CI 0.5 to 1.17)	No serious concerns
Liimatta 2019[567]	exrc & mfa-(w/med)	ac	Homecare (visits/ per person-years)	24 months	n=211; mean ± SE: 6.73 ± 1.93	n=211; mean ± SE: 11.81 ± 4.26		No serious concerns
von Bonsdorff 2008[624]	exrc	ac	Homecare (pts)	2 years	n=290; 15 persons (5.2%)	n=274; 26 persons (9.5%)	HR: 0.51 (95% CI 0.27 to 0.97)	No serious concerns
Bernabei 1998[508]	hmcr & mfar(w/med)	hmcr	Homecare (hours/ person/ year)	12 months	n=99; mean (SD): 120 (20)<br>n assumed=numbers of persons randomised	n=100; mean (SD): 154 (29)<br>n assumed=numbers of persons randomised		Serious concerns
Howel 2019[552]	wlfr	ac	Homecare (Only pts receiving care/ hours per week)	24 months	n=42; mean (SD): 53.7 (66.3)	n=52; mean (SD): 42 (56)	MD values: 26.3 (95% CI 0.8 to 56.1)	Serious concerns
Howel 2019[552]	wlfr	ac	Homecare (pts)	24 months	n=283; 42 persons (14.8%)	n=279; 52 persons (18.6%)		Serious concerns
Kerse 2014[556]	rsk-mfa-	ac	Homecare - domestic care only (pts)	3 years	n=1553; 627 persons (40.4%)	n=1428; 502 persons (35.2%)		Serious concerns
Kerse 2014[556]	rsk-mfa-	ac	Homecare - personal care only (pts)	3 years	n=1553; 104 persons (6.7%)	n=1428; 80 persons (5.6%)		Serious concerns
Metzelthin 2013[576]	educ & mfar(w/med+slfm)	ac	Homecare (hours)	24 months	n=134; mean (SD): 228.2 (288.5)	n=119; mean (SD): 220.2 (319.9)	MD values: 8 (95% CI -67.3 to 83.36); P = 0.83	Serious concerns
Monteserin Nadal 2008[578]	educ & rsk-mfa-	ac	Homecare (pts)	18 months	n=234; 17 persons (7.3%)	n=232; 19 persons (8.2%)		Serious concerns

Study	Intervention 1	Intervention 2	Outcome measure	Timepoint	Group 1 result	Group 2 result	Comparison	RoB assessment
Newbury 2001[582]	mfa-(w/med)	ac	Homecare (pts)	12 months	n=45; 6 persons (13.3%)	n=44; 5 persons (11.4%)		Serious concerns
Pathy 1992[588]	rsk-mfa-	ac	Homecare (pts)	3 years	n=223; 46 persons (20.6%)	n=196; 34 persons (17.3%)		Serious concerns
Thomas 2007[611]	mfar(w/med) (RAI-HC Assessment)	ac	Homecare (pts)	12 months	n=147; 4 persons(2.7%)	n=143; 4 persons (2.8%)		Serious concerns
Thomas 2007[611]	mfar(w/med) (RAI-HC Assessment Plus Referral)	ac	Homecare (pts)	12 months	n=152; 7 persons (4.6%)	n=143; 4 persons (2.8%)		Serious concerns
Thomas 2007[611]	mfar(w/med) (RAI-HC Assessment arm)	ac	Homecare (pts)	2 years	n=127; 6 persons (4.7%)	n=122; 1 person (0.8%)		Serious concerns
Thomas 2007[611]	mfar(w/med) (RAI-HC Assessment Plus Referral arm)	ac	Homecare (pts)	2 years	n=126; 8 persons (6.3%)	n=122; 1 person(0.8%)		Serious concerns
Whitehead 2016[627]	hmcr & ADL & aids & mfa-	hmcr & mfa-	Homecare (pts/ last 3 mth)	8 months	n=10; 6 persons (60.0%)	n=12; 6 persons (50.0%)		Serious concerns
Blom 2016[510]	mfa-(w/med+slfm)	ac	Homecare (pts)	12 months	n=185; 84 persons (45.4%)	n=657; 292 persons (44.4%)		Very serious concerns
Blom 2016[510]	mfa-(w/med+slfm)	ac	Homecare (hours)	12 months	n=288; mean: 137	n=1091; mean: 140	MD values: -2 (95% CI -128 to 123); P = 0.97	Very serious concerns
Lewin 2013[565]	hmcr & educ & mfar	hmcr	Homecare - personal care only (pts)	12 months	n=262; 67 persons (25.6%)	n=254; 151 persons (59.4%)	P <0.001	Very serious concerns

Results are organised by RoB assessment, Study name and timepoint.

Table 111 - Loneliness results

Study	Intervention 1	Intervention 2	Outcome measure	Timepoint	Group 1 result	Group 2 result	Comparison	RoB assessment
Jing 2018[554]	exrc & psyc	psyc	Loneliness (3-point Likert scale; 3=often lonely; 2=sometimes lonely; 1=not lonely) (smaller is better)	6 Months	n=39; Mean (SD): 1.41 (0.68)	n=40; Mean (SD): 1.85 (0.7)	P<0.05	No serious concerns
Bouman 2008[513]	mfar(w/med)	ac	Loneliness (de Jong-Gierveld Scale) (0-11) (smaller is better)	18 Months	n=139; Mean (SD): 3.5 (2.98);	n=154; Mean (SD): 4.0 (3.39);	MD values: 0.44; 95% CI: -0.37 to 1.24; P=0.29	Serious concerns

Study	Intervention 1	Intervention 2	Outcome measure	Timepoint	Group 1 result	Group 2 result	Comparison	RoB assessment
de Craen 2006[525]	mfa-	ac	Loneliness (de Jong-Gierveld Scale) (0-11) (smaller is better)	6 Months	n=175; Δ mean (SE): -0.1±0.2	n=176; Δ mean (SE): -0.1±0.2	MD change: -0.1; 95% CI: -0.5 to 0.4; P=0.78	Serious concerns
de Craen 2006[525]	mfa-	ac	Loneliness (de Jong-Gierveld Scale) (0-11) (smaller is better)	24 Months	n=143; Δ mean (SE): 0.1±0.2	n=154; Δ mean (SE): 0.1±0.2	MD change: 0.0 (95% CI: -0.7 to 0.6); p: 0.92	Serious concerns
van Rossum 1993 1993[621]	mfar	ac	Loneliness (de Jong-Gierveld Scale) (0-11) (smaller is better)	36 Months	n=232; Mean: 2.1	n=221; Mean: 1.9	MD values: 0.2 (95% CI: -0.2 to 0.6)	Serious concerns
Blom 2016 2016[510]	mfa-(w/med+slfm)	ac	Loneliness (de Jong-Gierveld Scale) (0-11) (smaller is better)	12 Months	n=288;	n=1091; pBaseline: 0.410;; Δ mean: -0.1 (95% CI -0.3 to 0.1)	MD change: -0.1; 95% CI: -0.5 to 0.3; P=0.661	Very serious concerns
Sherman 2016 2016[599]	mfa-(w/med)	ac	Loneliness (in Health Index) (1 item, 4-point scale) (larger is better)	1 Year	n=173; pBaseline: 0.03; Mean: 3.3	n=255; pBaseline: 0.03; Mean: 3.4	P=0.57<br>(Wilcoxon rank-sum test)	Very serious concerns

Results are organised by RoB assessment, Study name and timepoint.

Table 112 - Falls results

Study	Intervention 1	Intervention 2	Outcome measure	Timepoint	Group 1 result	Group 2 result	Comparison	RoB assessment
Rooijackers 2021[594]	hmcr & ADL & mfar(w/slfm)	hmcr	Falls (pts fell once or more / last 6 mths)	6 months	n=117; 42 fallers (35.9%)	n=117; 37 fallers (31.6%)	MD values: 0 (95% CI -0.7 to 0.6); P = .930	No serious concerns
Walters 2017[626]	mfar(w/slfm)	ac	Falls (pts fell once or more)	6 months	n=25; 6 fallers (24.0%)	n=23; 7 fallers (30.4%)		No serious concerns
Brettschneider 2015[514]	mfar(w/med)	ac	Falls (incidents / last 12 mths)	18 months	n=121; mean (SD): 2.2 (2.5); Δ mean ± SE: 0.63 ± 0.1 [95% CI 0.47 to 0.86]	n=119; mean (SD): 3.7 (4.2); Δ mean ± SE: 1.96 ± 0.29 [95% CI 1.46 to 2.63]	IRR: 0.32±0.07 (95% CI 0.22 to 0.49); P <0.001<br>Poisson regression	Serious concerns
Carpenter 1990[516]	rsk-mfa-	ac	Falls (incidents / last 1 mth)	3 years	n=206; 12 falls (5.8%)	n=213; 36 falls (16.9%)	P <0.05	Serious concerns
Coleman 1999[521]	educ & mfar(w/med+slfm)	ac	Falls (pts fell once or more / last 12 mths)	12 months	n=79	n=63	P = 0.27	Serious concerns
Coleman 1999[521]	educ & mfar(w/med+slfm)	ac	Falls (pts fell once or more / last 12 mths)	24 months	n=78, 43.5%	n=49, 35.6%	P = 0.63	Serious concerns
Dorresteijn 2016[526]	ADL	ac	Falls (pts fell once or more / last 12 mths)	12 months	n=166; 94 fallers (56.6%)	n=180; 106 fallers (58.9%)	OR: 0.79 (95% CI 0.5 to 1.23); P = 0.292	Serious concerns
Dorresteijn 2016[526]	ADL	ac	Falls (incidents / last 12 mths)	12 months	n=166; 362 falls (218.1%)	n=180; 429 falls (238.3%)	IRR: 0.86 (95% CI 0.65 to 1.13); P = 0.273	Serious concerns

Study	Intervention 1	Intervention 2	Outcome measure	Timepoint	Group 1 result	Group 2 result	Comparison	RoB assessment
Fabacher 1994[528]	mfar(w/med)	ac	Falls (pts fell once or more)	1 years	n=100, 14%	n=95, 23%		Serious concerns
Jitapunkul 1998[555]	rsk-mfa-	ac	Falls (pts fell once or more / last 3 mths)	3 years	n=57, 5.3%	n=59, 10.2%		Serious concerns
King 2012[557]	hmcr & ADL & mfar(w/slfm)	hmcr	Falls (pts fell once or more / last 3 mths)	7 months	n=93; 17 fallers (18.3%)	n=90; 23 fallers (25.7%)		Serious concerns
Kono 2016[558]	mfar(w/med)	mfar	Falls (pts fell once or more / last 12 mths)	12 months	n=154; 38 fallers (24.7%)	n=151; 42 fallers (27.8%)		Serious concerns
Kono 2016[558]	mfar(w/med)	mfar	Falls (pts fell once or more / last 12 mths)	24 months	n=138; 41 fallers (29.7%)	n=142; 48 fallers (33.8%)		Serious concerns
Monteserin Nadal 2008[578]	educ & rsk-mfa-	ac	Falls (pts fell once or more)	18 months	n=217; 50 fallers (23.0%)	n=213; 44 fallers (20.7%)		Serious concerns
Newbury 2001[582]	mfa-(w/med)	ac	Falls (pts fell once or more / last 12 mths)	12 months	n=45; 12 fallers (26.7%)	n=44; 17 fallers (38.6%)	OR: 0.58 (95% CI 0.21 to 1.55); P = 0.32	Serious concerns
Ng 2015[584]	cgn & ntr & exrc	ac	Falls (pts fell once or more)	6 months	n=47; 1 faller (2.1%)	n=47; 5 fallers (10.6%)	P = 0.38	Serious concerns
Ng 2015[584]	cgn & ntr & exrc	ac	Falls (pts fell once or more)	12 months	n=46; 2 fallers (4.3%)	n=46; 5 fallers (10.9%)	P = 0.67	Serious concerns
Profener 2016[591]	educ & mfar	ac	Falls (pts fell once or more / last 12 mths)	2 years	n=134; 59 fallers (44.0%)	n=259; 123 fallers (47.5%)		Serious concerns
Rooijackers 2021[594]	hmcr & ADL & mfar(w/slfm)	hmcr	Falls (pts fell once or more / last 6 mths)	12 months	n=102; 36 fallers (35.3%)	n=99; 27 fallers (27.3%)	MD values: 0 (95% CI -0.7 to 0.4); P = 0.951	Serious concerns
Rubenstein 2007[595]	mfar(w/med)	ac	Falls (pts fell once or more / last 3 mths)	24 months	n=298; 81 fallers (27.2%)	n=309; 71 fallers (23.0%)	P >0.05	Serious concerns
Serra-Prat 2017[597]	ntr & exrc	ac	Falls (pts fell once or more / last 3 mths)	12 months	n=61; 11 fallers (18.0%)	n=72; 14 fallers (19.4%)	OR: 0.87 (95% CI 0.36 to 2.11); P = 0.76	Serious concerns
Suijker 2016[605]	mfar(w/med)	ac	Falls (incidents / last 6 mths)<br>Estimated incidence rates adjusted for baseline age, sex, socio-economic status, level of education, and score of outcome. Two other ratios available without adjustment and with baseline adjustment only.	6 months	n=1017; mean: 0.25 (95% CI 0.21 to 0.29)	n=918; mean: 0.22 (95% CI 0.18 to 0.26)		Serious concerns
Suijker 2016[605]	mfar(w/med)	ac	Falls (incidents / last 6 mths)	12 months	n=936; mean: 0.26 (95% CI 0.21 to 0.3)	n=817; mean: 0.22 (95% CI 0.19 to 0.26)		Serious concerns
Suijker 2016[605]	mfar(w/med)	ac	Falls (incidents / last 6 mths)	24 months	n=924; mean: 0.29 (95% CI 0.24 to 0.34)	n=812; mean: 0.25 (95% CI 0.21 to 0.3)	IRR: 1.15 (95% CI 0.98 to 1.34); P = 0.08	Serious concerns

Study	Intervention 1	Intervention 2	Outcome measure	Timepoint	Group 1 result	Group 2 result	Comparison	RoB assessment
Whitehead 2016[627]	hmcr & ADL & aids & mfa-	hmcr & mfa-	Falls (pts fell once or more / last 3 mths)	8 months	n=10; 2 fallers (20.0%)	n=12; 6 fallers (50.0%)		Serious concerns
Whitehead 2016[627]	hmcr & ADL & aids & mfa-	hmcr & mfa-	Falls (incidents / only pts that fell / last 3 mths)	8 months	n=10; mean (SD): 1 (0)	n=12; mean (SD): 1.5 (1.22)		Serious concerns
Henderson 2005[548]	mfar	ac	Falls (pts fell once or more)	12 months	n=61; 11 fallers (18.0%)	n=63; 21 fallers (33.3%)		Very serious concerns
Henderson 2005[548]	mfar	ac	Falls (incidents)	12 months	n=61; mean (SD): 0.25 (0.65); range: 0 to 4	n=63; mean (SD): 0.51 (1.05); range: 0 to 7	P = 0.098, F=2.778, df=1,108	Very serious concerns
Parsons M 2012[587]	hmcr & mfar	hmcr & mfa-	Falls (incidents)	24 months	n=169; 152 falls (89.9%)	n=182; 175 falls (96.2%)		Very serious concerns
Parsons M 2012[587]	hmcr & mfar	hmcr & mfa-	Falls (pts fell once or more)	24 months	n=169; 69 fallers (40.8%)	n=182; 77 fallers (42.3%)		Very serious concerns
Parsons M 2017[586]	hmcr & ADL & mfar(w/slfm)	hmcr & mfa-	Falls (pts fell once or more)	24 months	n=56; 24 fallers (42.9%)	n=57; 23 fallers (40.4%)	P = 0.8503	Very serious concerns
Parsons M 2017[586]	hmcr & ADL & mfar(w/slfm)	hmcr & mfa-	Falls (incidents)	24 months	n=56; 63 falls (112.5%)	n=57; 43 falls (75.4%)		Very serious concerns

Results are organised by RoB assessment, Study name and timepoint.

References

1 Crocker TF, Lam N, Ensor J, et al. Community-based complex interventions to sustain independence in older people, stratified by frailty: a systematic review and network meta-analysis. *Health Technol Assess* in press.

2 Crocker TF, Clegg A, Riley RD, et al. Community-based complex interventions to sustain independence in older people, stratified by frailty: a protocol for a systematic review and network meta-analysis. *BMJ Open* 2021; **11**: e045637. doi: 10.1136/bmjopen-2020-045637.

3 Doi SA, Furuya-Kanamori L, Xu C, et al. The Odds Ratio is “portable” across baseline risk but not the Relative Risk: Time to do away with the log link in binomial regression. *J Clin Epidemiol* 2022; **142**: 288–93. doi: 10.1016/j.jclinepi.2021.08.003.

4 Schünemann H, Higgins J, Vist G, et al. Chapter 14: Completing ‘Summary of findings’ tables and grading the certainty of the evidence. In: Higgins J, Thomas J, Chandler J, et al., eds. Cochrane Handbook for Systematic Reviews of Interventions version 6.3 (updated February 2022): Cochrane; 2022.

5 Guyatt GH, Oxman AD, Schünemann HJ, Tugwell P, Knottnerus A. GRADE guidelines: a new series of articles in the Journal of Clinical Epidemiology. *J Clin Epidemiol* 2011; **64**: 380–2. doi: 10.1016/j.jclinepi.2010.09.011.

6 Schünemann H, Brożek J, Guyatt G, Oxman A, editors. Handbook for grading the quality of evidence and the strength of recommendations using the GRADE approach. Updated October 2013.: The GRADE Working Group; 2013.

7 Nikolakopoulou A, Higgins JPT, Papakonstantinou T, et al. CINeMA: An approach for assessing confidence in the results of a network meta-analysis. *PLoS Med* 2020; **17**: e1003082. doi: 10.1371/journal.pmed.1003082.

8 Papakonstantinou T, Nikolakopoulou A, Higgins JPT, Egger M, Salanti G. CINeMA: Software for semiautomated assessment of the confidence in the results of network meta-analysis. *Campbell Systematic Reviews* 2020; **16**: e1080. doi: 10.1002/cl2.1080.

9 Shields GE, Elvidge J. Challenges in synthesising cost-effectiveness estimates. *Syst Rev* 2020; **9**: 289. doi: 10.1186/s13643-020-01536-x.

10 Langan D, Higgins JPT, Jackson D, et al. A comparison of heterogeneity variance estimators in simulated random-effects meta-analyses. *Res Synth Methods* 2019; **10**: 83–98. doi: 10.1002/jrsm.1316.

11 White IR. Multivariate Random-effects Meta-analysis. *Stata J* 2009; **9**: 40–56. doi: 10.1177/1536867X0900900103.

12 Beswick AD, Rees K, Dieppe P, et al. Complex interventions to improve physical function and maintain independent living in elderly people: a systematic review and meta-analysis. *Lancet* 2008; **371**: 725–35. doi: 10.1016/s0140-6736(08)60342-6.

13 Stuck AE, Egger M, Hammer A, Minder CE, Beck JC. Home visits to prevent nursing home admission and functional decline in elderly people: Systematic review and meta-regression analysis. *JAMA* 2002; **287**: 1022–8. doi: 10.1001/jama.287.8.1022.

14 Tappenden P, Campbell F, Rawdin A, Wong R, Kalita N. The clinical effectiveness and cost-effectiveness of home-based, nurse-led health promotion for older people: a systematic review. *Health Technol Assess* 2012; **16**: 1–72. doi: 10.3310/hta16200.

15 Lefebvre C, Glanville J, Briscoe S, et al. Cochrane Highly Sensitive Search Strategy for identifying randomized trials in MEDLINE: sensitivity- and precision-maximizing version (2008 revision); Ovid format. In: Technical Supplement to Chapter 4: Searching for and selecting studies. In: Higgins J, Thomas J, Chandler J, et al., eds. Cochrane Handbook for Systematic Reviews of Interventions version 6.2 (updated February 2021): Cochrane; 2021.

16 Cooper C, Varley-Campbell J, Carter P. Established search filters may miss studies when identifying randomized controlled trials. *J Clin Epidemiol* 2019; **112**: 12–9. doi: 10.1016/j.jclinepi.2019.04.002.

17 Lefebvre C, Glanville J, Briscoe S, et al. Cochrane Highly Sensitive Search Strategy for identifying controlled trials in Embase: (2018 revision); Ovid format. In: Technical Supplement to Chapter 4: Searching for and selecting studies. In: Higgins J, Thomas J, Chandler J, et al., eds. Cochrane Handbook for Systematic Reviews of Interventions version 6.2 (updated February 2021); 2021.

18 Eady AM, Wilczynski NL, Haynes RB. PsycINFO search strategies identified methodologically sound therapy studies and review articles for use by clinicians and researchers. *J Clin Epidemiol* 2008; **61**: 34–40. doi: 10.1016/j.jclinepi.2006.09.016.

19 McGowan J, Sampson M, Salzwedel DM, Cogo E, Foerster V, Lefebvre C. PRESS Peer Review of Electronic Search Strategies: 2015 Guideline Statement. *J Clin Epidemiol* 2016; **75**: 40–6. doi: 10.1016/j.jclinepi.2016.01.021.

20 Santesso N, Glenton C, Dahm P, et al. GRADE guidelines 26: informative statements to communicate the findings of systematic reviews of interventions. *J Clin Epidemiol* 2020; **119**: 126–35. doi: 10.1016/j.jclinepi.2019.10.014.
21 Glenton C, Santesso N, Rosenbaum S, et al. Presenting the results of Cochrane Systematic Reviews to a consumer audience: a qualitative study. *Med Decis Making* 2010; **30**: 566–77. doi: 10.1177/0272989x10375853.
22 Brydges CR. Effect size guidelines, sample size calculations, and statistical power in gerontology. *Innov Aging* 2019; **3**: 1–8. doi: 10.1093/geroni/igz036.
23 Aguilar MI, Hart R, Pearce LA. Oral anticoagulants versus antiplatelet therapy for preventing stroke in patients with non-valvular atrial fibrillation and no history of stroke or transient ischemic attacks. *Cochrane Database Syst Rev* 2007. doi: 10.1002/14651858.CD006186.pub2.
24 Sudlow CLM, Mason G, Maurice JB, Wedderburn CJ, Hankey GJ. Thienopyridine derivatives versus aspirin for preventing stroke and other serious vascular events in high vascular risk patients. *Cochrane Database Syst Rev* 2009. doi: 10.1002/14651858.CD001246.pub2.
25 Wright JM, Musini VM, Gill R. First-line drugs for hypertension. *Cochrane Database Syst Rev* 2018. doi: 10.1002/14651858.CD001841.pub3.
26 ACTRN12606000042549. *Tauranga Older Persons Comprehensive Assessment Trial (TOPCAT)*. World Health Organization: International Clinical Trials Registry Platform; 2006. URL: https://trialsearch.who.int/Trial2.aspx?TrialID=ACTRN12606000042549 (accessed 11 May 2020).
27 ACTRN12616000521426. *The EXPRESS pilot study: EXercise and PRotein Supplementation Supporting Autonomy in Prefrail & Frail Community Residing adults aged 65 years or older*. Australian New Zealand Clinical Trials Registry; 2016. URL: http://www.anzctr.org.au/ACTRN12616000521426.aspx (accessed 5 November 2020).
28 ACTRN12616001148460. *The impact of studying a new language and health-related lifestyle recommendations on thinking abilities and biomarkers in older individuals with memory complaints*. Australian New Zealand Clinical Trials Registry; 2016. URL: http://www.anzctr.org.au/ACTRN12616001148460.aspx (accessed 5 November 2020).
29 ACTRN12618001188224. *Improving mental health and resilience in older adults*. Australian New Zealand Clinical Trials Registry; 2018. URL: https://anzctr.org.au/ACTRN12618001188224.aspx (accessed 5 November 2020).
30 ACTRN12619000910101. *Can motor imagery training improve strength and walking in older adults following discharge from rehabilitation?* World Health Organization: International Clinical Trials Registry Platform; 2019. URL: https://trialsearch.who.int/Trial2.aspx?TrialID=ACTRN12619000910101 (accessed 5 November 2020).
31 ACTRN12619001055190. *Integrated physical and cognitive training intervention to reverse cognitive frailty among community dwelling older adults*. Australian New Zealand Clinical Trials Registry; 2019. URL: https://anzctr.org.au/ACTRN12619001055190.aspx (accessed 5 November 2020).
32 Adelman RD, Fields SD, Jutagir R. Geriatric education part II: The effect of a well elderly program on medical student attitudes toward geriatric patients. *J Am Geriatr Soc* 1992; **40**: 970–3.
33 Ahmad A, Hugtenburg J, Welschen LM, Dekker JM, Nijpels G. Effect of medication review and cognitive behaviour treatment by community pharmacists of patients discharged from the hospital on drug related problems and compliance: design of a randomized controlled trial. *BMC Public Health* 2010; **10**: 133. doi: 10.1186/1471-2458-10-133.
34 Aimonino Ricauda N, Tibaldi V, Bertone P, et al. The RAD-HOME Project: A pilot study of home delivery of radiology services. *Arch Intern Med* 2011; **171**: 1678–80.
35 Akihiro S, Taira Y, Maeda K, Natsume K, Sakakima H. Feasibility and effectiveness of home-based exercise programs on physical performance and health-related quality of life of the older people dwelling on an isolated, doctor-less island. *Geriatrics & Gerontology International* 2018; **18**: 1313–7. doi: 10.1111/ggi.13459.
36 Albert SM, King J, Dew MA, et al. Design and Recruitment for a Randomized Controlled Trial of Problem-Solving Therapy to Prevent Depression among Older Adults with Need for Supportive Services. *Am J Geriatr Psychiatry* 2016; **24**: 94–102. doi: 10.1016/j.jagp.2015.11.003.
37 Anders J, Profener F, Dapp U, et al. [Health and competence: detection and decoding using comprehensive assessments in the Longitudinal Urban Cohort Ageing Study (LUCAS)]. *Z Gerontol Geriatr* 2012; **45**: 271–8. doi: 10.1007/s00391-012-0312-8.
38 Andrew M. Deprescribing in Primary Health Care. https://ClinicalTrials.gov/show/NCT03903484; 2019.
39 Anonymous. IVCP (Interfaith Volunteer Caregivers Program) helps frail elderly, disabled people in their homes on large scale. *Volunt Action Leadersh* 1989: 10–2.

40 Anonymous. Regular health assessments increase nursing home admissions and provide small improvements in quality of life in elderly people. *Evidence-Based Healthcare and Public Health* 2005; **9**: 135–6. doi: 10.1016/j.ehbc.2005.01.003.

41 Anttila SK, Huhtala HS, Pekurinen MJ, Pitkajarvi TK. Cost-effectiveness of an innovative four-year post-discharge programme for elderly patients--prospective follow-up of hospital and nursing home use in project elderly and randomized controls. *Scand J Public Health* 2000; **28**: 41–6. doi: 10.1177/140349480002800108.

42 Applebaum R, Straker J, Mehdizadeh S, Warshaw G, Gothelf E. Using high-intensity care management to integrate acute and long-term care services: substitute for large scale system reform? *Care Management Journals* 2002; **3**: 113–9. doi: 10.1891/cmaj.3.3.113.57445.

43 Arendts G, Bullow K, Etherton-Beer C, et al. A randomized-controlled trial of a patient-centred intervention in high-risk discharged older patients. *Eur J Emerg Med* 2018; **25**: 237-41. doi: 10.1097/MEJ.0000000000000444.

44 Aung MN, Moolphate S, Yuasa M, et al. Community-Integrated Intermediary Care (CIIC) Service Model to Enhance Family-Based, Long-Term Care for Older People: Protocol for a Cluster Randomized Controlled Trial in Thailand. *JMIR Research Protocols* 2021; **10**: e20196. doi: https://dx.doi.org/10.2196/20196.

45 Baker MK, Kennedy DJ, Bohle PL, et al. Efficacy and feasibility of a novel tri-modal robust exercise prescription in a retirement community: A randomized, controlled trial. *J Am Geriatr Soc* 2007; **55**: 1–10.

46 Ball K, Berch DB, Helmers KF, et al. Effects of cognitive training interventions with older adults: A randomized controlled trial. *J Am Med Assoc* 2002; **288**: 2271–81.

47 Ball KK, Ross LA, Roth DL, Edwards JD. Speed of Processing Training in the ACTIVE Study: How Much Is Needed and Who Benefits? *J Aging Health* 2013; **25**: 65S–84S.

48 Bandinelli S, Lauretani F, Boscherini V, et al. A randomized, controlled trial of disability prevention in frail older patients screened in primary care: the FRASI study. Design and baseline evaluation. *Aging Clin Exp Res* 2006; **18**: 359–66.

49 Bardsley M, Steventon A, Doll H. Impact of telehealth on general practice contacts: findings from the whole systems demonstrator cluster randomised trial. *BMC Health Serv Res* 2013; **13**: 395.

50 Bauer J. RN news watch: clinical highlights. Preventive exercise program can benefit some frail elderly patients. *RN* 2002; **65**: 18–79.

51 Bauer J. Clinical highlights. Nurse 'coaches' can help keep elderly patients from being readmitted. *RN* 2006; **69**: 14–.

52 Baumann H, Oswald WD, Hagen B, Rupprecht R. Maintaining and Supporting Independent Living in Old Age (SIMA)-Part XIV: Longitudinal analysis of long-term training effects on the psychomotor status. *Zeitschrift fur Gerontopsychologie und -psychiatrie* 1999; **12**: 227–44.

53 Beck AM, Hansen BS. Hand grip strenght as an outcome predictor of the efficacy of nutritional support to increase functional and nutritional status of geriatric medical patients after discharge. *Clinical nutrition (edinburgh, scotland)* 2013; **32**: S80-S1.

54 Beck AM, Kjaer S, Hansen BS, Storm RL, Thal-Jantzen K, Bitz C. Follow-up home visits with registered dietitians have a positive effect on the functional and nutritional status of geriatric medical patients after discharge: a randomized controlled trial. *Clin Rehabil* 2013; **27**: 483–93.

55 Beck AM, Rask K, Leedo E, Jensen LL, Martins K, Vedelspang A. Nutritional support in a cross-sector model for the rehabilitation of geriatric patients: a randomised controlled trial. *Clinical nutrition (edinburgh, scotland)* 2014; **33**: S37-.

56 Beck A, Andersen UT, Leedo E, et al. Does adding a dietician to the liaison team after discharge of geriatric patients improve nutritional outcome: a randomised controlled trial. *Clin Rehabil* 2015; **29**: 1117–28.

57 Beland F, Bergman H, Lebel P, et al. Integrated services for frail elders (SIPA): a trial of a model for Canada. *Can J Aging* 2006; **25**: 5–42.

58 Belchior P, Yam A, Thomas KR, et al. Computer and Videogame Interventions for Older Adults' Cognitive and Everyday Functioning. *Games for Health Journal* 2019; **8**: 129–43.

59 Belleville S, Cuesta M, Bieler-Aeschlimann M, et al. Rationale and protocol of the StayFitLonger study: a multicentre trial to measure efficacy and adherence of a home-based computerised multidomain intervention in healthy older adults. *BMC Geriatr* 2020; **20**: 315. doi: https://dx.doi.org/10.1186/s12877-020-01709-2.

60 Belqaid K, Brandt CF, Lugnet K, et al. Adding a dietitian to a discharge follow-home team is cost-effective for geriatric patients at nutritional risk. *Clinical nutrition (edinburgh, scotland)* 2016; **35**: S160-.

61 Berglund H, Hasson H, Kjellgren K, Wilhelmson K. Effects of a continuum of care intervention on frail older persons' life satisfaction: a randomized controlled study. *Journal of clinical nursing* 2015; **24**: 1079-90.

62 Bernabei R, Cesari M, Landi F. Physical frailty and sarcopenia: background, rationale, and design of the SPRINT-T project. *Eur Geriatr Med* 2014; **5**: S33-S4.
63 Binder EF, Schechtman KB, Ehsani AA, et al. Effects of exercise training on frailty in community-dwelling older adults: Results of a randomized, controlled trial. *J Am Geriatr Soc* 2002; **50**: 1921–8.
64 Blanchard CM, Fortier M, Sweet S, et al. Explaining physical activity levels from a self-efficacy perspective: The physical activity counseling trial. *Ann Behav Med* 2007; **34**: 323–8.
65 Bondoc I, Cochrane SK, Church TS, et al. Effects of a One-Year Physical Activity Program on Serum C-Terminal Agrin Fragment (CAF) Concentrations among Mobility-Limited Older Adults. *J Nutr Health Aging* 2015; **19**: 922-7.
66 Bonnefoy M, Boutitie F, Mercier C, et al. Efficacy of a home-based intervention programme on the physical activity level and functional ability of older people using domestic services: a randomised study. *J Nutr Health Aging* 2012; **16**: 370–7.
67 Bosch-Lenders D, Jansen J, Stoffers HEJH, et al. The effect of a comprehensive, interdisciplinary medication review on quality of life and medication use in community dwelling older people with polypharmacy. *Journal of Clinical Medicine* 2021; **10**: 1–13. doi: 10.3390/jcm10040600.
68 Botoseneanu A, Ambrosius WT, Beavers DP, et al. Prevalence of metabolic syndrome and its association with physical capacity, disability, and self-rated health in Lifestyle Interventions and Independence for Elders Study participants. *J Am Geriatr Soc* 2015; **63**: 222–32.
69 Boult C, Reider L, Frey K, et al. Early effects of "Guided Care" on the quality of health care for multimorbid older persons: a cluster-randomized controlled trial. *J Gerontol A Biol Sci Med Sci* 2008; **63**: 321–7.
70 Brandon LJ, Boyette LW, Lloyd A, Gaasch DA. Resistive Training and Long-Term Function in Older Adults. *J Aging Phys Act* 2004; **12**: 10–28.
71 Brazil K, Scott D, Wallace E, et al. Anticipatory care planning intervention for older adults at risk of functional decline: Study protocol for a primary care cluster feasibility randomised trial. *Trials* 2020; **21**: 168. doi: 10.1186/s13063-020-4100-2.
72 NCT03902743. *Anticipatory Care Planning Intervention for Older Adults at Risk of Functional Decline: a Primary Care Feasibility Study*. US National Library of Medicine: ClinicalTrials.gov; 2019. URL: https://clinicaltrials.gov/show/NCT03902743 (accessed 11 May 2020).
73 Buford TW, Manini TM, Hsu FC, et al. Angiotensin-converting enzyme inhibitor use by older adults is associated with greater functional responses to exercise. *J Am Geriatr Soc* 2012; **60**: 1244-52.
74 Buford TW, Miller ME, Church TS, et al. Antihypertensive Use and the Effect of a Physical Activity Intervention in the Prevention of Major Mobility Disability Among Older Adults: The LIFE Study. *J Gerontol A Biol Sci Med Sci* 2016: 974–81.
75 Burke L, Lee AH, Jancey J, et al. Physical activity and nutrition behavioural outcomes of a home-based intervention program for seniors: a randomized controlled trial. *International Journal of Behavioral Nutrition & Physical Activity* 2013; **10**: 14.
76 Burton LC, Paglia MJ, German PS, Shapiro S, Damiano AM. The effect among older persons of a general preventive visit on three health behaviors: smoking, excessive alcohol drinking, and sedentary lifestyle. The Medicare Preventive Services Research Team. *Prev Med* 1995; **24**: 492–7.
77 Burton E, Lewin G, Clemson L, Boldy D. Physical activity programs for older people receiving a restorative home care service, which is more effective? *Journal of science and medicine in sport* 2012; **15**: S304-.
78 Burton E, Lewin G, Clemson L, Boldy D. Effectiveness of a lifestyle exercise program for older people receiving a restorative home care service: study protocol for a pragmatic randomised controlled trial. *BMC Health Serv Res* 2013; **13**: 419.
79 Burton E, Lewin G, Clemson L, Boldy D. Effectiveness of a lifestyle exercise program for older people receiving a restorative home care service: a pragmatic randomized controlled trial. *Clin Interv Aging* 2013; **8**: 1591–601.
80 Burton E, Lewin G, Clemson L, Boldy D. Determining the feasibility of a lifestyle activity program for inclusion in a restorative home care service: A pilot study. *Act Adapt Aging* 2014; **38**: 79–93.
81 Buss A, Wolf-Ostermann K, Dassen T, Lahmann N, Strupeit S. Effectiveness of educational nursing home visits on quality of life, functional status and care dependency in older adults with mobility impairments: a randomized controlled trial. *J Eval Clin Pract* 2016; **22**: 213–21.
82 Byles JE, Tavener M, Fitzgerald P, Nair BR. The preventive care trial: a three year randomised controlled trial of home based health assessments for the over 70's. *Intern Med J* 2002; **32**: A45.
83 Byles JE, Tavener M, O'Connell RL, et al. Randomised controlled trial of health assessments for older Australian veterans and war widows. *Med J Aust* 2004; **181**: 186–90.

84 Callahan KE, Lovato L, Miller ME, et al. Self-Reported Physical Function As a Predictor of Hospitalization in the Lifestyle Interventions and Independence for Elders Study. *J Am Geriatr Soc* 2018; **66**: 1927–33.
85 Caplan GA, Williams AJ, Daly B, Abraham K. A randomized, controlled trial of comprehensive geriatric assessment and multidisciplinary intervention after discharge of elderly from the emergency department--the DEED II study. *J Am Geriatr Soc* 2004; **52**: 1417–23.
86 Carrie I, Van Kan GA, Gillette-Guyonnet S, et al. Recruitment strategies for preventive trials. the MAPT study (Multidomain Alzheimer preventive trial). *J Nutr Health Aging* 2012; **16**: 355–9.
87 Carrie I, Delrieu J, Lala F, et al. Consultation for Alzheimer's disease prevention: an effective recruitment strategy for preventive trials. *Journal of prevention of alzheimer's disease* 2018; **5**: S159-S60.
88 Cartwright M, Hirani SP, Rixon L, et al. Effect of telehealth on quality of life and psychological outcomes over 12 months (Whole Systems Demonstrator telehealth questionnaire study): nested study of patient reported outcomes in a pragmatic, cluster randomised controlled trial. *BMJ* 2013; **346**: f653.
89 Cavaillon C. Effect of physical activity on cognitive function: Results of the LIFE (lifestyle interventions and independence for elders) pilot study. [French]. *Cah de l'Annee Gerontol* 2010; **2**: 221–3.
90 Cesari M, Marzetti E, Calvani R, et al. The need of operational paradigms for frailty in older persons: the SPRINTT project. *Aging Clin Exp Res* 2017; **29**: 3–10.
91 Chan DCD, Tsou HH, Yang RS, et al. A pilot randomized controlled trial to improve geriatric frailty. *BMC Geriatr* 2012; **12**: 1–12.
92 Chin APMJM, de Jong N, Schouten EG, Hiddink GJ, Kok FJ. Physical exercise and/or enriched foods for functional improvement in frail, independently living elderly: a randomized controlled trial. *Arch Phys Med Rehabil* 2001; **82**: 811–7.
93 Ching Wong AK, Yuet Wong FK, Sum Chow KK, Man Wong S. Effects of a Video-Based mHealth Program for Homebound Older Adults: Study Protocol for a Pilot Randomized Controlled Trial. *Gerontology.* 2021. doi: 10.1159/000516967.
94 Choi M, Mezuk B, Lohman MC, Edwards JD, Rebok GW. Gender and Racial Disparities in Driving Cessation Among Older Adults. *J Aging Health* 2013; **25**: 147S–62S.
95 Clarke M, Clarke SJ, Jagger C. Social intervention and the elderly: a randomized controlled trial. *Am J Epidemiol* 1992; **136**: 1517–23.
96 Coburn KD, Marcantonio S, Lazansky R, Keller M, Davis N. Effect of a community-based nursing intervention on mortality in chronically ill older adults: a randomized controlled trial. *PLoS Medicine / Public Library of Science* 2012; **9**: e1001265.
97 Cochrane SK, Chen SH, Fitzgerald JD, et al. Association of Accelerometry-Measured Physical Activity and Cardiovascular Events in Mobility-Limited Older Adults: the LIFE (Lifestyle Interventions and Independence for Elders) Study. *J Am Heart Assoc* 2017; **6**.
98 Cornu C, Carre C, Guetemme G, et al. Prevention of Dependency in Elderly Persons: A Randomised Study in the Community. [French]. *Therapie* 2003; **58**: 451–6.
99 Corrado OJ. Caring for older hospital-at-home patients. *Age Ageing* 2000; **29**: 97–8.
100 Crandall KJ, Steenbergen KI. Older adults' functional performance and health knowledge after a combination exercise, health education, and bingo game. *Gerontology and Geriatric Medicine.* 2015; **1**.
101 Crews DC, Delaney AM, Walker JL, et al. Improving physical function and social networks of older adults with esrd: Development and testing of seniors optimizing community integration to advance better living with ESRD (sociable). *J Am Soc Nephrol* 2017; **28**: 643.
102 Ctri, Indian Council of Medical Research Y. Elderly Peer Volunteer led Happiness Program. Effect of Peer Volunteer Led Intervention on addressing Loneliness and its consequences among Elderly residing in Urban Field Practice Area of JIPMER, Puducherry - A Single Arm Before-After Intervention Study. Dr Swaroop Kumar Sahu Department of Preventive and Social Medicine, Room No 411, JIPMER Puducherry Apartment No A602, Sreenivas Towers, Ariyankuppam, Puducherry-605007 swaroop.sahu@gmail.com 9488494613</Contact_Tel> JIPMER Indian Council of Medical Research <Ethics_review_status>Approved</Ethics_review_status> <Ethics_review_approval_date>01/01/1900</Ethics_review_approval_date> <Ethics_review_contact_name>InstitutionalEthicsCommitteeJIPMER</Ethics_review_contact_name> <Ethics_review_contact_address /> <Ethics_review_contact_phone /> <Ethics_review_contact_email /> <results_url_link /> </Trial>; 2019.
103 Cucinotta D, Savorani G, Piscaglia F, Galletti L, Petazzoni E, Bolondi L. The chronically ill elderly patients discharged from the hospital: interim report from a controlled study of home care attendance. *Archives of Gerontology & Geriatrics - Supplement* 2004: 103–8.
104 Cunliffe AL, Gladman JRF, Husbands SL, Miller P, Dewey ME, Harwood RH. Sooner and heathier: A randomised controlled trial and interview study of an early discharge rehabilitation service for older people. *Age Ageing* 2004; **33**: 246–52.

105 Cwirlej-Sozanska A, Wisniowska-Szurlej A, Wilmowska-Pietruszynska A, et al. Evaluation of the Effect of 16 Weeks of Multifactorial Exercises on the Functional Fitness and Postural Stability of a Low-Income Elderly Population. *Top Geriatr Rehabil* 2018; **34**: 251–61.
106 Czaja SJ, Boot WR, Charness N, Rogers WA, Sharit J. Improving Social Support for Older Adults Through Technology: Findings From the PRISM Randomized Controlled Trial. *Gerontologist* 2018; **58**: 467–77.
107 Daffner KR, Feng NC, Ryan E, et al. The feasibility of a home-based, subject-controlled, interactive physical exercise program to promote cognitive health in older adults. *Alzheimer's & dementia* 2017; **13**: 862-3.
108 Damanti S, Azzolino D, Riva S, Cano A, Marcucci M, collaborators FP. PoliFIT-INFOcus: A pilot study exploring how to promote physical activity in older people. *Eur J Intern Med* 2018; **57**: e41–e2.
109 Dangour AD, Albala C, Aedo C, et al. A factorial-design cluster randomised controlled trial investigating the cost-effectiveness of a nutrition supplement and an exercise programme on pneumonia incidence, walking capacity and body mass index in older people living in Santiago, Chile: the CENEX study protocol. *Nutr J* 2007; **6**: 14.
110 Danilovich MK, Diaz L, Ciolinio JD, Corcos DM. Functional resistance activities to impact frailty: a protocol for a randomized controlled trial involving home care aide and frail older adult dyads. *Contemporary clinical trials communications* 2017; **7**: 28-32.
111 Danilovich MK, Hughes SL, Corcos DM, Marquez DX, Eisenstein AR. Translating Strong for Life Into the Community Care Program: Lessons Learned. *J Appl Gerontol* 2017; **36**: 553–69.
112 Dapp U, Fertmann R, Anders J, et al. [The Longitudinal Urban Cohort Ageing Study (LUCAS)]. *Z Gerontol Geriatr* 2011; **44**: 55–72.
113 Dapp U, Anders J, Golgert S, von Renteln-Kruse W, Minder CE. Resources and risks in old age. The LUCAS-I marker set for a classification of elderly people as FIT, pre-FRAIL and FRAIL. first results on validity from the longitudinal urban cohort ageing study (LUCAS), Hamburg. *Z Gerontol Geriatr* 2012; **45**: 262–70.
114 Dapp U, Anders J, Santos-Eggimann B, Seematter-Bagnoud L. Correlates of frailty, prediction of functional decline and preventative approaches-selected results from the lausanne cohort 65+ (Lc65+) study (switzerland) (lausanne) and the longitudinal urban cohort ageing study (lucas) (Germany). *Eur Geriatr Med* 2013; **1**: S3–S4.
115 Dapp U, Anders J, Minder CE, et al. Multi-component health PROmotion and primary preventive intervention programmes and LONG-term evaluation in HEALTHy community-dwelling senior citizens (PROLONG-HEALTH). *Eur Geriatr Med* 2014; **5**: S152-.
116 Dapp U, Anders JAM, Suijker JJM, Bjorkman MP. Various aspects of interventions on physical functioning in community dwelling older persons. *Eur Geriatr Med* 2014; **5**: S26-S7.
117 Dapp U, Anders J, Neumann L, et al. Two versions of the LUCAS Functional Ability Index using either ability "to walk 500 m" or "to cycle"-long term analyses on survival and need of nursing care (prolong health). *Eur J Epidemiol* 2016; **31**: S39–S40.
118 Dapp U, Dirksen-Fischer M, Rieger-Ndakorerwa G, et al. [Comparability of studies of epidemiological research on aging : Results from the Longitudinal Urban Cohort Ageing Study (LUCAS) and three representative Hamburg cross-sectional studies of healthy aging]. *Bundesgesundheitsblatt, Gesundheitsforschung, Gesundheitsschutz* 2016; **59**: 662–78.
119 Dapp U, Neumann L, Golgert S, et al. Long-term effects of the intervention "active health promotion in old age" over a 13 year period-results from the LUCAS cohort study (prolong health). *Eur J Epidemiol* 2016; **31**: S39.
120 Darzins P, Smith P. Assistive technology and environmental interventions helped slow functional decline in frail elderly adults...commentary on Mann WC, Ottenbacher KJ, Fraas L, et al. Effectiveness of assistive technology and environmental interventions in maintaining independence and reducing home care costs for the frail elderly. A randomized controlled trial. ARCH FAM MED 1999 May/Jun;8:210-7. *ACP J Club* 1999; **131**: 71–.
121 Datta R, Trentalange M, Van Ness PH, et al. Serious adverse events of older adults in nursing home and community intervention trials. *Contemporary Clinical Trials Communications* 2018; **9**: 77–80.
122 De Luca R, Torrisi M, Bramanti A, et al. A multidisciplinary Telehealth approach for community dwelling older adults. *Geriatric Nursing* 2021; **42**: 635–42. doi: 10.1016/j.gerinurse.2021.03.015.
123 de Souto Barreto P, Rolland Y, Maltais M, Vellas B. Associations of Multidomain Lifestyle Intervention with Frailty: Secondary Analysis of a Randomized Controlled Trial. *Am J Med* 2018; **131**: 1382.e7–.e13.
124 De Vreede PL, Samson MM, Van Meeteren NL, Van Der Bom JG, Duursma SA, Verhaar HJ. Functional tasks exercise versus resistance exercise to improve daily function in older women: A feasibility study. *Arch Phys Med Rehabil* 2004; **85**: 1952–61.

125 de Vreede PL, Samson MM, van Meeteren NL, Duursma SA, Verhaar HJ. Functional-task exercise versus resistance strength exercise to improve daily function in older women: a randomized, controlled trial. *J Am Geriatr Soc* 2005; **53**: 2–10. doi: 10.1111/j.1532-5415.2005.53003.x.
126 De Vriendt P, Peersman W, Florus A, Verbeke M, Van de Velde D. Improving Health Related Quality of Life and Independence in Community Dwelling Frail Older Adults through a Client-Centred and Activity-Oriented Program. A Pragmatic Randomized Controlled Trial. *J Nutr Health Aging* 2016; **20**: 35–40.
127 de Vries NM, Staal JB, Teerenstra S, Adang EM, Rikkert MG, Nijhuis-van der Sanden MW. Physiotherapy to improve physical activity in community-dwelling older adults with mobility problems (Coach2Move): study protocol for a randomized controlled trial. *Trials* 2013; **14**: 434.
128 Delbaere K, Bourgois J, Van Den Noortgate N, Vanderstraeten G, Willems T, Cambier D. A home-based multidimensional exercise program reduced physical impairment and fear of falling. *Acta Clin Belg* 2006; **61**: 340–50.
129 Delrieu J, Payoux P, Carrie I, et al. Multidomain intervention and/or omega-3 in nondemented elderly subjects according to amyloid status. *Alzheimer's & Dementia* 2019; **15**: 1392–401.
130 Denny KG, Tomaszewski-Farias S. A multi-modal intervention to enhance cognitive compensation strategies and promote brain health activities. *Alzheimer's and Dementia* 2017; **13**: P824.
131 Di Pollina L, Guessous I, Petoud V, et al. Integrated care at home reduces unnecessary hospitalizations of community-dwelling frail older adults: a prospective controlled trial. *BMC Geriatr* 2017; **17**: 1–10.
132 Docent AMB, Christensen AG, Hansen BS, Damsbo-Svendsen S, Møller TKS. Author response re. "Rehabilitation nutrition for undernourished participants in nursing home and home care: cluster randomized controlled study". *Nutrition. 32 (4) (pp 504), 2016. Date of publication: 01 apr 2016.* 2016. doi: 10.1016/j.nut.2015.10.020.
133 Donelle L, Regan S, Kerr M, et al. Caring near and far by connecting community-based clients and family member/friend caregivers using passive remote monitoring: Protocol for a pragmatic randomized controlled trial. *JMIR Res Protoc* 2020; **9**: e15027. doi: 10.2196/15027.
134 ISRCTN79884651. *Caring Near and Far: investigating the use of home-based remote monitoring technology with chronic care older adults to see if the technology helps people to remain safely at home and put off admission to hospital or long term care*. World Health Organization: International Clinical Trials Registry Platform; 2016. URL: https://trialsearch.who.int/Trial2.aspx?TrialID=ISRCTN79884651 (accessed 11 May 2020).
135 Dotson VM, Hsu FC, Langaee TY, et al. Genetic Moderators of the Impact of Physical Activity on Depressive Symptoms. *The Journal of Frailty & Aging* 2016; **5**: 6–14.
136 DRKS00024638. *Prevention for more participation in older ages*. World Health Organization: International Clinical Trials Registry Platform; 2021. URL: https://trialsearch.who.int/Trial2.aspx?TrialID=DRKS00024638 (accessed 26 August 2021).
137 Dunn RB, Lewis PA, Vetter NJ, Guy PM, Hardman CS, Jones RW. Health visitor intervention to reduce days of unplanned hospital re-admission in patients recently discharged from geriatric wards: the results of a randomised controlled study. *Arch Gerontol Geriatr* 1994; **18**: 15–23.
138 Eekhof J, De Bock G, Schaapveld K, Springer M. Effects of screening for disorders among the elderly: an intervention study in general practice. *Fam Pract* 2000; **17**: 329–33.
139 Elliott JC, Brincks AM, Feaster DJ, et al. Psychosocial factors associated with problem drinking among substance users with poorly controlled HIV infection. *Alcohol and Alcoholism* 2018; **53**: 603–10.
140 Englund DA, Kirn DR, Koochek A, et al. Nutritional Supplementation With Physical Activity Improves Muscle Composition in Mobility-Limited Older Adults, The VIVE2 Study: A Randomized, Double-Blind, Placebo-Controlled Trial. *J Gerontol A Biol Sci Med Sci* 2017; **73**: 95–101. doi: 10.1093/gerona/glx141.
141 Espeland MA, Gill TM, Guralnik J, et al. Designing clinical trials of interventions for mobility disability: Results from the lifestyle interventions and independence for elders pilot (LIFE-P) trial. *J Gerontol A Biol Sci Med Sci* 2007; **62**: 1237–43.
142 Espeland MA, Newman AB, Sink K, et al. Associations Between Ankle-Brachial Index and Cognitive Function: Results From the Lifestyle Interventions and Independence for Elders Trial. *J Am Med Dir Assoc* 2015; **16**: 682–9.
143 Falvey JR, Mangione KK, Nordon-Craft A, et al. Progressive Multicomponent Intervention for Older Adults in Home Health Settings Following Acute Hospitalization: Randomized Clinical Trial Protocol. *Phys Ther* 2019; **99**: 1141–9.
144 NCT02905370. *Progressing Home Health Rehabilitation for Older Adults*. US National Library of Medicine: ClinicalTrials.gov; 2016. URL: https://clinicaltrials.gov/show/NCT02905370 (accessed 11 May 2020).
145 Fasce GP, Aravena JMC, Araya CO, et al. Domiciliary intervention by occupational therapy after hospital discharge in order to prevent re-admission in the elderly: Study protocol for a randomised clinical trial. *Rev Esp Geriatr Gerontol* 2018; **53**: 337–43.

146 NCT02052401. *Domiciliary Intervention by Occupational Therapy to Prevent Elderly Rehospitalization*. US National Library of Medicine: ClinicalTrials.gov; 2014. URL: https://clinicaltrials.gov/show/NCT02052401 (accessed 11 May 2020).
147 Feingold E, Werby E. Supporting the independence of elderly residents through control over their environment. *Journal of Housing for the Elderly* 1990; **6**: 25–32.
148 Feldman, P H, Latimer, Davidson. Medicaid-funded home care for the frail elderly and disabled: evaluating the cost savings and outcomes of a service delivery reform. *Health services research* 1996; **31**: 489-508.
149 Feng NC, Ryan E, Kidane M, et al. Feasibility of an at-home, web-based, interactive exercise program for older adults. *Alzheimer's and Dementia: Translational Research and Clinical Interventions* 2019; **5**: 825–33.
150 Ferrat E, Bastuji-Garin S, Paillaud E, et al. Efficacy of nurse-led and general practitioner-led comprehensive geriatric assessment in primary care: protocol of a pragmatic three-arm cluster randomised controlled trial (CEpiA study). *BMJ Open* 2018; **8**: e020597.
151 Ferreira MM. Efficacy of a primary care geriatric intervention model in reversing frailty-protocol of a randomized clinical trial. *Eur Geriatr Med* 2018; **9**: S92. doi: 10.1007/s41999-018-0097-4.
152 Fielding RA, Rejeski WJ, Blair S, et al. The Lifestyle Interventions and Independence for Elders Study: design and methods. *J Gerontol A Biol Sci Med Sci* 2011; **66**: 1226-37.
153 Finkelstein SM, Speedie SM, Demiris G, Veen M, Lundgren JM, Potthoff S. Telehomecare: quality, perception, satisfaction. *Telemed J E Health* 2004; **10**: 122–8.
154 Finkelstein SM, Speedie SM, Potthoff S. Home telehealth improves clinical outcomes at lower cost for home healthcare. *Telemed J E Health* 2006; **12**: 128–36.
155 Finkelstein SM, Speedie SM, Zhou X, Ratner E, Potthoff S. VALUE: Virtual Assisted Living Umbrella for the Elderly -- user patterns. *Conference Proceedings: ... Annual International Conference of the IEEE Engineering in Medicine & Biology Society* 2006; **1**: 3294–6.
156 Fisher K, Markle-Reid M, Ploeg J, et al. Self-management program versus usual care for community-dwelling older adults with multimorbidity: A pragmatic randomized controlled trial in Ontario, Canada. *Journal of Comorbidity* 2020; **10**: 2235042X20963390. doi: https://dx.doi.org/10.1177/2235042X20963390.
157 Fletcher AE, Jones DA, Bulpitt CJ, Tulloch AJ. The MRC trial of assessment and management of older people in the community: objectives, design and interventions [ISRCTN23494848]. *BMC Health Serv Res* 2002; **2**: 21.
158 Fletcher AE, Price GM, Ng ES, et al. Population-based multidimensional assessment of older people in UK general practice: a cluster-randomised factorial trial. *Lancet* 2004; **364**: 1667–77.
159 Fontan E. Long-term effectiveness of a home intervention to reduce functional problems: results of a randomised, controlled trial. *Cah de l'Annee Gerontol* 2010; **2**: 188-90.
160 Fontan E. Results of a randomised trial of mutidisciplinary domiciliary care in elderly patients with low incomes. [French]. *Cah de l'Annee Gerontol* 2010; **2**: 183–7.
161 Forbes DA. An educational programme for primary healthcare providers improved functional ability in older people living in the community. *Evidence Based Nursing* 2005; **8**: 122–.
162 France M, McDowell C. Seniors helping seniors: A model of peer counselling for the aged. *Can Ment Health* 1982; **30**: 13–5.
163 Franse CB, Voorham AJJ, van Staveren R, et al. Evaluation design of Urban Health Centres Europe (UHCE): preventive integrated health and social care for community-dwelling older persons in five European cities. *BMC Geriatr* 2017; **17**: 1–8.
164 Frese T, Deutsch T, Keyser M, Sandholzer H. In-home preventive comprehensive geriatric assessment (CGA) reduces mortality-A randomized controlled trial. *Arch Gerontol Geriatr* 2012; **55**: 639–44.
165 Friedberg MW. Low-income seniors may benefit from a home-based primary care program. *J Clin Outcomes Manag* 2008; **15**: 64–5.
166 Frieswijk N, Steverink N, Buunk BP, Slaets JP. The effectiveness of a bibliotherapy in increasing the self-management ability of slightly to moderately frail older people. *Patient Educ Couns* 2006; **61**: 219-27.
167 Fritz H, Hu Y-L, Tarraf W, Patel P. Feasibility of a Habit Formation Intervention to Delay Frailty Progression Among Older African Americans: A Pilot Study. *Gerontologist* 2020; **60**: 1353–63. doi: 10.1093/geront/gnz143.
168 Gagnon AJ, Schein C, McVey L, Bergman H. Randomized controlled trial of nurse case management of frail older people. *J Am Geriatr Soc* 1999; **47**: 1118–24.
169 Gasmann KG, Lang E, Oswald WD, Hagen B, Rupprecht R. Maintaining and Supporting Independent Living in Old Age (SIMA)-Part XIII: Longitudinal analysis of long-term training effects on physical health. *Zeitschrift fur Gerontopsychologie und -psychiatrie* 1999; **12**: 205–26.

170 Geller D, Nilsen D, Gillen G, Vanlew S, Bernardo M. A Randomized Controlled Comparative Effectiveness Pilot Study on Unimanual and Bimanual Mirror Therapy as a Home Program. *Am J Occup Ther* 2016; **70**: 1–.

171 Giannini R, Petazzoni E, Savorani G, et al. Outcomes from a program of home care attendance in very frail elderly subjects. *Arch Gerontol Geriatr* 2007; **44**: 95–103.

172 Gillette S. The multidomain Alzheimer preventive trial (MAPT): a new approach for the prevention of Alzheimer's disease. *Alzheimer's & dementia* 2009; **5**: 145-.

173 Gillette-Guyonnet S, Andrieu S, Dantoine T, Dartigues JF, Touchon J, Vellas B. Commentary on "A roadmap for the prevention of dementia II. Leon Thal Symposium 2008." The Multidomain Alzheimer Preventive Trial (MAPT): A new approach to the prevention of Alzheimer's disease. *Alzheimer's and Dementia* 2009; **5**: 114–21.

174 Ginis KAM, Latimer AE, Brawley LR, Jung ME, Hicks AL. Weight training to activities of daily living: helping older adults make a connection. *Med Sci Sports Exerc* 2006; **38**: 116–21.

175 Giudici KV, de Souto Barreto P, Soriano G, Rolland Y, Vellas B. Defining Vitality: Associations of Three Operational Definitions of Vitality with Disability in Instrumental Activities of Daily Living and Frailty among Elderly Over a 3-Year Follow-Up (MAPT Study). *J Nutr Health Aging* 2019; **23**: 386–92.

176 Giudici KV, Guyonnet S, Rolland Y, Vellas B, de Souto Barreto P, Nourhashemi F. Body Weight Variation Patterns as Predictors of Cognitive Decline over a 5 Year Follow-Up among Community-Dwelling Elderly (MAPT Study). *Nutrients* 2019; **11**: 1371.

177 Godwin M, Pike A, McCrate F, et al. The healthy aged: Descriptive analysis by sex of cognitively functioning elderly patients 80 years and older living independently in the community. *Canadian family physician Medecin de famille canadien* 2015; **61**: e142–e7.

178 Godwin M, Gadag V, Pike A, et al. A randomized controlled trial of the effect of an intensive 1-year care management program on measures of health status in independent, community-living old elderly: the Eldercare project. *Fam Pract* 2016; **33**: 37–41.

179 Golas SB, Nikolova-Simons M, Palacholla R, et al. Predictive analytics and tailored interventions improve clinical outcomes in older adults: a randomized controlled trial. *npj Digital Medicine* 2021; **4**. doi: 10.1038/s41746-021-00463-y.

180 Goldberg EM, Marks SJ, Resnik LJ, Long S, Mellott H, Merchant RC. Can an Emergency Department-Initiated Intervention Prevent Subsequent Falls and Health Care Use in Older Adults? A Randomized Controlled Trial. *Annals of Emergency Medicine* 2020; **76**: 739-50. doi: 10.1016/j.annemergmed.2020.07.025.

181 Gorenberg BD. Health maintenance of the frail elderly: Informal and formal help at home. *Dissertation Abstracts International* 1986; **47**: 132–3.

182 Granbom M, Kristensson J, Sandberg M. Effects on leisure activities and social participation of a case management intervention for frail older people living at home: a randomised controlled trial. *Health Soc Care Community* 2017; **25**: 1416–29.

183 Green CA, Capitman J, Leutz W. Expanded care and quality of life among elderly social HMO members. *J Appl Gerontol* 2002; **21**: 333–51.

184 Groessl EJ, Kaplan RM, Rejeski WJ, et al. Health-Related Quality of Life in Older Adults at Risk for Disability. *Am J Prev Med* 2007; **33**: 214–8.

185 Gross AL, Rebok GW, Unverzagt FW, Willis SL, Brandt J. Word list memory predicts everyday function and problem-solving in the elderly: results from the ACTIVE cognitive intervention trial. *Aging Neuropsychology & Cognition* 2011; **18**: 129–46.

186 Gross AL, Rebok GW, Brandt J, Tommet D, Marsiske M, Jones RN. Modeling Learning and Memory Using Verbal Learning Tests: Results From ACTIVE. *J Gerontol B Psychol Sci Soc Sci* 2013; **68**: 153–67.

187 Gross AL, Payne BR, Casanova R, et al. The ACTIVE conceptual framework as a structural equation model. *Exp Aging Res* 2018; **44**: 1–17.

188 Guerville F, Souto Barreto P, Giudici KV, Rolland Y, Vellas B. Association of 3-Year Multidomain Intervention and Omega-3 Supplementation with Frailty Incidence. *J Am Geriatr Soc* 2019; **67**: 1700–6.

189 Gunner-Svensson F, Ipsen J, Olsen J, Waldstrom B. Prevention of relocation of the aged in nursing homes. *Scand J Prim Health Care* 1984; **2**: 49–56.

190 Gunzelmann T, Oswald WD, Rupprecht R, Hagen B. Maintaining and supporting independent living in old age (SIMA): III. Sample and selectivity. *Zeitschrift fur Gerontopsychologie und -psychiatrie* 1996; **9**: 83–105.

191 Guralnik JM, Pahor M, Church T, et al. Effect of a Structured Exercise Program on the Overall Burden of Major Mobility Disability Among Older Adults. *Ann Intern Med* 2016; **165**. doi: 10.7326/P16-9023.

192 Guyonnet Sophie G, Vellas B, Sandrine A, Charlotte D, Isabelle C. Mapt study: a 3-year randomized trial of omega 3 and/or multidomain intervention for the prevention of cognitive decline in frail elderly subjects-rationale, design and baseline data. *Alzheimer's & dementia* 2011; **7**: S97-S8.

193 Hagen B, Oswald WD, Rupprecht R. Maintaining and supporting independent living in old age (SIMA)-Part XII: Longitudinal analysis of long-term training effects on independent living and coping with everyday problems. *Zeitschrift fur Gerontopsychologie und -psychiatrie* 1998; **11**: 240–56.
194 Hall WJ. 2012 - Telemonitoring did not reduce hospitalizations or ED visits in high-risk elderly patients. *ACP J Club* 2012; **157**: 1–.
195 Hammar T, Rissanen P, Perala ML. The cost-effectiveness of integrated home care and discharge practice for home care patients. *Health Policy* 2009; **92**: 10–20.
196 Hansen FR, Spedtsberg K, Schroll M. Geriatric follow-up by home visits after discharge from hospital: a randomized controlled trial. *Age Ageing* 1992; **21**: 445–50.
197 Hansen FR, Spedtsberg K, Schroll M. Follow-up home visits to elderly patients after hospitalization. A randomized controlled study. [Danish]. *Ugeskr Laeger* 1994; **156**: 3305–7, 10–11.
198 Hansen FR, Poulsen H, Sorensen KH. A model of regular geriatric follow-up by home visits to selected patients discharged from a geriatric ward: a randomized controlled trial. *Aging Clin Exp Res* 1995; **7**: 202–6.
199 Henderson C, Knapp M, Fernandez JL, et al. Cost effectiveness of telehealth for patients with long term conditions (Whole Systems Demonstrator telehealth questionnaire study): nested economic evaluation in a pragmatic, cluster randomised controlled trial. *BMJ* 2013; **346**: f1035.
200 Henderson C, Knapp M, Fernandez JL, et al. Cost-effectiveness of telecare for people with social care needs: the Whole Systems Demonstrator cluster randomised trial. *Age Ageing* 2014; **43**: 794–800.
201 Henderson RM, Miller ME, Fielding RA, et al. Maintenance of Physical Function 1 Year After Exercise Intervention in At-Risk Older Adults: follow-up From the LIFE Study. *J Gerontol A Biol Sci Med Sci* 2018; **73**: 688-94.
202 Hernandez-Ascanio J, Perula-de Torres LA, Roldan-Villalobos A, Perula-de Torres JC, Rich-Ruiz M, Collaborative Group Asys P. Effectiveness of a multicomponent intervention to reduce social isolation and loneliness in community-dwelling elders: A randomized clinical trial. Study protocol. *J Adv Nurs* 2020; **76**: 337–46. doi: https://dx.doi.org/10.1111/jan.14230.
203 Hinkka K, Karppi SL, Pohjolainen T, Rantanen T, Puukka P, Tilvis R. Network-based geriatric rehabilltation for frail elderly people: Feasibility and effects on subjective health and pain at one year. *J Rehabil Med* 2007; **39**: 473–8.
204 Hirani SP, Beynon M, Cartwright M, et al. The effect of telecare on the quality of life and psychological well-being of elderly recipients of social care over a 12-month period: the Whole Systems Demonstrator cluster randomised trial. *Age Ageing* 2014; **43**: 334–41.
205 Hitzel A, Alonso M, Delrieu J, et al. High prevalence of amyloid positive PET in frailty subjects. *European Journal of Nuclear Medicine and Molecular Imaging* 2011; **2**: S226.
206 Hochhalter AK, Song J, Rush J, Sklar L, Stevens A. Making the Most of Your Healthcare intervention for older adults with multiple chronic illnesses. *Patient Educ Couns* 2010; **81**: 207–13.
207 Hooper C, de Souto Barreto P, Coley N, et al. Cognitive changes with omega-3 polyunsaturated fatty acids in non-demented older adults with low omega-3 index. *J Nutr Health Aging* 2017; **21**: 988–93.
208 Hopp F, Woodbridge P, Subramanian U, Copeland L, Smith D, Lowery J. Outcomes associated with a home care telehealth intervention. *Telemed J E Health* 2006; **12**: 297–307.
209 Hsieh T-J, Su S-C, Chen C-W, et al. "Individualized home-based exercise and nutrition interventions improve frailty in older adults: A randomized controlled trial": Correction. *The International Journal of Behavioral Nutrition and Physical Activity Vol 16 2019, ArtID 136* 2019; **16**.
210 Hsieh T-J, Su S-C, Chen C-W, et al. Individualized home-based exercise and nutrition interventions improve frailty in older adults: a randomized controlled trial. *International Journal of Behavioral Nutrition & Physical Activity* 2019; **16**: N.PAG–N.PAG.
211 Hsin MC, Tsai CH. The effects of functional fitness education program on functional fitness, physical activity on knowledge and self-efficacy of elderly community care center. *Tzu Chi Medical Journal* 2018; **30**: S45–S6.
212 Hsu FC, Rejeski WJ, Ip EH, et al. Evaluation of the late life disability instrument in the Lifestyle Interventions and Independence for Elders Pilot (LIFE-P) study. *Health & Quality of Life Outcomes* 2010; **8**: 115.
213 Hughes SL, Weaver FM, Giobbie-Hurder A, et al. Effectiveness of team-managed home-based primary care: A randomized multicenter trial. *J Am Med Assoc* 2000; **284**: 2877–85.
214 ISRCTN13927531. *HERO - Home-based extended rehabilitation for older people*. BioMed Central: ISRCTN Registry; 2017. URL: http://isrctn.com/ISRCTN13927531 (accessed 11 May 2020).
215 ISRCTN16123291. *Does personalised care planning improve the quality of life of older people with frailty and is it cost-effective?* BioMed Central: ISRCTN Registry; 2020. URL: https://www.isrctn.com/ISRCTN16123291 (accessed 26 August 2021).

216 ISRCTN52788952. *Urban Health Centres Europe, a preventive integrated health and social care approach for active and healthy ageing among community-dwelling older citizens, adapted to five European settings*. World Health Organization: International Clinical Trials Registry Platform; 2017. URL: https://trialsearch.who.int/Trial2.aspx?TrialID=ISRCTN52788952 (accessed 5 November 2020).
217 ISRCTN54268283. *Enabling health and maintaining independence for older people at home: the 'HomeHealth' trial*. BioMed Central: ISRCTN Registry; 2020. URL: http://isrctn.com/ISRCTN54268283 (accessed 26 August 2021).
218 ISRCTN57066881. *'The Home-based Older People's Exercise (HOPE) Programme: An Exercise Programme to Improve the Functional Status of Older People*. BioMed Central: ISRCTN Registry; 2010. URL: http://isrctn.com/ISRCTN57066881 (accessed 5 November 2020).
219 Jackson JL, Scott S, Gutierrez F. Capsule Commentary on Uittenbroek et al., Integrated Care for Older Adults Improves Perceived Quality of Care: Results of a Randomized Controlled Trial of Embrace. *J Gen Intern Med* 2017; **32**: 557.
220 JPRN-UMIN000003877. *Effect of Preventive Home Visits for the Elderly: a Randomized Control Trial*. World Health Organization: International Clinical Trials Registry Platform; 2010. URL: https://trialsearch.who.int/Trial2.aspx?TrialID=JPRN-UMIN000003877 (accessed 5 November 2020).
221 JPRN-UMIN000004767. *Effect of Preventive Home Visits for the Elderly -the Functioning Improvement Tool vs. daily conversation -: a randomized, controlled Trial*. World Health Organization: International Clinical Trials Registry Platform; 2010. URL: https://trialsearch.who.int/Trial2.aspx?TrialID=JPRN-UMIN000004767 (accessed 5 November 2020).
222 JPRN-UMIN000022992. *Algorithm verification of facilities and visit type services for the prevention day care users*. World Health Organization: International Clinical Trials Registry Platform; 2016. URL: https://trialsearch.who.int/Trial2.aspx?TrialID=JPRN-UMIN000022992 (accessed 5 November 2020).
223 JPRN-UMIN000026448. *Evaluation of the effect of supporting social participation through a cosmetic program for senior citizen*. World Health Organization: International Clinical Trials Registry Platform; 2017. URL: https://trialsearch.who.int/Trial2.aspx?TrialID=JPRN-UMIN000026448 (accessed 5 November 2020).
224 June KJ, Lee JY, Yoon JL. Effects of case management using Resident Assessment Instrument-Home Care (RAI-HC) in home health services for older people. [Korean]. *Journal of Korean Academy of Nursing* 2009; **39**: 366–75.
225 Kallio V, Lahtela K, Pietila J, von Wright J, Vuori I. An activation programme for the elderly; base-line characteristics and preliminary evaluation of the programme. *Scand J Soc Med* 1977: 205–20.
226 LIFE Study Investigators. Effects of a physical activity intervention on measures of physical performance: Results of the lifestyle interventions and independence for Elders Pilot (LIFE-P) study. *J Gerontol A Biol Sci Med Sci* 2006; **61**: 1157–65.
227 Kerr M, Cramond J, Scott M. Service coordinators in the NECC trial: An RDNS perspective. *Australian Journal of Primary Health - Interchange* 2000; **6**: 104–7.
228 Kerse NM, Flicker L, Jolley D, Arroll B, Young D. Improving the health behaviours of elderly people: randomised controlled trial of a general practice education programme. *BMJ* 1999; **319**: 683–7.
229 Kerse N, Elley CR, Robinson E, Arroll B. Is physical activity counseling effective for older people? A cluster randomized, controlled trial in primary care. *J Am Geriatr Soc* 2005; **53**: 1951–6.
230 Kim CO, Jeong Y, Park Y, et al. Reinforcement Effects of Social Network Intervention during Nutritional Supplementation in Frail Older Adults. *Gerontology.* 2021. doi: 10.1159/000514676.
231 Kim H, Kojima N, Uchida R, et al. The additive effects of exercise and essential amino acid on muscle mass and strength in community-dwelling older Japanese women with muscle mass decline, but not weakness and slowness: a randomized controlled and placebo trial. *Aging Clin Exp Res* 2021; **33**: 1841–52. doi: https://dx.doi.org/10.1007/s40520-020-01713-x.
232 King AII, Boyd ML, Raphael DL, Jull A. The effect of a gerontology nurse specialist for high needs older people in the community on healthcare utilisation: a controlled before-after study. *BMC Geriatr* 2018; **18**: 22.
233 Kinney ED, Kennedy J, Cook CA, Freedman JA, Lane KA, Hui SL. A randomized trial of two quality improvement strategies implemented in a statewide public community-based, long-term care program. *Med Care* 2003; **41**: 1048–57.
234 Katula JA, Kritchevsky SB, Guralnik JM, et al. Lifestyle Interventions and Independence for Elders Pilot Study: recruitment and baseline characteristics. *J Am Geriatr Soc* 2007; **55**: 674–83.
235 Kivipelto M, Mangialasche F, Ngandu T. Lifestyle interventions to prevent cognitive impairment, dementia and Alzheimer disease. *Nature Reviews Neurology* 2018; **14**: 653–66.
236 Kivipelto M, Mangialasche F, Ngandu T. World Wide Fingers will advance dementia prevention. *The Lancet Neurology* 2018; **17**: 27.

237 Kivipelto M, Mangialsche F, Ngandu T, Solomon A, Tuomilehto J, Soininen H. From the finnish geriatric intervention study to prevent cognitive impairment and disability to the global dementia prevention initiative: applicability of multidomain interventions. *Alzheimer's & dementia* 2017; **13**: P1221-.
238 Kolbe-Alexander TL, Charlton KE, Lambert EV. Effectiveness of a community basedlow intensity exercise program for older adults. *J Nutr Health Aging* 2006; **10**: 21–9.
239 Kravitz RL, Reuben DB, Davis JW, et al. Geriatric home assessment after hospital discharge. *J Am Geriatr Soc* 1994; **42**: 1229–34.
240 Kristensson J, Ekwall AK, Jakobsson U, Midlöv P, Hallberg IR. Case managers for frail older people: a randomised controlled pilot study. *Scand J Caring Sci* 2010; **24**: 755–63.
241 Kwon J, Yoshida Y, Yoshida H, Kim H, Suzuki T, Lee Y. Effects of a combined physical training and nutrition intervention on physical performance and health-related quality of life in prefrail older women living in the community: a randomized controlled trial. *J Am Med Dir Assoc* 2015; **16**: 263.e1-8.
242 Klompstra L, Ekdahl AW, Krevers B, Milberg A, Eckerblad J. Factors related to health-related quality of life in older people with multimorbidity and high health care consumption over a two-year period. *BMC Geriatr* 2019; **19**: 187.
243 Lewin G, Calver J, McCormack B, et al. The Home Independence Project. *Geriaction* 2008; **26**: 13–21.
244 Latham NK, Anderson CS, Lee A, et al. A randomized, controlled trial of quadriceps resistance exercise and vitamin D in frail older people: the Frailty Interventions Trial in Elderly Subjects (FITNESS). *J Am Geriatr Soc* 2003; **51**: 291–9.
245 Li CM, Chen CY, Li CY, Wang WD, Wu SC. The effectiveness of a comprehensive geriatric assessment intervention program for frailty in community-dwelling older people: a randomized, controlled trial. *Arch Gerontol Geriatr* 2010; **50**: S39–42.
246 Liang HY, Hann Lin L, Yu Chang C, Mei Wu F, Yu S. Effectiveness of a Nurse-Led Tele-Homecare Program for Patients With Multiple Chronic Illnesses and a High Risk for Readmission: A Randomized Controlled Trial. *J Nurs Scholarsh* 2021; **53**: 161–70. doi: https://dx.doi.org/10.1111/jnu.12622.
247 Lihavainen K, Sipila S, Rantanen T, Hartikainen S. Effect of comprehensive geriatric assessment and individualized intervention on mobility among older persons with and without pain. *Eur J Pain Suppl* 2011; **5**: 246.
248 Lilamand M, Cesari M, Cantet C, et al. Relationship Between Brain Amyloid Deposition and Instrumental Activities of Daily Living in Older Adults: A Longitudinal Study from the Multidomain Alzheimer Prevention Trial. *J Am Geriatr Soc* 2018; **66**: 1940–7.
249 Lim WK, Lambert SF, Gray LC. Effectiveness of case management and post-acute services in older people after hospital discharge. *Med J Aust* 2003; **178**: 262–6.
250 Lin F, Chen DG, Vance DE, Ball KK, Mapstone M. Longitudinal relationships between subjective fatigue, cognitive function, and everyday functioning in old age. *Int Psychogeriatr* 2013; **25**: 275–85.
251 Lin F, Chen D-G, Vance D, Mapstone M. Trajectories of Combined Laboratory- and Real World-Based Speed of Processing in Community-Dwelling Older Adults. *J Gerontol B Psychol Sci Soc Sci* 2013; **68**: 364–73.
252 Liu CK, Leng X, Hsu FC, et al. The impact of sarcopenia on a physical activity intervention: the Lifestyle Interventions and Independence for Elders Pilot Study (LIFE-P). *J Nutr Health Aging* 2014; **18**: 59-64.
253 Liu CJ, Xu H, Keith NR, Clark DO. Promoting ADL independence in vulnerable, community-dwelling older adults: A pilot RrCT comparing 3-Sstep Workout for Llife versus resistance exercise. *Clin Interv Aging* 2017; **12**: 1141–9.
254 Liu M, Xue QL, Gitlin LN, et al. Disability Prevention Program Improves Life-Space and Falls Efficacy: A Randomized Controlled Trial. *J Am Geriatr Soc* 2020; **20**: 20. doi: https://dx.doi.org/10.1111/jgs.16808.
255 Lohman MC, Rebok GW, Spira AP, Parisi JM, Gross AL, Kueider AM. Depressive Symptoms and Memory Performance Among Older Adults: Results From the ACTIVE Memory Training Intervention. *J Aging Health* 2013; **25**: 209S–29S.
256 Lorig KR, Sobel DS, Stewart AL, et al. Evidence suggesting that a chronic disease self-management program can improve health status while reducing hospitalization: a randomized trial. *Med Care* 1999; **37**: 5–14.
257 Luger E, Dorner TE, Haider S, Kapan A, Lackinger C, Schindler K. Effects of a Home-Based and Volunteer-Administered Physical Training, Nutritional, and Social Support Program on Malnutrition and Frailty in Older Persons: a Randomized Controlled Trial. *J Am Med Dir Assoc* 2016; **17**: 671.e9–16. doi: 10.1016/j.jamda.2016.04.018.
258 Lum H, Sudore R, Kutner J, Levy C. Effectiveness of advance care planning group visits for older adults in primary care. *J Am Geriatr Soc* 2020; **68 (SUPPL 1)**: S219–S20. doi: 10.1111/jgs.16431.
259 Lurie N, Christianson J, Finch M, Moscovice I. The effects of capitation on health and functional status of the Medicaid elderly. A randomized trial. *Ann Intern Med* 1994; **120**: 506–11.

260 Lyndon H, Latour JM, Marsden J, Campbell S, Stevens K, Kent B. The holistic assessment and care planning in partnership intervention study (HAPPI): A protocol for a feasibility, cluster randomized controlled trial. *J Adv Nurs* 2019; **75**: 3078–87. doi: 10.1111/jan.14106.
261 ISRCTN74345449. *The HAPPI Study: developing and testing an assessment and care plan to support older people who live with frailty at home*. BioMed Central: ISRCTN Registry; 2018. URL: https://doi.org/10.1186/ISRCTN74345449
http://www.who.int/trialsearch/Trial2.aspx?TrialID=ISRCTN74345449 (accessed 11 May 2020).
262 Mangin D, Lamarche L, Oliver D, et al. Health TAPESTRY Ontario: protocol for a randomized controlled trial to test reproducibility and implementation. *Trials [Electronic Resource]* 2020; **21**: 714. doi: https://dx.doi.org/10.1186/s13063-020-04600-y.
263 Mankowski RT, Anton SD, Axtell R, et al. Device-Measured Physical Activity As a Predictor of Disability in Mobility-Limited Older Adults. *J Am Geriatr Soc* 2017; **65**: 2251–6.
264 Marcusson J, Nord M, Johansson MM, et al. Proactive healthcare for frail elderly persons: study protocol for a prospective controlled primary care intervention in Sweden. *BMJ open* 2019; **9**.
265 Marsh AP, Lovato LC, Glynn NW, et al. Lifestyle interventions and independence for elders study: recruitment and baseline characteristics. *J Gerontol A Biol Sci Med Sci* 2013; **68**: 1549-58.
266 Marsh AP, Applegate WB, Guralnik JM, et al. Hospitalizations During a Physical Activity Intervention in Older Adults at Risk of Mobility Disability: analyses from the Lifestyle Interventions and Independence for Elders Randomized Clinical Trial. *J Am Geriatr Soc* 2016; **64**: 933-43.
267 Martin F, Oyewole A, Moloney A. A randomized controlled trial of a high support hospital discharge team for elderly people. *Age Ageing* 1994; **23**: 228–34.
268 Martin Lesende I. Geriatric evaluation and home care for elderly people with a low income. [Spanish]. *FMC Formacion Medica Continuada en Atencion Primaria* 2008; **15**: 483.
269 Matthews MM, Hsu FC, Walkup MP, Barry LC, Patel KV, Blair SN. Depressive symptoms and physical performance in the lifestyle interventions and independence for elders pilot study. *J Am Geriatr Soc* 2011; **59**: 495-500.
270 Mayer JA, Jermanovich A, Wright BL, Elder JP, Drew JA, Williams SJ. Changes in health behaviors of older adults: The San Diego Medicare Preventive Health Project. *Preventive Medicine: An International Journal Devoted to Practice and Theory* 1994; **23**: 127–33.
271 McDermott MM, Applegate WB, Bonds DE, et al. Ankle brachial index values, leg symptoms, and functional performance among community-dwelling older men and women in the lifestyle interventions and independence for elders study. *J Am Heart Assoc* 2013; **2**: e000257.
272 McDougall GJ, Becker H, Pituch K, Acee TW, Vaughan PW, Delville CL. Differential benefits of memory training for minority older adults in the SeniorWISE study. *Gerontologist* 2010; **50**: 632-45.
273 McDowell BJ, Engberg S, Sereika S, et al. Effectiveness of behavioral therapy to treat incontinence in homebound older adults. *J Am Geriatr Soc* 1999; **47**: 309–18.
274 McEwan RT, Davison N, Forster DP, Pearson P, Stirling E. Screening elderly people in primary care: a randomized controlled trial. *Br J Gen Pract* 1990; **40**: 94–7.
275 McFarland ML. The effect of the provision of inhome services on the elderly person's informal support network. *Dissertation Abstracts International* 1991; **52**: 1079–80.
276 McMurdo MET, Johnstone R. A randomized controlled trial of a home exercise programme for elderly people with poor mobility. *Age Ageing* 1995; **24**: 425–8.
277 McWilliam CL, Stewart M, Brown JB, et al. Home-based health promotion for chronically ill older persons: Results of a randomized controlled trial of a critical reflection approach. *Health Promot Int* 1999; **14**: 27–41.
278 Meiling C. Langzeitwirkung präventiver Maßnahmen zum Erhalt der Selbstständigkeit in den ADL bei betagten Menschen. *Ergotherapie & Rehabilitation* 2012; **51**: 23–5.
279 Melin AL, Bygren LO. Efficacy of the rehabilitation of elderly primary health care patients after short-stay hospital treatment. *Med Care* 1992; **30**: 1004–15.
280 Melin AL, Bygren LO. Perceived functional health of frail elderly in a primary home care programme and correlation of self-perception with objective measurements. *Scand J Soc Med* 1993; **21**: 256–63.
281 Melin AL, Hakansson S, Bygren LO. The cost-effectiveness of rehabilitation in the home: a study of Swedish elderly. *Am J Public Health* 1993; **83**: 356–62.
282 Merete Pedersen M, Petersen J, Beyer N, Damkjær L, Bandholm T, Pedersen MM. Supervised progressive cross-continuum strength training compared with usual care in older medical patients: study protocol for a randomized controlled trial (the STAND-Cph trial). *Trials* 2016; **17**: 1–16.
283 Meuleman J. A controlled trial of geriatric evaluation. *N Engl J Med* 2002; **347**: 371–3; author reply –3.
284 Meziere A. Exercise inerventions with trained carers for preventing loss of autonomy and falls in elderly people at home (T4H): A cluster randomized controlled pilot trial. *Eur Geriatr Med* 2019; **10 (Supplement 1)**: S177–S8. doi: 10.1007/s41999-019-00221-0.

285 Miller P, Gladman JR, Cunliffe AL, Husbands SL, Dewey ME, Harwood RH. Economic analysis of an early discharge rehabilitation service for older people. *Age Ageing* 2005; **34**: 274–80.
286 Mohd Suffian NI, Adznam SN, Abu Saad H, et al. Frailty Intervention through Nutrition Education and Exercise (FINE). A health promotion intervention to prevent frailty and improve frailty status among pre-frail elderly-a study protocol of a cluster randomized controlled trial. *Nutrients* 2020; **12**: 10. doi: 10.3390/nu12092758.
287 Moller UO, Kristensson J, Midlov P, Ekdahl C, Jakobsson U. Effects of a one-year home-based case management intervention on falls in older people: a randomized controlled trial. *J Aging Phys Act* 2014; **22**: 457–64.
288 Moon SY, Hong CH, Jeong JH, et al. Facility-based and home-based multidomain interventions including cognitive training, exercise, diet, vascular risk management, and motivation for older adults: a randomized controlled feasibility trial. *Aging* 2021; **13**: 15898–916. doi: 10.18632/aging.203213.
289 Mor V, Granger CV, Sherwood CC. Discharged rehabilitation patients: impact of follow-up surveillance by a friendly visitor. *Arch Phys Med Rehabil* 1983; **64**: 346–53.
290 Mortenson WB, Demers L, Fuhrer MJ, Jutai JW, Lenker J, Deruyter F. Effects of an Assistive Technology Intervention on Older Adults with Disabilities and Their Informal Caregivers. *Am J Phys Med Rehabil* 2013; **92**: 297–306.
291 Mortsiefer A, Wilm S, Santos S, et al. Family conferences and shared prioritisation to improve patient safety in the frail elderly (COFRAIL): study protocol of a cluster randomised intervention trial in primary care. *Trials* 2020; **21**: 285. doi: 10.1186/s13063-020-4182-x.
292 DRKS00015055. *Family conferences and shared prioritization to improve patient safety in the frail elderly*. World Health Organization: International Clinical Trials Registry Platform; 2019. URL: https://trialsearch.who.int/Trial2.aspx?TrialID=DRKS00015055 (accessed 11 May 2020).
293 Mountain G, Chatters R. The "Lifestyle Matters" Study: Results From a Trial of an Occupational Therapy Lifestyle Intervention for Older Adults. *Am J Occup Ther* 2016; **70**: 1–.
294 Mugueta-Aguinaga I, Garcia-Zapirain B. FRED: exergame to Prevent Dependence and Functional Deterioration Associated with Ageing. A Pilot Three-Week Randomized Controlled Clinical Trial. *Int J Environ Res Public Health* 2017; **14**.
295 Mugueta-Aguinaga I, Garcia-Zapirain B. Frailty level monitoring and analysis after a pilot six-week randomized controlled clinical trial using the FRED exergame including biofeedback supervision in an elderly day care centre. *Int J Environ Res Public Health* 2019; **16**.
296 NCT00452465. *Primary Healthcare for Community Living Old Elderly*. US National Library of Medicine: ClinicalTrials.gov; 2007. URL: https://clinicaltrials.gov/ct2/show/study/NCT00452465 (accessed 23 August 2020).
297 NCT00672685. *Omega-3 Fatty Acids and/or Multi-domain Intervention in the Prevention of Age-related Cognitive Decline*. US National Library of Medicine: ClinicalTrials.gov; 2008. URL: https://ClinicalTrials.gov/show/NCT00672685 (accessed 5 November 2020).
298 NCT01345032. *The Effect of Nutrition Follow up After Hospital Discharge in Undernourished Elderly*. US National Library of Medicine: ClinicalTrials.gov; 2011. URL: https://clinicaltrials.gov/show/NCT01345032 (accessed 5 November 2020).
299 NCT02021565. *Evaluating the CG ASSIST Program for Caregiving Dyads*. US National Library of Medicine: ClinicalTrials.gov; 2013. URL: https://clinicaltrials.gov/show/NCT02021565 (accessed 5 November 2020).
300 NCT02335177. *Technology and Human Help at Home After Hospitalisation*. US National Library of Medicine: ClinicalTrials.gov; 2015. URL: https://clinicaltrials.gov/show/NCT02335177 (accessed 5 November 2020).
301 NCT02545257. *Development of a Coordinated, Community-Based Medication Management Model for Home-Dwelling Aged in Primary Care*. US National Library of Medicine: ClinicalTrials.gov; 2015. URL: https://clinicaltrials.gov/show/NCT02545257 (accessed 5 November 2020).
302 NCT02554838. *How to Act on the Mobility Restriction Linked to Senior's Phobia of Falling*. US National Library of Medicine: ClinicalTrials.gov; 2015. URL: https://clinicaltrials.gov/show/NCT02554838 (accessed 5 November 2020).
303 NCT02582138. *Multicomponent Intervention for Physical Frailty and Sarcopenia*. US National Library of Medicine: ClinicalTrials.gov; 2016. URL: https://ClinicalTrials.gov/show/NCT02582138 (accessed 5 November 2020).
304 NCT02847871. *Elderly Patient at Risk of Loss of Mobility, Exercise - Primary Care, Prevention, Care Pathways*. US National Library of Medicine: ClinicalTrials.gov; 2016. URL: https://clinicaltrials.gov/show/NCT02847871 (accessed 5 November 2020).

305 NCT02923843. *Are Geriatric Out-patient Care Better Than Usual Care?* US National Library of Medicine: ClinicalTrials.gov; 2016. URL: https://clinicaltrials.gov/show/NCT02923843 (accessed 5 November 2020).
306 NCT02942992. *Implementing Resistance Exercise to Reduce Frailty for Older Adult*. US National Library of Medicine: ClinicalTrials.gov; 2017. URL: https://ClinicalTrials.gov/show/NCT02942992 (accessed 5 November 2020).
307 NCT03147625. *Salutogenic Healthy Aging Program Embracement (SHAPE) for Elderly-only Households*. US National Library of Medicine: ClinicalTrials.gov; 2017. URL: https://clinicaltrials.gov/show/NCT03147625 (accessed 11 May 2020).
308 NCT03180606. *Enhanced Primary Care for Elderly*. US National Library of Medicine: ClinicalTrials.gov; 2017. URL: https://clinicaltrials.gov/show/NCT03180606 (accessed 5 November 2020).
309 NCT03212859. *Coach2Move: Sustainable in Daily Practice*. US National Library of Medicine: ClinicalTrials.gov; 2017. URL: https://ClinicalTrials.gov/show/NCT03212859 (accessed 5 November 2020).
310 NCT03336320. *Feasibility and Acceptability of a Web-based Multidomain Intervention to Maintain Cognitive Function in Older Adults*. US National Library of Medicine: ClinicalTrials.gov; 2017. URL: https://clinicaltrials.gov/show/NCT03336320 (accessed 5 November 2020).
311 NCT03342976. *Investigating My Active and Healthy Aging*. US National Library of Medicine: ClinicalTrials.gov; 2018. URL: https://ClinicalTrials.gov/show/NCT03342976 (accessed 5 November 2020).
312 NCT03394495. *Individualized Exercise Program Plus Behavioral Change Enhancement Strategies for Managing Fatigue in Frail Older People*. US National Library of Medicine: ClinicalTrials.gov; 2018. URL: https://clinicaltrials.gov/show/NCT03394495 (accessed 5 November 2020).
313 NCT03394534. *Comprehensive Geriatric Assessment in Primary Care: A Randomised Feasibility Trial*. US National Library of Medicine: ClinicalTrials.gov; 2018. URL: https://ClinicalTrials.gov/show/NCT03394534 (accessed 11 May 2020).
314 NCT03456128. *Reducing Disability Following Hospital Discharge in Vulnerable Older Adults: the CAPABLE Intervention*. US National Library of Medicine: ClinicalTrials.gov; 2018. URL: https://clinicaltrials.gov/show/NCT03456128 (accessed 11 May 2020).
315 NCT03474380. *Optimizing Function and Independence Through iHI-FIVES*. US National Library of Medicine: ClinicalTrials.gov; 2018. URL: https://clinicaltrials.gov/show/NCT03474380 (accessed 5 November 2020).
316 NCT03568084. *Effectiveness of an Intervention in Multicomponent Exercise in Patients Over 70 Years in Primary Care*. US National Library of Medicine: ClinicalTrials.gov; 2018. URL: https://ClinicalTrials.gov/show/NCT03568084 (accessed 5 November 2020).
317 NCT03577002. *Team-based Versus Primary Care Clinician-led Advance Care Planning in Practice-based Research Networks*. US National Library of Medicine: ClinicalTrials.gov; 2019. URL: https://ClinicalTrials.gov/show/NCT03577002 (accessed 5 November 2020).
318 NCT03591055. *Frailty Assessment and Treatment Strategies in the Elderly at Risk of Functional Decline in the Community*. US National Library of Medicine: ClinicalTrials.gov; 2018. URL: https://ClinicalTrials.gov/show/NCT03591055 (accessed 11 May 2020).
319 NCT03634033. *Implementation of CAPABLE in the Michigan Medicaid Waiver*. US National Library of Medicine: ClinicalTrials.gov; 2018. URL: https://clinicaltrials.gov/show/NCT03634033 (accessed 5 November 2020).
320 NCT03649698. *Exercise and Nutrition for Healthy AgeiNg*. US National Library of Medicine: ClinicalTrials.gov; 2018. URL: https://ClinicalTrials.gov/show/NCT03649698 (accessed 11 May 2020).
321 NCT03797352. *HOPE (Healthy Older People Everyday) To Age in Place*. US National Library of Medicine: ClinicalTrials.gov; 2019. URL: https://ClinicalTrials.gov/show/NCT03797352 (accessed 5 November 2020).
322 NCT03814161. *FRailty Among Elderly Emergency Department Patients With Outcome Measures*. US National Library of Medicine: ClinicalTrials.gov; 2019. URL: https://ClinicalTrials.gov/show/NCT03814161 (accessed 5 November 2020).
323 NCT03824106. *Frailty Rehabilitation*. US National Library of Medicine: ClinicalTrials.gov; 2019. URL: https://ClinicalTrials.gov/show/NCT03824106 (accessed 11 May 2020).
324 NCT03952858. *Telerehabilitation in Geriatric Patients at Aarhus University Hospital, Denmark*. US National Library of Medicine: ClinicalTrials.gov; 2019. URL: https://clinicaltrials.gov/show/NCT03952858 (accessed 5 November 2020).
325 NCT03979560. *Clinical and Medico-economic Evaluation of Taken Care Associating Nutritional Support and Adapted Physical Activity for Malnourished, or at Risk of Undernutrition, Elderly People on*

Discharge From Hospital. US National Library of Medicine: ClinicalTrials.gov; 2019. URL: https://clinicaltrials.gov/show/NCT03979560 (accessed 11 May 2020).
326 NCT04076319. *Implementing CAPABLE*. US National Library of Medicine: ClinicalTrials.gov; 2019. URL: https://ClinicalTrials.gov/show/NCT04076319 (accessed 5 November 2020).
327 NCT04416815. *Integrating Health Promotion With and for Older People - eHealth*. US National Library of Medicine: ClinicalTrials.gov; 2020. URL: https://clinicaltrials.gov/show/NCT04416815 (accessed 6 November 2020).
328 NCT04460742. *CAPABLE Transitions: a Home Health Agency-Based Intervention to Optimize the SNF-to-Home Transition*. US National Library of Medicine: ClinicalTrials.gov; 2020. URL: https://clinicaltrials.gov/show/NCT04460742 (accessed 6 November 2020).
329 NCT04500366. *GERAS Frailty Rehabilitation at Home During COVID-19*. US National Library of Medicine: ClinicalTrials.gov; 2020. URL: https://clinicaltrials.gov/show/NCT04500366 (accessed 6 November 2020).
330 NCT04531852. *Community-based Complex Intervention to Prevent Loss of Physical Function and Disability in Home-dwelling Older Adults*. US National Library of Medicine: ClinicalTrials.gov; 2020. URL: https://clinicaltrials.gov/show/NCT04531852 (accessed 26 August 2021).
331 NCT04574271. *Multidimensional Intervention in Pre-frail Patients Older Than 70 Years*. US National Library of Medicine: ClinicalTrials.gov; 2019. URL: https://ClinicalTrials.gov/show/NCT04574271 (accessed 26 August 2021).
332 NCT04628754. *A Randomized Control Trial of an Intervention to Reverse Frailty and Enhance Resilience Through Exercise and Education*. US National Library of Medicine: ClinicalTrials.gov; 2020. URL: https://ClinicalTrials.gov/show/NCT04628754 (accessed 26 August 2021).
333 Nelson ME, Layne JE, Bernstein MJ, et al. The effects of multidimensional home-based exercise on functional performance in elderly people. *J Gerontol A Biol Sci Med Sci* 2004; **59**: 154-60.
334 Neumann L, Dapp U, von Renteln-Kruse W, Minder CE. Health promotion and preventive care intervention for older community-dwelling people: Long-term effects of a randomised controlled trial (RCT) within the LUCAS cohort. *J Nutr Health Aging* 2017; **21**: 1016–23.
335 Newbury J, Marley J. Preventive home visits to elderly people in the community. Visits are most useful for people aged >/= 75. *BMJ (Clinical research ed.)* 2000; **321**: 512; discussion 3.
336 Ngandu T, Kulmala J, Antikainen R, et al. USE OF HEALTH CARE SERVICES AMONG OLDER ADULTS PARTICIPATING IN A MULTIDOMAIN LIFESTYLE INTERVENTION TO PREVENT COGNITIVE IMPAIRMENT (FINGER). *Alzheimer's & dementia* 2018; **14**: P1363-.
337 Nice CHUd. Safe & Easy Environment at Home for Patients Presenting a Frail Syndrome. https://ClinicalTrials.gov/show/NCT02288221; 2015.
338 Nicklas BJ, Hsu FC, Brinkley TJ, et al. Exercise training and plasma C-reactive protein and interleukin-6 in elderly people. *J Am Geriatr Soc* 2008; **56**: 2045–52.
339 Nielsen M, Blenkner M, Bloom M, Downs T, Beggs H. Older persons after hospitalization: a controlled study of home aide service. *Am J Public Health* 1972; **62**: 1094–101.
340 O'Connell M, Sint K, Rosenheck R. How do housing subsidies improve quality of life among homeless adults? A mediation analysis. *Am J Community Psychol* 2018; **61**: 433–44.
341 O'Connor C, Clemson L, Brodaty H, et al. Increasing activity engagement and improving carer management skills in FTD: two case studies using the Tailored Activities Program (TAP). *American journal of neurodegenerative diseases* 2014; **3**: 55-.
342 O'Connor CM, Clemson L, Brodaty H, et al. The tailored activities program (TAP) for the management of frontotemporal dementia: a case study. *Alzheimer's & dementia* 2015; **11**: P837-P8.
343 Oksman E, Linna M, Horhammer I, Lammintakanen J, Talja M. Cost-effectiveness analysis for a tele-based health coaching program for chronic disease in primary care. *BMC Health Serv Res* 2017; **17**: 138.
344 Olesen C, Harbig P, Buus KM, Barat I, Damsgaard EM. Impact of pharmaceutical care on adherence, hospitalisations and mortality in elderly patients. *Int J Clin Pharm* 2014; **36**: 163–71.
345 Oliva NL. A closer look at nurse case management of community-dwelling older adults: observations from a longitudinal study of care coordination in the chronically ill. *Prof Case Manag* 2010; **15**: 90–100.
346 Ollonqvist K, Gronlund R, Karppi S-L, Salmelainen U, Poikkeus L, Hinkka K. A network-based rehabilitation model for frail elderly people: Development and assessment of a new model. *Scand J Caring Sci* 2007; **21**: 253–61.
347 Ollonqvist K, Aaltonen T, Karppi SL, Hinkka K, Pontinen S. Network-based rehabilitation increases formal support of frail elderly home-dwelling persons in Finland: randomised controlled trial. *Health Soc Care Community* 2008; **16**: 115–25.
348 Olsson IN, Runnamo R, Engfeldt P. Drug treatment in the elderly: an intervention in primary care to enhance prescription quality and quality of life. *Scand J Prim Health Care* 2012; **30**: 3–9.

349 Olsson Möller U, Jakobsson U. Predictive validity and cut-off scores in four diagnostic tests for falls-a study in frail older people at home. *Eur Geriatr Med* 2012; **3**: S49-.
350 Olsson Möller U, Kristensson J, Midlöv P, Ekdahl C, Jakobsson U. Predictive Validity and Cut-Off Scores in Four Diagnostic Tests for Falls - A Study in Frail Older People at Home. *Phys Occup Ther Geriatr* 2012; **30**: 189–201.
351 Opdenacker J, Boen F, Coorevits N, Delecluse C. Effectiveness of a lifestyle intervention and a structured exercise intervention in older adults. *Prev Med* 2008; **46**: 518–24.
352 Opdenacker J, Delecluse C, Boen F. A 2-year follow-up of a lifestyle physical activity versus a structured exercise intervention in older adults. *J Am Geriatr Soc* 2011; **59**: 1602–11.
353 Ory MG, Lipman PD, Karlen PL, et al. Recruitment of older participants in frailty/injury prevention studies. *Prev Sci* 2002; **3**: 1–22.
354 Osborn DP, Fletcher AE, Smeeth L, et al. Geriatric Depression Scale Scores in a representative sample of 14 545 people aged 75 and over in the United Kingdom: results from the MRC Trial of Assessment and Management of Older People in the Community. *Int J Geriatr Psychiatry* 2002; **17**: 375–82.
355 Osborn DP, Fletcher AE, Smeeth L, et al. Factors associated with depression in a representative sample of 14 217 people aged 75 and over in the United Kingdom: results from the MRC trial of assessment and management of older people in the community. *Int J Geriatr Psychiatry* 2003; **18**: 623–30.
356 Osborn DP, Fletcher AE, Smeeth L, et al. Performance of a single screening question for depression in a representative sample of 13 670 people aged 75 and over in the UK: results from the MRC trial of assessment and management of older people in the community. *Fam Pract* 2003; **20**: 682–4.
357 Oswald WD, Rupprecht R, Hagen B, Fleischmann U. Maintaining and supporting independent living in old age (SIMA): IV. Results after one year of intervention. *Zeitschrift fur Gerontopsychologie und -psychiatrie* 1996; **9**: 107–44.
358 Oswald WD, Hagen B, Rupprecht R. Maintaining and supporting independent living in old age (SIMA)-Part X: Longitudinal analysis of long-term training effects on the cognitive status. *Zeitschrift fur Gerontopsychologie und -psychiatrie* 1998; **11**: 202–21.
359 Oswald WD, Hagen B, Rupprecht R, Gunzelmann T. Maintaining and supporting independent living in old age (SIMA). Part XVII: Summary of long-term training effects. *Zeitschrift fur Gerontopsychologie und -psychiatrie* 2002; **15**: 13–31.
360 Oswald WD, Gunzelmann T, Rupprecht R, Hagen B. Differential effects of single versus combined cognitive and physical training with older adults: The SimA study in a 5-year perspective. *Eur J Ageing* 2006; **3**: 179–92.
361 Overbeek A, Korfage IJ, Jabbarian LJ, et al. Advance Care Planning in Frail Older Adults: A Cluster Randomized Controlled Trial. *J Am Geriatr Soc* 2018; **66**: 1089–95.
362 Overbeek A, Polinder S, Haagsma J, et al. Advance Care Planning for frail older adults: Findings on costs in a cluster randomised controlled trial. *Palliat Med* 2019; **33**: 291–300.
363 Pacini M, Smith RD, Wilson ECF, Holland R. Home-based medication review in older people: Is it cost effective? *Pharmacoeconomics* 2007; **25**: 171–80.
364 Palacholla RS, Fischer NC, Agboola S, et al. Evaluating the Impact of a Web-Based Risk Assessment System (CareSage) and Tailored Interventions on Health Care Utilization: Protocol for a Randomized Controlled Trial. *JMIR Research Protocols* 2018; **7**: e10045.
365 Pardessus V, Puisieux F, Di Pompeo C, Gaudefroy C, Thevenon A, Dewailly P. Benefits of home visits for falls and autonomy in the elderly: A randomized trial study. *American Journal of Physical Medicine and Rehabilitation* 2002; **81**: 247–52.
366 Parsons M, Parsons J, Pillai A, et al. Post-Acute Care for Older People Following Injury: A Randomized Controlled Trial. *J Am Med Dir Assoc* 2020; **21**: 404–. doi: 10.1016/j.jamda.2019.08.015.
367 Peak T, Toseland RW, Banks SM. The impact of a spouse-caregiver support group on care recipient health care costs. *J Aging Health* 1995; **7**: 427–49.
368 Pedersen JL, Pedersen PU, Damsgaard EM. Early Nutritional Follow-Up after Discharge Prevents Deterioration of ADL Functions in Malnourished, Independent, Geriatric Patients Who Live Alone - A Randomized Clinical Trial. *J Nutr Health Aging* 2016; **20**: 845–53.
369 Peri K, Broad JB, Hikaka J, et al. Study protocol: older people in retirement villages. A survey and randomised trial of a multi-disciplinary invention designed to avoid adverse outcomes. *BMC Geriatr* 2020; **20**: 247. doi: 10.1186/s12877-020-01640-6.
370 Perkel RL, Plumb JD. The Urban Community Family Medicine Home Visit Program at Thomas Jefferson University Hospital. *Pride Inst J Long Term Home Health Care* 1983; **2**: 10–7.
371 Perman G, Prevettoni M, Guenzelovich T, et al. Effectiveness of a health and social care integration programme for home-dwelling frail older persons in Argentina. *International Journal of Care Coordination* 2021; **24**: 28–34. doi: 10.1177/20534345211002114.

372 Persson M, Hytter-Landahl A, Brismar K, Cederholm T. Nutritional supplementation and dietary advice in geriatric patients at risk of malnutrition. *Clinical nutrition (edinburgh, scotland)* 2007; **26**: 216-24.

373 Petersson I, Kottorp A, Bergstrm J, Lilja M. Longitudinal changes in everyday life after home modifications for people aging with disabilities. *Scand J Occup Ther* 2009; **16**: 78–87.

374 Phillips EM, Katula J, Miller ME, et al. Interruption of physical activity because of illness in the Lifestyle Interventions and Independence for Elders Pilot trial. *J Aging Phys Act* 2010; **18**: 61-74.

375 Picarsic JL, Glynn NW, Taylor CA, et al. Self-reported napping and duration and quality of sleep in the lifestyle interventions and independence for elders pilot study. *J Am Geriatr Soc* 2008; **56**: 1674-80.

376 Prossegger J, Huber D, Grafetstatter C, et al. Effects of moderate mountain hiking and balneotherapy on community-dwelling older people: A randomized controlled trial. *Exp Gerontol* 2019; **122**: 74–84. doi: https://dx.doi.org/10.1016/j.exger.2019.04.006.

377 Rantanen T, Hassandra M, Pynnonen K, et al. The effect of individualized, theory-based counselling intervention on active aging and quality of life among older people (the AGNES intervention study). *Aging Clin Exp Res* 2020; **32**: 2081–90. doi: https://dx.doi.org/10.1007/s40520-020-01535-x.

378 Rebok GW, Langbaum JBS, Jones RN, et al. Memory Training in the ACTIVE Study: How Much is Needed and Who Benefits? *J Aging Health* 2013; **25**: 21S–42S.

379 Rebok GW, Ball K, Guey LT, et al. Ten-year effects of the advanced cognitive training for independent and vital elderly cognitive training trial on cognition and everyday functioning in older adults. *J Am Geriatr Soc* 2014; **62**: 16–24.

380 Rejeski WJ, Marsh AP, Chmelo E, et al. The Lifestyle Interventions and Independence for Elders Pilot (LIFE-P): 2-year follow-up. *J Gerontol A Biol Sci Med Sci* 2009; **64**: 462-7.

381 Rejeski WJ, Axtell R, Fielding R, et al. Promoting physical activity for elders with compromised function: The lifestyle interventions and independence for elders (LIFE) study physical activity intervention. *Clin Interv Aging* 2013; **8**: 1119–31.

382 Reuben DB, Frank JC, Hirsch SH, McGuigan KA, Maly RC. A randomized clinical trial of outpatient comprehensive geriatric assessment coupled with an intervention to increase adherence to recommendations. *J Am Geriatr Soc* 1999; **47**: 269–76.

383 Reuben DB. Meeting the Needs of Disabled Older Persons: Can the Fragments Be Pieced Together? *J Gerontol A Biol Sci Med Sci* 2006; **61**: 365–6.

384 Rexroth DF, Tennstedt SL, Jones RN, et al. Relationship of Demographic and Health Factors to Cognition in Older Adults in the ACTIVE Study. *J Aging Health* 2013; **25**: 128S–46S.

385 Ribera Hdl, Valencia Uo. Training Intervention in a Controlled Population of Frail Elderly. https://ClinicalTrials.gov/show/NCT02331459; 2013.

386 Richardson J, Law M, Wishart L, Guyatt G. The use of a simulated environment (easy street) to retrain independent living skills in elderly persons: A randomized controlled trial. *J Gerontol A Biol Sci Med Sci* 2000; **55**: M578–M84.

387 Rietkerk W, Gerritsen DL, Kollen BJ, et al. Effects Of Increasing The Involvement Of Community-Dwelling Frail Older Adults In A Proactive Assessment Service: A Pragmatic Trial. *Clin Interv Aging* 2019; **14**: 1985–95.

388 Rikard RV, Berkowsky RW, Cotten SR. Discontinued Information and Communication Technology Usage among Older Adults in Continuing Care Retirement Communities in the United States. *Gerontology* 2018; **64**: 188–200.

389 Ristolainen H, Kannasoja S, Tiilikainen E, Hakala M, Narhi K, Rissanen S. Effects of 'participatory group-based care management' on wellbeing of older people living alone: a randomized controlled trial. *Arch Gerontol Geriatr* 2020; **89**: 104095. doi: https://dx.doi.org/10.1016/j.archger.2020.104095.

390 Rivas-Ruiz F, Machón M, Mateo-Abad M, et al. Tackling frailty at primary care: evaluation of the effectiveness of a multicomponent intervention through a randomised controlled trial: study protocol. *BMJ Open* 2020; **10**: e034591. doi: 10.1136/bmjopen-2019-034591.

391 ISRCTN17143761. *A multicomponent frailty intervention in community-dwelling elderly persons*. BioMed Central: ISRCTN Registry; 2019. URL: http://isrctn.com/ISRCTN17143761 (accessed 11 May 2020).

392 Robichaud L, Hebert R, Roy PM, Roy C. A preventive program for community-dwelling elderly at risk of functional decline: a pilot study. *Arch Gerontol Geriatr* 2000; **30**: 73–84.

393 Rodrigues R, Azeredo Z, Ferreira P. Elderly dwelling assessment: Functional status and services utilization. *Eur Geriatr Med* 2010; **1**: S125.

394 Rollins G. Occupational therapy is cost-effective and improves quality of life for seniors. *Rep Med Guidel Outcomes Res* 2002; **13**: 9–10, 2.

395 Rosie J, Taylor D. Sit-to-stand as home exercise for mobility-limited adults over 80 years of age - GrandStand SystemTM may keep you standing? *Age Ageing* 2007; **36**: 555–62.

396 Rosstad T, Salvesen O, Steinsbekk A, Grimsmo A, Sletvold O, Garasen H. Generic care pathway for elderly patients in need of home care services after discharge from hospital: a cluster randomised controlled trial. *BMC Health Serv Res* 2017; **17**: 275.
397 Rubenstein LV, Calkins DR, Young RT, et al. Improving patient function: a randomized trial of functional disability screening. *Ann Intern Med* 1989; **111**: 836–42.
398 Ruikes FG, Zuidema SU, Akkermans RP, Assendelft WJ, Schers HJ, Koopmans RT. Multicomponent Program to Reduce Functional Decline in Frail Elderly People: A Cluster Controlled Trial. *Journal of the American Board of Family Medicine: JABFM* 2016; **29**: 209–17.
399 Rydwik E, Lammes E, Frandin K, Akner G. Effects of a physical and nutritional intervention program for frail elderly people over age 75. A randomized controlled pilot treatment trial. *Aging Clin Exp Res* 2008; **20**: 159–70.
400 Rydwik E, Frandin K, Akner G. Effects of a physical training and nutritional intervention program in frail elderly people regarding habitual physical activity level and activities of daily living-A randomized controlled pilot study. *Arch Gerontol Geriatr* 2010; **51**: 283–9.
401 Rydwik E, Gustafsson T, Frändin K, Akner G. Effects of physical training on aerobic capacity in frail elderly people (75+ years). Influence of lung capacity, cardiovascular disease and medical drug treatment: a randomized controlled pilot trial. *Aging Clin Exp Res* 2010; **22**: 85–94.
402 Saeterbakken AH, Bårdstu HB, Brudeseth A, Andersen V. Effects of Strength Training on Muscle Properties, Physical Function, and Physical Activity among Frail Older People: a Pilot Study. *J Aging Res* 2018; **2018**. doi: 10.1155/2018/8916274.
403 Sahlen KG, Dahlgren L, Hellner BM, Stenlund H, Lindholm L. Preventive home visits postpone mortality--a controlled trial with time-limited results. *BMC Public Health* 2006; **6**: 220.
404 Saito T, Kai I, Takizawa A. Effects of a program to prevent social isolation on loneliness, depression, and subjective well-being of older adults: a randomized trial among older migrants in Japan. *Arch Gerontol Geriatr* 2012; **55**: 539-47.
405 Salem BE, Ma-Pham J, Chen S, Brecht ML, Antonio AL, Ames M. Impact of a Community-Based Frailty Intervention Among Middle-Aged and Older Prefrail and Frail Homeless Women: A Pilot Randomized Controlled Trial. *Community Ment Health J* 2017; **53**: 688–94.
406 Sandberg M, Jakobsson U, Midlov P, Kristensson J. Cost-utility analysis of case management for frail older people: effects of a randomised controlled trial. *Health Econ Rev* 2015; **5**: 51.
407 Sandberg M, Kristensson J, Midlov P, Jakobsson U. Effects on healthcare utilization of case management for frail older people: a randomized controlled trial (RCT). *Arch Gerontol Geriatr* 2015; **60**: 71–81.
408 Sanders C, Rogers A, Bowen R, et al. Exploring barriers to participation and adoption of telehealth and telecare within the Whole System Demonstrator trial: a qualitative study. *BMC Health Serv Res* 2012; **12**: 220.
409 Sanjuan M, Calero M-D, Abarca S, Navarro E. Evaluation of the effects a caregiver training programme has on the cognitive and functional maintenance of the elderly. *Estudios de Psicologia* 2018; **39**: 437–64.
410 Santanasto AJ, Glynn NW, Lovato LC, et al. Effect of Physical Activity versus Health Education on Physical Function, Grip Strength and Mobility. *J Am Geriatr Soc* 2017; **65**: 1427-33.
411 Sato D, Kaneda K, Wakabayashi H, Nomura T. Comparison two-year [sic] effects of once-weekly and twice-weekly water exercise on health-related quality of life of community-dwelling frail elderly people at a day-service facility. *Disabil Rehabil* 2009; **31**: 84–93.
412 Schraeder C, Shelton P, Sager M. The effects of a collaborative model of primary care on the mortality and hospital use of community-dwelling older adults. *J Gerontol A Biol Sci Med Sci* 2001; **56**: M106–12.
413 Scott AH, Butin DN, Tewfik D, Burkhardt A, Mandel D, Nelson L. Occupational therapy as a means to wellness with the elderly. *Physical and Occupational Therapy in Geriatrics* 2001; **18**: 3–22.
414 Senior HEJ, Parsons M, Kerse N, et al. Promoting independence in frail older people: A randomised controlled trial of a restorative care service in New Zealand. *Age Ageing* 2014; **43**: 418–24.
415 Sherwood DA. The effects of homemaker and day care services on the association of elderly recipients with their family networks. 1980.
416 Sink KM, Espeland MA, Rushing J, et al. The LIFE Cognition Study: design and baseline characteristics. *Clin Interv Aging* 2014; **9**: 1425-36.
417 Sisco SM, Marsiske M, Gross AL, Rebok GW. The Influence of Cognitive Training on Older Adults' Recall for Short Stories. *J Aging Health* 2013; **25**: 230S–48S.
418 Smeeth L, Fletcher AE, Stirling S, et al. Randomised comparison of three methods of administering a screening questionnaire to elderly people: findings from the MRC trial of the assessment and management of older people in the community. *BMJ* 2001; **323**: 1403–7.
419 Smeeth L, Ng ES. Intraclass correlation coefficients for cluster randomized trials in primary care: data from the MRC Trial of the Assessment and Management of Older People in the Community. *Control Clin Trials* 2002; **23**: 409–21.

420 Smith DT, Judge S, Malone A, Moynes RC, Conviser J, Skinner JS. Effects of bioDensity Training and Power Plate Whole-Body Vibration on Strength, Balance, and Functional Independence in Older Adults. *J Aging Phys Act* 2016; **24**: 139–48.
421 Spoelstra SL, Schueller M, Sikorskii A. Testing an implementation strategy bundle on adoption and sustainability of evidence to optimize physical function in community-dwelling disabled and older adults in a Medicaid waiver: a multi-site pragmatic hybrid type III protocol. *Implement Sci* 2019; **14**: 60.
422 Spoelstra SL, Sikorskii A, Gitlin LN, Schueller M, Kline M, Szanton SL. Dissemination of the CAPABLE Model of Care in a Medicaid Waiver Program to Improve Physical Function. *J Am Geriatr Soc* 2019; **67**: 363–70.
423 Spoorenberg SLW, Wynia K, Uittenbroek RJ, Kremer HPH, Reijneveld SA. Effects of a population-based, person-centred and integrated care service on health, wellbeing and self-management of community-living older adults: A randomised controlled trial on Embrace. *PLoS One* 2018; **13**: e0190751.
424 Spoorenberg SL, Reijneveld SA, Uittenbroek RJ, Kremer HP, Wynia K. Health-Related Problems and Changes After 1 Year as Assessed With the Geriatric ICF Core Set (Geriatrics) in Community-Living Older Adults Who Are Frail Receiving Person-Centered and Integrated Care From Embrace. *Arch Phys Med Rehabil* 2019; **100**: 2334–45.
425 Stathi A, Withall J, Greaves CJ, et al. A community-based physical activity intervention to prevent mobility-related disability for retired older people (REtirement in ACTion (REACT)): study protocol for a randomised controlled trial. *Trials* 2018; **19**: 228.
426 Withall J, Greaves CJ, Thompson JL, et al. The Tribulations of Trials: Lessons Learnt Recruiting 777 Older Adults Into REtirement in ACTion (REACT), a Trial of a Community, Group-Based Active Aging Intervention Targeting Mobility Disability. *J Gerontol A Biol Sci Med Sci* 2020; **75**: 2387–95. doi: 10.1093/gerona/glaa051.
427 ISRCTN45627165. *The REACT (retirement in action) study*. BioMed Central: ISRCTN Registry; 2016. URL: https://doi.org/10.1186/ISRCTN45627165

http://www.who.int/trialsearch/Trial2.aspx?TrialID=ISRCTN45627165 (accessed 11 May 2020).
428 Steventon A, Bardsley M, Billings J, et al. Effect of telehealth on use of secondary care and mortality: findings from the Whole System Demonstrator cluster randomised trial. *BMJ* 2012; **344**: e3874.
429 Steventon A, Bardsley M, Billings J, et al. Effect of telecare on use of health and social care services: findings from the Whole Systems Demonstrator cluster randomised trial. *Age Ageing* 2013; **42**: 501–8.
430 Stewart AL, Verboncoeur CJ, McLellan BY, et al. Physical activity outcomes of CHAMPS II: a physical activity promotion program for older adults. *J Gerontol A Biol Sci Med Sci* 2001; **56**: M465–70.
431 Summers MJ, Rainero I, Vercelli AE, et al. The My Active and Healthy Aging (My-AHA) ICT platform to detect and prevent frailty in older adults: Randomized control trial design and protocol. *Alzheimer's and Dementia: Translational Research and Clinical Interventions* 2018; **4**: 252–62.
432 Tarazona-Santabalbina FJ, Gomez-Cabrera MC, Perez-Ros P, et al. A Multicomponent Exercise Intervention that Reverses Frailty and Improves Cognition, Emotion, and Social Networking in the Community-Dwelling Frail Elderly: A Randomized Clinical Trial. *J Am Med Dir Assoc* 2016; **17**: 426–33.
433 Taube E, Kristensson J, Midlov P, Jakobsson U. The use of case management for community-dwelling older people: the effects on loneliness, symptoms of depression and life satisfaction in a randomised controlled trial. *Scand J Caring Sci* 2018; **32**: 889–901.
434 Teh R, Kerse N, Waters DL, et al. Study protocol of a randomised controlled trial to examine the impact of a complex intervention in pre-frail older adults. *Aging Clin Exp Res* 2019; **31**: 1407–17.
435 Tennstedt S, Howland J, Lachman M, Peterson E, Kasten L, Jette A. A randomized, controlled trial of a group intervention to reduce fear of falling and associated activity restriction in older adults. *J Gerontol B Psychol Sci Soc Sci* 1998; **53**: P384–92.
436 Tennstedt SL, Unverzagt FW. The ACTIVE Study: Study Overview and Major Findings. *J Aging Health* 2013; **25**: 3S–20S.
437 Thom JM, Nelis SM, Cooney JK, Hindle JV, Jones IR, Clare L. Promotion of Healthy Aging Within a Community Center Through Behavior Change: Health and Fitness Findings From the AgeWell Pilot Randomized Controlled Trial. *J Aging Phys Act* 2021; **29**: 80–8. doi: 10.1123/japa.2019-0396.
438 Tieland M, Dirks ML, van der Zwaluw N, et al. Protein supplementation increases muscle mass gain during prolonged resistance-type exercise training in frail elderly people: a randomized, double-blind, placebo-controlled trial. *J Am Med Dir Assoc* 2012; **13**: 713–9. doi: 10.1016/j.jamda.2012.05.020.
439 Timonen L, Rantanen T, Makinen E, Timonen TE, Tormakangas T, Sulkava R. Effects of group-based exercise program on functional abilities in frail older women after hospital discharge. *Aging Clin Exp Res* 2006; **18**: 50–6.
440 Toivo T, Airaksinen M, Dimitrow M, et al. Enhanced coordination of care to reduce medication risks in older home care clients in primary care: a randomized controlled trial. *BMC Geriatr* 2019; **19**: 1–11.

441 Toledano-González A, Labajos-Manzanares T, Romero-Ayuso D. Well-Being, Self-Efficacy and Independence in older adults: A Randomized Trial of Occupational Therapy. *Arch Gerontol Geriatr* 2019; **83**: 277–84.
442 Townsend J, Piper M, Frank AO, Dyer S, North WR, Meade TW. Reduction in hospital readmission stay of elderly patients by a community based hospital discharge scheme: a randomised controlled trial. *BMJ* 1988; **297**: 544–7.
443 Trombini-Souza F, de Maio Nascimento M, da Silva TFA, de Araujo RC, Perracini MR, Sacco ICN. Dual-task training with progression from variable- to fixed-priority instructions versus dual-task training with variable-priority on gait speed in community-dwelling older adults: A protocol for a randomized controlled trial : Variable- and fixed-priority dual-task for older adults. *BMC Geriatr* 2020; **20**: 76. doi: 10.1186/s12877-020-1479-2.
444 Turunen KM, Aaltonen-Määttä L, Törmäkangas T, et al. Effects of an individually targeted multicomponent counseling and home-based rehabilitation program on physical activity and mobility in community-dwelling older people after discharge from hospital: a randomized controlled trial. *Clin Rehabil* 2020; **34**: 491–503. doi: 10.1177/0269215519901155.
445 Uittenbroek RJ, Spoorenberg SL, Brans R, et al. [Embrace, a model for integrated elderly care]. *Tijdschr Gerontol Geriatr* 2014; **45**: 92–104.
446 Uittenbroek RJ, van Asselt ADI, Spoorenberg SLW, Kremer HPH, Wynia K, Reijneveld SA. Integrated and Person-Centered Care for Community-Living Older Adults: A Cost-Effectiveness Study. *Health Services Research* 2018; **53**: 3471–94.
447 Ukawa S, Satoh H, Yuasa M, et al. A randomized controlled trial of a Functioning Improvement Tool home-visit program and its effect on cognitive function in older persons. *Int J Geriatr Psychiatry* 2012; **27**: 557–64.
448 Ukawa S, Yuasa M, Ikeno T, et al. Randomised controlled pilot study in Japan comparing a home visit program using a Functioning Improvement Tool with a home visit with conversation alone. *Australas J Ageing* 2012; **31**: 187–9.
449 Ukawa S, Yuasa M, Ikeno T, Ikoma K, Kishi R. The effect of a Functioning Improvement Tool home visit program on instrumental activities of daily living and depressive status in older people. *Int J Geriatr Psychiatry* 2012; **27**: 1206–8.
450 Ukawa S, Tamakoshi A, Ono K. A randomized controlled trial of a long-term functioning improvement tool home visit program for frail older Japanese people. *Int J Geriatr Psychiatry* 2015; **30**: 887–8.
451 van de Sant AJW, de Vries NM, Hoogeboom TJ, Nijhuis-van der Sanden MWG. Implementation of a Personalized, Cost-Effective Physical Therapy Approach (Coach2Move) for Older Adults: Barriers and Facilitators. *J Geriatr Phys Ther* 2019; **42**: E1–E16.
452 van den Helder J, Mehra S, Tieland M, et al. Effects of a Blended Home-Based Exercise Program and Protein Counselling in Community Dwelling Older Adults: Results of the Vitamin Rct. *Clin Nutr* 2019; **38**: S2.
453 van den Helder J, Mehra S, van Dronkelaar C, et al. Blended home-based exercise and dietary protein in community-dwelling older adults: a cluster randomized controlled trial. *Journal of Cachexia, Sarcopenia and Muscle* 2020; **11**: 1590–602. doi: 10.1002/jcsm.12634.
454 van Haaren E. The elderly and volunteer assistance (2). The volunteer assistance system: un unstable balance. [Dutch]. *TVZ : het vakblad voor de verpleging* 1993: 25–8.
455 van Haaren E. The elderly and volunteer assistance (1). The volunteer assistance system: the couple P. [Dutch]. *TVZ : het vakblad voor de verpleging* 1993: 23–4.
456 Vaz Fragoso CA, Beavers DP, Hankinson JL, et al. Respiratory impairment and dyspnea and their associations with physical inactivity and mobility in sedentary community-dwelling older persons. *J Am Geriatr Soc* 2014; **62**: 622–8.
457 Vaz Fragoso CA, Miller ME, King AC, et al. Effect of structured physical activity on sleep-wake behaviors in sedentary elderly adults with mobility limitations. *J Am Geriatr Soc* 2015; **63**: 1381-90.
458 Vaz Fragoso CA, Beavers DP, Anton SD, et al. Effect of Structured Physical Activity on Respiratory Outcomes in Sedentary Elderly Adults with Mobility Limitations. *J Am Geriatr Soc* 2016; **64**: 501–9.
459 Vellas B, Touchon J, Weiner M. MAPT (multidomain Alzheimer preventive trial) imaging (MRI, FDG-PET, amyloid-PET) data. *J Nutr Health Aging* 2012; **16**: 812-5.
460 Venturelli M, Lanza M, Muti E, Schena F. Positive effects of physical training in activity of daily living-dependent older adults. *Exp Aging Res* 2010; **36**: 190–205.
461 Vetter NJ, Lewis PA, Ford D. Can health visitors prevent fractures in elderly people? *BMJ* 1992; **304**: 888–90.
462 Vienna MUo. Healthy Life for Frail Malnourished Seniors Performed Together With Trained Lay Buddies. https://ClinicalTrials.gov/show/NCT01991639; 2014.

463 von Renteln-Kruse W, Dapp U, Anders J, et al. The LUCAS consortium: objectives of interdisciplinary research on selected aspects of ageing and health care for older people in an urban community. *Z Gerontol Geriatr* 2011; **44**: 250–5.

464 Wadley VG, Crowe M, Marsiske M, et al. Changes in everyday function in individuals with psychometrically defined mild cognitive impairment in the advanced cognitive training for independent and vital elderly study. *J Am Geriatr Soc* 2007; **55**: 1192–8.

465 Wagner EH, LaCroix AZ, Grothaus L, et al. Preventing disability and falls in older adults: a population-based randomized trial. *Am J Public Health* 1994; **84**: 1800–6.

466 Walker MF, Hawkins K, Gladman JRF, Lincoln NB. Randomised controlled trial of occupational therapy at home: Results at 1 year [4]. *Journal of Neurology Neurosurgery and Psychiatry* 2001; **70**: 267.

467 Walker DG, Aedo C, Albala C, et al. Methods for economic evaluation of a factorial-design cluster randomised controlled trial of a nutrition supplement and an exercise programme among healthy older people living in Santiago, Chile: the CENEX study. *BMC Health Serv Res* 2009; **9**: 85.

468 Wallace R, Lees C, Minou M, Singleton D, Stratton G. Effects of a 12-week community exercise programme on older people. *Nurs Older People* 2014; **26**: 20–6.

469 Wallen M, Stanley M. Occupational therapy intervention, involving preventive health promotion, maintained an improvement in well being in older independent-living adults 6 months following completion of intervention. *Aust Occup Ther J* 2003; **50**: 109-10.

470 Wan TTH, Weissert WG, Livieratos BB. Geriatric day care and homemaker services: An experimental study. *J Gerontol* 1980; **35**: 256–74.

471 Wang X, Hsu FC, Isom S, et al. Effects of a 12-month physical activity intervention on prevalence of metabolic syndrome in elderly men and women. *J Gerontol A Biol Sci Med Sci* 2012; **67**: 417–24.

472 Wasson E, Rosso AL, Santanasto AJ, et al. Neural correlates of perceived physical and mental fatigability in older adults: A pilot study. *Exp Gerontol* 2019; **115**: 139–47.

473 Watanabe Y, Yamada Y, Yokoyama K, et al. Comprehensive geriatric intervention program with and without weekly class-style exercise: research protocol of a cluster randomized controlled trial in Kyoto-Kameoka Study. *Clin Interv Aging* 2018; **13**: 1019-33.

474 Watanabe Y, Yamada Y, Yoshida T, et al. Comprehensive geriatric intervention in community-dwelling older adults: a cluster-randomized controlled trial. *Journal of Cachexia, Sarcopenia and Muscle* 2020; **11**: 26–37. doi: 10.1002/jcsm.12504.

475 White H, McConnell E, Clipp E, et al. A randomized controlled trial of the psychosocial impact of providing internet training and access to older adults. *Aging and Mental Health* 2002; **6**: 213–21.

476 Whitehead PJ, James M, Belshaw S, Dawson T, Day MR, Walker MF. Bathing adaptations in the homes of older adults (BATH-OUT): protocol for a feasibility randomised controlled trial (RCT). *BMJ Open* 2016; **6**: e013448.

477 Whitehead PJ, Golding-Day MR, Belshaw S, Dawson T, James M, Walker MF. Bathing adaptations in the homes of older adults (BATH-OUT): results of a feasibility randomised controlled trial (RCT). *BMC Public Health* 2018; **18**: 1293.

478 Wilber KH, Allen D, Shannon GR, Alongi S. Partnering Managed Care and Community-Based Services for Frail Elders: The Care Advocate Program. *J Am Geriatr Soc* 2003; **51**: 807–12.

479 Wilhelmson K, Duner A, Eklund K, et al. Design of a randomized controlled study of a multi-professional and multidimensional intervention targeting frail elderly people. *BMC Geriatr* 2011; **11**: 24.

480 Williams H, Blue B, Langlois PF. Do follow-up home visits by military nurses of chronically ill medical patients reduce readmissions? *Mil Med* 1994; **159**: 141–4.

481 Williamson JD, Espeland M, Kritchevsky SB, et al. Changes in cognitive function in a randomized trial of physical activity: results of the lifestyle interventions and independence for elders pilot study. *J Gerontol A Biol Sci Med Sci* 2009; **64**: 688-94.

482 Willis SL, Caskie GIL. Reasoning Training in the ACTIVE Study: How Much Is Needed and Who Benefits? *J Aging Health* 2013; **25**: 43S–64S.

483 Wilson DJ, Mitchell JM, Kemp BJ, Adkins RH, Mann W. Effects of assistive technology on functional decline in people aging with a disability. *Assist Technol* 2009; **21**: 208–17.

484 Wolf SL, Barnhart HX, Kutner NG, McNeely E, Coogler C, Xu T. Reducing frailty and falls in older persons: an investigation of Tai Chi and computerized balance training. Atlanta FICSIT Group. Frailty and Injuries: cooperative Studies of Intervention Techniques. *J Am Geriatr Soc* 1996; **44**: 489-97.

485 Wolinsky FD, Vander Weg MW, Martin R, et al. The effect of speed-of-processing training on depressive symptoms in ACTIVE. *J Gerontol A Biol Sci Med Sci* 2009; **64**: 468-72.

486 Wong FK, Ho MM, Yeung S, Tam SK, Chow SK. Effects of a health-social partnership transitional program on hospital readmission: a randomized controlled trial. *Soc Sci Med* 2011; **73**: 960–9.

487 Wong AKC, Wong FKY, Chang KKP. A proactive mobile health application program for promoting self-care health management among older adults in the community: Study protocol of a three-arm randomized controlled trial. *Gerontology* 2020; **66**: 506–13. doi: 10.1159/000509129.
488 Wong AKC, Wong FKY, Ngai JSC, Hung SYK, Li WC. Effectiveness of a health-social partnership program for discharged non-frail older adults: a pilot study. *BMC Geriatr* 2020; **20**: 339. doi: 10.1186/s12877-020-01722-5.
489 Wooldridge DB, McInnis JB, Nelson R, Piller J, Scott S, Whiting P. A geriatric evaluation and management (GEM) program: Evaluation of patient outcomes. *Aging Clin Exp Res* 1995; **7**: 251–4.
490 Xie H, Bai H, Wang Y, et al. Effects of Oral Nutritional Supplement on Nutritional Status and Muscle Function: A Randomized, Controlled Trial in Community-Dwell Elderly. *Clin Nutr* 2019; **38**: S73.
491 Yao S, Zheng P, Ji L, et al. The effect of comprehensive assessment and multi-disciplinary management for the geriatric and frail patient: A multi-center, randomized, parallel controlled trial. *Medicine* 2020; **99**: e22873. doi: 10.1097/MD.0000000000022873.
492 Yeo G, Ingram L, Skurnick J, Crapo L. Effects of a geriatric clinic on functional health and well-being of elders. *J Gerontol* 1987; **42**: 252–8.
493 Yim VWT, Rainer TH, Graham CA, et al. Emergency department intervention for high-risk elders: Identification strategy and randomised controlled trial to reduce hospitalisation and institutionalisation. *Hong Kong Medical Journal* 2011; **17**: S4–S7.
494 Yoon JS, Roque NA, Andringa R, et al. Intervention Comparative Effectiveness for Adult Cognitive Training (ICE-ACT) Trial: Rationale, design, and baseline characteristics. *Contemp Clin Trials* 2019; **78**: 76–87.
495 Young JB, Robinson M, Chell S, et al. A whole system study of intermediate care services for older people. *Age Ageing* 2005; **34**: 577–83.
496 Yu R, Tong C, Ho F, Woo J. Effects of a Multicomponent Frailty Prevention Program in Prefrail Community-Dwelling Older Persons: A Randomized Controlled Trial. *J Am Med Dir Assoc* 2020; **21**: 294.e1–.e10. doi: 10.1016/j.jamda.2019.08.024.
497 Zaragoza Ud. Physical Fitness, Body Composition and Frailty in Elderly People.Exercise Program Effecsts. EXERNET Elder 3.0. https://ClinicalTrials.gov/show/NCT03831841; 2018.
498 Zauszniewski JA, Eggenschwiler K, Preechawong S, Roberts BL, Morris DL. Effects of teaching resourcefulness skills to elders. *Aging and Mental Health* 2006; **10**: 404–12.
499 Zhu LY, Chan R, Kwok T, Cheng KCC, Ha A, Woo J. Effects of exercise and nutrition supplementation in community-dwelling older Chinese people with sarcopenia: A randomized controlled trial. *Age Ageing* 2019; **48**: 220–8.
500 Zijlstra GAR, van Haastregt JCM, van Eijk JT, de Witte LP, Ambergen T, Kempen GIJM. Mediating effects of psychosocial factors on concerns about falling and daily activity in a multicomponent cognitive behavioral group intervention. *Aging Ment Health* 2011; **15**: 68–77.
501 Zillich AJ, Snyder ME, Frail CK, et al. A randomized, controlled pragmatic trial of telephonic medication therapy management to reduce hospitalization in home health patients. *Health Services Research* 2014; **49**: 1537–54.
502 Zimmer JG, Groth-Juncker A, McCusker J. A randomized controlled study of a home health care team. *Am J Public Health* 1985; **75**: 134–41.
503 Alegria M, Frontera W, Cruz-Gonzalez M, et al. Effectiveness of a Disability Preventive Intervention for Minority and Immigrant Elders: The Positive Minds-Strong Bodies Randomized Clinical Trial. *Am J Geriatr Psychiatry* 2019; **27**: 1299–313. doi: 10.1016/j.jagp.2019.08.008.
504 Arthanat S. Promoting information communication technology adoption and acceptance for aging-in-place: A randomized controlled trial. *J Appl Gerontol* 2019; **40**: 471–80. doi: 10.1177/0733464819891045.
505 Auvinen K, Voutilainen A, Jyrkkä J, Lönnroos E, Mäntyselkä P. Interprofessional medication assessment among home care patients: any impact on functioning? Results from a randomised controlled trial. *BMC Geriatr* 2020; **20**: 390. doi: 10.1186/s12877-020-01796-1.
506 Balaban DJ, Goldfarb NI, Perkel RL, Carlson BL. Follow-up study of an urban family medicine home visit program. *J Fam Pract* 1988; **26**: 307–12.
507 Barenfeld E, Dahlin-Ivanoff S, Wallin L, Gustafsson S. Promoting aging migrants' capabilities: A randomized controlled trial concerning activities of daily living and self-rated health. *AIMS Public Health* 2018; **5**: 173–88. doi: 10.3934/publichealth.2018.2.173.
508 Bernabei R, Landi F, Gambassi G, et al. Randomised trial of impact of model of integrated care and case management for older people living in the community. *BMJ* 1998; **316**: 1348–51. doi: 10.1136/bmj.316.7141.1348.
509 Bleijenberg N, Drubbel I, Schuurmans MJ, et al. Effectiveness of a proactive primary care program on preserving daily functioning of older people: a cluster randomized controlled trial. *J Am Geriatr Soc* 2016; **64**: 1779–88. doi: 10.1111/jgs.14325.

510 Blom J, den Elzen W, van Houwelingen AH, et al. Effectiveness and cost-effectiveness of a proactive, goal-oriented, integrated care model in general practice for older people. A cluster randomised controlled trial: Integrated Systematic Care for older People--the ISCOPE study. *Age Ageing* 2016; **45**: 30–41. doi: 10.1093/ageing/afv174.

511 Borrows A, Holland R. Independent living centre occupational therapy (OT) versus routine community OT. *Int J Ther Rehabil* 2013; **20**: 187–94. doi: 10.12968/ijtr.2013.20.4.187.

512 Botjes E. Methodebeschrijving EigenKrachtWijzer: Databank Effectieve sociale interventies Movisie, 2013.

513 Bouman A, van Rossum E, Ambergen T, Kempen G, Knipschild P. Effects of a home visiting program for older people with poor health status: a randomized, clinical trial in The Netherlands. *J Am Geriatr Soc* 2008; **56**: 397–404. doi: 10.1111/j.1532-5415.2007.01565.x.

514 Brettschneider C, Luck T, Fleischer S, et al. Cost-utility analysis of a preventive home visit program for older adults in Germany. *BMC Health Serv Res* 2015; **15**: 141. doi: 10.1186/s12913-015-0817-0.

515 Cameron ID, Fairhall N, Langron C, et al. A multifactorial interdisciplinary intervention reduces frailty in older people: randomized trial. *BMC Med* 2013; **11**: 65. doi: 10.1186/1741-7015-11-65.

516 Carpenter GI, Demopoulos GR. Screening the elderly in the community: controlled trial of dependency surveillance using a questionnaire administered by volunteers. *BMJ* 1990; **300**: 1253–6. doi: 10.1136/bmj.300.6734.1253.

517 Cesari M, Demougeot L, Boccalon H, Guyonnet S, Vellas B, Andrieu S. The Multidomain Intervention to preveNt disability in ElDers (MINDED) project: rationale and study design of a pilot study. *Contemp Clin Trials* 2014; **38**: 145–54. doi: 10.1016/j.cct.2014.04.006.

518 Challis D, Clarkson P, Williamson J, et al. The value of specialist clinical assessment of older people prior to entry to care homes. *Age Ageing* 2004; **33**: 25–34. doi: 10.1093/ageing/afh007.

519 Clark F, Azen SP, Zemke R, et al. Occupational therapy for independent-living older adults. A randomized controlled trial. *JAMA* 1997; **278**: 1321–6. doi: 10.1001/jama.1997.03550160041036.

520 Clark F, Jackson J, Carlson M, et al. Effectiveness of a lifestyle intervention in promoting the well-being of independently living older people: results of the Well Elderly 2 Randomised Controlled Trial. [Erratum in: J Epidemiol Community Health 2012;66:1079-82]. *J Epidemiol Community Health* 2012; **66**: 782–90. doi: 10.1136/jech.2009.099754.

521 Coleman EA, Grothaus LC, Sandhu N, Wagner EH. Chronic Care Clinics: A randomized controlled trial of a model of primary care for frail older adults. *J Am Geriatr Soc* 1999; **47**: 775–83. doi: 10.1111/j.1532-5415.1999.tb03832.x.

522 Counsell SR, Callahan CM, Clark DO, et al. Geriatric care management for low-income seniors: a randomized controlled trial. *JAMA* 2007; **298**: 2623–33. doi: 10.1001/jama.298.22.2623.

523 Cutchin MP, Coppola S, Talley V, Svihula J, Catellier D, Shank KH. Feasibility and effects of preventive home visits for at-risk older people: design of a randomized controlled trial. *BMC Geriatr* 2009; **9**: 54. doi: 10.1186/1471-2318-9-54.

524 Dalby DM, Sellors JW, Fraser FD, Fraser C, van Ineveld C, Howard M. Effect of preventive home visits by a nurse on the outcomes of frail elderly people in the community: a randomized controlled trial. *CMAJ* 2000; **162**: 497–500.

525 de Craen AJ, Gussekloo J, Blauw GJ, Willems CG, Westendorp RG. Randomised controlled trial of unsolicited occupational therapy in community-dwelling elderly people: the LOTIS trial. *PLoS Clin Trials* 2006; **1**: e2. doi: 10.1371/journal.pctr.0010002.

526 Dorresteijn TA, Zijlstra GA, Ambergen AW, Delbaere K, Vlaeyen JW, Kempen GI. Effectiveness of a home-based cognitive behavioral program to manage concerns about falls in community-dwelling, frail older people: results of a randomized controlled trial. *BMC Geriatr* 2016; **16**: 2. doi: 10.1186/s12877-015-0177-y.

527 Dupuy L, Froger C, Consel C, Sauzeon H. Everyday functioning benefits from an assisted living platform amongst frail older adults and their caregivers. *Front Aging Neurosci* 2017; **9**: 302. doi: 10.3389/fnagi.2017.00302.

528 Fabacher D, Josephson K, Pietruszka F, Linderborn K, Morley JE, Rubenstein LZ. An in-home preventive assessment program for independent older adults: a randomized controlled trial. *J Am Geriatr Soc* 1994; **42**: 630–8. doi: 10.1111/j.1532-5415.1994.tb06862.x.

529 Fairhall N, Kurrle SE, Sherrington C, et al. Effectiveness of a multifactorial intervention on preventing development of frailty in pre-frail older people: study protocol for a randomised controlled trial. *BMJ Open* 2015; **5**: e007091. doi: 10.1136/bmjopen-2014-007091.

530 Faul AC, Yankeelov PA, Rowan NL, et al. Impact on geriatric assessment and self-management support on community-dwelling older adults with chronic illnesses. *J Gerontol Soc Work* 2009; **52**: 230–49. doi: 10.1080/01634370802609288.

531 Fernandez-Barres S, Garcia-Barco M, Basora J, et al. The efficacy of a nutrition education intervention to prevent risk of malnutrition for dependent elderly patients receiving Home Care: A randomized controlled trial. *Int J Nurs Stud* 2017; **70**: 131–41. doi: 10.1016/j.ijnurstu.2017.02.020.
532 Fischer G, Sandholzer H, Perschke-Hartmann C. Final report of the scientific support of “Getting Healthy Elderly (GÄW)”. A prevention project of the AOK Lower Saxony.)[German] (Abschlussbericht der wissenschaftlichen Begleitung von "Gesund Älter Werden (GÄW)“): AOK Niedersachsen, 2009.
533 Ford AB, Katz S, Downs TD, Adams M. Results of long-term home nursing: the influence of disability. *J Chronic Dis* 1971; **24**: 591–6. doi: 10.1016/0021-9681(71)90047-6.
534 Fox PJ, Breuer W, Wright JA. Effects of a health promotion program on sustaining health behaviors in older adults. *Am J Prev Med* 1997; **13**: 257–64.
535 Fristedt S, Nystedt P, Skogar O. Mobile geriatric teams - a cost-effective way of improving patient safety and reducing traditional healthcare utilization among the frail elderly? A randomized controlled trial. *Clin Interv Aging* 2019; **14**: 1911–24. doi: 10.2147/CIA.S208388.
536 Gene Huguet L, Navarro Gonzalez M, Kostov B, et al. Pre Frail 80: Multifactorial intervention to prevent progression of pre-frailty to frailty in the elderly. *J Nutr Health Aging* 2018; **22**: 1266–74. doi: 10.1007/s12603-018-1089-2.
537 Gill TM, Baker DI, Gottschalk M, Peduzzi PN, Allore H, Byers A. A program to prevent functional decline in physically frail, elderly persons who live at home. *N Engl J Med* 2002; **347**: 1068–74. doi: 10.1056/NEJMoa020423.
538 Giné-Garriga M, Sansano-Nadal O, Tully MA, et al. Accelerometer-measured sedentary and physical activity time and their correlates in European older adults: The SITLESS study. *J Gerontol A Biol Sci Med Sci* 2020; **75**: 1754–62. doi: 10.1093/gerona/glaa016.
539 Gitlin LN, Winter L, Dennis MP, Corcoran M, Schinfeld S, Hauck WW. A randomized trial of a multicomponent home intervention to reduce functional difficulties in older adults. *J Am Geriatr Soc* 2006; **54**: 809–16. doi: 10.1111/j.1532-5415.2006.00703.x.
540 Grimmer K, Luker J, Beaton K, Kumar S, Crockett A, Price K. TRialing individualized interventions to prevent functional decline in at-risk older adults (TRIIFL): study protocol for a randomized controlled trial nested in a longitudinal observational study. *Trials* 2013; **14**: 266. doi: 10.1186/1745-6215-14-266.
541 Gustafson DH, Kornfield R, Mares M-L, et al. Effect of an eHealth intervention on older adults' quality of life and health-related outcomes: a randomized clinical trial. *J Gen Intern Med* 2021; **37**: 521–30. doi: 10.1007/s11606-021-06888-1.
542 Gustafsson S, Eklund K, Wilhelmson K, et al. Long-Term Outcome for ADL Following the Health-Promoting RCT—Elderly Persons in the Risk Zone. *Gerontologist* 2013; **53**: 654–63. doi: 10.1093/geront/gns121.
543 Hall N, De Beck P, Johnson D, Mackinnon K, Gutman G, Glick N. Randomized trial of a health promotion program for frail elders. *Can J Aging* 1992; **11**: 72–91.
544 Harari D, Iliffe S, Kharicha K, et al. Promotion of health in older people: a randomised controlled trial of health risk appraisal in British general practice. *Age Ageing* 2008; **37**: 565–71. doi: 10.1093/ageing/afn150.
545 Hattori S, Yoshida T, Okumura Y, Kondo K. Effects of reablement on the independence of community-dwelling older adults with mild disability: A randomized controlled trial. *Int J Environ Res Public Health* 2019; **16**: 3954. doi: 10.3390/ijerph16203954.
546 Hay WI, van Ineveld C, Browne G, et al. Prospective care of elderly patients in family practice. Is screening effective? *Can Fam Physician* 1998; **44**: 2677–87.
547 Hebert R, Robichaud L, Roy PM, Bravo G, Voyer L. Efficacy of a nurse-led multidimensional preventive programme for older people at risk of functional decline. A randomized controlled trial. *Age Ageing* 2001; **30**: 147–53. doi: 10.1093/ageing/30.2.147.
548 Henderson MJ. In-home preventive health assessment and telephone case management for over 75s living alone in independent living units: A cluster randomized controlled trial [PhD thesis]. Queensland: Queensland University of Technology; 2005.
549 Hendriksen C, Lund E, Stromgard E. Consequences of assessment and intervention among elderly people: a three year randomised controlled trial. *Br Med J (Clin Res Ed)* 1984; **289**: 1522–4. doi: 10.1136/bmj.289.6457.1522.
550 Hogg W, Lemelin J, Dahrouge S, et al. Randomized controlled trial of anticipatory and preventive multidisciplinary team care: for complex patients in a community-based primary care setting. *Can Fam Physician* 2009; **55**: e76–85.
551 Holland SK, Greenberg J, Tidwell L, Malone J, Mullan J, Newcomer R. Community-based health coaching, exercise, and health service utilization. *J Aging Health* 2005; **17**: 697–716. doi: 10.1177/0898264305277959.
552 Howel D, Moffatt S, Haighton C, et al. Does domiciliary welfare rights advice improve health-related quality of life in independent-living, socio-economically disadvantaged people aged ≥60 years?

Randomised controlled trial, economic and process evaluations in the North East of England. *PLoS One* 2019; **14**: e0209560. doi: 10.1371/journal.pone.0209560.

553 Imhof L, Naef R, Wallhagen MI, Schwarz J, Mahrer-Imhof R. Effects of an advanced practice nurse in-home health consultation program for community-dwelling persons aged 80 and older. *J Am Geriatr Soc* 2012; **60**: 2223–31. doi: 10.1111/jgs.12026.

554 Jing L, Jin Y, Zhang X, Wang F, Song Y, Xing F. The effect of Baduanjin qigong combined with CBT on physical fitness and psychological health of elderly housebound. *Medicine* 2018; **97**: e13654. doi: 10.1097/MD.0000000000013654.

555 Jitapunkul S. A randomised controlled trial of regular surveillance in Thai elderly using a simple questionnaire administered by non-professional personnel. *J Med Assoc Thai* 1998; **81**: 352–6.

556 Kerse N, McLean C, Moyes SA, et al. The cluster-randomized BRIGHT trial: Proactive case finding for community-dwelling older adults. *Ann Fam Med* 2014; **12**: 514–24. doi: 10.1370/afm.1696.

557 King AII, Parsons M, Robinson E, Jorgensen D. Assessing the impact of a restorative home care service in New Zealand: A cluster randomised controlled trial. *Health Soc Care Community* 2012; **20**: 365–74. doi: 10.1111/j.1365-2524.2011.01039.x.

558 Kono A, Izumi K, Yoshiyuki N, Kanaya Y, Rubenstein LZ. Effects of an updated preventive home visit program based on a systematic structured assessment of care needs for ambulatory frail older adults in Japan: A randomized controlled trial. *J Gerontol A Biol Sci Med Sci* 2016; **71**: 1631–7. doi: 10.1093/gerona/glw068.

559 Kono A, Kai I, Sakato C, Harker JO, Rubenstein LZ. Effect of preventive home visits for ambulatory housebound elders in Japan: a pilot study. *Aging Clin Exp Res* 2004; **16**: 293–9. doi: 10.1007/BF03324554.

560 Kono A, Kanaya Y, Fujita T, et al. Effects of a preventive home visit program in ambulatory frail older people: a randomized controlled trial. *J Gerontol A Biol Sci Med Sci* 2012; **67**: 302–9. doi: 10.1093/gerona/glr176.

561 Kukkonen-Harjula K, Karmeniemi P, Suikkanen S, et al. Long-term home-based physiotherapy for older people with signs of frailty-RCT (NCT02305433) [P-229]. *Eur Geriatr Med* 2017; **8**: S105. doi: 10.1016/S1878-7649(17)30179-1.

562 Lambotte D, De Donder L, De Roeck EE, et al. Randomized controlled trial to evaluate a prevention program for frail community-dwelling older adults: a D-SCOPE protocol. *BMC Geriatr* 2018; **18**: 194. doi: 10.1186/s12877-018-0875-3.

563 Leung AC-t, Liu C-p, Chow NW-s, Chi I. Cost-Benefit Analysis of a Case Management Project for the Community-Dwelling Frail Elderly in Hong Kong. *J Appl Gerontol* 2004; **23**: 70–85. doi: 10.1177/0733464804263088.

564 Leveille SG, Wagner EH, Davis C, et al. Preventing disability and managing chronic illness in frail older adults: a randomized trial of a community-based partnership with primary care. *J Am Geriatr Soc* 1998; **46**: 1191–8. doi: 10.1111/j.1532-5415.1998.tb04533.x.

565 Lewin G, De San Miguel K, Knuiman M, et al. A randomised controlled trial of the Home Independence Program, an Australian restorative home-care programme for older adults. *Health Soc Care Community* 2013; **21**: 69–78. doi: 10.1111/j.1365-2524.2012.01088.x.

566 Liddle J, March L, Carfrae B, et al. Can occupational therapy intervention play a part in maintaining independence and quality of life in older people? A randomised controlled trial. *Aust N Z J Public Health* 1996; **20**: 574–8. doi: 10.1111/j.1467-842x.1996.tb01068.x.

567 Liimatta H, Lampela P, Laitinen-Parkkonen P, Pitkala KH. Effects of preventive home visits on health-related quality-of-life and mortality in home-dwelling older adults. *Scand J Prim Health Care* 2019; **37**: 90–7. doi: 10.1080/02813432.2019.1569372.

568 Loh DA, Hairi NN, Choo WY, et al. MultiComponent Exercise and theRApeutic lifeStyle (CERgAS) intervention to improve physical performance and maintain independent living among urban poor older people--a cluster randomised controlled trial. *BMC Geriatr* 2015; **15**: 8. doi: 10.1186/s12877-015-0002-7.

569 Lood Q, Gustafsson S, Dahlin Ivanoff S. Bridging barriers to health promotion: a feasibility pilot study of the 'Promoting Aging Migrants' Capabilities study'. *J Eval Clin Pract* 2015; **21**: 604–13. doi: 10.1111/jep.12345.

570 Mann J, Thompson F, McDermott R, Esterman A, Strivens E. Impact of an integrated community-based model of care for older people with complex conditions on hospital emergency presentations and admissions: a step-wedged cluster randomized trial. *BMC Health Serv Res* 2021; **21**: 701. doi: 10.1186/s12913-021-06668-x.

571 Mann WC, Ottenbacher KJ, Fraas L, Tomita M, Granger CV. Effectiveness of assistive technology and environmental interventions in maintaining independence and reducing home care costs for the frail elderly. A randomized controlled trial. *Arch Fam Med* 1999; **8**: 210–7. doi: 10.1001/archfami.8.3.210.

572 Markle-Reid M, Weir R, Browne G, Roberts J, Gafni A, Henderson S. Health promotion for frail older home care clients. *J Adv Nurs* 2006; **54**: 381–95. doi: 10.1111/j.1365-2648.2006.03817.x.
573 Melis RJ, van Eijken MI, Teerenstra S, et al. A randomized study of a multidisciplinary program to intervene on geriatric syndromes in vulnerable older people who live at home (Dutch EASYcare Study). *J Gerontol A Biol Sci Med Sci* 2008; **63**: 283–90. doi: 10.1093/gerona/63.3.283.
574 Meng H, Friedman B, Wamsley BR, Mukamel D, Eggert GM. Effect of a consumer-directed voucher and a disease-management-health-promotion nurse intervention on home care use. *Gerontologist* 2005; **45**: 167–76. doi: 10.1093/geront/45.2.167.
575 Messens L, Quinn S, Saez I, Cuidad Mas MJ, Squillace P, Laura A-G. Health monitoring and sOcial integration environMent for Supporting WidE ExTension of independent life at HOME (Home Sweet Home): Final Trial Evaluation Report. Antwerp: Zorgbedrijf Antwerpen, 2014.
576 Metzelthin SF, Van Rossum E, De Witte LP, et al. Effectiveness of interdisciplinary primary care approach to reduce disability in community dwelling frail older people: Cluster randomised controlled trial. *BMJ* 2013; **347**: f5264. doi: 10.1136/bmj.f5264.
577 Moll van Charante EP, Richard E, Eurelings LS, et al. Effectiveness of a 6-year multidomain vascular care intervention to prevent dementia (preDIVA): a cluster-randomised controlled trial. *Lancet* 2016; **388**: 797–805. doi: 10.1016/S0140-6736(16)30950-3.
578 Monteserin Nadal R, Altimir Losada S, Brotons Cuixart C, et al. Randomized clinical trial on the efficacy of global geriatric assessment in primary care. [Spanish]. *Rev Esp Geriatr Gerontol* 2008; **43**: 5–12. doi: 10.1016/s0211-139x(08)71144-2.
579 Morey MC, Ekelund C, Pearson M, et al. Project LIFE: a partnership to increase physical activity in elders with multiple chronic illnesses. *J Aging Phys Act* 2006; **14**: 324–43. doi: 10.1123/japa.14.3.324.
580 Morey MC, Peterson MJ, Pieper CF, et al. The Veterans Learning to Improve Fitness and Function in Elders Study: a randomized trial of primary care–based physical activity counseling for older men. *J Am Geriatr Soc* 2009; **57**: 1166–74. doi: 10.1111/j.1532-5415.2009.02301.x.
581 Morgan GS, Haase AM, Campbell RM, Ben-Shlomo Y. A pilot randomised controlled trial of physical activity facilitation for older adults: feasibility study findings. *Pilot Feasibility Stud* 2019; **5**: 40. doi: 10.1186/s40814-019-0414-9.
582 Newbury JW, Marley JE, Beilby JJ. A randomised controlled trial of the outcome of health assessment of people aged 75 years and over. *Med J Aust* 2001; **175**: 104–7. doi: 10.5694/j.1326-5377.2001.tb143541.x.
583 Newcomer R, Maravilla V, Faculjak P, Graves MT. Outcomes of preventive case management among high-risk elderly in three medical groups: a randomized clinical trial. *Eval Health Prof* 2004; **27**: 323–48. doi: 10.1177/0163278704270011.
584 Ng TP, Feng L, Nyunt MS, et al. Nutritional, physical, cognitive, and combination interventions and frailty reversal among older adults: A randomized controlled trial. *Am J Med* 2015; **128**: 1225–36.e1. doi: 10.1016/j.amjmed.2015.06.017.
585 Parsons J, Rouse P, Robinson EM, Sheridan N, Connolly MJ. Goal setting as a feature of homecare services for older people: does it make a difference? *Age Ageing* 2012; **41**: 24–9. doi: 10.1093/ageing/afr118.
586 Parsons M, Senior H, Kerse N, Chen MH, Jacobs S, Anderson C. Randomised trial of restorative home care for frail older people in New Zealand. *Nurs Older People* 2017; **29**: 27–33. doi: 10.7748/nop.2017.e897.
587 Parsons M, Senior H, Kerse N, et al. Should care managers for older adults be located in primary care? A randomized controlled trial. *J Am Geriatr Soc* 2012; **60**: 86–92. doi: 10.1111/j.1532-5415.2011.03763.x.
588 Pathy MS, Bayer A, Harding K, Dibble A. Randomised trial of case finding and surveillance of elderly people at home. *Lancet* 1992; **340**: 890–3. doi: 10.1016/0140-6736(92)93294-W.
589 Phelan EA, Balderson B, Levine M, et al. Delivering effective primary care to older adults: a randomized, controlled trial of the senior resource team at group health cooperative. *J Am Geriatr Soc* 2007; **55**: 1748–56. doi: 10.1111/j.1532-5415.2007.01416.x.
590 Ploeg J, Brazil K, Hutchison B, et al. Effect of preventive primary care outreach on health related quality of life among older adults at risk of functional decline: randomised controlled trial. *BMJ* 2010; **340**: c1480. doi: 10.1136/bmj.c1480.
591 Profener F, Anders J, Dapp U, Minder CE, Golgert S, von Renteln-Kruse W. Acceptance of preventive home visits among frail elderly persons : Participants an non-participants in a Follow-up after 2 and 4 years within the LUCAS longitudinal study. [German]. *Z Gerontol Geriatr* 2016; **49**: 596–605. doi: 10.1007/s00391-016-1127-9.
592 Rockwood K, Stadnyk K, Carver D, et al. A clinimetric evaluation of specialized geriatric care for rural dwelling, frail older people. *J Am Geriatr Soc* 2000; **48**: 1080–5. doi: 10.1111/j.1532-5415.2000.tb04783.x.

593 Romera-Liebana L, Orfila F, Segura JM, et al. Effects of a primary care-based multifactorial intervention on physical and cognitive function in frail, elderly individuals: A randomized controlled trial. *J Gerontol A Biol Sci Med Sci* 2018; **73**: 1688-74. doi: 10.1093/gerona/glx259.
594 Rooijackers TH, Kempen GIJM, Zijlstra GAR, et al. Effectiveness of a reablement training program for homecare staff on older adults' sedentary behavior: A cluster randomized controlled trial. *J Am Geriatr Soc* 2021; **69**: 2566–78. doi: 10.1111/jgs.17286.
595 Rubenstein LZ, Alessi CA, Josephson KR, Trinidad Hoyl M, Harker JO, Pietruszka FM. A randomized trial of a screening, case finding, and referral system for older veterans in primary care. *J Am Geriatr Soc* 2007; **55**: 166–74. doi: 10.1111/j.1532-5415.2007.01044.x.
596 Ryvicker M, Feldman PH, Rosati RJ, Sobolewski S, Maduro GA, Jr., Schwartz T. Improving functional outcomes in home care patients: impact and challenges of disseminating a quality improvement initiative. *J Healthc Qual* 2011; **33**: 28–36. doi: 10.1111/j.1945-1474.2011.00156.x.
597 Serra-Prat M, Sist X, Domenich R, et al. Effectiveness of an intervention to prevent frailty in pre-frail community-dwelling older people consulting in primary care: a randomised controlled trial. *Age Ageing* 2017; **46**: 401-7. doi: 10.1093/ageing/afw242.
598 Shapiro A, Taylor M. Effects of a community-based early intervention program on the subjective well-being, institutionalization, and mortality of low-income elders. *Gerontologist* 2002; **42**: 334–41. doi: 10.1093/geront/42.3.334.
599 Sherman H, Soderhielm-Blid S, Forsberg C, Karp A, Tornkvist L. Effects of preventive home visits by district nurses on self-reported health of 75-year-olds. *Prim Health Care Res Dev* 2016; **17**: 56–71. doi: 10.1017/S1463423614000565.
600 Siemonsma PC, Blom JW, Hofstetter H, et al. The effectiveness of functional task exercise and physical therapy as prevention of functional decline in community dwelling older people with complex health problems. *BMC Geriatr* 2018; **18**: 164. doi: 10.1186/s12877-018-0859-3.
601 Stewart S, Harvey I, Poland F, Lloyd-Smith W, Mugford M, Flood C. Are occupational therapists more effective than social workers when assessing frail older people? Results of CAMELOT, a randomised controlled trial. *Age Ageing* 2005; **34**: 41–6. doi: 10.1093/ageing/afh230.
602 Stuck AE, Aronow HU, Steiner A, et al. A trial of annual in-home comprehensive geriatric assessments for elderly people living in the community. *N Engl J Med* 1995; **333**: 1184–9. doi: 10.1056/NEJM199511023331805.
603 Stuck AE, Minder CE, Peter-Wuest I, et al. A randomized trial of in-home visits for disability prevention in community-dwelling older people at low and high risk for nursing home admission. *Arch Intern Med* 2000; **160**: 977–86. doi: 10.1001/archinte.160.7.977.
604 Stuck AE, Moser A, Morf U, et al. Effect of health risk assessment and counselling on health behaviour and survival in older people: a pragmatic randomised trial. *PLoS Med* 2015; **12**: e1001889. doi: 10.1371/journal.pmed.1001889.
605 Suijker JJ, van Rijn M, Buurman BM, Ter Riet G, Moll van Charante EP, de Rooij SE. Effects of nurse-led multifactorial care to prevent disability in community-living older people: Cluster randomized trial. *PLoS One* 2016; **11**: e0158714. doi: 10.1371/journal.pone.0158714.
606 Szanton SL, Thorpe RJ, Boyd C, et al. Community aging in place, advancing better living for elders: a bio-behavioral-environmental intervention to improve function and health-related quality of life in disabled older adults. *J Am Geriatr Soc* 2011; **59**: 2314–20. doi: 10.1111/j.1532-5415.2011.03698.x.
607 Szanton SL, Xue QL, Leff B, et al. Effect of a biobehavioral environmental approach on disability among low-income older adults: A randomized clinical trial. *JAMA Intern Med* 2019; **179**: 204–11. doi: 10.1001/jamainternmed.2018.6026.
608 Takahashi PY, Pecina JL, Upatising B, et al. A randomized controlled trial of telemonitoring in older adults with multiple health issues to prevent hospitalizations and emergency department visits. *Arch Intern Med* 2012; **172**: 773–9. doi: 10.1001/archinternmed.2012.256.
609 Teut M, Schnabel K, Baur R, et al. Effects and feasibility of an Integrative Medicine program for geriatric patients-a cluster-randomized pilot study. *Clin Interv Aging* 2013; **8**: 953-61. doi: 10.2147/CIA.S45242.
610 Thiel C, Braun T, Grüneberg C. Physical training as core component of multimodal treatment of older frail people-study protocol of a randomized controlled pilot study. *Z Gerontol Geriatr* 2019; **52**: 45-60. doi: 10.1007/s00391-018-1443-3.
611 Thomas R, Worrall G, Elgar F, Knight J. Can they keep going on their own? A four-year randomized trial of functional assessments of community residents. *Can J Aging* 2007; **26**: 379–90. doi: 10.3138/cja.26.4.379.
612 Tomita MR, Mann WC, Stanton K, Tomita AD, Sundar V. Use of currently available smart home technology by frail elders: process and outcomes. *Top Geriatr Rehabil* 2007; **23**: 24–34. doi: 10.1097/00013614-200701000-00005.

613 Tulloch AJ, Moore V. A randomized controlled trial of geriatric screening and surveillance in general practice. *J R Coll Gen Pract* 1979; **29**: 733–40.

614 Tuntland H, Aaslund MK, Espehaug B, Førland O, Kjeken I. Reablement in community-dwelling older adults: a randomised controlled trial. *BMC Geriatr* 2015; **15**: 1–11. doi: 10.1186/s12877-015-0142-9.

615 van der Pols-Vijlbrief R, Wijnhoven HAH, Bosmans JE, Twisk JWR, Visser M. Targeting the underlying causes of undernutrition. Cost-effectiveness of a multifactorial personalized intervention in community-dwelling older adults: A randomized controlled trial. *Clin Nutr* 2017; **36**: 1498–508. doi: 10.1016/j.clnu.2016.09.030.

616 van Dongen EJ, Haveman-Nies A, Doets EL, Dorhout BG, de Groot LC. Effectiveness of a diet and resistance exercise intervention on muscle health in older adults: ProMuscle in Practice. *J Am Med Dir Assoc* 2020; **21**: 1065–72. doi: 10.1016/j.jamda.2019.11.026.

617 van Heuvelen MJ, Hochstenbach JB, Brouwer WH, et al. Differences between participants and non-participants in an RCT on physical activity and psychological interventions for older persons. *Aging Clin Exp Res* 2005; **17**: 236-45. doi: 10.1007/BF03324603.

618 van Hout HP, Jansen AP, van Marwijk HW, Pronk M, Frijters DF, Nijpels G. Prevention of adverse health trajectories in a vulnerable elderly population through nurse home visits: a randomized controlled trial [ISRCTN05358495]. *J Gerontol A Biol Sci Med Sci* 2010; **65**: 734–42. doi: 10.1093/gerona/glq037.

619 van Leeuwen KM, Bosmans JE, Jansen AP, et al. Cost-effectiveness of a chronic care model for frail older adults in primary care: Economic evaluation alongside a stepped-wedge cluster-randomized trial. *J Am Geriatr Soc* 2015; **63**: 2494–504. doi: 10.1111/jgs.13834.

620 van Lieshout MRJ, Bleijenberg N, Schuurmans MJ, de Wit NJ. The effectiveness of a PRoactive multicomponent intervention program on disability in independently living older people: A randomized controlled trial. *J Nutr Health Aging* 2018; **22**: 1051–9. doi: 10.1007/s12603-018-1101-x.

621 van Rossum E, Frederiks CM, Philipsen H, Portengen K, Wiskerke J, Knipschild P. Effects of preventive home visits to elderly people. *BMJ* 1993; **307**: 27–32. doi: 10.1136/bmj.307.6895.27.

622 Vass M, Avlund K, Lauridsen J, Hendriksen C. Feasible model for prevention of functional decline in older people: municipality-randomized, controlled trial. *J Am Geriatr Soc* 2005; **53**: 563–8. doi: 10.1111/j.1532-5415.2005.53201.x.

623 Vetter NJ, Jones DA, Victor CR. Effect of health visitors working with elderly patients in general practice: a randomised controlled trial. *Br Med J (Clin Res Ed)* 1984; **288**: 369–72. doi: 10.1136/bmj.288.6414.369.

624 von Bonsdorff MB, Leinonen R, Kujala UM, et al. Effect of physical activity counseling on disability in older people: A 2-year randomized controlled trial. *J Am Geriatr Soc* 2008; **56**: 2188–94. doi: 10.1111/j.1532-5415.2008.02000.x.

625 Wallace JI, Buchner DM, Grothaus L, et al. Implementation and effectiveness of a community-based health promotion program for older adults. *J Gerontol A Biol Sci Med Sci* 1998; **53**: M301–6. doi: 10.1093/gerona/53a.4.m301.

626 Walters K, Frost R, Kharicha K, et al. Home-based health promotion for older people with mild frailty: the HomeHealth intervention development and feasibility RCT. *Health Technol Assess* 2017; **21**: 1–128. doi: 10.3310/hta21730.

627 Whitehead PJ, Walker MF, Parry RH, Latif Z, McGeorge ID, Drummond AE. Occupational Therapy in HomEcare Re-ablement Services (OTHERS): results of a feasibility randomised controlled trial. *BMJ Open* 2016; **6**: e011868. doi: 10.1136/bmjopen-2016-011868.

628 Williams EI, Greenwell J, Groom LM. The care of people over 75 years old after discharge from hospital: an evaluation of timetabled visiting by Health Visitor Assistants. *J Public Health Med* 1992; **14**: 138–44. doi: 10.1093/oxfordjournals.pubmed.a042711.

629 Wolter A, Stolle C, Roth G, Rothgang H. Does the resident care assessment instrument improve long-term home care? - results of a nation-wide study in Germany. [German]. *Gesundheitswesen* 2013; **75**: 29–32. doi: 10.1055/s-0032-1309013.

630 Wong AKC, Wong FKY, Chang K. Effectiveness of a community-based self-care promoting program for community-dwelling older adults: A randomized controlled trial. *Age Ageing* 2019; **48**: 852–8. doi: 10.1093/ageing/afz095.

631 Yamada Y, Ikegami N. Preventive home visits for community- dwelling frail elderly people based on minimum data set-home care: randomized controlled trial. *Geriatr Gerontol Int* 2003; **3**: 236–42. doi: 10.1111/j.1444-1586.2003.00103.x.

632 NCT02317432. *Building Community Capacity for Disability Prevention for Minority Elders (Positive Minds - Strong Bodies)*. US National Library of Medicine: ClinicalTrials.gov; 2014. URL: https://clinicaltrials.gov/ct2/show/NCT02317432 (accessed 19 May 2020).

633 Porteny T, Alegría M, del Cueto P, et al. Barriers and strategies for implementing community-based interventions with minority elders: positive minds-strong bodies. *Implement Sci Commun* 2020; **1**: 1–13.

634 Arthanat S, Vroman KG, Lysack C. A home-based individualized information communication technology training program for older adults: a demonstration of effectiveness and value. *Disabil Rehabil Assist Technol* 2016; **11**: 316–24.
635 Arthanat S, Vroman KG, Lysack C, Grizzetti J. Multi-stakeholder perspectives on information communication technology training for older adults: implications for teaching and learning. *Disabil Rehabil Assist Technol* 2019; **14**: 453–61.
636 Auvinen K, Raisanen J, Merikoski M, et al. The Finnish Interprofessional Medication Assessment (FIMA): baseline findings from home care setting. *Aging Clin Exp Res* 2019; **31**: 1471–9.
637 Merikoski M, Auvinen K, Kumpusalo-Vauhkonen A, et al. Iäkkäiden Lääkehoidon Moniammatillinen Arviointi (ILMA). Helsinki: Sosiaali-ja Terveysministeriö, 2017.
638 NCT02398812. *The Interprofessional Medication Assessment for Older Patients*. US National Library of Medicine: ClinicalTrials.gov; 2015. URL: https://clinicaltrials.gov/ct2/show/NCT02398812 (accessed 28 April 2020).
639 Gustafsson S, Berglund H, Faronbi J, Barenfeld E, Ottenvall Hammar I. Minor positive effects of health-promoting senior meetings for older community-dwelling persons on loneliness, social network, and social support. *Clin Interv Aging* 2017; **12**: 1867–77.
640 Arola A, Dahlin-Ivanoff S, Haggblom-Kronlof G. Impact of a person-centred group intervention on life satisfaction and engagement in activities among persons aging in the context of migration. *Scand J Occup Ther* 2019: 1–11.
641 Gustafsson S, Lood Q, Wilhelmson K, Haggblom-Kronlof G, Landahl S, Dahlin-Ivanoff S. A person-centred approach to health promotion for persons 70+ who have migrated to Sweden: promoting aging migrants' capabilities implementation and RCT study protocol. *BMC Geriatr* 2015; **15**: 10.
642 NCT01841853. *RCT of Health-promoting Intervention for Older Foreign-born Adults*. US National Library of Medicine: ClinicalTrials.gov; 2013. URL: https://clinicaltrials.gov/ct2/show/NCT01841853 (accessed 28 April 2020).
643 Coleston-Shields DM. Integration of medical and social services for elderly people in the community reduced costs and use of health services. *Evidence Based Mental Health* 1998; **1**: 106. doi: 10.1136/ebmh.1.4.106.
644 Britian O. Integration of services for elderly people reduced costs and use of health services [commentary on Bernabei R, Landi F, Gambassi G et al. Randomized trial of impact of model of integrated care and case management for older people living in the community. BR MED J 1998;316(7141):1348-51]. *Evidence Based Nursing* 1999: 20–.
645 Bleijenberg N, Drubbel I, Ten Dam VH, Numans ME, Schuurmans MJ, de Wit NJ. Proactive and integrated primary care for frail older people: design and methodological challenges of the Utrecht primary care PROactive frailty intervention trial (U-PROFIT). *BMC Geriatr* 2012; **12**: 16.
646 Bleijenberg N, ten Dam VH, Drubbel I, Numans ME, de Wit NJ, Schuurmans MJ. Development of a proactive care program (U-CARE) to preserve physical functioning of frail older people in primary care. *J Nurs Scholarsh* 2013; **45**: 230–7.
647 Bleijenberg N, ten Dam VH, Drubbel I, Numans ME, Wit NJ, Schuurmans MJ. Treatment fidelity of an evidence-based nurse-led intervention in a proactive primary care program for older people. *Worldviews Evid Based Nurs* 2016; **13**: 75–84.
648 Bleijenberg N, Ten Dam VH, Steunenberg B, et al. Exploring the expectations, needs and experiences of general practitioners and nurses towards a proactive and structured care programme for frail older patients: a mixed-methods study. *J Adv Nurs* 2013; **69**: 2262–73.
649 Laan W, Zuithoff NP, Drubbel I, et al. Validity and reliability of the Katz-15 scale to measure unfavorable health outcomes in community-dwelling older people. *J Nutr Health Aging* 2014; **18**: 848–54.
650 NTR2288. *Central Utrecht Elderly Care Project 'Om U': "Somebody who knows what's going on and can see things from my side."*. World Health Organization: International Clinical Trials Registry Platform; 2010. URL: https://trialsearch.who.int/Trial2.aspx?TrialID=NTR2288 (accessed 11 May 2020).
651 ten Dam VH, Bleijenberg N, Numans ME, Drubbel I, Schuurmans MJ, de Wit NJ. [Proactive and structured care for the elderly in primary care]. [Dutch]. *Tijdschr Gerontol Geriatr* 2013; **44**: 81–9.
652 Metzelthin SF, Bleijenberg N, Blom JW, Imhof L. Primary care strategies to maintain independence of frail older people: looking for evidence across borders. *Eur Geriatr Med* 2014; **5**: S31-S2.
653 Bleijenberg N, Imhof L, Mahrer-Imhof R, Wallhagen MI, de Wit NJ, Schuurmans MJ. Patient Characteristics Associated With a Successful Response to Nurse-Led Care Programs Targeting the Oldest-Old: A Comparison of Two RCTs. *Worldviews Evid Based Nurs* 2017; **14**: 210–22.
654 NL1836. *Integrated Systematic Care for Older PEople (ISCOPE)*. Netherlands National Trial Register; 2009. URL: https://www.trialregister.nl/trial/1836 (accessed 28 April 2020).

655 van Blijswijk SCE, Blom JW, de Craen AJM, den Elzen WPJ, Gussekloo J. Prediction of functional decline in community-dwelling older persons in general practice: a cohort study. *BMC Geriatr* 2018; **18**: 1–9.
656 ISRCTN66679751. *Implementation of an internet-based instrument, the 'Eigen Kracht Wijzer' in older people*. BioMed Central: ISRCTN Registry; 2013. URL: https://doi.org/10.1186/ISRCTN66679751 (accessed 11 May 2020).
657 Botjes E. EigenKrachtWijzer. 20 September 2013 2013. https://www.movisie.nl/interventie/eigenkrachtwijzer (accessed 17 May 2020).
658 Nicolaides-Bouman A, van Rossum E, Kempen GI, Knipschild P. Effects of home visits by home nurses to elderly people with health problems: design of a randomised clinical trial in the Netherlands [ISRCTN92017183]. *BMC Health Serv Res* 2004; **4**: 35.
659 Nicolaides-Bouman A, van Rossum E, Habets H, Kempen GI, Knipschild P. Home visiting programme for older people with health problems: process evaluation. *J Adv Nurs* 2007; **58**: 425–35.
660 ISRCTN92017183. *Effects of home visits by home nurses to elderly people with health problems*. BioMed Central: ISRCTN Registry; 2004. URL: http://www.isrctn.com/ISRCTN92017183 (accessed 28 April 2020).
661 Bouman AIE, Van Rossum E, Ambergen TW, Kempen GIJM, Knipschild PG. House calls to elderly with health problems: Effects of a random experiment. [Dutch]. *Ned Tijdschr Geneeskd* 2009; **153**: 644–50.
662 Bouman A, van Rossum E, Evers S, Ambergen T, Kempen G, Knipschild P. Effects on health care use and associated cost of a home visiting program for older people with poor health status: a randomized clinical trial in the Netherlands. *J Gerontol A Biol Sci Med Sci* 2008; **63**: 291–7.
663 Brettschneider C, Luck T, Fleischer S, et al. Cost-utility analysis of preventive home visits in older adults. *Value Health* 2014; **17**: A511.
664 Fleischer S, Roling G, Beutner K, et al. Growing old at home - A randomized controlled trial to investigate the effectiveness and cost-effectiveness of preventive home visits to reduce nursing home admissions: Study protocol [NCT00644826]. *BMC Public Health* 2008; **8**: 185. doi: 10.1186/1471-2458-8-185.
665 Luck T, Roling G, Heinrich S, Luppa M, Matschinger H, Fleischer S. Altern zu Hause-Unterstützung durch präventive Hausbesuche-Eine randomisierte kontrollierte Interventionsstudie. *Hallesche Beitr Gesundh Pflegewissenschaften* 2011; **10**.
666 NCT00644826. *Growing Old at Home*. US National Library of Medicine: ClinicalTrials.gov; 2008. URL: https://clinicaltrials.gov/ct2/show/NCT00644826 (accessed 28 April 2020).
667 ACTRN12608000250336. *Frailty Intervention Trial*. Australian New Zealand Clinical Trials Registry; 2008. URL: https://www.anzctr.org.au/Trial/Registration/TrialReview.aspx?ACTRN=12608000250336 (accessed 8 May 2020).
668 ACTRN12608000507381. *Increasing participation. A sub-study of the Frailty Intervention Trial (FIT)*. Australian New Zealand Clinical Trials Registry; 2008. URL: http://www.anzctr.org.au/ACTRN12608000507381.aspx (accessed 11 May 2020).
669 ACTRN12608000565347. *The impact of a community based multidisciplinary frailty intervention for older people on informal carers' experience of caregiving.* Australian New Zealand Clinical Trials Registry; 2008. URL: https://www.anzctr.org.au/Trial/Registration/TrialReview.aspx?id=83207 (accessed 12 May 2020).
670 Aggar C, Ronaldson S, Cameron ID. Reactions to caregiving during an intervention targeting frailty in community living older people. *BMC Geriatr* 2012; **12**: 66.
671 Fairhall N, Aggar C, Kurrle SE, et al. Frailty Intervention Trial (FIT). *BMC Geriatr* 2008; **8**: 27.
672 Fairhall N, Sherrington C, Cameron ID, et al. A multifactorial intervention for frail older people is more than twice as effective among those who are compliant: complier average causal effect analysis of a randomised trial. *J Physiother* 2017; **63**: 40–4.
673 Fairhall N, Sherrington C, Kurrle SE, Lord SR, Lockwood K, Cameron ID. Effect of a multifactorial interdisciplinary intervention on mobility-related disability in frail older people: randomised controlled trial. *BMC Med* 2012; **10**: 120.
674 Fairhall N, Sherrington C, Kurrle SE, et al. Economic evaluation of a multifactorial, interdisciplinary intervention versus usual care to reduce frailty in frail older people. *J Am Med Dir Assoc* 2015; **16**: 41-8.
675 Fairhall N, Sherrington C, Lord S, Susan K, Cameron ID. A multifactorial interdisciplinary intervention reduces frailty, increases function and is cost-effective in older adults who are frail: randomised controlled trial. *Physiotherapy* 2015; **101**: eS371-eS2. doi: 10.1016/j.physio.2015.03.588.
676 Fairhall N, Sherrington C, Lord SR, et al. Effect of a multifactorial, interdisciplinary intervention on risk factors for falls and fall rate in frail older people: a randomised controlled trial. *Age Ageing* 2013; **43**: 616–22.
677 Cesari M, Demougeot L, Vellas B. Multidomain intervention to prevent disability in elders-The MINDED study. *Eur Geriatr Med* 2013; **1**: S84–S5.

678 Cesari MM. Multidomain Intervention to preveNt Disability in ElDers – MINDED. n.d. https://anr.fr/Project-ANR-11-CHEX-0008 (accessed 6 November 2020).
679 Fougère B, Aubertin-Leheudre M, Vellas B, et al. Clinical research for older adults in rural areas: the MINDED study experience. *Age (Dordr)* 2016; **38**: 30.
680 Fougère B, Vellas B, Andrieu S, Demougeot L, Cluzan C, Cesari M. Difficulties encountered and solutions provided in the implementation of a multidisciplinary intervention for the prevention of dependency in the older population in rural areas: the MINDED study. *Geriatr Psychol Neuropsychiatr Vieil* 2015; **13**: 259–64.
681 NCT02082171. *Multidomain Intervention to Prevent Disability in Elders*. US National Library of Medicine: ClinicalTrials.gov; 2013. URL: https://ClinicalTrials.gov/show/NCT02082171 (accessed 11 May 2020).
682 Zengarini E, Ruggiero C, Mecocci P, Vellas B, Cesari M. Fatigue as a clinical sign of biological aging: exploratory analyses from the MINDED project. *Geriatr Gerontol Int* 2016; **16**: 533.
683 Clarkson P, Venables D, Hughes J, Burns A, Challis D. Integrated specialist assessment of older people and predictors of care-home admission. *Psychol Med* 2006; **36**: 1011–21.
684 Azen SP, Palmer JM, Carlson M, et al. Psychometric properties of a Chinese translation of the SF-36 health survey questionnaire in the Well Elderly Study. *J Aging Health* 1999; **11**: 240–51.
685 Clark F, Azen SP, Carlson M, et al. Embedding health-promoting changes into the daily lives of independent-living older adults: long-term follow-up of occupational therapy intervention. *J Gerontol B Psychol Sci Soc Sci* 2001; **56**: P60–3.
686 Clark FA, Carlson M, Jackson J, Mandel D. Lifestyle redesign improves health and is cost-effective. *OT Practice* 2003; **8**: 9–13.
687 Hay J, LaBree L, Luo R, et al. Cost-effectiveness of preventive occupational therapy for independent-living older adults. *J Am Geriatr Soc* 2002; **50**: 1381–8.
688 Jackson J, Carlson M, Mandel D, Zemke R, Clark F. Occupation in lifestyle redesign: the Well Elderly Study Occupational Therapy Program. *Am J Occup Ther* 1998; **52**: 326–36.
689 Carlson M, Jackson J, Mandel D, et al. Predictors of retention among African American and Hispanic older adult research participants in the Well Elderly 2 randomized controlled trial. *J Appl Gerontol* 2014; **33**: 357–82.
690 Jackson J, Mandel D, Blanchard J, et al. Confronting challenges in intervention research with ethnically diverse older adults: The USC Well Elderly II Trial. *Clin Trials* 2009; **6**: 90–101.
691 NCT00786344. *USC Well Elderly Study 2*. US National Library of Medicine: ClinicalTrials.gov; 2008. URL: https://clinicaltrials.gov/ct2/show/NCT00786344 (accessed 21 August 2020).
692 Schelly D, Ohl A, Nadres R. Dosage and Efficacy in Behavioral Interventions With Community Dwelling Older Adults: Lifestyle Redesign Revisited. *J Appl Gerontol* 2021; **40**: 1087–95. doi: 10.1177/0733464820911335.
693 Seidle JS. The Impact of Response to Stressful Events on Participation in Meaningful Activity: A Secondary Data Analysis Using The Well Elderly 2 Study. *Arch Phys Med Rehabil* 2020; **101 (12)**: e126. doi: 10.1016/j.apmr.2020.10.004.
694 Sonnenfeld M, Karmarkar A. Cortisol as a biomarker in rehabilitation services for community dwelling older adults. *Arch Phys Med Rehabil* 2019; **100**: e13.
695 Bielaszka-DuVernay C. Innovation profile: The 'GRACE' model: In-home assessments lead to better care for dual eligibles. *Health Affairs* 2011; **30**: 431–4.
696 Counsell SR, Callahan CM, Buttar AB, Clark DO, Frank KI. Geriatric Resources for Assessment and Care of Elders (GRACE): a new model of primary care for low-income seniors. *J Am Geriatr Soc* 2006; **54**: 1136–41.
697 Counsell SR, Callahan CM, Tu W, Stump TE, Arling GW. Cost analysis of the Geriatric Resources for Assessment and Care of Elders care management intervention. *J Am Geriatr Soc* 2009; **57**: 1420–6.
698 Iloabuchi TC, Mi D, Tu W, Counsell SR. Risk Factors for Early Hospital Readmission in Low-Income Elderly Adults. *J Am Geriatr Soc* 2014; **62**: 489–94. doi: 10.1111/jgs.12688.
699 NCT00182962. *GRACE: Geriatric Resources for Assessment and Care of Elders*. US National Library of Medicine: ClinicalTrials.gov; 2005. URL: https://clinicaltrials.gov/ct2/show/NCT00182962 (accessed 21 August 2020).
700 NCT00985283. *Feasibility and Effects of Preventive Home Visits for Older Adults*. US National Library of Medicine: ClinicalTrials.gov; 2009. URL: https://clinicaltrials.gov/ct2/show/NCT00985283 (accessed 4 August 2020).
701 Dalby DM, Sellors JW, Fraser FD, et al. Screening seniors for risk of functional decline: results of a survey in family practice. *Can J Public Health* 1999; **90**: 133–7. doi: 10.1007/BF03404117.
702 Claus E, Willen A, de Craen A, Knops H, Willems C. Protocol development for individual occupational therapy in the oldest old [article in Dutch]. *Ned Tijdschr Ergotherapie* 2003; **31**: 83–6.

703 De Craen AJM, Westendorp RGJ, Willems CG, Buskens ICM, Gussekloo J. Assistive devices and community-based services among 85-year-old community-dwelling elderly in The Netherlands: Ownership, use,and need for intervention. *Disabil Rehabil Assist Technol* 2006; **1**: 199–203. doi: 10.1080/17483100612331392835.
704 NCT00278096. *Randomised Controlled Trial of Unsolicited Occupational Therapy in Community-Dwelling Elderly*. US National Library of Medicine: ClinicalTrials.gov; 2006. URL: https://clinicaltrials.gov/show/NCT00278096 (accessed 11 May 2020).
705 NCT01358032. *Evaluating an in-home multicomponent program to manage concerns about falling in frail community dwelling older people*. https://ClinicalTrials.gov/show/; 2009. URL: https://clinicaltrials.gov/ct2/show/NCT01358032 (accessed 11 May 2020).
706 Dorresteijn TA, Zijlstra G, Van Haastregt JC, Vlaeyen JW, Kempen GI. Feasibility of a nurse-led in-home cognitive behavioral program to manage concerns about falls in frail older people: A process evaluation. *Res Nurs Health* 2013; **36**: 257–70.
707 Evers S, Dorresteijn TAC, Wijnen BFM, van Haastregt JCM, Kempen G, Zijlstra GAR. Economic evaluation of a home-based programme to reduce concerns about falls in frail, independently-living older people. *Expert Rev Pharmacoecon Outcomes Res* 2020; **20**: 641–51. doi: 10.1080/14737167.2019.1666714.
708 Dorresteijn TA, Zijlstra GA, Delbaere K, van Rossum E, Vlaeyen JW, Kempen GI. Evaluating an in-home multicomponent cognitive behavioural programme to manage concerns about falls and associated activity avoidance in frail community-dwelling older people: Design of a randomised control trial [NCT01358032]. *BMC Health Serv Res* 2011; **11**: 228.
709 Dupuy L, Consel C, Sauzéon H. Self determination-based design to achieve acceptance of assisted living technologies for older adults. *Comput Human Behav* 2016; **65**: 508–21. doi: 10.1016/j.chb.2016.07.042.
710 ACTRN12613000043730. *Pre-FIT: a multifactorial interdisciplinary treatment program for older people who are pre-frail*. Australian New Zealand Clinical Trials Registry; 2013. URL: https://www.anzctr.org.au/Trial/Registration/TrialReview.aspx?ACTRN=12613000043730 (accessed 16 November 2020).
711 Cameron I, Fairhall N, John B, et al. A multifactorial interdisciplinary intervention in pre-frail older people: randomised trial. *Journal of Innovation in Aging* 2017; **1**: 196.
712 Rowan NL, Gillette PD, Faul AC, et al. Innovative Interdisciplinary Training in and Delivery of Evidence-Based Geriatric Services: Creating a Bridge With Social Work and Physical Therapy. *Gerontol Geriatr Educ* 2009; **30**: 187–204. doi: 10.1080/02701960903133448.
713 Arija V, Martín N, Canela T, et al. Nutrition education intervention for dependent patients: protocol of a randomized controlled trial. *BMC Public Health* 2012; **12**: 373. doi: 10.1186/1471-2458-12-373.
714 NCT01360775. *Nutritional Education for Dependant Patients (atdom_nut)*. US National Library of Medicine: ClinicalTrials.gov; 2011. URL: https://clinicaltrials.gov/ct2/show/NCT01360775 (accessed 28 April 2020).
715 AOKN. Preventive home visits to seniors The prevention program of the AOK Lower Saxony: Getting healthy older Qualitative experience report [German] (Präventive Hausbesuche bei Senioren Das Präventionsprogramm der AOK Niedersachsen: Gesund Älter Werden Qualitativer Erfahrungsbericht). Hannover: AOK – Institut für Gesundheitsconsulting, 2010.
716 Katz S, Ford AB, Downs TD, Adams M, Rusby DI. The effects of continued care: A study of chronic illness in the home. Rockville, MA: United States Department of Health, Education, and Welfare; 1972.
717 NCT03662945. *Mobile Geriatric Teams: Patient Safety and Healthcare Utilization (MGT)*. US National Library of Medicine: ClinicalTrials.gov; 2018. URL: https://clinicaltrials.gov/ct2/show/NCT03662945 (accessed 6 November 2020).
718 Gill TM, Baker DI, Gottschalk M, et al. A prehabilitation program for physically frail community-living older persons. *Arch Phys Med Rehabil* 2003; **84**: 394–404.
719 Gill TM, Baker DI, Gottschalk M, Peduzzi PN, Allore H, Van Ness PH. A prehabilitation program for the prevention of functional decline: effect on higher-level physical function. *Arch Phys Med Rehabil* 2004; **85**: 1043–9.
720 Gill TM, McGloin JM, Gahbauer EA, Shepard DM, Bianco LM. Two Recruitment Strategies for a Clinical Trial of Physically Frail Community-Living Older Persons. *J Am Geriatr Soc* 2001; **49**: 1039–45. doi: 10.1046/j.1532-5415.2001.49206.x.
721 Peduzzi P, Guo Z, Marottoli RA, Gill TM, Araujo K, Allore HG. Improved self-confidence was a mechanism of action in two geriatric trials evaluating physical interventions. *J Clin Epidemiol* 2007; **60**: 94–102.
722 Blackburn NE, Skjodt M, Tully MA, et al. Older Adults' Experiences of a Physical Activity and Sedentary Behaviour Intervention: A Nested Qualitative Study in the SITLESS Multi-Country Randomised Clinical Trial. *Int J Environ Res Public Health* 2021; **18**: 4730. doi: 10.3390/ijerph18094730.

723 Coll-Planas L, Blancafort Alias S, Tully M, et al. Exercise referral schemes enhanced by self-management strategies to reduce sedentary behaviour and increase physical activity among community-dwelling older adults from four European countries: protocol for the process evaluation of the SITLESS randomised controlled trial. *BMJ Open* 2019; **9**: e027073. doi: 10.1136/bmjopen-2018-027073.
724 SITLESS Consortium. SITLESS. 2021. https://sitless.eu/ (accessed 7 May 2021).
725 Deidda M, Coll-Planas L, Gine-Garriga M, et al. Cost-effectiveness of exercise referral schemes enhanced by self-management strategies to battle sedentary behaviour in older adults: protocol for an economic evaluation alongside the SITLESS three-armed pragmatic randomised controlled trial. *BMJ Open* 2018; **8**: e022266.
726 Giné-Garriga M, Coll-Planas L, Guerra M, et al. The SITLESS project: exercise referral schemes enhanced by self-management strategies to battle sedentary behaviour in older adults: study protocol for a randomised controlled trial. *Trials* 2017; **18**: 221. doi: 10.1186/s13063-017-1956-x.
727 NCT02629666. *Exercise Referral Schemes Enhanced by Self-Management Strategies to Battle Sedentary Behaviour (SitLESS)*. US National Library of Medicine: ClinicalTrials.gov; 2015. URL: https://clinicaltrials.gov/ct2/show/NCT02629666 (accessed 14 May 2020).
728 Tully MA, McMullan I, Blackburn NE, et al. Sedentary behavior, physical activity, and mental health in older adults: An isotemporal substitution model. *Scand J Med Sci Sports* 2020; **30**: 1957–65. doi: 10.1111/sms.13762.
729 Tully MA, McMullan I, Blackburn NE, et al. Is Sedentary Behavior or Physical Activity Associated With Loneliness in Older Adults? Results of the European-Wide SITLESS Study. *J Aging Phys Act* 2020; **28**: 549–55. doi: 10.1123/japa.2019-0311.
730 Wilson JJ, Blackburn NE, O'Reilly R, Kee F, Caserotti P, Tully MA. Association of objective sedentary behaviour and self-rated health in English older adults. *BMC Res Notes* 2019; **12**: 12. doi: 10.1186/s13104-019-4050-5.
731 Wilson JJ, McMullan I, Blackburn NE, et al. Associations of sedentary behavior bouts with community-dwelling older adults' physical function. *Scand J Med Sci Sports* 2021; **31**: 153–62. doi: 10.1111/sms.13827.
732 Wilson JJ, Skjødt M, McMullan I, et al. Consequences of Choosing Different Settings When Processing Hip-Based Accelerometry Data From Older Adults: A Practical Approach Using Baseline Data From the SITLESS Study. *J Meas Phys Behav* 2020; **3**: 89–99. doi: 10.1123/jmpb.2019-0037 10.1123/jmpb.2019-0037.
733 Gitlin LN. Enhancing Quality of Life in Functionally Vulnerable Older Adults: From Randomized Trial to Standard Care: Individuals at any age can learn new strategies to engage in valued activities. *Generations (San Francisco, Calif.)* 2010; **34**: 84–7.
734 Gitlin LN, Hauck WW, Dennis MP, Schulz R. Depressive Symptoms in Older African-American and White Adults with Functional Difficulties: The Role of Control Strategies. *J Am Geriatr Soc* 2007; **55**: 1023–30. doi: 10.1111/j.1532-5415.2007.01224.x.
735 Gitlin LN, Hauck WW, Dennis MP, Winter L, Hodgson N, Schinfeld S. Long-term effect on mortality of a home intervention that reduces functional difficulties in older adults: results from a randomized trial. *J Am Geriatr Soc* 2009; **57**: 476–81.
736 Gitlin LN, Hauck WW, Winter L, Dennis MP, Schulz R. Effect of an in-home occupational and physical therapy intervention on reducing mortality in functionally vulnerable older people: preliminary findings. *J Am Geriatr Soc* 2006; **54**: 950–5.
737 Gitlin LN, Parisi J, Huang J, Winter L, Roth DL. Attachment to Life: Psychometric Analyses of the Valuation of Life Scale and Differences Among Older Adults. *Gerontologist* 2016; **56**: e21–31.
738 Gitlin LN, Winter L, Dennis MP, Hauck WW. Variation in response to a home intervention to support daily function by age, race, sex, and education. *J Gerontol A Biol Sci Med Sci* 2008; **63**: 745–50.
739 Gitlin LN, Winter L, Stanley IH. Compensatory Strategies: Prevalence of Use and Relationship to Physical Function and Well-Being. *J Appl Gerontol* 2017; **36**: 647–66. doi: 10.1177/0733464815581479.
740 Jutkowitz E, Gitlin L, Pizzi LT, Lee EH, Dennis M. Cost-effectiveness of able a functional program to decrease mortality in community-dwelling older adults. *Value Health* 2011; **14**: A108.
741 Jutkowitz E, Gitlin LN, Pizzi LT, Lee E, Dennis MP. Cost effectiveness of a home-based intervention that helps functionally vulnerable older adults age in place at home. *J Aging Res* 2012; **2012**: 680265.
742 NCT00249925. *Project ABLE: Advancing Better Living for Elders*. US National Library of Medicine: ClinicalTrials.gov; 2005. URL: https://clinicaltrials.gov/ct2/show/NCT00249925 (accessed 28 April 2020).
743 Rose KC, Gitlin LN, Dennis MP. Readiness to use compensatory strategies among older adults with functional difficulties. *Int Psychogeriatr* 2010; **22**: 1225–39.

744 ACTRN12613000234718. *TRialing Individualised Interventions to prevent FunctionaL decline in at-risk older adults (TRIIFL): A nested randomized controlled trial*. Australian New Zealand Clinical Trials Registry; 2013. URL: http://www.anzctr.org.au/ACTRN12613000234718.aspx (accessed 11 May 2020).
745 Gustafson DH, Sr., McTavish F, Gustafson DH, Jr., et al. The effect of an information and communication technology (ICT) on older adults' quality of life: study protocol for a randomized control trial. *Trials* 2015; **16**: 191.
746 NCT02128789. *Bring Communities and Technology Together for Healthy Aging (ElderTree)*. US National Library of Medicine: ClinicalTrials.gov; 2014. URL: https://clinicaltrials.gov/ct2/show/NCT02128789 (accessed 8 June 2020).
747 Behm L, Eklund K, Wilhelmson K, et al. Health Promotion Can Postpone Frailty: Results from the RCT Elderly Persons in the Risk Zone. *Public Health Nurs* 2016; **33**: 303–15.
748 Behm L, Wilhelmson K, Falk K, Eklund K, Ziden L, Dahlin-Ivanoff S. Positive health outcomes following health-promoting and disease-preventive interventions for independent very old persons: long-term results of the three-armed RCT Elderly Persons in the Risk Zone. *Arch Gerontol Geriatr* 2014; **58**: 376–83.
749 Dahlin-Ivanoff S, Eklund K, Wilhelmson K, et al. For whom is a health-promoting intervention effective? Predictive factors for performing activities of daily living independently. *BMC Geriatr* 2016; **17**: 171–.
750 Dahlin-Ivanoff S, Gosman-Hedstrom G, Edberg AK, et al. Elderly persons in the risk zone. Design of a multidimensional, health-promoting, randomised three-armed controlled trial for "prefrail" people of 80+ years living at home. *BMC Geriatr* 2010; **10**: 27.
751 Gustafsson S, Wilhelmson K, Eklund K, et al. Health-promoting interventions for persons aged 80 and older are successful in the short term-results from the randomized and three-armed elderly persons in the risk zone study. *J Am Geriatr Soc* 2012; **60**: 447–54.
752 NCT00877058. *Support for frail elderly persons - from prevention to palliation*. US National Library of Medicine: ClinicalTrials.gov; 2008. URL: https://ClinicalTrials.gov/show/NCT00877058 (accessed 11 May 2020).
753 Wilhelmson K, Eklund K. Positive Effects on Life Satisfaction Following Health-Promoting Interventions for Frail Older Adults: A Randomized Controlled Study. *Health Psychol Res* 2013; **1**: e12.
754 Ziden L, Haggblom-Kronlof G, Gustafsson S, Lundin-Olsson L, Dahlin-Ivanoff S. Physical function and fear of falling 2 years after the health-promoting randomized controlled trial: elderly persons in the risk zone. *Gerontologist* 2014; **54**: 387–97.
755 Biddulph JP, Iliffe S, Kharicha K, et al. Risk factors for depressed mood amongst a community dwelling older age population in England: cross-sectional survey data from the PRO-AGE study. *BMC Geriatr* 2014; **14**: 5. doi: 10.1186/1471-2318-14-5.
756 Carmaciu C, Iliffe S, Kharicha K, et al. Health risk appraisal in older people 3: prevalence, impact, and context of pain and their implications for GPs. *Br J Gen Pract* 2007; **57**: 630-5.
757 Harari D, Iliffe S, Kharicha K, Stuck AE, Swift CG. Multi-domain health promotion for older people: randomised controlled study of health risk appraisal in primary...British Geriatrics Society: Abstracts of papers presented at the Spring Scientific Meeting, 6-7 April 2006. *Age Ageing* 2006; **35**: i67.
758 Iliffe S, Kharicha K, Carmaciu C, et al. The relationship between pain intensity and severity and depression in older people: exploratory study. *BMC Fam Pract* 2009; **10**: 54.
759 Iliffe S, Kharicha K, Harari D, Swift C, Gillmann G, Stuck AE. Health risk appraisal in older people 2: the implications for clinicians and commissioners of social isolation risk in older people. *Br J Gen Pract* 2007; **57**: 277–82.
760 Iliffe S, Kharicha K, Harari D, Swift C, Stuck AE. Health risk appraisal for older people in general practice using an expert system: a pilot study. *Health Soc Care Community* 2005; **13**: 21–9.
761 Iliffe S, Swift C, Harari D, Kharicha K, Goodman C, Manthorpe J. Health promotion in later life: public and professional perspectives on an expert system for health risk appraisal. *Prim Health Care Res Dev* 2010; **11**: 187–96. doi: 10.1017/S1463423609990442.
762 Kharicha K, Iliffe S, Harari D, Swift C, Gillmann G, Stuck AE. Health risk appraisal in older people 1: are older people living alone an "at-risk" group? *Br J Gen Pract* 2007; **57**: 271–6.
763 Kharicha K, Iliffe S, Harari D, et al. Feasibility of repeated use of the Health Risk Appraisal for Older people system as a health promotion tool in community-dwelling older people: retrospective cohort study 2001–05. *Age Ageing* 2011; **41**: 128–31. doi: 10.1093/ageing/afr126.
764 Raymond M, Iliffe S, Kharicha K, et al. Health risk appraisal for older people 5: self-efficacy in patient-doctor interactions. *Prim Health Care Res Dev* 2011; **12**: 348–56.
765 Tsakos G, Sheiham A, Iliffe S, et al. The impact of educational level on oral health-related quality of life in older people in London. *Eur J Oral Sci* 2009; **117**: 286-92.
766 Blozik E, Wagner JT, Gillmann G, et al. Social network assessment in community-dwelling older persons: results from a study of three European populations. *Aging Clin Exp Res* 2009; **21**: 150–7.

767 Stuck AE, Elkuch P, Dapp U, Anders J, Iliffe S, Swift CG. Feasibility and yield of a self-administered questionnaire for health risk appraisal in older people in three European countries. *Age Ageing* 2002; **31**: 463–7. doi: 10.1093/ageing/31.6.463.

768 Stuck AE, Kharicha K, Dapp U, et al. Development, feasibility and performance of a health risk appraisal questionnaire for older persons. *BMC Med Res Methodol* 2007; **7**: 1. doi: 10.1186/1471-2288-7-1.

769 Stuck AE, Kharicha K, Dapp U, et al. The PRO-AGE study: An international randomised controlled study of health risk appraisal for older persons based in general practice. *BMC Med Res Methodol* 2007; **7**: 2. doi: 10.1186/1471-2288-7-2.

770 UMIN000031329. *Evaluation of effectiveness of preventive rehabilitation programs for the frail elderly by physical therapist*. University hospital Medical Information Network Center Clinical Trials Registry (UMIN-CTR); 2018. URL: https://upload.umin.ac.jp/cgi-open-bin/ctr/ctr.cgi?function=brows&action=brows&recptno=R000035759&type=summary&language=E (accessed 4 May 2020).

771 Hay WI, Browne G, Roberts J, Jamieson E. Prospective care of elderly patients in family practice. Part 3: Prevalence of unrecognized treatable health concerns. *Can Fam Physician* 1995; **41**: 1695.

772 ACTRN12605000134628. *In-home preventive health assessment and telephone case management for over 75s living alone in independent living units: a cluster randomised controlled trial*. World Health Organization: International Clinical Trials Registry Platform; 2005. URL: https://trialsearch.who.int/Trial2.aspx?TrialID=ACTRN12605000134628 (accessed 11 May 2020).

773 Hendriksen C. An intervention study among elderly people. Methodological and practical experiences. *Scand J Prim Health Care* 1986; **4**: 39–42.

774 Hendriksen C, Lund E, Strømgård E. Use of Social and Health Services by Elderly People during the Terminal 18 Months of Life. *Scand J Soc Med* 1987; **15**: 169–74. doi: 10.1177/140349488701500308.

775 Hendriksen C, Lund E, Stromgard E. Hospitalization of elderly people. A 3-year controlled trial. *J Am Geriatr Soc* 1989; **37**: 117–22.

776 NCT00238836. *Anticipatory & Preventive Team Care (APTCare): At Risk Patients of Family Health Networks*. US National Library of Medicine: ClinicalTrials.gov; 2005. URL: https://clinicaltrials.gov/ct2/show/NCT00238836 (accessed 28 April 2020).

777 Humbert J, Legault F, Dahrouge S, et al. Integration of nurse practitioners into a family health network. *Can Nurse* 2007; **103**: 30–4.

778 Gray D, Armstrong CD, Dahrouge S, Hogg W, Zhang W. Cost-effectiveness of Anticipatory and Preventive multidisciplinary Team Care for complex patients: evidence from a randomized controlled trial. *Can Fam Physician* 2010; **56**: e20–9.

779 Fletcher J, Hogg W, Farrell B, et al. Effect of nurse practitioner and pharmacist counseling on inappropriate medication use in family practice. *Can Fam Physician* 2012; **58**: 862–8.

780 Dahrouge S, Hogg W, Lemelin J, Liddy C, Legault F. Methods for a study of Anticipatory and Preventive multidisciplinary Team Care in a family practice. *Can Fam Physician* 2010; **56**: e73–83.

781 Holland SK, Greenberg J, Tidwell L, Newcomer R. Preventing disability through community-based health coaching. *J Am Geriatr Soc* 2003; **51**: 265–9.

782 Tidwell L, Holland SK, Greenberg J, Malone J, Mullan J, Newcomer R. Community-Based Nurse Health Coaching and Its Effect on Fitness Participation. *Lipincotts Case Manag* 2004; **9**: 267-79. doi: 10.1097/00129234-200411000-00006.

783 Haighton C, Moffatt S, Howel D, et al. The Do-Well study: protocol for a randomised controlled trial, economic and qualitative process evaluations of domiciliary welfare rights advice for socio-economically disadvantaged older people recruited via primary health care. *BMC Public Health* 2012; **12**: 382.

784 Haighton C, Moffatt S, Howel D, et al. Randomised controlled trial with economic and process evaluations of domiciliary welfare rights advice for socioeconomically disadvantaged older people recruited via primary health care (the Do-Well study). *Public Health Res* 2019; **7**: 3. doi: 10.3310/phr07030.

785 ISRCTN37380518. *Advice on welfare rights for disadvantaged older people recruited via primary health care*. BioMed Central: ISRCTN Registry; 2012. URL: http://isrctn.com/ISRCTN37380518 (accessed 11 May 2020).

786 Lin SY, Kerse N, McLean C, Moyes SA. Validation of quality of life and functional measures for older people for telephone administration. *J Prim Health Care* 2010; **2**: 35–42.

787 Wilkinson-Meyers L, Brown P, McLean C, Kerse N. Met and unmet need for personal assistance among community-dwelling New Zealanders 75 years and over. *Health Soc Care Community* 2014; **22**: 317–27.

788 Wham CA, McLean C, Teh R, Moyes S, Peri K, Kerse N. The BRIGHT Trial: what are the factors associated with nutrition risk? *J Nutr Health Aging* 2014; **18**: 692-7.

789 Schäfers A, Martini N, Moyes S, et al. Medication use in community-dwelling older people: pharmacoepidemiology of psychotropic utilisation. *J Prim Health Care* 2014; **6**: 267–78.

790 McLean C, Kerse N, Moyes SA, Ng T, Lin SY, Peri K. Recruiting older people for research through general practice: the Brief Risk Identification Geriatric Health Tool trial. *Australas J Ageing* 2014; **33**: 257–63.
791 ACTRN12606000256572. *A cluster randomised control trial to compare the effects of a restorative home based model versus usual care to improve wellbeing for community dwelling older people*. Australian New Zealand Clinical Trials Registry; 2006. URL: https://www.anzctr.org.au/Trial/Registration/TrialReview.aspx?id=1417 (accessed 21 August 2020).
792 King AII. Creating sustainable home care services for older people [PhD thesis]. Auckland: The University of Auckland; 2010.
793 King AII, Parsons M, Robinson E. A restorative home care intervention in New Zealand: Perceptions of paid caregivers. *Health Soc Care Community* 2012; **20**: 70–9.
794 JPRN-UMIN000006463. *Development and effects of preventive home visit program among ambulatory frail older people living at home*. World Health Organization: International Clinical Trials Registry Platform; 2011. URL: https://trialsearch.who.int/Trial2.aspx?TrialID=JPRN-UMIN000006463 (accessed 11 May 2020).
795 Kono A, Izumi K, Kanaya Y, Tsumura C, Rubenstein LZ. Assessing the quality and effectiveness of an updated preventive home visit programme for ambulatory frail older Japanese people: research protocol for a randomized controlled trial. *J Adv Nurs* 2014; **70**: 2363–72.
796 JPRN-UMIN000001113. *Effects of preventive home visits for ambulatory frail elders: a randomized clinical trial in Osaka, Japan*. World Health Organization: International Clinical Trials Registry Platform; 2008. URL: https://trialsearch.who.int/Trial2.aspx?TrialID=JPRN-UMIN000001113 (accessed 11 May 2020).
797 Kono A, Fujita T, Tsumura C, Kondo T, Kushiyama K, Rubenstein LZ. Preventive home visit model targeted to specific care needs of ambulatory frail elders: preliminary report of a randomized trial design. *Aging Clin Exp Res* 2009; **21**: 167–73.
798 Kono A, Kanaya Y, Tsumura C, Rubenstein LZ. Effects of preventive home visits on health care costs for ambulatory frail elders: a randomized controlled trial. *Aging Clin Exp Res* 2013; **25**: 575–81.
799 Kukkonen-Harjula K, Karmeniemi P, Suikkanen S, Sipil, Pitkala K, Hupli M. Long-term home-based physiotherapy for older people with signs of frailty or consequent to a hip fracture operation-Design of RCT (NCT02305433). *Eur Geriatr Med* 2016; **7**: S149-.
800 NCT02305433. *Effects of Long-term Intensive Home-based Physiotherapy on Older People With an Operated Hip Fracture or Frailty (RCT)*. US National Library of Medicine: ClinicalTrials.gov; 2014. URL: https://clinicaltrials.gov/show/NCT02305433 (accessed 11 May 2020).
801 Soukkio P, Suikkanen S, Kaaria S, et al. Effects of 12-month home-based physiotherapy on duration of living at home and functional capacity among older persons with signs of frailty or with a recent hip fracture - protocol of a randomized controlled trial (HIPFRA study). *BMC Geriatr* 2018; **18**: 232.
802 Suikkanen S, Soukkio P, Pitkala K, et al. Older persons with signs of frailty in a home-based physical exercise intervention: baseline characteristics of an RCT. *Aging Clin Exp Res* 2019; **31**: 1419–27.
803 Suikkanen SA, Soukkio PK, Aartolahti EM, et al. Effects of Home-Based Physical Exercise on Days at Home and Cost-Effectiveness in Pre-Frail and Frail Persons: Randomized Controlled Trial. *J Am Med Dir Assoc* 2020; **18**: 18. doi: 10.1016/j.jamda.2020.06.005.
804 Domènech-Abella J, Switsers L, Mundó J, Dierckx E, Dury S, De Donder L. The association between perceived social and physical environment and mental health among older adults: mediating effects of loneliness. *Aging Ment Health* 2021; **25**: 962-8. doi: 10.1080/13607863.2020.1727853.
805 Duppen D, Rossi G, Dierckx E, Hoeyberghs L, De Donder L. Focusing on positive outcomes in frailty research: development of a short well-being instrument for older adults (SWIO). *Int Psychogeriatr* 2019; **31**: 767–77. doi: 10.1017/S1041610219000401.
806 Dury S, Dierckx E, van der Vorst A, et al. Detecting frail, older adults and identifying their strengths: results of a mixed-methods study. *BMC Public Health* 2018; **18**: 191. doi: 10.1186/s12889-018-5088-3.
807 NCT03168204. *Evaluating the Efficacy of a Detection and Prevention Program for Frail Community-dwelling Older Adults (D-SCOPE)*. US National Library of Medicine: ClinicalTrials.gov; 2017. URL: https://clinicaltrials.gov/ct2/show/NCT03168204 (accessed 21 August 2020).
808 Smetcoren A-S, Dury S, den Donder L, Dierckx E. D-SCOPE: towards a positive view on prevention in frail elderly [D-SCOPE: naar een positieve kijk op preventie bij kwetsbare ouderen]. *Geron* 2017; **19**: 35–8. doi: 10.1007/s40718-017-0010-0.
809 Switsers L, Dierckx E, Domènech-Abella J, De Donder L, Dury S. Negative old-age life events and well-being in later life: the moderating and mediating role of loneliness. *Int Psychogeriatr* 2021; **33**: 1265-76. doi: 10.1017/S1041610220004196.

810 Van der Elst MCJ, Schoenmakers B, Op het Veld LPM, et al. Concordances and differences between a unidimensional and multidimensional assessment of frailty: a cross-sectional study. *BMC Geriatr* 2019; **19**: 346. doi: 10.1186/s12877-019-1369-7.

811 van der Vorst A, Zijlstra GAR, De Witte N, et al. Explaining discrepancies in self-reported quality of life in frail older people: a mixed-methods study. *BMC Geriatr* 2017; **17**: 251. doi: 10.1186/s12877-017-0641-y.

812 Leung AC, Liu CP, Tsui LL, et al. The use of the Minimum Data Set. Home Care in a case management project in Hong Kong. *Care Manag J* 2001; **3**: 8–13.

813 Phelan EA, Williams B, Penninx BWJH, LoGerfo JP, Leveille SG. Activities of daily living function and disability in older adults in a randomized trial of the Health Enhancement Program. *J Gerontol A Biol Sci Med Sci* 2004; **59**: 838–43.

814 Phelan EA, Cheadle A, Schwartz SJ, et al. Promoting health and preventing disability in older adults: lessons from intervention studies carried out through an academic-community partnership. *Fam Community Health* 2003; **26**: 214–20.

815 Lewin G, Allan J, Patterson C, Knuiman M, Boldy D, Hendrie D. A comparison of the home-care and healthcare service use and costs of older Australians randomised to receive a restorative or a conventional home-care service. *Health Soc Care Community* 2014; **22**: 328–36.

816 Lewin G, Concanen K, Youens D. The home independence program with non-health professionals as care managers: An evaluation. *Clin Interv Aging* 2016; **11**: 807–17.

817 Lewin G, Vandermeulen S. A non-randomised controlled trial of the Home Independence Program (HIP): an Australian restorative programme for older home-care clients. *Health Soc Care Community* 2010; **18**: 91–9.

818 ACTRN12616001411437. *Evaluation of preventive home visits in elderly over 75 years*. Australian New Zealand Clinical Trials Registry; 2016. URL: https://anzctr.org.au/Trial/Registration/TrialReview.aspx?ACTRN=12616001411437 (accessed 11 May 2020).

819 Liimatta H, Lampela P, Kautiainen H, Laitinen-Parkkonen P, Pitkala KH. The Effects of Preventive Home Visits on Older People's Use of Health Care and Social Services and Related Costs. *J Gerontol A Biol Sci Med Sci* 2019; **29**: 29. doi: 10.1093/gerona/glz139.

820 Liimatta H, Lampela P, Laitinen-Parkkonen P, Pitkala KH. Preventive home visits to promote the health-related quality of life of home-dwelling older people: baseline findings and feasibility of a randomized, controlled trial. *Eur Geriatr Med* 2017; **8**: 440-5.

821 ISRCTN22749696. *CERgAS: multiComponent Exercise and theRApeutic lifeStyle intervention to improve physical function and maintain independent living among urban poor older people*. BioMed Central: ISRCTN Registry; 2014. URL: http://www.isrctn.com/ISRCTN22749696 (accessed 5 November 2020).

822 Rosli R, Loh D, Choo W, et al. Effects of multicomponent exercise and therapeutic lifestyle (CERgAS) intervention on cognitive function in lower income elderly population: A cluster randomised controlled trial. *Age Ageing* 2017; **46**: ii7. doi: 10.1093/ageing/afx118.26.

823 ACTRN12617000198325. *Efficacy and cost-effectiveness of a community based model of care for older patients with complex needs: a study protocol for a multicentre randomized controlled trial using a stepped wedge cluster design*. Australian New Zealand Clinical Trials Registry; 2017. URL: https://www.anzctr.org.au/Trial/Registration/TrialReview.aspx?id=372235 (accessed 11 May 2020).

824 Kinchin I, Jacups S, Mann J, et al. Efficacy and cost-effectiveness of a community-based model of care for older patients with complex needs: a study protocol for a multicentre randomised controlled trial using a stepped wedge cluster design. *Trials* 2018; **19**: 668.

825 Mann J, Quigley R, Harvey D, Tait M, Williams G, Strivens E. OPEN ARCH: integrated care at the primary–secondary interface for the community-dwelling older person with complex needs. *Aust J Prim Health* 2020; **26**: 104–8. doi: 10.1071/PY19184.

826 Mann J, Thompson F, Quigley R, McDermott R, Devine S, Strivens E. Beyond multimorbidity: primary care and the older person with complex needs. *Aust J Prim Health* 2021; **27**: 194–201. doi: 10.1071/PY20125.

827 Quigley R, Russell S, Harvey D, Mann J. OPEN ARCH integrated care model: experiences of older Australians and their carers. *Aust J Prim Health* 2021; **27**: 236–42. doi: 10.1071/py20203.

828 Markle-Reid M. Frail elderly home care clients: the effects and expense of adding nursing health promotion and preventative care to personal support services [PhD thesis]. Hamilton, Ontario: McMaster University; 2002.

829 Markle-Reid M, Weir R, Browne G, Henderson S, Roberts J, Gafni A. Frail elderly homecare clients: The costs and effects of adding nursing health promotion and preventive care to personal support services. Ottawa, Ontario: Canadian Health Services Research Foundation, 2003.

830 Houles M. Cost-effectiveness analysis of a multidisciplinary intervention for vulnerable elderly individuals living at home. [French]. *Cah de l'Annee Gerontol* 2010; **2**: 169–72.
831 Houles M. Randomised study of a multi-disciplinary programme focusing on geriatric syndromes in vulnerable, elderly individuals living at home (Dutch EASYcare Study). [French]. *Cah de l'Annee Gerontol* 2010; **2**: 166–8.
832 Melis RJ, Adang E, Teerenstra S, et al. Cost-effectiveness of a multidisciplinary intervention model for community-dwelling frail older people. *J Gerontol A Biol Sci Med Sci* 2008; **63**: 275–82.
833 Melis RJ, Teerenstra S, Rikkert MG, Borm GF. Pseudo cluster randomization performed well when used in practice. *J Clin Epidemiol* 2008; **61**: 1169–75.
834 Melis RJ, van Eijken MI, Boon ME, Olde Rikkert MG, van Achterberg T. Process evaluation of a trial evaluating a multidisciplinary nurse-led home visiting programme for vulnerable older people. *Disabil Rehabil* 2010; **32**: 937–46.
835 Melis RJ, van Eijken MI, Borm GF, et al. The design of the Dutch EASYcare study: a randomised controlled trial on the effectiveness of a problem-based community intervention model for frail elderly people [NCT00105378]. *BMC Health Serv Res* 2005; **5**: 65.
836 NCT00105378. *Dutch EASYcare Study*. US National Library of Medicine: ClinicalTrials.gov; 2005. URL: https://clinicaltrials.gov/ct2/show/NCT00105378 (accessed 28 April 2020).
837 Friedman B, Li Y, Liebel DV, Powers BA. Effects of a home visiting nurse intervention versus care as usual on individual activities of daily living: a secondary analysis of a randomized controlled trial. *BMC Geriatr* 2014; **14**: 24.
838 Friedman B, Wamsley BR, Liebel DV, Saad ZB, Eggert GM. Patient satisfaction, empowerment, and health and disability status effects of a disease management–health promotion nurse intervention among Medicare beneficiaries with disabilities. *Gerontol* 2009; **49**: 778–92.
839 Li Y, Liebel DV, Friedman B. An investigation into which individual instrumental activities of daily living are affected by a home visiting nurse intervention. *Age Ageing* 2013; **42**: 27–33.
840 Liebel DV. Process evaluation of a nurse home visiting intervention that postpones disability worsening in older adults. *Diss Abst Int Pt B - Sci & Eng* 2008; **68**: 5860.
841 Liebel DV, Powers BA, Friedman B, Watson NM. Barriers and facilitators to optimize function and prevent disability worsening: A content analysis of a nurse home visit intervention. *J Adv Nurs* 2012; **68**: 80–93.
842 Meng H, Friedman B, Dick AW, et al. Impact of a disease management-health promotion nurse intervention on personal assistance use and expenditures. *Home Health Care Serv Q* 2009; **28**: 113–29.
843 Meng H, Friedman B, Dick AW, Wamsley BR, Eggert GM, Mukamel D. Effect of a voucher benefit on the demand for paid personal assistance. *Gerontologist* 2006; **46**: 183–92.
844 Keijser W, Bond R, Saez I, Vandewoude M. Health monitoring and sOcial integration environMent for Supporting WidE ExTension of independent life at HOME (Home Sweet Home): Trial Protocol Version 1.0. Antwerp: Zorgbedrijf Antwerpen, 2010.
845 NCT01218373. *The Clinical Evaluation of Continuous Assistance Offered to Older People Living Independently*. US National Library of Medicine: ClinicalTrials.gov; 2010. URL: https://ClinicalTrials.gov/show/NCT01218373 (accessed 11 May 2020).
846 Daniels R, van Rossum E, Metzelthin S, et al. A disability prevention programme for community-dwelling frail older persons. *Clin Rehabil* 2011; **25**: 963–74.
847 ISRCTN31954692. *The reduction of disability in community-dwelling frail elderly*. BioMed Central: ISRCTN Registry; 2009. URL: http://isrctn.com/ISRCTN31954692 (accessed 11 May 2020).
848 Metzelthin SF, Daniëls R, van Rossum E, et al. A nurse-led interdisciplinary primary care approach to prevent disability among community-dwelling frail older people: A large-scale process evaluation. *Int J Nurs Stud* 2013; **50**: 1184–96. doi: 10.1016/j.ijnurstu.2012.12.016.
849 Metzelthin SF, Van Rossum E, De Witte LP, et al. Frail elderly people living at home; effects of an interdisciplinary primary care programme. [Dutch]. *Ned Tijdschr Geneeskd* 2014; **158**: A7355.
850 Metzelthin SF, van Rossum E, de Witte LP, et al. The reduction of disability in community-dwelling frail older people: design of a two-arm cluster randomized controlled trial. *BMC Public Health* 2010; **10**: 511–.
851 Metzelthin SF, van Rossum E, Hendriks MR, et al. Reducing disability in community-dwelling frail older people: cost-effectiveness study alongside a cluster randomised controlled trial. *Age Ageing* 2015; **44**: 390–6.
852 Beishuizen CRL, Coley N, Moll van Charante EP, van Gool WA, Richard E, Andrieu S. Determinants of Dropout and Nonadherence in a Dementia Prevention Randomized Controlled Trial: The Prevention of Dementia by Intensive Vascular Care Trial. *J Am Geriatr Soc* 2017; **65**: 1505–13.
853 Bussel EF, Richard E, Busschers WB, et al. A cardiovascular risk prediction model for older people: Development and validation in a primary care population. *J Clin Hypertens* 2019; **21**: 1145–52.

854 ISRCTN29711771. *Prevention of dementia by intensive vascular care*. BioMed Central: ISRCTN Registry; 2006. URL: http://www.isrctn.com/ISRCTN29711771 (accessed 28 April 2020).
855 Ligthart SA, Richard E, van Gool WA, Moll van Charante EP. Cardiovascular risk management in community-dwelling elderly: opportunities for prevention. *European Journal of Preventive Cardiology* 2012; **19**: 1365–72.
856 Ligthart SA, van den Eerenbeemt KD, Pols J, van Bussel EF, Richard E, Moll van Charante EP. Perspectives of older people engaging in nurse-led cardiovascular prevention programmes: a qualitative study in primary care in the Netherlands. *Br J Gen Pract* 2015; **65**: e41–8.
857 Richard E, Ligthart S, van Charante EM, Van Gool W. Methodological issues in a cluster-randomized trial to prevent dementia by intensive vascular care. *J Nutr Health Aging* 2010; **14**: 315–7.
858 Richard E, Van den Heuvel E, van Charante EPM, et al. Prevention of dementia by intensive vascular care (PreDIVA): a cluster-randomized trial in progress. *Alzheimer Dis Assoc Disord* 2009; **23**: 198–204.
859 van Bussel EF, Hoevenaar-Blom MP, Busschers WB, et al. Effects of Primary Cardiovascular Prevention on Vascular Risk in Older Adults. *Am J Prev Med* 2018; **55**: 368–75.
860 van Dalen JW, Moll van Charante EP, Caan MWA, et al. Effect of Long-Term Vascular Care on Progression of Cerebrovascular Lesions: Magnetic Resonance Imaging Substudy of the PreDIVA Trial (Prevention of Dementia by Intensive Vascular Care). *Stroke* 2017; **48**: 1842–8.
861 van Dalen JW, Moll van Charante EP, van Gool WA, Richard E. Discontinuation of Antihypertensive Medication, Cognitive Complaints, and Incident Dementia. *J Am Med Dir Assoc* 2019; **20**: 1091–7. doi: 10.1016/j.jamda.2018.12.006.
862 van Dalen JW, Van Wanrooij LL, van Charante EPM, Richard E, van Gool WA, Moll van Charante EP. Apathy is associated with incident dementia in community-dwelling older people. *Neurology* 2018; **90**: e82–e9.
863 Monteserin R, Brotons C, Moral I, et al. Effectiveness of a geriatric intervention in primary care: a randomized clinical trial. *Fam Pract* 2010; **27**: 239–45.
864 Morey MC, Sloane R, Pieper CF, et al. Effect of Physical Activity Guidelines on Physical Function in Older Adults. *J Am Geriatr Soc* 2008; **56**: 1873–8. doi: 10.1111/j.1532-5415.2008.01937.x.
865 Peterson MJ, Sloane R, Cohen HJ, et al. Effect of telephone exercise counseling on frailty in older veterans: Project LIFE. *Am J Mens Health* 2007; **1**: 326–34.
866 Hall KS, Sloane R, Pieper CF, et al. Long-term changes in physical activity following a one-year home-based physical activity counseling program in older adults with multiple morbidities. *J Aging Res* 2011; **2011**: 308407. doi: 10.4061/2011/308407.
867 Huffman KM, Sloane R, Peterson MJ, et al. The impact of self-reported arthritis and diabetes on response to a home-based physical activity counselling intervention. *Scand J Rheumatol* 2010; **39**: 233-9.
868 Morey MC, Peterson MJ, Pieper CF, et al. Project LIFE--Learning to Improve Fitness and Function in Elders: methods, design, and baseline characteristics of randomized trial. *J Rehabil Res Dev* 2008; **45**: 31–42.
869 NCT00435188. *Life 2: Improving Fitness and Function in Elders (Project LIFE)*. US National Library of Medicine: ClinicalTrials.gov; 2007. URL: https://clinicaltrials.gov/ct2/show/NCT00435188 (accessed 20 June 2020).
870 Haase AM, Taylor AH, Fox KR, Thorp H, Lewis G. Rationale and development of the physical activity counselling intervention for a pragmatic TRial of Exercise and Depression in the UK (TREAD-UK). *Ment Health Phys Act* 2010; **3**: 85–91. doi: 10.1016/j.mhpa.2010.09.004.
871 ISRCTN80470273. *The PACE Study: physical activity facilitation for older adults*. BioMed Central: ISRCTN Registry; 2013. URL: http://www.isrctn.com/ISRCTN80470273 (accessed 20 June 2020).
872 Morgan GS, Haase AM, Campbell R, Ben-Shlomo Y. Physical ACtivity facilitation for Elders (PACE): study protocol for a randomised controlled trial. *Trials* 2015; **16**: 91.
873 Newbury JW. 75+ Health assessments: a randomised controlled trial [MD thesis]. Adelaide: Adelaide University; 2001.
874 Graves MT, Slater MA, Maravilla V, Reissler L, Faculjak P, Newcomer RJ. Implementing an early intervention case management program in three medical groups. *Case Manag* 2003; **14**: 48–52. doi: 10.1016/S1061-9259(03)00212-1.
875 Maravilla V, Graves MT, Newcomer R. Development of a standardized language for case management among high-risk elderly. *Lippincotts Case Manag* 2005; **10**: 3–13. doi: 10.1097/00129234-200501000-00002.
876 NCT00973258. *Randomized Controlled Trial of Community-based Nutritional, Physical and Cognitive Training Intervention Programmes for At Risk Frail Elderly (FIT)*. US National Library of Medicine: ClinicalTrials.gov; 2009. URL: https://clinicaltrials.gov/ct2/show/NCT00973258 (accessed 28 April 2020).

877 Ng TP, Ling LHA, Feng L, et al. Cognitive Effects of Multi-Domain Interventions Among Pre-Frail and Frail Community-Living Older Persons: Randomized Controlled Trial. *J Gerontol A Biol Sci Med Sci* 2018; **73**: 806–12.
878 ACTRN12608000027314. *Examination of the effect of a designated client centred goal facilitation tool on service provision, quality of life and independence among older people*. Australian New Zealand Clinical Trials Registry; 2008. URL: https://www.anzctr.org.au/Trial/Registration/TrialReview.aspx?id=82511&isReview=true (accessed 8 June 2020).
879 Parsons JG, Sheridan N, Rouse P, Robinson E, Connolly M. A randomized controlled trial to determine the effect of a model of restorative home care on physical function and social support among older people. *Arch Phys Med Rehabil* 2013; **94**: 1015–22.
880 Parsons M, Senior HE, Kerse N, et al. The Assessment of Services Promoting Independence and Recovery in Elders Trial (ASPIRE): a pre-planned meta-analysis of three independent randomised controlled trial evaluations of ageing in place initiatives in New Zealand. *Age Ageing* 2012; **41**: 722–8.
881 Parsons M, Anderson C, Senior H, et al. ASPIRE: Assessment of Services Promoting Independence and Recovery in Elders: New Zealand Ministry of Health, 2006.
882 Auckland UniServices Limited. An economic evaluation of the Assessment of Service Promoting Independence and Recovery in Elders (ASPIRE) - final report: New Zealand Ministry of Health, 2006.
883 ACTRN12605000140651. *Assessment of Services Promoting Independence and Recovery in Elders*. Australian New Zealand Clinical Trials Registry; 2005. URL: http://www.anzctr.org.au/ACTRN12605000140651.aspx (accessed 11 May 2020).
884 NCT00134836. *Preventive Primary Care Outreach for High Risk Older Persons*. US National Library of Medicine: ClinicalTrials.gov; 2004. URL: https://ClinicalTrials.gov/show/NCT00134836 (accessed 11 May 2020).
885 Dapp U, Anders J, von Renteln-Kruse W, Golgert S, Meier-Baumgartner HP, Minder CE. The longitudinal urban cohort ageing study (LUCAS): study protocol and participation in the first decade. *BMC Geriatr* 2012; **12**: 35. doi: 10.1186/1471-2318-12-35.
886 Dapp U, Anders JA, von Renteln-Kruse W, et al. A randomized trial of effects of health risk appraisal combined with group sessions or home visits on preventive behaviors in older adults. *J Gerontol A Biol Sci Med Sci* 2011; **66**: 591–8. doi: 10.1093/gerona/glr021.
887 von Renteln-Kruse W, Anders J, Dapp U, Meier-Baumgartner HP. Präventive Hausbesuche durch einespeziell fortgebildete Pflegefachkraft bei 60-jährigen undälteren Personen in Hamburg. *Z Gerontol Geriatr* 2003; **36**: 378–91. doi: 10.1007/s00391-003-0179-9.
888 Rockwood K, Howlett S, Stadnyk K, Carver D, Powell C, Stolee P. Responsiveness of goal attainment scaling in a randomized controlled trial of comprehensive geriatric assessment. *J Clin Epidemiol* 2003; **56**: 736–43.
889 NCT01969526. *Effectiveness of a Multifactorial Intervention on Frailty*. US National Library of Medicine: ClinicalTrials.gov; 2013. URL: https://clinicaltrials.gov/ct2/show/NCT01969526 (accessed 27 August 2021).
890 Romera L, Orfila F, Segura JM, et al. Effectiveness of a primary care based multifactorial intervention to improve frailty parameters in the elderly: a randomised clinical trial: rationale and study design. *BMC Geriatr* 2014; **14**: 125.
891 Metzelthin SF, Rooijackers TH, Zijlstra GAR, et al. Effects, costs and feasibility of the 'Stay Active at Home' Reablement training programme for home care professionals: study protocol of a cluster randomised controlled trial. *BMC Geriatr* 2018; **18**: 276.
892 Metzelthin SF, Zijlstra GA, van Rossum E, et al. 'Doing with ...' rather than 'doing for ...' older adults: Rationale and content of the 'Stay Active at Home' programme. *Clin Rehabil* 2017; **31**: 1419–30.
893 NCT03293303. *The Feasibility, Effects and Costs of the 'Stay Active at Home Programme'*. US National Library of Medicine: ClinicalTrials.gov; 2017. URL: https://clinicaltrials.gov/ct2/show/NCT03293303 (accessed 28 April 2020).
894 Rooijackers TH, Zijlstra GAR, van Rossum E, et al. Process evaluation of a reablement training program for homecare staff to encourage independence in community-dwelling older adults. *BMC Geriatr* 2021; **21**: 5. doi: 10.1186/s12877-020-01936-7.
895 Smeets RGM, Kempen GIJM, Zijlstra GAR, et al. Experiences of home-care workers with the 'Stay Active at Home' programme targeting reablement of community-living older adults: An exploratory study. *Health Soc Care Community* 2020; **28**: 291-9. doi: 10.1111/hsc.12863.
896 Ryvicker M, Marren J, Sobolewski S, et al. Spreading improvement strategies within a large home healthcare organization. *J Healthc Qual* 2008; **30**: 48–58.
897 Serra-Prat M. Clinical and economic assessment of a pre-frail screening program. NCT02138968. 2014. https://ClinicalTrials.gov/show/NCT02138968 (accessed 7 February, 2020).

898 Sherman H. Preventive home visits for 75 years old persons by the district nurse [thesis]. Stockholm: Karolinska Institutet; 2012.
899 Fleuren MAH, Vrijkotte S, Jans MP, et al. The implementation of the functional task exercise programme for elderly people living at home. *BMC Musculoskelet Disord* 2012; **13**: 1–9.
900 NTR2407. *Training of the ability to live independently 75+*. Netherlands National Trial Register; 2010. URL: https://www.trialregister.nl/trial/2280 (accessed 8 May 2020).
901 Stewart S, Lloyd-Smith W, Poland F. Running a community clinical trial: lessons from the CAMELOT project. *Br J Ther Rehabil* 2003; **10**: 443–8. doi: 10.12968/bjtr.2003.10.10.13475.
902 Flood C, Mugford M, Stewart S, Harvey I, Poland F, Lloyd-Smith W. Occupational therapy compared with social work assessment for older people. An economic evaluation alongside the CAMELOT randomised controlled trial. *Age Ageing* 2005; **34**: 47–52.
903 Alessi CA, Stuck AE, Aronow HU, et al. The process of care in preventive in-home comprehensive geriatric assessment. *J Am Geriatr Soc* 1997; **45**: 1044–50.
904 Büla CJ, Alessi CA, Aronow HU, et al. Community physicians' cooperation with a program of in-home comprehensive geriatric assessment. *J Am Geriatr Soc* 1995; **43**: 1016-20.
905 Büla CJ, Bérod AC, Stuck AE, et al. Effectiveness of preventive in-home geriatric assessment in well functioning, community-dwelling older people: secondary analysis of a randomized trial. *J Am Geriatr Soc* 1999; **47**: 389-95.
906 Cho CY, Alessi CA, Cho M, et al. The association between chronic illness and functional change among participants in a comprehensive geriatric assessment program. *J Am Geriatr Soc* 1998; **46**: 677–82.
907 Rubenstein LZ, Aronow HU, Schloe M, et al. A home-based geriatric assessment, follow-up and health promotion program: design, methods, and baseline findings from a 3-year randomized clinical trial. *Aging Clin Exp Res* 1994; **6**: 105–20.
908 Stuck AE, Gafner Zwahlen H, Neuenschwander BE, Meyer Schweizer RA, Bauen G, Beck JC. Erratum for: Methodologic challenges of randomized controlled studies on in-home comprehensive geriatric assessment: The EIGER project. Aging Clin Exp Res 1995;7:218-23. *Aging Clin Exp Res* 1995; **7**: 237.
909 Stuck AE, Gafner Zwahlen H, Neuenschwander BE, Meyer Schweizer RA, Bauen G, Beck JC. Erratum for: Methodologic challenges of randomized controlled studies on in-home comprehensive geriatric assessment: The EIGER project. Aging Clin Exp Res 1995;7:218-23. *Aging Clin Exp Res* 1998; **10**: 348.
910 Stuck AE, Stuckelberger A, Gafner Zwahlen H, Minder CE, Beck JC. A randomised trial of in-home preventive visits with annual comprehensive geriatric assessment: Base-line findings of the EIGER project. [French]. *Med Hyg (Geneve)* 1995; **53**: 2385–97.
911 Stuck AE, Gafner Zwahlen H, Neuenschwander BE, Meyer Schweizer RA, Bauen G, Beck JC. Methodologic challenges of randomized controlled studies on in-home comprehensive geriatric assessment: The EIGER project. *Aging Clin Exp Res* 1995; **7**: 218–23.
912 ISRCTN28458424. *Disability prevention in the older population: use of information technology for health risk appraisal and prevention of functional decline*. BioMed Central: ISRCTN Registry; 2005. URL: http://www.isrctn.com/ISRCTN28458424 (accessed 20 May 2020).
913 NL2535. *FIT-study: Maintaining Functionality in Transition*. Netherlands National Trial Register; 2010. URL: https://www.trialregister.nl/trial/2535 (accessed 28 April 2020).
914 Suijker J, MacNeil-Vroomen J, Van Rijn M, et al. Economic evaluation of nurse-led multifactorial care to prevent or postpone new disabilities in community-living older people: results of a cluster randomized trial. *Eur Geriatr Med* 2016; **7**: S103-.
915 Suijker JJ, Buurman BM, ter Riet G, et al. Comprehensive geriatric assessment, multifactorial interventions and nurse-led care coordination to prevent functional decline in community-dwelling older persons: protocol of a cluster randomized trial. *BMC Health Serv Res* 2012; **12**: 85.
916 Suijker JJ, MacNeil-Vroomen JL, van Rijn M, et al. Cost-effectiveness of nurse-led multifactorial care to prevent or postpone new disabilities in community-living older people: Results of a cluster randomized trial. *PLoS One* 2017; **12**: e0175272.
917 Suijker JJ, van Rijn M, Ter Riet G, Moll van Charante EP, de Rooij SE, Buurman BM. Minimal Important Change and Minimal Detectable Change in Activities of Daily Living in Community-Living Older People. *J Nutr Health Aging* 2017; **21**: 165–72.
918 Van Rijn M, Hoogteijling N, Suijker J, De Rooij S, Buurman B, Van Charante EM. Preventive home-visits and nurse-led care coordination: a qualitative study on the experiences, needs and preferences of community dwelling older people. *Eur Geriatr Med* 2016; **7**: S103-S4.
919 Szanton SL, Leff B, Li Q, et al. CAPABLE program improves disability in multiple randomized trials. *J Am Geriatr Soc* 2021; **69**: 3631-40. doi: 10.1111/jgs.17383.
920 Szanton SL, Tanner EK, Thorpe RJ, et al. A multi-component pilot to enhance aging-in-place capacity for low-income older adults. *Clin Transl Sci* 2010; **3**: S20.

921 LaFave SE, Granbom M, Cudjoe TKM, Gottsch A, Shorb G, Szanton SL. Attention control group activities and perceived benefit in a trial of a behavioral intervention for older adults. *Res Nurs Health* 2019; **42**: 476–82.
922 NCT01576133. *Reducing Disability Via a Bundled Bio-Behavioral-Environmental Approach (CAPABLE)*. US National Library of Medicine: ClinicalTrials.gov; 2012. URL: https://clinicaltrials.gov/ct2/show/study/NCT01576133 (accessed 28 April 2020).
923 NCT01743495. *CAPABLE for Frail Dually Eligible Older Adults (CAPABLE500)*. US National Library of Medicine: ClinicalTrials.gov; 2012. URL: https://clinicaltrials.gov/ct2/show/NCT01743495 (accessed 28 April 2020).
924 Nkimbeng M, Roberts L, Thorpe Jr RJ, et al. Recruiting older adults with functional difficulties into a community-based research study: Approaches and costs. *J Appl Gerontol* 2020; **39**: 644–50. doi: 10.1177/0733464818786612.
925 Patadia P, Roberts L, Szanton S. Are difficulties with daily activities related to neighborhood? A geospatial analysis of CAPABLE baseline data. *J Am Geriatr Soc* 2018; **66**: S289.
926 Smith PD, Becker K, Roberts L, Walker J, Szanton SL. Associations among pain, depression, and functional limitation in low-income, home-dwelling older adults: An analysis of baseline data from CAPABLE. *Geriatr Nurs* 2016; **37**: 348–52.
927 Szanton SL, Alfonso YN, Leff B, et al. Medicaid cost savings of a preventive home visit program for disabled older adults. *J Am Geriatr Soc* 2018; **66**: 614–20.
928 Szanton SL, Wolff JL, Leff B, et al. Preliminary data from Community Aging in Place, Advancing Better Living for Elders, a patient-directed, team-based intervention to improve physical function and decrease nursing home utilization: the first 100 individuals to complete a Centers for Medicare and Medicaid Services innovation project. *J Am Geriatr Soc* 2015; **63**: 371–4. doi: 10.1111/jgs.13245.
929 Szanton SL, Wolff JW, Leff B, et al. CAPABLE trial: a randomized controlled trial of nurse, occupational therapist and handyman to reduce disability among older adults: rationale and design. *Contemp Clin Trials* 2014; **38**: 102–12.
930 Waldersen BW, Wolff JL, Roberts L, Bridges AE, Gitlin LN, Szanton SL. Functional goals and predictors of their attainment in low-income community-dwelling older adults. *Arch Phys Med Rehabil* 2017; **98**: 896–903.
931 NCT01056640. *Telemonitoring Versus Usual Care*. US National Library of Medicine: ClinicalTrials.gov; 2010. URL: https://clinicaltrials.gov/ct2/show/study/NCT01056640 (accessed 18 May 2021).
932 Pecina JL, Hanson GJ, Van Houten H, Takahashi PY. Impact of telemonitoring on older adults health-related quality of life: the Tele-ERA study. *Qual Life Res* 2013; **22**: 2315–21. doi: 10.1007/s11136-013-0361-5.
933 Pecina JL, Vickers KS, Finnie DM, Hathaway JC, Hanson GJ, Takahashi PY. Telemonitoring Increases Patient Awareness of Health and Prompts Health-Related Action: Initial Evaluation of the TELE-ERA Study. *Telemed E Health* 2011; **17**: 461–6. doi: 10.1089/tmj.2010.0213.
934 Takahashi PY, Hanson GJ, Pecina JL, et al. A randomized controlled trial of telemonitoring in older adults with multiple chronic conditions: the Tele-ERA study. *BMC Health Serv Res* 2010; **10**: 255.
935 Takahashi PY, Hanson GJ, Thorsteinsdottir B, et al. The impact of telemonitoring upon hospice referral in the community: a randomized controlled trial. *Clin Interv Aging* 2012; **7**: 445–51. doi: 10.2147/CIA.S36461.
936 Upatising B, Hanson GJ, Kim YL, Cha SS, Yih Y, Takahashi PY. Effects of home telemonitoring on transitions between frailty states and death for older adults: a randomized controlled trial. *Int J Gen Med* 2013; **6**: 145–51. doi: 10.2147/IJGM.S40576.
937 Upatising B, Wood DL, Kremers WK, et al. Cost comparison between home telemonitoring and usual care of older adults: a randomized trial (Tele-ERA). *Telemed E Health* 2015; **21**: 3–8.
938 NCT00974506. *Pilot Study: Complementary Therapies in Geriatric Patients*. US National Library of Medicine: ClinicalTrials.gov; 2009. URL: https://clinicaltrials.gov/ct2/show/NCT00974506 (accessed 21 August 2020).
939 DRKS00011831. *A multimodal intervention program for older people with frailty – a randomized controlled pilot trial*. World Health Organization: International Clinical Trials Registry Platform; 2017. URL: https://trialsearch.who.int/Trial2.aspx?TrialID=DRKS00011831 (accessed 11 May 2020).
940 Ziller C, Braun T, Thiel C. Frailty phenotype prevalence in community-dwelling older adults according to physical activity assessment method. *Clin Interv Aging* 2020; **15**: 343–55. doi: 10.2147/CIA.S238204.
941 Kjerstad E, Tuntland HK. Reablement in community-dwelling older adults: a cost-effectiveness analysis alongside a randomized controlled trial. *Health Econ Rev* 2016; **6**: 15.
942 NCT02043262. *The Effectiveness of Reablement in Home Dwelling Older Adults. A Randomized Controlled Trial*. US National Library of Medicine: ClinicalTrials.gov; 2012. URL: https://clinicaltrials.gov/ct2/show/NCT02043262 (accessed 28 April 2020).

943 Tuntland H, Espehaug B, Forland O, Hole AD, Kjerstad E, Kjeken I. Reablement in community-dwelling adults: study protocol for a randomised controlled trial. *BMC Geriatr* 2014; **14**: 139.
944 NTR5184. *PROTO-study: Prevention of undernutrition in community dwelling older adults*. Netherlands National Trial Register; 2015. URL: https://www.trialregister.nl/trial/5045 (accessed 21 August 2020).
945 Dorhout BG, Haveman-Nies A, van Dongen EJI, et al. Cost-effectiveness of a Diet and Resistance Exercise Intervention in Community-Dwelling Older Adults: ProMuscle in Practice. *J Am Med Dir Assoc* 2021; **22**: 792–. doi: 10.1016/j.jamda.2020.12.036.
946 NTR6038. *ProMuscle in Practice: Effectiveness of a combined resistance exercise and nutrition intervention to promote maintenance of physical functioning of community-dwelling elderly in a real-life setting*. Netherlands National Trial Register; 2016. URL: https://www.trialregister.nl/trial/5858 (accessed 16 May 2020).
947 van Dongen EJI, Doets EL, de Groot LCPGM, Dorhout BG, Haveman-Nies A. Process Evaluation of a Combined Lifestyle Intervention for Community-Dwelling Older Adults: ProMuscle in Practice. *Gerontol* 2020; **60**: 1538–54. doi: 10.1093/geront/gnaa027.
948 van Dongen EJI, Haveman-Nies A, Wezenbeek NLW, Dorhout BG, Doets EL, de Groot L. Effect, process, and economic evaluation of a combined resistance exercise and diet intervention (ProMuscle in Practice) for community-dwelling older adults: design and methods of a randomised controlled trial. *BMC Public Health* 2018; **18**: 877.
949 University of Groningen. Appendix in the final report of the project: "The effects of physical and cognitive interventions on disability in older persons". The Netherlands: University of Groningen, 2005.
950 Van Hout H. *The cost-effectiveness of systematic home visits by nurses of frail elderly primary care patients and caregivers of demented patients*. BioMed Central: ISRCTN Registry; 2005. URL: http://www.isrctn.com/ISRCTN05358495 (accessed.
951 van Hout HP, Nijpels G, van Marwijk HW, et al. Design and pilot results of a single blind randomized controlled trial of systematic demand-led home visits by nurses to frail elderly persons in primary care [ISRCTN05358495]. *BMC Geriatr* 2005; **5**: 11.
952 Hoogendijk EO, van der Horst HE, van de Ven PM, et al. Effectiveness of a Geriatric Care Model for frail older adults in primary care: Results from a stepped wedge cluster randomized trial. *Eur J Intern Med* 2016; **28**: 43–51.
953 Muntinga ME, Hoogendijk EO, van Leeuwen KM, et al. Implementing the chronic care model for frail older adults in the Netherlands: study protocol of ACT (frail older adults: care in transition). *BMC Geriatr* 2012; **12**: 19.
954 Muntinga ME, Mokkink LB, Knol DL, Nijpels G, Jansen APD. Measurement properties of the Client-centered Care Questionnaire (CCCQ): factor structure, reliability and validity of a questionnaire to assess self-reported client-centeredness of home care services in a population of frail, older people. *Qual Life Res* 2014; **23**: 2063–72.
955 Muntinga ME, Van Leeuwen KM, Schellevis FG, Nijpels G, Jansen AP. From concept to content: assessing the implementation fidelity of a chronic care model for frail, older people who live at home. *BMC Health Serv Res* 2015; **15**: 18.
956 NL2043. *The frail elderly person at the centre of cohesive care*. Netherlands National Trial Register; 2010. URL: https://trialregister.nl/trial/2043 (accessed 11 May 2020).
957 NTR3980. *KWIEK. Investigating the effectiveness on self-management and quality of life of an integrated multidimensional lifestyle program in older adults.* Netherlands National Trial Register; 2013. URL: https://www.trialregister.nl/trial/3814 (accessed 22 May 2020).
958 van Rossum E, Frederiks C, Philipsen H, et al. Design of a Dutch study to test preventive home visits to the elderly. *Nurs Res* 1991; **40**: 185–8.
959 van Rossum HJL. Effects of preventive home visits to the elderly [thesis]. Maastricht: Rijksuniversiteit Limburg; 1993.
960 Avlund K, Vass M, Hendriksen C. Education of preventive home visitors: The effects on change in tiredness in daily activities. *Eur J Ageing* 2007; **4**: 125–31.
961 Avlund K, Vass M, Kvist K, Hendriksen C, Keiding N. Educational intervention toward preventive home visitors reduced functional decline in community-living older women. *J Clin Epidemiol* 2007; **60**: 954–62.
962 Avlund K, Vass M, Lund R, Yamada Y, Hendriksen C. Influence of psychological characteristics and social relations on receiving preventive home visits in older men and women. *Eur J Ageing* 2008; **5**: 191–201. doi: 10.1007/s10433-008-0086-4.
963 Ekmann A, Vass M, Avlund K. Preventive home visits to older home-dwelling people in Denmark: are invitational procedures of importance? *Health Soc Care Community* 2010; **18**: 563–71. doi: 10.1111/j.1365-2524.2010.00941.x.
964 Elkjaer E, Poulsen T, Avlund K. Stability and change in physical activity in old age: the role of changes in disability. *Eur J Ageing* 2006; **3**: 89–97. doi: 10.1007/s10433-006-0025-1.

965 Hendriksen C, Vass M. Preventive home visits to elderly people in Denmark. *Z Gerontol Geriatr* 2005; **38**: I/31–I/3.
966 Jørgensen TSH, Lund R, Siersma VD, Nilsson CJ. Interplay between financial assets and social relations on decline in physical function and mortality among older people. *Eur J Ageing* 2018; **15**: 133–42. doi: 10.1007/s10433-017-0437-0.
967 Kronborg C, Vass M, Lauridsen J, Avlund K. Cost effectiveness of preventive home visits to the elderly: economic evaluation alongside randomized controlled study. *Eur J Health Econ* 2006; **7**: 238–46.
968 Lund R, Nilsson CJ, Avlund K. Can the higher risk of disability onset among older people who live alone be alleviated by strong social relations? A longitudinal study of non-disabled men and women. *Age Ageing* 2010; **39**: 319–26. doi: 10.1093/ageing/afq020.
969 Nilsson CJ, Avlund K, Lund R. Social Inequality in Onset of Mobility Disability Among Older Danes: The Mediation Effect of Social Relations. *J Aging Health* 2010; **22**: 522–41. doi: 10.1177/0898264309359684.
970 Nilsson CJ, Lund R, Avlund K. Cohabitation Status and Onset of Disability Among Older Danes: Is Social Participation a Possible Mediator? *J Aging Health* 2007; **20**: 235–53. doi: 10.1177/0898264307310474.
971 Nilsson CJ, Siersma V, Mänty M, Avlund K, Vass M, Lund R. Mobility decline in old age: the combined effect of mobility-related fatigue and socioeconomic position. *J Epidemiol Community Health* 2014; **68**: 510. doi: 10.1136/jech-2013-203060.
972 Poulsen T, Elkjaer E, Vass M, Hendriksen C, Avlund K. Promoting physical activity in older adults by education of home visitors. *Eur J Ageing* 2007; **4**: 115–24. doi: 10.1007/s10433-007-0057-1.
973 Poulsen T, Siersma VD, Lund R, Christensen U, Vass M, Avlund K. Educational intervention and functional decline among older people: The modifying effects of social capital. *Scand J Public Health* 2014; **42**: 295–303. doi: 10.1177/1403494813520353.
974 Vass M, Avlund K, Hendriksen C. Randomized intervention trial on preventive home visits to older people: baseline and follow-up characteristics of participants and non-participants. *Scand J Public Health* 2007; **35**: 410–7.
975 Vass M, Avlund K, Hendriksen C, Andersen CK, Keiding N. Preventive home visits to older people in Denmark: methodology of a randomized controlled study. *Aging Clin Exp Res* 2002; **14**: 509–15.
976 Vass M, Avlund K, Kvist K, Hendriksen C, Andersen CK, Keiding N. Structured home visits to older people. Are they only of benefit for women? A randomised controlled trial. *Scand J Prim Health Care* 2004; **22**: 106–11.
977 Vass M, Avlund K, Parner ET, Hendriksen C. Preventive home visits to older home-dwelling people and different functional decline patterns. *Eur J Ageing* 2007; **4**: 107–13. doi: 10.1007/s10433-007-0059-z.
978 Vass M, Avlund K, Siersma V, Hendriksen C. A feasible model for prevention of functional decline in older home-dwelling people - The GP role. A municipality-randomized intervention trial. *Fam Pract* 2009; **26**: 56–64.
979 Vass M, Hendriksen C, Thomsen JL, Parner ET, Avlund K. Preventive home visits to home-dwelling older people and hospital admissions: a municipality-randomised intervention trial. *Eur J Ageing* 2008; **5**: 67–76.
980 Vass M, Holmberg R, Fiil-Nielsen H, Lauridsen J, Avlund K, Hendriksen C. Preventive home visitation programmes for older people: The role of municipality organisation. *Eur J Ageing* 2007; **4**: 133–40.
981 ISRCTN07330512. *Screening and Counseling for physical Activity and Mobility in Older people*. BioMed Central: ISRCTN Registry; 2005. URL: https://doi.org/10.1186/ISRCTN07330512 (accessed 21 August 2020).
982 Leinonen R, Heikkinen E, Hirvensalo M, et al. Customer-oriented counseling for physical activity in older people: study protocol and selected baseline results of a randomized-controlled trial (ISRCTN 07330512). *Scand J Med Sci Sports* 2007; **17**: 156–64. doi: 10.1111/j.1600-0838.2006.00536.x.
983 Mänty M, Heinonen A, Leinonen R, et al. Long-term Effect of Physical Activity Counseling on Mobility Limitation Among Older People: A Randomized Controlled Study. *J Gerontol A Biol Sci Med Sci* 2009; **64A**: 83–9. doi: 10.1093/gerona/gln029.
984 Rasinaho M, Hirvensalo M, Törmäkangas T, Leinonen R, Lintunen T, Rantanen T. Effect of physical activity counseling on physical activity of older people in Finland (ISRCTN 07330512). *Health Promot Int* 2011; **27**: 463–74. doi: 10.1093/heapro/dar057.
985 Sallinen J, Mänty M, Leinonen R, et al. Factors associated with maximal walking speed among older community-living adults. *Aging Clin Exp Res* 2011; **23**: 273–8. doi: 10.1007/BF03337753.
986 von Bonsdorff MB, Leinonen R, Kujala UM, et al. Effect of physical activity counseling on home care use in older people: Letters to the editor. *J Am Geriatr Soc* 2009; **57**: 571–3.
987 Avgerinou C, Gardner B, Kharicha K, et al. Health promotion for mild frailty based on behaviour change: Perceptions of older people and service providers. *Health Soc Care Community* 2019; **27**: 1333–43. doi: 10.1111/hsc.12781.

988 ISRCTN11986672. *The development and feasibility of a new service to promote health and well-being in older people who are starting to become frailer: the HomeHealth study*. World Health Organization: International Clinical Trials Registry Platform; 2015. URL: https://trialsearch.who.int/Trial2.aspx?TrialID=ISRCTN11986672 (accessed 11 May 2020).

989 ISRCTN21710246. *Occupational therapy in homecare re-ablement services*. BioMed Central: ISRCTN Registry; 2014. URL: http://www.isrctn.com/ISRCTN21710246 (accessed 28 April 2020).

990 Whitehead PJ, Drummond AE, Walker MF, et al. Occupational Therapy in HomEcare Re-ablement Services (OTHERS): study protocol for a randomized controlled trial. *Trials* 2014; **15**: 447–.

991 Williams EI, Greenwell J, Groom LM. Characteristics of patients aged 75 years and over who are discharged from hospital without district nursing support. *J Public Health Med* 1992; **14**: 321–7. doi: 10.1093/oxfordjournals.pubmed.a042749.

992 Roth G, Wolter A, Stolle C, Rothgang H. The long and bumpy road to outcome-oriented management of long-term care in Germany: implementation of the Resident Assessment Instrument in home-care services. *Int J Health Plann Manag* 2014; **29**: 316–29. doi: 10.1002/hpm.2186.

993 Stolle C, Wolter A, Roth G, Rothgang H. Effects of the Resident Assessment Instrument in home care settings: results of a cluster randomized controlled trial. *Z Gerontol Geriatr* 2012; **45**: 315–22.

994 Stolle C, Wolter A, Roth G, Rothgang H. Improving health status and reduction of institutionalization in long-term care-Effects of the Resident Assessment Instrument- Home Care by degree of implementation. *Int J Nurs Pract* 2015; **21**: 612–21.

995 NCT02286375. *Effects of Health-social Partnership Programme*. US National Library of Medicine: ClinicalTrials.gov; 2014. URL: https://clinicaltrials.gov/show/NCT02286375 (accessed 11 May 2020).

996 Wong AKC, Wong FKY. The psychological impact of a nurse-led proactive self-care program on independent, non-frail community-dwelling older adults: A randomized controlled trial. *Int J Nurs Stud* 2020; **110**: 103724. doi: 10.1016/j.ijnurstu.2020.103724.

997 Wong AKC, Wong FKY, So C. Cost-effectiveness of a preventive self-care health management program for community-dwelling older adults: a randomised controlled trial. *Age Ageing* 2020; **07**: 07. doi: 10.1093/ageing/afaa127.

998 Wong KC, Wong FK, Chang KK. Health-social partnership intervention programme for community-dwelling older adults: a research protocol for a randomized controlled trial. *J Adv Nurs* 2015; **71**: 2673–85.

999 Higgins JPT, Savović J, Page MJ, Elbers RG, Sterne JAC. Chapter 8: Assessing risk of bias in a randomized trial. In: Higgins J, Thomas J, Chandler J, et al., eds. Cochrane handbook for systematic reviews of interventions Version 6.3 (updated February 2022). Available from: www.training.cochrane.org/handbook/: Cochrane; 2022.

1000 Sterne JAC, Savović J, Page MJ, et al. RoB 2: a revised tool for assessing risk of bias in randomised trials. *BMJ* 2019; **366**: l4898. doi: 10.1136/bmj.l4898.

1001 Higgins JPT, Savović J, Page MJ, Sterne JAC, RoB2 Development Group. Revised Cochrane risk-of-bias tool for randomized trials (RoB 2), 2019.

1002 Eldridge S, Campbell MK, Campbell MJ, et al. Revised Cochrane risk of bias tool for randomized trials (RoB 2): Additional considerations for cluster-randomized trials (RoB 2 CRT) (18 March 2021). 2021. https://www.riskofbias.info/welcome/rob-2-0-tool/rob-2-for-cluster-randomized-trials (accessed 12 June 2023).
